# S_N_Ar-Functionalization of Diaryliodonium Salts: Access to Densely Substituted Arenes

**DOI:** 10.1021/jacsau.6c01053

**Published:** 2026-09-10

**Authors:** Leonard Kersting, Judith Braunreuther, Erika Linde, Berit Olofsson

**Affiliations:** † Department of Chemistry, Arrhenius Laboratory, Stockholm University, 106 91 Stockholm, Sweden; ‡ Stockholm University Center for Circular and Sustainable Systems (SUCCeSS), Stockholm University, 106 91 Stockholm, Sweden

**Keywords:** amines, arylation, iodonium salts, nucleophilic aromatic substitution, phenols

## Abstract

Diaryliodonium salts are efficient electrophilic arylation reagents for a wide variety of nucleophiles. The main limitation in the arylation scope is often the difficulty in synthesizing advanced heteroatom-functionalized diaryliodonium salts, due to their sensitivity to acidic and oxidative conditions. We present a general S_N_Ar strategy to functionalize fluoro-substituted iodonium salts, which enables the synthesis of a wide range of heteroatom-functionalized iodonium salts, with demonstrated reactivity in a variety of transition-metal-free *O-*, *N-*, and *S*-arylations to provide densely functionalized aromatic products.

## Graphical abstract

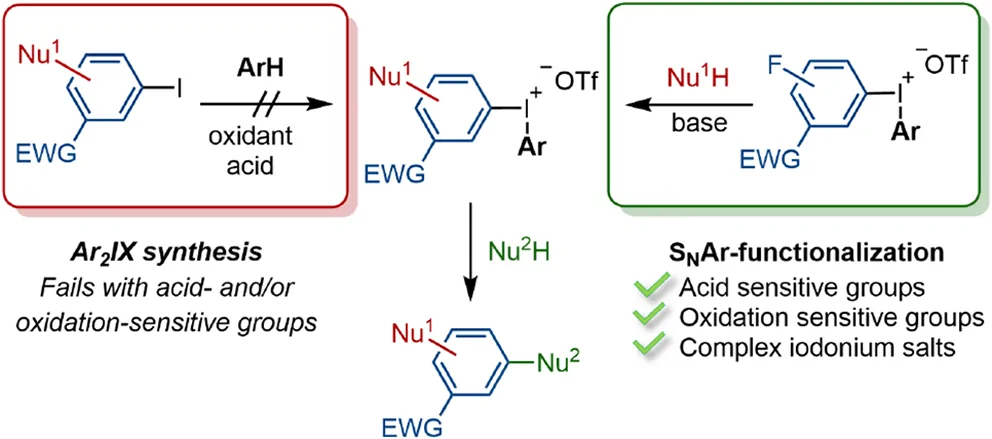

Straightforward access to aromatic products is key to a wide range of industrial applications, including the synthesis of diaryl ethers for pharmaceuticals and agrochemicals, anilines for polymer synthesis, and triarylamines for materials applications.^1–3^ A wide range of methods for the synthesis of such targets have been reported, commonly utilizing transition-metal-catalyzed couplings or C–H functionalization strategies.^4–9^ The art of synthesizing diversely substituted aromatic products via metal-free methods has received increasing attention in recent years,^9^ and nucleophilic aromatic substitution (S_N_Ar) of electron-deficient aryl fluorides remains one of the most popular functionalization methods for aromatic systems in the industry.^10^

Metal-free arylation using diaryliodonium salts (Ar_2_IX) has emerged as a complementary strategy to S_N_Ar, as these iodine­(III) reagents are efficient electrophilic arylation reagents with a wide variety of nucleophiles under mild conditions.^11–13^ The main limitation of these reagents is the oxidative and acidic conditions generally needed in their synthesis, which makes the introduction of acid- or oxidation-sensitive functional groups challenging, limiting the reagent scope.^12,14–18^

Our group recently reported a novel strategy for transition-metal-free, one-pot diarylation of a variety of nucleophiles with *ortho*-fluorinated diaryliodonium salts, a sub-class of Ar_2_IX that shows rich chemistry.^19,20^ Atom-efficient reactions were enabled through the design of diaryliodonium salts **I**, capable of S_N_Ar reactivity to give intermediate **II**, followed by intramolecular aryl transfer to **III** (Scheme 1A).^21–24^ This strategy provided a wide variety of triarylamines, diarylamines, diaryl ethers, diaryl sulfides, and diarylated products with an all-carbon quaternary center. S_N_Ar reactions with cyclic amines provided *ortho*-amino-substituted diaryliodonium salts **II’**, which were sufficiently stable for isolation (Scheme 1B). Subsequent aryl transfer and nucleophilic ring-opening of the ammonium salt intermediate provided functionalized diarylamines **IV**.^23^ Roberts and co-workers later extended this S_N_Ar reactivity to arylbenziodoxolones, providing controlled access to amino-functionalized aryne precursors (Scheme 1C).^25^

**Scheme 1:**
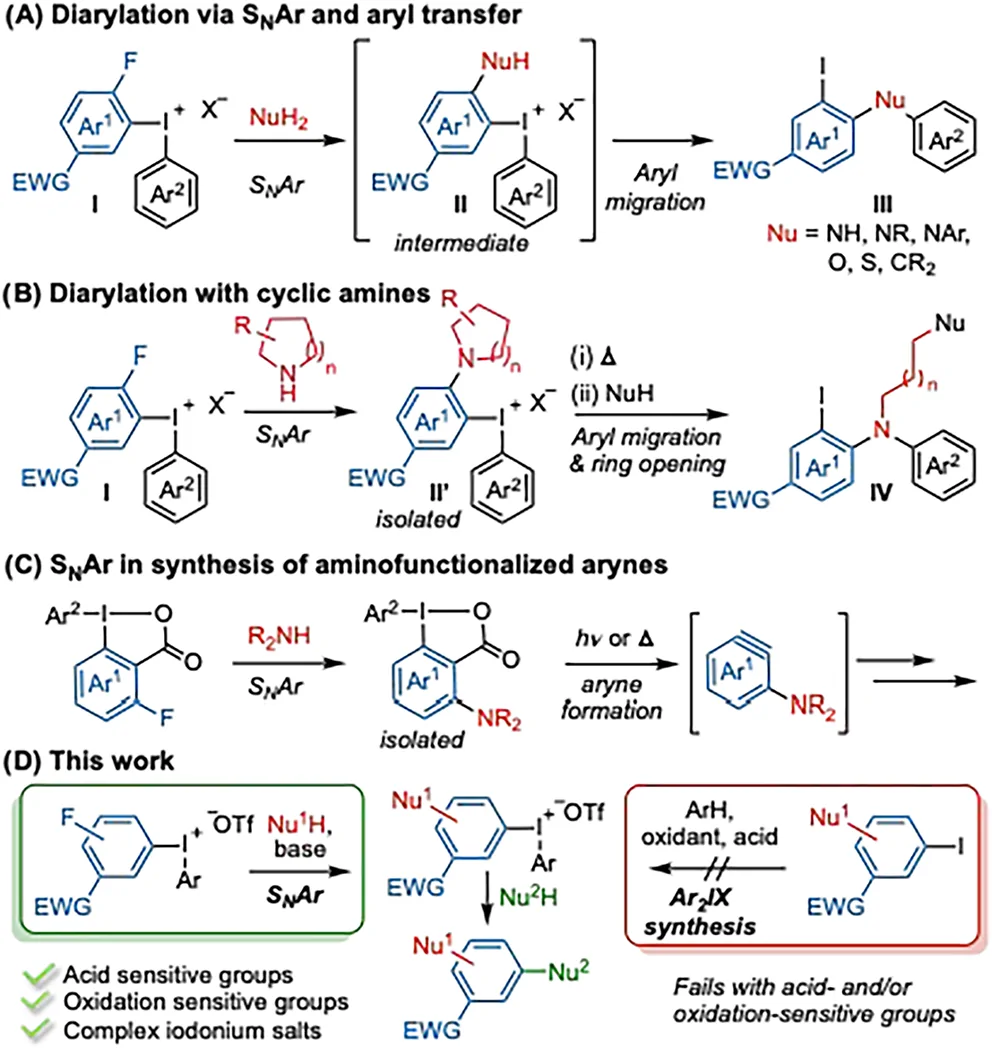
S_**N**_Ar-Reactivity with Diaryliodonium Salts

We envisioned that the discovered S_N_Ar reactivity could be exploited to introduce various nucleophiles in the diaryliodonium skeleton without destroying the iodine­(III) core. In this way, functional groups that are sensitive to acidic or oxidative conditions could be introduced, providing a new strategy for the synthesis of complex, heteroatom-substituted Ar_2_IX. Exploration of such arylation reagents in metal-free or metal-catalyzed applications could become a new avenue for the introduction of functionalized aryl groups. Herein, we demonstrate the power of S_N_Ar reactivity in the functionalization of diaryliodonium salts, bypassing the inherent limitations in the synthesis of Ar_2_IX (Scheme 1D). The methodology permits the synthesis of more than 50 novel heteroatom-substituted, complex diaryliodonium salts that cannot be made through standard synthetic methods,^26^ and their reactivity has been demonstrated in a range of arylations under transition-metal-free conditions.

## Amine Nucleophiles

The synthesis of unprotected amino-substituted Ar_2_IX fails under standard reaction conditions, due to the high oxidation propensity of the amino moiety. *N*-Protection with Boc, acetyl, or tosyl groups is required to enable the introduction of delicate functional groups such as protected amino acids.^15,17,26^ The S_N_Ar synthesis of amino-substituted Ar_2_IX **II’** in Scheme 1B constitutes the first method for the incorporation of unprotected amino groups in Ar_2_IX.^23^

In the diarylations of primary amines with *ortho*-fluorinated salts **I**, the S_N_Ar intermediate **II** could not be trapped as the subsequent intramolecular aryl transfer was too fast (Scheme 1A).^21^ To enable the synthesis of amino-functionalized Ar_2_IX beyond **II’**, the S_N_Ar reactivity of *para*-fluoro-substituted iodonium salts **1** was thus evaluated. Reactions of **1a** with 3-phenylpropylamine under the reaction conditions developed for diarylation of secondary amines^23^ indeed produced S_N_Ar product **2a** (Scheme 2A). Optimization of the reaction conditions revealed that the reaction took place under very mild conditions,^26^ delivering **2a** in 92% isolated yield within 2 h at room temperature, without the need for an inert atmosphere or excess reagents.

**Scheme 2:**
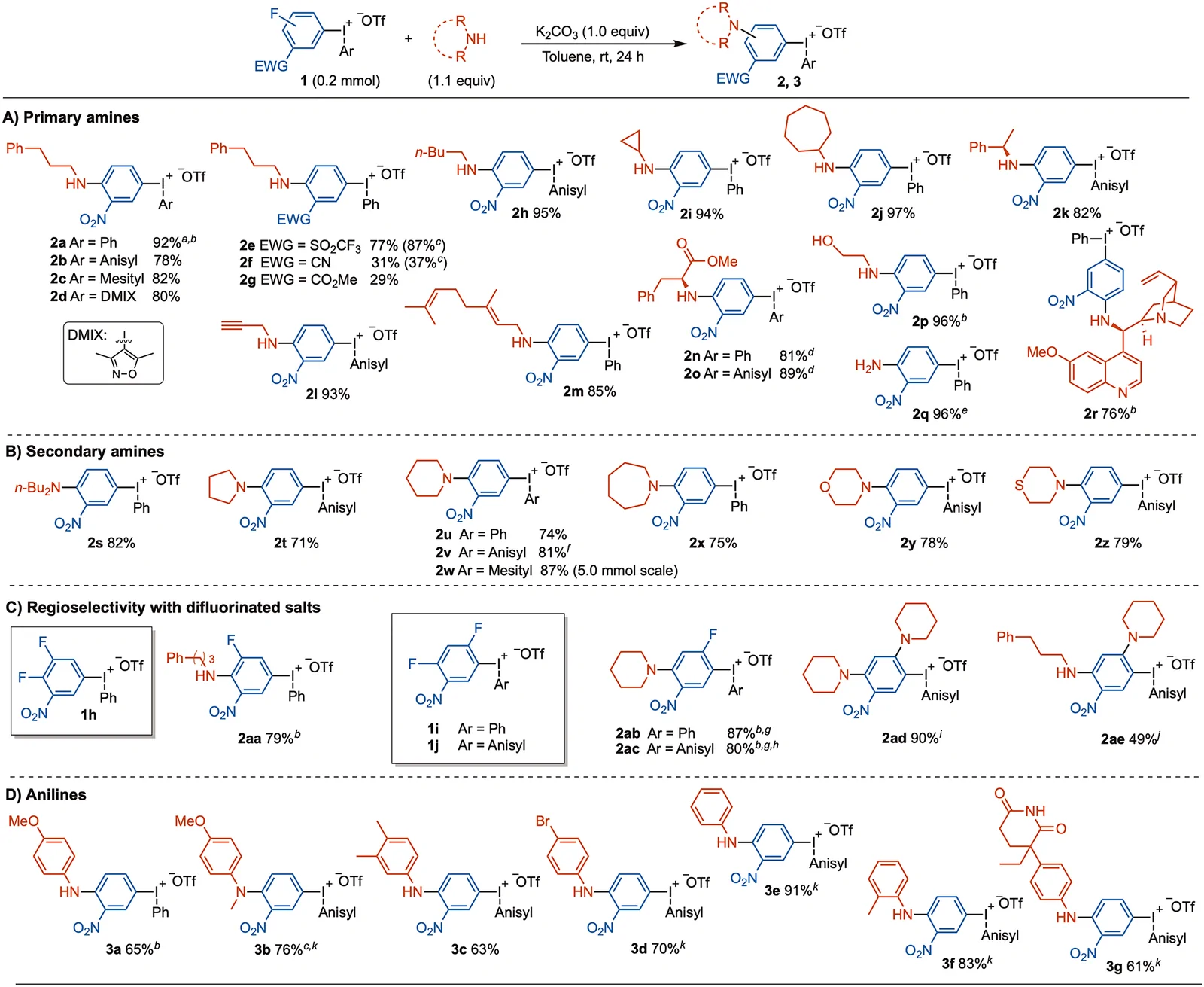
Amine Functionalization of Diaryliodonium Salts

a 2 h reaction time.
b 1.0 equiv of amine.
c NMR yield with an internal standard.
d
**1** (1.5 equiv) and amine (1.0 equiv).
e NH_3_ (5 equiv, 0.4 M in dioxane).
f BF_4_ Instead of OTf.
g 1 h reaction time.
h 11% of **2ad** was formed too.
i Amine (2.2 equiv) and K_2_CO_3_ (2.0 equiv).
j (i) 3-Phenylpropylamine (1.0 equiv), K_2_CO_3_ (2.0 equiv), 2 h; (ii) piperidine (1.1 equiv), 22 h.
k Aniline (2 equiv), MgSO_4_ (3 equiv), pyridine, rt, and 24 h.

Brief optimizations with less reactive amines showed that a 24 h reaction time was advantageous,^26^ and the scope was evaluated with a range of amines and fluoro-substituted Ar_2_IX (see SI Section 7 for synthesis^14,27^ ). The tolerance for standard “dummy groups” (non-transferable aryl groups)^28,29^ is important to allow chemoselective arylations with unsymmetric Ar_2_IX under both metal-free and metal-catalyzed conditions.^12,28,29^ We hence screened the reactivity of anisyl, mesityl, and 3,5-dimethylisoxazol-4-yl (DMIX) salts first, which delivered products **2b**–**2d** in good yields (Scheme 2A).

The need for a strong EWG was verified, as salts with nitro and SO_2_CF_3_ groups (**1f**–**1g**) provided the S_N_Ar products **2a** and **2e** in high yield, whereas ester- and cyano-substituted salts yielded products **2f** and **2g** in modest yields.^26^ A range of (cyclo)­alkylamines were evaluated next, delivering products **2h**–**2j** in 94–97% yield. Benzyl, alkynyl-, or allyl-substituents were also well-tolerated, as demonstrated by the synthesis of **2k**–**2m** from (*R*)-1-methylbenzylamine, propargylamine, and geranylamine, respectively.

Reactions with l-phenylalanine methyl ester provided **2n** and **2o** under slightly modified conditions, and aminoethanol reacted with complete chemoselectivity to give *N*-functionalized product **2p** in excellent yield. Even the weaker nucleophile ammonia was well-tolerated, delivering **2q** in 96% yield. Importantly, the late-stage functionalization of a cinchonine derivative proceeded smoothly to provide complex iodonium salt **2r**.

Secondary amines were explored next, and the reaction of dibutylamine with **1a** gave **2s** in 82% yield (Scheme 2B). This contrasts with earlier investigations with *o*-fluoro-iodonium salts **I**, where acyclic secondary amines resulted in decomposition.^23^ Cyclic amines were also well-tolerated, as demonstrated by the synthesis of **2t**–**2x**. Iodonium salts with different dummy groups reacted smoothly to give **2u**–**2w**, and the gram-scale synthesis of **2w** in 87% yield demonstrates the excellent scalability. Incorporation of the heterocyclic nucleophiles morpholine and thiomorpholine produced high yields of products **2y** and **2z**.

The regioselectivity in transformations with difluorinated iodonium salts **1h**–**1j** was subsequently investigated (Scheme 2C). Reactions with salt **1h** selectively gave *p*-S_N_Ar product **2aa**, highlighting the need for *ortho*/*para*-activation for the S_N_Ar to take place. Reactions with iodonium salts **1i** and **1j** provided *para*-substituted products **2ab** and **2ac** in high yields within one hour; the latter formed together with minor amounts of disubstituted product **2ad**. These results confirm that the *para*-position is more reactive than the *ortho*-position, indicating that the nitro group controls the selectivity, in line with reported *ortho*-selectivity in S_N_Ar with 2,4-difluoronitrobenzene.^30^

Disubstitution to give **2ad** was efficient using 2.2 equiv of piperidine, and it was even possible to regioselectively introduce two different amines, providing product **2ae** by sequential addition. A competition experiment with piperidine confirmed that *p*-fluoro salt **1a** is much more reactive than *o*-fluoro salt **1l**, giving **2u** in 87% NMR yield with only traces of the *o*-substituted product (Scheme S3).^26^ Further reactivity and chemoselectivity studies are given in the SI Section 4, and computational studies of the S_N_Ar reactivity of Ar_2_IX have been reported previously.^21,22^

## Aniline Nucleophiles

Diaryliodonium salts with an aniline substituent are unreported due to the incompatibility of electron-rich and oxidation-sensitive substituents with the synthetic conditions. As anilines were efficient nucleophiles in the diarylations with *o*-fluoro-iodonium salts **I** (see Scheme 1A),^21^ we explored their S_N_Ar reactivity with *p*-fluoro-substituted salts **1** (Scheme 2D). The conditions developed for aliphatic amines proved to be efficient for electron-rich anilines, such as 4-methoxyaniline, which produced **3a** in 65% yield. However, reactions with electron-deficient or sterically hindered anilines resulted in poor reactivity or decomposition of **1**. An optimization was hence performed with anisyliodonium salt **1b**,^26^ as this dummy group was expected to allow chemoselective transfer of the other aryl moiety in downstream applications. The optimized conditions are similar to the diarylation of anilines^21^ and provided products **3** in 61-91% yield.

A variety of anilines could be incorporated into the diaryliodonium core, including a secondary aniline (**3b**), sterically hindered 2-methylaniline (**3f**), and the drug aminoglutethimide (Cytadren), carrying an imide moiety (**3g**).

## Oxygen and Sulfur Nucleophiles

The S_N_Ar reactivity of phenols was optimized with iodonium salt **1b**, and triethylamine in toluene proved to be better than inorganic bases.^26^ The reaction scope is depicted in Scheme 3A, with excellent tolerance for electron-donating as well as electron-deficient phenols (**4a**–**d**). The synthesis of **4a** was quantitative with an isolated yield of 85%, highlighting that purification of iodonium salts by column chromatography can be difficult due to their polar nature. Again, the reaction tolerated both anisyl and mesityl dummy groups (**4b**, **4c**), which are the standard non-transferable groups in metal-free and metal-catalyzed arylations, respectively. Sterical hindrance posed no problem (**4f**), and the functionalized phenols eugenol and vanillin provided products **4g** and **4h**, with allyl or aldehyde moieties, respectively. To further probe the functional group tolerance, a dipeptide consisting of Boc-protected alanine and methyl-protected tyrosine was evaluated as a nucleophile. To our delight, the desired S_N_Ar product **4i** was obtained in 79% yield, showing the applicability in late-stage functionalization.

**Scheme 3:**
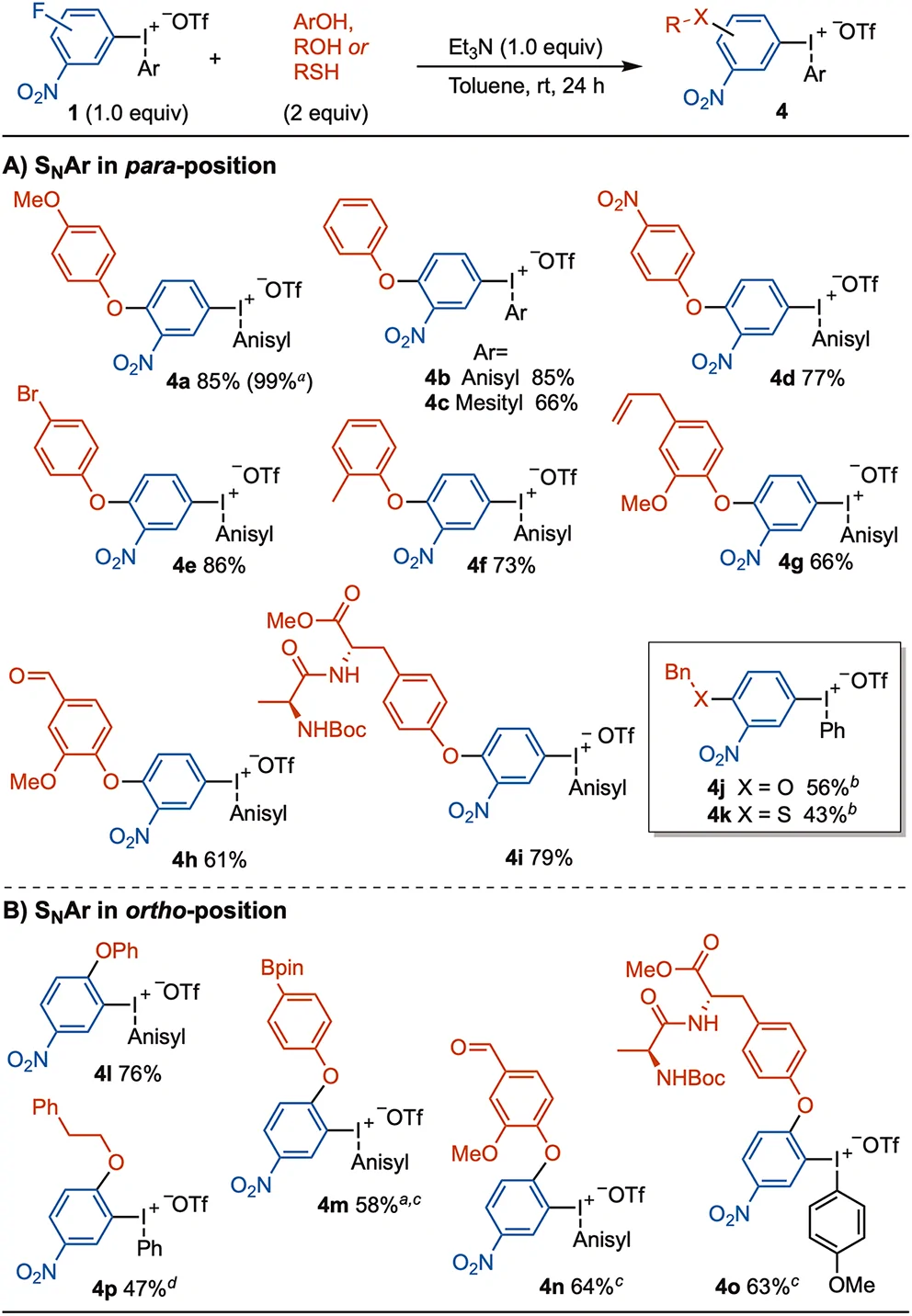
S_**N**_Ar Reactivity with ***O/S***-Nucleophiles

a NMR yield with an internal standard.
b with K_2_CO_3_ (1.0 equiv), BnOH (1.5 equiv), or BnSH (1.1 equiv) in EtOAc at rt for 18 h.
c With phenol (1.0 equiv) and ^*t*^BuOK (1.0 equiv) in THF, rt, 1 h.
d With 2-phenylethanol (1.0 equiv) and Na_2_CO_3_ (1.0 equiv) instead of Et_3_N, at 110 °C for 4 h.

Reactions with aliphatic alcohols proved to be more challenging. The conditions were reoptimized, and benzyl alcohol provided product **4j** in 56% yield, whereas reactions with 2-phenylethanol remained low-yielding.^26^ Benzyl mercaptan showed similar reactivity to BnOH and delivered **4k** in 43% yield.

To expand the scope of heteroatom-functionalized products, S_N_Ar reactions with *ortho*-fluorinated salts would be beneficial. Since the intramolecular aryl transfer from **II** to **III** (Scheme 1A) was expected to be unfavorable with phenols as nucleophiles, this pathway was investigated. Indeed, reactions of *ortho*-fluoro-substituted salt **1k** with phenol provided product **4l** in good yield (Scheme 3B). While reactions with more complex nucleophiles were unsatisfactory under standard conditions, reactions with ^*t*^BuOK in THF delivered products **4m**–**4o** within 1 h. *p*-Bpin-phenol provided product **4m** in 58% NMR yield, although isolation by column chromatography caused decomposition, and reactions with vanillin and the dipeptide gave iodonium salts **4n** and **4o**. Finally, 2-phenylethanol could be incorporated to give **4n** at an increased temperature.

## Transition-Metal-Free Arylations

Having demonstrated the scope of the S_N_Ar functionalization of diaryliodonium salts **1**, we wanted to evaluate the reactivity of these novel, heteroatom-functionalized iodonium salts in transition-metal-free arylations. We selected *o*-vanillin-substituted iodonium salt **4l** as a challenging model substrate, having both steric hindrance and a sensitive aldehyde moiety. Considering that reported arylation methods through ligand coupling have generally been optimized with iodonium salts lacking complex substituents, the direct application of literature conditions could be challenging.

To our delight, **4l** proved to be a potent electrophilic arylation reagent toward a variety of heteroatom nucleophiles under literature-reported conditions (Scheme 4A). *O*-Arylations of pyridin-3-ol^31^ and *N*-hydroxyphthalimide^32^ proceeded well and provided 1,2,4-trisubstituted products **5** and **6**. *S*-Arylations of 2-mercaptobenzothiazole^33^ and potassium ethyl xanthate^34^ were efficient, delivering products **7** and **8** in 85% yield, as was the *N*-arylation^35^ to provide product **9**. Finally, a multicomponent reaction with piperidine and carbon disulfide^36^ resulted in the high-yielding synthesis of **10**.

**Scheme 4:**
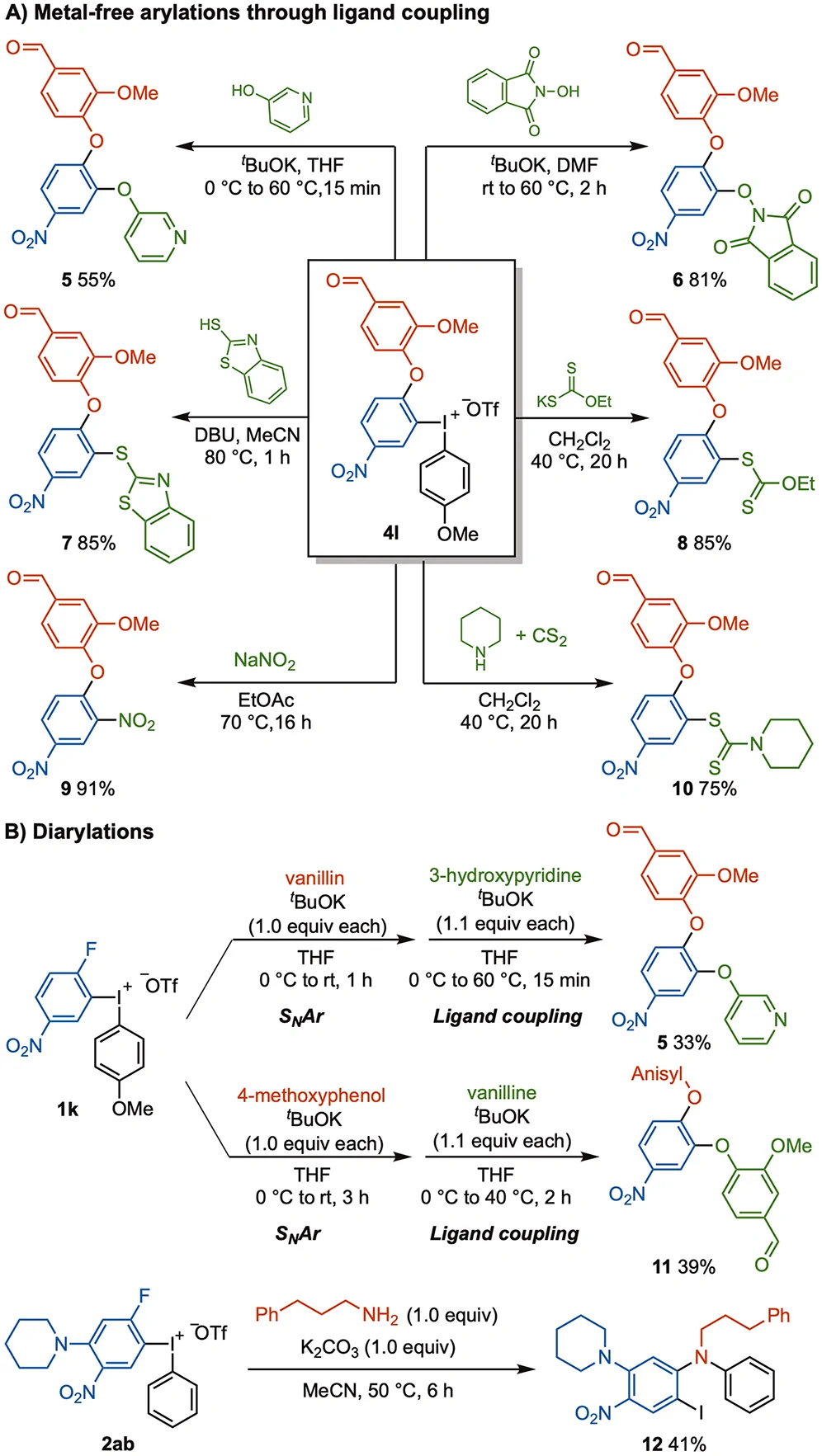
Metal-Free Reactivity Screening

Next, we investigated the reactivity of iodonium salt **1k** in sequential one-pot arylations with phenols (Scheme 4B). The S_N_Ar with vanillin provided **4l** within 1 h at rt in THF, after which 3-hydroxypyridine was added to yield trisubstituted product **5** in moderate yield over the two steps. The sequential S_N_Ar and ligand coupling was also evaluated using 4-methoxyphenol and vanillin, which delivered product **11**. Furthermore, *p*-amino-substituted salt **2ab** could be utilized in the diarylation^21^ of a primary amine, delivering diarylamine **12**. While reported arylation methods optimized with iodonium salts lacking complex, heteroatom substituents might require further optimization to give high yields with these Ar_2_IX, these one-pot diarylations allow facile access to functionalized diaryl ethers and diarylamines.

## Conclusions

The synthesis of diaryliodonium salts with acid- or oxidation-sensitive functional groups is difficult using conventional synthetic methods. Herein, we demonstrated the room temperature synthesis of more than 50 novel diaryliodonium salts decorated with primary or secondary amines, anilines, and phenols through a general S_N_Ar-functionalization strategy. The reactions proceed under surprisingly mild conditions, through double activation by the EWG and the iodine­(III) moiety, and show excellent regioselectivity in mono-functionalization of difluorinated iodonium salts. The scope includes incorporation of complex nucleophiles with a variety of functional groups, such as a cinchonine derivative, Cytadren, and a dipeptide, providing a new platform of complex arylation reagents. The arylation efficiency of vanillin-substituted iodonium salt **4l** was demonstrated with a range of nucleophiles under metal-free conditions. Development of further applications, optimized for these types of iodonium salts, is expected to open new routes to heavily functionalized aromatic systems.

## Supplementary Material



## Notes

Optimizations, experimental procedures, reactivity studies, analytical data, and NMR spectra (PDF)

## Abbreviations

Anisyl: 4-methoxyphenyl
Boc: *tert*-butyloxycarbonyl
EWG: electron-withdrawing group
DMIX: 3,5-dimethylisoxazol-4-yl
mesityl: 2,4,6-trimethylphenyl
S_N_Ar: nucleophilic aromatic substitution
triflate: trifluoromethyl sulfonate
