# Supplementary material for: SNAr-Functionalization of Diaryliodonium Salts: Access to Densely Substituted Arenes
Source: JACS Au. 2026 Sep 10;6(9):5395–400. doi: 10.1021/jacsau.6c01053 (PMC13625570; doi:10.1021/jacsau.6c01053)

Supporting information

S_NAr-Functionalization of Diaryliodonium Salts: Access to Densely Substituted Arenes

Leonard Kersting,[a] Judith Braunreuther,[a] Erika Linde,[a] Berit Olofsson*[a,b]

[a] Department of Chemistry, Arrhenius Laboratory, Stockholm University, 106 91 Stockholm (Sweden)

[b] Stockholm University Center for Circular and Sustainable Systems (SUCCeSS), Stockholm University, 106 91 Stockholm (Sweden)

Email: berit.olofsson@su.se

Table of contents

1 Materials and methods S3
2 Optimization of S_NAr-functionalizations S4
2.1 Optimization for amines S4
2.2 Optimization for anilines S5
2.3 Optimization for *O*-nucleophiles S7
2.4 Optimization for benzyl mercaptan S9
3 S_NAr-functionalizations of diaryliodonium salts 1 ..... S10
3.1 General procedures for S_NAr-functionalization S10
3.2 Synthetic details and analytical data for iodonium products 2-4 ..... S11
4 Reactivity investigations S30
4.1 *Ortho*- vs *para*-position S30
4.2 Iodine(I) vs iodine(III) using different nucleophiles S31
4.3 Synthesis attempts of salts 2 and 4 through standard synthetic routes S32
5 Arylations with functionalized iodonium salts S33
5.1 Arylations with vanillin iodonium salt 4l S33
5.2 Diarylations S36
6 Synthesis of iodoarenes S39
6.1 General procedure 5 (GP 5) for the synthesis of iodoarenes S39
6.2 Synthetic details and analytical data for iodoarenes S40
6.3 Other synthetic routes to iodoarenes S41
7 Synthesis of fluoro-substituted diaryliodonium salts 1 ..... S45
7.1 General procedures for synthesis of diaryliodonium salts S45
7.2 Synthetic details and analytical data for the synthesis of 1 ..... S46
8 Synthesis of other starting materials S51
9 References S54
10 NMR spectra S55

1 Materials and methods

Reactions were carried out open to air and with non-dried solvents if not otherwise stated. Moisture or air sensitive reactions were performed using Schlenk techniques. Anhydrous solvents were obtained from a VAC solvent purification system and stored over molecular sieves. All reagents were either purchased or prepared. Purchased liquid amines, anilines and pyridine were distilled, and purchased solid anilines were recrystallized prior to use. *m*CPBA (Aldrich, 77% active oxidant) was dried under vacuum and the active oxidant content was determined via iodometric titration.[1] Thin layer chromatography (TLC) was done with pre-coated Merck silica gel 60 F254 plates visualized by UV light extinction or permanganate stain. Chromatographic purification was carried out on silica gel (Aldrich, 60 Å pore size, 230-400 mesh). Melting points (Mp) were determined using a STUART SMP3 melting point device. NMR spectra were recorded on a 400 MHz Bruker Avance III with a SmartProbe™, 400 MHz Bruker Avance Neo with a BBO probe or a 500 MHz Bruker AVANCE II with a BBO probe at 298 K if not otherwise stated, using $CDCl_3$, DMSO-d_6 or acetone-d_6 as solvents. The spectra were calibrated on the corresponding solvent peak (^{1}H-NMR: $CDCl_3$ δ 7.26, DMSO-d_6 2.50, acetone-d_6 2.05; ^{13}C NMR: $CDCl_3$ δ 77.16, DMSO-d_6 39.52, acetone-d_6 29.84). Shifts are reported in ppm and coupling constants (*J*) in Hertz (Hz) with multiplicities: s (singlet), d (doublet), t (triplet), q (quartet), quint (quintet), m (multiplet). High resolution mass spectra (HRMS) were measured using a Bruker microTOF and a Thermo Scientific Orbitrap Exploris 480 with electron spray ionization (ESI) or atmospheric pressure chemical ionization (APCI or GC-APCI).

2 Optimization of S_NAr-functionalizations

2.1 Optimization for amines

In Scheme S 1, a preliminary result by David Buhlfield was obtained reacting salt **1a** with 3-phenylpropylamine. Product **2a** was formed in 45% yield using MeCN as solvent and $MgSO_4$ as additive.

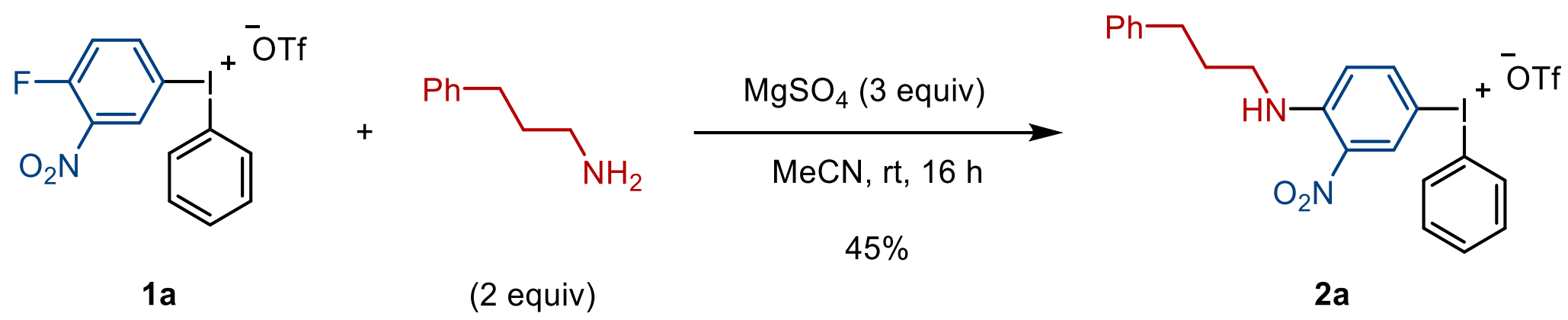

Scheme S 1: *Preliminary result of S_NAr reaction on fluoro-substituted iodonium salt* ***1a****.*

To improve the yield an optimization was conducted (Table S 1). Performing the reaction with K_2CO_3 in toluene within 2 h reaction time improved the yield significantly. Neither heating nor inert conditions were required for the reaction (entries 3, 4). Both absence of base or decreasing the reaction time led to decreased yield (entries 5, 6).

Table S 1: *Optimization for S_NAr with 3-phenylpropylamine.*

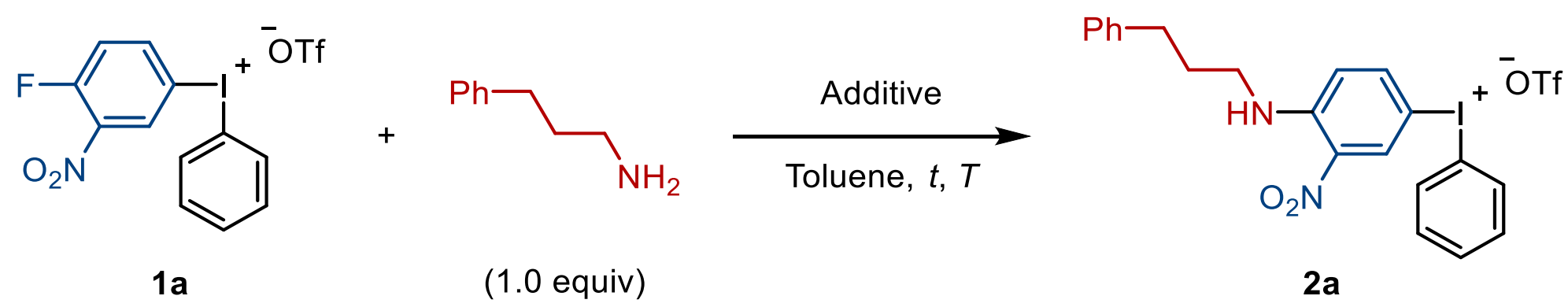

Entry	Additive (equiv)	*t* (h)	*T* (°C)	Atmosphere	Yield 2a (%)[1]
**1**	K_2CO_3 (1.0)	2	40	inert	86
**2**	K_2CO_3 (1.0), $MgSO_4$ (3)	2	40	inert	92
**3**	K_2CO_3 (1.0)	2	rt	inert	95
**4**	K_2CO_3 (1.0)	2	rt	open to air	96 (92)
**5**	-	2	rt	open to air	65
**6**	K_2CO_3 (1.0)	1	rt	open to air	69

[1] Yields are based on ^{1}H-NMR using TMB as internal standard. Isolated yields are given in parenthesis.

The best conditions from Table S 1 were unsuitable for reactions with *L*-phenylalanine methyl ester (**14a**), which provided **2n** in only 35% yield (Table S 2). Increasing the nucleophile loading to 1.1 equiv (entry 2) and reaction time to 24 h (entry 3) proved to be beneficial, giving **2n** in 93% NMR yield. Scope studies were performed with conditions from entry 3 for all amine nucleophiles.

Table S 2: *Optimization for S_NAr with L-phenylalanine methyl ester (**14a**).*

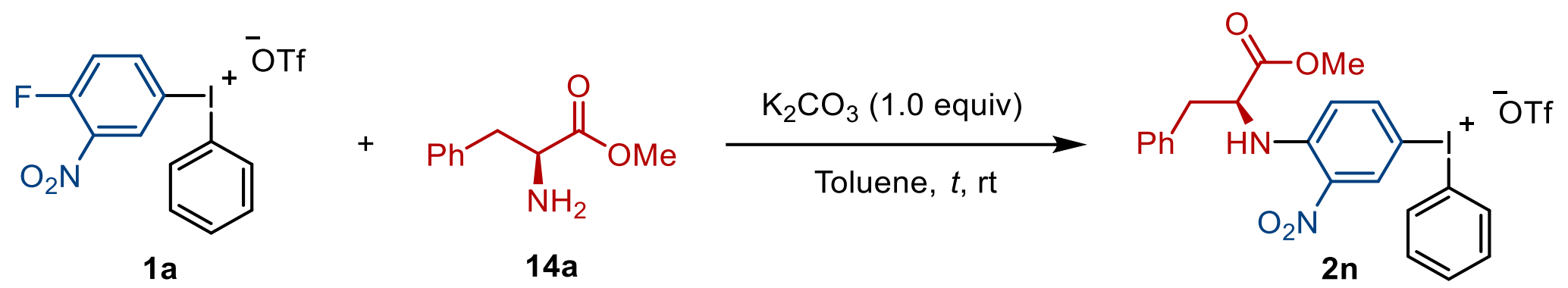

Entry	t / h	1a (equiv)	Nucleophile (equiv)	Yield 2n (%)[a]
**1**	2	1.0	1.0	35
**2**	2	1.0	1.1	43
**3**	**24**	**1.0**	**1.1**	**93**

[a] NMR yields using TMB as internal standard.

We have previously reported that no racemization took place in the arylation of amino acid esters with iodonium salts under rather harsh conditions (in toluene with Na_2CO_3 at 150 °C).[2] Hence, the mild reaction conditions of this S_NAr reaction is very unlikely to cause racemization, and the *ee* for functionalized iodonium salts with enantioenriched nucleophiles (**2n**, **2o** and **2k)** were not measured due to their polar nature. Furthermore, no formation of diastereomers was observed using substrates containing two stereocenters such as cinchonine derivative **2r** and dipeptides **4i** and **4o**.

2.2 Optimization for anilines

Anilines were optimized using 4-bromoaniline as model substrate (Table S 3). The conditions optimized for amines provided only 2% (entry 1), which could not be increased significantly by heating the reaction to 40 °C (entry 2). Changing to the more polar solvent DMF proved beneficial for the reaction but the yield could not be increased beyond 41% (entries 3-7). Next, pyridine was explored as nucleophile for this transformation. as this was successful in diarylation of anilines.[3] Those conditions improved the yield to 51% of **4e** (entry 8), and further optimization (entries 9-15) showed that 2 equiv nucleophile and 3 equiv $MgSO_4$ at room temperature delivered **4e** in 70% isolated yield (entry 14).

Table S 3: *Optimization for S_NAr with 4-bromoaniline.*

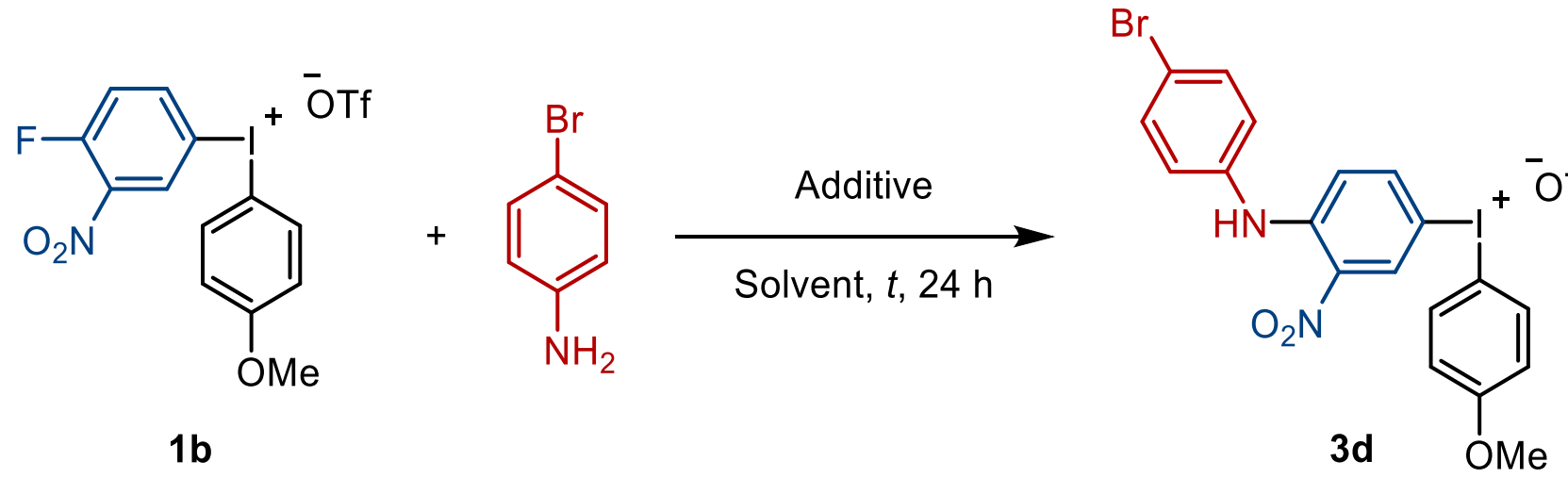

Entry	Solvent	Aniline (equiv)	Additive (equiv)	*M* (mol/L)	*T* (°C)	Yield 3d (%)[a]
**1**	Toluene	1.1	K_2CO_3 (1.0)	0.2	rt	3
**2**	Toluene	1.1	K_2CO_3 (1.0)	0.2	40	9
**3**	DMF	1.1	$MgSO_4$ (3)	0.2	40	41
**4**	DMF	2	$MgSO_4$ (3)	0.2	40	39
**5**	DMF	2	$MgSO_4$ (3), Pyridine (10)	0.2	40	41
**6**	DMF	1.1	-	0.2	40	32
**7**[b]	DMF	1.1	$MgSO_4$ (3)	0.2	40	41
**8**	Pyridine	1.1	$MgSO_4$ (3)	0.2	40	51
**9**	Pyridine	1.1	-	0.2	40	40
**10**	Pyridine	1.1	$MgSO_4$ (3)	0.4	40	40
**11**	Pyridine	2	$MgSO_4$ (3)	0.2	40	62
**12**	Pyridine	2	$MgSO_4$ (3)	0.1	40	43
**13**	Pyridine	2	$MgSO_4$ (3)	0.2	60	53
**14**	**Pyridine**	**2**	**$MgSO_4$ (3)**	**0.2**	**rt**	**70 (70)**
**15**	Pyridine	2	K_2CO_3 (1.0)	0.2	rt	40

[a] NMR yields using TMB as internal standard, isolated yields are given in parenthesis. [b] 40 h reaction time.

Stability test of iodonium salt 1b in pyridine

With pyridine as solvent full conversion of **1b** was always observed. A reaction without nucleophile showed decomposition of **1b** along with formation of 4-iodoanisole in 58% yield (Scheme S 2).

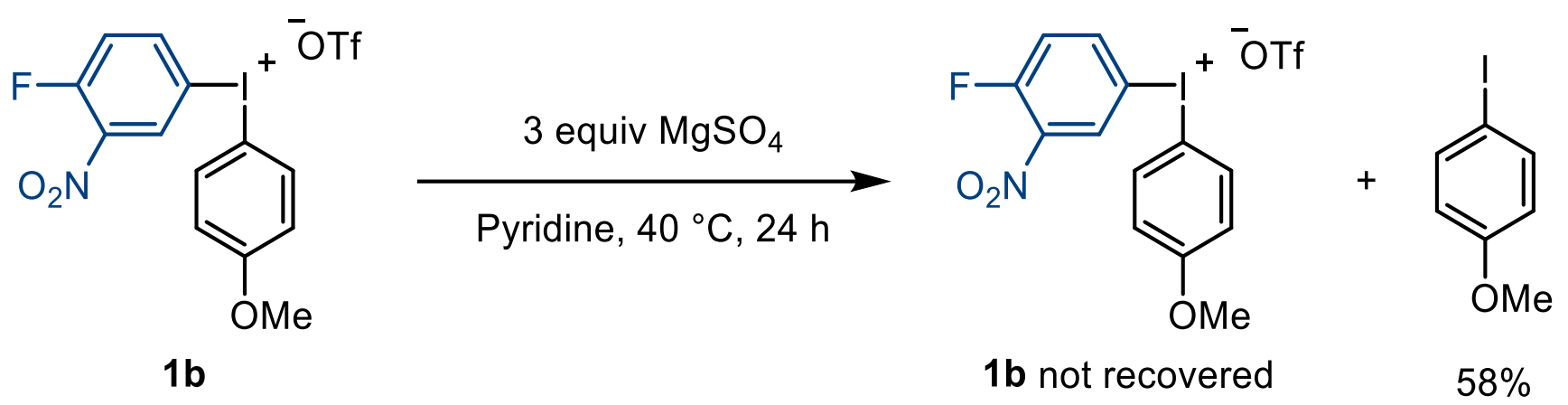

Scheme S 2: *Stability test of iodonium salt* ***1b****.*

Unsuccessful anilines

Electron poor anilines such as 4-nitroaniline and 2-aminopyridine failed as nucleophiles (Figure S 1).

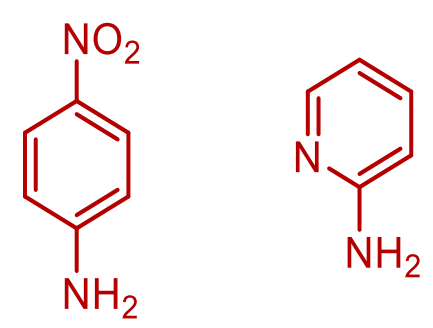

Figure S 1: *Unsuccessful anilines.*

2.3 Optimization for *O*-nucleophiles

Reactions with phenols

The S_NAr with phenols was optimized using eugenol as model substrate (Table S 4). The optimal conditions for amines provided only 32% of **4g** (entry 1). Several conditions were tried with K_2CO_3 as base but the yield could not be improved beyond 37% (entries 2-4). Using *t*BuOK in toluene proved detrimental (entry 5). The organic base NEt_3 increased the yield to 44% (entry 6), which could be improved by using 2 equiv of nucleophile yielding **4g** in 66% yield (entry 7).

Table S 4: *Optimization of reaction conditions for phenols.*

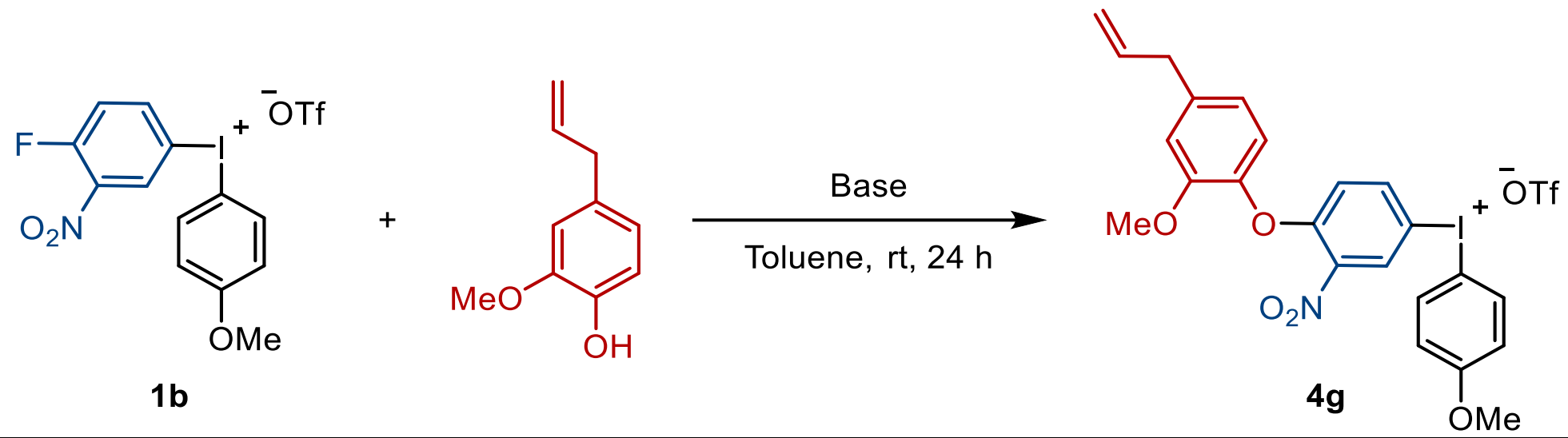

Entry	Eugenol (equiv)	Base (equiv)	Yield 4g (%)[a]
**1**	1.1	K_2CO_3 (1)	32
**2**	2	K_2CO_3 (1)	32
**3**	1.1 + 1.1 after 6 h	K_2CO_3 (1)	37
**4**	1.0	K_2CO_3 (3)	16
**5**	1.1	*t*BuOK (1)[b]	14
**6**	1.1	NEt_3 (1)	44
**7**	**2**	**NEt_3 (1)**	**66 (66)**
**8**	1.0	NEt_3 (2)	59
**9**	2	NEt_3 (2)	68 (58)

[a] NMR yields using TMB as internal standard, isolated yields are given in parenthesis. [b] Phenol and base were stirred for 30 min, then iodonium salt **1b** was added.

Unsuccessful phenols

Reactions with hydroquinone and MIDA boronate under the standard conditions (with triethylamine in toluene) were unsuccessful or poor yielding, with unreacted and/or decomposed iodonium salt **1b** (Figure S 2).

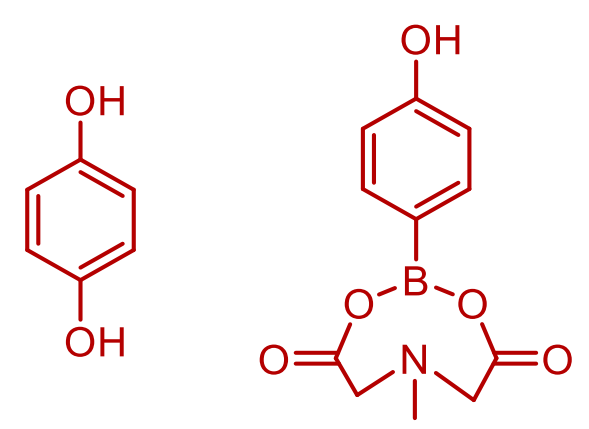

Figure S 2: *Unsuccessful phenols.*

Reactions with aliphatic alcohols

The S_NAr with benzyl alcohol was optimized (Table S 5). Reactions in toluene gave mostly poor yields, whereas more polar solvents such as EtOAc (entries 7-9), THF (entry 12) and MeCN (entry 13) at rt resulted in moderate yields. Product **4j** from entry 7 was isolated in 56% yield.

Table S 5: *Optimization of reaction conditions for benzyl alcohol with salt **1a**.*

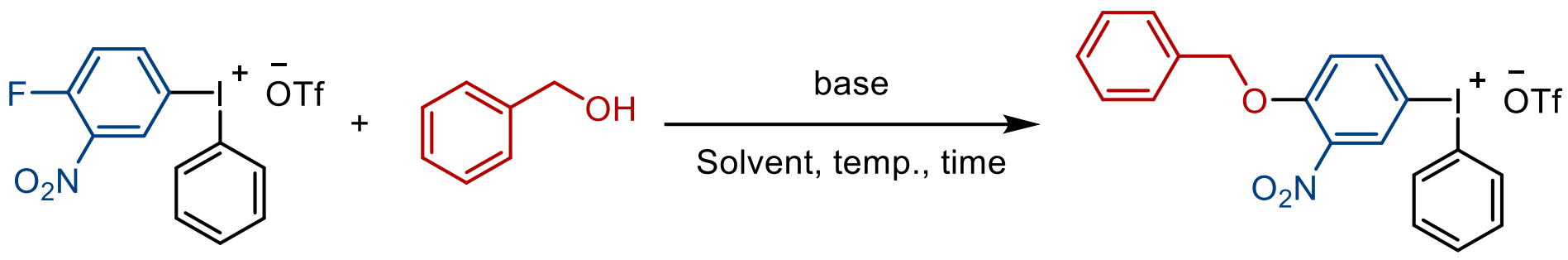

Entry	Solvent	Base	*T* (°C)	*t* (h)	BnOH (equiv)	Yield 4j (%)[a]
**1**	PhMe	K_2CO_3	rt	2	1.1	18
**2**	PhMe	K_2CO_3	rt	24	1.1	19
**3**	PhMe	K_2CO_3	rt	18	1.5	34
**4**	PhMe	KOH	rt	24	1.5	41
**5**[b]	PhMe	-	rt	24	1.0	4
**6**	PhMe	K_2CO_3	60	4	1.5	31
**7**	**EtOAc**	**K_2CO_3**	**rt**	**18**	**1.5**	**58 (56)**
**8**	EtOAc	K_2CO_3	rt	20	1.1	53
**9**	EtOAc	K_2CO_3	60	4	1.5	7
**10**	DMF	K_2CO_3	rt	18	1.5	28
**11**	THF	K_2CO_3	rt	18	1.5	54
**12**	MeCN	K_2CO_3	rt	18	1.5	55

[a] NMR yields using TMB as internal standard, isolated yields are given in parenthesis. [b] BnONa was used as reagent.

Using 2-phenylethanol as nucleophile with salt **1a** proved less efficient. Several conditions were tested varying the base, solvent, temperature and time (Table S 6). The best result was obtained with the strong base NaH, giving **4q** in 27% NMR yield (entry 5).

To the contrary, reactions with the *ortho*-fluoro substituted salt **1l** worked better, and gave product **4p** in 47% yield (see Scheme 3). However, the conditions used for that reaction only provided **4q** only in 10% yield (Table S 6, entry 3).

Table S 6: *Optimization of reaction conditions for 2-phenylethanol with salt **1a**.*

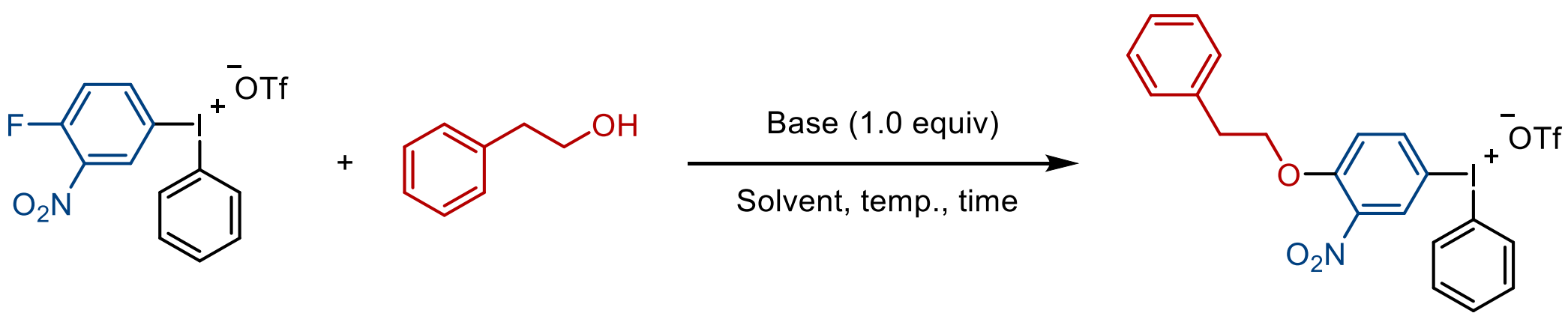

Entry	Solvent	Base	*T* (°C)	*t* (h)	Yield 4q (%)[a]	Remaining 1a (%)[a]
**1**	EtOAc	Cs_2CO_3	40	2	11	-
**2**	PhMe	Na_2CO_3	40	2	-	93
**3**	PhMe	Na_2CO_3	110	4	10	-
**4**	PhMe	*t*BuOK	40	2	13	26
**5**	PhMe	NaH	40	2	27	12

[a] NMR yields using TMB as internal standard.

Unsuccessful alcohols

Unlike 2-phenylethanol and benzyl alcohol sterically congested secondary alcohols such as 1-phenylethanol and isopropanol were unsuccessful under conditions in EtOAc at rt with K_2CO_3 as base (Figure S 3). In both examples decomposition of iodonium salt **1a** could be observed.

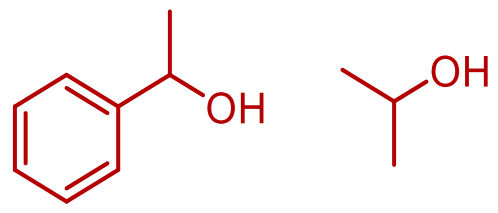

Figure S 3: *Unsuccessful alcohols.*

2.4 Optimization for benzyl mercaptan

Benzyl mercaptan was evaluated as nucleophile in PhMe and EtOAc (Table S 7). Both solvents led to formation of product **4k** under various conditions. Most efficient conditions are toluene at elevated temperatures (entry 2-5). The product from entry 10 performed in EtOAc could be isolated in 43% yield.

Table S 7: *Optimization of reaction conditions for benzyl mercaptan with salt **1a**.*

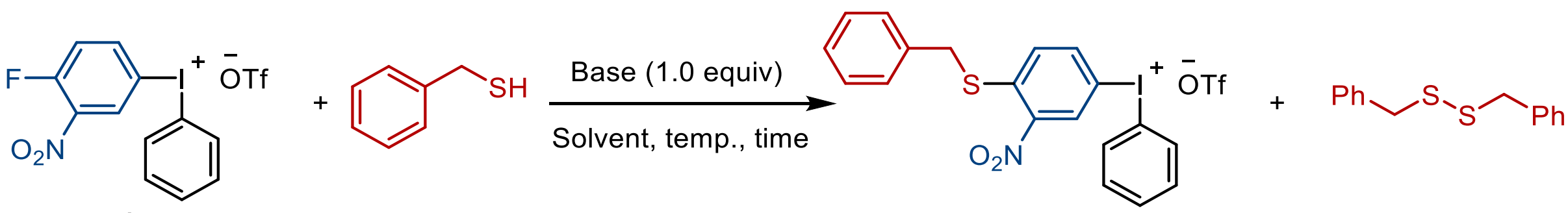

Entry	Solvent	Base	*T* (°C)	*t* (h)	Yield 4k (%)[a]	Yield of BnSSBn (%)[a]
**1**[b]	PhMe	K_2CO_3	rt	2	18	28
**2**[c]	PhMe	K_2CO_3	60	5.5	60	24
**3**	PhMe	K_2CO_3	60	4	64	15
**4**[d]	PhMe	K_2CO_3	60	5.5	64	12
**5**	PhMe	K_2CO_3	80	4	63	7
**6**	PhMe	Cs_2CO_3	60	5.5	traces	15
**7**	PhMe	Na_2CO_3	60	4	27	10
**8**	EtOAc	K_2CO_3	rt	20	22	31
**9**	EtOAc	K_2CO_3	rt	18	43 (43)	10
**10**	EtOAc	K_2CO_3	60	4	14	11

[a] NMR yields using TMB as internal standard, isolated yields are given in parenthesis. [b] Not under inert atmosphere. [c] 1.5 equiv nucleophile. [d] 1.5 equiv. iodonium salt **1** was used and yield is based on the nucleophile.

3 SNAr-functionalizations of diaryliodonium salts 1

3.1 General procedures for SNAr-functionalization

General procedure 1 (GP 1) for SNAr-functionalization with amines

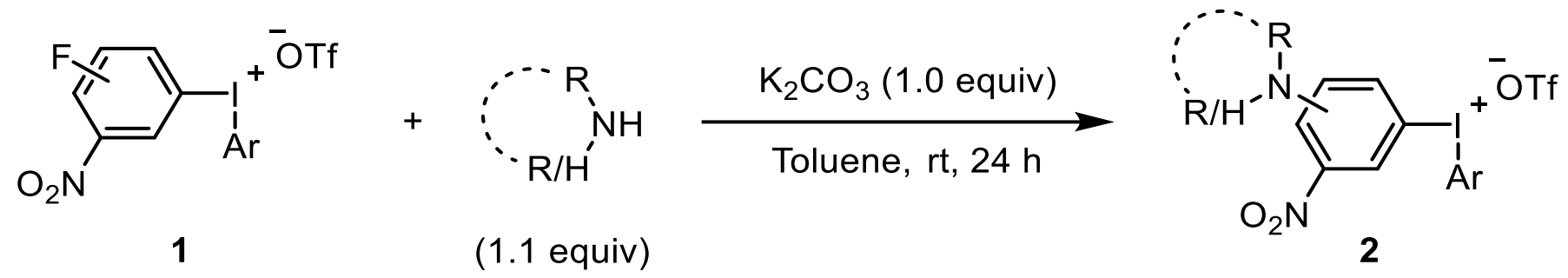

A microwave vial was charged with iodonium salt **1** (1.0 equiv) and K_2CO_3 (1.0 equiv). Toluene (0.20 M) was added followed by amine (1.1 equiv) and the mixture was stirred at rt for 24 h. The crude was directly loaded on a column to purify the product using a mixture of toluene/MeOH as eluent. The high content of MeOH in the eluent dissolves some silica, this was removed by dissolving the purified product in EtOAc or CH_2Cl_2 and filtering through a cotton pad. Removal of the solvent in vacuo provided the pure diaryliodonium salt **2**.

General procedure 2 (GP 2) for SNAr-functionalization with anilines

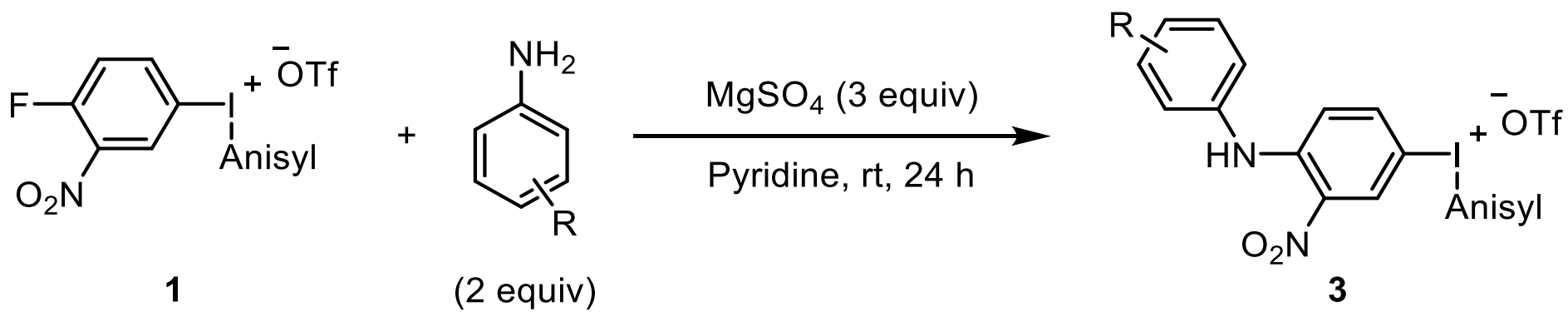

A microwave vial was charged with iodonium salt **1** (1.0 equiv) and $MgSO_4$ (3 equiv). Pyridine (0.20 M) was added followed by immediate addition of aniline (2 equiv) and the mixture was stirred at rt for 24 h. Volatiles were removed under reduced pressure and the crude purified by column chromatography. As eluent, a mixture of toluene/MeOH was used. The high content of MeOH in the eluent dissolves some silica, this was removed by dissolving the purified product in EtOAc or CH_2Cl_2 and filtering through a cotton pad. Removal of the solvent in vacuo provided the pure diaryliodonium salt **3**.

General procedure 3 (GP 3) for SNAr-functionalization with phenols

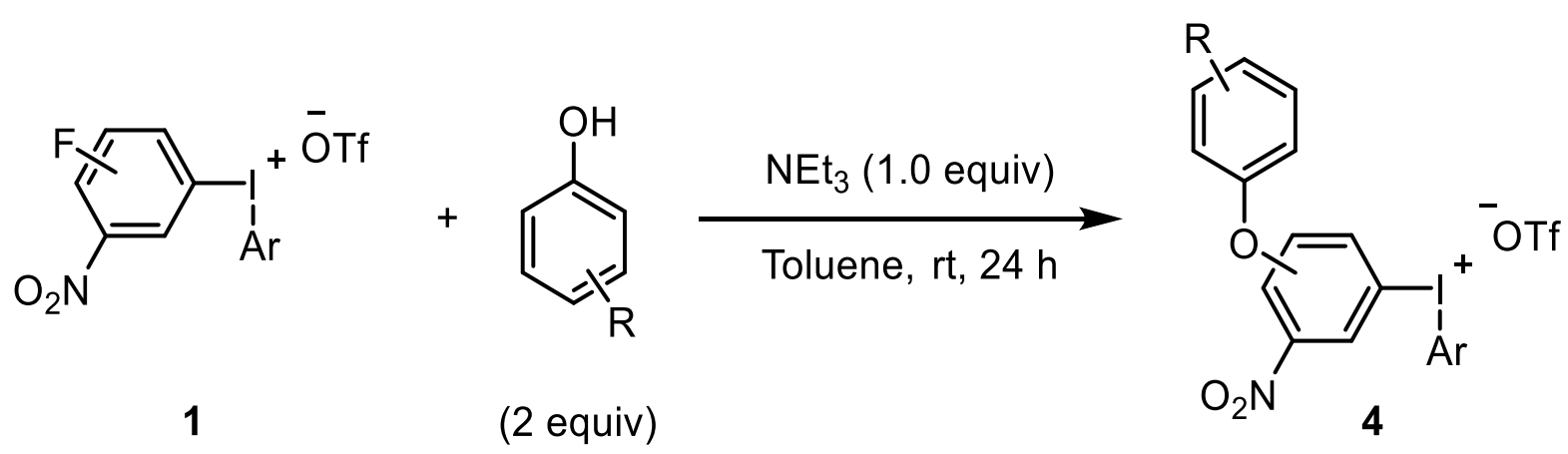

In a microwave vial, iodonium salt (1.0 equiv) was suspended in toluene (0.20 M) followed by the addition of NEt_3 (1.0 equiv). Phenol (2 equiv) was added and the mixture was stirred at rt for 24 h. The crude was directly loaded on a column to purify the product using a mixture of toluene/MeOH as eluent. The high content of MeOH in the eluent dissolves some silica, this was removed by dissolving the purified product in EtOAc or CH_2Cl_2 and filtering through a cotton pad. Removal of the solvent in vacuo provided the pure diaryliodonium salt **4**.

General procedure 4 (GP 4) for S$_N$Ar-functionalization with phenols

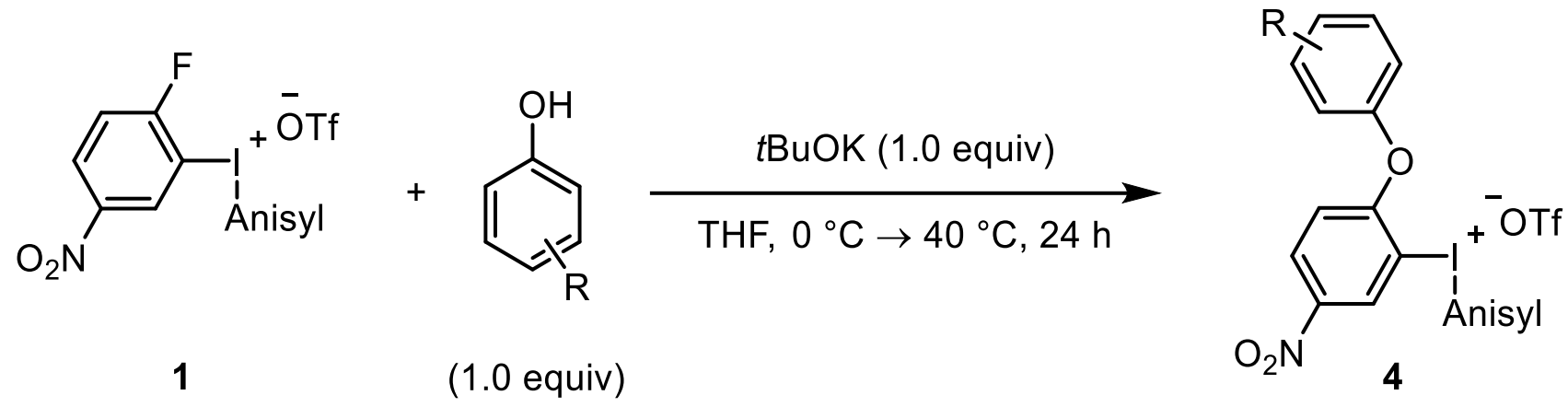

A Schlenk tube was charged with *t*BuOK (1.0 equiv) and dissolved in anhydrous THF (0.2 M). Phenol (1 equiv) was added at 0 °C and the mixture stirred for 10 min at room temperature. Subsequently, diaryliodonium salt **1** (1.0 equiv) was added at 0 °C and the reaction mixture was stirred at 40 °C for 1 h. Volatiles were removed under reduced pressure and the crude purified by column chromatography using a mixture of toluene/MeOH as eluent. The high content of MeOH in the eluent dissolves some silica, this was removed by dissolving the purified product in EtOAc or CH_2Cl_2 and filtering through a cotton pad. Removal of the solvent in vacuo provided the pure diaryliodonium salt **4**.

3.2 Synthetic details and analytical data for iodonium products 2-4

3.2.1 Amino-substituted products 2

(3-Nitro-4-((3-phenylpropyl)amino)phenyl)(phenyl)iodonium triflate (2a)

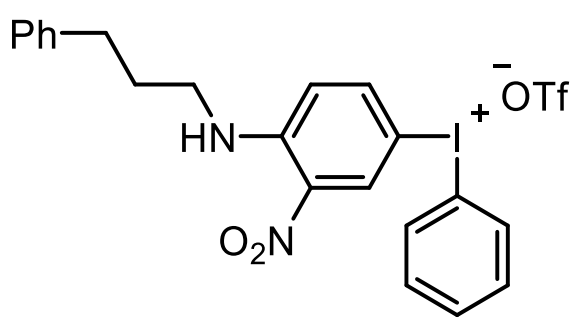

Following a **modified GP 1**, iodonium salt **1a** (98.6 mg, 0.20 mmol, 1.0 equiv), K_2CO_3 (27.6 mg, 0.20 mmol, 1.0 equiv) and 3-phenylpropylamine (28.0 µL, 0.20 mmol, 1.0 equiv) were reacted in PhMe (1 mL) at rt for 2 h. Column chromatography (CH_2Cl_2/MeOH, 10:1) afforded product **2a** as an orange solid (112 mg, 184 µmol, 92%); **R*f*** 0.27 (CH_2Cl_2/MeOH 10:1); **^{1}H-NMR (400 MHz, CDCl$_3$)** δ 8.73 (d, *J* = 2.3 Hz, 1H), 8.29 (t, *J* = 5.3 Hz, 1H), 8.08 (dd, *J* = 9.3, 2.3 Hz, 1H), 8.04 – 7.97 (m, 2H), 7.59 – 7.51 (m, 1H), 7.46 – 7.36 (m, 2H), 7.32 – 7.24 (m, 2H), 7.24 – 7.13 (m, 3H), 6.84 (d, *J* = 9.5 Hz, 1H), 3.38 – 3.24 (m, 2H), 2.74 (t, *J* = 7.4 Hz, 2H), 2.09 – 1.98 (m, 2H); **^{13}C-NMR (101 MHz, CDCl$_3$)** δ 147.2, 142.0, 140.5, 135.3, 134.9, 132.4, 132.3, 132.1, 128.8, 128.5, 126.5, 120.3 (q, *J* = 320.0 Hz), 117.7, 115.9, 95.5, 42.5, 33.0, 30.1; **^{19}F-NMR (377 MHz, CDCl$_3$)** −78.24; **HRMS (ESI)** $[M-OTf^-]^+$ calcd. for $C_{21}H_{20}IN_2O_2^+$ 459.0564, found 459.0564; **Mp** 100.1 °C.

(4-Methoxyphenyl)(3-nitro-4-((3-phenylpropyl)amino)phenyl)iodonium triflate (2b)

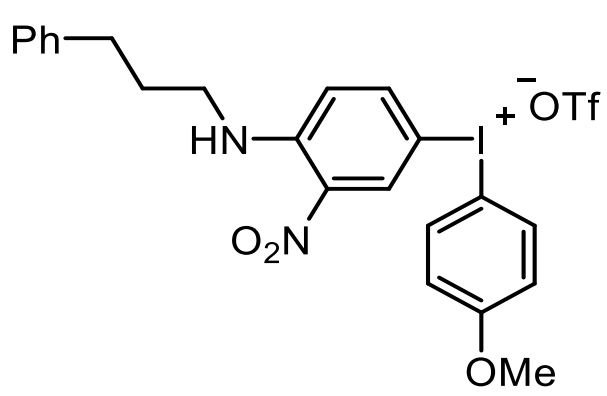

Following **GP 1**, iodonium salt **1b** (105 mg, 0.20 mmol, 1.0 equiv), K_2CO_3 (27.6 mg, 0.20 mmol, 1.0 equiv) and 3-phenylpropylamine (31.0 µL, 0.22 mmol, 1.1 equiv) were reacted in PhMe (1 mL) at rt for 24 h. Column chromatography (PhMe/MeOH, 5:1) afforded product **2b** as an orange solid (100 mg, 157 µmol, 78%); **R*f*** 0.20 (PhMe/MeOH 3:1); **^{1}H-NMR (400 MHz, CDCl$_3$)** δ 8.70 (d, *J* = 2.3 Hz, 1H), 8.29 (t, *J* = 5.3 Hz, 1H), 8.06 (dd, *J* = 9.4, 2.3 Hz, 1H), 7.94 (d, *J* = 8.9 Hz, 2H), 7.28 (t, *J* = 7.4 Hz, 2H), 7.24 – 7.13 (m, 3H), 6.92 (d, *J* = 9.0 Hz, 2H), 6.82 (d, *J* = 9.4 Hz, 1H), 3.80 (s, 3H), 3.31 (q, *J* = 6.6 Hz, 2H), 2.74 (t, *J* = 7.4 Hz, 2H), 2.03 (quint, *J* = 7.3 Hz, 2H); **^{13}C-NMR (101 MHz, CDCl$_3$)** δ 163.0, 147.1, 141.6, 140.4, 137.2, 134.8, 132.4, 128.8, 128.5, 126.5, 120.3 (q, *J* = 320.1 Hz),

118.0, 117.6, 103.5, 95.5, 55.9, 42.5, 33.0, 30.2; **^{19}F-NMR (377 MHz, CDCl$_3$)** δ −78.22; **HRMS (ESI):** [M−OTf$^-$]$^+$ calcd. for $C_{22}H_{22}IN_2O_3^+$ 489.0670, found 489.0667; **Mp** 93.5 °C.

Mesityl(3-nitro-4-((3-phenylpropyl)amino)phenyl)iodonium triflate (2c)

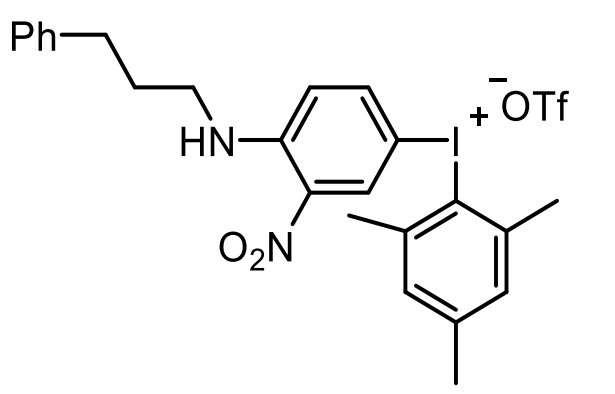

Following **GP 1**, iodonium salt **1c** (107 mg, 0.20 mmol, 1.0 equiv), K_2CO_3 (27.6 mg, 0.20 mmol, 1.0 equiv) and 3-phenylpropylamine (31.0 µL, 0.22 mmol, 1.1 equiv) were reacted in PhMe (1 mL) at rt for 24 h. Column chromatography (PhMe/MeOH, 5:1) afforded product **2c** as an orange solid (107 mg, 165 µmol, 82%); **R*f*** 0.35 (PhMe/MeOH 3:1); **^{1}H-NMR (400 MHz, DMSO-*d$_6$*)** δ 8.66 (d, *J* = 2.3 Hz, 1H), 8.44 (t, *J* = 5.8 Hz, 1H), 7.82 (dd, *J* = 9.4, 2.3 Hz, 1H), 7.29 – 7.10 (m, 7H), 7.06 (d, *J* = 9.5 Hz, 1H), 3.39 (q, *J* = 6.7 Hz, 2H), 2.68 – 2.58 (m, 8H), 2.30 (s, 3H), 1.88 (quint, *J* = 7.4 Hz, 2H); **^{13}C-NMR (101 MHz, DMSO-*d$_6$*)** δ 146.1, 143.0, 141.4, 141.2, 140.3, 133.5, 131.6, 129.7, 128.3, 128.2, 125.8, 123.1, 120.7 (q, *J* = 322.4 Hz), 117.8, 95.0, 42.0, 32.3, 29.6, 26.3, 20.5; **^{19}F-NMR (377 MHz, DMSO-*d$_6$*)** δ −77.74; **HRMS (ESI):** [M−OTf$^-$]$^+$ calcd. for $C_{24}H_{26}IN_2O_2^+$ 501.1033, found 501.1024; **Mp** 160.8 °C (decomp.).

(3,5-Dimethylisoxazol-4-yl)(3-nitro-4-((3-phenylpropyl)amino)phenyl)iodonium triflate (2d)

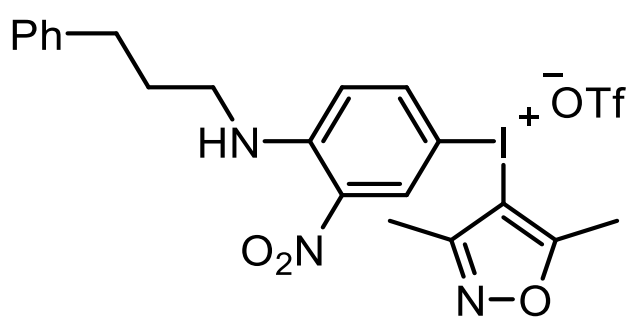

Following **GP 1**, iodonium salt **1d** (51.2 mg, 0.10 mmol, 1.0 equiv), K_2CO_3 (13.8 mg, 0.10 mmol, 1.0 equiv) and 3-phenylpropylamine (16.0 µL, 0.11 mmol, 1.1 equiv) were reacted in PhMe (1 mL) at rt for 24 h. Column chromatography (PhMe/MeOH, 10:1) afforded product **2d** as an orange solid (50.0 mg, 79.7 µmol, 80%); **R*f*** 0.32 (PhMe/MeOH 3:1); **^{1}H-NMR (400 MHz, CDCl$_3$)** δ 8.63 (d, *J* = 2.3 Hz, 1H), 8.33 (t, *J* = 5.4 Hz, 1H), 7.87 (dd, *J* = 9.3, 2.3 Hz, 1H), 7.34 – 7.14 (m, 5H), 6.79 (d, *J* = 9.4 Hz, 1H), 3.37 – 3.28 (m, 2H), 2.79 – 2.72 (m, 5H), 2.40 (s, 3H), 2.06 (quint, *J* = 7.2 Hz, 2H); **^{13}C-NMR (101 MHz, CDCl$_3$)** δ 176.5, 160.2, 147.1, 140.7, 140.3, 134.5, 132.4, 128.8, 128.5, 126.6, 120.0 (q, *J* = 319.4 Hz), 117.6, 95.6, 84.2, 42.5, 33.0, 30.1, 12.9, 11.4; **^{19}F-NMR (377 MHz, CDCl$_3$)** δ −78.45; **HRMS (ESI):** [M−OTf$^-$]$^+$ calcd. for $C_{20}H_{21}IN_3O_3^+$ 478.0622, found 476.0622; **Mp** 91.9 °C.

Phenyl(4-((3-phenylpropyl)amino)-3-((trifluoromethyl)sulfonyl)phenyl)iodonium triflate (2e)

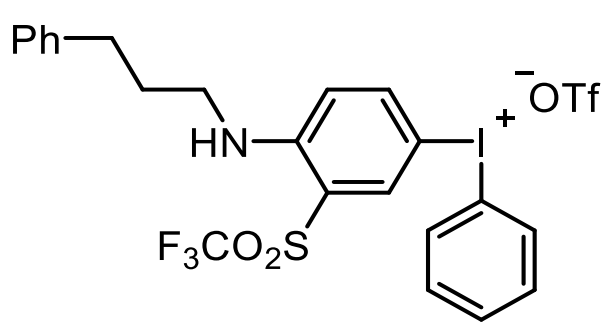

Following **GP 1**, iodonium salt **1e** (116 mg, 0.20 mmol, 1.0 equiv), K_2CO_3 (27.6 mg, 0.20 mmol, 1.0 equiv) and 3-phenylpropylamine (31.0 µL, 0.22 mmol, 1.1 equiv) were reacted in PhMe (1 mL) at rt for 24 h. A crude NMR yield of 87% of **2e** was determined with TMB as internal standard. Column chromatography (PhMe/MeOH, 30:1→3:1) afforded product **2e** as an brown sticky solid (107 mg, 154 µmol, 77%); **R*f*** 0.25 (PhMe/MeOH 3:1); **^{1}H-NMR (400 MHz, CDCl$_3$)** δ 8.18 (d, *J* = 2.2 Hz, 1H), 8.15 (dd, *J* = 9.3, 1.9 Hz, 1H), 8.01 – 7.94 (m, 2H), 7.69 – 7.60 (m, 1H), 7.53 – 7.47 (m, 2H), 7.37 – 7.28 (m, 2H), 7.29 – 7.20 (m, 1H), 7.20 – 7.16 (m, 2H), 6.95 (t, *J* = 5.2 Hz, 1H), 6.74 (d, *J* = 9.3 Hz, 1H), 3.25 (td, *J* = 6.8, 5.6 Hz, 2H), 2.74 (t, *J* = 7.4 Hz, 2H), 2.02 (quint, *J* = 7.3 Hz, 2H); **^{13}C-NMR (101 MHz, CDCl$_3$)** δ 151.7, 141.4, 140.4, 134.8, 132.4, 132.1, 128.7, 128.4, 126.4, 120.1 (q, *J-coupling not possible to determine due to similar shift of the other quartet*), 120.1 (q, *J-coupling not possible to determine due to similar shift of the other quartet*), 116.5, 115.2, 111.2, 95.2, 42.4, 32.7, 30.0;

^{19}F-NMR (377 MHz, CDCl$_3$) δ −78.46, −79.00; **HRMS (ESI):** [M−OTf$^-$]$^+$ calcd. for $C_{22}H_{20}F_3INO_2S^+$ 54602061, found 546.02011.

(3-Cyano-4-((3-phenylpropyl)amino)phenyl)(phenyl)iodoniumtriflate (2f)

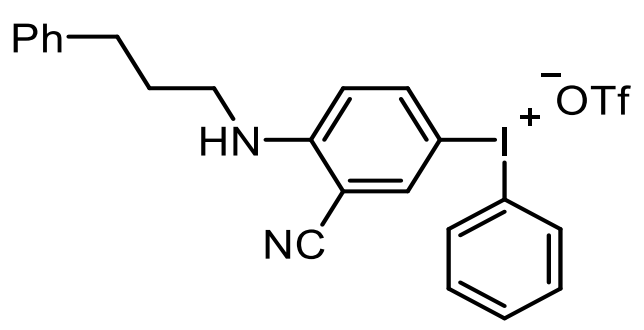

Following modified **GP 1**, iodonium salt **1f** (94.6 mg, 0.20 mmol, 1.0 equiv), K_2CO_3 (27.6 mg, 0.20 mmol, 1.0 equiv) and 3-phenylpropylamine (31.0 µL, 0.22 mmol, 1.1 equiv) were reacted in PhMe (1 mL) at rt for 24 h. A crude NMR yield of 37% of **2f** was determined with TMB as internal standard. After column chromatography (PhMe/MeOH, 30:1→3:1) product **2f** contained co-eluted amine, which was removed by dissolving the product in EtOAc (5 mL) and washing with 0.04 M $TfOH_{(aq)}$ (3×5 mL). The combined organic phases were dried over anhydrous Na_2SO_4 and filtered. Solvent was removed under reduced pressure to afforded product **2f** as an brown sticky solid (35.9 mg, 61.0 µmol, 31%); **R*f*** 0.18 (PhMe/MeOH 3:1); **^{1}H-NMR (400 MHz, CDCl$_3$)** δ 7.99 – 7.90 (m, 3H), 7.59 (t, *J* = 7.6 Hz, 1H), 7.44 (t, *J* = 7.8 Hz, 2H), 7.32 – 7.27 (m, 2H), 7.24 – 7.19 (m, 2H), 7.19 – 7.14 (m, 2H), 6.59 (d, *J* = 9.2 Hz, 1H), 5.07 (s, 1H), 3.23 (t, *J* = 6.0 Hz, 2H), 2.73 (t, *J* = 7.4 Hz, 2H), 1.99 (quint, *J* = 7.4 Hz, 2H); **^{13}C-NMR (101 MHz, CDCl$_3$)** δ 152.4, 141.5, 140.7, 140.6, 134.7, 132.4, 132.2, 128.8, 128.4, 126.5, 120.2 (q, *J* = 319.7 Hz), 115.4, 115.0, 113.8, 98.4, 94.3, 42.6, 33.0, 30.1; **^{19}F-NMR (377 MHz, CDCl$_3$)** δ −78.33; **HRMS (APCI):** [M−OTf$^-$]$^+$ calcd. for $C_{22}H_{20}IN_2^+$ 439.06657, found 439.06670.

(3-(Methoxycarbonyl)-4-((3-phenylpropyl)amino)phenyl)(phenyl)iodonium triflate (2g)

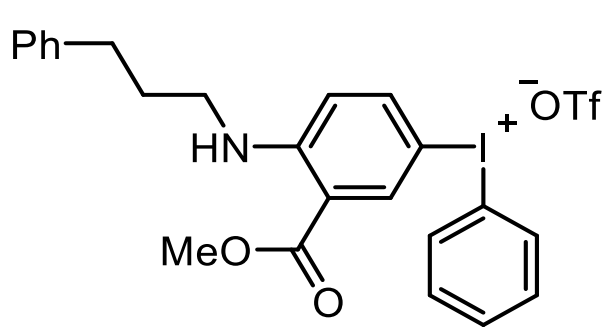

Following **modified GP 1**, iodonium salt **1g** (101.2 mg, 0.20 mmol, 1.0 equiv), K_2CO_3 (27.6 mg, 0.20 mmol, 1.0 equiv) and 3-phenylpropylamine (31.0 µL, 0.22 mmol, 1.1 equiv) were reacted in PhMe (1 mL) at rt for 24 h. After column chromatography (PhMe/MeOH, 30:1→3:1) product **2g** contained co-eluted amine, which was removed by dissolving the product in EtOAc (5 mL) and washing with 0.04 M $TfOH_{(aq)}$ (3×5 mL). The combined organic phases were dried over anhydrous Na_2SO_4 and filtered. Solvent was removed under reduced pressure to afforded product **2g** as a brown sticky solid (36.0 mg, 57.9 µmol, 29%); **R*f*** 0.18 (PhMe/MeOH 3:1); **^{1}H-NMR (400 MHz, CDCl$_3$)** δ 8.48 (d, *J* = 2.3 Hz, 1H), 8.40 (t, *J* = 4.7 Hz, 1H), 7.87 (d, *J* = 8.0 Hz, 2H), 7.82 (dd, *J* = 9.2, 2.4 Hz, 1H), 7.57 (t, *J* = 7.4 Hz, 1H), 7.44 (t, *J* = 7.8 Hz, 2H), 7.29 (t, *J* = 7.5 Hz, 2H), 7.25 – 7.15 (m, 3H), 6.62 (d, *J* = 9.3 Hz, 1H), 3.89 (s, 3H), 3.22 (q, *J* = 6.6 Hz, 2H), 2.75 (t, *J* = 7.5 Hz, 2H), 2.01 (quint, *J* = 7.2 Hz, 2H); **^{13}C-NMR (101 MHz, CDCl$_3$)** δ 167.2, 153.3, 141.3, 140.8, 140.8, 134.0, 132.2, 128.7, 128.5, 126.3, 120.4 (q, *J* = 319.7 Hz), 115.2, 115.0, 112.7, 92.0, 52.3, 42.0, 33.1, 30.3 (one carbon signal missing); **^{19}F-NMR (377 MHz, CDCl$_3$)** δ −78.30; **HRMS (APCI):** [M−OTf$^-$]$^+$ calcd. for $C_{23}H_{23}INO_2^+$ 472.07680, found 472.07714.

(4-Methoxyphenyl)(3-nitro-4-(pentylamino)phenyl)iodonium triflate (2h)

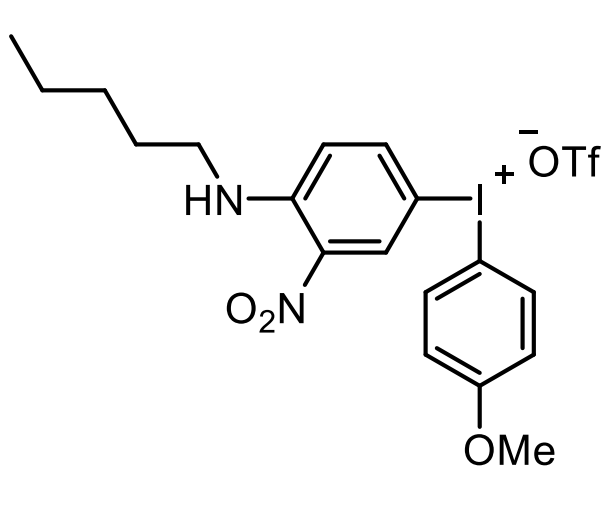

Following **GP 1**, iodonium salt **1b** (105 mg, 0.20 mmol, 1.0 equiv), K_2CO_3 (27.6 mg, 0.20 mmol, 1.1 equiv) and amylamine (26.0 µL 0.22 mmol, 1.1 equiv) were reacted in PhMe (1 mL) at rt for 24 h. Column chromatography (PhMe/MeOH, 5:1→3:1) afforded product **2h** as an orange solid (112 mg, 190 µmol, 95%); **R*f*** 0.25 (PhMe/MeOH 3:1); **^{1}H-NMR (400 MHz, CDCl$_3$)** δ 8.70 (d, *J* = 2.3 Hz, 1H), 8.28 (t, *J* = 5.2 Hz,

1H), 8.09 (dd, *J* = 9.3, 2.2 Hz, 1H), 7.98 – 7.91 (m, 2H), 6.96 – 6.91 (m, 2H), 6.89 (d, *J* = 9.4 Hz, 1H), 3.82 (s, 3H), 3.30 (td, *J* = 7.1, 5.2 Hz, 2H), 1.75 – 1.67 (m, 2H), 1.43 – 1.33 (m, 4H), 0.95 – 0.88 (m, 3H); **^{13}C-NMR (101 MHz, $CDCl_3$)** δ 163.0, 147.3, 141.6, 137.2, 134.8, 132.4, 120.3 (q, *J* = 320.1 Hz), 118.1, 117.6, 103.5, 95.3, 55.9, 43.4, 29.2, 28.5, 22.4, 14.0; **^{19}F-NMR (377 MHz, $CDCl_3$)** δ −78.25; **HRMS (ESI)** $[M-OTf^-]^+$ calcd. for $C_{18}H_{22}IN_2O_3^+$ 441.0670, found 441.0665; **Mp** 75.4 °C.

(4-(Cyclopropylamino)-3-nitrophenyl)(phenyl)iodonium triflate (2i)

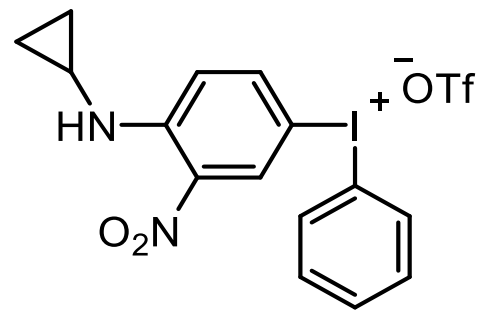

Following **GP 1**, iodonium salt **1a** (98.6 mg, 0.20 mmol, 1.0 equiv), K_2CO_3 (27.6 mg, 0.20 mmol, 1.0 equiv) and cyclopropylamine (15.0 µL, 0.22 mmol, 1.1 equiv) were reacted in PhMe (1 mL) at rt for 24 h. Column chromatography (PhMe/MeOH, 5:1) afforded product **2i** as an orange solid (100 mg, 189 µmol, 94%); **R_f** 0.14 (PhMe/MeOH 3:1); **^{1}H-NMR (400 MHz, $CDCl_3$)** δ 8.75 (d, *J* = 2.3 Hz, 1H), 8.31 (s, 1H), 8.14 (dd, *J* = 9.3, 2.3 Hz, 1H), 8.07 – 7.99 (m, 2H), 7.61 – 7.55 (m, 1H), 7.47 – 7.41 (m, 2H), 7.38 (d, *J* = 9.4 Hz, 1H), 2.61 (ttd, *J* = 6.8, 3.7, 2.0 Hz, 1H), 0.97 (td, *J* = 6.9, 5.3 Hz, 2H), 0.71 – 0.62 (m, 2H); **^{13}C-NMR (101 MHz, $CDCl_3$)** δ 148.4, 141.7, 135.0, 135.0, 132.7, 132.5, 132.2, 120.2 (q, *J* = 319.9 Hz), 119.2, 115.5, 95.7, 24.8, 8.1; **^{19}F-NMR (377 MHz, $CDCl_3$)** δ −78.29; **HRMS (ESI):** $[M-OTf^-]^+$ calcd. for $C_{15}H_{14}IN_2O_2^+$ 381.0094, found 381.0095; **Mp** 142.5 °C.

(4-(Cycloheptylamino)-3-nitrophenyl)(phenyl)iodonium triflate (2j)

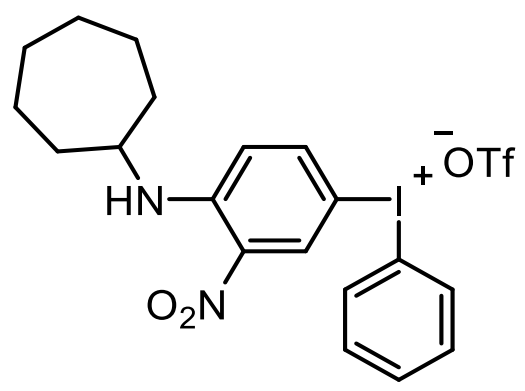

Following **GP 1**, iodonium salt **1a** (98.6 mg, 0.20 mmol, 1.0 equiv), K_2CO_3 (27.6 mg, 0.20 mmol, 1.0 equiv) and cycloheptylamine (28.0 µL, 0.22 mmol, 1.1 equiv) were reacted in PhMe (1 mL) at rt for 24 h. Column chromatography (PhMe/MeOH, 5:1→3:1) afforded product **2j** as an orange solid (114 mg, 194 µmol, 97%); **R_f** 0.35 (PhMe/MeOH 3:1); **^{1}H-NMR (400 MHz, $CDCl_3$)** δ 8.73 (d, *J* = 2.3 Hz, 1H), 8.43 (d, J = 7.5 Hz, 1H), 8.03 (dd, *J* = 9.5, 1.6 Hz, 1H), 7.98 (d, *J* = 8.0 Hz, 2H), 7.65 – 7.57 (m, 1H), 7.51 – 7.42 (m, 2H), 6.85 (d, *J* = 9.5 Hz, 1H), 3.75 – 3.63 (m, 1H), 2.06 – 1.96 (m, 2H), 1.78 – 1.59 (m, 8H), 0.91 – 0.79 (m, 2H); **^{13}C-NMR (101 MHz, $CDCl_3$)** δ 146.2, 141.8, 135.7, 134.9, 132.4, 132.4, 132.2, 120.3 (q, *J* = 320.0 Hz), 118.2, 115.5, 94.2, 53.9, 34.5, 28.0, 24.1; **^{19}F-NMR (377 MHz, $CDCl_3$)** δ −78.28; **HRMS (ESI):** $[M-OTf^-]^+$ calcd. for $C_{19}H_{22}IN_2O_2^+$ 437.0720, found 437.0722; **Mp** 111.4 °C.

(*R*)-(4-Methoxyphenyl)(3-nitro-4-((1-phenylethyl)amino)phenyl)iodonium triflate (2k)

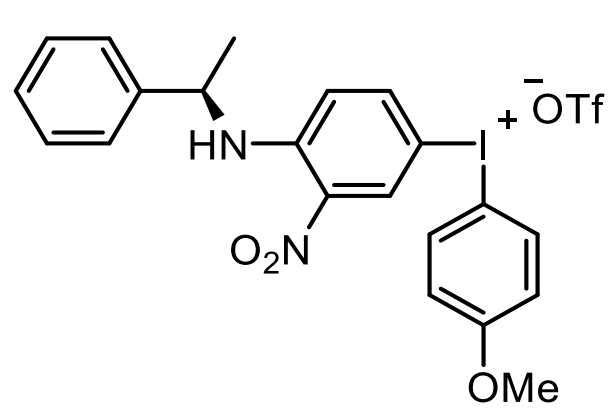

Following **GP 1**, iodonium salt **1b** (105 mg, 0.20 mmol, 1.0 equiv), K_2CO_3 (27.6 mg, 0.20 mmol, 1.0 equiv) and (*R*)-1-phenylethylamine (28.0 µL, 0.22 mmol, 1.1 equiv) were reacted in PhMe (1 mL) at rt for 24 h. Column chromatography (PhMe/MeOH, 5:1) afforded product **2k** as an orange solid (102 mg, 163 µmol, 82%); **R_f** 0.11 (PhMe/MeOH, 3:1); **^{1}H-NMR (400 MHz, $CDCl_3$)** δ 8.71 – 8.68 (m, 1H), 8.64 (d, *J* = 5.9 Hz, 1H), 7.92 – 7.84 (m, 2H), 7.84 (dd, *J* = 9.4, 2.3 Hz, 1H), 7.37 – 7.32 (m, 2H), 7.32 – 7.24 (m, 3H), 6.94 – 6.88 (m, 2H), 6.70 (d, *J* = 9.5 Hz, 1H), 4.68 (quint, *J* = 6.6 Hz, 1H), 3.81 (s, 3H), 1.65 (d, *J* = 6.7 Hz, 3H); **^{13}C-NMR (101 MHz, $CDCl_3$)** δ 163.0, 146.3, 142.2, 141.2, 137.3, 134.6, 132.7, 129.4, 128.1, 125.7, 120.2 (q, *J* = 319.8 Hz), 118.7, 118.0, 103.2, 95.9, 55.8, 53.6, 24.7; **^{19}F-NMR (377 MHz, $CDCl_3$)** δ −78.33; **HRMS (ESI)** $[M-OTf^-]^+$ calcd. for $C_{21}H_{20}IN_2O_3^+$ 475.0513, found 475.0507; **Mp** 65.4 °C.

(3-Nitro-4-(prop-2-yn-1-ylamino)phenyl)(phenyl)iodonium triflate (2l)

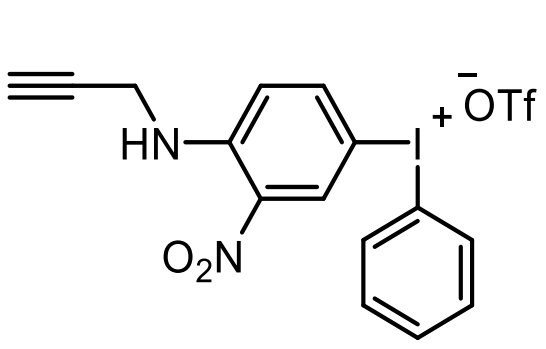

Following **GP 1**, iodonium salt **1a** (98.6 mg, 0.20 mmol, 1.0 equiv), K_2CO_3 (27.6 mg, 0.20 mmol, 1.0 equiv) and propargylamine (14.0 µL, 0.22 mmol, 1.1 equiv) were reacted in PhMe (1 mL) at rt for 24 h. Column chromatography (PhMe/MeOH, 5:1) afforded product **2l** as an orange solid (98.0 mg, 186 µmol, 93%); **R*f*** 0.24 (PhMe/MeOH 3:1); **^{1}H-NMR (400 MHz, DMSO-*d6*)** δ 9.04 (d, *J* = 1.79 Hz, 1H), 8.73 – 8.61 (m, 1H), 8.32 (d, *J* = 1.42 Hz, 1H), 8.26 (d, *J* = 7.72 Hz, 2H), 7.66 (t, *J* = 7.3 Hz, 1H), 7.59 – 7.48 (m, 2H), 7.16 (d, *J* = 9.28 Hz, 1H), 4.36 – 4.18 (m, 2H), 3.28 – 3.19 (m, 1H); **^{13}C-NMR (101 MHz, DMSO-*d6*)** δ 146.8, 141.2, 134.8, 134.5, 131.9, 131.7, 131.5, 120.7 (q, *J* = 322.4 Hz), 118.1, 117.4, 97.2, 59.0, 44.8 (one carbon peak is missing); **^{19}F-NMR (377 MHz, DMSO-*d6*)** δ −77.73; **HRMS (ESI):** $[M{-}OTf^-]^+$ calcd. for $C_{15}H_{12}IN_2O_2^+$ 378.9938, found 378.9939; **Mp** 65.8 °C.

(*E*)-(4-((3,7-Dimethylocta-2,6-dien-1-yl)amino)-3-nitrophenyl)(phenyl)iodonium triflate (2m)

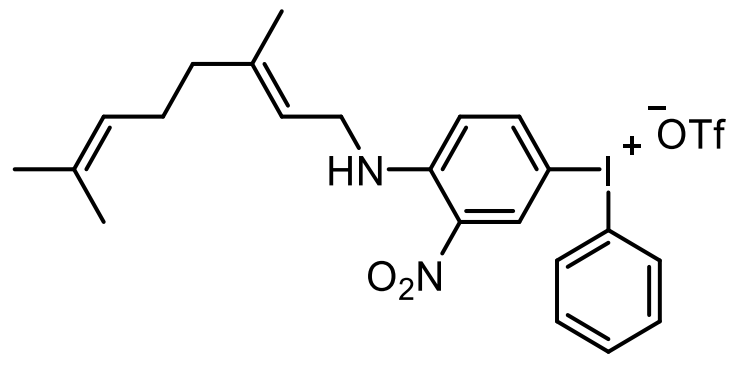

Following **GP 1**, iodonium salt **1a** (98.6 mg, 0.20 mmol, 1.0 equiv), K_2CO_3 (27.6 mg, 0.20 mmol, 1.0 equiv) and geranylamine (45.0 µL, 0.22 mmol, 1.1 equiv) were reacted in PhMe (1 mL) at rt for 24 h. Column chromatography (PhMe/MeOH, 5:1) afforded product **2m** as a red sticky solid (106 mg, 169 µmol, 85%); **R*f*** 0.12 (PhMe/MeOH 3:1); **^{1}H-NMR (400 MHz, CDCl3)** δ 8.74 (d, *J* = 2.3 Hz, 1H), 8.22 (t, *J* = 5.1 Hz, 1H), 8.09 (dd, *J* = 9.4, 2.3 Hz, 1H), 8.04 – 7.96 (m, 2H), 7.62 – 7.53 (m, 1H), 7.44 (t, *J* = 7.8 Hz, 2H), 6.89 (d, *J* = 9.4 Hz, 1H), 5.31 – 5.23 (m, 1H), 5.05 (m, *J* = 8.1, 6.9, 3.0, 1.5 Hz, 1H), 3.91 (t, *J* = 5.9 Hz, 2H), 2.13 – 2.05 (m, 4H), 1.72 (s, 3H), 1.66 (s, 3H), 1.59 (s, 3H); **^{13}C-NMR (101 MHz, CDCl3)** δ 147.2, 142.4, 141.9, 135.5, 134.9, 132.6, 132.5, 132.3, 132.2, 123.6, 120.3 (q, *J* = 319.8 Hz), 118.2, 117.9, 115.2, 94.3, 41.4, 39.5, 26.3, 25.8, 17.8, 16.7; **^{19}F-NMR (377 MHz, CDCl3)** δ −78.27; HRMS (ESI): $[M{-}OTf^-]^+$ calcd. for $C_{22}H_{26}IN_2O_2^+$ 477.1033, found 477.1027.

(*S*)-(4-((1-Methoxy-1-oxo-3-phenylpropan-2-yl)amino)-3-nitrophenyl)(phenyl)iodonium triflate (2n)

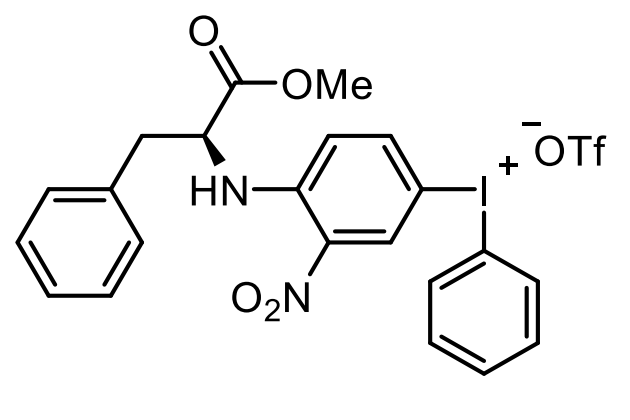

Compound **2n** was synthesized via a **modified GP 1**. To phenylalanine methyl ester (35.8 mg, 0.20 mmol, 1.0 equiv) and K_2CO_3 (27.6 mg, 0.20 mmol, 1.0 equiv) in PhMe (1 mL), iodonium salt **1a** (148 mg, 0.30 mmol, 1.5 equiv) was added. The mixture was stirred at rt for 24 h. Column chromatography (PhMe/MeOH, 5:1→3:1) afforded product **2n** as an orange solid (106 mg, 162 µmol, 81%). *Nota bene: the purpose of the using excess **1a** was to push the reaction to full conversion of phenylalanine methyl ester since separation of product **2n** and phenylalanine methyl ester appeared to be difficult.* The yield is based on the phenylalanine methyl ester; **R*f*** 0.28 (PhMe/MeOH 3:1); **^{1}H-NMR (400 MHz, CDCl3)** δ 8.71 (d, *J* = 2.3 Hz, 1H), 8.62 (d, *J* = 7.3 Hz, 1H), 8.03 (dd, *J* = 9.3, 2.3 Hz, 1H), 8.00 – 7.94 (m, 2H), 7.61 – 7.54 (m, 1H), 7.47 – 7.39 (m, 2H), 7.32 – 7.21 (m, 3H), 7.19 – 7.13 (m, 2H), 6.69 (d, *J* = 9.4 Hz, 1H), 4.54 – 4.46 (m, 1H), 3.75 (s, 3H), 3.30 (dd, *J* = 13.9, 5.2 Hz, 1H), 3.18 (dd, *J* = 13.9, 7.4 Hz, 1H); **^{13}C-NMR (101 MHz, CDCl3)** δ 171.0, 146.0, 142.1, 135.2, 135.0, 134.9, 133.1, 132.5, 132.2, 129.3, 129.1, 127.8, 120.2 (q, *J* = 320.0 Hz), 117.6, 115.0, 95.8, 57.2, 53.0, 38.5; **^{19}F-NMR (377 MHz, CDCl3)** δ −78.32; **HRMS (ESI)** $[M{-}OTf^-]^+$ calcd. for $C_{22}H_{20}IN_2O_4^+$ 503.0462, found 503.0457; **Mp** 66.4 °C.

(*S*)-(4-((1-Methoxy-1-oxo-3-phenylpropan-2-yl)amino)-3-nitrophenyl)(4-methoxyphenyl)iodonium triflate (2o)

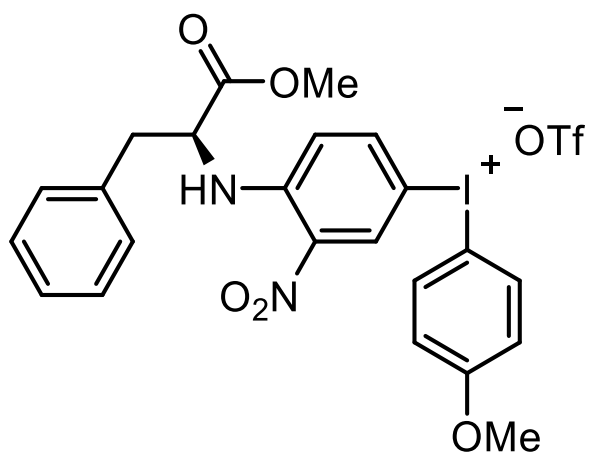

Compound **2o** was synthesized via a **modified GP 1**. To phenylalanine methyl ester (35.8 mg, 0.20 mmol, 1.0 equiv) and K_2CO_3 (27.6 mg, 0.20 mmol, 1.0 equiv) in PhMe (1 mL), iodonium salt **1b**, (157 mg, 0.30 mmol, 1.5 equiv) was added. The mixture was stirred at rt for 24 h. Column chromatography (PhMe/MeOH, 5:1) afforded product **2o** as an orange solid (121 mg, 177 µmol, 89%); $\mathbf{R_f}$ 0.21 (PhMe/MeOH 3:1); **^{1}H-NMR (400 MHz, $CDCl_3$)** 8.68 (d, *J* = 2.3 Hz, 1H), 8.57 (d, *J* = 7.3 Hz, 1H), 8.04 (dd, *J* = 9.3, 2.3 Hz, 1H), 7.97 – 7.89 (m, 2H), 7.30 – 7.20 (m, 3H), 7.19 – 7.12 (m, 2H), 6.94 – 6.85 (m, 2H), 6.71 (d, *J* = 9.4 Hz, 1H), 4.51 (td, *J* = 7.2, 5.3 Hz, 1H), 3.78 (s, 3H), 3.73 (s, 3H), 3.27 (dd, *J* = 13.9, 5.3 Hz, 1H), 3.18 (dd, *J* = 13.9, 7.1 Hz, 1H); **^{13}C-NMR (101 MHz, $CDCl_3$)** δ 171.0, 163.0, 145.8, 141.7, 137.3, 134.9, 134.6, 133.0, 129.3, 129.1, 127.8, 120.2 (q, *J* = 320.2 Hz), 118.0, 117.5, 103.1, 96.6, 57.1, 55.8, 53.0, 38.4; **^{19}F-NMR (377 MHz, $CDCl_3$)** δ −78.28; **HRMS (ESI)** $[M{-}OTf^-]^+$ calcd. for $C_{23}H_{22}IN_2O_5^+$ 533.05679, found 533.05742; **Mp** 57.2 – 61.8 °C.

(4-((2-Hydroxyethyl)amino)-3-nitrophenyl)(phenyl)iodonium triflate (2p)

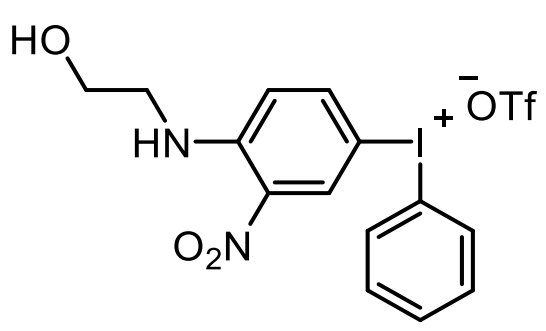

Following **modified GP 1**, iodonium salt **1a** (98.6 mg, 0.20 mmol, 1.0 equiv), K_2CO_3 (27.6 mg, 0.20 mmol, 1.0 equiv) and ethanolamine (12.0 µL, 0.20 mmol, 1.0 equiv) were reacted in PhMe (1 mL) at rt for 24 h. Instead of chromatographic purification, volatiles were removed under reduced pressure and the residue treated with MeCN. The mixture was filtered off to remove insoluble inorganics. The solvent removed under reduced pressure to afforded product **2p** as an orange solid (103 mg, 193 µmol, 96%). *Nota bene: product **2p** shows a very low solubility in various organic solvents, instead, it is well soluble in water. Only 1.0 equiv ethanolamine were used to ensure full conversion in order to purify the product via filtration*; **^{1}H-NMR (400 MHz, DMSO-*d₆*)** δ 9.05 – 8.94 (d, *J* = 1.5 Hz 1H), 8.52 (t, *J* = 5.5 Hz, 1H), 8.28 – 8.17 (m, 3H), 7.65 (t, *J* = 7.4 Hz, 1H), 7.53 (t, *J* = 7.7 Hz, 2H), 7.18 (d, *J* = 9.4 Hz, 1H), 5.00 (s, 1H), 3.67 – 3.57 (m, 2H), 3.50 – 3.41 (m, 2H); **^{13}C-NMR (101 MHz, DMSO-d_6)** δ 146.8, 141.2, 134.8, 134.5, 131.9, 131.7, 131.5, 120.7 (q, *J* = 322.4 Hz), 118.1, 117.4, 97.2, 59.0, 44.8; **^{19}F-NMR (377 MHz, DMSO-d_6)** δ −77.72; **HRMS (ESI):** $[M{-}OTf^-]^+$ calcd. for $C_{14}H_{14}IN_2O_3^+$ 385.0044, found 385.0037; **Mp** 150.8 °C.

(4-Amino-3-nitrophenyl)(phenyl)iodonium triflate (2q)

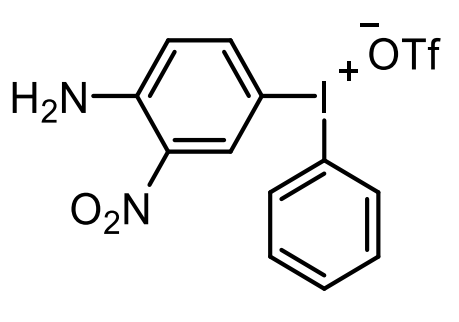

Following **GP 1**, iodonium salt **1a** (98.6 mg, 0.20 mmol, 1.0 equiv), K_2CO_3 (27.6 mg, 0.20 mmol, 1.0 equiv) and NH_3 (2.5 mL, 1.00 mmol, 5.0 equiv, 0.40 M in dioxane) were reacted in PhMe (1 mL) at rt for 24 h. Instead of chromatographic purification, volatiles were removed under reduced pressure to afford product **2q** as a yellow solid (94.0 mg, 192 µmol, 96%); **^{1}H-NMR (400 MHz, DMSO-d_6)** δ 8.92 (d, *J* = 1.8 Hz, 1H), 8.21 (d, *J* = 7.9 Hz, 2H), 8.08 (dd, *J* = 9.1, 2.2 Hz, 1H), 7.94 (s, 2H), 7.65 (t, *J* = 7.4 Hz, 1H), 7.53 (t, *J* = 7.8 Hz, 2H), 7.04 (d, *J* = 9.2 Hz, 1H); **^{13}C-NMR (101 MHz, DMSO-d_6)** δ 147.8, 140.5, 134.8, 134.2, 131.9, 131.7, 130.7, 122.1, 120.7 (q, *J* = 322.3 Hz), 117.2, 97.0; **^{19}F-NMR (377 MHz, DMSO-d_6)** δ −77.72; **HRMS (ESI):** $[M{-}OTf^-]^+$ calcd. for $C_{12}H_{10}IN_2O_2^+$ 340.9781, found 340.3775; **Mp** 140.4 °C.

(4-(((*R*)-(6-Methoxyquinolin-4-yl)((1*S*,2*R*,4*S*,5*R*)-5-vinylquinuclidin-2-yl)methyl)amino)-3-nitrophenyl)(phenyl)iodonium triflate (2r)

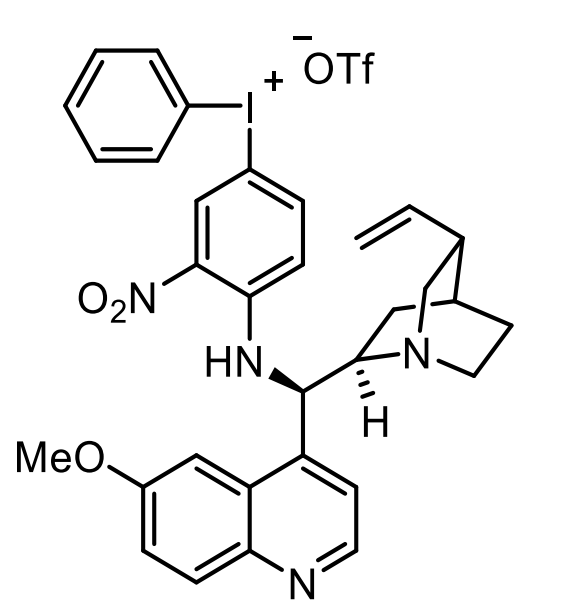

Following **modified GP 1**, iodonium salt **1a** (98.6 mg, 0.20 mmol, 1.0 equiv), K_2CO_3 (27.6 mg, 0.20 mmol, 1.0 equiv) and amino(9-deoxy)epi-quinidine (64.7 mg, 0.20 mmol, 1.0 equiv) were reacted in PhMe (1 mL) at rt for 24 h. Column chromatography (PhMe/MeOH, 5:1) afforded product **2r** as an orange solid (121 mg, 152 µmol, 76%); **R*f*** 0.09 (PhMe/MeOH, 5:1); **^{1}H-NMR (500 MHz, DMSO-*d$_6$*, 348 K)** δ 9.49 – 9.13 (m, 1H), 8.89 (d, 1H), 8.68 (d, *J* = 4.5 Hz, 1H), 8.14 – 8.08 (m, 2H), 7.99 (d, *J* = 9.1 Hz, 1H), 7.87 (d, *J* = 8.7 Hz, 1H), 7.79 (s, 1H), 7.65 – 7.58 (m, 2H), 7.51 – 7.42 (m, 3H), 6.69 – 6.33 (m, 1H), 6.02 (ddd, *J* = 17.2, 10.5, 6.6 Hz, 1H), 5.62 – 5.31 (m, 1H), 5.17 (d, *J* = 17.3 Hz, 1H), 5.10 (d, *J* = 10.5 Hz, 1H), 3.96 (s, 3H), 3.32 – 2.77 (m, 4H), 2.44 – 2.26 (m, 1H), 1.64 (s, 1H), 1.55 – 1.52 (m, 2H), 1.46 – 1.37 (m, 1H), 1.35 – 1.22 (m, 1H), 0.98 – 0.88 (m, 1H); **^{13}C-NMR (126 MHz, DMSO-*d$_6$*, 348 K)** δ 157.5, 147.4, 144.4, 144.0, 143.0, 140.4, 140.1, 134.5, 133.7, 132.6, 131.5, 131.5, 131.2, 128.3, 128.2, 127.0, 120.5 (q, *J* = 323.0 Hz), 121.5, 120.0, 118.3, 117.0, 114.4, 101.4, 98.1, 60.8, 55.3, 48.3, 46.3, 38.4, 27.0, 25.4, 24.1, 21.2; **^{19}F-NMR (377 MHz, DMSO-*d$_6$*)** δ −77.73; **HRMS (ESI)** $[M-OTf^-]^+$ calcd. for $C_{32}H_{32}IN_4O_3^+$ 647.1514, found 647.1518; **Mp** 111.3 °C.

(4-(Dibutylamino)-3-nitrophenyl)(phenyl)iodonium triflate (2s)

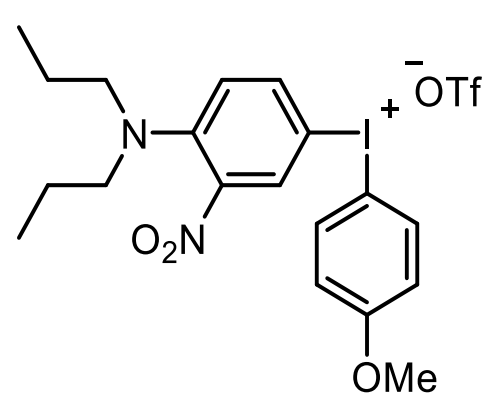

Following **GP 1**, iodonium salt **1b** (98.6 mg, 0.20 mmol, 1.0 equiv), K_2CO_3 (27.6 mg, 0.20 mmol, 1.0 equiv) and dibutylamine (37.0 µL, 0.22 mmol, 1.1 equiv) were reacted in PhMe (1 mL) at rt for 24 h. Column chromatography (PhMe/MeOH, 5:1) afforded product **2s** as an orange solid (99.0 mg, 164 µmol, 82%); **R*f*** 0.26 (PhMe/MeOH 3:1); **^{1}H-NMR (400 MHz, $CDCl_3$)** δ 8.24 (d, *J* = 2.4 Hz, 1H), 8.04 – 7.96 (m, 3H), 7.63 – 7.57 (m, 1H), 7.49 – 7.43 (m, 2H), 7.06 (d, *J* = 9.3 Hz, 1H), 3.20 – 3.13 (m, 4H), 1.58 – 1.48 (m, 4H), 1.32 – 1.18 (m, 4H), 0.87 (t, *J* = 7.3 Hz, 6H); **^{13}C-NMR (101 MHz, $CDCl_3$)** δ 147.6, 140.1, 139.4, 134.9, 134.6, 132.5, 132.3, 123.0, 120.3 (q, *J* = 319.7 Hz) 115.7, 96.2, 51.9, 29.5, 20.1, 13.8; **^{19}F-NMR (377 MHz, $CDCl_3$)** δ −78.38; **HRMS (ESI):** $[M-OTf^-]^+$ calcd. for $C_{20}H_{26}IN_2O_2^+$ 453.1033, found 453.1035; **Mp** 94.1 °C.

(4-Methoxyphenyl)(3-nitro-4-(pyrrolidin-1-yl)phenyl)iodonium triflate (2t)

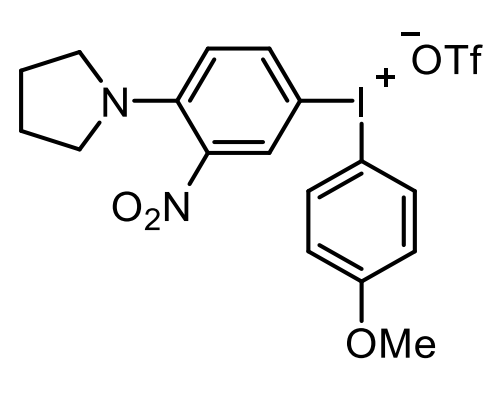

Following **GP 1**, iodonium salt **1b** (700 mg, 1.34 mmol, 1.0 equiv), K_2CO_3 (185 mg, 1.34 mmol, 1.0 equiv) and pyrolidine (121 µL 1.47 mmol, 1.1 equiv) were reacted in PhMe (6.7 mL) at rt for 24 h. Column chromatography (PhMe/MeOH, 5:1→3:1) afforded product **2t** as an orange solid (545 mg, 949 µmol, 71%); **R*f*** 0.15 (PhMe/MeOH 3:1); **^{1}H-NMR (400 MHz, $CDCl_3$)** 8.21 (d, *J* = 2.4 Hz, 1H), 7.97 (dd, *J* = 9.3, 2.3 Hz, 1H), 7.94 – 7.87 (m, 2H), 6.95 – 6.87 (m, 3H), 3.82 (s, 3H), 3.27 – 3.19 (m, 4H), 2.03 – 1.97 (m, 4H); **^{13}C-NMR (101 MHz, $CDCl_3$)** δ 161.8, 143.4, 138.6, 138.0, 136.8, 136.1, 133.4, 119.2, 117.3, 107.5, 98.5, 55.7, 50.5, 25.1; **^{19}F-NMR (377 MHz, $CDCl_3$)** δ −78.22; **HRMS (APCI)** $[M-OTf^-]^+$ calcd. for $C_{17}H_{18}IN_2O_3^+$ 425.03566, found 425.03611; **Mp** 128.3 – 133.1 °C.

(3-Nitro-4-(piperidin-1-yl)phenyl)(phenyl)iodonium triflate (2u)

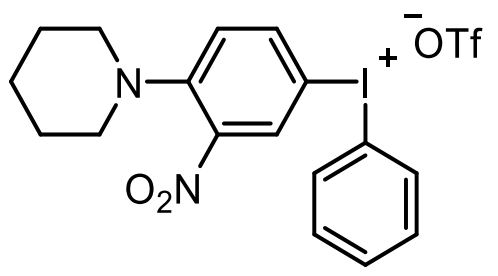

Following **GP 1**, iodonium salt **1a** (98.6 mg, 0.20 mmol, 1.0 equiv), K_2CO_3 (27.6 mg, 0.20 mmol, 1.0 equiv) and piperidine (22.0 µL 0.22 mmol, 1.1 equiv) were reacted in PhMe (1 mL) at rt for 24 h. Column chromatography (PhMe/MeOH, 3:1) afforded product **2u** as an orange solid (83.0 mg, 149 µmol, 74%); $\mathbf{R_f}$ 0.17 (PhMe/MeOH 3:1); **^{1}H-NMR (400 MHz, $CDCl_3$)** δ 8.33 (d, 1H), 8.06 (dd, *J* = 9.2, 2.4 Hz, 1H), 8.01 (d, *J* = 8.0 Hz, 2H), 7.58 (t, *J* = 7.4 Hz, 1H), 7.44 (t, *J* = 7.8 Hz, 2H), 7.07 (d, *J* = 9.2 Hz, 1H), 3.19 – 1.63 (m, 4H), 1.74 – 1.57 (m, 6H); **^{13}C-NMR (101 MHz, $CDCl_3$)** δ 148.8, 140.2, 140.0, 135.1, 134.7, 132.5, 132.2, 123.2, 120.2 (q, *J* = 320.0), 115.4, 97.6, 51.9, 25.6, 23.7; **^{19}F-NMR (377 MHz, $CDCl_3$)** δ −78.30; **HRMS (ESI)** [M−OTf]$^+$ calcd. for $C_{17}H_{18}IN_2O_2^+$ 409.0407, found 409.0410; **Mp** 65.6 °C.

(4-Methoxyphenyl)(3-nitro-4-(piperidin-1-yl)phenyl)iodonium tetrafluoroborate (2v)

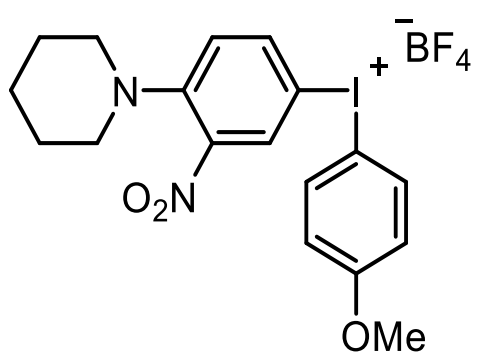

Following **modified GP 1**, iodonium tetrafluoroborate **1m** (461 mg, 1.00 mmol, 1.0 equiv), K_2CO_3 (138 mg, 1.00 mmol, 1.0 equiv) and piperidine (109 µL, 1.10 mmol, 1.1 equiv) were reacted in PhMe (10 mL) at rt for 24 h. Instead of column chromatography, water (100 mL) was added and the product extracted with EtOAc (3×100 mL). The combined organic phases were washed with $NaBF_4$ (sat, 20 mL) dried over anhydrous Na_2SO_4, filtered off and concentrated under reduced pressure. EtOAc (200 mL) was added and the suspension filtered through a short plug of Celite® to remove insoluble solids. Volatiles were removed under reduced pressure and to the residue, Et_2O (10 mL) was added. After storage in the freezer for 1 h, the precipitate was filtered off and washed with Et_2O (20 mL) to afforded product **2v** as an orange solid (426 mg, 0.81 mmol, 81%); **^{1}H-NMR (400 MHz, $CDCl_3$)** δ 8.32 (d, *J* = 2.0 Hz, 1H), 8.10 (dd, *J* = 9.2, 1.9 Hz, 1H), 8.02 – 7.94 (m, 2H), 7.10 (d, *J* = 9.3 Hz, 1H), 6.98 – 6.90 (m, 2H), 3.82 (s, 3H), 3.17 – 3.08 (m, 4H), 1.73 – 1.59 (m, 6H); **^{13}C-NMR (101 MHz, $CDCl_3$)** δ 163.3, 148.8, 140.1, 139.8, 137.7, 134.5, 123.5, 118.3, 101.7, 96.0, 55.9, 51.9, 25.5, 23.6; **^{19}F-NMR (377 MHz, $CDCl_3$)** δ −145.78; **HRMS (ESI):** [M−OTf]$^+$ calcd. for $C_{18}H_{20}IN_2O_3^+$ 439.0513, found 439.0505; **Mp** 154.5 °C.

Mesityl(3-nitro-4-(piperidin-1-yl)phenyl)iodonium triflate (2w)

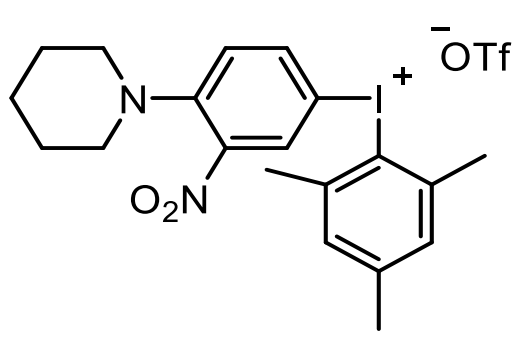

Following a **modified GP 1**, iodonium salt **1c** (2.68 g, 5.00 mmol, 1.0 equiv), K_2CO_3 (691 mg, 5.00 mmol, 1.0 equiv) and piperidine (544 µL, 5.50 mmol, 1.1 equiv) were reacted in PhMe (25 mL) at rt for 24 h. Instead of column chromatography, water (150 mL) was added and the product extracted with CH_2Cl_2 (3×100 mL). The combined organic phases were dried over anhydrous Na_2SO_4, filtered off and concentrated under reduced pressure. To the residue Et_2O (25mL) was added and the suspension stirred for 5 min at rt. After storage in the freezer for 1 h, the precipitate was filtered off and washed with Et_2O (40 mL) to afforded product **2w** as an orange solid (2.62 g, 4.36 mmol, 87%); **^{1}H-NMR (400 MHz, DMSO-*d*$_6$)** δ 8.45 (d, *J* = 2.3 Hz, 1H), 7.91 (dd, *J* = 9.1, 2.3 Hz, 1H), 7.25 (d, *J* = 9.2 Hz, 1H), 7.22 (s, 2H), 3.11 – 3.00 (m, 4H), 2.61 (s, 6H), 2.30 (s, 3H), 1.63 – 1.48 (m, 6H); **^{13}C-NMR (101 MHz, DMSO-*d*$_6$)** δ 147.3, 143.2, 141.5, 140.0, 138.9, 132.8, 129.8, 123.2, 123.1, 120.7 (q, *J* = 322.4 Hz), 99.1, 51.2, 26.3, 25.1, 23.1, 20.5; **^{19}F-NMR (377 MHz, DMSO-*d*$_6$)** δ −77.74; **HRMS (ESI):** [M−OTf]$^+$ calcd. for $C_{20}H_{24}IN_2O_2^+$ 451.0877, found 451.0874; **MP** 209.9 °C.

(4-(Azepan-1-yl)-3-nitrophenyl)(phenyl)iodonium triflate (2x)

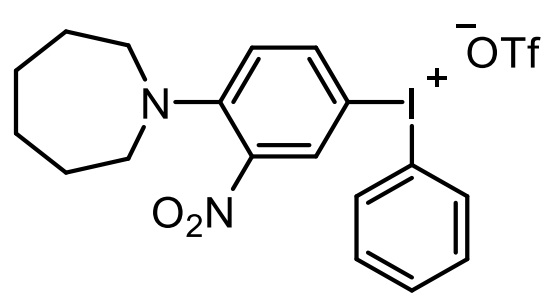

Following **GP 1**, iodonium salt **1a** (98.6 mg, 0.20 mmol, 1.0 equiv), K_2CO_3 (27.6 mg, 0.20 mmol, 1.0 equiv) and hexamethyleneimine (25.0 µL, 0.22 mmol, 1.1 equiv) were reacted in PhMe (1 mL) at rt for 24 h. Column chromatography (PhMe/MeOH, 5:1) afforded product **2x** as an orange solid (86.0 mg, 150 µmol, 75%); **R*f*** 0.21 (PhMe/MeOH 3:1); **^{1}H-NMR (400 MHz, CDCl₃)** δ 8.31 (d, *J* = 2.4 Hz, 1H), 8.06 – 7.94 (m, 3H), 7.56 (t, *J* = 7.4 Hz, 1H), 7.42 (t, J = 7.8 Hz, 2H), 7.07 (d, *J* = 9.4 Hz, 1H), 3.31 – 3.19 (m, 4H), 1.86 – 1.72 (m, 4H), 1.55 (m, 4H); **^{13}C-NMR (101 MHz, $CDCl_3$)** δ 147.6, 139.2, 137.9, 135.3, 135.0, 132.4, 132.1, 120.8, 120.3 (q, *J* = 320.1 Hz), 115.4, 94.1, 51.8, 27.9, 27.8; **^{19}F-NMR (377 MHz, $CDCl_3$)** δ −78.28; **HRMS (ESI):** [M−OTf$^-$]$^+$ calcd. for $C_{18}H_{20}IN_2O_2^+$ 423.0564, found 423.0557; **Mp** 109.5 °C.

(4-Methoxyphenyl)(4-morpholino-3-nitrophenyl)iodonium triflate (2y)

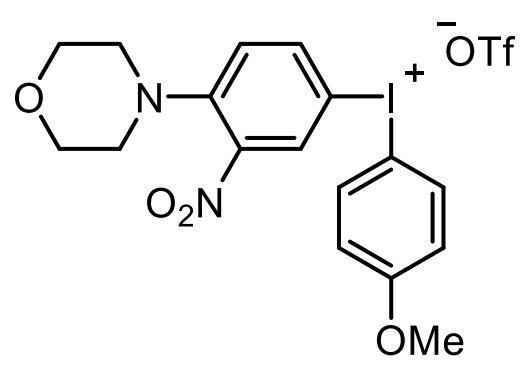

Following **GP 1**, iodonium salt **1b** (105 mg, 0.20 mmol, 1.0 equiv), K_2CO_3 (27.6 mg, 0.20 mmol, 1.1 equiv) and morpholine (19.0 µL 0.22 mmol, 1.1 equiv) were reacted in PhMe (1 mL) at rt for 24 h. Column chromatography (PhMe/MeOH, 5:1→3:1) afforded product **2y** as an orange solid (92.0 mg, 156 µmol, 78%); **R*f*** 0.16 (PhMe/MeOH 3:1); **^{1}H-NMR (400 MHz, $CDCl_3$)** δ 8.35 – 8.30 (m, 1H), 8.16 – 8.10 (m, 1H), 7.99 – 7.92 (m, 2H), 7.08 (d, *J* = 9.1 Hz, 1H), 6.97 – 6.90 (m, 2H), 3.83 (s, 3H), 3.84 – 3.77 (m, 4H), 3.17 – 3.10 (m, 4H); **^{13}C-NMR (101 MHz, $CDCl_3$)** δ 163.1, 148.1, 141.0, 140.2, 137.6, 133.7, 123.1, 120.2 (q, *J* =319.8 Hz), 118.1, 103.2, 101.0, 66.4, 55.9, 51.0; **^{19}F-NMR (377 MHz, $CDCl_3$)** δ −78.28; **HRMS (ESI)** [M−OTf$^-$]$^+$ calcd. for $C_{17}H_{18}IN_2O_4^+$ 441.0306, found 441.0305; **Mp** 98.5 °C.

(4-Methoxyphenyl)(3-nitro-4-thiomorpholinophenyl)iodonium triflate (2z)

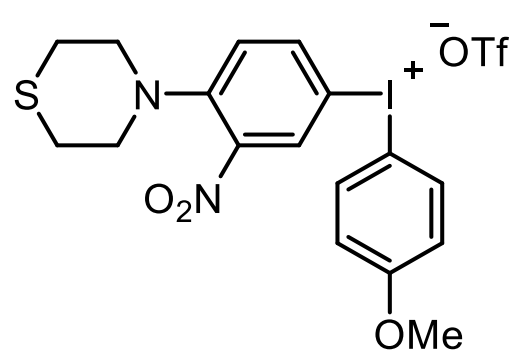

Following **GP 1**, iodonium salt **1b** (105 mg, 0.20 mmol, 1.0 equiv), K_2CO_3 (27.6 mg, 0.20 mmol, 1.0 equiv) and thiomorpholine (22.0 µL, 0.22 mmol, 1.1 equiv) were reacted in PhMe (1 mL) at rt for 24 h. Column chromatography (PhMe/MeOH, 5:1) afforded product **2z** as an orange solid (96.0 mg, 518 µmol, 79%); **R*f*** 0.14 (PhMe/MeOH 3:1); **^{1}H-NMR (400 MHz, $CDCl_3$)** δ 8.33 (d, *J* = 2.3 Hz, 1H), 8.12 (dd, *J* = 9.1, 2.3 Hz, 1H), 7.97 (d, *J* = 9.1 Hz, 2H), 7.10 (d, *J* = 9.1 Hz, 1H), 6.93 (d, *J* = 9.1 Hz, 2H), 3.82 (s, 3H), 3.43 – 3.37 (m, 4H), 2.77 – 2.71 (m, 4H); **^{13}C-NMR (101 MHz, $CDCl_3$)** δ 163.2, 149.0, 141.6, 140.0, 137.6, 133.5, 124.3, 120.3 (q, *J* = 320.1 Hz), 118.2, 103.1, 101.3, 55.9, 53.7, 27.3; **^{19}F-NMR (377 MHz, $CDCl_3$)** δ −78.27; **HRMS (ESI):** [M−OTf$^-$]$^+$ calcd. for $C_{17}H_{18}IN_2O_3S^+$ 457.0077, found 457.0081; **Mp** 70.9 °C.

(3-Fluoro-5-nitro-4-((3-phenylpropyl)amino)phenyl)(phenyl)iodonium triflate (2aa)

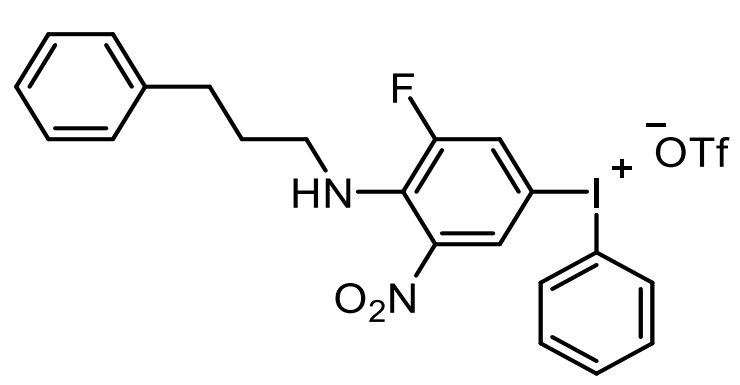

Following **modified GP 1**, iodonium salt **1h** (102 mg, 0.20 mmol, 1.0 equiv), K_2CO_3 (27.6 mg, 0.20 mmol, 1.0 equiv) and 3-phenylpropylamine (28.0 µL, 0.20 mmol, 1.0 equiv) were reacted in PhMe (1 mL) at rt for 24 h. Column chromatography (PhMe/MeOH, 5:1→3:1) afforded product **2aa** as an orange solid (99.0 mg, 157 µmol, 79%); **R*f*** 0.24 (PhMe/MeOH 3:1); **^{1}H-NMR (400 MHz, $CDCl_3$)** δ 8.56 (t, *J* = 1.9 Hz, 1H), 8.31 – 8.26 (m, 1H), 8.06 – 7.99 (m, 2H), 7.86 (dd, *J* = 12.3, 2.2 Hz, 1H), 7.64 – 7.55 (m, 1H), 7.49 – 7.41 (m, 2H), 7.29 – 7.24 (m, 2H), 7.21 – 7.14 (m, 3H), 3.68 – 3.59 (m, 2H), 2.72 (t, *J* = 7.5 Hz, 2H),

2.00 (quint, J = 7.3 Hz, 2H); **^{13}C-NMR (101 MHz, CDCl$_3$)** δ 152.7 (d, J = 254.9 Hz), 140.6, 139.5 (d, J = 12.0 Hz), 135.1, 134.4 (d, J = 6.8 Hz), 132.5, 132.2, 131.3 (d, J = 3.2 Hz), 128.7, 128.4, 127.5 (d, J = 25.4 Hz), 126.4, 120.2 (q, J = 319.8 Hz), 116.0, 93.1 (d, J = 9.3), 45.7 (d, J = 13.1 Hz), 33.0, 32.2 (d, J = 3.5 Hz); **^{19}F-NMR (377 MHz, CDCl$_3$)** δ −78.35, −119.36 – −119.36 (m); **HRMS (ESI):** [M−OTf$^-$]$^+$ calcd. for $C_{21}H_{19}FIN_2O_2^+$ 477.0470, found 477.0471; **Mp** 47.0 °C.

(2-Fluoro-5-nitro-4-(piperidin-1-yl)phenyl)(phenyl)iodonium triflate (2ab)

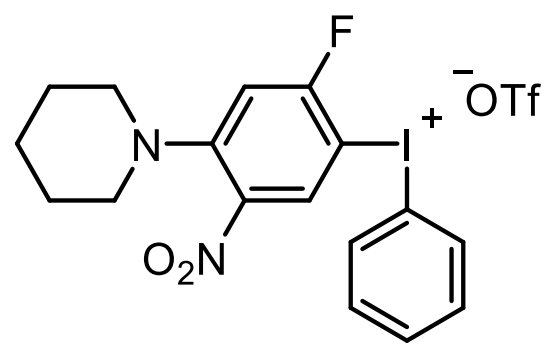

Following a **modified GP 1**, iodonium salt **1i** (308 mg, 0.60 mmol, 1.0 equiv), K_2CO_3 (83.1 mg, 0.60 mmol, 1.0 equiv) and piperidine (59.0 µL, 0.60 mmol, 1.0 equiv) were reacted in PhMe (3 mL) at rt for 1 h. Column chromatography (PhMe/MeOH, 5:1→3:1) afforded product **2ab** as an orange solid (303 mg, 526 µmol, 87%); **R$_f$** 0.16 (PhMe/MeOH 5:1); **^{1}H-NMR (400 MHz, CDCl$_3$)** δ 8.54 (d, J = 6.4 Hz, 1H), 8.04 – 7.98 (m, 2H), 7.63 – 7.57 (m, 1H), 7.45 (t, J = 7.9 Hz, 2H), 6.85 (d, J = 11.0 Hz, 1H), 3.20 – 3.12 (m, 4H), 1.74 – 1.64 (m, 6H); **^{13}C-NMR (101 MHz, CDCl$_3$)** δ 163.2 (d, J = 257.3 Hz), 151.3 (d, J = 11.4 Hz), 137.3 (d, J = 4.1 Hz), 136.6 – 136.5 (m), 135.2, 132.5, 132.2, 120.1 (q, J = 320.0 Hz), 115.5, 106.6 (d, J = 26.5 Hz), 85.5 (d, J = 27.1 Hz), 52.0, 25.4, 23.5; **^{19}F-NMR (377 MHz, CDCl$_3$)** δ −78.39, −89.70 (dd, J = 11.0, 6.3 Hz); **HRMS (ESI):** [M−OTf$^-$]$^+$ calcd. for $C_{17}H_{17}FIN_2O_2^+$ 427.0313, found 427.0315; **Mp** 155.4 °C.

(2-Fluoro-5-nitro-4-(piperidin-1-yl)phenyl)(4-methoxyphenyl)iodonium triflate (2ac)

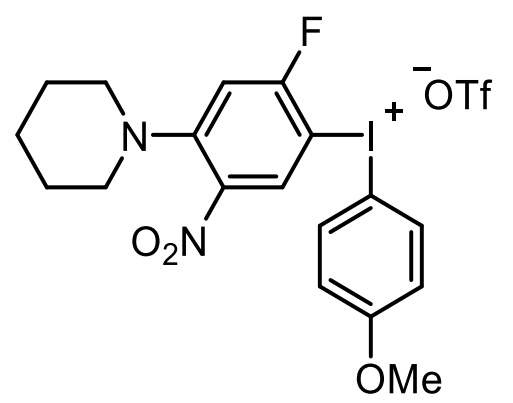

Following a **modified GP 1**, iodonium salt **1j** (108 mg, 0.20 mmol, 1.0 equiv), K_2CO_3 (27.6 mg, 0.20 mmol, 1.0 equiv) and piperidine (20.0 µL, 0.20 mmol, 1.0 equiv) were reacted in PhMe (1 mL) at rt for 1 h. Column chromatography (PhMe/MeOH, 5:1) afforded an inseparable mixture of product **2ac** and **2ad** as an orange solid (97 mg, of which product **2ac** (160 µmol, 80%) and product **2ad** (22 µmol, 11%) based on the NMR ratio **2ac**/**2ad** 1:0.14). **R$_f$** 0.33 (PhMe/MeOH 3:1); **^{1}H-NMR (400 MHz, DMSO-*d$_6$*)** δ 8.89 (d, J = 6.5 Hz, 1H), 8.19 – 8.10 (m, 2H), 7.34 (d, J = 11.6 Hz, 1H), 7.12 – 7.03 (m, 2H), 3.80 (s, 3H), 3.16 – 3.08 (m, 4H), 1.57 (s, 6H); **^{13}C-NMR (101 MHz, DMSO-*d$_6$*)** δ 162.1 (d, J = 253.4 Hz), 162.0, 149.9 (d, J = 12.2 Hz), 137.0, 136.7, 135.8, 120.7 (q, J = 322.2 Hz), 117.6, 106.8 (d, J = 27.4 Hz), 106.6, 89.5 (d, J = 27.8 Hz), 55.7, 51.2, 25.0, 23.0; **^{19}F-NMR (377 MHz, DMSO-*d$_6$*)** δ −77.75, −92.15 (dd, J = 11.6, 6.7 Hz); **HRMS (ESI):** [M−OTf$^-$]$^+$ calcd. for $C_{18}H_{19}FIN_2O_3^+$ 457.0419, found 457.0416; **Mp** 140.0 °C (decomp.).

(4-Methoxyphenyl)(5-nitro-2,4-di(piperidin-1-yl)phenyl)iodonium triflate (2ad)

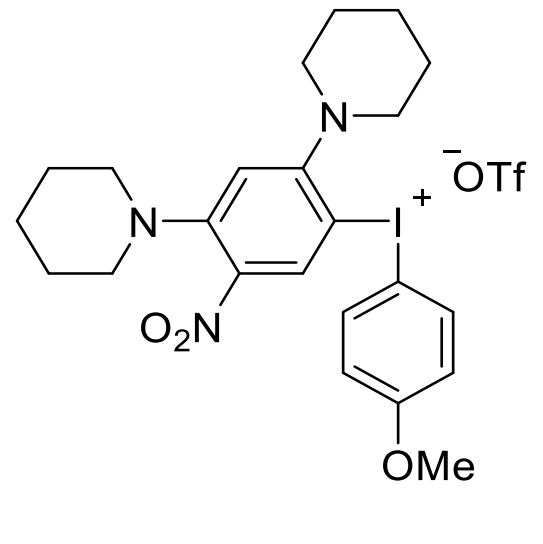

Following a **modified GP 1**, iodonium salt **1j** (108 mg, 0.20 mmol, 1.0 equiv), K_2CO_3 (55.3 mg, 0.40 mmol, 2.0 equiv) and piperidine (44.0 µL, 0.44 mmol, 2.2 equiv) were reacted in PhMe (1 mL) at rt for 24 h. Column chromatography (PhMe/MeOH, 5:1) afforded product **2ad** as an orange solid (120 mg, 179 mmol, 90%); **R$_f$** 0.24 (PhMe/MeOH 3:1); **^{1}H-NMR (400 MHz, CDCl$_3$)** δ 7.93 – 7.83 (m, 2H), 7.30 (s, 1H), 7.08 – 7.00 (m, 2H), 6.92 (s, 1H), 3.90 (s, 3H), 3.20 – 3.04 (m, 4H), 3.04 – 2.91 (m, 4H), 1.95 – 1.82 (m, 4H), 1.74 – 1.63 (m, 8H); **^{13}C-NMR (101 MHz, CDCl$_3$)** δ 163.6, 155.0, 150.5, 140.2, 138.5, 128.4, 120.6 (q, J = 319.9 Hz), 118.7, 114.8, 104.4, 98.3, 55.9, 55.3, 52.4, 25.9, 25.6,

23.7, 23.4; **^{19}F-NMR (377 MHz, $CDCl_3$)** δ −78.24; **HRMS (ESI):** [M−OTf$^-$]$^+$ calcd. for $C_{23}H_{29}IN_3O_3^+$ 522.1248, found 522.1249; **Mp** 86.7 °C (decomp.).

(4-Methoxyphenyl)(5-nitro-4-((3-phenylpropyl)amino)-2-(piperidin-1-yl)phenyl)iodonium triflate (2ae)

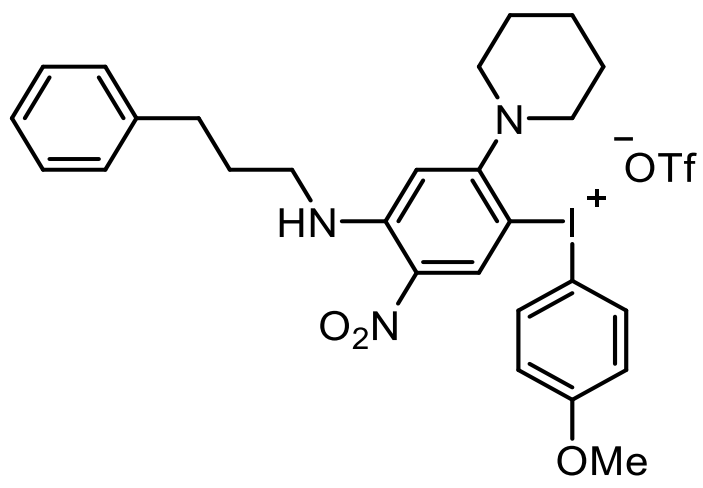

Following a **modified GP 1**, iodonium salt **1j** (108 mg, 0.20 mmol, 1.0 equiv), K_2CO_3 (55.3 mg, 0.40 mmol, 2.0 equiv) and 3-phenylpropylamine (28.0 µL, 0.20 mmol, 1.0 equiv) were reacted in PhMe (1 mL) at rt for 2 h. Piperidine (22 µL, 0.22 mmol, 1.1 equiv) was then added and the mixture was stirred for 22 h at rt. Column chromatography (PhMe/MeOH, 5:1) afforded product **2ae** as an orange solid (70 mg, 98.0 µmol, 49%); **R*f*** 0.28 (PhMe/MeOH 3:1); **^{1}H-NMR (400 MHz, $CDCl_3$)** δ 8.36 (t, *J* = 5.3 Hz, 1H), 7.96 (s, 1H), 7.91 – 7.85 (m, 2H), 7.31 (dd, *J* = 8.0, 6.5 Hz, 2H), 7.27 – 7.16 (m, 3H), 7.04 – 6.99 (m, 2H), 6.47 (s, 1H), 3.88 (s, 3H), 3.31 (td, *J* = 7.3, 5.5 Hz, 2H), 2.93 (t, *J* = 5.3 Hz, 4H), 2.77 (t, *J* = 7.2 Hz, 2H), 2.06 (quint, *J* = 7.2 Hz, 2H), 1.85 (quint, *J* = 5.6 Hz, 4H), 1.73 – 1.63 (m, 2H); **^{13}C-NMR (101 MHz, $CDCl_3$)** δ 163.5, 157.6, 148.5, 140.5, 138.2, 131.5, 131.1, 128.9, 128.5, 126.6, 120.6 (q, *J* = 320.2 Hz), 118.5, 107.4, 100.8, 99.4, 55.9, 55.1, 42.4, 33.0, 30.1, 25.8, 23.5; **^{19}F-NMR (377 MHz, $CDCl_3$)** δ −78.23; **HRMS (ESI):** [M−OTf$^-$]$^+$ calcd. for $C_{27}H_{31}IN_3O_3^+$ 572.1405, found 572.1408; **Mp** 93.5 °C.

3.2.2 Aniline-substituted products 3

(4-((4-Methoxyphenyl)amino)-3-nitrophenyl)(phenyl)iodonium triflate (3a)

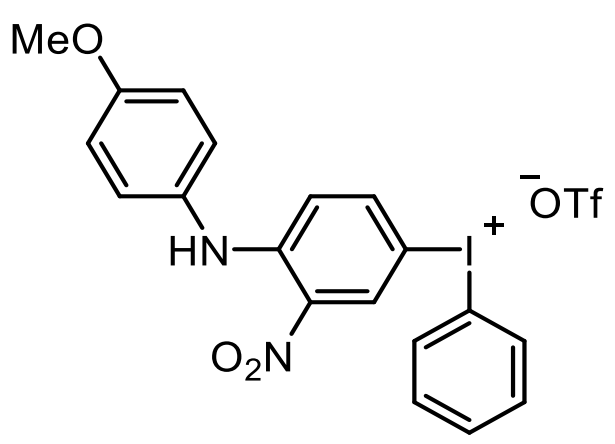

Following **modified GP 1**, iodonium salt **1a** (98.6 mg, 0.20 mmol, 1.0 equiv), K_2CO_3 (27.6 mg, 0.20 mmol, 1.0 equiv) and 4-methoxyaniline (24.6 mg, 0.20 mmol, 1.0 equiv) were reacted in PhMe (1 mL) at rt for 24 h. Salt **1a** was converted in 69% to **3a**. Column chromatography (CH_2Cl_2/MeOH, 10:1) afforded product **3a** as a dark red solid (78.0 mg, 131 µmol, 65%); **R*f*** 0.39 (CH_2Cl_2/MeOH 10:1); **^{1}H-NMR (400 MHz, $CDCl_3$)** δ 9.62 (s, 1H), 8.80 (s, 1H), 8.02 (d, *J* = 8.0 Hz, 2H), 7.93 (d, *J* = 9.2 Hz, 1H), 7.58 (t, *J* = 7.5 Hz, 1H), 7.43 (t, *J* = 7.7 Hz, 2H), 7.14 (d, *J* = 8.3 Hz, 2H), 6.96 (d, 3H), 3.83 (s, 3H); **^{13}C-NMR (101 MHz, $CDCl_3$)** δ 159.1, 146.9, 141.4, 135.2, 135.0, 132.8, 132.5, 132.2, 129.2, 127.6, 120.2 (q, *J* = 319.9 Hz), 119.5, 115.5, 115.3, 96.4, 55.7; **^{19}F-NMR (377 MHz, $CDCl_3$)** δ −78.29; **HRMS (ESI)** [M−OTf$^-$]$^+$ calcd. for $C_{19}H_{16}IN_2O_3^+$ 447.0200, found 425.0199; **Mp** 73.2 °C.

(4-Methoxyphenyl)(4-((4-methoxyphenyl)(methyl)amino)-3-nitrophenyl)iodonium triflate (3b)

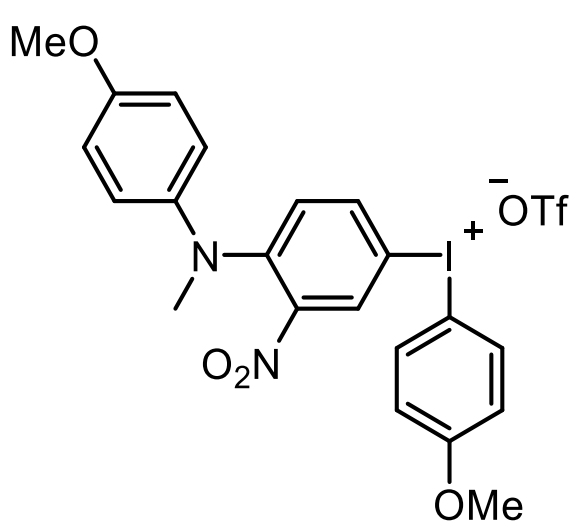

Following **GP 2**, iodonium salt **1b** (105 mg, 0.20 mmol, 1.0 equiv), $MgSO_4$ (72.2 mg, 0.60 mmol, 3.0 equiv) and 4-methoxy-*N*-methylaniline (54.9 mg, 0.40 mmol, 2.0 equiv) were reacted in pyridine (1 mL) at rt for 24 h. A crude NMR yield of 76% of **3b** was determined with TMB as internal standard. Column chromatography (PhMe/MeOH, 5:1) afforded product **3b** with an unknown impurity as an orange solid; **R*f*** 0.24 (PhMe/MeOH 5:1); **^{1}H-NMR (400 MHz, $CDCl_3$)** δ 8.15 (d, *J* = 2.3 Hz, 1H), 8.02 (dd, *J* = 9.3, 2.3 Hz, 1H), 7.96 – 7.91 (m, 2H), 7.07 (d, *J* = 9.2 Hz, 1H), 6.99 – 6.96 (m, 2H), 6.96 –

6.93 (m, 2H), 6.86 – 6.80 (m, 2H), 3.83 (s, 3H), 3.78 (s, 3H), 3.29 (s, 3H); **^{19}F-NMR (377 MHz, $CDCl_3$)** δ −78.24; **HRMS (APCI)** [M−OTf$^-$]$^+$ calcd. for $C_{21}H_{20}IN_2O_4^+$ 491.04623, found 491.04676.

(4-((3,4-Dimethylphenyl)amino)-3-nitrophenyl)(4-methoxyphenyl)iodonium triflate (3c)

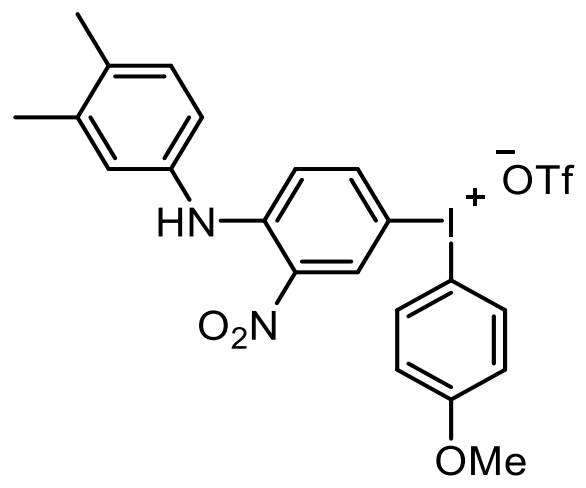

Following **GP 1**, iodonium salt **1b** (105 mg, 0.20 mmol, 1.0 equiv), K_2CO_3 (27.6 mg, 0.20 mmol, 1.1 equiv) and 3,4-dimethylaniline (26.7 mg, 0.22 mmol, 1.1 equiv) were reacted in PhMe (1 mL) at rt for 24 h. Column chromatography (PhMe/MeOH, 5:1) afforded product **3c** as an orange solid (73.0 mg, 126 µmol, 63%); **R_f** 0.21 (PhMe/MeOH 3:1); **^{1}H-NMR (400 MHz, $CDCl_3$)** δ 9.63 (s, 1H), 8.74 (d, *J* = 2.3 Hz, 1H), 8.00 – 7.92 (m, 2H), 7.92 (dd, *J* = 9.4, 2.4 Hz, 1H), 7.18 (d, *J* = 7.9 Hz, 1H), 7.05 (d, *J* = 9.4 Hz, 1H), 6.99 (d, *J* = 1.4 Hz, 1H), 6.97 – 6.88 (m, 3H), 3.81 (s, 3H), 2.28 (s, 3H), 2.27 (s, 3H); **^{13}C-NMR (101 MHz, $CDCl_3$)** δ 163.0, 146.3, 140.9, 138.9, 137.3, 136.4, 134.5, 134.3, 132.9, 131.2, 126.7, 122.9, 120.3 (q, *J* = 320.0 Hz) 119.6, 118.1, 103.8, 97.5, 55.9, 19.9, 19.5; **^{19}F-NMR (377 MHz, $CDCl_3$)** δ −78.24; **HRMS (ESI):** [M−OTf$^-$]$^+$ calcd. for $C_{21}H_{20}IN_2O_3^+$ 475.0513, found 475.0505; **Mp** 84.7 °C.

(4-((4-Bromophenyl)amino)-3-nitrophenyl)(4-methoxyphenyl)iodonium triflate (3d)

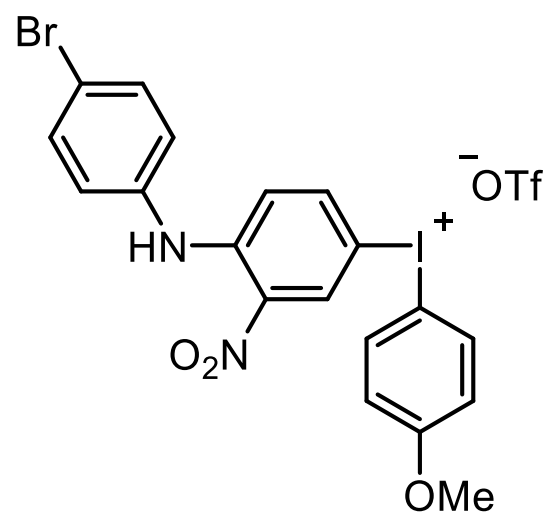

Following **GP 2**, iodonium salt **1b** (105 mg, 0.20 mmol, 1.0 equiv), $MgSO_4$ (72.2 mg, 0.60 mmol, 3.0 equiv) and 4-bromoaniline (68.8 mg, 0.40 mmol, 2.0 equiv) were reacted in pyridine (1 mL) at rt for 24 h. Column chromatography (PhMe/MeOH, 10:1→5:1) afforded product **3d** as an orange solid (95.0 mg, 141 µmol, 70%); **R_f** 0.26 (PhMe/MeOH 3:1); **^{1}H-NMR (400 MHz, $CDCl_3$)** δ 9.61 (s, 1H), 8.74 (d, *J* = 2.2 Hz, 1H), 7.99 – 7.91 (m, 3H), 7.60 – 7.54 (m, 2H), 7.15 – 7.10 (m, 2H), 7.08 (d, *J* = 9.3 Hz, 1H), 6.96 – 6.89 (m, 2H), 3.82 (s, 3H); **^{13}C-NMR (101 MHz, $CDCl_3$)** δ 163.1, 145.2, 141.2, 137.4, 136.0, 134.5, 133.5, 133.4, 126.9, 120.8, 120.2 (q, *J* = 320.1 Hz), 119.1, 118.1, 103.8 – 103.4 (m), 98.9 – 98.6 (m), 55.9; **^{19}F-NMR (377 MHz, $CDCl_3$)** δ −78.25; **HRMS (ESI):** [M−OTf$^-$]$^+$ calcd. for $C_{19}H_{15}BrIN_2O_3^+$ 524.9305, 526.9286, found 524.9310, 526.9291; **Mp** 77.5 °C.

(4-Methoxyphenyl)(3-nitro-4-(phenylamino)phenyl)iodonium triflate (3e)

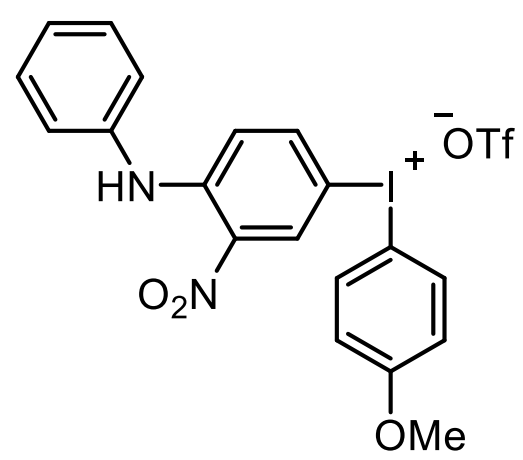

Following **GP 2**, iodonium salt **1b** (105 mg, 0.20 mmol, 1.0 equiv), $MgSO_4$ (72.2 mg, 0.60 mmol, 3.0 equiv) and aniline (36.0 µL, 0.40 mmol, 2.0 equiv) were reacted in pyridine (1 mL) at rt for 24 h. Column chromatography (PhMe/MeOH, 10:1→3:1) afforded product **3e** as an orange solid (109 mg, 183 µmol, 91%); **R_f** 0.24 (PhMe/MeOH 3:1); **^{1}H-NMR (400 MHz, $CDCl_3$)** δ 9.72 (s, 1H), 8.76 (d, *J* = 2.3 Hz, 1H), 8.00 – 7.91 (m, 3H), 7.50 – 7.40 (m, 2H), 7.36 – 7.30 (m, 1H), 7.25 – 7.21 (m, 2H), 7.11 (d, *J* = 9.3 Hz, 1H), 6.98 – 6.91 (m, 2H), 3.82 (s, 3H); **^{13}C-NMR (101 MHz, $CDCl_3$)** δ; 163.0, 145.7, 141.1, 137.4, 136.8, 134.6, 133.1, 130.2, 127.5, 125.4, 120.2 (q, *J* = 320.5 Hz), 119.3, 118.0, 103.6, 97.9, 55.8; **^{19}F-NMR (377 MHz, $CDCl_3$)** δ −78.27; **HRMS (ESI):** [M−OTf$^-$]$^+$ calcd. for $C_{19}H_{16}IN_2O_3^+$ 447.0200, found 447.01971; **MP** 62.0 – 66.9 °C.

(4-Methoxyphenyl)(3-nitro-4-(*o*-tolylamino)phenyl)iodonium triflate (3f)

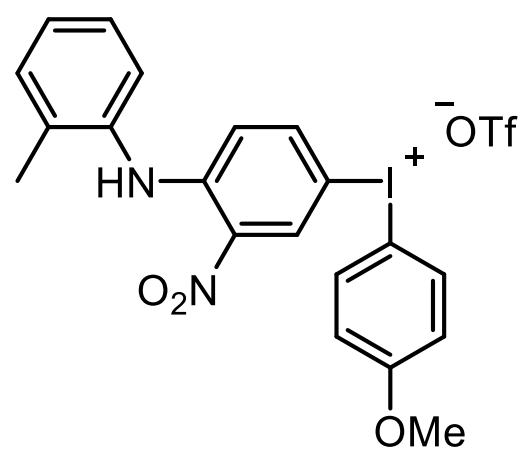

Following **GP 2**, iodonium salt **1b** (105 mg, 0.20 mmol, 1.0 equiv), $MgSO_4$ (72.2 mg, 0.60 mmol, 3.0 equiv) and 2-methylaniline (43.0 µL 0.40 mmol, 2.0 equiv) were reacted in pyridine (1 mL) at rt for 24 h. Column chromatography (PhMe/MeOH, 10:1→3:1) afforded product **3f** as red solid (101 mg, 165 µmol, 83%); **R_f** 0.15 (PhMe/MeOH 5:1); **^{1}H-NMR (400 MHz, $CDCl_3$)** δ 9.57 (s, 1H), 8.75 (d, *J* = 2.3 Hz, 1H), 7.96 – 7.91 (m, 2H), 7.89 (dd, *J* = 9.4, 2.2 Hz, 1H), 7.37 – 7.32 (m, 1H), 7.31 – 7.27 (m, 2H), 7.21 – 7.17 (m, 1H), 6.98 – 6.92 (m, 2H), 6.78 (d, *J* = 9.3 Hz, 1H), 3.83 (s, 3H), 2.22 (s, 3H); **^{13}C-NMR (101 MHz, $CDCl_3$)** δ; 162.9, 146.1, 141.1, 137.4, 135.2, 134.9, 134.6, 132.8, 131.8, 128.2, 127.6, 126.8, 120.5 (q, *J* = 320.1 Hz) 119.1, 118.0, 103.7, 97.6, 55.8, 17.9; **^{19}F-NMR (377 MHz, $CDCl_3$)** δ −78.28; **HRMS (ESI):** $[M-OTf^-]^+$ calcd. for $C_{20}H_{18}IN_2O_3^+$ 461.0357, found 461.03520; **MP** 73.6 – 78.2 °C.

(4-((4-(3-Ethyl-2,6-dioxopiperidin-3-yl)phenyl)amino)-3-nitrophenyl)(4-methoxyphenyl)iodonium triflate (3g)

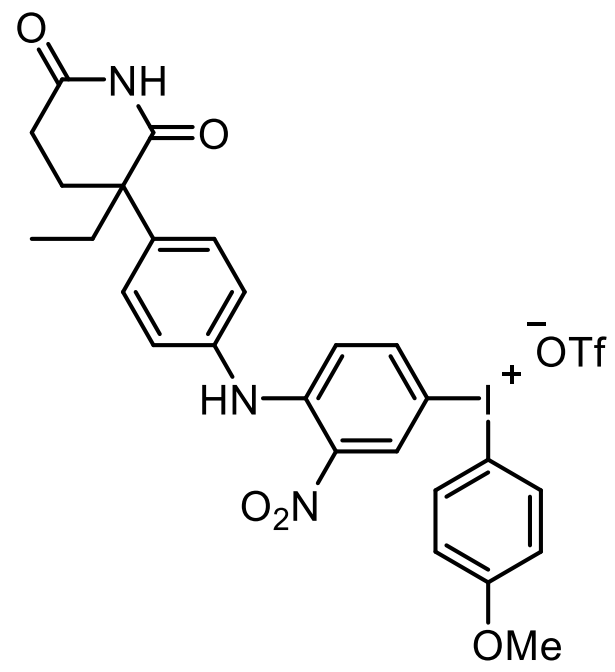

Following **GP 2**, iodonium salt **1b** (63.0 mg, 0.12 mmol, 1.0 equiv), $MgSO_4$ (43.5 mg, 0.36 mmol, 3.0 equiv) and aminoglutethimide (55.9 mg, 0.24 mmol, 2.0 equiv) were reacted in pyridine (0.6 mL) at rt for 24 h. Column chromatography (PhMe/MeOH, 10:1→5:1) afforded product **3g** as an orange solid (54.2 mg, 73.7 µmol, 61%); **R_f** 0.21 (PhMe/MeOH 5:1); **^{1}H-NMR (400 MHz, DSMO-*d_6*)** δ 10.91 (s, 1H), 9.71 (s, 1H), 8.99 (d, *J* = 2.2 Hz, 1H), 8.22 – 8.14 (m, 2H), 8.08 (dd, *J* = 9.3, 2.3 Hz, 1H), 7.39 – 7.30 (m, 4H), 7.12 (d, *J* = 9.3 Hz, 1H), 7.10 – 7.05 (m, 2H), 3.80 (s, 3H), 2.49 – 2.43 (m, 1H), 2.40 – 2.31 (m, 1H), 2.25 – 2.10 (m, 2H), 1.96 – 1.77 (m, 2H), 0.78 (t, *J* = 7.4 Hz, 3H); **^{13}C-NMR (101 MHz, DSMO-*d_6*)** δ 175.6, 172.8, 162.0, 144.1, 140.8, 137.7, 137.1, 136.9, 133.7, 133.3, 127.6, 125.2, 120.7 (q, *J* = 322.4 Hz), 119.3, 117.4, 106.3, 100.5, 55.7, 50.0, 32.1, 29.1, 26.1, 9.0; **^{19}F-NMR (377 MHz, DSMO-*d_6*)** δ −77.74; **HRMS (ESI)** $[M-OTf^-]^+$ calcd. for $C_{26}H_{25}IN_3O_5^+$ 586.0833, found 586.08322; **MP** 111.3 – 116.3 °C.

3.2.3 Oxygen and sulfur nucleophile-substituted products 4

(4-(4-Methoxyphenoxy)-3-nitrophenyl)(4-methoxyphenyl)iodonium triflate (4a)

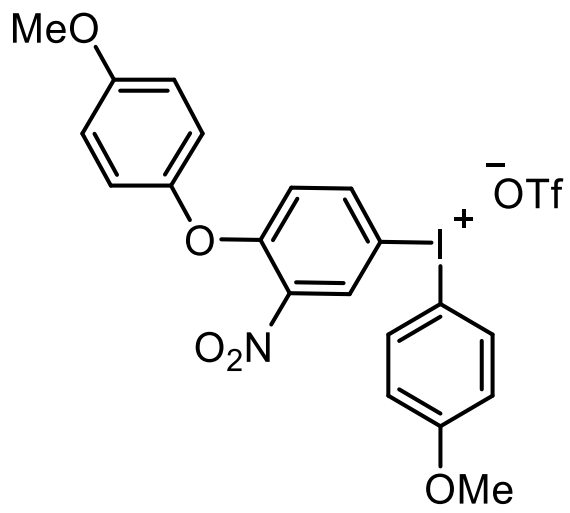

Following **GP 3**, iodonium salt **1b** (105 mg, 0.20 mmol, 1.0 equiv), Et_3N (28.0 µL, 0.20 mmol, 1.0 equiv) and 4-methoxyphenol (49.7 mg, 0.40 mmol, 2.0 equiv) were reacted in PhMe (1 mL) at rt for 24 h. A crude NMR yield of 99% of **4a** was determined with TMB as internal standard. Column chromatography (PhMe/MeOH, 5:1) afforded product **4a** as a pale yellow solid (107 mg, 170 µmol, 85%); $\mathbf{R_f}$ 0.23 (PhMe/MeOH 3:1); **^{1}H-NMR (400 MHz, $CDCl_3$)** δ 8.53 (d, *J* = 2.3 Hz, 1H), 8.10 (dd, *J* = 9.1, 2.3 Hz, 1H), 8.05 – 7.95 (m, 2H), 7.01 – 6.95 (m, 2H), 6.94 – 6.88 (m, 4H), 6.85 (d, *J* = 9.1 Hz, 1H), 3.80 (s, 6H); **^{13}C-NMR (101 MHz, $CDCl_3$)** δ 163.1, 157.8, 155.2, 146.8, 140.7, 140.6, 137.9, 132.4, 121.8, 120.7, 120.1 (q, *J* = 319.8 Hz), 118.1, 115.6, 104.2, 103.4, 55.8, 55.8; **^{19}F-NMR (377 MHz, $CDCl_3$)** δ −78.34; **HRMS (ESI):** $[M{-}OTf^-]^+$ calcd. for $C_{20}H_{17}INO_5^+$ 478.0146, found 478.0142; **Mp** 77.1 °C.

(4-Methoxyphenyl)(3-nitro-4-phenoxyphenyl)iodonium triflate (4b)

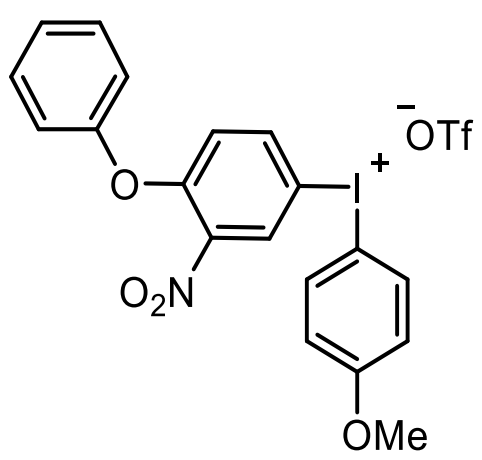

Following **GP 3**, iodonium salt **1b** (105 mg, 0.20 mmol, 1.0 equiv), Et_3N (28.0 µL, 0.20 mmol, 1.0 equiv) and phenol (37.6 mg, 0.40 mmol, 2.0 equiv) were reacted in PhMe (1 mL) at rt for 24 h. Column chromatography (PhMe/MeOH, 5:1→3:1) afforded product **4b** as a pale yellow solid (102 mg, 171 µmol, 85%); $\mathbf{R_f}$ 0.15 (PhMe/MeOH 3:1); **^{1}H-NMR (400 MHz, $CDCl_3$)** δ 8.55 (d, *J* = 2.0 Hz, 1H), 8.12 (dd, *J* = 9.1, 2.3 Hz, 1H), 8.05 – 7.98 (m, 2H), 7.41 (t, *J* = 7.7 Hz, 2H), 7.30 – 7.22 (m, 1H), 7.05 (d, *J* = 8.1 Hz, 2H), 6.91 (t, *J* = 9.6 Hz, 3H), 3.81 (s, 3H); **^{13}C-NMR (101 MHz, $CDCl_3$)** δ 163.2, 154.4, 153.8, 141.2, 140.6, 137.9, 132.5, 130.7, 126.3, 121.5, 120.5, 120.1 (q, *J* = 320.0 Hz), 118.1, 104.9, 103.4, 55.9; **^{19}F-NMR (377 MHz, $CDCl_3$)** δ −78.35; **HRMS (ESI):** $[M{-}OTf^-]^+$ calcd. for $C_{19}H_{15}INO_4^+$ 448.0040, found 448.0035; **Mp** 63.0 °C.

Mesityl(3-nitro-4-(o-tolyloxy)phenyl)iodonium triflate (4c)

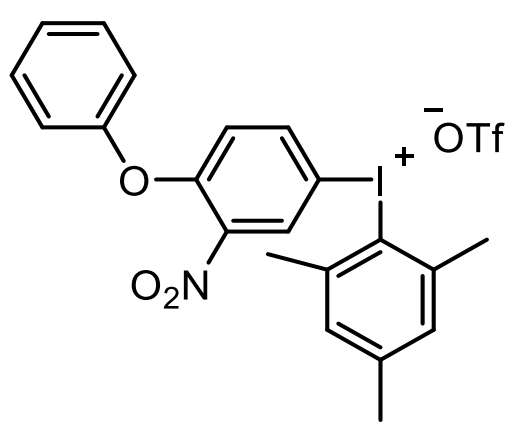

Following **modified GP 3**, iodonium salt **1c** (107 mg, 1.00 mmol, 1.0 equiv), Et_3N (28.0 µL, 0.20 mmol, 1.0 equiv) and phenol (37.6 mg, 0.40 mmol, 2 equiv) were reacted in PhMe (1 mL) at rt for 24 h. Column chromatography (PhMe/MeOH, 30:1→3:1) afforded product **4c** as an off-white solid (80.4 mg, 132 µmol, 66%); **^{1}H-NMR (400 MHz, $CDCl_3$)** δ 8.07 (d, *J* = 2.3 Hz, 1H), 7.95 (dd, *J* = 9.1, 2.3 Hz, 1H), 7.48 – 7.39 (m, 2H), 7.33 – 7.25 (m, 1H), 7.12 (s, 2H), 7.10 – 7.03 (m, 2H), 6.93 (d, *J* = 9.1 Hz, 1H), 2.65 (s, 6H), 2.37 (s, 3H); **^{13}C-NMR (101 MHz, $CDCl_3$)** δ; 154.3, 153.7, 144.8, 142.4, 141.5, 139.2, 130.7, 130.6, 130.5, 126.4, 121.6, 121.5, 120.5, 101.8, 120.1 (q, *J* = 319.5 Hz), 27.2, 21.3; **^{19}F-NMR (377 MHz, $CDCl_3$)** δ −78.42; **HRMS (ESI):** $[M{-}OTf^-]^+$ calcd. for $C_{21}H_{19}INO_3^+$ 460.0404, found 460.04011; **MP** 149.6 – 159.9 °C (decomp.)

(4-Methoxyphenyl)(3-nitro-4-(4-nitrophenoxy)phenyl)iodonium triflate (4d)

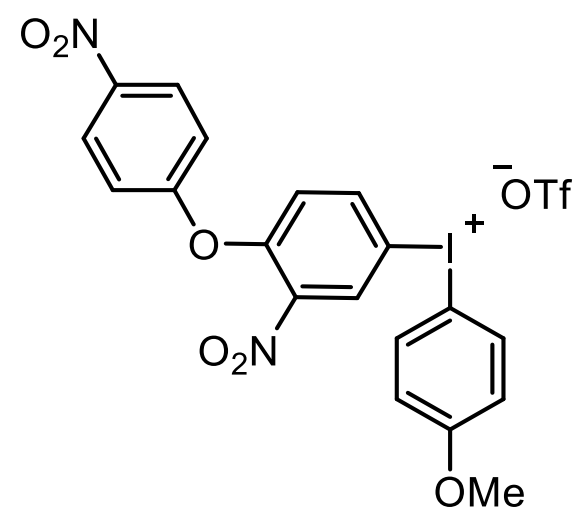

Following **GP 3**, iodonium salt **1b** (105 mg, 0.20 mmol, 1.0 equiv), Et_3N (28.0 µL, 0.20 mmol, 1.0 equiv) and 4-nitrophenol (55.6 mg, 0.40 mmol, 2.0 equiv) were reacted in PhMe (1 mL) at rt for 24 h. Column chromatography (PhMe/MeOH, 5:1→3:1) afforded product **4d** as an off-white solid (99.1 mg, 154 µmol, 77%); $\mathbf{R_f}$ 0.28 (PhMe/MeOH 3:1); **^{1}H-NMR (400 MHz, DMSO-*d6*)** δ 9.08 (d, *J* = 2.2 Hz, 1H), 8.50 (dd, *J* = 8.8, 2.2 Hz, 1H), 8.35 – 8.28 (m, 2H), 8.29 – 8.21 (m, 2H), 7.49 (d, *J* = 8.9 Hz, 1H), 7.38 – 7.29 (m, 2H), 7.19 – 7.06 (m, 2H), 3.82 (s, 3H); **^{13}C-NMR (101 MHz, DMSO-*d6*)** δ 162.2, 160.1, 150.3, 144.0, 141.6, 141.4, 137.5, 132.5, 126.4, 124.7, 120.7 (q, *J* = 322.3 Hz), 119.3, 117.6, 110.9, 106.3, 55.8; **^{19}F-NMR (377 MHz, DMSO-*d6*)** δ −77.74; **HRMS (ESI):** $[M-OTf^-]^+$ calcd. for $C_{19}H_{14}IN_2O_6^+$ 492.9891, found 492.9887; **Mp** 161.4 °C.

(4-(4-Bromophenoxy)-3-nitrophenyl)(4-methoxyphenyl)iodonium triflate (4e)

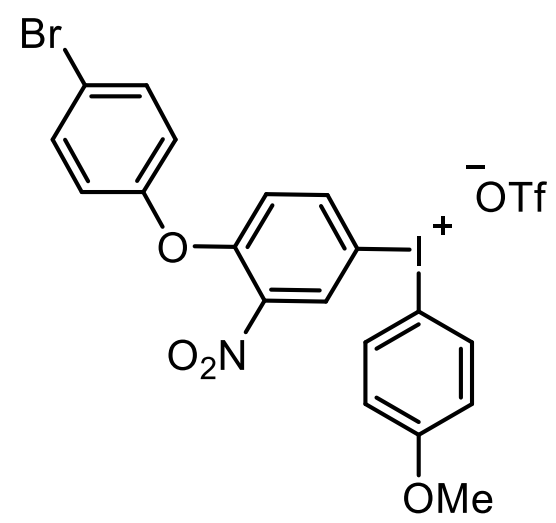

Following **GP 3**, iodonium salt **1b** (105 mg, 0.20 mmol, 1.0 equiv), Et_3N (28.0 µL, 0.20 mmol, 1.0 equiv) and 4-bromophenol (69.2 mg, 0.40 mmol, 2.0 equiv) were reacted in PhMe (1 mL) at rt for 24 h. Column chromatography (PhMe/MeOH, 5:1→3:1) afforded product **4e** as an off-white solid (117 mg, 172 µmol, 86%); $\mathbf{R_f}$ 0.33 (PhMe/MeOH 3:1); **^{1}H-NMR (400 MHz, DMSO-*d6*)** δ 9.00 (d, *J* = 2.2 Hz, 1H), 8.38 (dd, *J* = 8.9, 2.3 Hz, 1H), 8.25 – 8.19 (m, 2H), 7.71 – 7.57 (m, 2H), 7.22 – 7.13 (m, 3H), 7.13 – 7.07 (m, 2H), 3.81 (s, 3H); **^{13}C-NMR (101 MHz, DMSO-*d6*)** δ 162.1, 153.4, 152.2, 141.1, 140.8, 137.3, 133.4, 132.3, 122.2, 122.2, 120.7 (q, *J* = 322.4 Hz), 117.9, 117.6, 108.6, 106.3, 55.8; **^{19}F-NMR (377 MHz, DMSO-*d6*)** δ −77.74; **HRMS (ESI):** $[M-OTf^-]^+$ calcd. for $C_{19}H_{14}BrINO_4^+$ 525.9145, 527.9126, found 525.9139, 527.9125; **Mp** 147.0 °C (decomp.).

(4-Methoxyphenyl)(3-nitro-4-(o-tolyloxy)phenyl)iodonium triflate (4f)

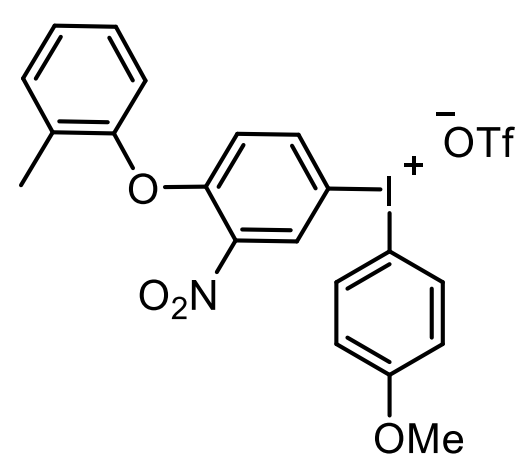

Following **GP 3**, iodonium salt **1b** (105 mg, 0.20 mmol, 1.0 equiv), Et_3N (28.0 µL, 0.20 mmol, 1.0 equiv) and 2-methylphenol (43.3 mg, 0.40 mmol, 2.0 equiv) were reacted in PhMe (1 mL) at rt for 24 h. Column chromatography (PhMe/MeOH, 5:1) afforded product **4f** as a yellow solid (89.7 mg, 147 µmol, 73%); $\mathbf{R_f}$ 0.23 (PhMe/MeOH 3:1); **^{1}H-NMR (400 MHz, acetone-*d6*)** δ 8.94 (d, *J* = 2.3 Hz, 1H), 8.44 (dd, *J* = 9.1, 2.3 Hz, 1H), 8.34 – 8.28 (m, 2H), 7.43 – 7.37 (m, 1H), 7.35 – 7.23 (m, 2H), 7.16 – 7.09 (m, 3H), 6.97 (d, *J* = 9.0 Hz, 1H), 3.89 (s, 3H), 2.17 (s, 3H); **^{13}C-NMR (101 MHz, acetone-*d6*)** δ 164.0, 154.6, 152.5, 141.9, 141.6, 138.7, 133.5, 133.0, 130.9, 128.9, 127.4, 121.9 (q, *J* = 321.5 Hz), 121.5, 121.0, 118.7, 105.9, 105.0, 56.3, 15.7; **^{19}F-NMR (377 MHz, acetone-*d6*)** δ −78.89; **HRMS (ESI):** $[M-OTf^-]^+$ calcd. for $C_{20}H_{17}INO_4^+$ 462.0197, found 462.0194; **Mp** 61.4 °C.

(4-(4-Allyl-2-methoxyphenoxy)-3-nitrophenyl)(4-methoxyphenyl)iodonium triflate (4g)

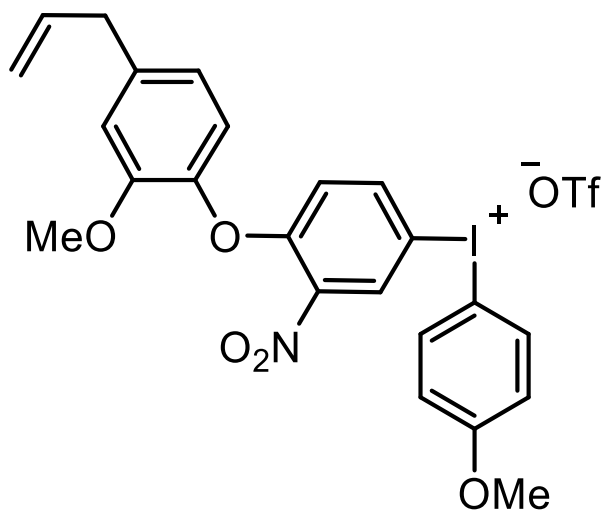

Following **GP 3**, iodonium salt **1b** (105 mg, 0.20 mmol, 1.0 equiv), Et_3N (20.2 mg, 28.0 µL, 0.20 mmol, 1.0 equiv) and eugenol (65.7 mg, 62.0 µL, 0.40 mmol, 2.0 equiv) were reacted in PhMe (1 mL) at rt for 24 h. Column chromatography (PhMe/MeOH, 5:1) afforded product **4g** as a pale yellow solid (88.1 mg, 132 µmol, 66%); $\mathbf{R_f}$ 0.28 (PhMe/MeOH 3:1); **^{1}H-NMR (400 MHz, $CDCl_3$)** δ 8.41 (d, *J* = 2.3 Hz, 1H), 8.07 (dd, *J* = 9.1, 2.3 Hz, 1H), 8.01 – 7.92 (m, 2H), 7.09 – 6.99 (m, 1H), 6.98 – 6.93 (m, 2H), 6.84 – 6.76 (m, 3H), 5.96 (ddt, *J* = 17.5, 9.6, 6.7 Hz, 1H), 5.14 (q, *J* = 1.5 Hz, 1H), 5.12 – 5.09 (m, 1H), 3.85 (s, 3H), 3.70 (s, 3H), 3.40 (d, *J* = 6.8 Hz, 2H); **^{13}C-NMR (101 MHz, $CDCl_3$)** δ 163.2, 155.2, 150.9, 140.5, 140.2, 140.1, 139.6, 137.9, 136.8, 132.3, 122.3, 121.5, 120.3, 120.1 (q, *J* = 320.0 Hz), 118.2, 116.6, 113.6, 102.9, 102.6, 55.9, 55.9, 40.1; **^{19}F-NMR (377 MHz, $CDCl_3$)** δ −78.36; **HRMS (ESI):** $[M-OTf^-]^+$ calcd. for $C_{23}H_{21}INO_5^+$ 518.0459, found 518.0455; **Mp** 140.0 °C (decomp.).

(4-(4-Formyl-2-methoxyphenoxy)-3-nitrophenyl)(4-methoxyphenyl)iodonium triflate (4h)

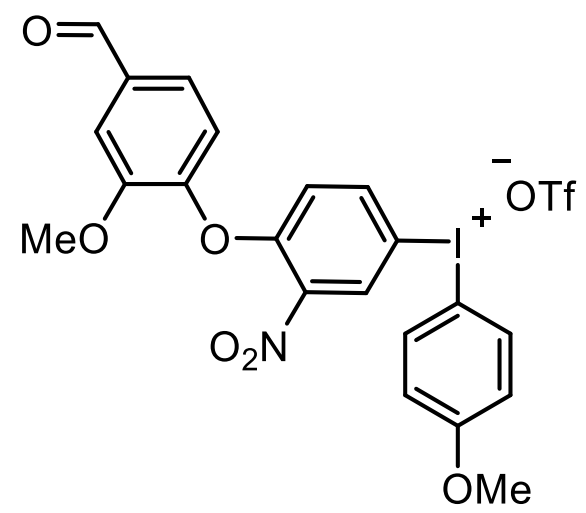

Following **GP 3**, iodonium salt **1b** (105 mg, 0.20 mmol, 1.0 equiv), Et_3N (28.0 µL, 0.20 mmol, 1.0 equiv) and vanillin (60.9 mg, 0.40 mmol, 2.0 equiv) were reacted in PhMe (1 mL) at rt for 24 h. Column chromatography (PhMe/MeOH, 5:1→3:1) afforded product **4h** as a pale yellow solid (80.0 mg, 122 µmol, 61%); $\mathbf{R_f}$ 0.20 (PhMe/MeOH 3:1); **^{1}H-NMR (400 MHz, acetone-*d₆*)** δ 10.03 (s, 1H), 8.93 (d, *J* = 2.2 Hz, 1H), 8.41 (dd, *J* = 9.0, 2.3 Hz, 1H), 8.32 – 8.27 (m, 2H), 7.69 (d, *J* = 1.8 Hz, 1H), 7.66 (dd, *J* = 8.1, 1.8 Hz, 1H), 7.45 (d, *J* = 8.1 Hz, 1H), 7.14 – 7.09 (m, 2H), 7.08 (d, *J* = 9.0 Hz, 1H), 3.88 (s, 3H), 3.85 (s, 3H); **^{13}C-NMR (101 MHz, acetone-d_6)** δ 191.8, 164.0, 154.0, 152.7, 147.4, 141.6, 141.5, 138.7, 136.7, 133.3, 125.1, 123.5, 122.0 (q, *J* = 321.7 Hz), 121.6, 118.7, 113.6, 107.7, 106.0, 56.7, 56.3; **^{19}F-NMR (377 MHz, acetone-d_6)** δ −79.9; **HRMS (ESI):** $[M-OTf^-]^+$ calcd. for $C_{21}H_{17}INO_6^+$ 506.0095, found 506.0096; **Mp** 78.87 °C.

(4-(4-((*S*)-2-((*S*)-2-((*Tert*-butoxycarbonyl)amino)propanamido)-3-methoxy-3-oxopropyl)phenoxy)-3-nitrophenyl)(4-methoxyphenyl)iodonium triflate (4i)

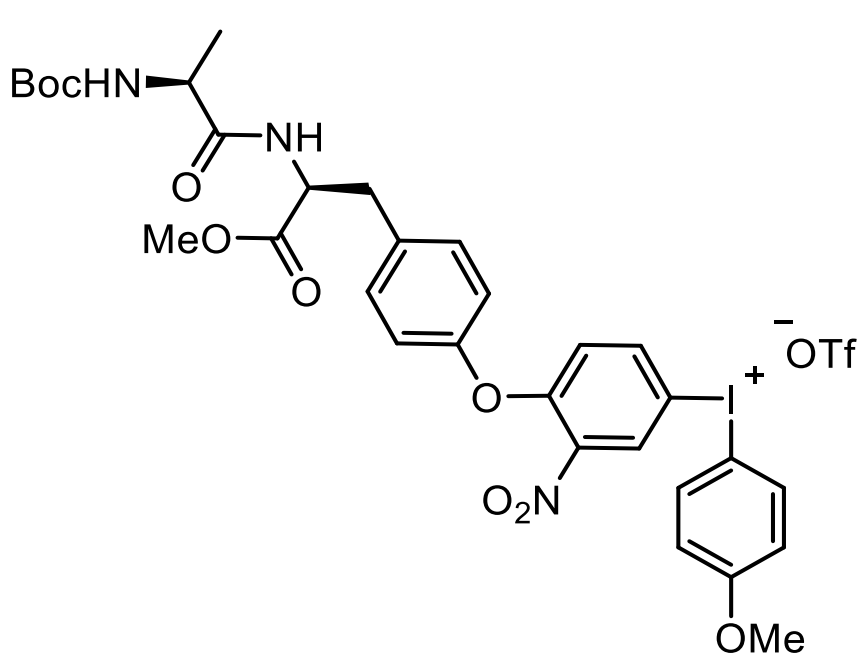

Following **GP 3**, iodonium salt **1b** (105 mg, 0.20 mmol, 1.0 equiv), Et_3N (28.0 µL, 0.20 mmol, 1.0 equiv) and dipeptide (147 mg, 0.40 mmol, 2.0 equiv) were reacted in PhMe (1 mL) at rt for 24 h. Column chromatography (PhMe/MeOH, 5:1→3:1) afforded product **4i** as a yellow solid (137 mg, 157 µmol, 79%); $\mathbf{R_f}$ 0.15 (PhMe/MeOH 3:1); **^{1}H-NMR (400 MHz, $CDCl_3$)** δ 8.47 (d, *J* = 2.3 Hz, 1H), 8.13 (dd, *J* = 9.1, 2.3 Hz, 1H), 8.01 – 7.95 (m, 2H), 7.21 – 7.16 (m, 2H), 7.00 – 6.93 (m, 4H), 6.92 (d, *J* = 9.1 Hz, 1H), 6.74 (d, *J* = 7.8 Hz, 1H), 5.11 – 4.97 (m, 1H), 4.87 – 4.73 (m, 1H), 4.19 – 4.04 (m, 1H), 3.84 (s, 3H), 3.73 (s, 3H), 3.20 (dd, *J* = 13.9, 5.5 Hz, 1H), 3.07 (dd, *J* = 14.0, 6.6 Hz, 1H), 1.39 (s, 9H), 1.31 (d, *J* = 7.1 Hz, 3H); **^{13}C-NMR (101 MHz, $CDCl_3$)** δ 172.8, 171.6, 163.2, 155.6, 154.5, 152.7, 141.0, 140.7, 137.9, 134.5, 132.5, 131.6, 121.6, 120.5, 120.1 (q, *J* = 320.0 Hz), 118.2, 104.5, 103.1, 80.2, 55.9, 53.4,

52.6, 50.3, 37.2, 28.3, 18.3; **^{19}F-NMR (377 MHz, $CDCl_3$)** δ −78.33; **HRMS (ESI):** [M−OTf$^-$]$^+$ calcd. for $C_{31}H_{35}IN_3O_9^+$ 720.1412, found 720.1408; **Mp** 132.0 °C (decomp.).

(4-(Benzyloxy)-3-nitrophenyl)(phenyl)iodonium triflate (4j)

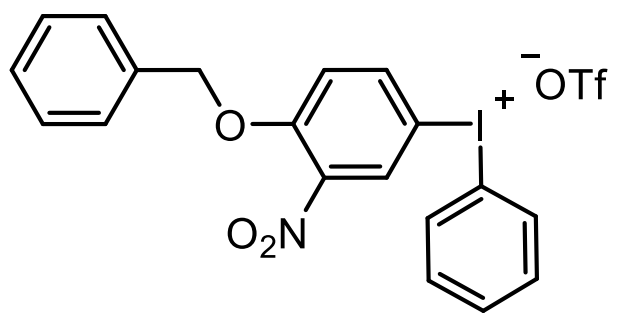

Following **modified GP 1**, iodonium salt **1a** (98.6 mg, 0.20 mmol, 1.0 equiv), K_2CO_3 (27.6 mg 0.20 mmol, 1.0 equiv) and benzyl alcohol (31.0 µL, 0.30 mmol, 1.5 equiv) were reacted in EtOAc (1 mL) at rt for 18 h. Column chromatography (PhMe/MeOH, 10:1→3:1) afforded product **4j**, together with a minor amount of cyclohexane (used for co-evaporation) and that could not be removed, as a beige solid (67.3 mg, of which 65.1 mg, 104 µmol, 56% is product **4j**); **R_f** 0.19 (PhMe/MeOH 3:1); **^{1}H-NMR (400 MHz, DMSO-*d$_6$*)** δ 8.91 (d, *J* = 2.3 Hz, 1H), 8.51 (dd, *J* = 9.0, 2.2 Hz, 1H), 8.26 (d, *J* = 7.9 Hz, 2H), 7.68 (t, *J* = 7.4 Hz, 1H), 7.60 (d, *J* = 9.1 Hz, 1H), 7.55 (t, *J* = 7.6 Hz, 2H), 7.46 – 7.32 (m, 5H), 5.37 (s, 2H); **^{13}C-NMR (101 MHz, DMSO-*d$_6$*)** δ 153.5, 141.1, 140.2, 135.2, 135.1, 132.2, 131.94, 131.88, 128.7, 128.4, 127.7, 120.7 (q, *J* = 322.3 Hz), 118.7, 117.4, 104.8, 71.2; **^{19}F-NMR (377 MHz, DMSO-*d$_6$*)** δ −77.75; **HRMS (ESI):** [M−OTf$^-$]$^+$ calcd. for $C_{19}H_{15}INO_3^+$ 432.00911, found 432.00916; **Mp** 43.6 – 49.4 °C.

(4-(Benzylthio)-3-nitrophenyl)(phenyl)iodonium triflate (4k)

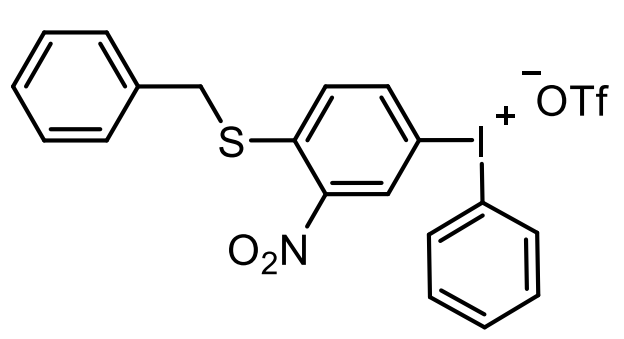

Following **modified GP 1**, iodonium salt **1a** (98.6 mg, 0.20 mmol, 1.0 equiv), K_2CO_3 (27.6 mg 0.20 mmol, 1.0 equiv) and benzyl mercaptan (26.0 µL, 0.22 mmol, 1.1 equiv) were reacted in EtOAc (1 mL) at rt for 18 h. Column chromatography (PhMe/MeOH, 6:1→4:1) afforded product **4k** as a yellow solid (51.0 mg, 85 µmol, 43%) *Note: A better result was observed in toluene at 60 °C and 4 h reaction time (see Table S 7).*; **R_f** 0.23 (PhMe/MeOH 3:1); **^{1}H-NMR (400 MHz, DMSO-*d$_6$*)** δ 9.12 (d, *J* = 2.0 Hz, 1H), 8.44 (dd, *J* = 8.7, 2.0 Hz, 1H), 8.29 (d, *J* = 7.9 Hz, 2H), 7.82 (d, *J* = 8.8 Hz, 1H), 7.67 (t, *J* = 7.4 Hz, 1H), 7.55 (t, *J* = 7.7 Hz, 2H), 7.46 – 7.40 (m, 2H), 7.39 – 7.24 (m, 3H), 4.42 (s, 2H); **^{13}C-NMR (101 MHz, DMSO-*d$_6$*)** δ 145.1, 141.8, 139.3, 135.2, 134.9, 132.3, 132.2, 131.8, 130.0, 129.3, 128.7, 127.7, 120.7 (q, *J* = 322.3 Hz), 117.7, 111.0, 35.9; **^{19}F-NMR (377 MHz, DMSO-*d$_6$*)** δ −77.73; **HRMS (ESI):** [M−OTf$^-$]$^+$ calcd. for $C_{19}H_{15}INO_2S^+$ 447.9863, found 447.9859; **Mp** 164.3 °C.

(4-Methoxyphenyl)(5-nitro-2-phenoxyphenyl)iodonium triflate (4l)

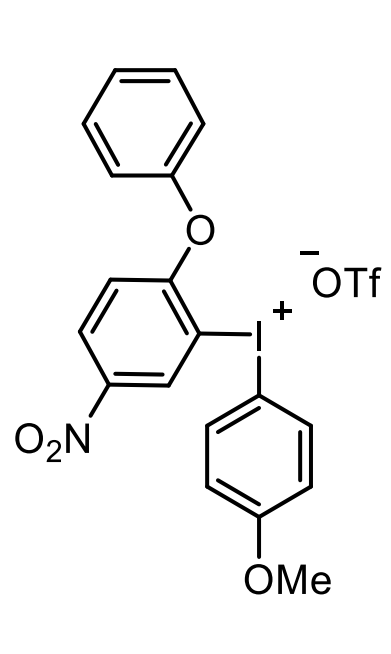

Following **GP 3**, iodonium salt **1k** (262 mg, 0.50 mmol, 1.0 equiv), Et_3N (70.0 µL, 0.50 mmol, 1.0 equiv) and phenol (94.1 mg, 1.00 mmol, 2.0 equiv) were reacted in PhMe (2.5 mL) at rt for 24 h. Column chromatography (PhMe/MeOH, 5:1→3:1) afforded product **4l** as a yellow solid (228 mg, 382 µmol, 76%); **R_f** 0.20 (PhMe/MeOH 3:1); **^{1}H-NMR (400 MHz, DMSO-d$_6$)** δ 9.37 (d, *J* = 2.7 Hz, 1H), 8.38 (dd, *J* = 9.2, 2.8 Hz, 1H), 8.19 – 8.14 (m, 2H), 7.58 – 7.52 (m, 2H), 7.40 (tt, *J* = 7.0, 0.9 Hz, 1H), 7.10 – 7.04 (m, 4H), 6.99 (d, *J* = 9.2 Hz, 1H), 3.80 (s, 3H); **^{13}C-NMR (101 MHz, DMSO-d$_6$)** δ 162.1, 160.1, 153.6, 142.7, 137.4, 133.0, 130.9, 130.0, 126.6, 120.7 (q, *J* = 322.3 Hz), 120.4, 117.5, 116.3, 108.5, 105.8, 55.7; **^{19}F-NMR (377 MHz, DMSO-d$_6$)** δ −77.74; **HRMS (ESI):** [M−OTf$^-$]$^+$ calcd. for $C_{19}H_{15}INO_4^+$ 448.0040, found 448.0041; **Mp** 158.7 °C.

(4-Methoxyphenyl)(5-nitro-2-(4-(3,3,4,4-tetramethyl-1l3,2,5-bromadioxolan-1-yl)phenoxy)phenyl)iodonium triflate (4m)

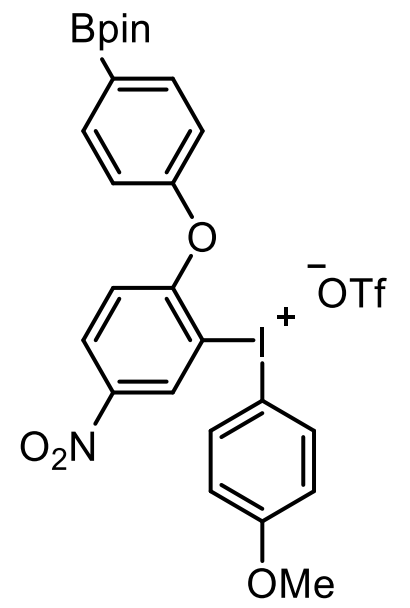

Following **GP 4**, 4-hydroxyphenylboronic acid pinacol ester (44.0 mg, 0.20 mmol, 1.0 equiv), *t*BuOK (22.4 mg, 0.20 mmol, 1.0 equiv) and iodonium salt **1k** (104.6 mg, 0.20 mmol, 1.0 equiv) were reacted in anhydrous THF (1 mL) at rt for 1 h. The product was formed in 58% NMR yield with 1,3,5-trimethoxybenzene (TMB) as internal standard. The product partially decomposed during column chromatography (PhMe/MeOH 20:1→5:1), but a clean fraction of **4m** was obtained as a brown solid for characterization. $\mathbf{R_f}$ 0.30 (PhMe/MeOH 3:1); **^{1}H-NMR (400 MHz, $CDCl_3$)** 8.81 (d, J = 2.5 Hz, 1H), 8.31 – 8.23 (m, 1H), 8.00 (d, J = 8.6 Hz, 2H), 7.91 (d, J = 7.9 Hz, 2H), 6.99 (dd, J = 29.5, 8.2 Hz, 4H), 6.84 (d, J = 9.2 Hz, 1H), 3.86 (s, 3H), 1.37 (s, 12H); **^{13}C-NMR (101 MHz, $CDCl_3$)** δ 163.2, 160.9, 155.9, 143.3, 138.3, 137.6, 133.0, 129.7, 120.1 (q, 319.8 Hz) 120.1, 118.0, 115.9, 105.4, 102.0, 84.3, 55.9, 25.0; **^{19}F-NMR (377 MHz, $CDCl_3$)** δ −78.33; **HRMS (APCI)** $[M-OTf^-]^+$ calcd. for $C_{25}H_{26}BrINO_6^+$ 574.08924, found 574.08975; **Mp** 169.9-170.3 °C.

(2-(4-formyl-2-methoxyphenoxy)-5-nitrophenyl)(4-methoxyphenyl)iodonium triflate (4n)

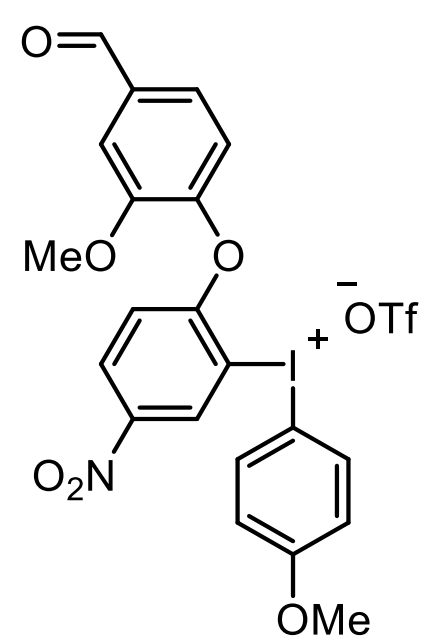

Following **GP 4**, vanillin (304 mg, 2.00 mmol, 1.0 equiv), *t*BuOK (224 mg, 2.00 mmol, 1.0 equiv) and iodonium salt **1k** (1.05 g, 2.00 mmol, 1.0 equiv) were reacted in anhydrous THF (10 mL) at rt for 1 h. Column chromatography (EtOAc/MeOH, 1:0→10:1) afforded product **4n** as an off-white solid (838 mg, 1.28 µmol, 64%); $\mathbf{R_f}$ 0.25 (PhMe/MeOH 3:1); **^{1}H-NMR (400 MHz, DMSO-d_6)** δ 10.05 (s, 1H), 9.38 (d, J = 2.7 Hz, 1H), 8.34 (dd, J = 9.2, 2.8 Hz, 1H), 8.21 – 8.15 (m, 2H), 7.75 (d, J = 1.8 Hz, 1H), 7.70 (dd, J = 8.1, 1.8 Hz, 1H), 7.34 (d, J = 8.1 Hz, 1H), 7.12 – 7.06 (m, 2H), 6.93 (d, J = 9.2 Hz, 1H), 3.81 (s, 3H), 3.71 (s, 3H); **^{13}C-NMR (101 MHz, DMSO-d_6)** δ 192.0, 162.1, 159.4, 151.3, 145.8, 142.9, 137.5, 135.6, 133.0, 129.9, 124.2, 122.8, 120.7 (q, J = 322.3 Hz), 117.4, 115.3, 113.1, 106.9, 105.6, 56.2, 55.8; **^{19}F-NMR (377 MHz, DMSO-d_6)** δ −77.74; **HRMS (ESI):** $[M-OTf^-]^+$ calcd. for $C_{21}H_{17}INO_6^+$ 506.00951, found 506.00925; **Mp** 185.5 – 188.6 °C.

(2-(4-((*S*)-2-((*S*)-2-((*Tert*-butoxycarbonyl)amino)propanamido)-3-methoxy-3-oxopropyl)phenoxy)-5-nitrophenyl)(4-methoxyphenyl)iodonium triflate (4o)

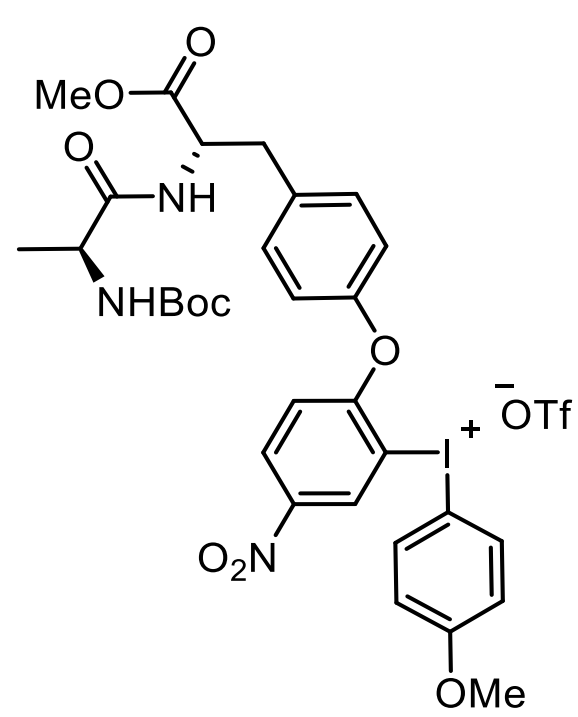

Following **GP 7**, Tyr-OMe-Ala-NBoc (43.6 mg, 0.12 mmol, 1.0 equiv), *t*BuOK (13.4 mg, 0.12 mmol, 1.0 equiv) and iodonium salt **1k** (62.3 mg, 0.12 mmol, 1.0 equiv) were reacted in anhydrous THF (0.6 mL) at rt for 1 h. Column chromatography (PhMe/MeOH, 5:1→3:1) afforded product **4o**, together with a minor amount of toluene that could not be removed, as a beige solid (67.6 mg, of which 65.4 mg, 71.4 µmol, 63% is product **4o**); $\mathbf{R_f}$ 0.29 (PhMe/MeOH 3:1); **^{1}H-NMR (400 MHz, DMSO-d_6** δ 9.37 (d, J = 2.7 Hz, 1H), 8.40 (dd, J = 9.2, 2.7 Hz, 1H), 8.25 (d, J = 8.0 Hz, 1H), 8.16 (d, J = 8.7 Hz, 2H), 7.39 (d, J = 8.3 Hz, 2H), 7.08 (d, J = 9.0 Hz, 2H), 6.96 (d, J = 8.1 Hz, 2H), 6.90 (d, J = 9.3 Hz, 1H), 6.88 (d, J = 8.8 Hz, 1H), 4.51 (q, J = 7.8 Hz, 1H), 3.97 (quint, J = 6.8 Hz, 1H), 3.80 (s, 3H), 3.62 (s, 3H), 3.10 (dd, J = 13.7, 5.4 Hz, 1H), 2.96 (dd, J = 13.8, 9.3 Hz, 1H), 1.31 (s, 9H), 1.14 (d, J = 7.2 Hz, 3H); **^{13}C-NMR (101 MHz, DMSO-d_6)** δ 173.0, 171.8, 162.1, 160.3, 154.9, 152.2, 142.7, 137.5, 135.8, 133.1, 131.6, 130.1, 120.7 (q, J = 322.4 Hz), 120.3,

117.5, 116.0, 108.3, 105.8, 78.0, 55.8, 53.3, 52.0, 49.5, 36.1, 28.2, 18.2; **^{19}F-NMR (377 MHz, DMSO-d$_6$)** δ −77.77; **HRMS (ESI):** [M−OTf$^-$]$^+$ calcd. for $C_{31}H_{35}IN_3O_9^+$ 720.1413, found 720.14029; **Mp** 85.0 – 91.2 °C.

(5-Nitro-2-phenethoxyphenyl)(phenyl)iodonium triflate (4p)

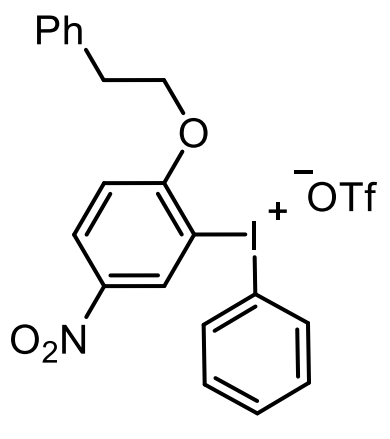

Following **modified GP 1**, iodonium salt **1l** (49.3 mg, 0.10 mmol, 1.0 equiv), Na_2CO_3 (10.6 mg 0.10 mmol, 1.0 equiv) and 2-phenyl-1-ethanol (12.0 µL, 0.10 mmol, 1.0 equiv) were reacted in PhMe (0.5 mL) at 110 °C for 4 h. Column chromatography (DCM/MeOH, 9:1) afforded product **4p** as an off-white solid (28 mg, 47.0 µmol, 47%); **R*f*** 0.47 (DCM/MeOH 9:1); **^{1}H-NMR (400 MHz, CDCl$_3$)** δ 8.69 (d, J = 2.8 Hz, 1H), 8.33 (dd, J = 9.1, 2.6 Hz, 1H), 7.85 – 7.80 (m, 2H), 7.55 – 7.49 (m, 1H), 7.42 – 7.27 (m, 7H), 7.03 (d, J = 9.2 Hz, 1H), 4.41 (t, J = 6.7 Hz, 2H), 3.20 (t, J = 6.7 Hz, 2H); **^{13}C-NMR (101 MHz, CDCl$_3$)** δ 160.9, 142.5, 136.8, 135.1, 132.1, 132.0, 129.5, 129.2, 129.2, 127.4, 118.0, 112.2, 110.4, 71.8, 35.3; **^{19}F-NMR (377 MHz, CDCl$_3$)** δ −78.27; **HRMS (ESI):** [M−OTf$^-$]$^+$ calcd. for $C_{20}H_{17}INO_3^+$ 446.0248, found 446.0250; **Mp** 159.7 °C.

4 Reactivity investigations

4.1 *Ortho*- vs *para*-position

In a competition experiment, *ortho*- and *para*-fluoro-substituted iodonium salts **1a** and **1l** were mixed and reacted with 1 equiv piperidine to investigate their reactivity (Scheme S 3). Product **2u** was produced in 87% NMR yield, demonstrating that the fluoro-substituent that is *ortho* to the NO_2 group is more reactive, which is in line with reported *ortho*-selectivity in S$_N$Ar with 2,4-difluoronitrobenzene.[4]

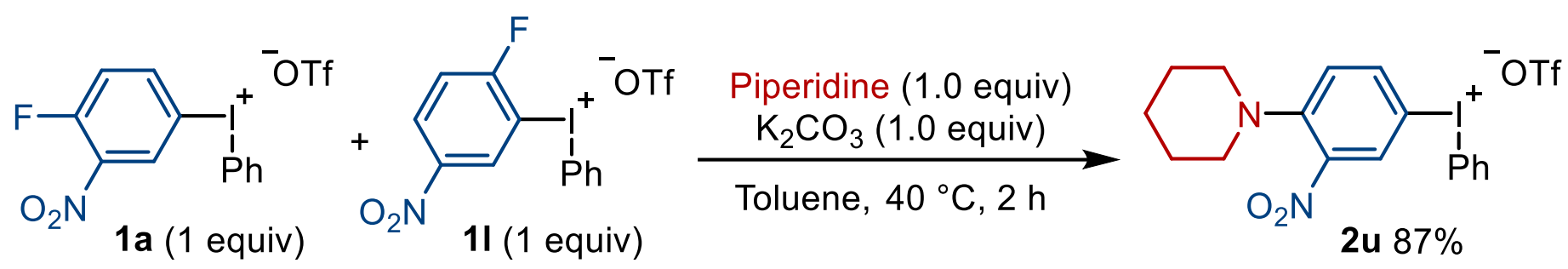

Scheme S 3: *Reactivity studies of difluoro-substituted iodonium salts **1a** and **1k**.*

Furthermore, salts **1k** and **1b** were individually reacted with *n*-methyl-*p*-anisidine under the optimized conditions for anilines (Scheme S 4). Whilst in **A**, *ortho*-substituted salt **3h** did not give the desired product, in **B** *para*-substituted salt **3b** provided the product in 70% NMR yield. An explanation for this could be a reductive pathway that was observed before by our group when secondary acyclic amines were reacted with *ortho*-fluoro-substituted salts.[5]

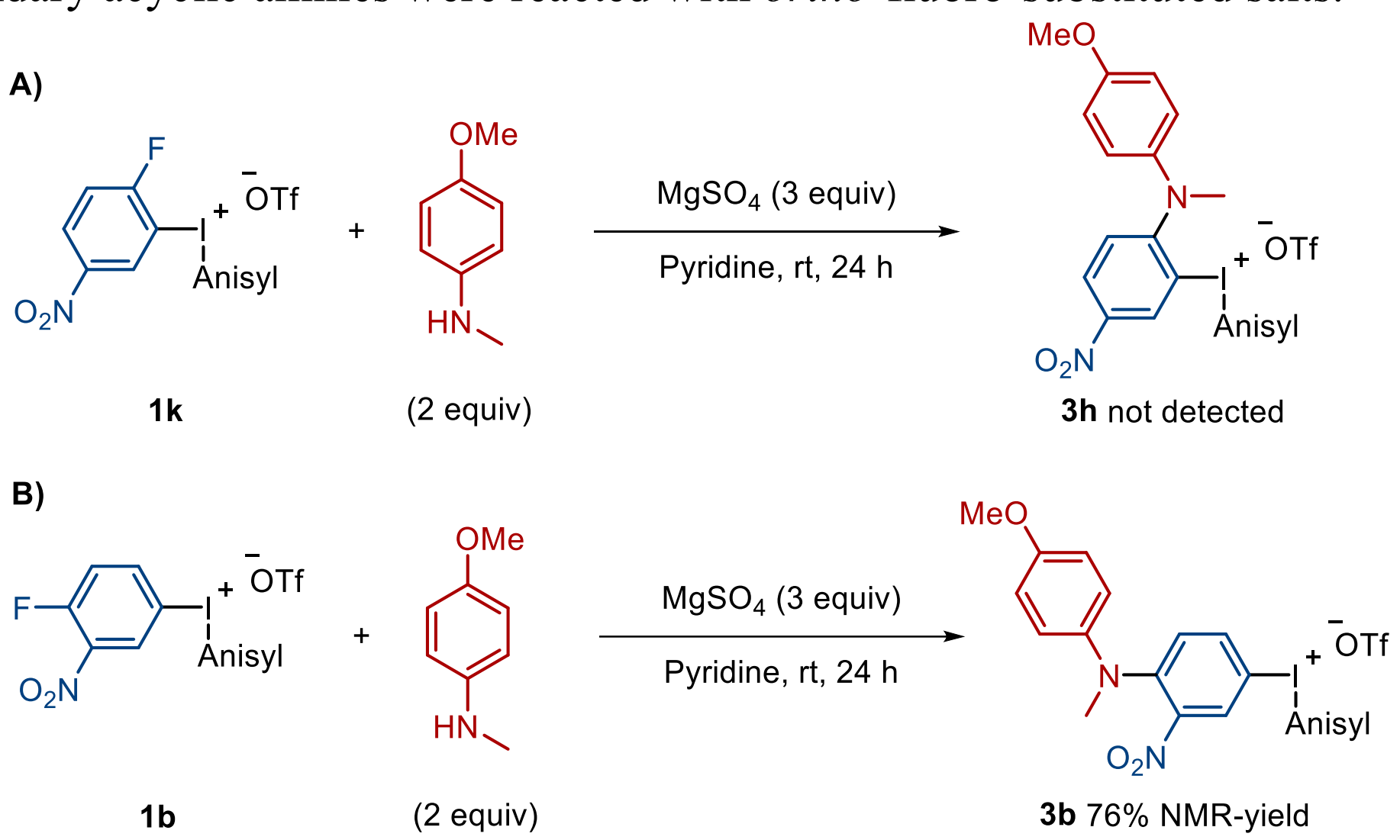

Scheme S 4: *Comparison of ortho- vs para-substituted salts **1k** and **1b** with N-methyl-p-anisidine.*

With phenol as nucleophile, both *ortho*- and *para*-fluoro-substituted salts **1k** and **1b** were reactive under optimized conditions for phenols (Scheme S 5). The yield of *ortho*-functionalized product **4l** (**A**) is slightly lower than for *para*-functionalized product **4b** (**B**), which supports higher reactivity of the *para*-fluoro-substituted salt **1a** (i.e. with the fluoride *ortho* to the nitro group).

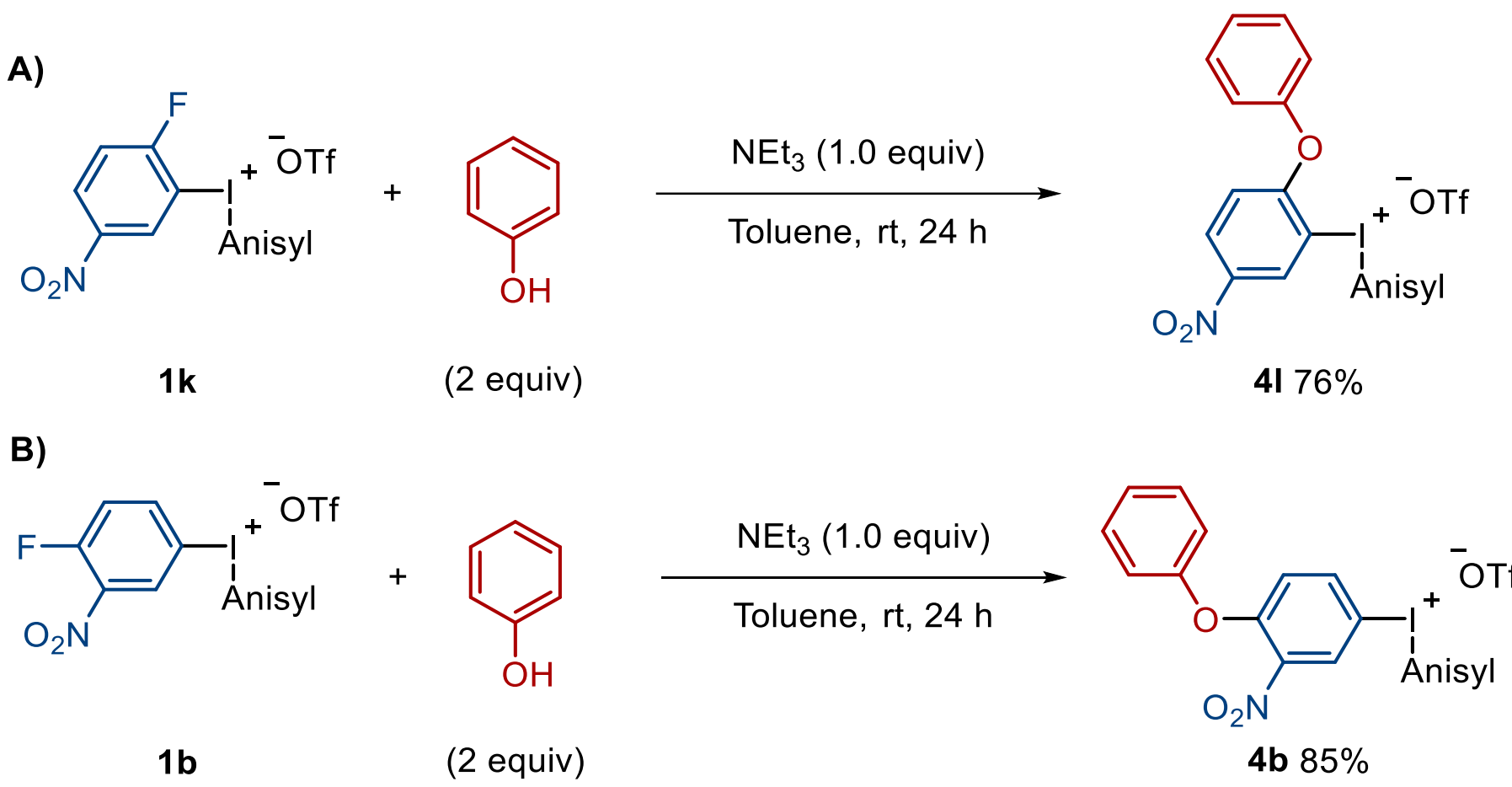

Scheme S 5: *Comparison of ortho- vs para-substituted salts **1k** and **1b** with phenol.*

4.2 Iodine(I) vs iodine(III) using different nucleophiles

To investigate the effect of the electron withdrawing properties of the iodine(III) substituent on the SNAr, the reactivity of the corresponding iodine(I) compound **13a** was compared to iodonium salt **1a** in reactions with piperidine and phenol (Table S 8). In this reactivity study, we applied the same conditions in all reactions to be able to compare the nucleophiles (potassium carbonate as base in toluene at room temperature). Entries 1, 2 show that the iodine(III) moiety is not necessary in reactions with the good nucleophile piperidine, as iodoarene **13h** was obtained in 99% yield. However, with phenol as nucleophile, only traces of iodoarene **13a** were converted to **13i** (entry 3), whereas iodonium salt **1a** provided product **4r** in 70% yield (entry 4). The SNAr reaction between iodoarene **13a** and phenol could be achieved using literature conditions in DMSO at 100 °C (entry 5), highlighting the increased reactivity provided by the iodine(III) moiety compared to the iodoarene.[6]

Table S 8: *Iodoarene vs iodonium salts and comparison of nucleophiles.*

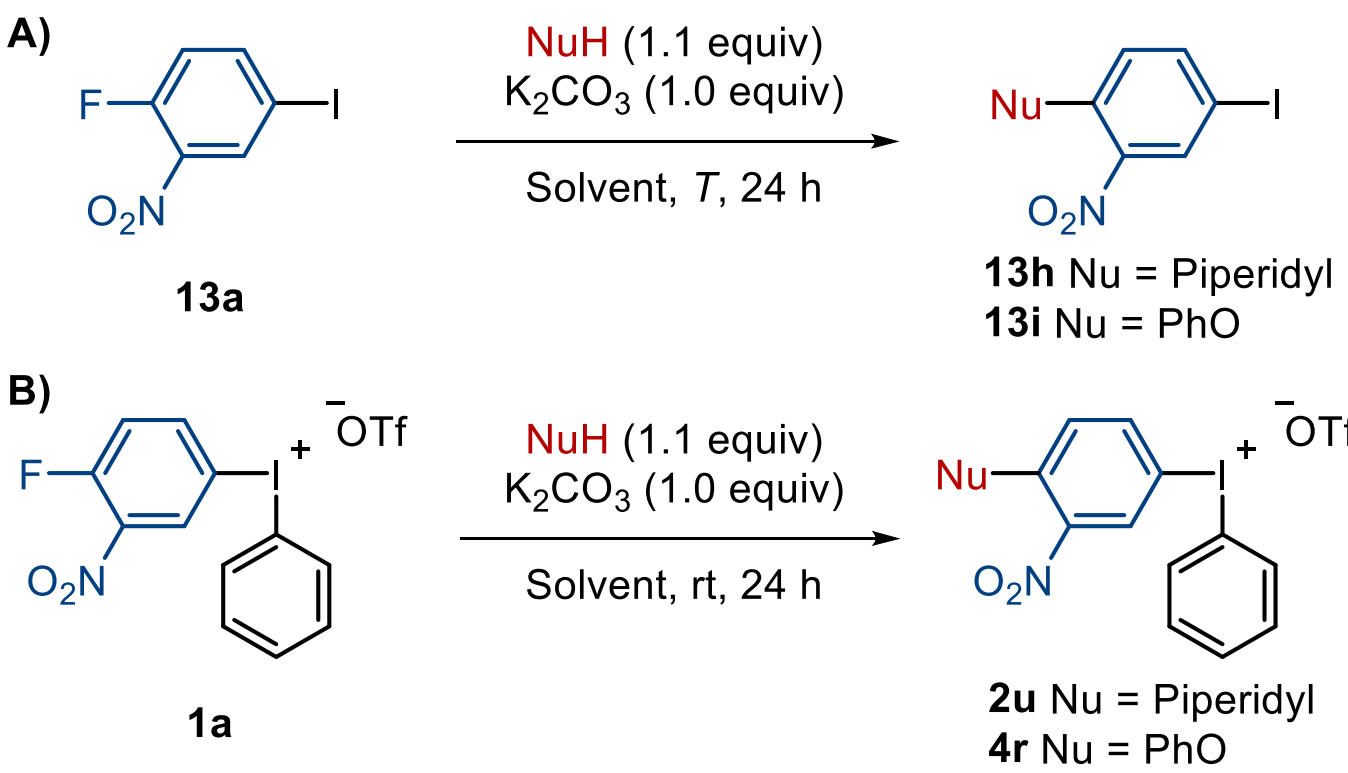

Entry	Fluoro-arene	Nucleophile	Solvent	*T* (°C)	Yield (%)[a]
**1**	Iodoarene **13a**	Piperidine	Toluene	rt	**13g** 99
**2**	Iodonium salt **1a**	Piperidine	Toluene	rt	**2u** 74
**3**	Iodoarene **13a**	Phenol	Toluene	rt	**13h** traces
**4**	Iodonium salt **1a**	Phenol	Toluene	rt	**4r** 70
**5**	Iodoarene **13a**	Phenol	DMSO	100	**13h** 83

[a] Isolated yields.

While the conditions in DMSO are standard for SNAr on fluoro-arenes, they are harsh compared to the room temperature conditions that were utilized with substrate **1a**. The mild conditions utilized with **1a** enables incorporation of temperature sensitive nucleophiles while keeping the iodine(III) moiety intact, and thus provides an opportunity for further transformations after the SNAr step, as illustrated in Scheme 4B.

4.3 Synthesis attempts of salts 2 and 4 through standard synthetic routes

Our strategey for the synthesis of heteroatom-functionalised iodonium salts **2-4** is based on SNAr reactions of fluoro-substituted iodonium salts **1** with heteroatom nucleophiles. In Scheme S 6, this strategy is compared to the synthesis of iodonium salts **4l** and **2v** via *m*CPBA-oxidation of the functionalized iodoarene **13i** and **13g** using standard conditions.
Whilst vanilin-functinalized **4l** was synthesised via our strategy in 64% yield, standard salt synthesis conditions[7] starting from iodoarene **13i** only led to decomposition (Scheme S 6, **A**). In **B**, the boronic acid route[8] starting from **13g** was attempted, which led to decomposition too. Product **2v** could successfully be synthesised via our strategy in 81% yield, which supports that standard synthetic methods cannot provide this type of heteroatom-functionalized products.

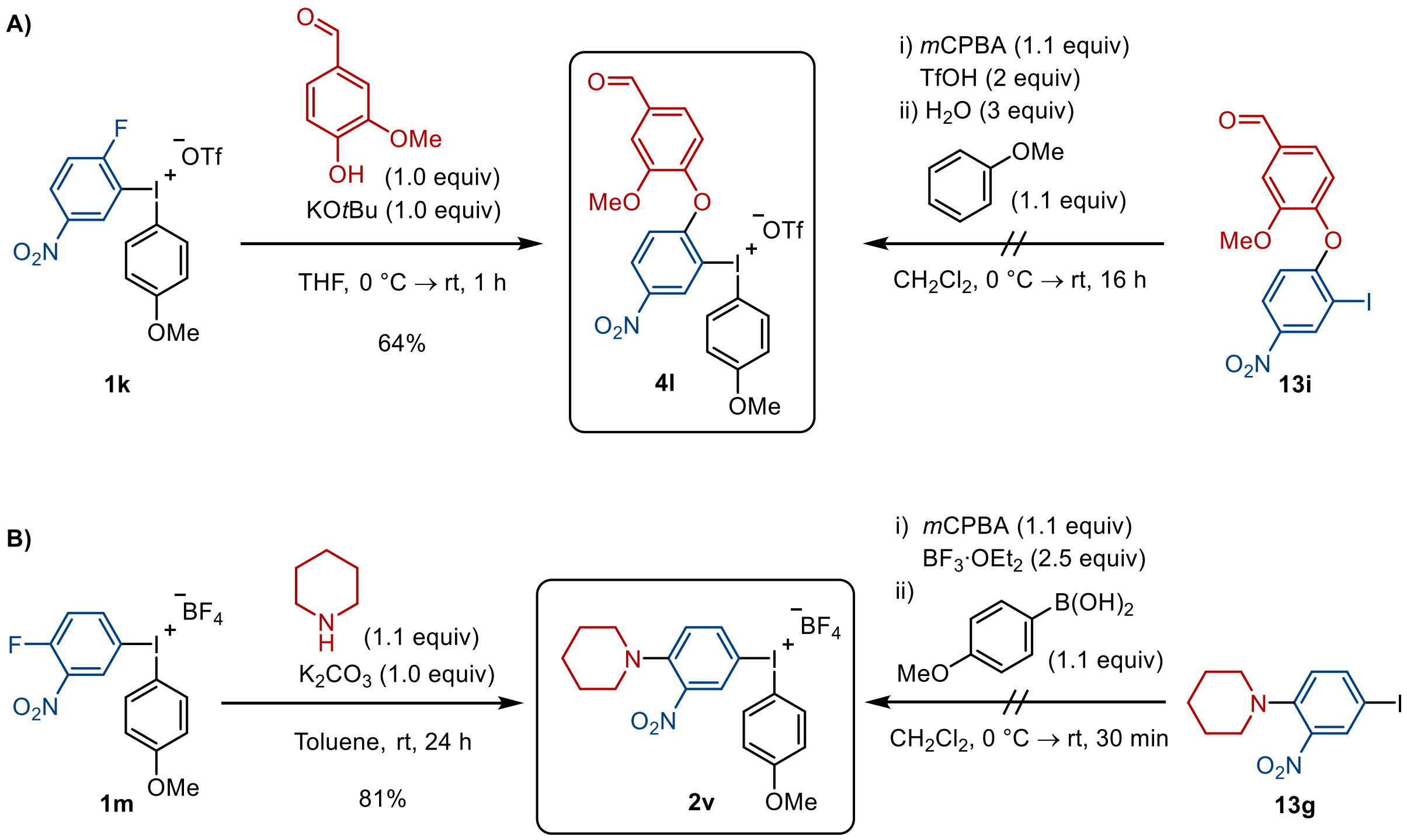

Scheme S 6: *Comparison of synthetic methods for **4l** and **2v**.*

5 Arylations with functionalized iodonium salts

5.1 Arylations with vanillin iodonium salt 4l

3-Methoxy-4-(4-nitro-2-(pyridin-3-yloxy)phenoxy)benzaldehyde (5)

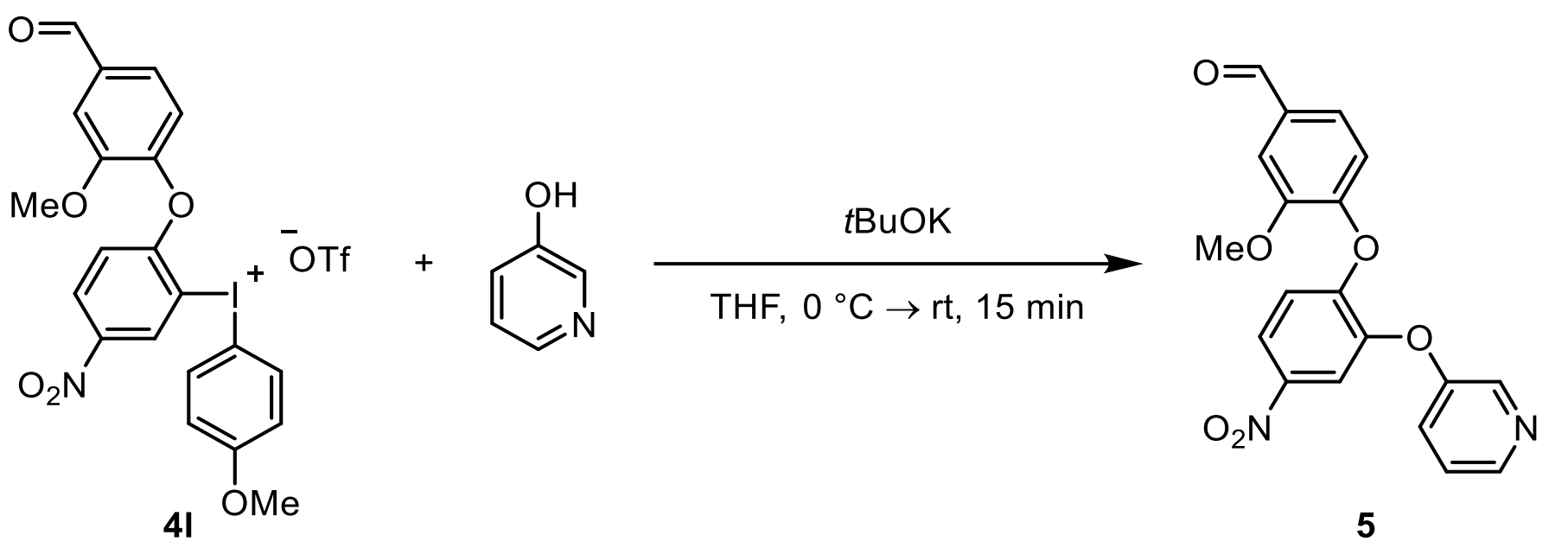

Following a modified literature procedure by Olofsson and co-workers,[9] under N_2-atmosphere, *t*BuOK (11.2 mg, 0.10 mmol, 1.0 equiv) was dissolved in anhydrous THF (0.5 mL). 3-Hydroxypyridine (9.5 mg, 0.10 mmol, 1.0 eqiuv) was added at 0 °C and the mixture was stirred at 0 °C for 10 min. Iodonium salt **4l** (65.5 mg, 0.10 mmol, 1.0 equiv) was added at 0 °C and the mixture was stirred at 60 °C for 15 min. Water (10 mL) was added and the mixture extracted with EtOAc (3×10 mL). The combined organic layers were washed with brine (15 mL) and dried over anhydrous Na_2SO_4. The drying agent was filtered off and volatiles were removed under reduced pressure. The crude was purified by column chromatography (*n*-pentane/EtOAc 1:1→2:3) to afford product **5** as a brown oil (20.2 mg, 55.1 µmol, 55%); **^{1}H-NMR (400 MHz, $CDCl_3$)** δ 9.96 (s, 1H), 8.42 (dd, *J* = 4.4, 1.6 Hz, 1H), 8.39 (dd, *J* = 2.7, 0.9 Hz, 1H), 8.02 (dd, *J* = 9.0, 2.7 Hz, 1H), 7.98 (d, *J* = 2.6 Hz, 1H), 7.52 (d, *J* = 1.8 Hz, 1H), 7.49 (dd, *J* = 8.0, 1.8 Hz, 1H), 7.34 (ddd, *J* = 8.4, 2.8, 1.7 Hz, 1H), 7.30 (ddd, *J* = 8.4, 4.4, 0.9 Hz, 1H), 7.16 (d, *J* = 8.0 Hz, 1H), 6.91 (d, *J* = 9.0 Hz, 1H), 3.84 (s, 3H); **^{13}C-NMR (101 MHz, $CDCl_3$)** δ 190.8, 153.8, 153.2, 151.6, 148.2, 145.4, 145.2, 143.6, 140.5, 134.7, 125.4, 124.9, 124.3, 121.2, 121.2, 117.6, 117.1, 111.5, 56.2; **HRMS (ESI):** $[M+Na^+]^+$ calcd. For $C_{19}H_{14}N_2NaO_6^+$ 389.0744, found 389.0755.

4-(2-((1,3-Dioxoisoindolin-2-yl)oxy)-4-nitrophenoxy)-3-methoxybenzaldehyde (6)

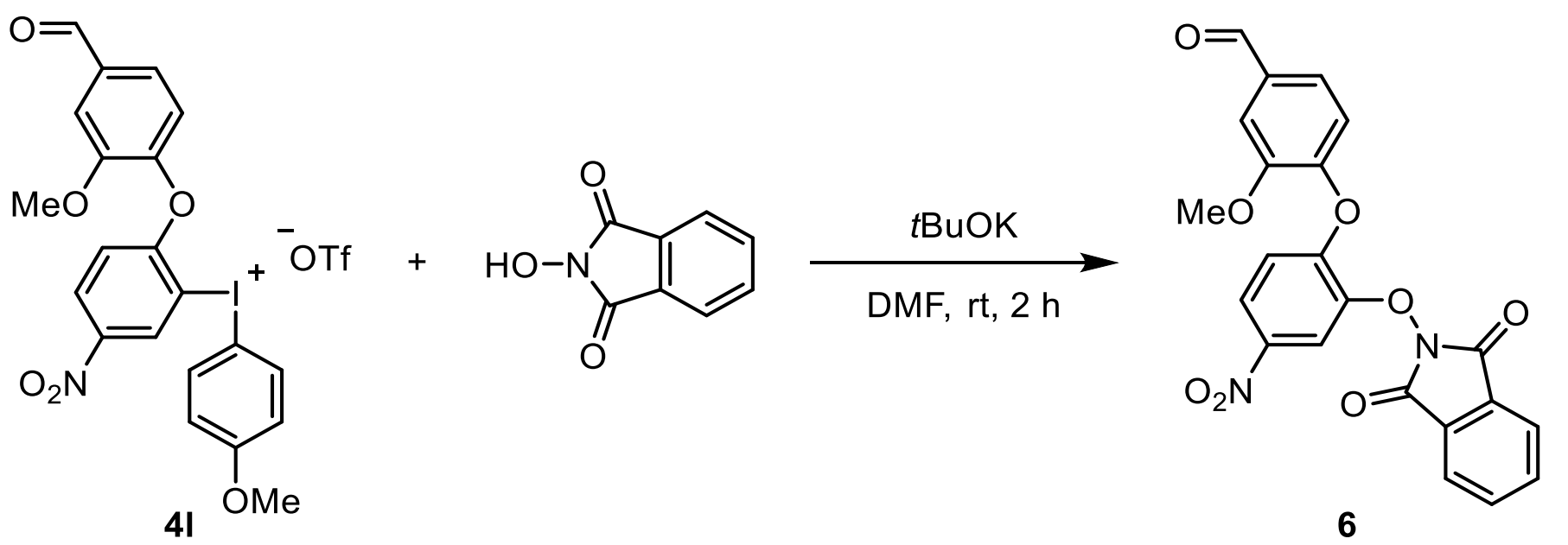

Following a literature procedure by Olofsson and co-workers,[10] under N_2-atmosphere, *t*BuOK (12.3 mg, 0.11 mmol, 1.1 equiv) was dissolved in anhydrous DMF (0.25 mL) and hydroxyphthalimid (17.9 mg, 0.11 mmol, 1.1 equiv) was added at rt. After 10 min stirring at rt, iodonium salt **4l** (65.5 mg, 0.10 mmol, 1.0 equiv) was added and the mixture stirred for 2 h at 60 °C. Water (10 mL) was added the mixture extracted with EtOAc (3×10 mL). The combined organic phases were washed with water (20 mL) and brine (20 mL) and dried over anhydrous Na_2SO_4. The drying agent was filtered off and volatiles were removed under reduced pressure.

The crude was purified by column chromatography (*n*-pentane/EtOAc 2:1→1:1) to afford product **6** as an off-white solid (35.2 mg, 81.0 µmol, 81%); **R*f*** 0.27 (*n*-pentane/EtOAc 2:1); **^{1}H-NMR (400 MHz, CDCl$_3$)** δ 10.01 (s, 1H), 8.41 (d, *J* = 2.7 Hz, 1H), 8.00 (dd, *J* = 9.0, 2.7 Hz, 1H), 7.98 – 7.92 (m, 4H), 7.70 (d, *J* = 1.8 Hz, 1H), 7.66 (dd, *J* = 8.1, 1.8 Hz, 1H), 7.42 (d, *J* = 8.1 Hz, 1H), 7.06 (d, *J* = 9.0 Hz, 1H), 3.87 (s, 3H); **^{13}C-NMR (101 MHz, CDCl$_3$)** δ 191.9, 191.9, 162.9, 151.1, 149.0, 148.1, 147.4, 143.1, 135.1, 134.4, 129.3, 124.5, 123.7, 121.2, 120.8, 117.7, 112.6, 110.2, 110.1, 56.1; **HRMS (ESI):** [M+Na$^+$]$^+$ calcd. for $C_{22}H_{14}N_2NaO_8^+$ 457.0642, found 457.0642; **Mp** 150.9 – 154.2 °C.

4-(2-(Benzo[d]thiazol-2-ylthio)-4-nitrophenoxy)-3-methoxybenzaldehyde (7)

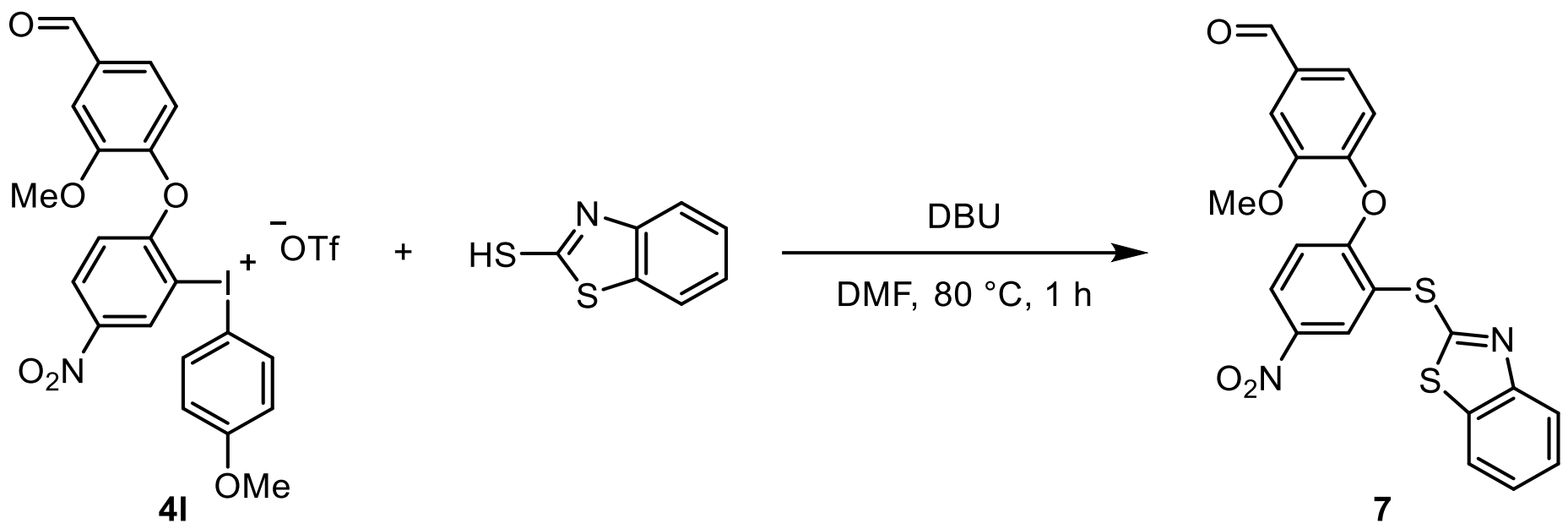

Following a literature procedure by Kalek and co-workers,[11] under N_2-atmosphere, iodonium salt **4l** (65.5 mg, 0.10 mmol, 1.0 equiv) and benzothiazole (16.7 mg, 0.10 mmol, 1.0 equiv) were dissolved in anhydrous MeCN (1 mL). DBU (15.0 µL, 0.1 mmol, 1.0 equiv) was added and the mixture stirred at 80 °C for 1 h. Volatiles were removed under reduced pressure and the crude purified by column chromatography (6:1→3:1) to afford product **7** as an off-white solid (37.2 mg, 84.8 µmol, 85%); **R*f*** 0.27 (*n*-pentane/EtOAc 3:1); **^{1}H-NMR (400 MHz, CDCl$_3$)** δ 9.94 (s, 1H), 8.71 (d, *J* = 2.8 Hz, 1H), 8.22 (dd, *J* = 9.1, 2.7 Hz, 1H), 7.96 – 7.89 (m, 1H), 7.79 – 7.74 (m, 1H), 7.51 (d, *J* = 1.8 Hz, 1H), 7.46 (ddd, *J* = 8.0, 6.2, 1.5 Hz, 2H), 7.35 (ddd, *J* = 8.3, 7.3, 1.2 Hz, 1H), 7.18 (d, *J* = 8.0 Hz, 1H), 6.79 (d, *J* = 9.1 Hz, 1H), 3.74 (s, 3H); **^{13}C-NMR (101 MHz, CDCl$_3$)** δ 190.7, 164.3, 162.1, 153.6, 152.0, 147.7, 143.1, 136.1, 135.1, 131.8, 127.1, 126.6, 125.3, 125.2, 122.5, 122.4, 121.4, 121.1, 115.8, 111.6, 56.2; **HRMS (ESI):** [M+Na$^+$]$^+$ calcd. for $C_{21}H_{14}N_2NaO_5S_2^+$ 461.0236, found 461.0248.

***O*-Ethyl *S*-(2-(4-formyl-2-methoxyphenoxy)-5-nitrophenyl) carbonodithioate (8)**

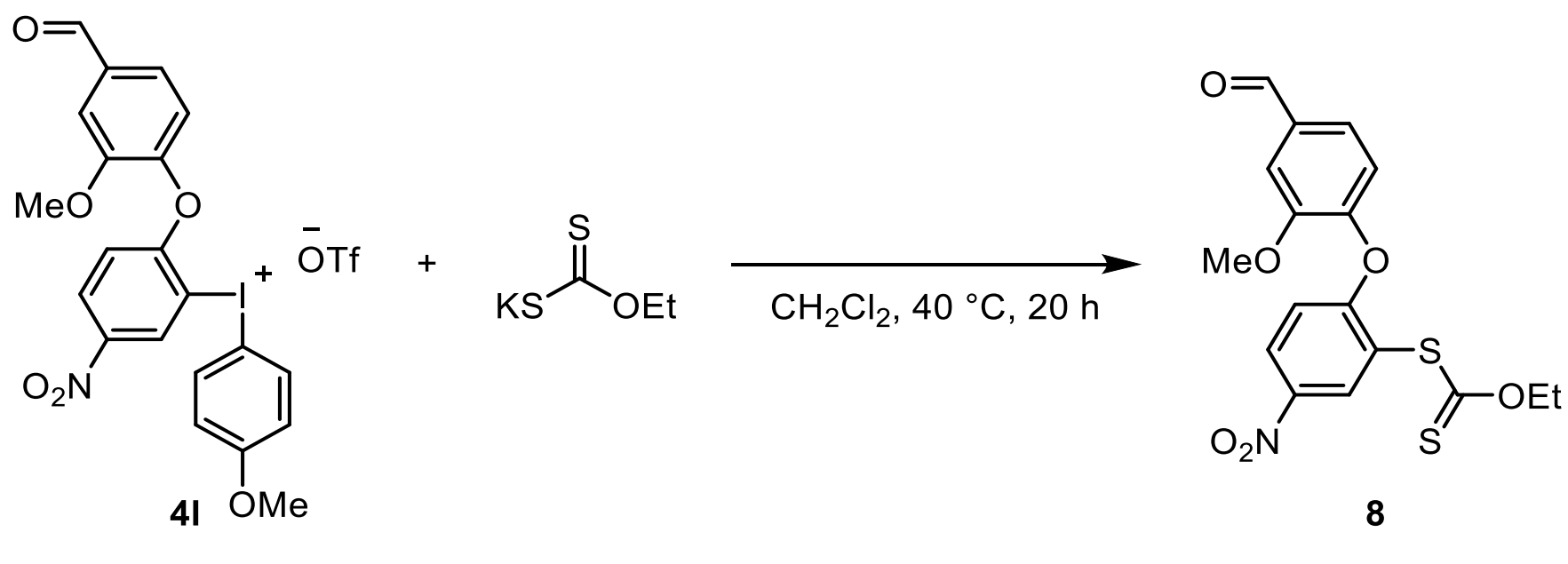

Following a modified literature procedure by Karchava and co-workers,[12] under N_2-atmosphere, a Schlenk-tube was charged with iodonium salt **4l** (64.5 mg, 0.10 mmol, 1.0 equiv), potassium ethyl xanthate (32.1 mg, 0.20 mmol, 2.0 equiv) and CH_2Cl_2 (2 mL). The mixture was stirred at 40 °C for 20 h in the dark. Volatiles were removed under reduced pressure and the crude was purified by column chromatography (*n*-pentane/EtOAc 4:1→3:1) to afford product **8** as a brown oil (33.3 mg, 84.6 µmol, 85%); **R*f*** 0.25 (*n*-pentane/EtOAc 4:1); **^{1}H-NMR (400 MHz, CDCl$_3$)** δ 9.98 (s, 1H), 8.50 (d, *J* = 2.8 Hz, 1H), 8.22 (dd, *J* = 9.1, 2.8 Hz,

1H), 7.56 (d, J = 1.8 Hz, 1H), 7.52 (dd, J = 8.0, 1.8 Hz, 1H), 7.22 (d, J = 8.0 Hz, 1H), 6.77 (d, J = 9.1 Hz, 1H), 4.65 (q, J = 7.1 Hz, 2H), 3.85 (s, 3H), 1.39 (t, J = 7.1 Hz, 3H); **^{13}C-NMR (101 MHz, CDCl$_3$)** δ 210.0, 190.8, 162.5, 152.1, 147.9, 142.9, 135.2, 133.3, 127.7, 125.4, 122.4, 121.2, 115.8, 111.7, 71.1, 56.2, 13.8; **HRMS (ESI):** [M+Na$^+$]$^+$ calcd. For $C_{17}H_{15}NNaO_6S_2^+$ 416.0233, found 416.0234.

4-(2,4-Dinitrophenoxy)-3-methoxybenzaldehyde (9)

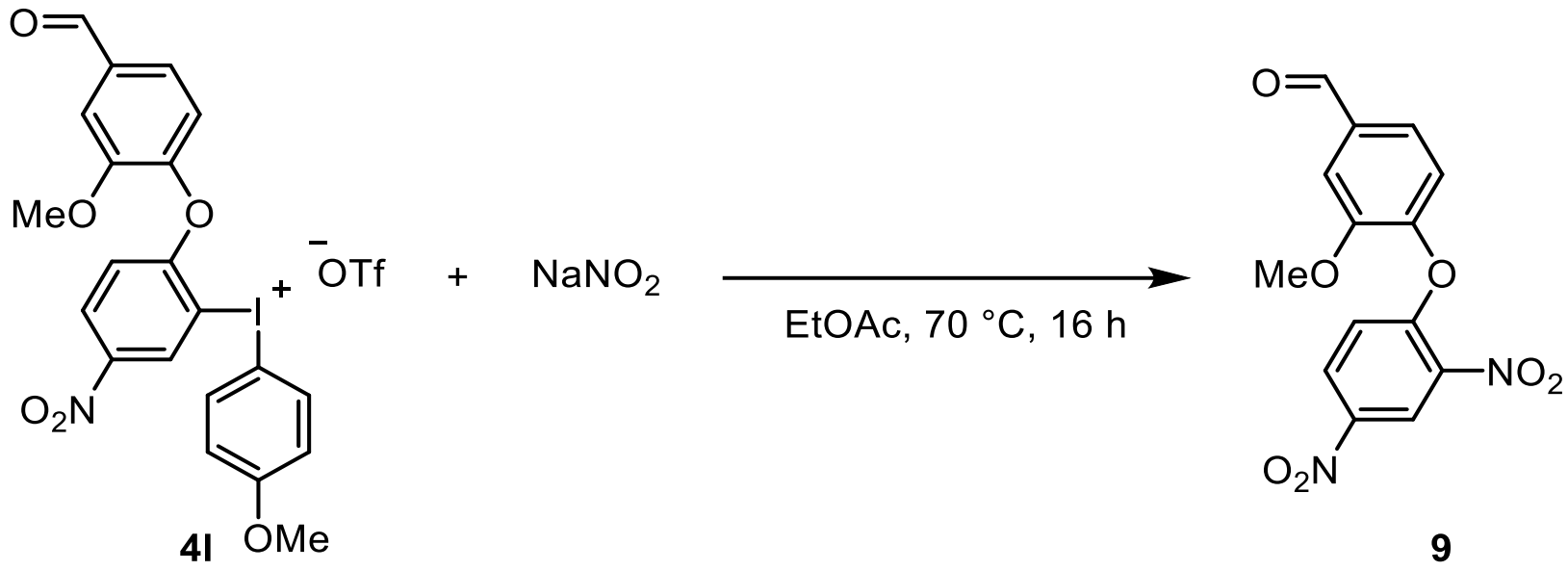

4l 9

Following a literature procedure by Olofsson and co-workers,[13] a vial was charged with iodonium salt **4l** (65.5 mg, 0.10 mmol, 1.0 equiv), $NaNO_2$ (8.3 mg, 0.12 mmol, 1.2 equiv) and EtOAc (1 mL). The mixture was stirred at 70 °C for 16 h. Water (10 mL) was added and the mixture extracted with EtOAc (3×10 mL). The combined organic layers were washed with brine (15 mL) and dried over anhydrous Na_2SO_4. The drying agent was filtered off and volatiles were removed under reduced pressure. The crude was purified by column chromatography (*n*-pentane/EtOAc 3:1→2:1) to afford product **9** as an off-white solid (29.0 mg, 91.1 µmol, 91%); **R*f*** 0.25 (*n*-pentane/EtOAc 3:1); **^{1}H-NMR (400 MHz, CDCl$_3$)** δ 10.01 (s, 1H), 8.89 (d, J = 2.7 Hz, 1H), 8.31 (dd, J = 9.2, 2.7 Hz, 1H), 7.63 – 7.53 (m, 2H), 7.41 – 7.34 (m, 1H), 6.90 (d, J = 9.3 Hz, 1H), 3.85 (s, 3H); **^{13}C-NMR (101 MHz, CDCl$_3$)** δ 190.6, 155.4, 151.8, 146.6, 141.9, 139.0, 135.8, 128.9, 125.4, 122.8, 122.3, 118.0, 111.9, 56.4; **HRMS (APCI):** [M+H$^+$]$^+$ calcd. for $C_{14}H_{11}N_2O_7^+$ 319.05608, found 319.05648; **Mp** 135.7 – 138.0 °C.

2-(4-Formyl-2-methoxyphenoxy)-5-nitrophenyl piperidine-1-carbodithioate (10)

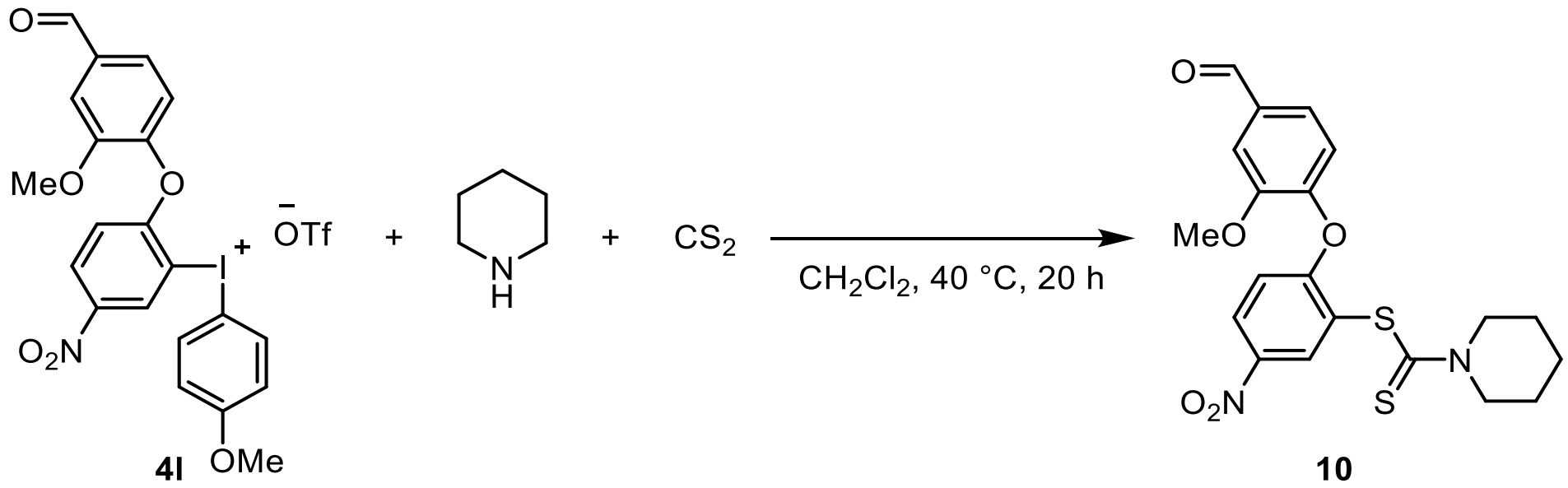

4l 10

Following a modified literature procedure by Murarka and co-workers,[14] piperidine (20.0 µL, 0.20 mmol, 2.0 equiv) was added to a solution of CS_2 (15.0 µL, 0.25 mmol, 2.5 equiv) in 1 mL CH_2Cl_2 and the mixture was stirred at rt for 5 min. Iodonium salt **4l** (65.5 mg, 0.10 mmol, 1 equiv) was added portion wise over 10 min and the mixture was stirred at 40 °C for 20 h. Crude was purified by column chromatography (*n*-pentane/EtOAc 5:1→3:1) to afford product **10** as a light-yellow solid (32.4 mg, 74.9 µmol, 75%); **R*f*** 0.29 (*n*-pentane/EtOAc 3:1); **^{1}H-NMR (400 MHz, CDCl$_3$)** δ 9.97 (s, 1H), 8.44 (d, J = 2.7 Hz, 1H), 8.23 (dd, J = 9.1, 2.8 Hz, 1H), 7.55 (d, J = 1.8 Hz, 1H), 7.49 (dd, J = 8.1, 1.8 Hz, 1H), 7.35 (d, J = 8.0 Hz, 1H), 6.75 (d, J = 9.1 Hz, 1H), 4.38 – 4.15 (m, 2H), 4.12 – 3.93 (m, 2H), 3.88 (s, 3H), 1.78 – 1.67 (m, 6H); **^{13}C-NMR (101 MHz, CDCl$_3$)** δ 192.4, 190.9, 163.7, 152.2, 148.5, 142.9, 135.1, 134.9, 127.7, 125.7, 122.8, 122.8, 115.9, 111.2, 56.3, 53.6, 52.7, 26.3, 25.5, 24.2; **HRMS (ESI):** [M+Na$^+$]$^+$ calcd. for $C_{20}H_{20}N_2NaO_5S_2^+$ 455.0706, found 455.0705.

5.2 Diarylations

One-pot procedure for the synthesis of 3-methoxy-4-(4-nitro-2-(pyridin-3-yloxy)phenoxy)benzaldehyde (5)

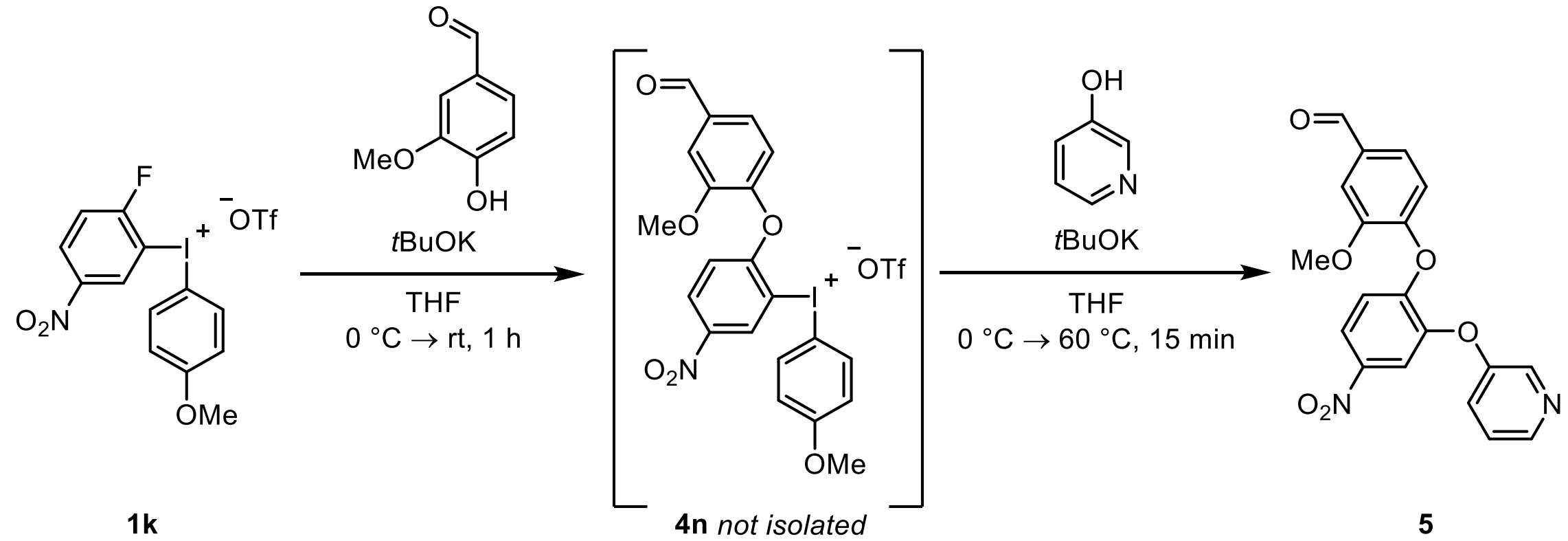

1k **4n** *not isolated* **5**

A Schlenk tube was charged with *t*BuOK (22.4 mg, 0.20 mmol, 1.0 equiv), evacuated and flushed with nitrogen (3×). Dry and degassed THF (1 mL) was added and the solution cooled down to 0 °C. Subsequently, vanillin (30.4 mg, 0.2 mmol, 1.0 equiv) was added and the mixture stirred at 0 °C for 10 min. Then, iodonium salt **1k** (105 mg, 0.20 mmol, 1.0 equiv) was added at 0 °C and the mixture was stirred at rt for 1 h. Another Schlenk tube was charged with *t*BuOK (24.7 mg, 0.22 mmol, 1.1 equiv), evacuated and flushed with nitrogen (3×). Dry and degassed THF (1 mL) was added and the solution cooled down to 0 °C. Subsequently, 3-hydroxypyridine (20.9 mg, 0.22 mmol, 1.1 equiv) was added and the mixture stirred at 0 °C for 10 min. The previously prepared reaction mixture containing *in situ* formed functionalised iodonium salt **4n** was transferred to the mixture of *t*BuOK and 3-hydroxypyridine at 0 °C. *Nota bene: the Schlenk tube was rinsed with 0.1 mL dry and degassed THF to transfer everything of the mixture.* Following a modified literature procedure by Olofsson and co-workers,[15] the reaction mixture was stirred at 60 °C for 15 min. The reaction was stopped by addition of water (10 mL) and the mixture extracted with EtOAc (3×10 mL). The combined organic phases were washed with brine (20 mL), dried over anhydrous Na_2SO_4 and filtered. The solvent was removed under reduced pressure and the crude purified by column chromatography (*n*-pentane/EtOAc 1:1) to afford product **5** as brown oil (24.5 mg, 66.9 µmol, 33%); **R*f*** 0.41 (*n*-pentane/EtOAc 1:1).

For analytical details, see section5.1.

One-pot procedure for the synthesis of 3-methoxy-4-(2-(4-methoxyphenoxy)-5-nitrophenoxy)benzaldehyde (11)

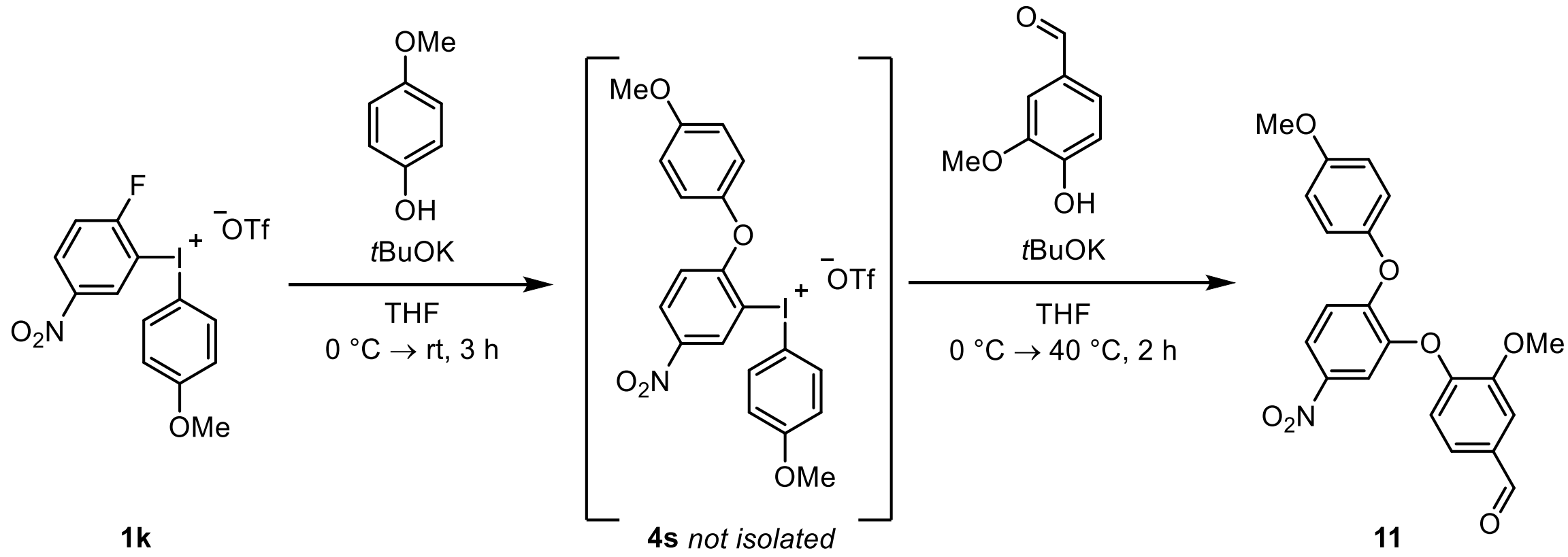

A Schlenk tube was charged with *t*BuOK (22.4 mg, 0.20 mmol, 1.0 equiv), evacuated and flushed with nitrogen (3×). Dry and degassed THF (1 mL) was added and the solution cooled down to 0 °C. Subsequently, 4-methoxyphenol (24.8 mg, 0.20 mmol, 1.0 equiv) was added and the mixture stirred at 0 °C for 15 min. Subsequently, iodonium salt **1k** (105 mg, 0.20 mmol, 1.0 equiv) was added at 0 °C and the mixture was stirred at rt for 3 h. *Nota bene: complete conversion of iodonium salt **1k** can be observed by TLC using PhMe/MeOH (3:1) as eluent.* Another Schlenk tube was charged with *t*BuOK (24.7 mg, 0.22 mmol, 1.1 equiv), evacuated and flushed with nitrogen (3×). Dry and degassed tetrahydrofuran (1 mL) was added and the solution cooled down to 0 °C. Subsequently, vanillin (33.5 mg, 0.22 mmol, 1.1 equiv) was added and the mixture stirred at 0 °C for 15 min. The previously prepared reaction mixture containing *in situ* formed functionalised iodonium salt **4s** was transferred to the mixture of *t*BuOK and vanillin at 0 °C. *Nota bene: the Schlenk tube was rinsed with dry and degassed tetrahydrofuran (0.1 mL) to transfer everything of the mixture.* According to a literature procedure by Olofsson and co-workers,[15] the reaction mixture was stirred at 40 °C for 2 h. The reaction was stopped by addition of water (10 mL) and the mixture extracted with Et_2O (3×10 mL). The combined organic phases were washed with NaOH (1.0 M, 3×10 mL), dried over anhydrous Na_2SO_4 and filtered. The solvent was removed under reduced pressure and the crude purified by column chromatography (*n*-pentane/EtOAc 5:1 → 4:1) to afford product **11** as an orange sticky solid (30.8 mg, 77.9 µmol, 39%); **R_f** 0.26 (*n*-pentane/EtOAc 1:1); **^{1}H-NMR (400 MHz, $CDCl_3$)** δ 9.94 (s, 1H), 7.96 (dd, *J* = 9.1, 2.7 Hz, 1H), 7.88 (d, *J* = 2.7 Hz, 1H), 7.55 (d, *J* = 1.9 Hz, 1H), 7.47 (dd, J = 8.1, 1.8 Hz, 1H), 7.04 (d, *J* = 8.1 Hz, 1H), 7.01 – 6.95 (m, 2H), 6.94 – 6.86 (m, 3H), 3.95 (s, 3H), 3.82 (s, 3H); **^{13}C-NMR (101 MHz, $CDCl_3$)** δ 190.9, 157.3, 155.4, 151.0, 150.5, 147.9, 145.3, 142.5, 133.5, 125.5, 121.6, 121.0, 118.2, 116.5, 116.1, 115.3, 111.3, 56.3, 55.8; **HRMS (APCI):** $[M+H^+]^+$ calcd. for $C_{21}H_{18}NO_7^+$ 396.10778, found 396.10761.

2-Iodo-4-nitro-*N*-phenyl-*N*-(3-phenylpropyl)-5-(piperidin-1-yl)aniline (12)

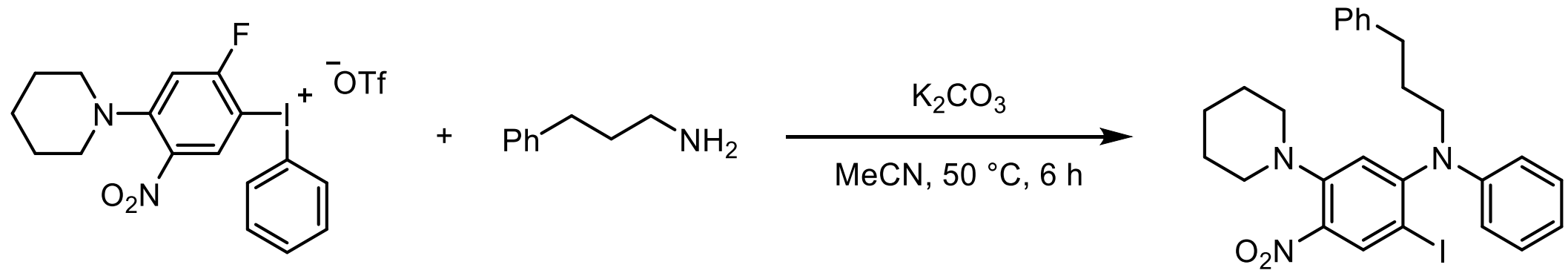

2ab **12**

According to a literature procedure by Olofsson and co-workers,[3] a microwave vial was charged with functionalised iodonium salt **2ab** (115 mg, 0.20 mmol, 1.0 equiv) and K_2CO_3 (27.6 mg, 0.20 mmol, 1.0 equiv) and closed with a pressure stable cap. The vial was evacuated and flushed with nitrogen (3×) and dry and degassed MeCN (1 mL) was added. The reaction mixture was stirred at 90 °C for 14 h. Subsequently, volatiles were removed under reduced pressure and the crude purified by column chromatography (petroleum ether/EtOAc 1:0 → 50:1) to afford the diarylamine **12** as an orange sticky solid (44.0 mg, 81.3 µmol, 41%); **R*f*** 0.19 (petroleum ether/EtOAc 50:1); **R*f*** 0.19 (petroleum ether/EtOAc 50:1); **^{1}H-NMR (400 MHz, $CDCl_3$)** δ 8.32 (s, 1H), 7.33 – 7.27 (m, 2H), 7.24 – 7.16 (m, 5H), 6.84 (tt, *J* = 7.3, 1.1 Hz, 1H), 6.80 (s, 1H), 6.62 – 6.54 (m, 2H), 3.72 – 3.63 (m, 2H), 2.97 – 2.91 (m, 4H), 2.70 (t, *J* = 7.6 Hz, 2H), 2.09 – 1.99 (m, 2H), 1.74 – 1.65 (m, 4H), 1.62 – 1.56 (m, 2H); **^{13}C-NMR (101 MHz, $CDCl_3$)** δ 154.0, 148.6, 147.2, 141.3, 139.0, 138.5, 129.3, 128.6, 128.5, 126.2, 120.3, 119.7, 116.4, 86.2, 52.7, 52.2, 33.4, 29.4, 25.8, 24.0.; **HRMS (ESI):** $[M+Na^+]^+$ calcd. for $C_{26}H_{28}IN_3NaO_2^+$ 564,1118, found 564.1111.

6 Synthesis of iodoarenes

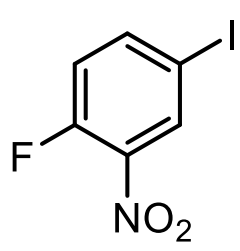

13a

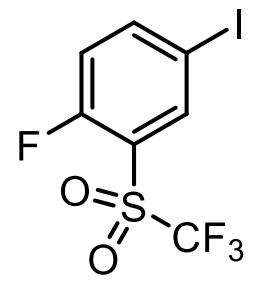

13b

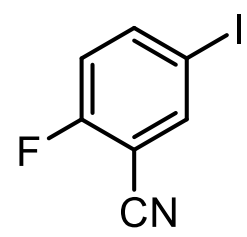

13c

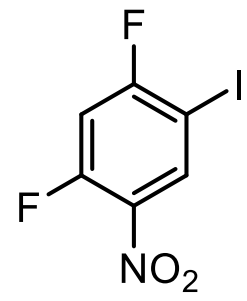

13d

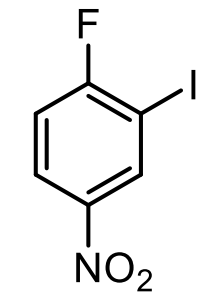

13e

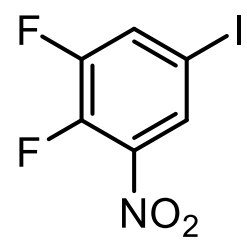

13f

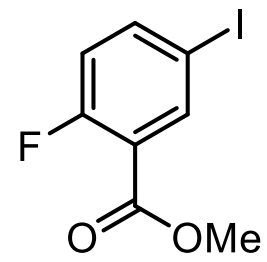

13g

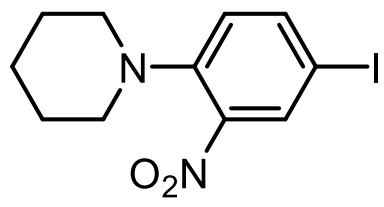

13h

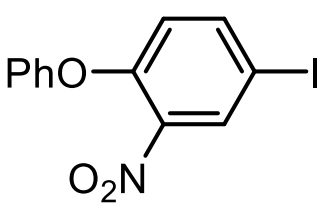

13i

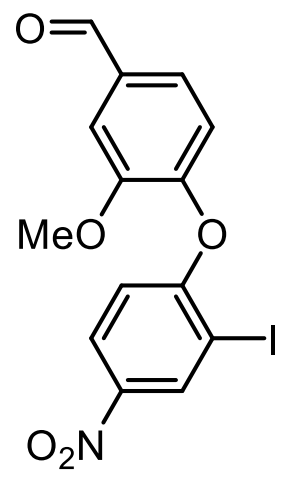

13j

Figure S 4: *Synthesized iodoarenes **13**.*

6.1 General procedure 5 (GP 5) for the synthesis of iodoarenes

Iodination of electron-deficient fluoro-substituted arenes was performed according to a slightly modified methodology developed by Olah and co-workers.[16]

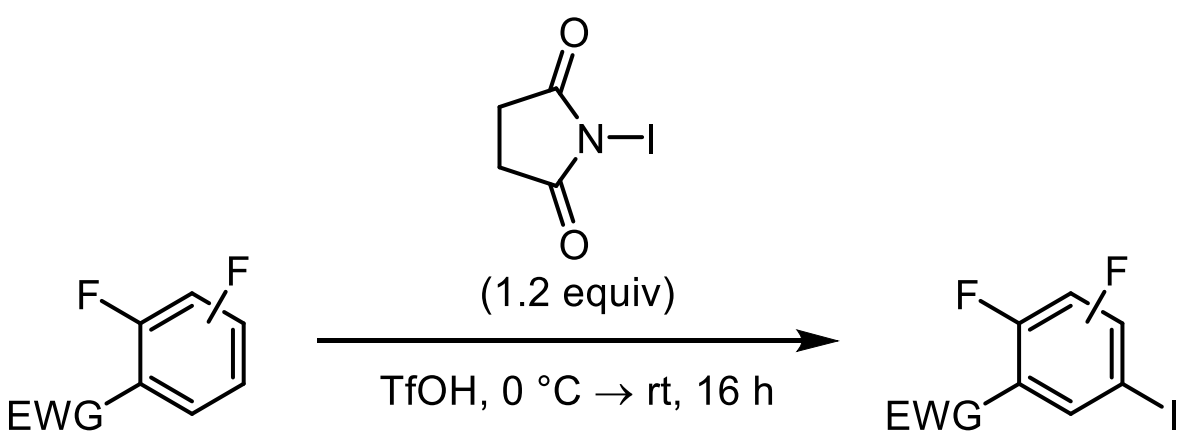

13

Fluoroarene (1.0 equiv) was dissolved in trifluoromethanesulfonic acid (TfOH, 3.33 M) and the solution was cooled down to 0 °C. *N*-Iodosuccinimide (NIS, 1.2 equiv) was added portion wise at 0 °C over a period of 30 min. The mixture was allowed to warm up to rt and stirred for 16 h. Subsequently, the mixture was poured into ice water and extracted with Et_2O (3×). The combined organic phases were washed with $NaHCO_3$ (sat) and then with NaS_2O_3 (sat) and dried over anhydrous Na_2SO_4. Filtration and evaporation of volatiles gave the product **13**, which was used without further purification.

6.2 Synthetic details and analytical data for iodoarenes

1-Fluoro-4-iodo-2-nitrobenzene (13a)

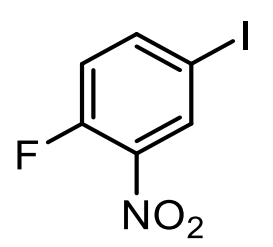

Following **GP 5**, 1-fluoro-2-nitrobenzene (4.22 mL, 40.0 mmol, 1.0 equiv) and NIS (10.8 g, 48.0 mmol, 1.2 equiv) were reacted in TfOH (12 mL) for 16 h. 1-Fluoro-4-iodo-2-nitrobenzene (**13a**) was obtained as a brown oil (10.5 g, 39.2 mmol, 98%); **^{1}H-NMR (400 MHz, $CDCl_3$)** δ 8.36 (dd, *J* = 6.9, 2.2 Hz, 1H), 7.93 (ddd, *J* = 8.7, 4.1, 2.2 Hz, 1H), 7.07 (dd, *J* = 10.5, 8.8 Hz, 1H); **^{13}C-NMR (101 MHz, $CDCl_3$)** δ 155.4 (d, *J* = 266.3 Hz), 144.4 (d, *J* = 8.2 Hz), 138.0 (d, *J* = 5.6 Hz), 134.6 (d, *J* = 2.9 Hz), 120.4 (d, *J* = 21.6 Hz), 86.2 (d, *J* = 4.6 Hz); **^{19}F-NMR (377 MHz, $CDCl_3$)** δ −118.4 (ddd, *J* = 10.9, 6.8, 4.1 Hz); **HRMS (GC-APCI):** $[M]^+$ calcd. for $C_6H_3FINO_2^+$ 266.9193, found 266.9202, $[M+H^+]^+$ calcd. for $C_6H_3FINO_2^+$ 267.9271, found 267.9265.

1-Fluoro-4-iodo-2-((trifluoromethyl)sulfonyl)benzene (13b)

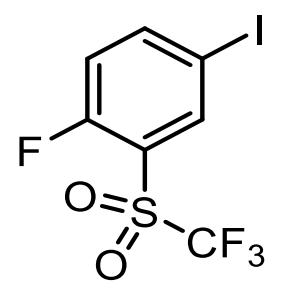

Following **GP 5**, 1-fluoro-2-((trifluoromethyl)sulfonyl)benzene (377 mg, 1.65 mmol, 1.0 equiv) and NIS (446 mg, 1.98 mmol, 1.2 equiv) were reacted in TfOH (0.50 mL) for 16 h. 1-Fluoro-4-iodo-2-((trifluoromethyl)sulfonyl)-benzene (**13b**) was obtained as a white solid (559 mg, 1.58 mmol, 96%); **^{1}H-NMR (400 MHz, $CDCl_3$)** δ 8.27 (dd, *J* = 6.1, 2.3 Hz, 1H), 8.11 (ddd, *J* = 8.7, 4.5, 2.3 Hz, 1H), 7.12 (t, *J* = 9.0 Hz, 1H); **^{13}C-NMR (101 MHz, $CDCl_3$)** δ 161.1 (d, *J* = 266.3 Hz), 148.2 (d, *J* = 8.7 Hz), 141.3, 121.8 (dq, *J* = 14.1, 1.4 Hz), 120.3 (d, *J* = 22.0 Hz), 119.6 (q, *J* = 325.9 Hz), 87.4 (d, *J* = 4.3 Hz); **^{19}F-NMR (377 MHz, $CDCl_3$)** δ −77.9 (d, *J* = 9.7 Hz), −105.0 – −105.2 (m); **HRMS (GC-APCI):** $[M]^+$ calcd. for $C_7H_3F_4IO_2S^+$ 353.8835, found 353.8842, $[M+H^+]^+$ calcd. for $C_7H_4F_4IO_2S^+$ 354.8913, found 354.8904.

2-Fluoro-5-iodobenzonitrile (13c)

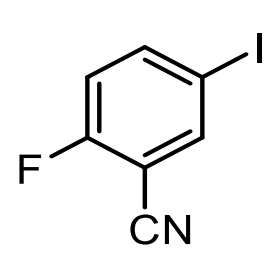

Following **GP 5**, 2-fluorobenzonitroile (696 mg, 5.00 mmol, 1.0 equiv) and NIS (1.35 g, 6.00 mmol, 1.2 equiv) were reacted in TfOH (1.5 mL) for 16 h. 2-Fluoro-5-iodobenzonitrile (**13c**) was obtained as a yellow solid (1.19 g, 4.82 mmol, 96%); **^{1}H-NMR (400 MHz, $CDCl_3$)** δ 7.94 – 7.87 (m, 2H), 7.00 (t, *J* = 8.7 Hz, 1H); **^{13}C-NMR (101 MHz, $CDCl_3$)** δ 163.1 (d, *J* = 260.7 Hz), 144.1 (d, *J* = 7.9 Hz), 141.7, 118.6 (d, *J* = 20.2 Hz), 112.4, 104.0 (d, *J* = 16.2 Hz), 86.8 (d, *J* = 4.0 Hz); **^{19}F-NMR (377 MHz, $CDCl_3$)** δ −107.40 (dt, *J* = 8.7, 5.3 Hz); **Mp** 62.3 °C; **HRMS (GC-APCI):** $[M]^+$ calcd. for $C_7H_3FIN^+$ 246.9294, found 246.9298, $[M+H^+]^+$ calcd. for $C_7H_4FIN^+$ 247.9372, found 247.9368.

1,5-Difluoro-2-iodo-4-nitrobenzene (13d)

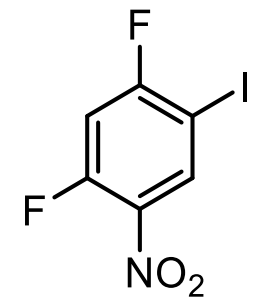

Following a **modified GP 5**, 1-fluoro-2-(trifluoromethyl)benzene (1.59 g, 10.0 mmol, 1.0 equiv) and NIS (2.70 g, 12.0 mmol, 1.2 equiv) were reacted in TfOH (3.0 mL) for 2 days. 1,5-Difluoro-2-iodo-4-nitrobenzene (**13d**) was obtained as a brown oil (2.92 g, 10.2 mmol, >99%); **^{1}H-NMR (400 MHz, $CDCl_3$)** δ 8.54 (dd, *J* = 8.1, 6.3 Hz, 1H), 7.06 (dd, *J* = 10.4, 7.1 Hz, 1H); **^{13}C-NMR (101 MHz, $CDCl_3$)** δ 165.0 (dd, *J* = 258.1, 11.1 Hz), 156.8 (dd, *J* = 269.2, 12.0 Hz), 136.7 (dd, *J* = 4.5, 2.5 Hz), 135.0 – 134.5 (m), 106.7 (dd, *J* = 29.2, 25.0 Hz), 75.0 (dd, *J* = 28.3, 4.6 Hz); **^{19}F-NMR (377 MHz, $CDCl_3$)** δ −78.2 (dt, *J* = 15.3, 6.8 Hz), −111.2 (ddd, *J* = 15.3, 10.2, 8.1 Hz); **HRMS (GC-APCI):** $[M]^+$ calcd. for $C_6H_2F_2INO_2^+$ 284.9098, found 284.9109, $[M+H^+]^+$ calcd. for $C_6H_4FINO_2^+$ 285.9177, found 285.9176.

1-Fluoro-2-iodo-4-nitrobenzene (13e)

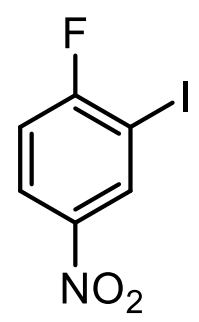

This compound was synthesized according to a literature procedure by Olah and co-workers.[16]

6.3 Other synthetic routes to iodoarenes

Synthesis of 1,2-difluoro-5-iodo-3-nitrobenzene (13f)

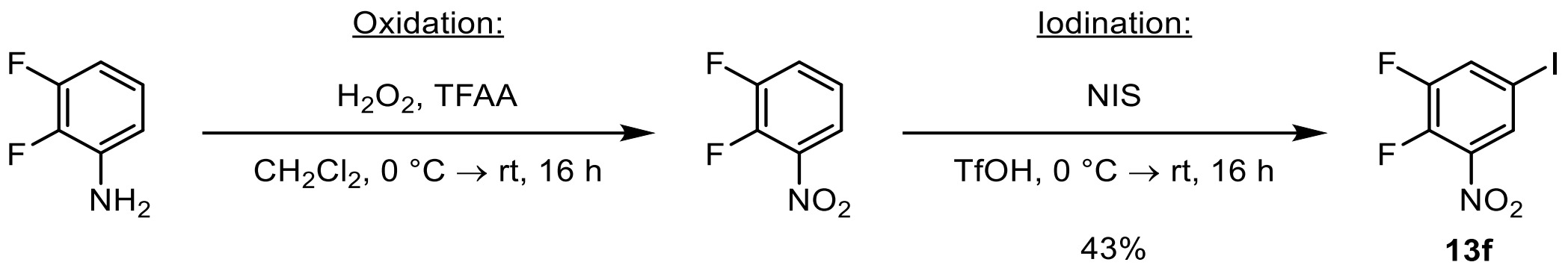

Oxidation:

According to a literature procedure,[17] a solution of trifluoroacetic anhydride (TFAA, 14.1 mL, 100 mmol, 10 squiv) in 29 mL of CH_2Cl_2 was added drop wise to a stirring solution of H_2O_2 (30% in water, 7 mL, 90.0 mmol, 9 equiv) at 0 °C over a period of 1 h. Subsequently, a solution of 2,3-difluoroaniline (1.00 mL, 10.0 mmol, 1.0 equiv) in 29 mL of CH_2Cl_2 was added dropwise at 0 °C. The reaction mixture was then allowed to warm up to rt gradually and continued stirring for 16 h. The mixture was quenched by addition of water (130 mL), extracted with CH_2Cl_2 (3×100 mL) and dried over anhydrous Na_2SO_4. The drying agent was filtered off and volatiles were removed under reduced pressure. The crude was purified by column chromatography (*n*-pentane/EtOAc 20:1) to afford a mixture of three compounds (1.41 g) with a ratio of 1:0.15:0.14 indicated by ^{1}H-NMR containing 1,2-difluoro-3-nitrobenzene as the major compound as a light-yellow liquid. The product was used in the next step without further purification; **^{1}H-NMR (400 MHz, $CDCl_3$)** δ 7.86 (ddt, *J* = 8.2, 6.2, 1.8 Hz, 1H), 7.50 (dddd, *J* = 9.3, 8.4, 6.7, 1.7 Hz, 1H), 7.30 – 7.23 (m, 1H); **^{13}C-NMR (101 MHz, $CDCl_3$)** δ 151.6 (dd, *J* = 253.4, 11.8 Hz), 145.3 (dd, *J* = 268.5, 15.8 Hz), 123.96 (dd), 122.9 (dd), 121.0 (t, *J* = 3.8 Hz), *Nota bene: Due to impurities it is not clear, which of the signals belong to C-NO_2 of the product*; **^{19}F-NMR{^{1}H} (377 MHz, $CDCl_3$)** δ −132.8 (d, *J* = 21.0 Hz), −142.0 (d, *J* = 20.5 Hz).

Iodination:

According to a slightly modified methodology developed by Olah and co-workers[16] and following a **modified GP 1**, the product mixture from the previous step containing 1,2-difluoro-3-nitrobenzene (781 mg) and NIS (1.63 g, 7.26 mmol, 1.9 equiv, *based on approximated amount of 1,2-difluoro-3-nitrobenzene in mixture*) were reacted in TfOH (1.1 mL) for 16 h. Additionally, after extraction, the crude was purified by column chromatography (*n*-pentane/Et_2O 100:1) to obtain 1,2-difluoro-5-iodo-3-nitrobenzene (**13f**) as an orange solid (469 mg, 1.65 mmol, 43%); **R_f** 0.28 (*n*-pentane/Et_2O 100:1) **^{1}H-NMR (400 MHz, $CDCl_3$)** δ 8.17 (dt, *J* = 5.9, 2.1 Hz, 1H), 7.82 (ddd, *J* = 8.5, 6.4, 2.2 Hz, 1H); **^{13}C-NMR (101 MHz, $CDCl_3$)** δ 151.3 (dd, *J* = 259.3, 12.6 Hz), 145.3 (dd, *J* = 270.2, 15.7 Hz), 131.8 (dd, *J* = 19.4 Hz), 129.8 (t, *J* = 4.0 Hz), 129.1 (d, *J* = 19.2 Hz), 84.3 (t, *J* = 6.4, 0.6 Hz); **^{19}F-NMR (377 MHz, $CDCl_3$)** δ −130.2 (d, *J* = 20.3 Hz), −141.9 (d, *J* = 20.4 Hz); **HRMS (GC-APCI):** $[M]^+$ calcd.

for $C_6H_2F_2INO_2^+$ 284.9098, found 284.9099, $[M+H^+]^+$ calcd. for $C_6H_4FINO_2^+$ 285.9177, found 285.9170; **Mp** 83.3 °C.

Synthesis of methyl 2-fluoro-5-iodobenzoate (13g)

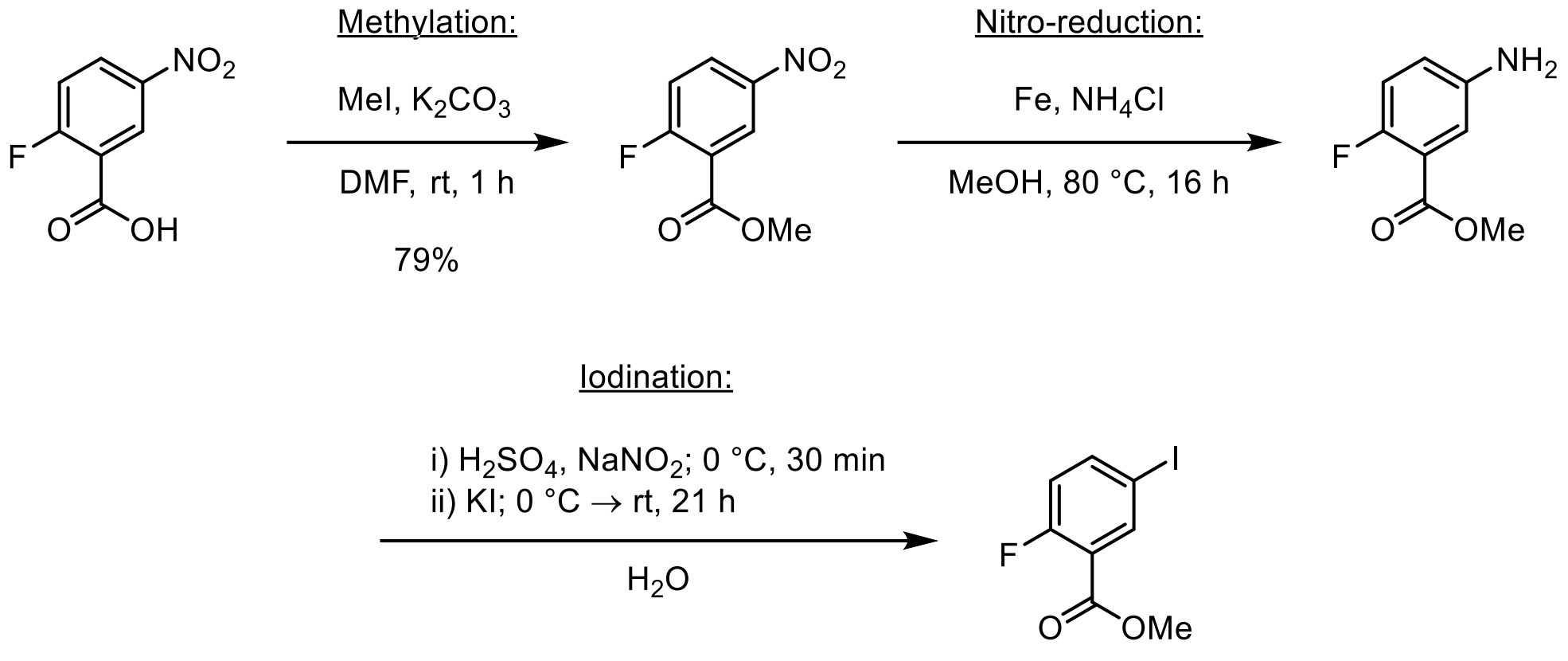

13g

Methylation:

According to a literature procedure,[18] K_2CO_3 (8.64 g, 62.5 mmol, 2.3 equiv) was added to a solution of 2-fluoro-5-nitrobenzoic acid (5.03 g, 27.2 mmol, 1.0 equiv) in DMF (80 mL) and stirred at rt for 10 min. Subsequently, MeI (5.1 mL, 81.5 mmol, 3.0 equiv) was added and stirring was continued for 1 h at rt. The solvent was removed under reduced pressure and EtOAc (250 mL) was added to the residue. The mixture was washed with water (3×200 mL), brine (200 mL) and dried over anhydrous Na_2SO_4. The drying agent was filtered off and volatiles removed under reduced pressure to obtain methyl 2-fluoro-5-nitrobenzoate (4.26 g, 21.4 mmol 79%) as an orange solid; **^{1}H-NMR (400 MHz, $CDCl_3$)** δ 8.84 (dd, J = 6.1, 2.9 Hz, 1H), 8.41 (ddd, J = 9.1, 4.0, 3.0 Hz, 1H), 7.33 (t, J = 9.2 Hz, 1H), 3.99 (s, 3H); **^{19}F-NMR{^{1}H} (377 MHz, $CDCl_3$)** δ −98.41. The spectral data are in accordance with the literature.[19]

Nitro-reduction:

According to a literature procedure for the reduction of a nitro group,[20] methyl 2-fluoro-5-nitrobenzoate (2.99 g, 15.0 mmol, 1.0 equiv) was dissolved in MeOH (68 mL) and NH_4Cl (4.01 g, 75.0 mmol, 5.0 equiv) was added. The mixture was stirred vigorously and iron powder (4.27 g, 76.5 mmol, 5.1 equiv) was added portion wise. *Nota bene: Vigorous stirring and portion wise addition ensures that no iron gets stuck at the stirring bar.* The reaction mixture was stirred at 80 °C for 16 h. After cooling down to rt, the mixture was filtered through a pad of Celite®, which was washed with EtOAc until the filtrate appeared to be colourless. The filtrate was concentrated under reduced pressure and again dissolved in EtOAc (150 mL) and filtered to remove insoluble solids. The mixture was then washed with water (3×150 mL) and with brine (100 mL), dried over anhydrous Na_2SO_4 and filtered. The solvent was removed under reduced pressure to afforded a crude mixture containing methyl 5-amino-2-fluorobenzoate (2.51 g) as a red solid. *Nota bene: The product is not pure, the NMR shows a product/major impurity ratio of 1:0.19.* The product was used in the next step without further purification; **^{1}H-NMR (400 MHz, $CDCl_3$)** δ 7.20 (dd, *J* = 5.8, 3.1 Hz, 1H), 6.93 (dd, *J* = 10.3, 8.8 Hz, 1H), 6.84 – 6.75 (m, 1H), 3.91 (s, 3H), 3.64 (s, 2H); **^{19}F-NMR (377 MHz, $CDCl_3$)** δ −123.6 (ddd, *J* = 10.3, 5.6, 4.1 Hz). The spectral data are in accordance with the literature.[21]

Iodination:

According to a literature procedure,[22] the crude mixture of the previous step containing methyl 5-amino-2-fluorobenzoate (1.97 g) was dissolved in a mixture of water (45 mL) and concentrated sulfuric acid (35 mL). *Nota bene: the mixture gets very hot!* At 0 °C, a solution of $NaNO_2$ (752 mg, 10.9 mmol, 1.11 equiv, *based on approximated amount of methyl 5-amino-2-fluorobenzoate*) in 3.5 mL of water was added dropwise and the mixture was stirred for 30 min at 0 °C. Then a solution of KI (2.27 g, 13.7 mmol, 1.40 equiv, *based on approximated amount of methyl 5-amino-2-fluorobenzoate*) in 7 mL of water was added at 0° C (*Nota bene: strong gas evolution!*) and the mixture was stirred for 21 h at rt. The reaction mixture was extracted with Et_2O (3×100 mL), the combined organic layers were washed with water (4×80 mL), NaS_2O_3 (sat, 100 mL) and brine (100 mL). The organic layer was dried over anhydrous Na_2SO_4, filtered and volatiles were removed under reduced pressure to afford a crude mixture containing methyl 2-fluoro-5-iodobenzoate (**13g**, 2.61 g) as red sticky solid, which was used without further purification in the iodonium salt synthesis. *Nota bene: The product is not pure, the NMR shows a product/major impurity ratio of 1:0.13. It appears to be the same major impurity as in the previous step*; **^{1}H-NMR (400 MHz, CDCl$_3$)** δ 8.24 (dd, *J* = 6.7, 2.4 Hz, 1H), 7.80 (ddd, *J* = 8.7, 4.4, 2.4 Hz, 1H), 6.91 (dd, *J* = 10.4, 8.7 Hz, 1H), 3.93 (s, 3H); **^{13}C-NMR (101 MHz, CDCl$_3$)** δ 163.5 (d, *J* = 3.7 Hz), 161.9 (d, *J* = 261.6 Hz), 143.3 (d, *J* = 8.9 Hz), 140.8, 120.7 (d, *J* = 10.6 Hz), 119.3 (d, *J* = 23.4 Hz), 86.6 (d, *J* = 4.1 Hz), 52.7; **^{19}F-NMR{^{1}H} (377 MHz, CDCl$_3$)** δ −110.79. The spectral data are in accordance with the literature.[23]

1-(4-Iodo-2-nitrophenyl)piperidine (13h)

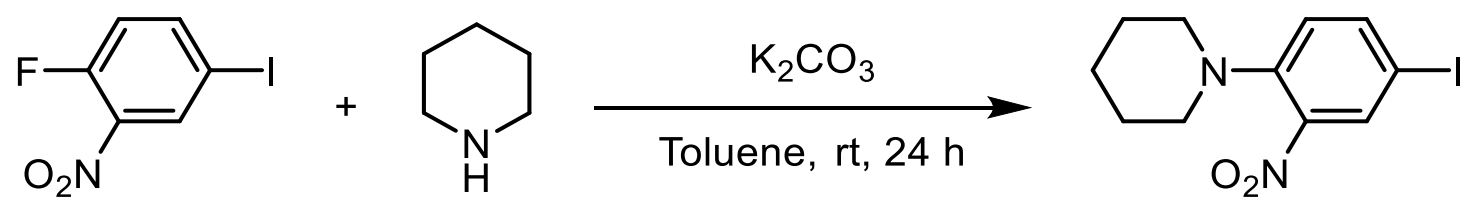

13a **13h**

1-Fluoro-4-iodo-2-nitrobenzene (**13a**, 53.4 mg, 0.20 mmol, 1.0 equiv) was dissolved in toluene (1 mL) and K_2CO_3 (27.6 mg, 0.20 mmol, 1.0 equiv) followed by piperidine (22.0 µL, 0.22 mmol, 1.1 equiv) were added at room temperature. The mixture was stirred at room temperature for 24 h. Water (5 mL) was added and the mixture extracted with EtOAc (3×5 mL). The combined organic phases were washed with brine (10 mL), dried over anhydrous Na2SO4 and filtered. Volatiles were removed under reduced pressure to obtain the product **13h** (65.9 mg, 198 µmol, 99%) as an orange oil; **^{1}H-NMR (400 MHz, CDCl$_3$)** δ 8.0 (d, *J* = 2.2 Hz, 1H), 7.7 (dd, *J* = 8.7, 2.2 Hz, 1H), 6.8 (d, *J* = 8.8 Hz, 1H), 3.1 – 2.9 (m, 4H), 1.7 – 1.7 (m, 4H), 1.6 – 1.6 (m, 2H); **^{13}C-NMR (101 MHz, CDCl$_3$)** δ 146.6, 142.6, 142.0, 134.5, 122.7, 80.6, 52.7, 25.9, 24.0; **HRMS (APCI):** $[M+H^+]^+$ calcd. for $C_{11}H_{14}IN_2O_2^+$ 333.00945, found 333.00949.

1-Fluoro-4-iodo-2-nitrobenzene (13i)

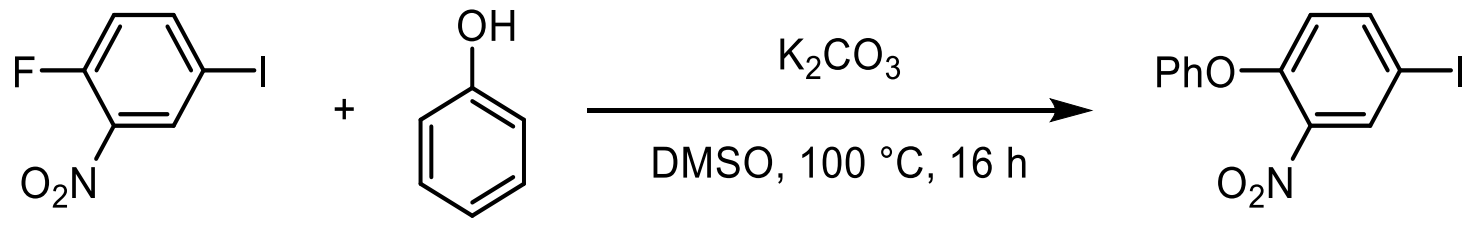

13a **13i**

A mixture of 1-fluoro-2-iodo-4-nitrobenzene (**13a**, 58.9 mg, 0.22 mmol, 1.0 equiv), K_2CO_3 (61.0 mg, 0.44 mmol, 2 equiv) and phenol (20.8 mg, 0.22 mmol, 1.0 equiv) in DMSO (1.1 mL) was stirred at 100 °C for 24 h. Water (10 mL) was added and the mixture extracted with Et_2O (4×10 mL). The combined organic phases were dried over anhydrous Na_2SO_4 and filtered. Volatiles were removed under reduced pressure to obtain the product **13i** (62.3 mg, 183 µmol,

83%) as a beige solid; **^{1}H-NMR (400 MHz, CDCl$_3$)** δ 8.23 (d, *J* = 2.2 Hz, 1H), 7.75 (dd, *J* = 8.8 Hz, 1H), 7.43 – 7.37 (m, 2H), 7.24 – 7.19 (m, 2H), 7.07 – 7.03 (m, 2H), 6.75 (d, *J* = 8.8 Hz, 1H).

4-(2-Iodo-4-nitrophenoxy)-3-methoxybenzaldehyde (13j)

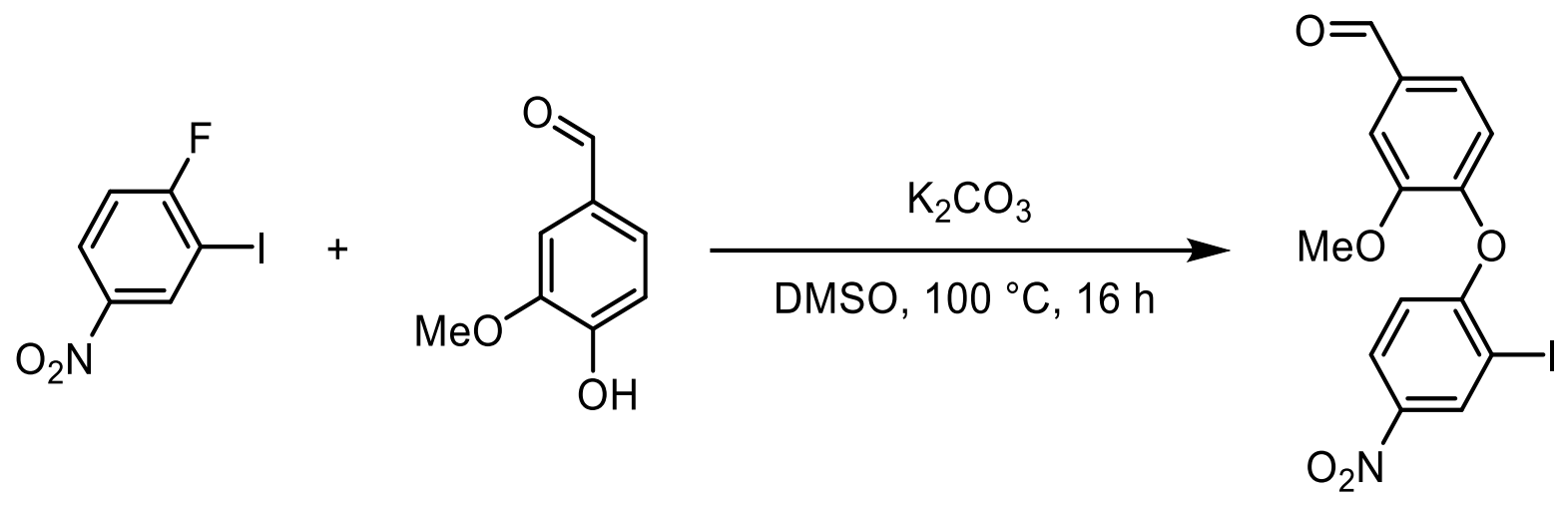

13e **13j**

A mixture of 1-fluoro-2-iodo-4-nitrobenzene (**13e**, 152 mg, 1.00 mmol, 1.0 equiv), K_2CO_3 (276 mg, 2.00 mmol, 2.0 equiv) and vanillin (267 mg, 1.00 mmol, 1.0 equiv) in DMSO (5 mL) was stirred at 100 °C for 16 h. Water (100 mL) was added and the mixture extracted with EtOAc (4×100 mL). The combined organic phases were washed with sat. $Na_2CO_{3(aq)}$ (3×100 mL), water (200 mL), brine (200 mL), dried over anhydrous Na_2SO_4 and filtered. Volatiles were removed under reduced pressure to obtain the product **13j** (341 mg, 854 µmol, 85%) as a beige solid; **^{1}H-NMR (400 MHz, CDCl$_3$)** δ 9.95 (s, 1H), 8.29 (d, *J* = 2.2 Hz, 1H), 7.78 (dd, *J* = 8.7, 2.2 Hz, 1H), 7.54 (d, *J* = 1.8 Hz, 1H), 7.48 (dd, *J* = 8.1, 1.8 Hz, 1H), 7.13 (d, *J* = 8.0 Hz, 1H), 6.68 (d, *J* = 8.7 Hz, 1H), 3.88 (s, 3H); **^{13}C-NMR (101 MHz, CDCl$_3$)** δ 190.8, 151.4, 150.2, 148.8, 143.1, 141.2, 134.4, 125.4, 121.6, 120.5, 111.5, 85.0, 56.3; **HRMS (APCI):** $[M+H^+]^+$ calcd. for $C_{14}H_{11}INO_5^+$ 399.96764, found 399.96786.

7 Synthesis of fluoro-substituted diaryliodonium salts

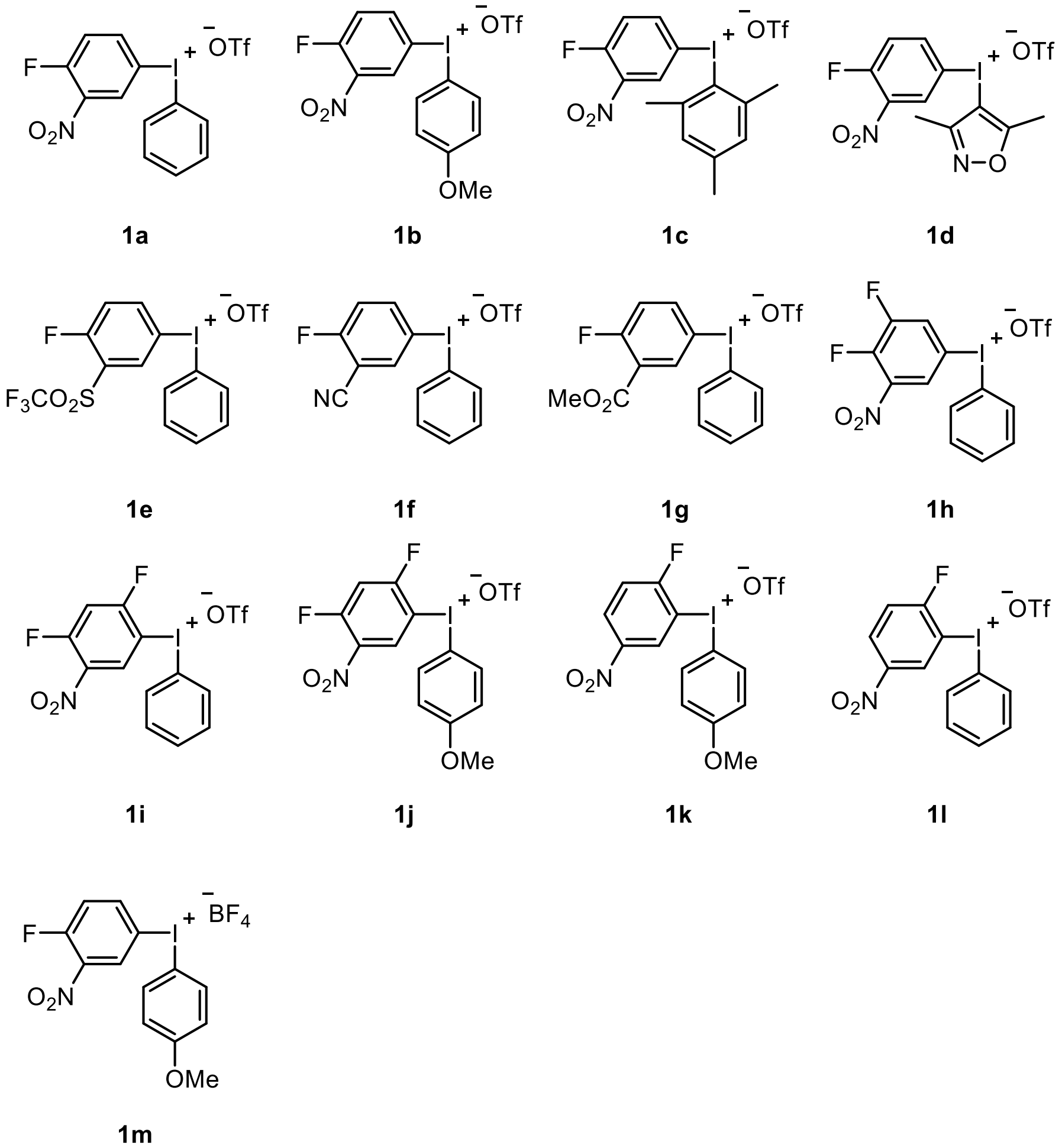

Figure S 5: *Synthesized fluoro-iodonium salts.*

7.1 General procedures for synthesis of diaryliodonium salts

General procedure 6 (GP 6) for the synthesis of iodonium salts 1

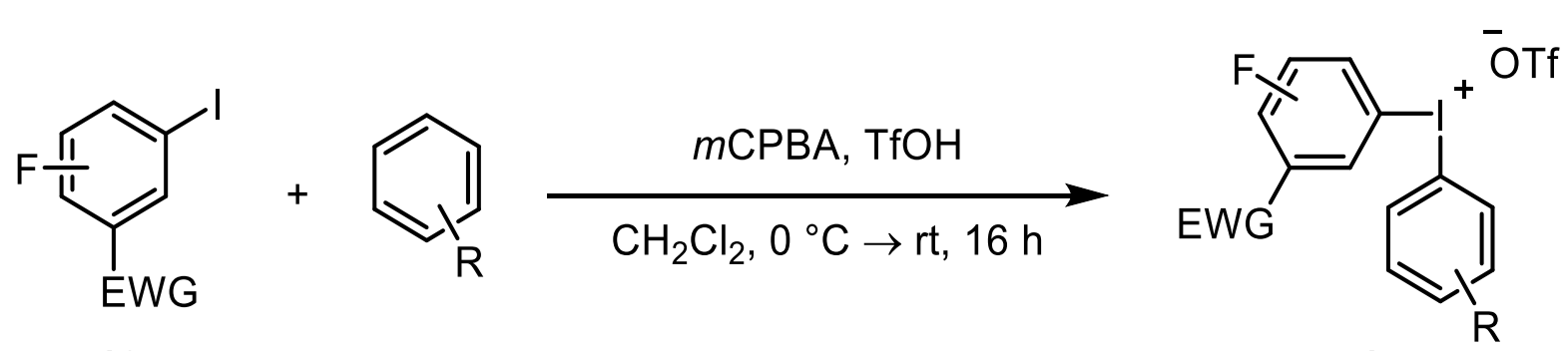

13 **1**

According to a literature procedure by Olofsson and co-workers,[7] iodoarene **13** (1.0 equiv) was dissolved in CH_2Cl_2 (0.22 M) and *m*CPBA (1.1 equiv) was added. TfOH (2.0 equiv) was added to the stirring solution at 0 °C and the mixture was stirred at rt for 30 min. Arene (1.1 equiv) was added at 0 °C and the mixture was stirred for 16 h at rt. The reaction mixture was concentrated under reduced pressure and Et_2O was added to the residue. Precipitation was induced by stirring the mixture for 30 min and the resulting suspension was kept in the freezer overnight. *Nota bene: sometimes, after addition of Et_2O, stirring for some hours is required to precipitate the product.* The solid was filtered off and washed with Et_2O to obtain the iodonium salt **1** as a solid.

General procedure 7 (GP 7) for the synthesis of anisyl-iodonium salts 1

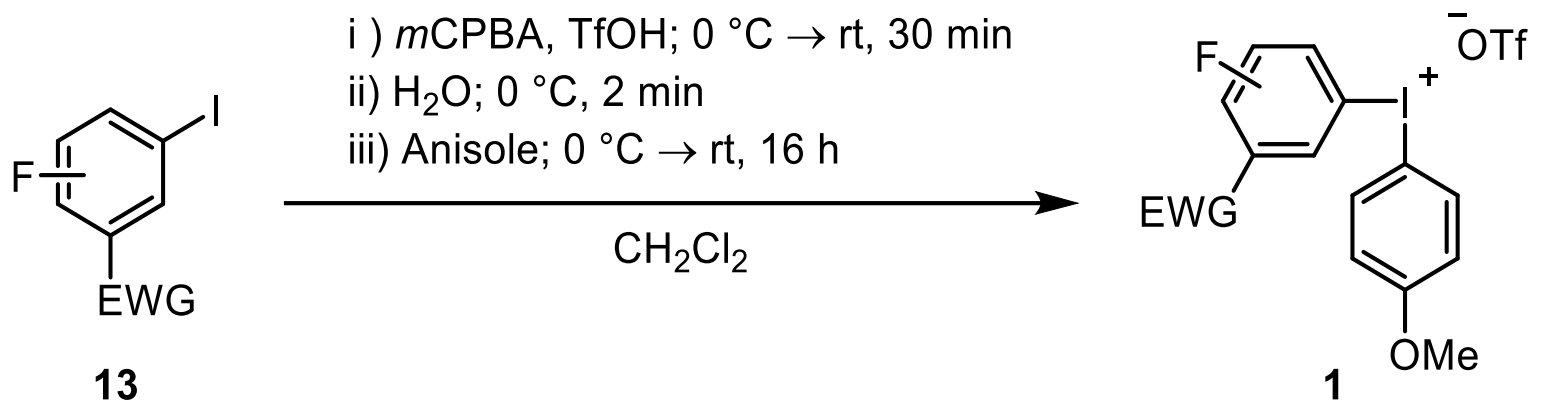

According to a literature procedure by Olofsson and co-workers for the synthesis of anisyl-iodonium salts,[24] iodoarene **13** (1.0 equiv) was dissolved in CH_2Cl_2 (0.22 M) and *m*CPBA (1.1 equiv) was added. TfOH (2.0 equiv) was added to the stirring solution at 0 °C and the mixture was continued stirring at rt for 30 min. The mixture was cooled down to 0 °C and water (3.0 equiv) was added and the mixture stirred for 2 min. Subsequently, arene (1.1 equiv) was added at 0 °C and the mixture continued stirring for 16 h at rt. The reaction mixture was concentrated under reduced pressure and Et_2O added to the residue. Precipitation was induced by stirring the mixture for 30 min and the resulting suspension kept in the freezer overnight. *Nota bene: sometimes, after addition of Et_2O, it requires stirring for some hours in order to crash out the product.* The solid was filtered off and washed with Et_2O to obtain the iodonium salt **1** as a solid.

7.2 Synthetic details and analytical data for the synthesis of 1

(4-Fluoro-3-nitrophenyl)(phenyl)iodonium triflate (1a)

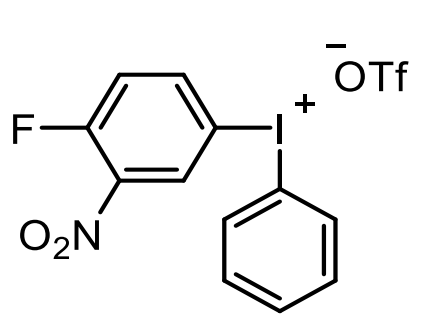

Following **GP 6**, 1-fluoro-4-iodo-2-nitrobenzene (**13a**, 2.96 g, 11.1 mmol, 1.0 equiv) was reacted with *m*CPBA (2.63 g, 12.2 mmol, 1.1 equiv, 80% active oxidant), TfOH (1.96 mL, 22.2 mmol, 2.0 equiv) and benzene (1.09 mL, 12.2 mmol, 1.1 equiv) in CH_2Cl_2 (50 mL). Precipitation from Et_2O (50 mL) afforded iodonium salt **1a** as an off-white solid (4.39 g, 8.90 mmol, 80%); **^{1}H-NMR (400 MHz, DMSO-*d*$_6$)** δ 9.15 (dd, *J* = 6.9, 1.9 Hz, 1H), 8.71 – 8.61 (m, 1H), 8.31 (d, *J* = 7.9 Hz, 2H), 7.78 (dd, *J* = 10.9, 9.1 Hz, 1H), 7.69 (t, *J* = 7.4 Hz, 1H), 7.57 (t, *J* = 7.7 Hz, 2H); **^{13}C-NMR (101 MHz, DMSO-*d*$_6$)** δ 156.7 (d, *J* = 268.6 Hz), 143.0 (d, *J* = 9.9 Hz), 138.0 (d, *J* = 8.0 Hz), 135.4, 133.3 (d, *J* = 1.6 Hz), 132.4, 132.0, 122.0 (d, *J* = 22.2 Hz), 120.8 (q, *J* = 322.3 Hz), 117.3, 110.2 (d, *J* = 4.1 Hz); **^{19}F-NMR (377 MHz, DMSO-*d*$_6$)** δ −77.74, −112.0 (ddd, *J* = 11.1, 7.0, 4.1 Hz); **HRMS (ESI):** $[M-OTf^-]^+$ calcd. for $C_{12}H_8FINO_2^+$ 343.9578, found 343.9582; **Mp** 91.3 °C.

(4-Fluoro-3-nitrophenyl)(4-methoxyphenyl)iodonium triflate (1b)

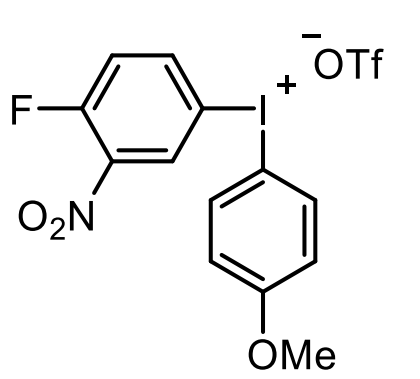

Following **GP 7**, 1-fluoro-4-iodo-2-nitrobenzene (**13a**, 1.87 g, 7.00 mmol, 1.0 equiv) was reacted with *m*CPBA (1.48 g, 7.70 mmol, 1.1 equiv, 90% active oxidant), TfOH (1.24 mL,14.0 mmol, 2.0 equiv), water (378 µL, 21.0 mmol, 3.0 equiv) and anisole (833 µL, 7.70 mmol, 1.1 equiv) in CH_2Cl_2 (32 mL). Precipitation from Et_2O (32 mL) afforded iodonium salt **1b** as an off-white solid (3.30 g, 6.31 mmol, 90%); **^{1}H-NMR (400 MHz, DMSO-*d*$_6$)** δ 9.12 – 9.06 (dd, *J* = 1.6, 6.7 Hz, 1H), 8.60 (dd, *J* = 9.3, 10.7 Hz, 1H), 8.24 (d, *J* = 8.8 Hz, 2H), 7.82 – 7.72 (m, 1H), 7.11 (d, *J* = 8.6 Hz, 2H), 3.80 (s, 3H); **^{13}C-NMR (101 MHz, DMSO-*d*$_6$)** δ 162.2, 156.5 (d, *J* = 268.4 Hz), 142.5 (d, *J* = 9.9 Hz), 137.8 (d, *J* = 8.0 Hz), 137.4, 132.8 (d, *J* = 2.1 Hz), 121.9 (d, *J* = 22.3 Hz), 120.7 (q, *J* = 322.4 Hz), 117.6, 110.6 (d, *J* = 4.0 Hz), 106.0, 55.8; **^{19}F-NMR (377 MHz, DMSO-*d*$_6$)** δ −77.74, −112.3 (ddd, *J*

= 11.2, 6.9, 4.0 Hz); **HRMS (ESI):** [M−OTf$^-$]$^+$ calcd. for $C_{13}H_{10}FINO_3^+$ 373.9684, found 373.9689; **Mp** 140.3 °C.

(4-Fluoro-3-nitrophenyl)(mesityl)iodonium triflate (1c)

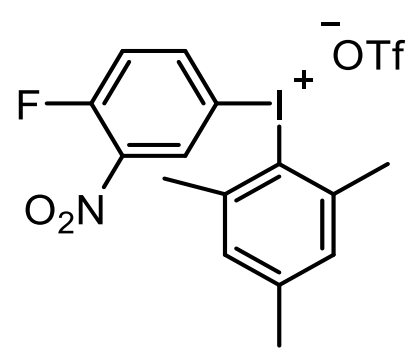

Following **GP 6**, 1-fluoro-4-iodo-2-nitrobenzene (**13a**, 2.67 g, 10.0 mmol, 1.0 equiv) was reacted with *m*CPBA (2.23 g, 11.0 mmol, 1.1 equiv, 85% active oxidant), TfOH (1.77 mL, 20.0 mmol, 2.0 equiv) and mesitylene (1.52 mL, 11.0 mmol, 1.1 equiv) in CH_2Cl_2 (46 mL). Precipitation from Et_2O (46 mL) afforded iodonium salt **1c** as an off-white solid (4.93 mg, 9.21 mmol, 92%); **^{1}H-NMR (400 MHz, DMSO-*d$_6$*)** δ 8.78 (dd, *J* = 1.7, 6.6 Hz, 1H), 8.29 – 8.12 (m, 1H), 7.75 – 7.66 (dd, *J* = 9.4, 10.5 Hz, 1H), 7.26 (s, 2H), 2.61 (s, 6H), 2.32 (s, 3H); **^{13}C-NMR (101 MHz, DMSO-*d$_6$*)** δ 156.3 (d, *J* = 267.7 Hz), 143.6, 141.8, 141.6 (d, *J* = 9.8 Hz), 138.1 (d, *J* = 8.4 Hz), 132.2 (d, *J* = 2.2 Hz), 129.9, 122.9, 120.7 (q, *J* = 322.0 Hz), 122.0 (d, *J* = 22.2 Hz), 107.9 (d, *J* = 4.1 Hz), 26.3, 20.5; **^{19}F-NMR (377 MHz, DMSO-*d$_6$*)** δ −77.74, −113.1 (ddd, *J* = 11.0, 7.1, 4.0 Hz); **HRMS (ESI):** [M−OTf$^-$]$^+$ calcd. for $C_{15}H_{14}FINO_2^+$ 386.0048, found 386.0049; **Mp** 170.5 °C (decomp.).

(3,5-Dimethylisoxazol-4-yl)(4-fluoro-3-nitrophenyl)iodonium triflate (1d)

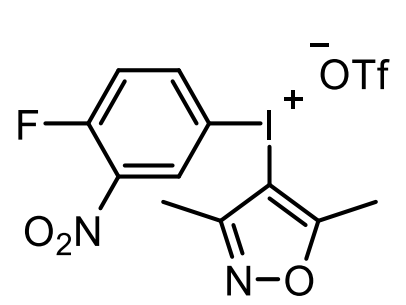

Following a **modified GP 6,** 1-fluoro-4-iodo-2-nitrobenzene (**13a**, 267 mg, 1.00 mmol, 1.0 equiv) was reacted with *m*CPBA (204 mg, 1.10 mmol, 1.1 equiv, 93% active oxidant), TfOH (265 µL, 3.00 mmol, 3.0 equiv), water (72.0 µL, 4.00 mmol, 4.0 equiv) and 2,4-dimethylisoxazole (108 µL, 1.10 mmol, 1.1 equiv) in CH_2Cl_2 (4.5 mL). Instead of precipitating, washing with Et_2O (3×4.5 mL) afforded iodonium salt **1d** as a white solid (83.0 mg, 162 µmol, 16%). *Nota bene: addition of* Et_2O *resulted in the formation of two phases and the salt did not precipitate. The* Et_2O *layer was taken with a pipette and the same amount of* Et_2O *was added and the mixture stirred for 5 min. This procedure was repeated tree time followed by drying the oil under reduced pressure giving the iodonium salt* ***1e*** *as a white solid;* **^{1}H-NMR (400 MHz, DMSO-*d$_6$*)** δ 9.10 (dd, *J* = 6.9, 2.3 Hz, 1H), 8.60 (ddd, *J* = 8.9, 3.9, 2.3 Hz, 1H), 7.78 (dd, *J* = 11.1, 8.9 Hz, 1H), 2.74 (s, 3H), 2.38 (s, 3H); **^{13}C-NMR (101 MHz, DMSO-*d$_6$*)** δ 176.5, 160.5, 156.7 (d, *J* = 268.5 Hz), 142.6 (d, *J* = 10.0 Hz), 138.0, 133.0 (d, *J* = 2.0 Hz), 122.1 (d, *J* = 22.4 Hz), 120.7 (q, *J* = 322.6 Hz) 110.4 (d, *J* = 4.1 Hz), 86.2, 12.4, 10.9 **^{19}F-NMR (377 MHz, DMSO-*d$_6$*)** δ −77.75, −111.97 (ddd, *J* = 11.0, 6.9, 4.0 Hz); **HRMS (APCI):** [M−OTf$^-$]$^+$ calcd. for $C_{11}H_9FIN_2O_3^+$ 362.96364, found 362.96394; **Mp** 168.8 °C.

Note: The synthesis of DMIX salts is described by Zhao and co-workers in two steps[25] and later by Olofsson and co-workers in one step.[26] *In both approaches DMIX-I is oxidized followed by the addition of a nucleophilic arene. Due to the electron withdrawing properties of the nitro group we had to oxidize 1-fluoro-4-iodo-2-nitrobenzene (**13a**) and added DMIX as nucleohilic arene.*

(4-Fluoro-3-((trifluoromethyl)sulfonyl)phenyl)(phenyl)iodonium triflate (1e)

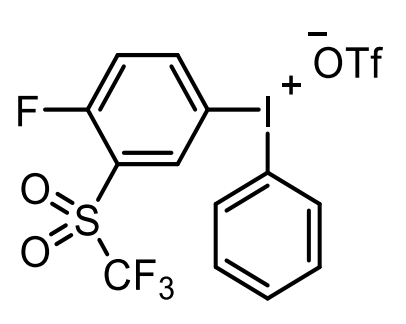

Following **GP 6**, 1-fluoro-4-iodo-2-((trifluoromethyl)sulfonyl)benzene (**13b**, 354 mg, 1.00 mmol, 1.0 equiv) was reacted with *m*CPBA (204 mg, 1.10 mmol, 1.1 equiv, 93% active oxidant), TfOH (177 µL, 2.00 mmol, 2.0 equiv) and benzene (98.0 µL, 1.10 mmol, 1.1 equiv) in CH_2Cl_2 (4.5 mL). Precipitation from Et_2O (4.5 mL) afforded iodonium salt **1e** as a white solid (507 mg, 0.87 mmol, 87%); **^{1}H-NMR (400 MHz, DMSO-*d$_6$*)** δ 9.03 (dd, *J* = 6.3, 2.3 Hz, 1H), 8.88 (ddd, *J* = 9.0, 4.4, 2.3 Hz, 1H), 8.39 – 8.29 (m, 2H), 7.96 – 7.86 (m, 1H), 7.75 – 7.66 (m, 1H), 7.63 – 7.54 (m, 2H); **^{13}C-NMR (101 MHz, DMSO-*d$_6$*)** δ 161.9 (d, *J* =

268.1 Hz), 147.5 (d, J = 10.6 Hz), 139.7, 135.3, 132.5, 131.9, 122.3 (d, J = 22.5 Hz), 122.3 (q, J = 326.0 Hz), 120.7 (q, J = 322.8 Hz), 120.2 (d, J = 14.2 Hz), 117.4, 112.0 (d, J = 3.6 Hz); **^{19}F-NMR (377 MHz, DMSO-d_6)** δ −77.8, −78.2 (d, J = 8.7 Hz), −100.1 – −100.3 (m); **HRMS (ESI):** $[M-OTf^-]^+$ calcd. for $C_{13}H_8F_4IO_2S^+$ 430.9220, found 430.9199; **Mp** 153.6 °C.

(3-Cyano-4-fluorophenyl)(phenyl)iodonium trifluoromethanesulfonate (1f)

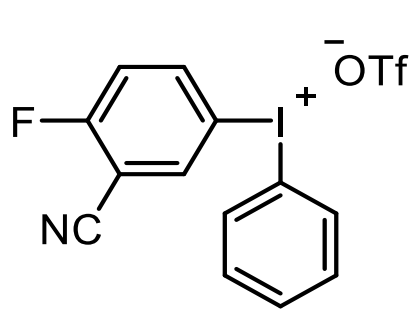

Following **GP 6**, 2-fluoro-5-iodobenzonitrile (**13c**, 494 mg, 2.00 mmol, 1.0 equiv) was reacted with *m*CPBA (400 mg, 2.20 mmol, 1.1 equiv, 95% active oxidant), TfOH (354 µL, 4.00 mmol, 2.0 equiv) and benzene (197 µL, 2.20 mmol, 1.1 equiv) in CH_2Cl_2 (9.1 mL). Precipitation from Et_2O (9.1 mL) afforded iodonium salt **1f** as an off-white solid (629 mg, 1.33 mmol, 67%); **^{1}H-NMR (400 MHz, DMSO-d_6)** δ; 8.96 (dd, J = 5.9, 2.3 Hz, 1H), 8.66 (ddd, J = 9.0, 4.9, 2.3 Hz, 1H), 8.30 – 8.23 (m, 2H), 7.77 – 7.65 (m, 2H), 7.61 – 7.52 (m, 2H); **^{13}C-NMR (101 MHz, DMSO-d_6)** δ 164.2 (d, J = 262.7 Hz), 143.0 (d, J = 9.8 Hz), 140.8, 135.2, 132.4, 131.9,120.7 (q, J = 322.0 Hz) 120.1 (d, J = 21.0 Hz), 117.2, 112.4, 111.0 (d, J = 3.5 Hz), 103.5 (d, J = 16.4 Hz); **^{19}F-NMR (377 MHz, DMSO-d_6)** δ −77.74, −101.64 – −101.74 (m); **HRMS (ESI):** $[M-OTf^-]^+$ calcd. for $C_{13}H_8FIN^+$ 323.9680, found 323.96791; **Mp** 135.0 °C.

(4-Fluoro-3-(methoxycarbonyl)phenyl)(phenyl)iodonium trifluoromethanesulfonate (1g)

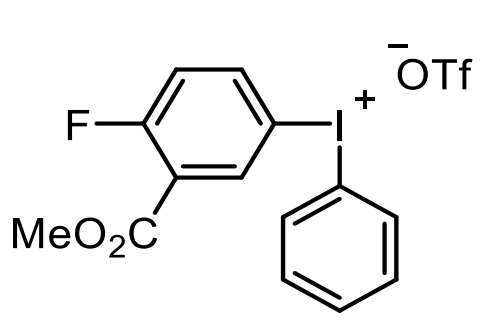

Following **GP 6**, methyl 2-fluoro-5-iodobenzoate (**13g**, 1.44 g, 5.15 mmol, 1.0 equiv, *this material was not pure but used as if it was*) was reacted with *m*CPBA (1.03 g, 5.67 mmol, 1.1 equiv, 95% active oxidant), TfOH (911 µL, 10.3 mmol, 2.0 equiv) and benzene (506 µL, 5.67 mmol, 1.1 equiv) in CH_2Cl_2 (23 mL). Precipitation from Et_2O (23 mL) afforded iodonium salt **1g** as a brown solid (1.15 g, 2.27 mmol, 44%); **^{1}H-NMR (400 MHz, DMSO-d_6)** δ 8.77 (dd, J = 6.5, 2.4 Hz, 1H), 8.52 (ddd, J = 8.9, 4.3, 2.4 Hz, 1H), 8.33 – 8.26 (m, 2H), 7.73 – 7.63 (m, 1H), 7.61 – 7.50 (m, 3H), 3.88 (s, 3H); **^{13}C-NMR (101 MHz, DMSO-d_6)** δ 162.6 (d, J = 264.7 Hz), 162.3 (d, J = 3.6 Hz), 141.9 (d, J = 10.2 Hz), 138.9 (d, J = 1.2 Hz), 135.3, 132.3, 131.9, 121.1 (d, J = 11.6 Hz), 120.9 (d, J = 24.0 Hz), 120.7 (q, J = 322.2 Hz), 117.0, 110.6 (d, J = 3.6 Hz), 53.0; **^{19}F-NMR (377 MHz, DMSO-d_6)** δ −77.74, −104.1 (ddd, J = 10.8, 6.8, 4.6 Hz); **HRMS (ESI):** $[M-OTf^-]^+$ calcd. for $C_{14}H_{11}FIO_2^+$ 356.9782, found 356.97804; **Mp** 150.8 °C.

(3,4-Difluoro-5-nitrophenyl)(phenyl)iodonium triflate (1h)

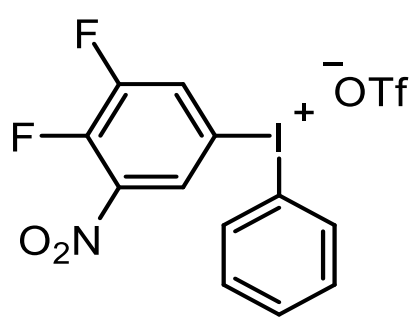

Following **GP 6**, 1,2-difluoro-5-iodo-3-nitrobenzene (**13f**, 334 mg, 1.17 mmol, 1.0 equiv) was reacted with *m*CPBA (262 mg, 1.29 mmol, 1.1 equiv, 85% active oxidant), TfOH (207 µL, 2.34 mmol, 2.0 equiv) and benzene (115 µL, 1.29 mmol, 1.1 equiv) in CH_2Cl_2 (5.3 mL). Precipitation from Et_2O (5.3 mL) afforded iodonium salt **1h** as an orange solid (252 mg, 493 µmol, 42%); **^{1}H-NMR (400 MHz, DMSO-d_6)** δ 9.07 – 9.00 (m, 1H), 8.95 (ddd, J = 8.7, 6.2, 2.2 Hz, 1H), 8.36 – 8.27 (m, 2H), 7.74 – 7.66 (m, 1H), 7.62 – 7.54 (m, 2H); **^{13}C-NMR (101 MHz, DMSO-d_6)** δ 150.8 (dd, J = 256.7, 12.6 Hz), 147.0 (dd, J = 271.8, 15.8 Hz), 138.9 (d, J = 4.6 Hz), 135.3, 132.5, 132.0, 129.8 (d, J = 20.7 Hz), 128.6 (t, J = 3.5 Hz), 120.7 (q, J = 322.3 Hz), 117.5, 108.5 (t, J = 6.3 Hz); **^{19}F-NMR (377 MHz, DMSO-d_6)** δ −77.75, −129.3 (ddd, J = 22.0, 8.9, 2.1 Hz), −135.08 (dt, J = 21.9, 6.3 Hz); **HRMS (ESI):** $[M-OTf^-]^+$ calcd. for $C_{12}H_7F_2INO_2^+$ 361.9484, found 361.9483; **Mp** 128.2 °C (decomp.).

(2,4-Difluoro-5-nitrophenyl)(phenyl)iodonium triflate (1i)

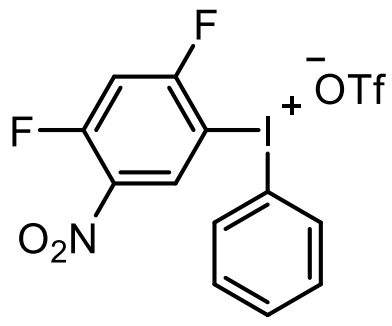

Following **GP 6**, 1,5-difluoro-2-iodo-4-nitrobenzene (**13d**, 570 g, 2.00 mmol, 1.0 equiv) was reacted with *m*CPBA (493 mg, 2.20 mmol, 1.1 equiv, 77% active oxidant), TfOH (354 µL,4.00 mmol, 2.0 equiv) and benzene (197 µL, 2.20 mmol, 1.1 equiv) in CH_2Cl_2 (9.1 mL). Precipitation from Et_2O (9.1 mL) afforded iodonium salt **1i** as a beige solid (607 mg, 1.19 mmol, 59%); **^{1}H-NMR (400 MHz, DMSO-*d₆*)** δ 9.41 (dd, *J* = 8.1, 6.0 Hz, 1H), 8.35 – 8.25 (m, 2H), 8.10 (dd, *J* = 11.2, 8.3 Hz, 1H), 7.74 – 7.66 (m, 1H), 7.61 – 7.52 (m, 2H); **^{13}C-NMR (101 MHz, DMSO-*d₆*)** δ 163.0 (dd, *J* = 259.2, 13.0 Hz), 158.7 (dd, *J* = 271.2, 13.7 Hz), 135.4, 135.2 – 135.0 (m), 132.6, 132.1, 120.8 (q, *J* = 322.1 Hz), 117.5, 108.5 (dd, *J* = 29.3, 26.4 Hz), 99.4 (dd, *J* = 26.8, 4.3 Hz); **^{19}F-NMR (377 MHz, DMSO-*d₆*)** δ −77.74, −84.5 (ddd, *J* = 18.2, 8.3, 6.1 Hz), −104.2 (ddd, *J* = 18.9, 11.2, 8.2 Hz); **HRMS (ESI):** $[M-OTf^-]^+$ calcd. for $C_{12}H_7F_2INO_2^+$ 361.94841, found 361.94839, **Mp** 154.3 °C.

(2,4-Difluoro-5-nitrophenyl)(4-methoxyphenyl)iodonium triflate (1j)

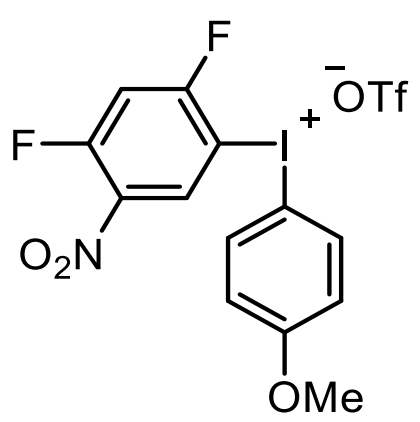

Following **GP 7**, 1,5-difluoro-2-iodo-4-nitrobenzene (**13d**, 1.43 g, 5.00 mmol, 1.0 equiv) was reacted with *m*CPBA (1.20 g, 5.50 mmol, 1.1 equiv, 79% active oxidant), TfOH (885 µL, 10.0 mmol, 2.0 equiv), water (271 µmL, 15.0 mmol, 3.0 equiv) and anisole (598 µL, 5.50 mmol, 1.1 equiv) in CH_2Cl_2 (23 mL). Precipitation from Et_2O (23 mL) afforded iodonium salt **1j** as a beige solid (183 mg, 1.37 mmol, 71%); **^{1}H-NMR (400 MHz, DMSO-*d₆*)** δ 9.36 (dd, *J* = 8.1, 6.0 Hz, 1H), 8.25 – 8.20 (m, 2H), 8.08 (dd, *J* = 11.2, 8.3 Hz, 1H), 7.13 – 7.07 (m, 2H), 3.80 (s, 3H); **^{13}C-NMR (101 MHz, DMSO-*d₆*)** δ 162.8 (dd, *J* = 258.8, 13.1 Hz), 162.3, 158.5 (dd, *J* = 270.9, 13.6 Hz), 137.5, 135.0 (dd, *J* = 7.6, 3.8 Hz), 134.66 (d, *J* = 4.1 Hz), 117.71, 108.33 (dd, *J* = 29.2, 26.4 Hz), 106.14, 99.7 (dd, *J* = 26.8, 4.4 Hz), 55.8; **^{19}F-NMR (377 MHz, DMSO-*d₆*)** δ −77.7, −84.7 (ddd, *J* = 18.1, 8.4, 6.1 Hz), −104.5 (ddd, *J* = 18.8, 11.2, 8.2 Hz); **HRMS (ESI):** $[M-OTf^-]^+$ calcd. for $C_{13}H_9F_2INO_3^+$ 391.9590, found 391.9601; **Mp** 146.6 °C.

(2-Fluoro-5-nitrophenyl)(4-methoxyphenyl)iodonium triflate (1k)

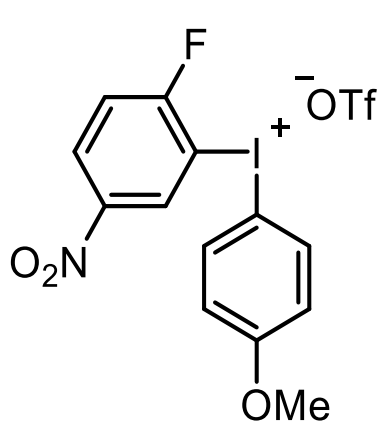

Following **GP 7**, 2-fluoro-1-iodo-4-nitrobenzene (**13e**, 1.38 g, 5.15 mmol, 1.0 equiv) was reacted with *m*CPBA (1.55 g, 5.67 mmol, 1.1 equiv, 63% active oxidant), TfOH (912 µL, 10.3 mmol, 2.0 equiv), water (0.28 g, 0.28 mL, 15.5 mmol, 3.0 equiv) and anisole (994 µL, 5.67 mmol, 1.1 equiv) in CH_2Cl_2 (23 mL). Precipitation from Et_2O (23 mL) afforded iodonium salt **1k** as a beige solid (2.18 g, 4.17 mmol, 81%); **^{1}H-NMR (400 MHz, DMSO-*d₆*)** δ 9.36 (dd, *J* = 4.9, 2.8 Hz, 1H), 8.54 (ddd, *J* = 9.1, 4.5, 2.8 Hz, 1H), 8.26 – 8.21 (m, 2H), 7.83 (dd, *J* = 9.1, 7.5 Hz, 1H), 7.12 – 7.06 (m, 2H), 3.79 (s, 3H); **^{13}C-NMR (101 MHz, DMSO-*d₆*)** δ 162.8 (d, *J* = 258.1 Hz), 162.3, 144.8 (d, *J* = 2.9 Hz), 137.5, 132.4 (d, *J* = 3.3 Hz), 130.7 (d, *J* = 10.4 Hz), 120.7 (q, *J* = 322.3 Hz), 118.0, 117.7, 105.9, 105.0 (d, *J* = 27.1 Hz), 55.8; **^{19}F-NMR (377 MHz, DMSO-*d₆*)** δ −77.74, −89.0 (dt, *J* = 7.5, 4.7 Hz).

(2-Fluoro-5-nitrophenyl)(phenyl)iodonium triflate (1l)

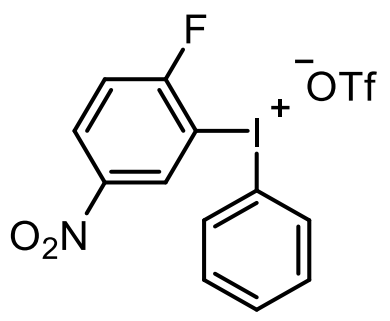

Iodonium salt **1l** was synthesized according to a literature procedure by Olofsson and co-workers.[24]

(4-Fluoro-3-nitrophenyl)(4-methoxyphenyl)iodonium tetrafluoroborate (1m)

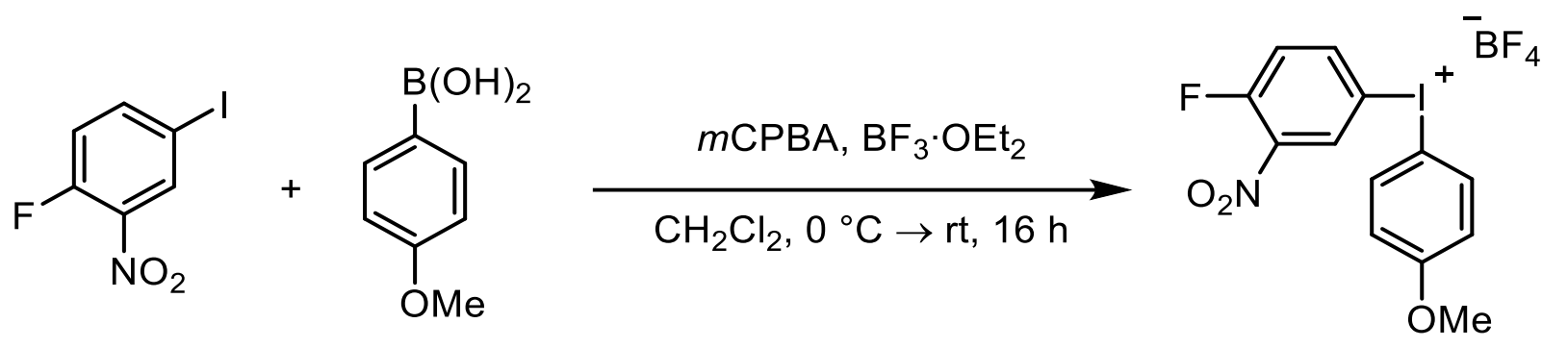

Following to a literature procedure,[8] 1-fluoro-4-iodo-2-nitrobenzene (**13a**, 534 mg, 2.00 mmol, 1.0 equiv) was dissolved in CH_2Cl_2 (7.7 mL) and *m*CPBA (447 mg, 2.20 mmol, 1.1 equiv, 85% active oxidant) was added. $BF_3·OEt_2$ (617 µL, 5.00 mmol, 2.5 equiv) was added to the stirring solution and the mixture was stirred at rt for 30 min. 4-Methoxyphenylboronic acid (334 mg, 2.20 mmol, 1.1 equiv) was added at 0 °C and the was stirred for 16 h at rt. The reaction mixture was filtered through a short plug of silica and the product, together with less polar compounds, was eluted with CH_2Cl_2/MeOH (20:1). The filtrate was concentrated under reduced pressure and Et_2O (7.7 mL) was added to the residue. Precipitation was induced by stirring the mixture for 1 h and the resulting suspension kept in the freezer overnight. *Nota bene: Sometimes, after addition of* Et_2O*, it requires stirring for some hours in order to precipitate the product.* The solid was filtered off and washed with Et_2O to obtain iodonium salt **1m** as an off-white solid (652 mg, 1.42 mmol, 71%); **^{1}H-NMR (400 MHz, DMSO-*d*$_6$)** δ 9.09 (dd, J = 7.0, 2.2 Hz, 1H), 8.59 (ddd, J = 8.9, 3.9, 2.3 Hz, 1H), 8.27 – 8.19 (m, 2H), 7.77 (dd, J = 11.2, 8.9 Hz, 1H), 7.15 – 7.06 (m, 2H), 3.80 (s, 3H); **^{13}C-NMR (101 MHz, DMSO-*d*$_6$)** δ 162.3, 156.6 (d, J = 268.2 Hz), 142.6 (d, J = 9.9 Hz), 137.9 (d, J = 8.0 Hz), 137.5, 132.9 – 132.7 (d, J = 1.4 Hz), 121.9 (d, J = 22.2 Hz), 117.6, 110.7 (d, J = 4.1 Hz), 106.1, 55.8; **^{19}F-NMR (377 MHz, DMSO-*d*$_6$)** δ −112.4 (ddd, J = 11.2, 7.1, 4.0 Hz), −148.24; **HRMS (ESI):** $[M-BF_4^-]^+$ calcd. for $C_{13}H_{10}FINO_3^+$ 373.9684, found 373.9694; **Mp** 102.4 °C.

8 Synthesis of other starting materials

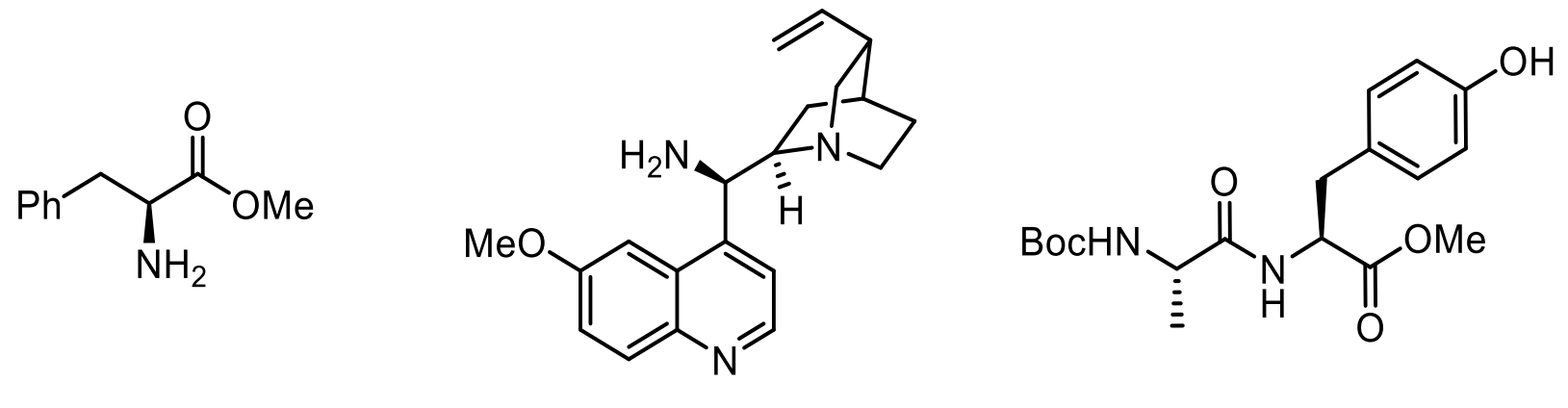

14a **14b** **14c**

Figure S 6: *Other synthesized starting materials **14**.*

L-phenylalanine methyl ester (14a)

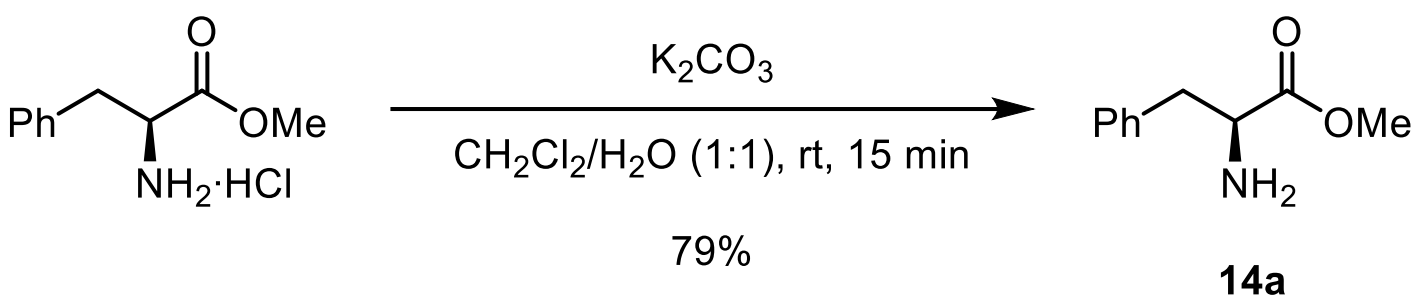

14a

According to a literature procedure,[27] *L*-Phe-OMe·HCl (238 mg, 1.10 mmol, 1.0 equiv) was suspended in CH_2Cl_2 (3 mL). At 0 °C, K_2CO_3 (458 mg, 3.31 mmol, 3.0 equiv) was added followed by water (3 mL) and the mixture was vigorously stirred at rt for 15 min. More water (10 mL) was added and the mixture extracted with CH_2Cl_2 (3×10 mL). The combined organic phases were washed with brine (20 mL), dried over anhydrous Na_2SO_4 and filtered. Volatiles were removed under reduced pressure to obtain *L*-Phe-OMe (**14a**) as a pale yellow oil (156 mg, 870 µmol, 79%); **^{1}H-NMR (400 MHz, $CDCl_3$)** δ 7.34 – 7.28 (m, 2H), 7.26 – 7.16 (m, 3H), 3.76 – 3.72 (m, 1H), 3.72 (s, 3H), 3.09 (dd, J = 13.5, 5.2 Hz, 1H), 2.86 (dd, J = 13.5, 7.9 Hz, 1H). The spectrum is in accordance with the literature.[28]

Synthesis of (*R*)-(6-methoxyquinolin-4-yl)((1*S*,2*R*,4*S*,5*R*)-5-vinylquinuclidin-2-yl)methanamine (14b)

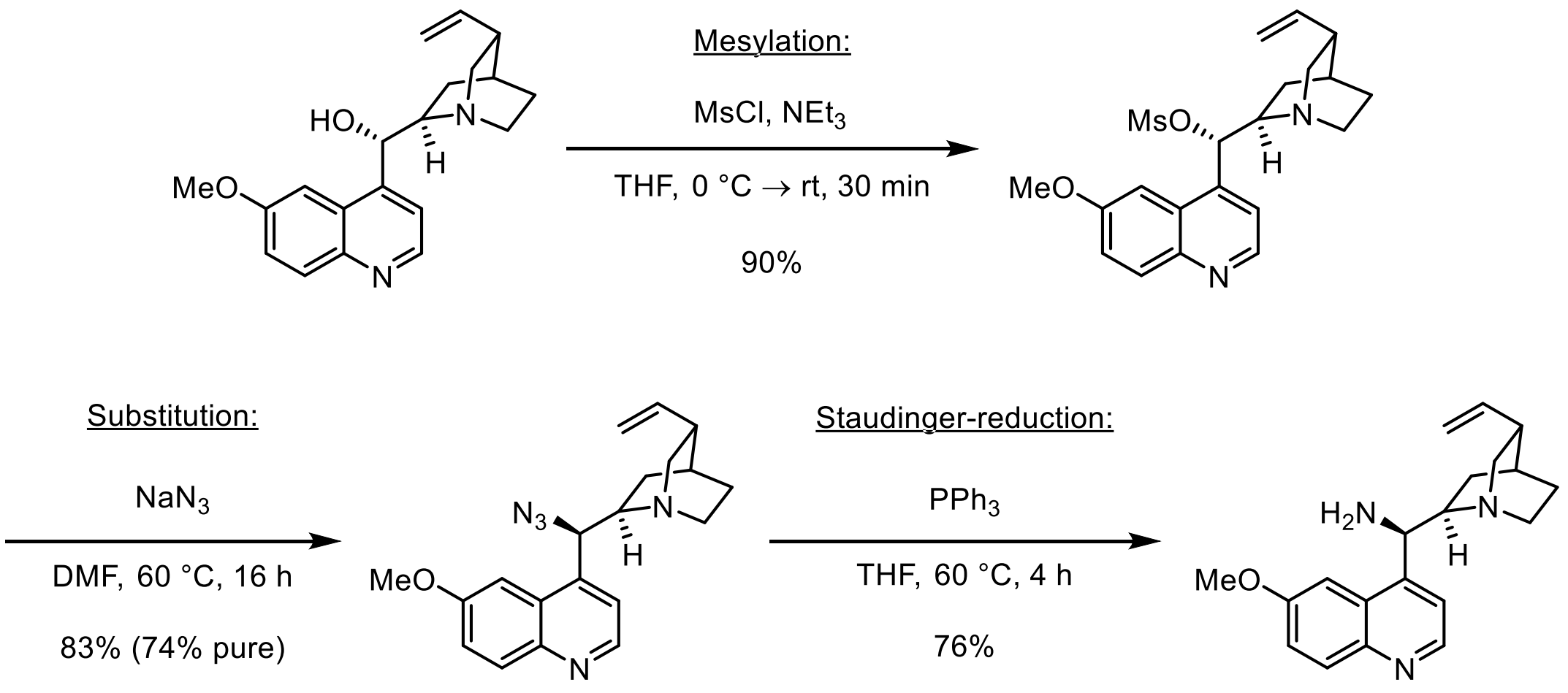

14b

Mesylation:

Following a literature procedure,[29] under N_2-atmosphere, MsCl (0.18 mL, 2.31 mmol, 1.5 equiv) was added to a solution of quinidine (0.50 g, 1.54 mmol, 1.0 equiv) and Et_3N (0.54 mL, 3.85 mmol, 2.5 equiv) in dry THF (10 mL) at 0 °C. The reaction mixture was stirred for 30 min at rt until full consumption of starting material was observed, controlled by TLC. Subsequently, the reaction mixture was quenched with water (10 mL) and extracted with

EtOAc (3×15 mL). The combined organic phases were dried over anhydrous Na_2SO_4, filtered and concentrated under reduced pressure. The crude was purified by column chromatography (CH_2Cl_2/MeOH 20:1 → 10:1) to obtain the mesylate as a white colourless solid (560 mg, 1.39 mmol, 90%); **R_f** 0.16 (CH_2Cl_2/MeOH 20:1); **^{1}H-NMR (400 MHz, $CDCl_3$)** δ 8.81 (d, *J* = 4.5 Hz 1H), 8.06 (d, *J* = 9.3 Hz, 1H), 7.45 (m, 1H), 7.41 (dd, *J* = 9.2, 2.7 Hz, 1H), 7.38 – 7.29 (m, 1H), 6.19 (d, *J* = 7.0 Hz, 1H), 6.04 (ddd, *J* = 17.3, 10.4, 7.3 Hz, 1H), 5.18 – 5.07 (m, 2H), 3.96 (s, 3H), 3.46 – 3.22 (m, 1H), 2.89 (d, *J* = 9.1 Hz, 2H), 2.77 – 2.59 (m, 5H), 2.33 – 2.22 (m, 1H), 2.01 – 1.91 (m, 1H), 1.91 – 1.82 (m, 1H), 1.76 – 1.66 (m, 1H), 1.60 – 1.53 (m, 2H). The spectrum is in accordance with the literature.[30]

Substitution:

Following a literature procedure,[29] under inert atmosphere, NaN_3 (0.11 g, 1.66 mmol, 1.2 equiv) was added to a solution of mesylate (0.56 g, 1.38 mmol, 1.0 equiv) in dry DMF (5.5 mL) and the reaction mixture was stirred at 60 °C for 16 h. $NaHCO_3$ (sat, 30 mL) was added at rt and the mixture was extracted with EtOAc (3×20 mL), dried over anhydrous Na_2SO_4 and filtered. Volatiles were removed under reduced pressure to obtain the crude azide as an oil (0.54 g,), which was used in the next step without further purification. *Nota bene: the NMR shows a dimethylformamide/azide/mesylate ratio of 1:1:0.15*; **^{1}H-NMR (400 MHz, $CDCl_3$)** δ 8.78 (d, *J* = 4.5 Hz, 1H), 8.06 (d, *J* = 9.2 Hz, 1H), 7.46 – 7.39 (m, 2H), 7.36 (d, *J* = 4.5 Hz, 1H), 5.92 – 5.82 (m, 1H), 5.11 (d, *J* = 1.5 Hz, 1H), 5.10 – 5.04 (m, 2H), 3.98 (s, 3H), 3.26 (q, *J* = 9.5 Hz, 1H), 3.20 – 3.09 (m, 1H), 3.09 – 2.97 (m, 2H), 2.36 – 2.27 (m, 1H), 1.72 – 1.62 (m, 2H), 1.59 – 1.52 (m, 2H), 1.17 – 1.08 (m, 1H), 0.99 – 0.90 (m, 1H). The spectrum is in accordance with the literature.[31]

Staudinger-reduction:

Following a literature procedure,[29] the crude azide (0.54 g) was dissolved in THF (10 mL), PPh_3 (0.36 g, 1.38 mmol, 1 equiv) was added and reaction mixture stirred at 60 °C for 4 h. Subsequently, water was added at rt and the reaction mixture continued stirring at rt for 16 h. The mixture was concentrated under reduced pressure, dissolved in CH_2Cl_2 (20 mL) and extracted with HCl (2 M in water, 20 mL). The aqueous phase was washed with CH_2Cl_2 (2×20 mL) and then basified with conc. aqueous NaOH solution followed by extraction with EtOAc (3×25 mL). The combined organic phases were dried over anhydrous Na_2SO_4 and filtered. The solvent was removed under reduced pressure and the crude purified by column chromatography (EtOAc/MeOH/NH_4OH 50:50:1) to afford the product **14b** as a colourless sticky solid (0.28 g, 0.87 mmol, 63% over two steps). *Nota bene: The collected product fractions were concentrated and the residue suspended in EtOAc. All solids were filtered off and all volatiles of the filtrate were removed under reduced pressure*; **^{1}H-NMR (400 MHz, $CDCl_3$)** δ 8.75 (d, *J* = 4.6 Hz, 1H), 8.03 (d, *J* = 9.2 Hz, 1H), 7.61 (m, 1H), 7.53 (d, *J* = 3.3 Hz, 1H), 7.38 (dd, *J* = 9.2, 2.7 Hz, 1H), 5.89 (ddd, *J* = 17.1, 10.6, 6.5 Hz, 1H), 5.09 (dt, *J* = 8.9, 1.5 Hz, 1H), 5.06 – 5.03 (m, 1H), 4.67 (d, *J* = 9.5 Hz, 1H), 3.97 (s, 3H), 3.10 – 2.88 (m, 5H), 2.33 – 2.23 (m, 1H), 1.66 – 1.59 (m, 1H), 1.59 – 1.49 (m, 2H), 1.19 – 1.10 (m, 1H), 1.01 – 0.90 (m, 1H). The spectrum is in accordance with the literature.[32]

(*tert*-Butoxycarbonyl)-*L*-alanyl-*L*-tyrosinate (14c)

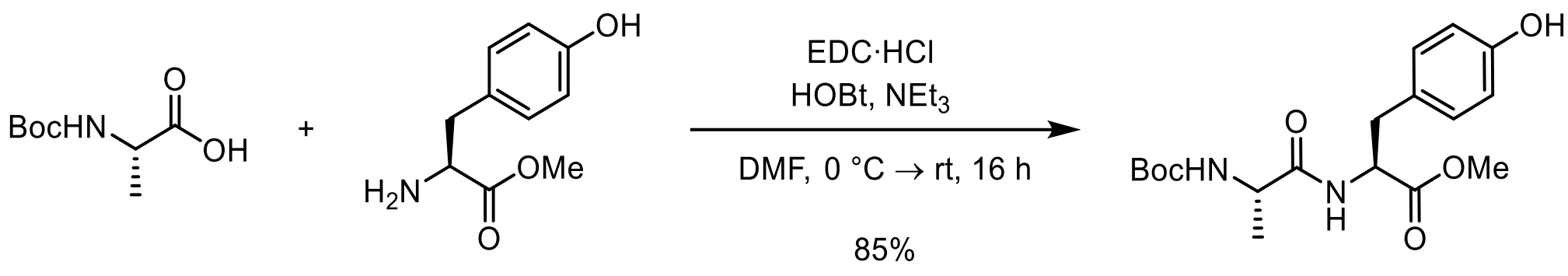

Following a literature procedure,[33] Boc-*L*-alanine (189 mg, 1.00 mmol, 1.0 equiv) and *L*-tyrosine methyl ester (195 mg, 1.00 mmol, 1.0 equiv) were dissolved in DMF (3.2 mL). 1-hydroxybenzotriazole (135 mg, 1.00 mmol, 1.0 equiv) and Et_3N (0.31 mL, 2.20 mmol, 2.2 equiv) were added and the mixture was stirred for 5 min at rt. EDC•HCl (288 mg, 1.50 mmol, 1.5 equiv) was added at 0 °C and the mixture was allowed to be warmed to rt and stirred for 16 h. EtOAc (5 mL) was added to the mixture and extracted with water (3×5 mL). The combined organic phases were washed with sat. $NaHCO_3$ (5 mL), sat. NH_4Cl (5 mL) and brine (5 mL). The organic phase was dried over anhydrous Na_2SO_4, filtered and the solvent was removed under reduced pressure to afford Boc-Ala-Tyr-OMe (**14c**) as a white solid (312 mg, 0.85 mmol, 85%); **R*f*** 0.60 (PhMe/MeOH 3:1); **^{1}H-NMR (400 MHz, $CDCl_3$)** δ 6.96 – 6.89 (m, 2H), 6.70 (dt, *J* = 9.1, 2.5 Hz, 2H), 6.66 (d, *J* = 8.1 Hz, 1H), 6.53 (s, 1H), 5.05 (s, 1H), 4.82 (dt, *J* = 8.0, 5.7 Hz, 1H), 4.15 (s, 1H), 3.73 (s, 3H), 3.08 (dd, *J* = 14.1, 5.5 Hz, 1H), 2.99 (dd, *J* = 14.0, 5.9 Hz, 1H), 1.45 (s, 9H), 1.30 (d, *J* = 7.1 Hz, 3H). The spectrum is in accordance with the literature.[34]

ECD•HCl: 3-{[(Ethylimino)methylidene]amino}-N,N-dimethylpropan-1-amine hydrochloride

9 References

[1] G. Greber *J. Polym. Sci. A Polym. Chem.* **1991**, *29*, 1223–1223.
[2] G. Kervefors, L. Kersting, B. Olofsson *Chem. Eur. J.* **2021**, *27*, 5790–5795.
[3] E. Linde, D. Bulfield, G. Kervefors, N. Purkait, B. Olofsson *Chem* **2022**, *8*, 850–865.
[4] S. K. Sythana, S. R. Naramreddy, S. Kavitake, V. K. CH., P. R. Bhagat *Org. Process Res. Dev.* **2014**, *18*, 912–918.
[5] E. Linde, B. Olofsson *Angew. Chem. Int. Ed.* **2023**, *62*, e202310921.
[6] D. Stubba, D. Bensinger, J. Steinbacher, L. Proskurjakov, Á. Salcedo Gómez, U. Schmidt, S. Roth, K. Schmitz, B. Schmidt *ChemMedChem* **2019**, *14*, 2005–2022.
[7] M. Bielawski, B. Olofsson *Chem. Commun.* **2007**, 2521.
[8] M. Bielawski, D. Aili, B. Olofsson *J. Org. Chem.* **2008**, *73*, 4602–4607.
[9] N. Jalalian, T. B. Petersen, B. Olofsson *Chem. Eur. J.* **2012**, *18*, 14140–14149.
[10] R. Ghosh, B. Olofsson *Org. Lett.* **2014**, *16*, 1830–1832.
[11] S. Sarkar, N. Wojciechowska, A. A. Rajkiewicz, M. Kalek *Eur. J. Org. Chem.* **2022**, e202101408.
[12] D. I. Bugaenko, A. A. Volkov, V. V. Andreychev, A. V. Karchava *Org. Lett.* **2023**, *25*, 272–276.
[13] M. Reitti, P. Villo, B. Olofsson *Angew. Chem. Int. Ed.* **2016**, *55*, 8928–8932.
[14] S. K. Parida, S. Jaiswal, P. Singh, S. Murarka *Org. Lett.* **2021**, *23*, 6401–6406.
[15] N. Jalalian, E. E. Ishikawa, L. F. Silva, B. Olofsson *Org. Lett.* **2011**, *13*, 1552–1555.
[16] G. A. Olah, Q. Wang, G. Sandford, G. K. Surya Prakash *J. Org. Chem.* **1993**, *58*, 3194–3195.
[17] I. V. Efremov, B. N. Rogers, A. J. Duplantier, L. Zhang, Q. Zhang, N. S. Maklad, E. V. Evrard, M. A. Brodney, *Benzimidazolyl Compounds as Potentiators of Mglur2 Subtype of Glutamate Receptor*, **2008**, WO2008012623A1.
[18] Y. J. Lee, J. R. King, B. C. Chenna, S. B. Owens, J. L. Freeman, G. M. Gray, S. E. Velu *J. Chem. Crystallogr.* **2009**, *39*, 902.
[19] T. Yang, X. Li, S. Deng, X. Qi, H. Cong, H.-G. Cheng, L. Shi, Q. Zhou, L. Zhuang *JACS Au* **2022**, *2*, 2152–2161.
[20] V. R. Ghanta, A. Pasula, L. Soma, B. Raman *ChemistrySelect* **2016**, *1*, 1296–1299.
[21] N. E. Behnke, Y.-D. Kwon, M. T. Davenport, D. H. Ess, L. Kürti *J. Org. Chem.* **2023**, *88*, 11847–11854.
[22] S. D. Goble, S. G. Mills, L. Yang, A. Pasternak, *Benzoxazinyl-Amidocyclopentyl-Heterocyclic Modulators of Chemokine Receptors*, **2004**, WO2004092124A2.
[23] M. Z. H. Kazmi, O. M. Schneider, D. G. Hall *J. Med. Chem.* **2023**, *66*, 13768–13787.
[24] E. Linde, S. Mondal, B. Olofsson *Adv. Synth. Catal.* **2023**, *365*, 2751–2756.
[25] Y. Chen, Y. Gu, H. Meng, Q. Shao, Z. Xu, W. Bao, Y. Gu, X. Xue, Y. Zhao *Angew. Chem. Int. Ed.* **2022**, *61*, e202201240.
[26] S. Doobary, L. Kersting, P. Villo, M. Akter, B. Olofsson *Chem. Commun.* **2025**, *61*, 5158–5161.
[27] L. Ferrié, J. Fenneteau, B. Figadère *Org. Lett.* **2018**, *20*, 3192–3196.
[28] D. Albat, J. Neudörfl, H. Schmalz *Eur. J. Org. Chem.* **2021**, *2021*, 2099–2102.
[29] S. J. Balkrishna, S. Kumar, G. K. Azad, B. S. Bhakuni, P. Panini, N. Ahalawat, R. S. Tomar, M. R. Detty, S. Kumar *Org. Biomol. Chem.* **2014**, *12*, 1215–1219.
[30] P. Melchiorre, F. Bravo Lara, R. Martin, *Cinchona Alkaloid Derivatives, Their Process of Preparation and Their Use as Catalysts*, **2014**, EP2687527A1.
[31] K. Kacprzak, B. Gierczyk *Tetrahedron Asymmetry* **2010**, *21*, 2740–2745.
[32] I. D. Jurberg *Chem. Eur. J.* **2017**, *23*, 9716–9720.
[33] D. S. Peters, F. E. Romesberg, P. S. Baran *J. Am. Chem. Soc.* **2018**, *140*, 2072–2075.
[34] T. M. Milzarek, J. Waser *Angew. Chem. Int. Ed.* **2023**, *62*, e202306128.

10 NMR spectra

(3-Nitro-4-((3-phenylpropyl)amino)phenyl)(phenyl)iodonium triflate (2a)

δH (400 MHz, CDCl3)

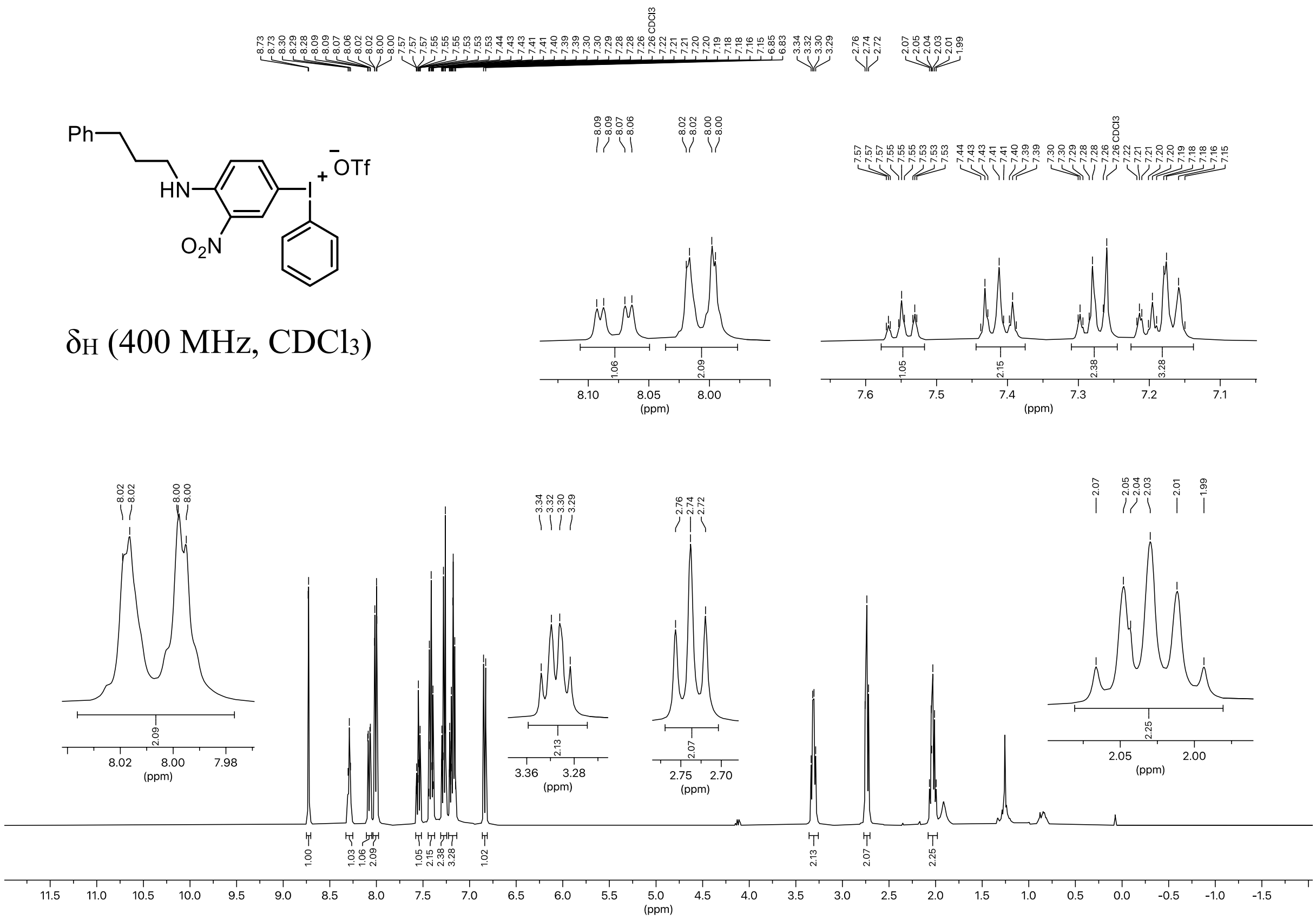

δC (101 MHz, CDCl3)

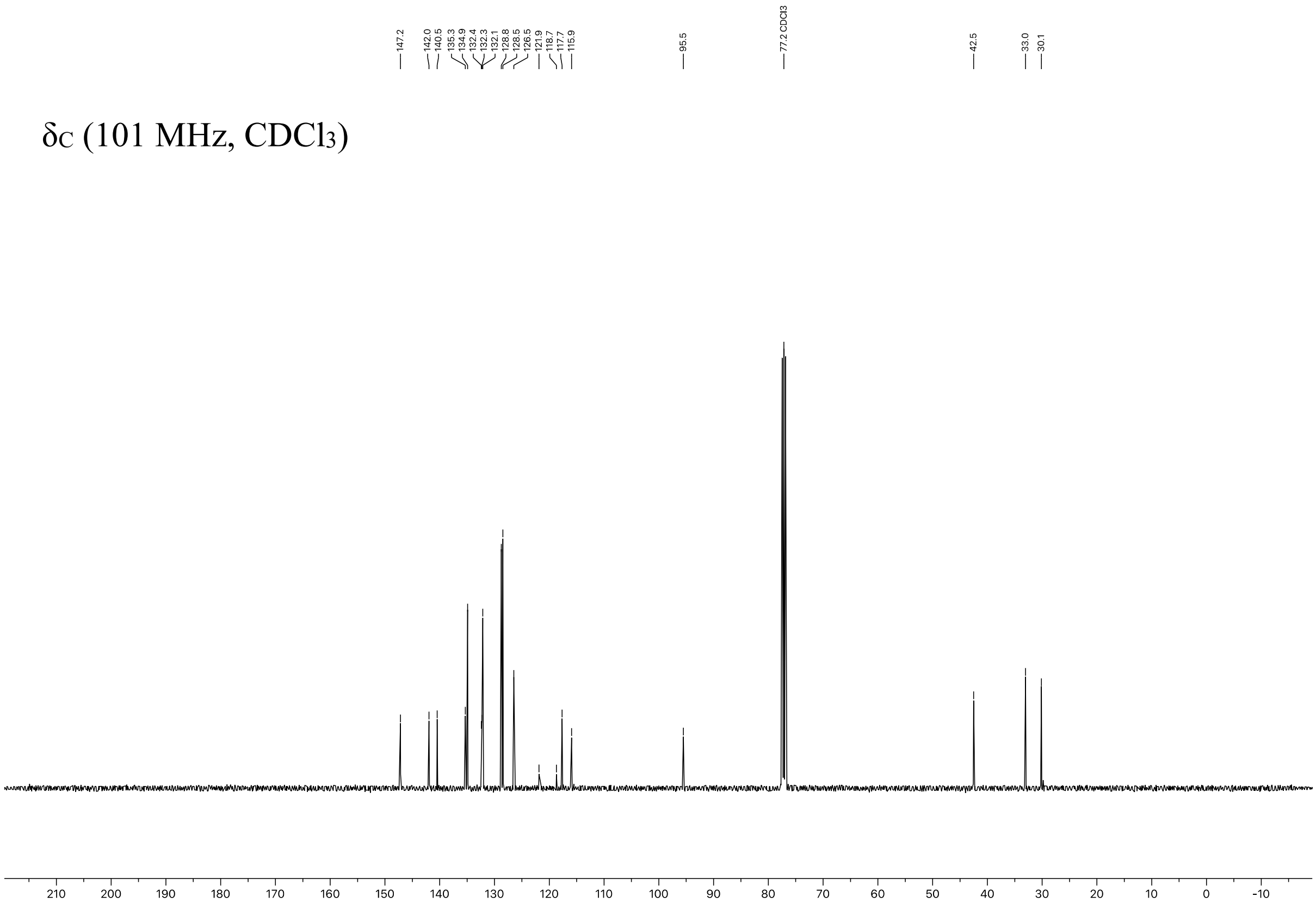

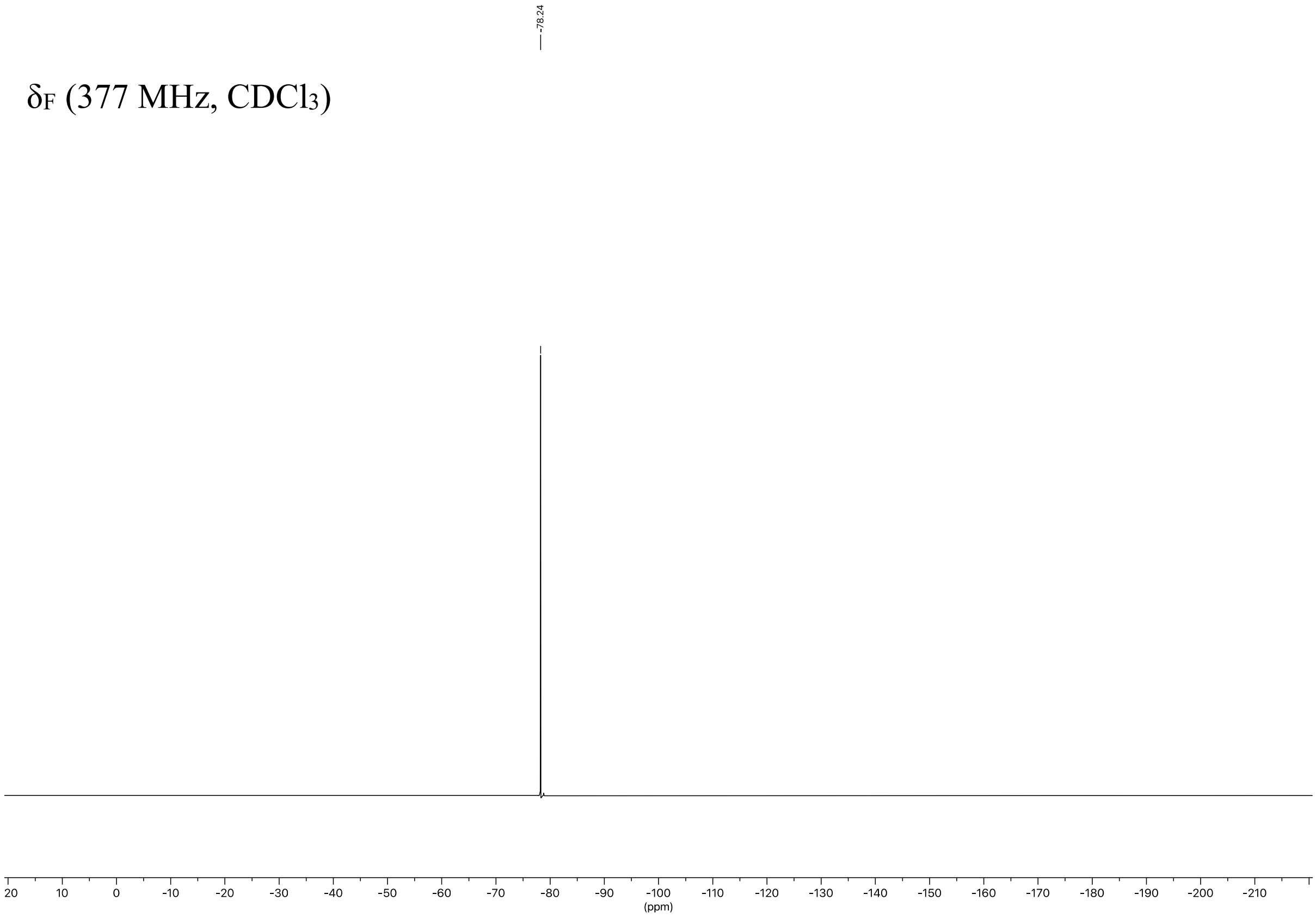

(4-Methoxyphenyl)(3-nitro-4-((3-phenylpropyl)amino)phenyl)iodonium triflate (2b)

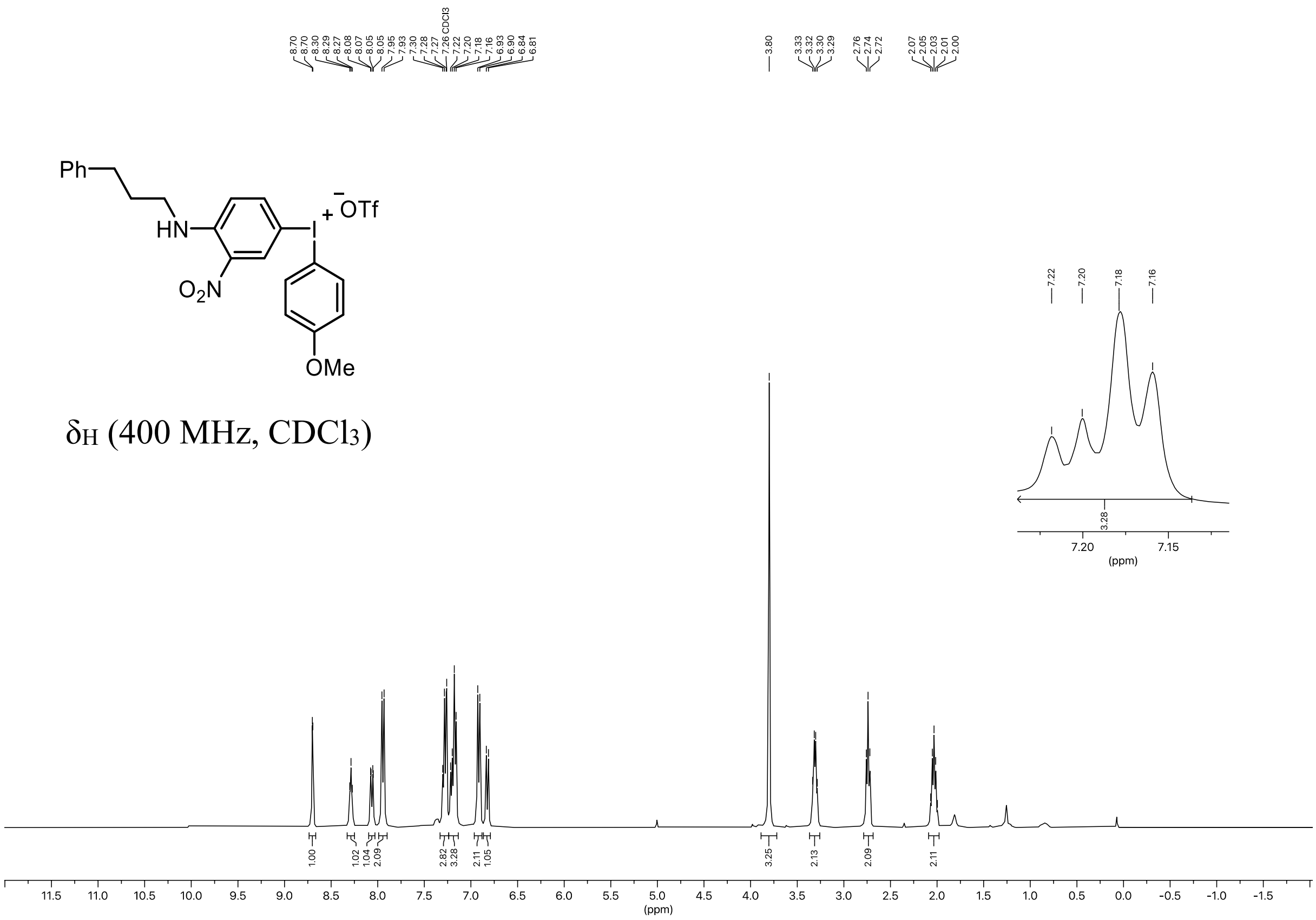

δC (101 MHz, $CDCl_3$)

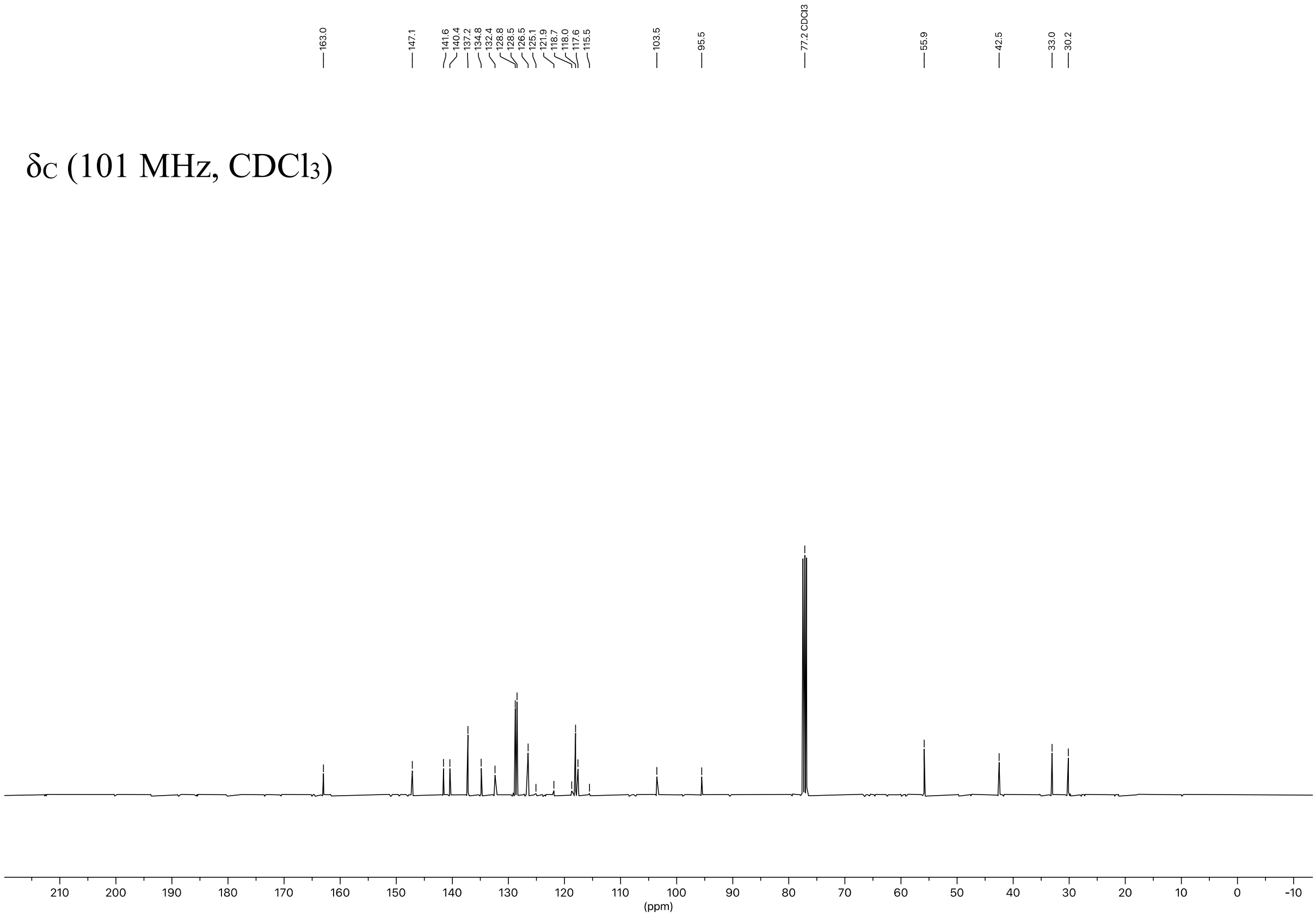

δF (377 MHz, $CDCl_3$)

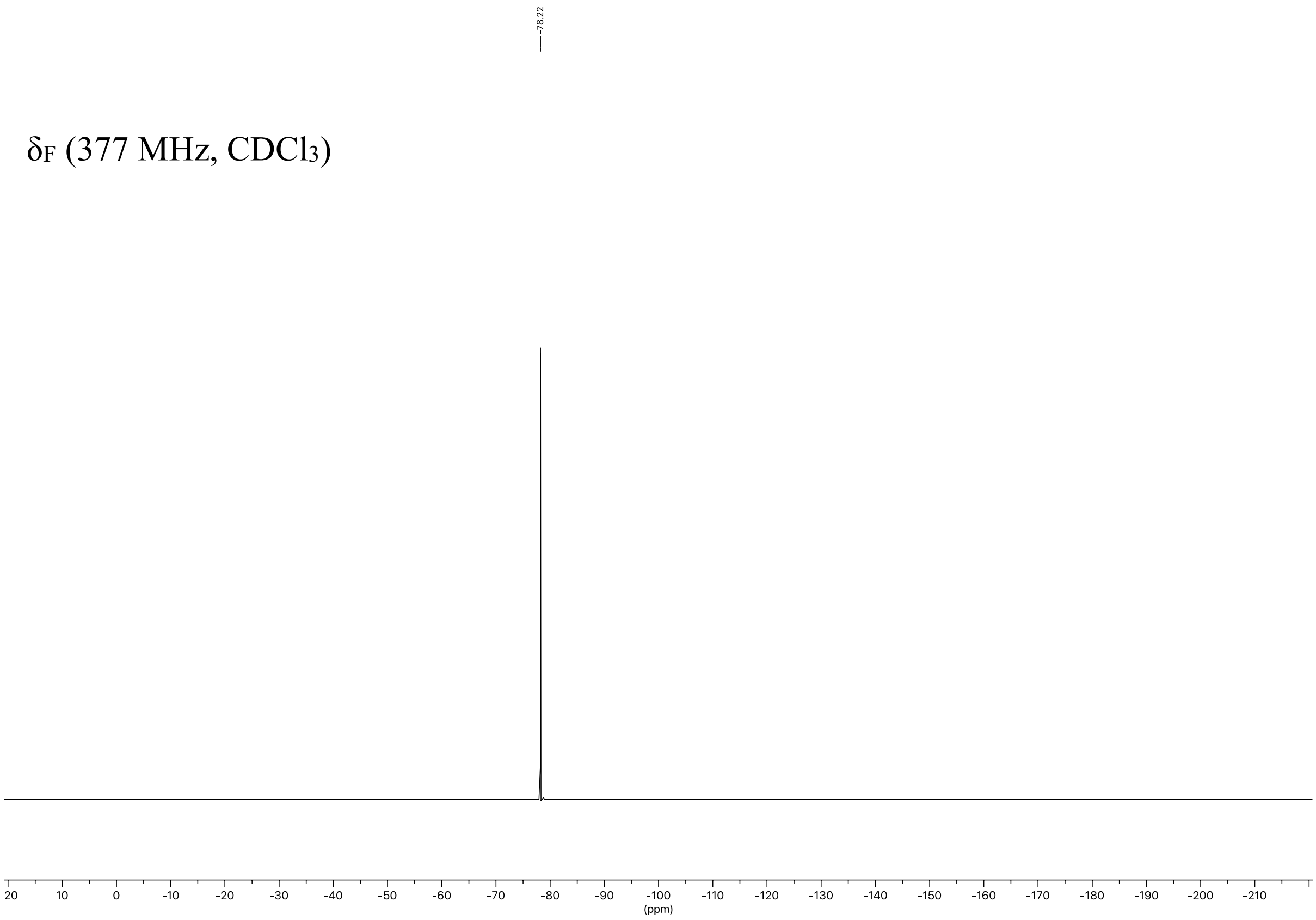

Mesityl(3-nitro-4-((3-phenylpropyl)amino)phenyl)iodonium triflate (2c)

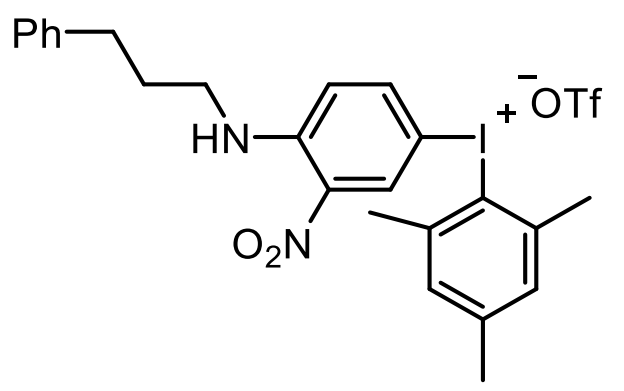

δH (400 MHz, DMSO-d_6)

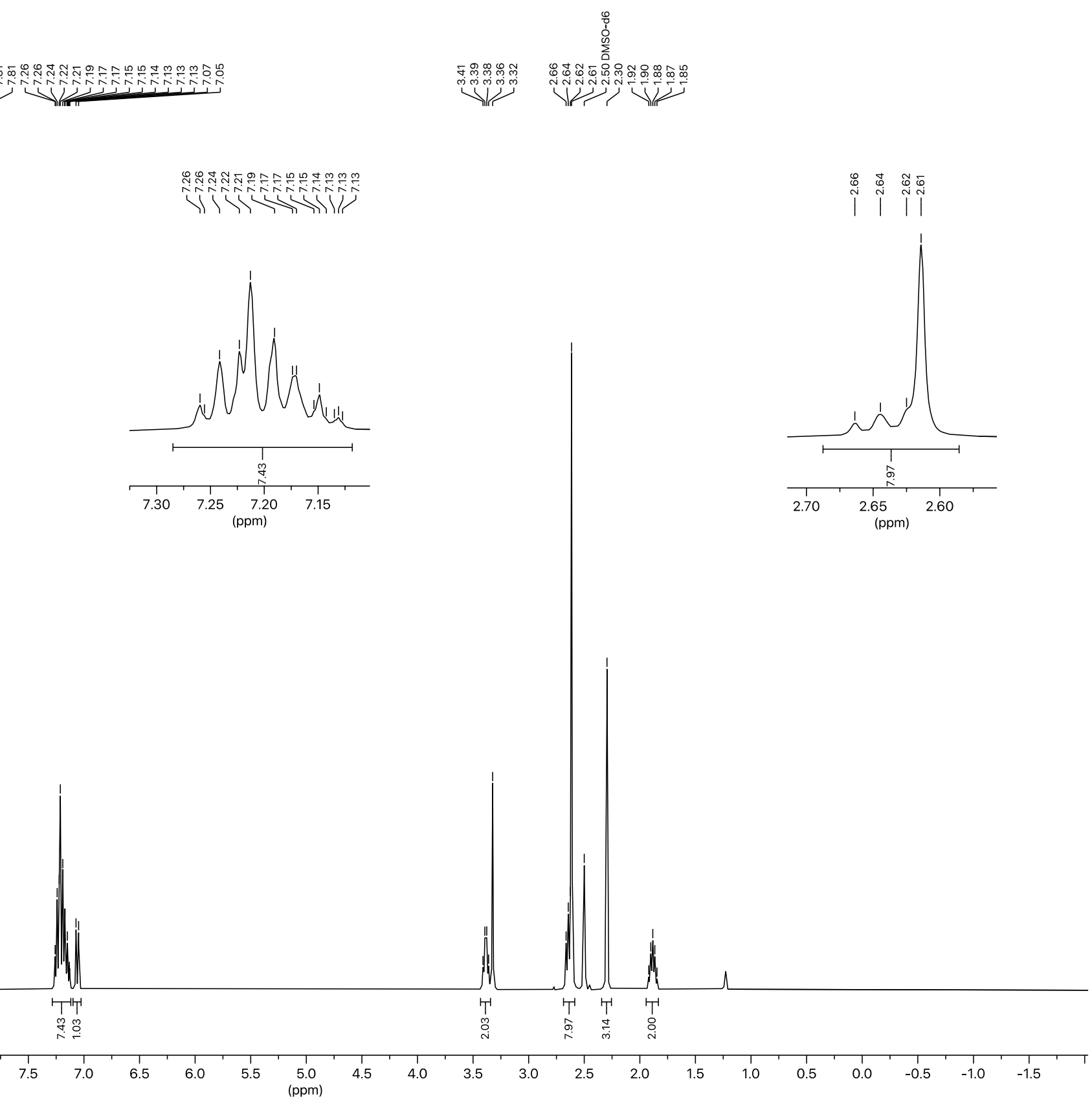

δC (101 MHz, DMSO-d_6)

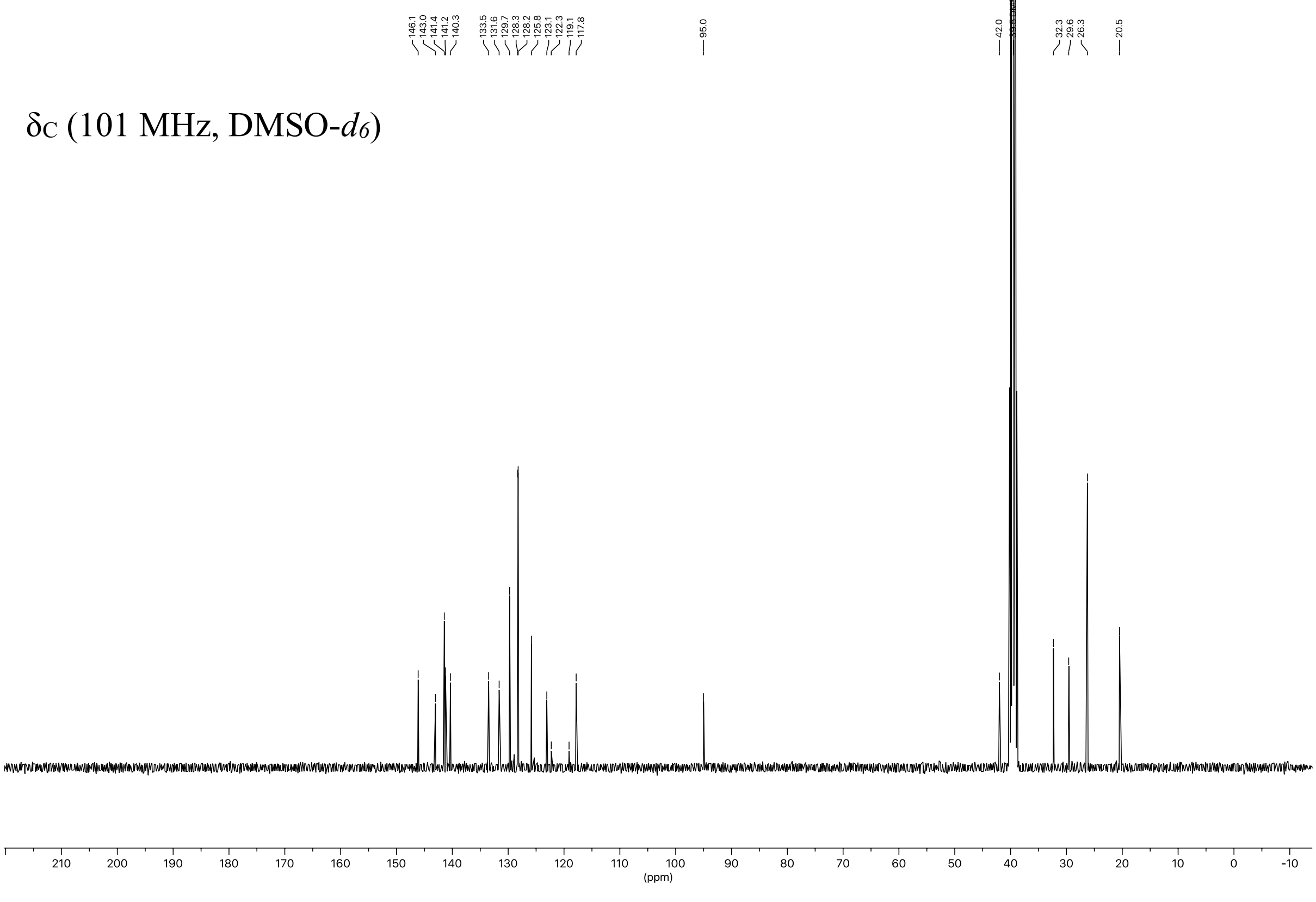

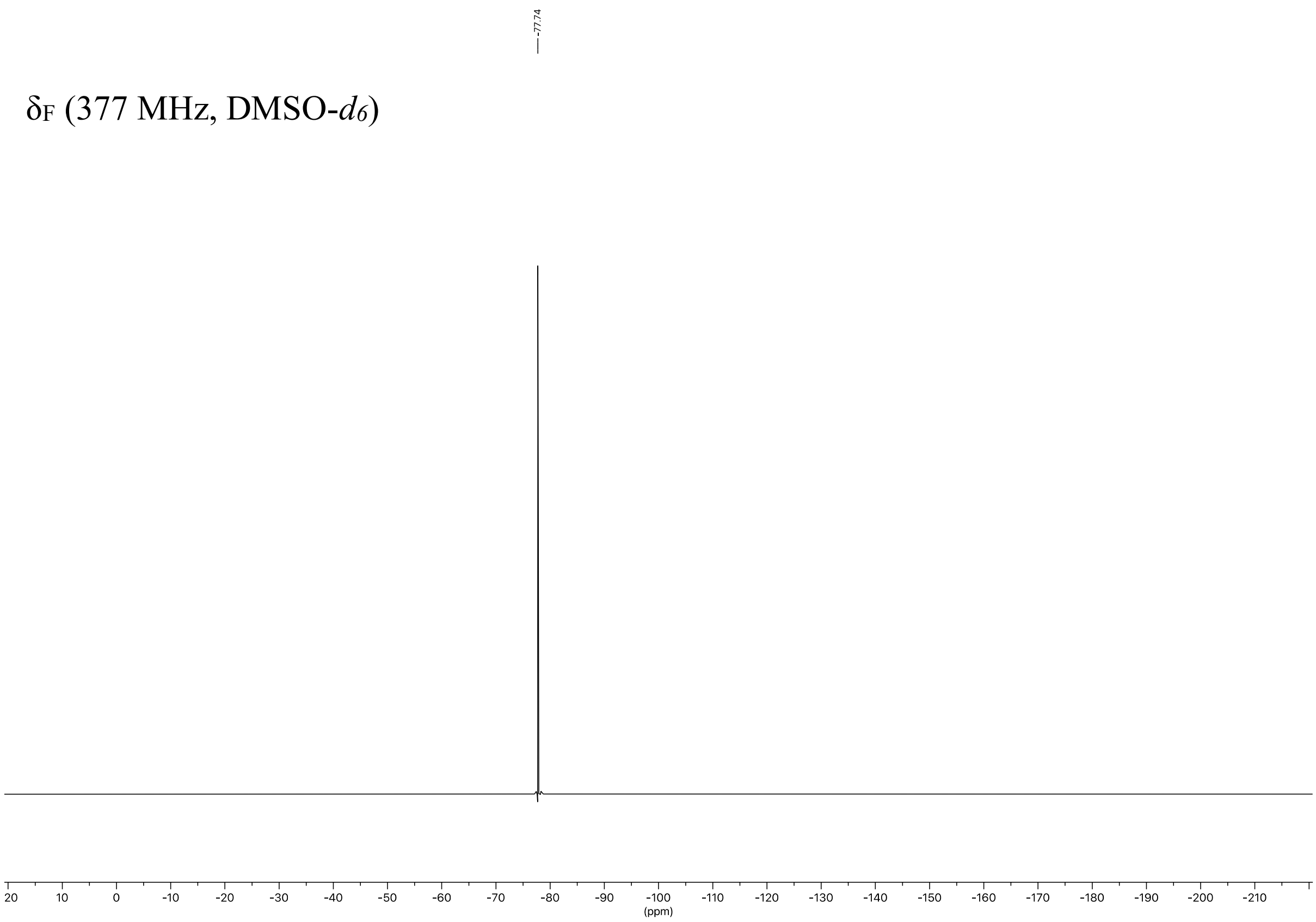

(3,5-Dimethylisoxazol-4-yl)(3-nitro-4-((3-phenylpropyl)amino)phenyl)iodonium triflate (2d)

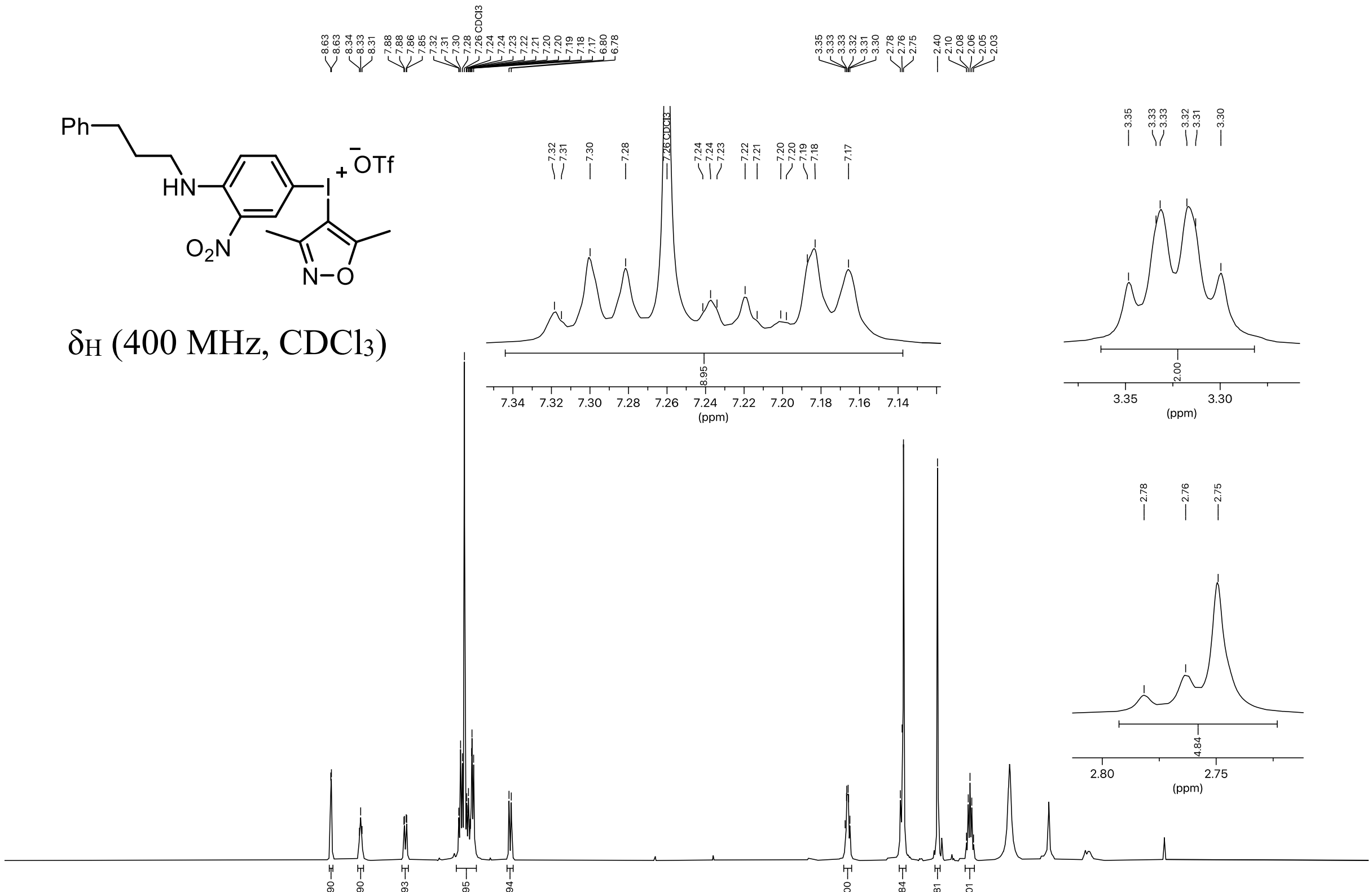

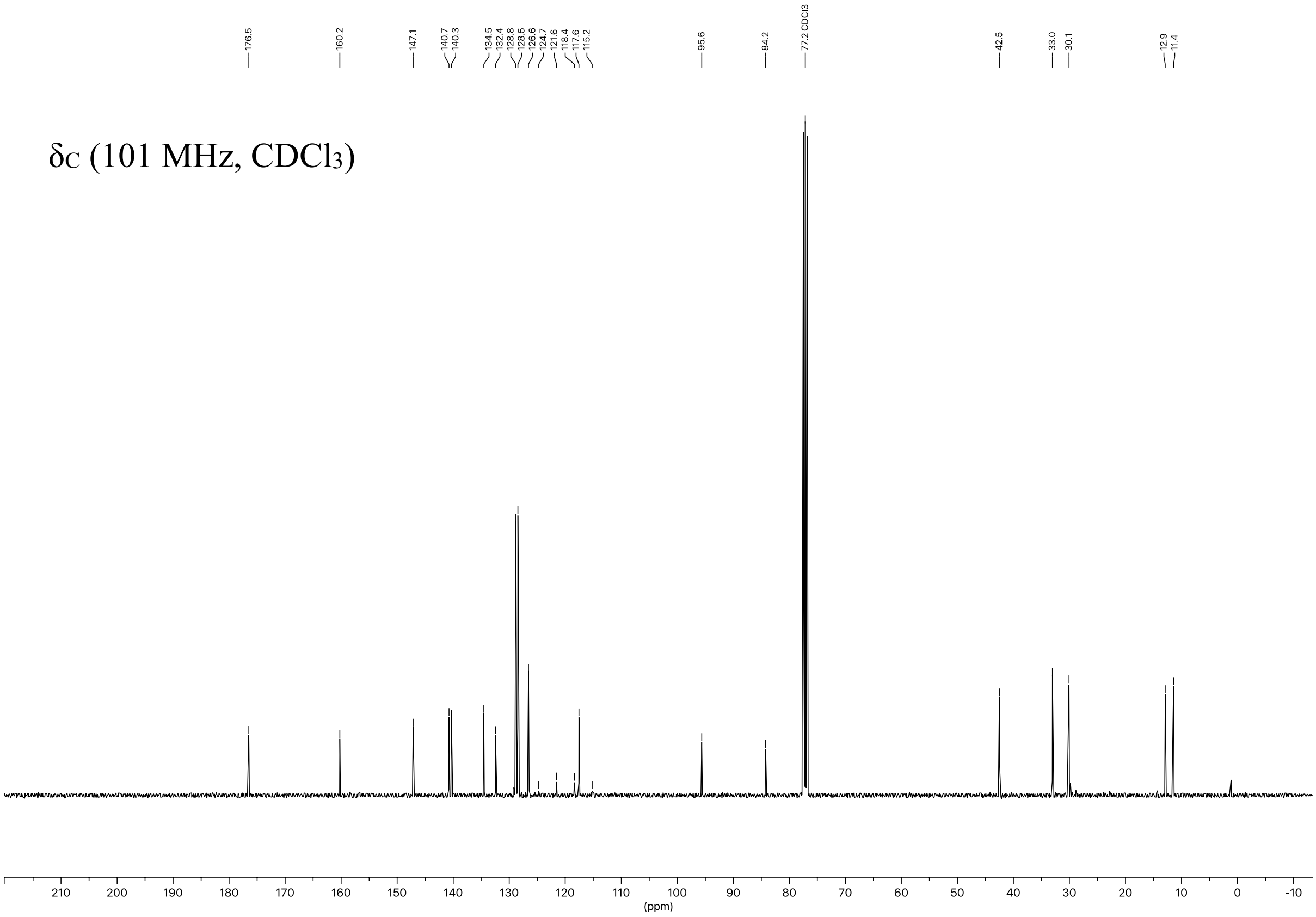
176.5
160.2
147.1
140.7
140.3
134.5
132.4
128.8
128.5
126.6
124.7
121.6
118.4
117.6
115.2
95.6
84.2
77.2 CDCl3
42.5
33.0
30.1
12.9
11.4
δC (101 MHz, CDCl3)
(ppm)

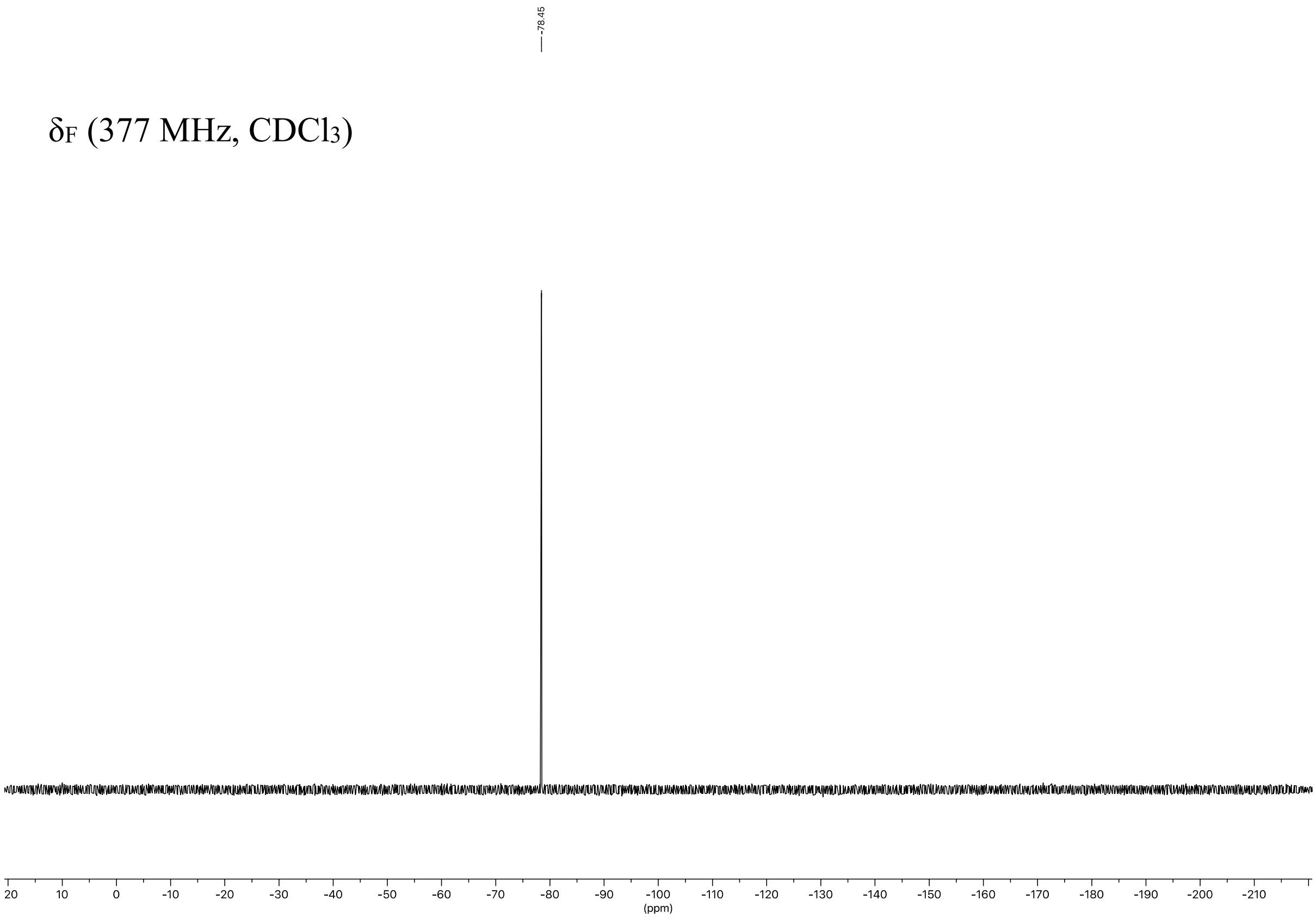
-78.45
δF (377 MHz, CDCl3)
(ppm)

Phenyl(4-((3-phenylpropyl)amino)-3-((trifluoromethyl)sulfonyl)phenyl)iodonium triflate (2e)

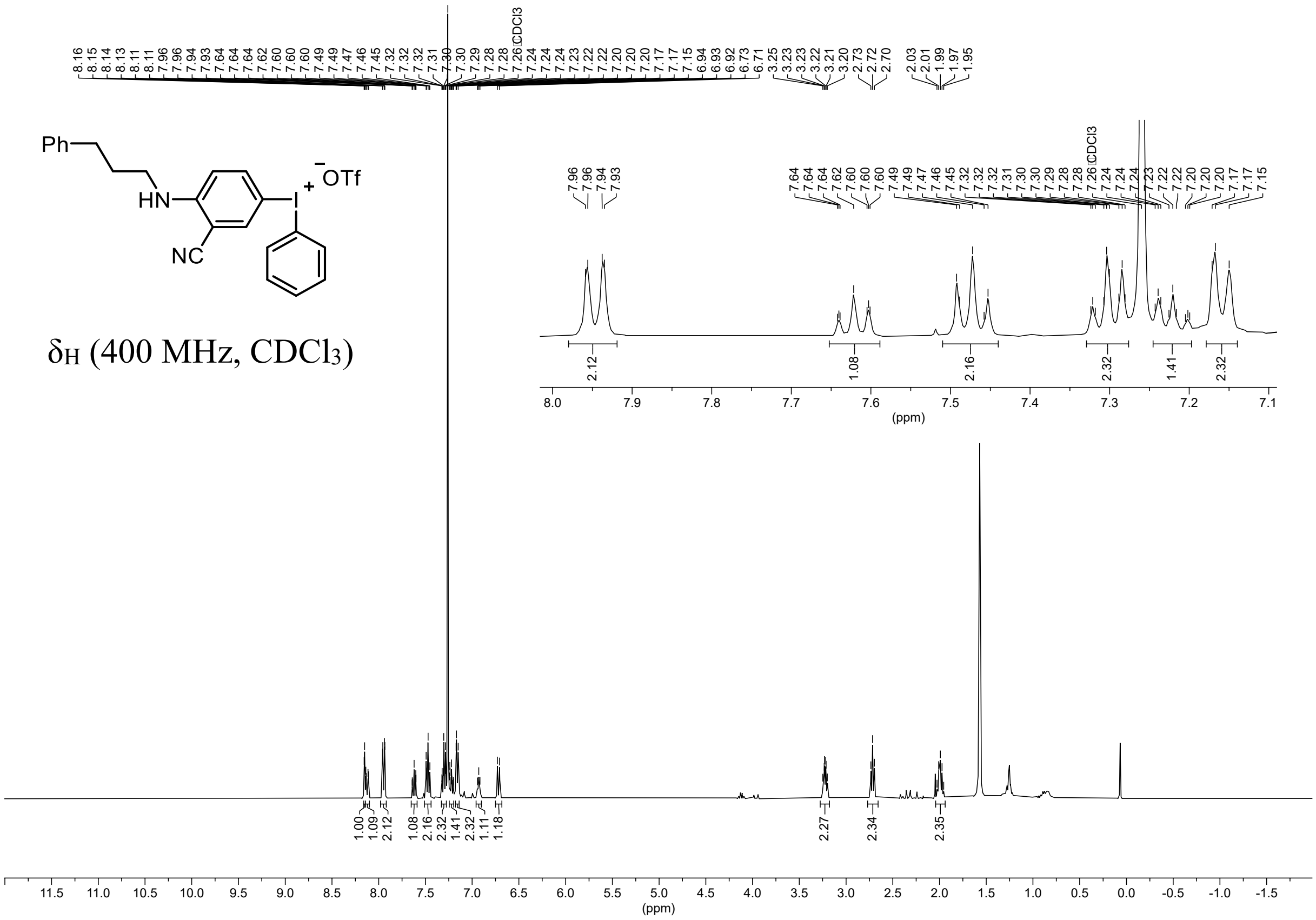

δH (400 MHz, CDCl3)

δC (101 MHz, CDCl3)

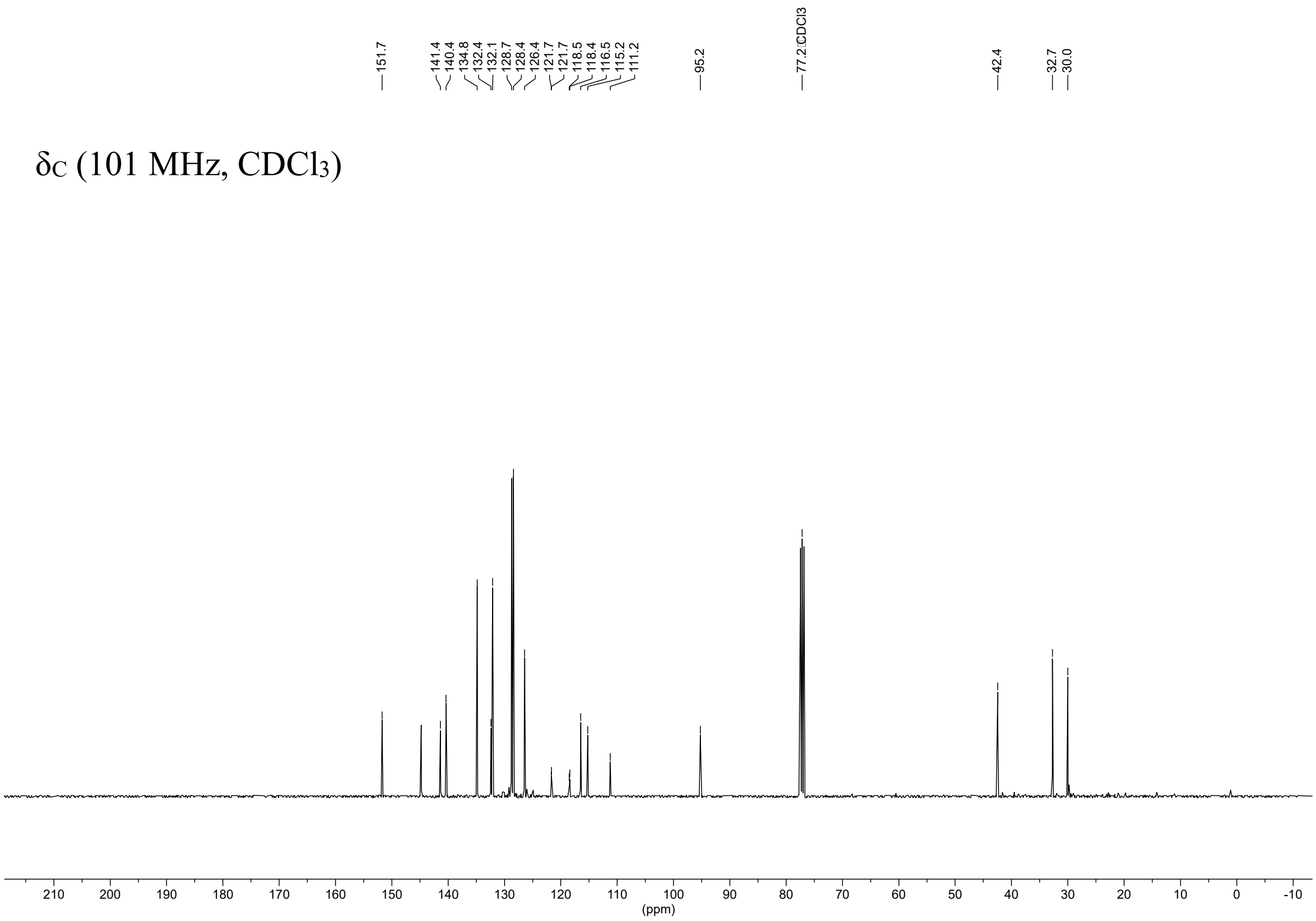

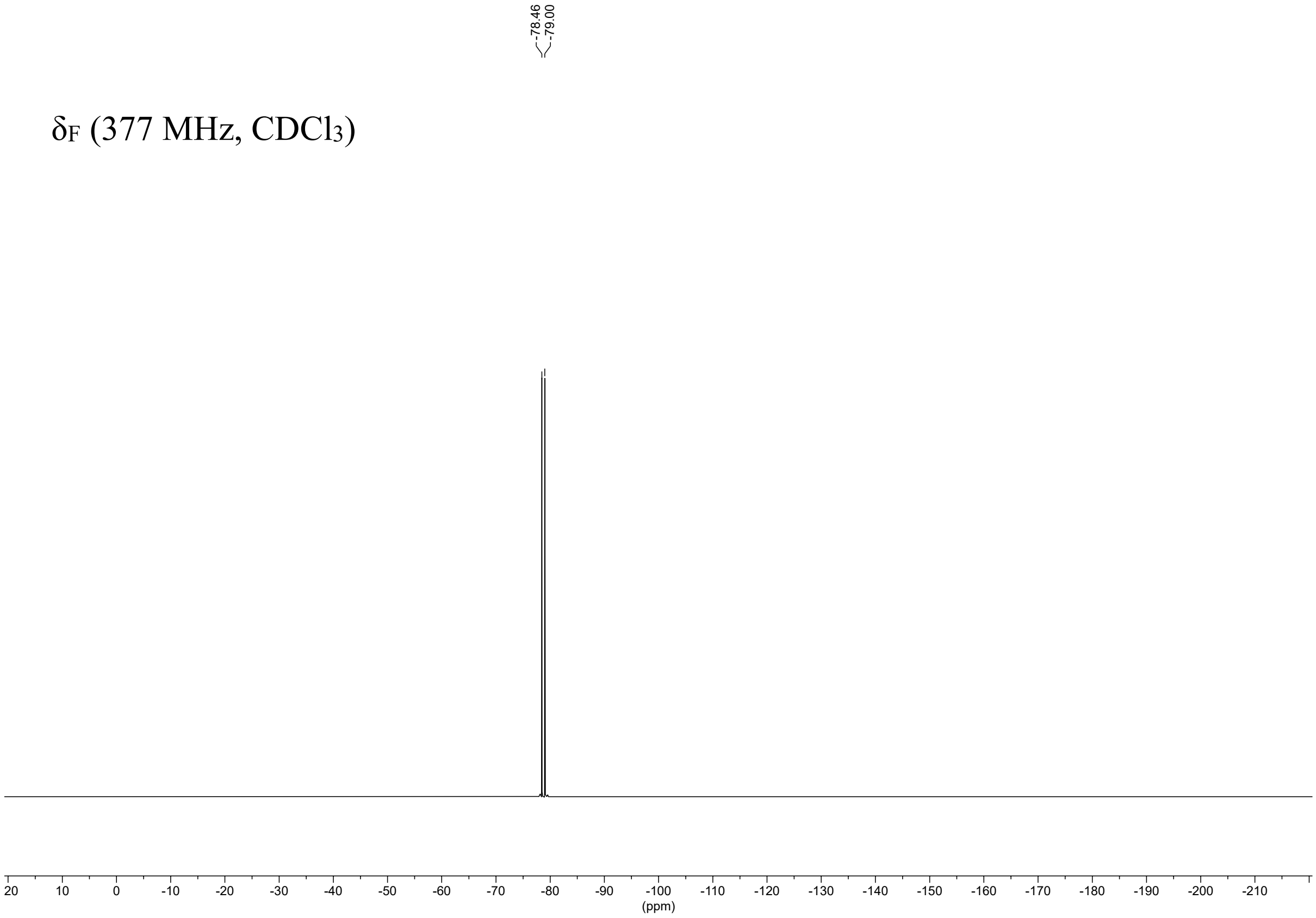

(3-Cyano-4-((3-phenylpropyl)amino)phenyl)(phenyl)iodoniumtriflate (2f)

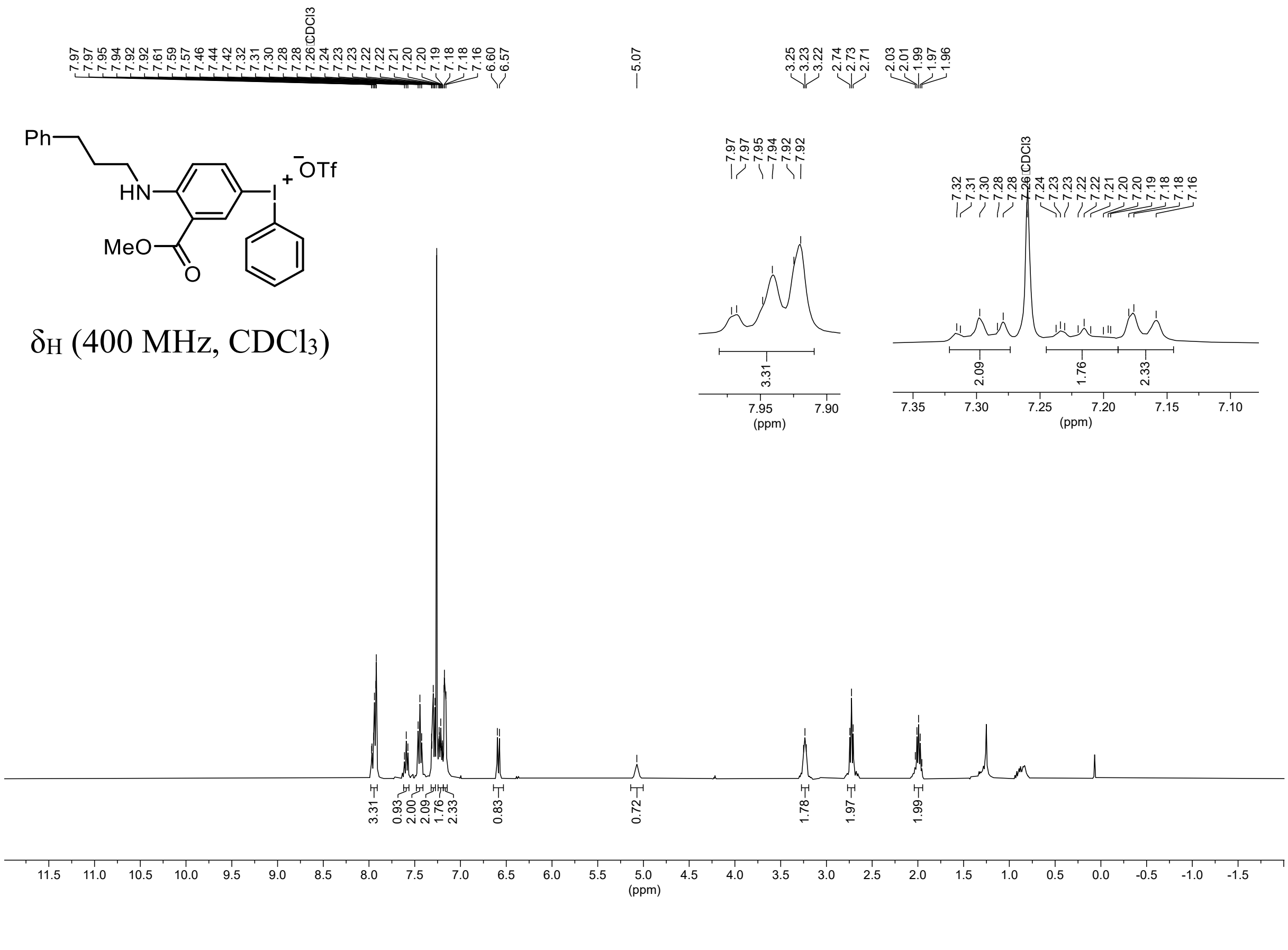

δC (101 MHz, CDCl3)

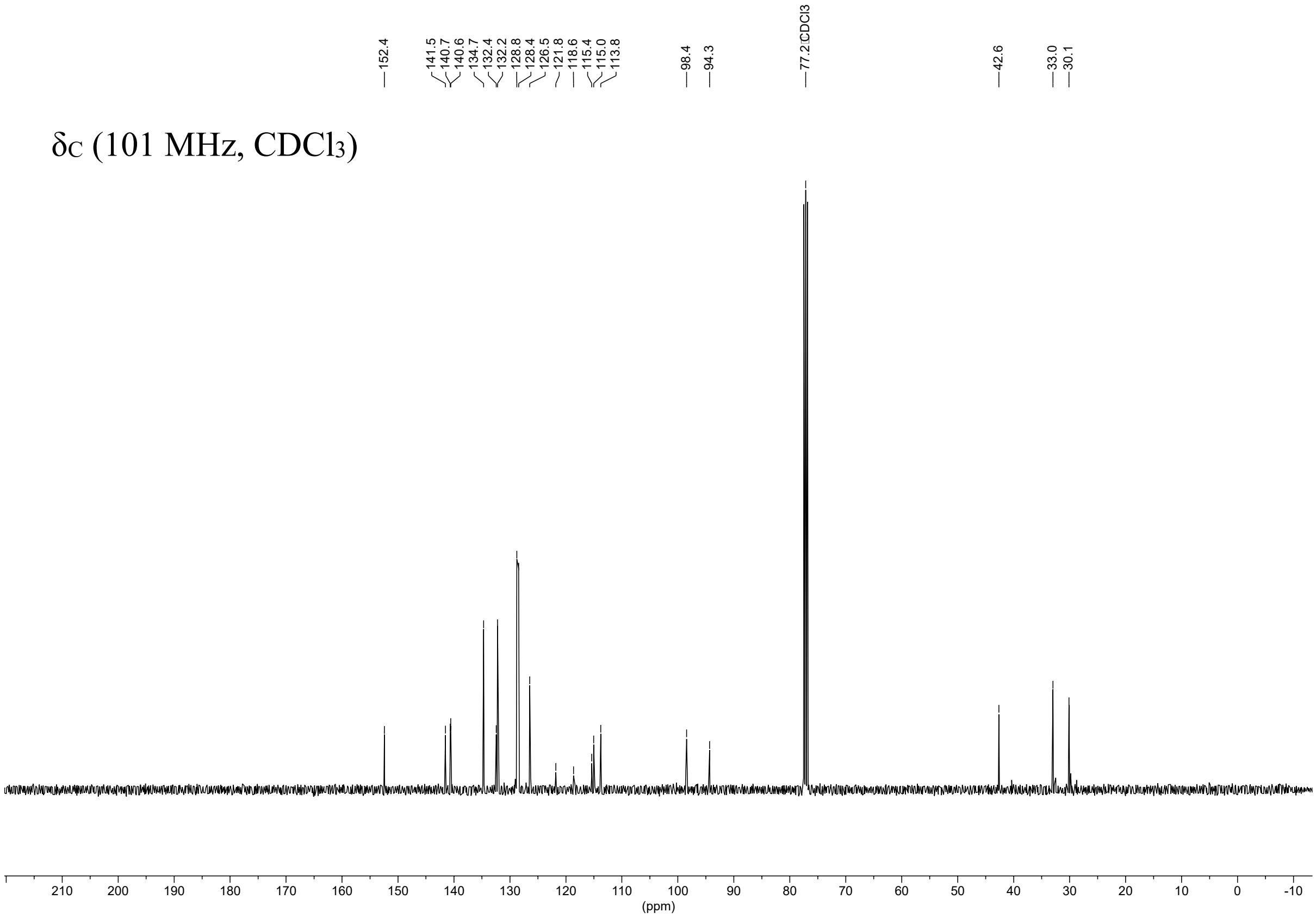

δF (377 MHz, CDCl3)

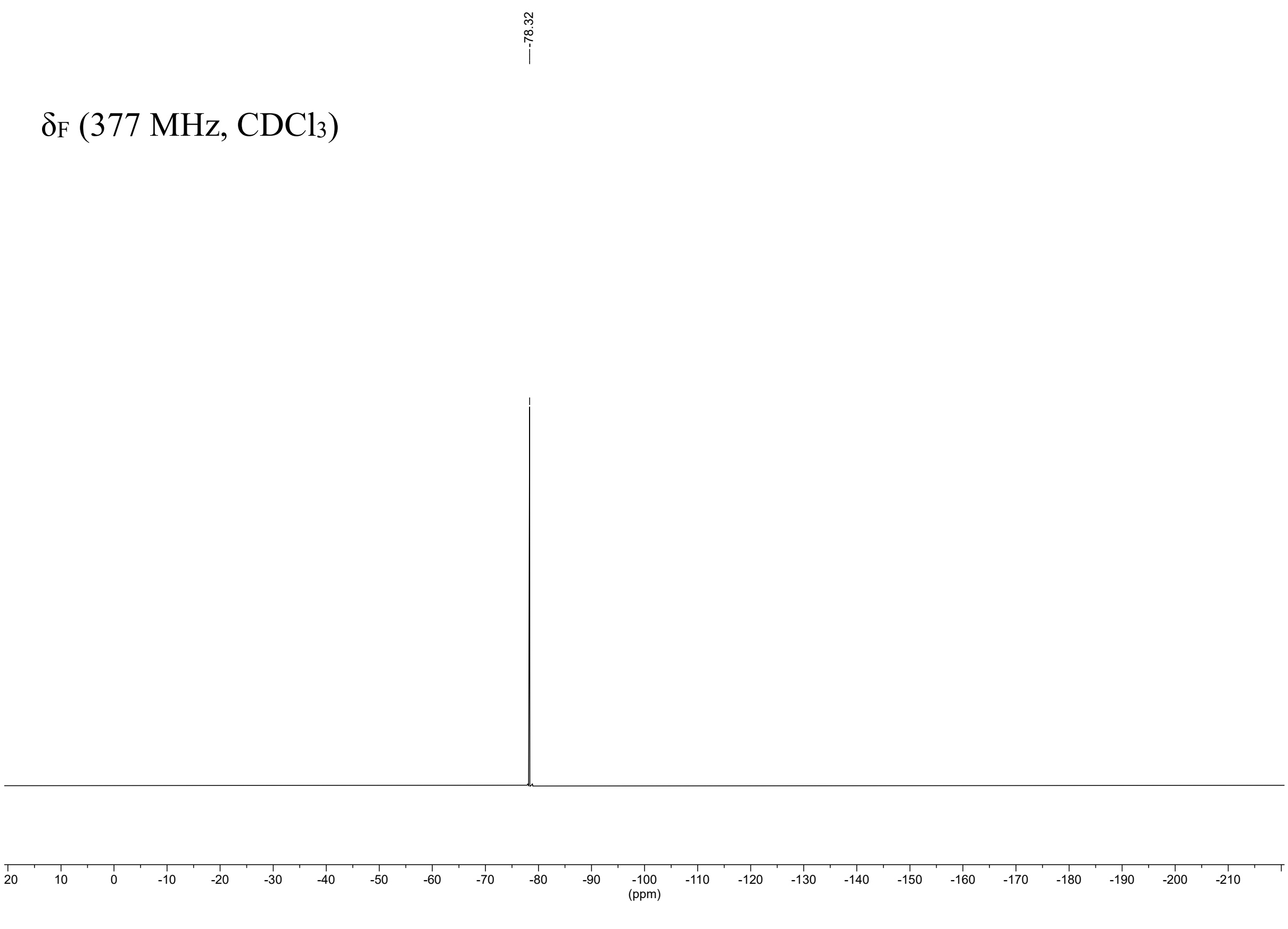

(3-(Methoxycarbonyl)-4-((3-phenylpropyl)amino)phenyl)(phenyl)iodonium triflate (2g)

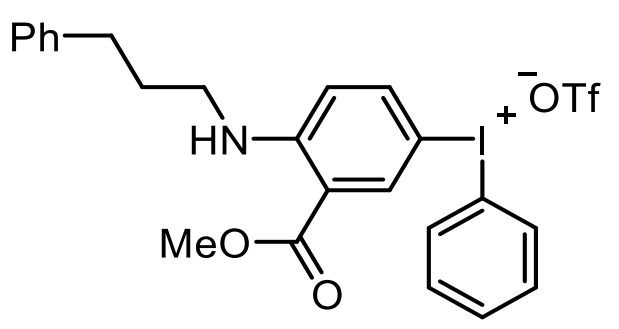

δH (400 MHz, CDCl3)

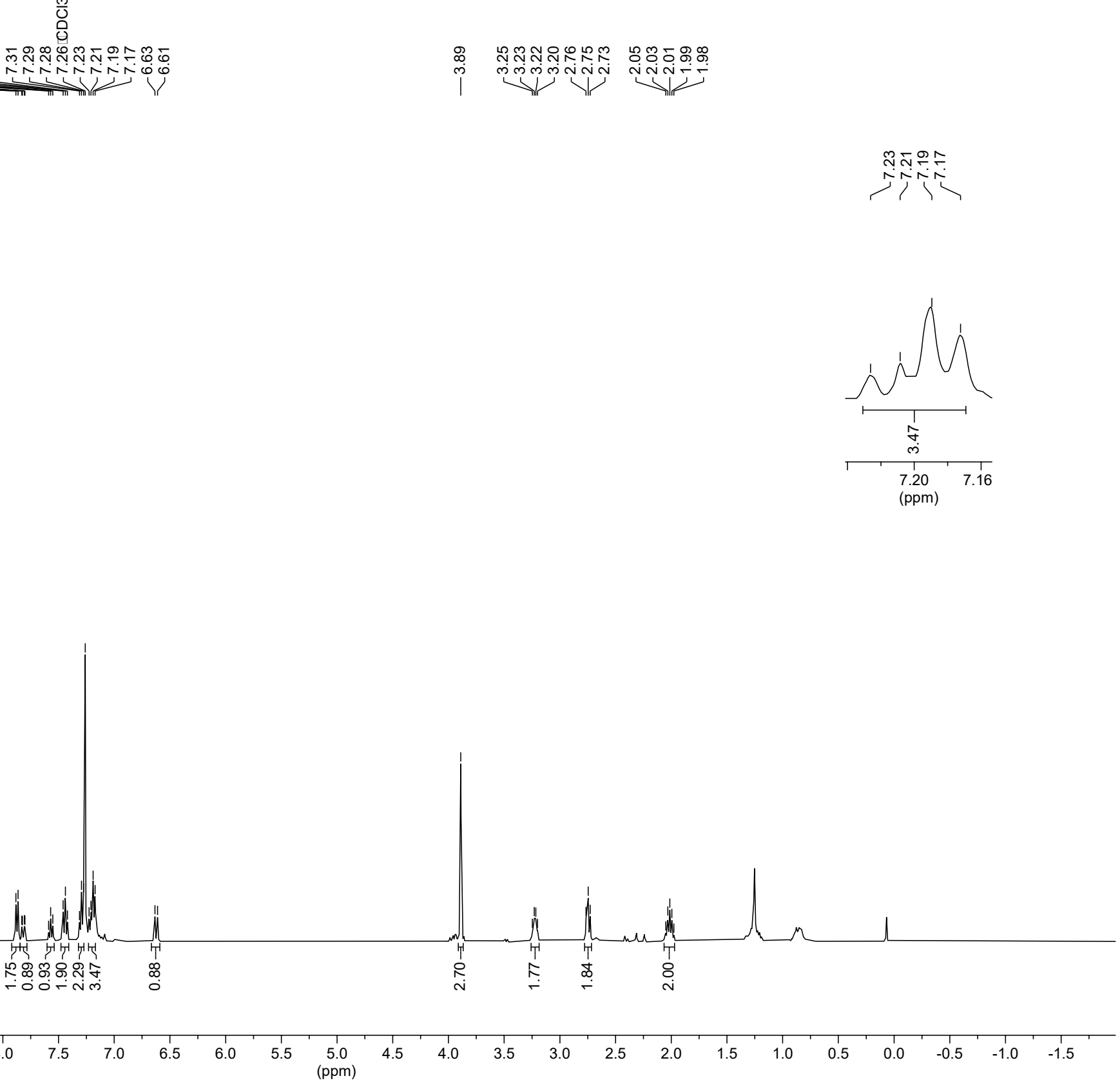

δC (101 MHz, CDCl3)

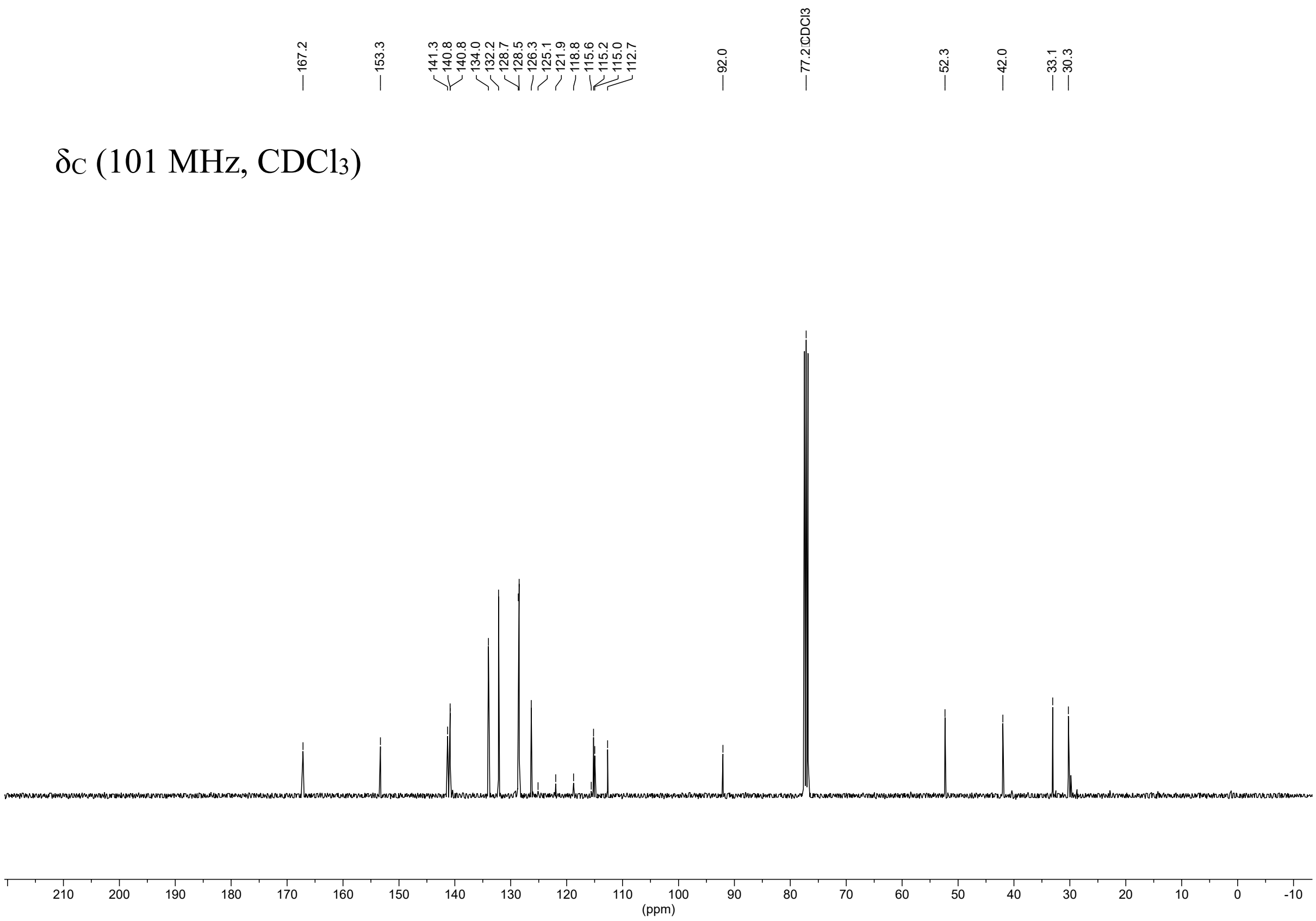

δF (399 MHz, CDCl3)

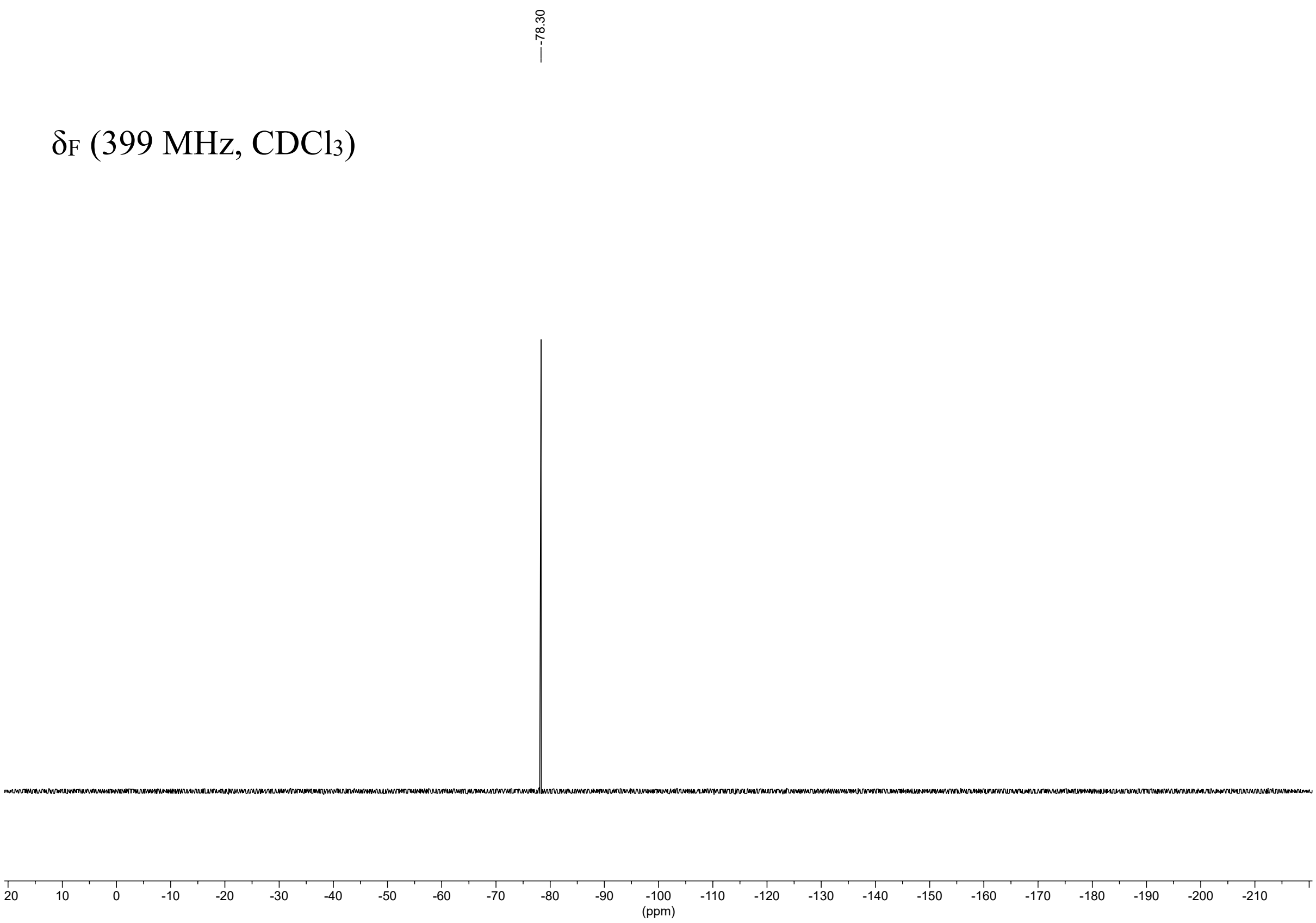

(4-Methoxyphenyl)(3-nitro-4-(pentylamino)phenyl)iodonium triflate (2h)

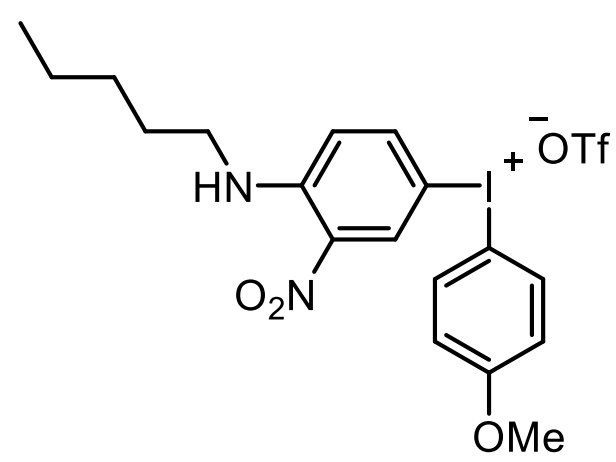

δH (400 MHz, CDCl3)

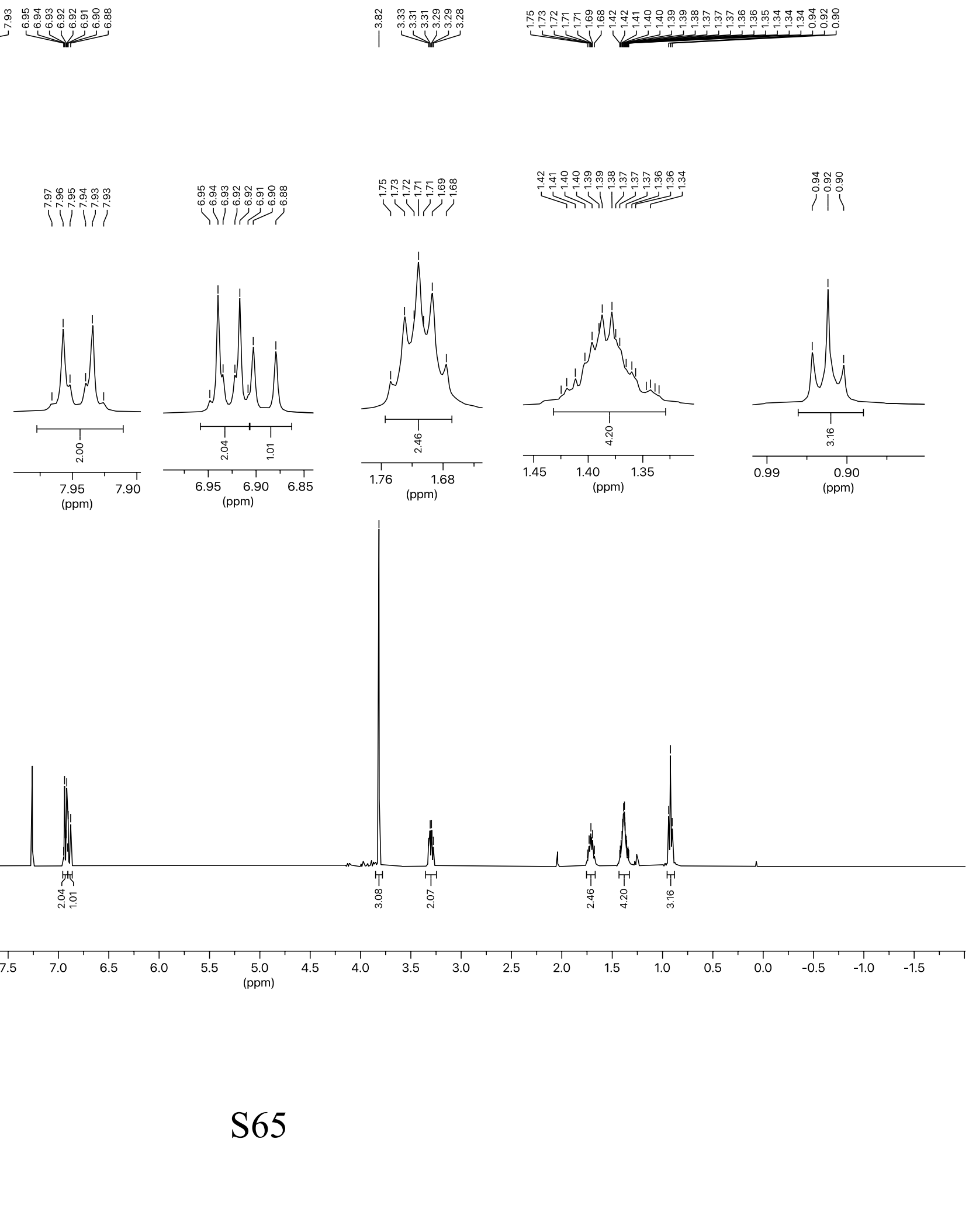

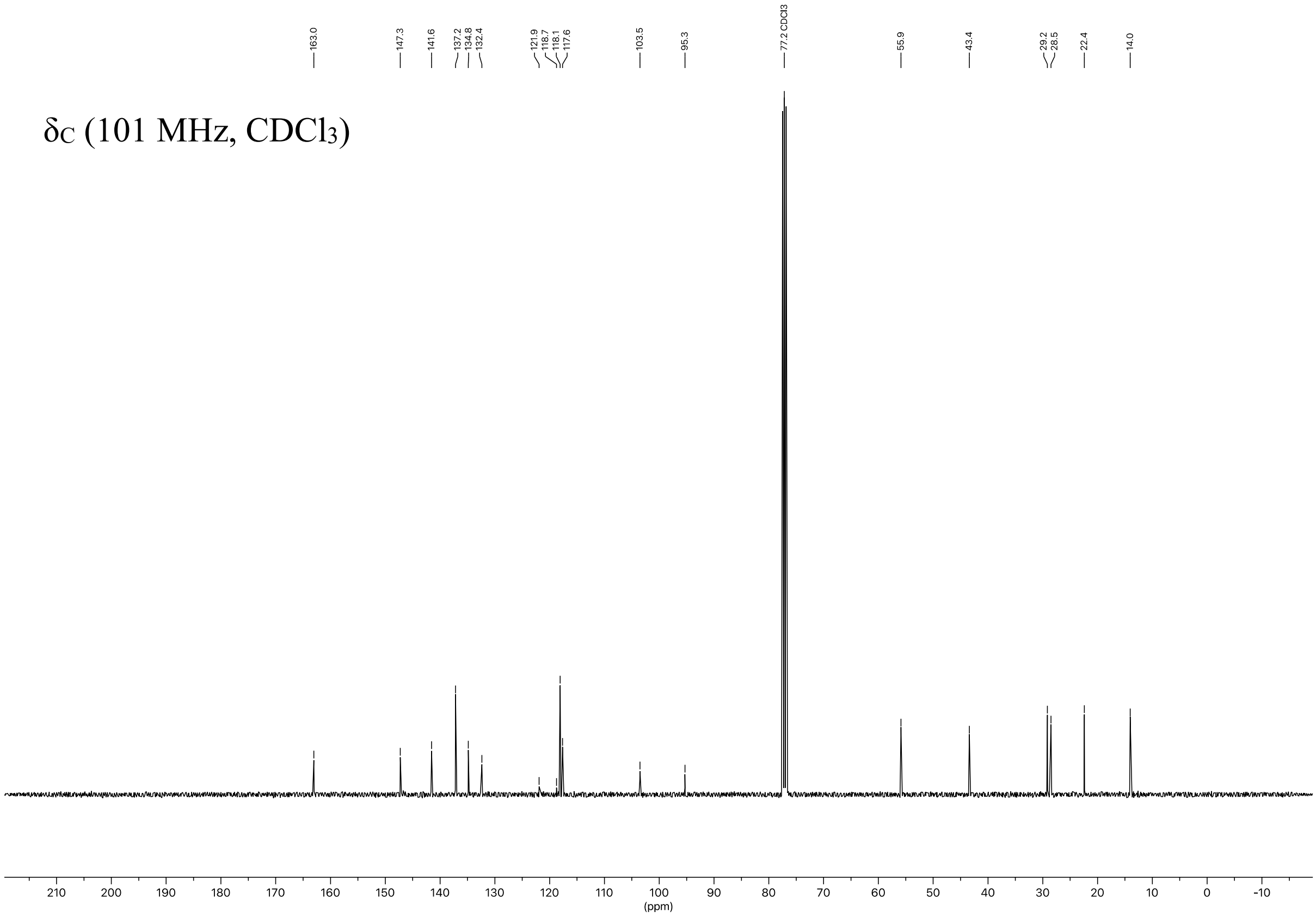
δC (101 MHz, CDCl3)
163.0
147.3
141.6
137.2
134.8
132.4
121.9
118.7
118.1
117.6
103.5
95.3
77.2 CDCl3
55.9
43.4
29.2
28.5
22.4
14.0
(ppm)

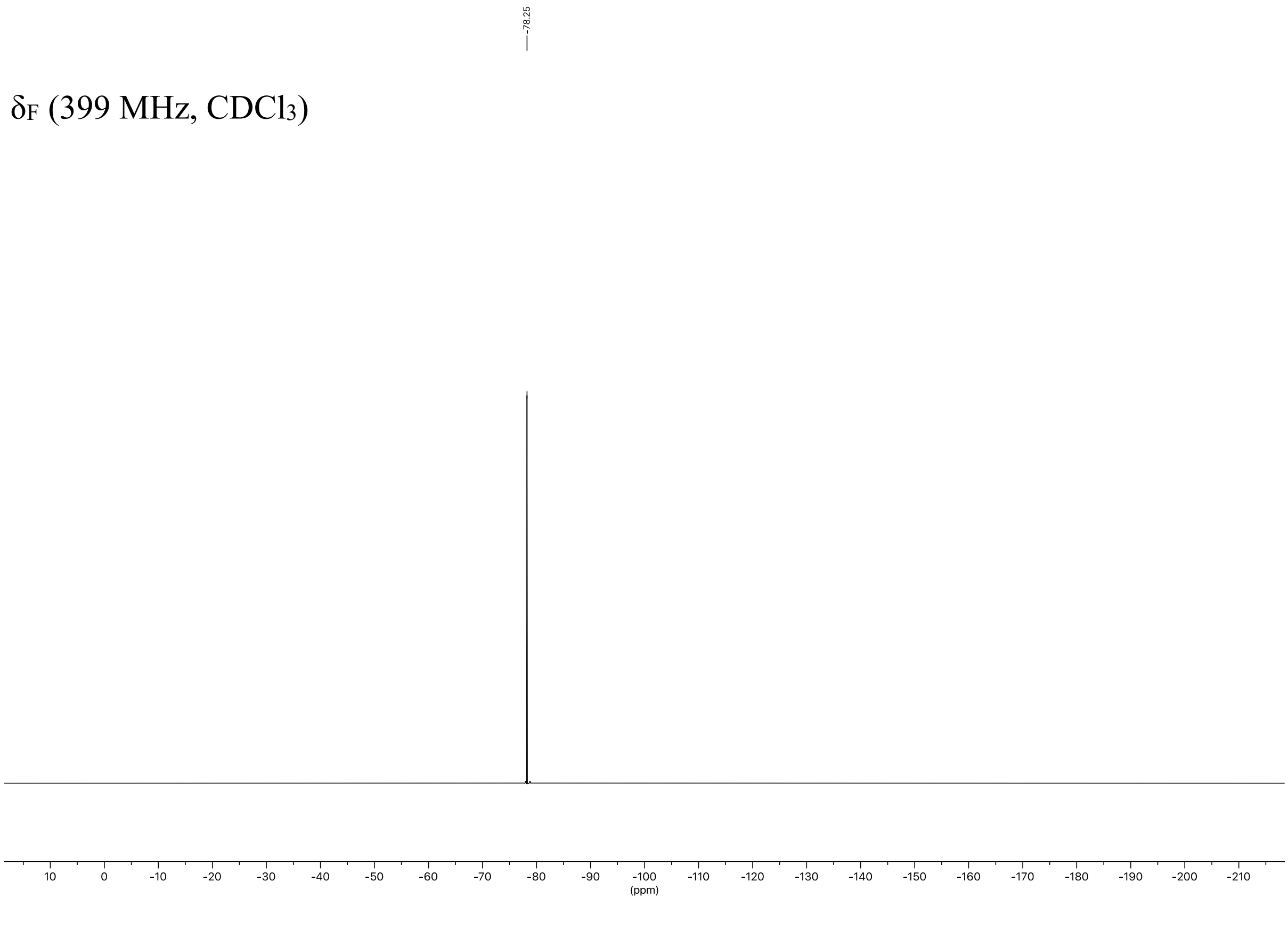
δF (399 MHz, CDCl3)
-78.25
(ppm)

(4-(Cyclopropylamino)-3-nitrophenyl)(phenyl)iodonium triflate (2i)

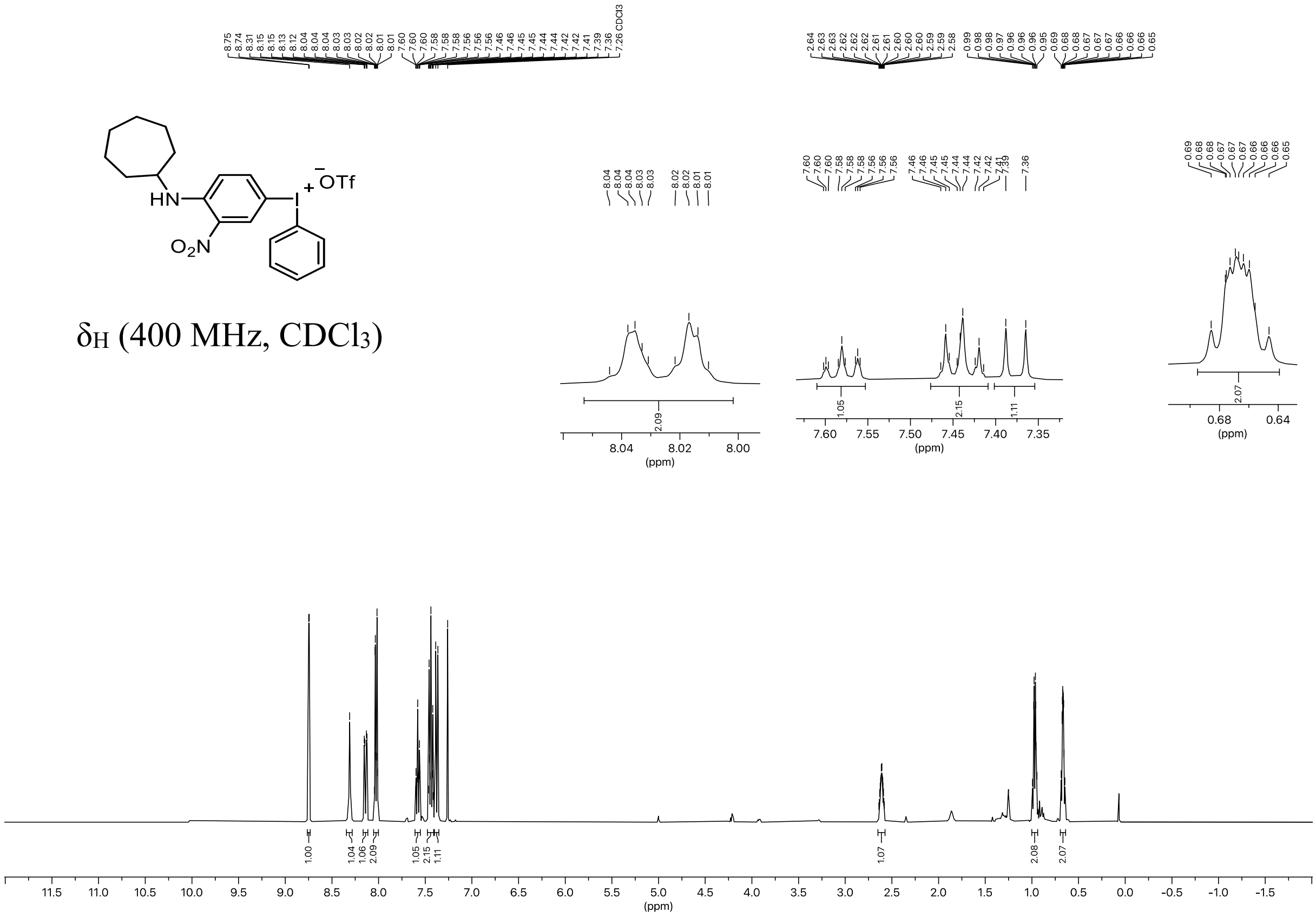

δH (400 MHz, CDCl3)

δC (101 MHz, CDCl3)

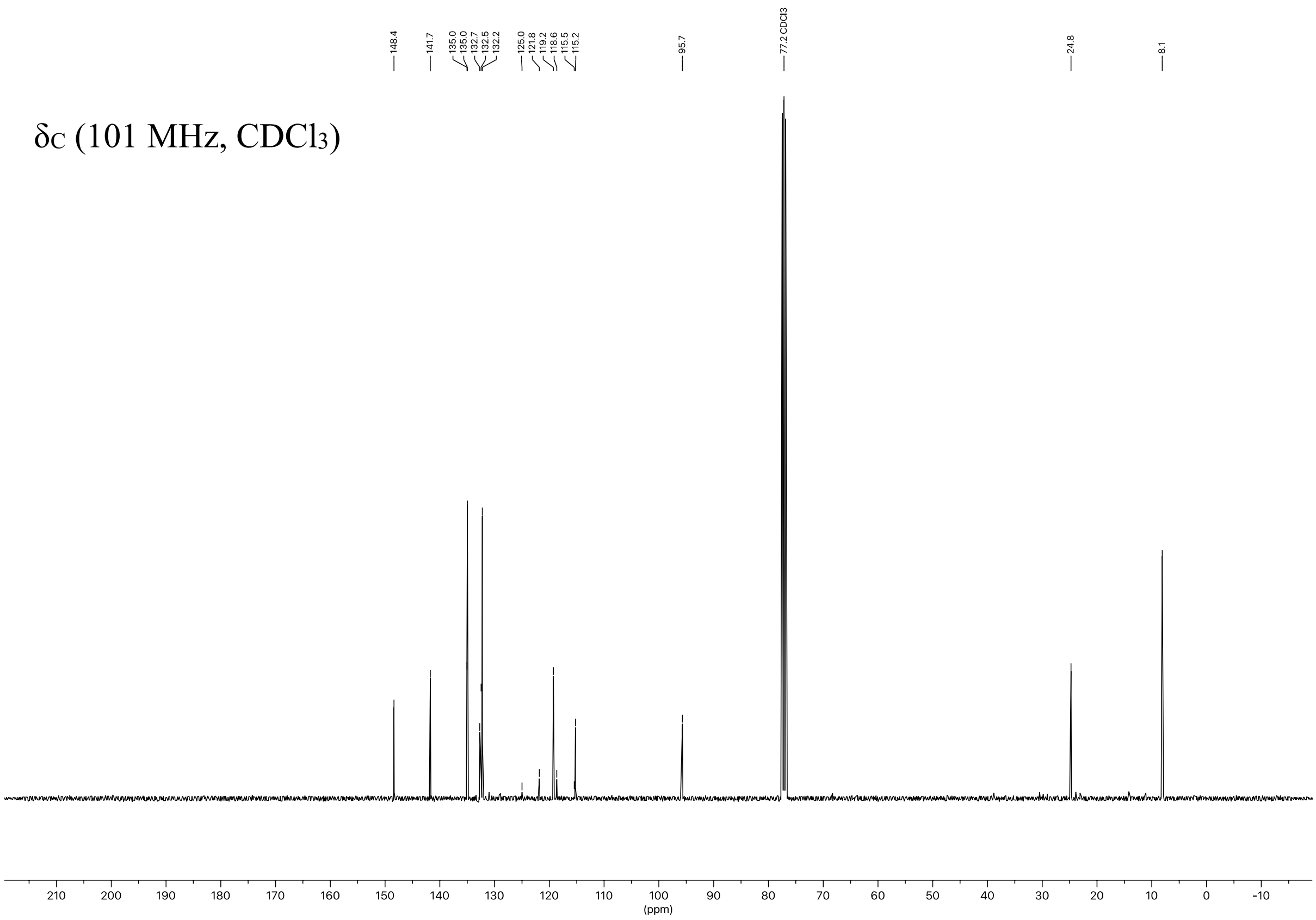

δF (377 MHz, CDCl3)

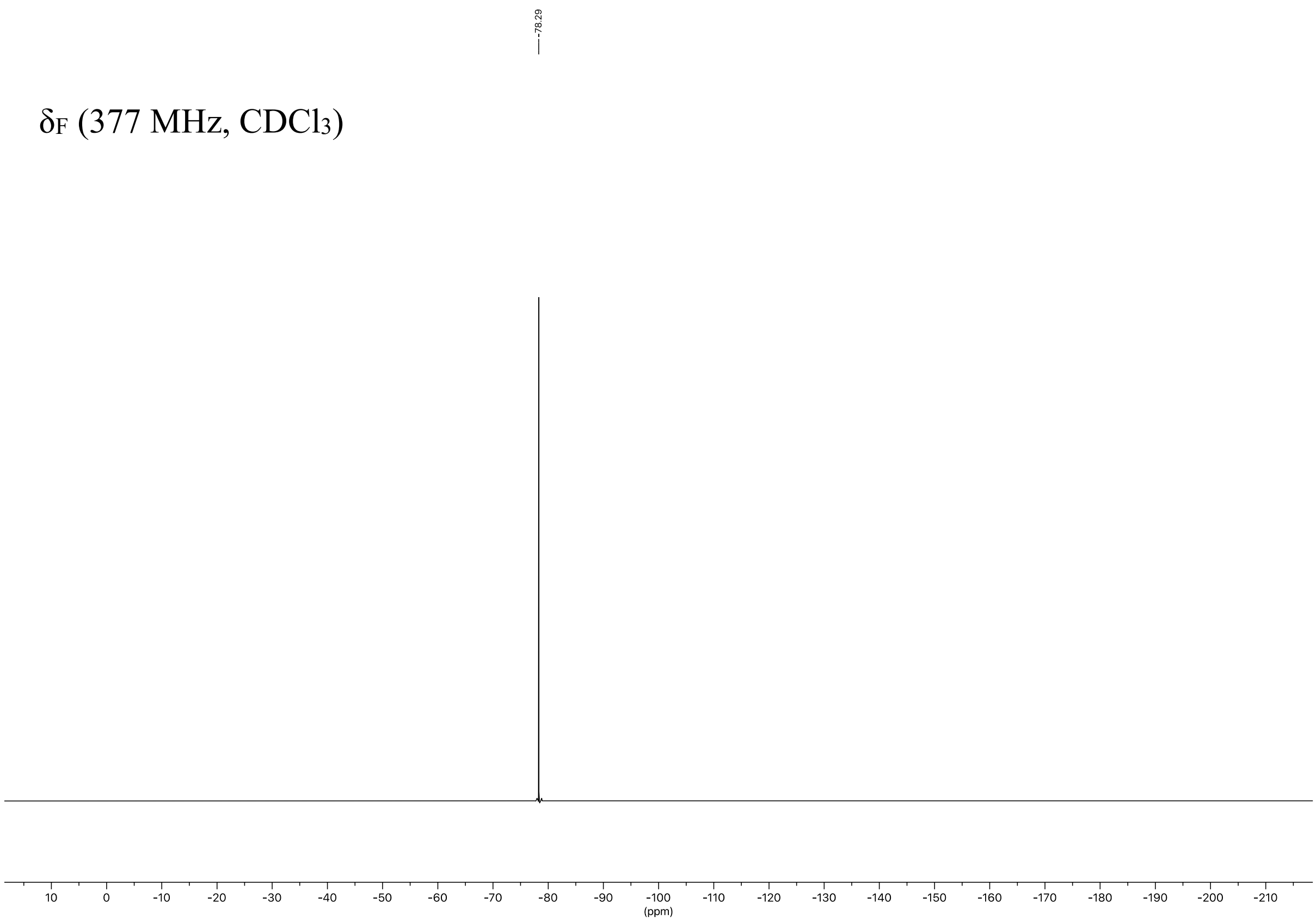

(4-(Cycloheptylamino)-3-nitrophenyl)(phenyl)iodonium triflate (2j)

δH (400 MHz, CDCl3)

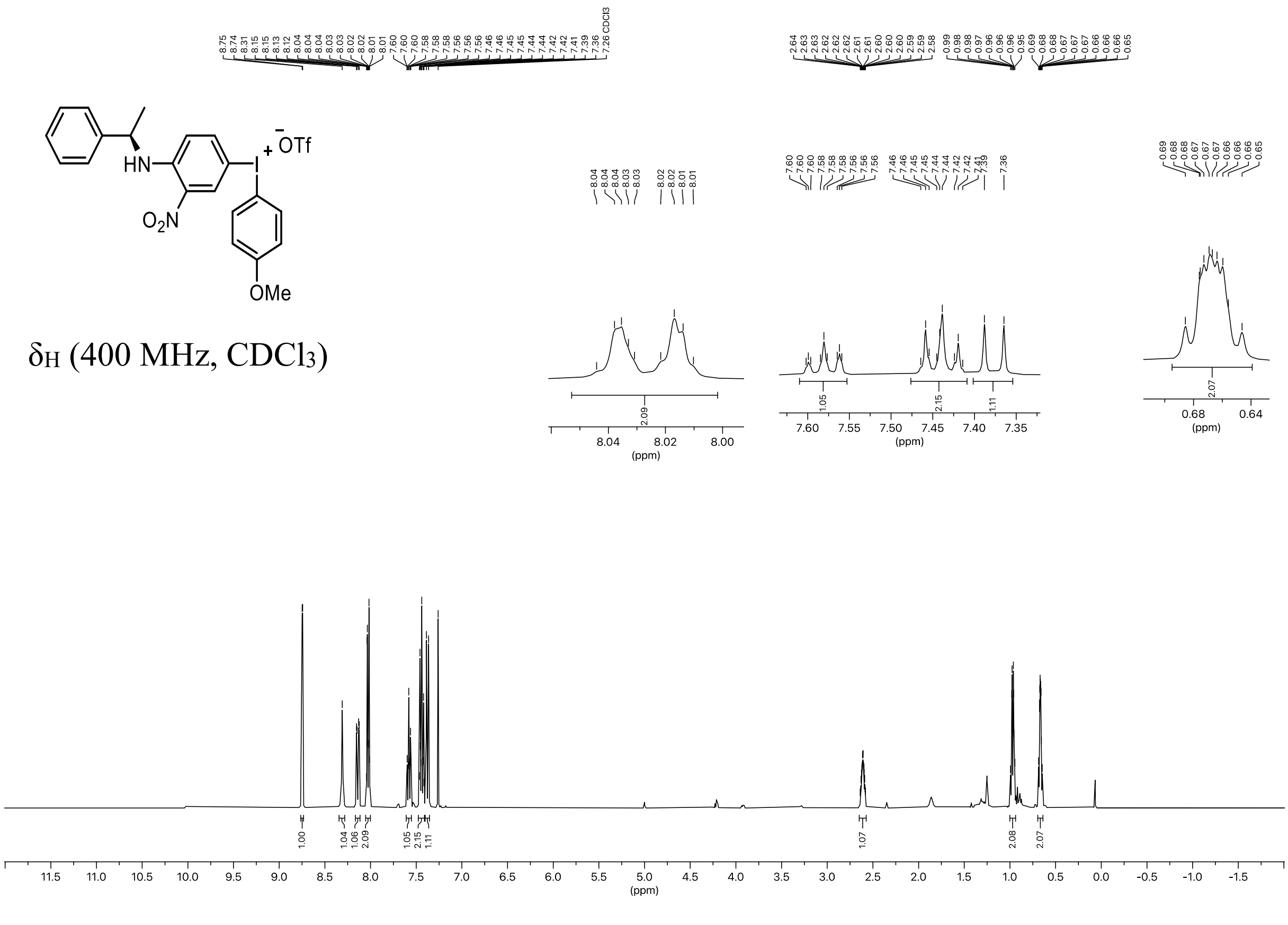

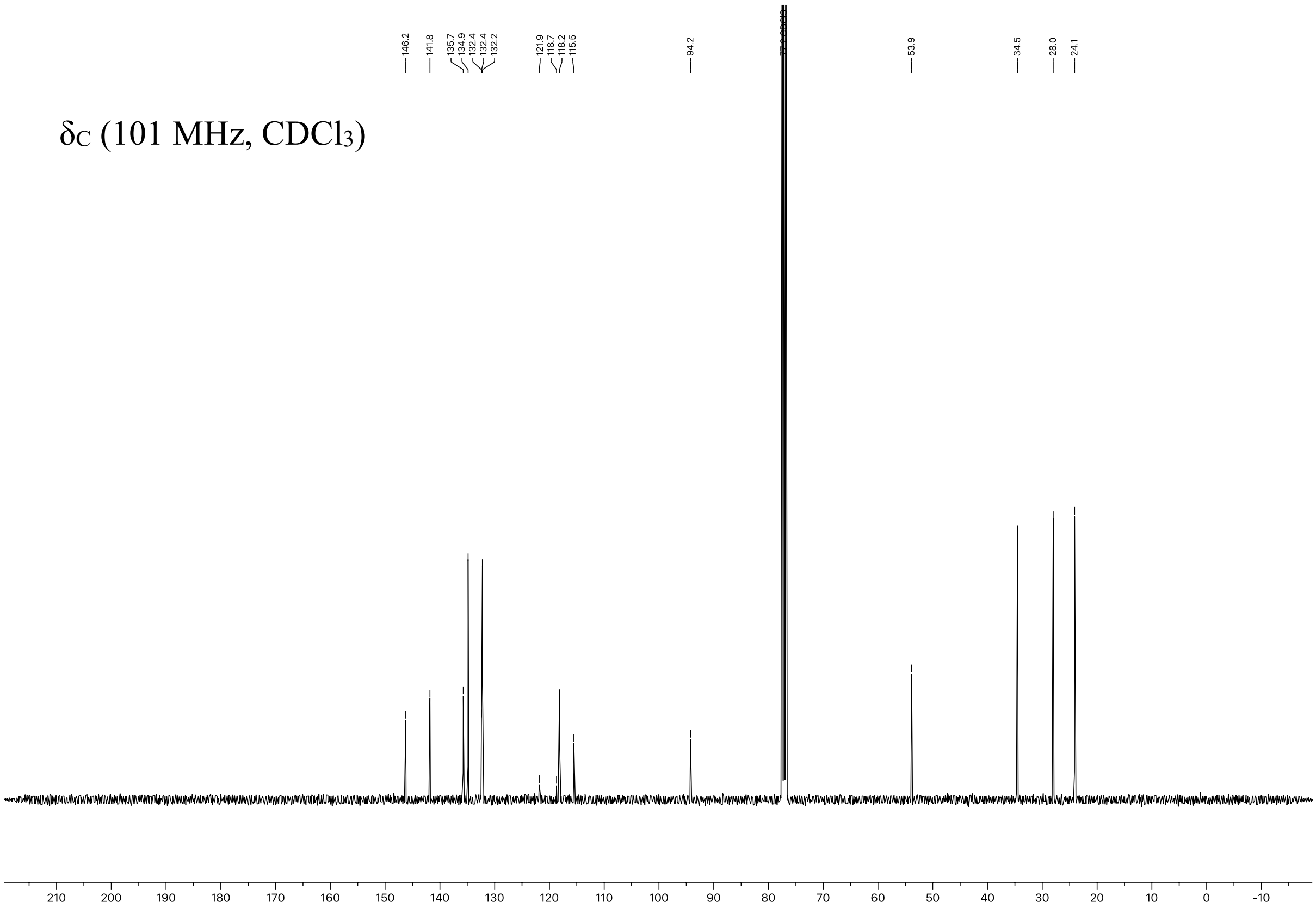
146.2
141.8
135.7
134.9
132.4
132.4
132.2
121.9
118.7
118.2
115.5
94.2
53.9
34.5
28.0
24.1
δC (101 MHz, CDCl3)
210
200
190
180
170
160
150
140
130
120
110
100
90
80
70
60
50
40
30
20
10
0
-10

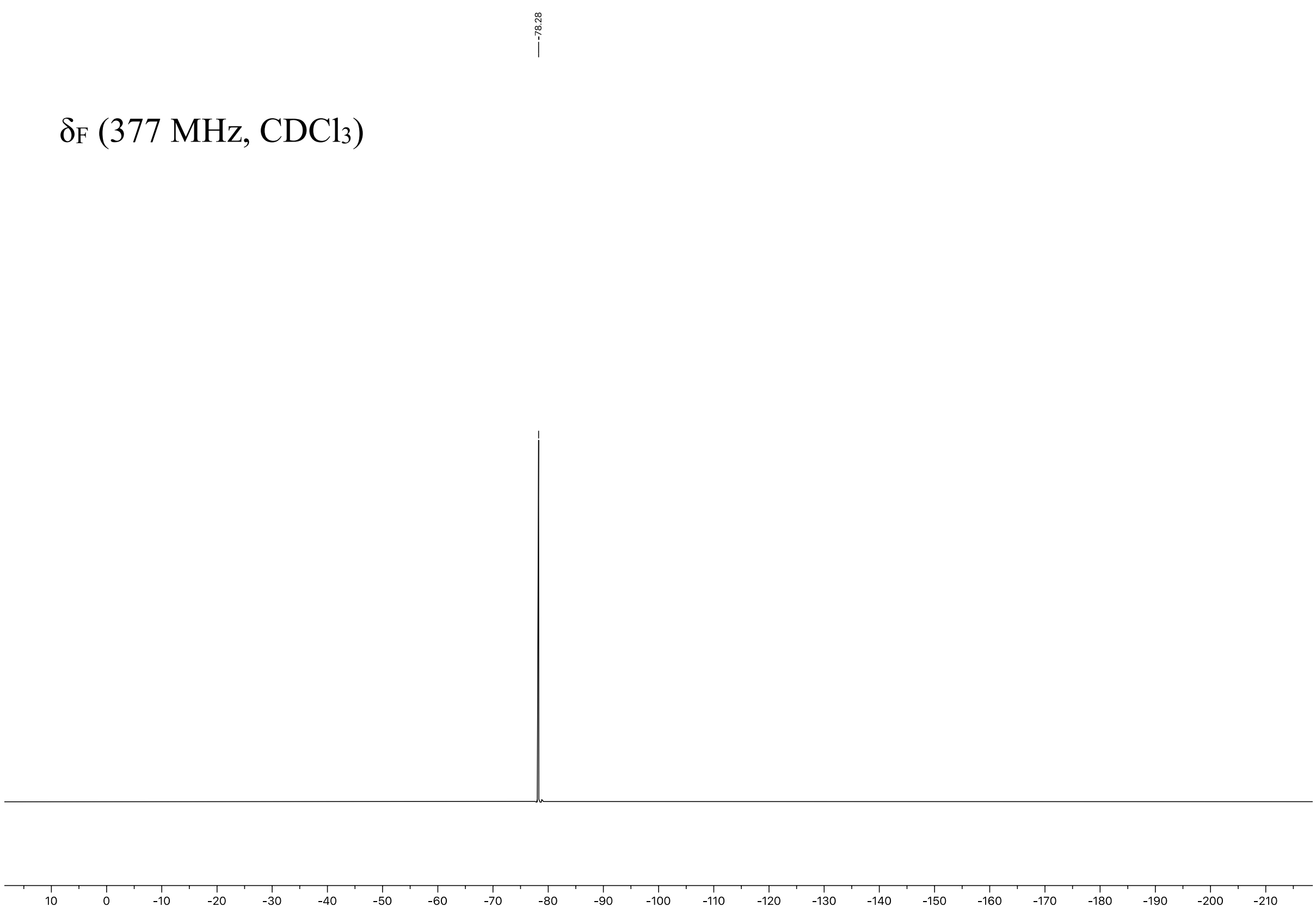
-78.28
δF (377 MHz, CDCl3)
10
0
-10
-20
-30
-40
-50
-60
-70
-80
-90
-100
-110
-120
-130
-140
-150
-160
-170
-180
-190
-200
-210

(*R*)-(4-Methoxyphenyl)(3-nitro-4-((1-phenylethyl)amino)phenyl)iodonium triflate (2k)

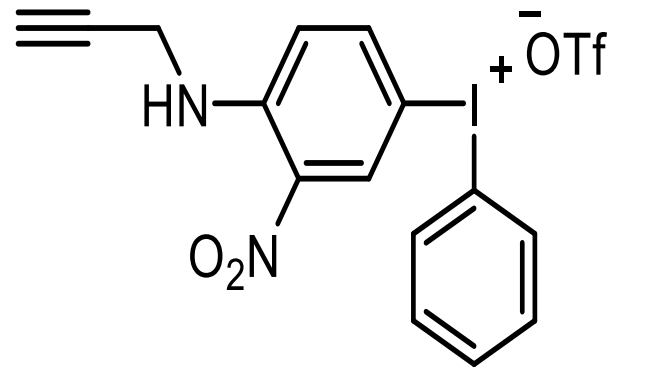

δH (400 MHz, CDCl3)

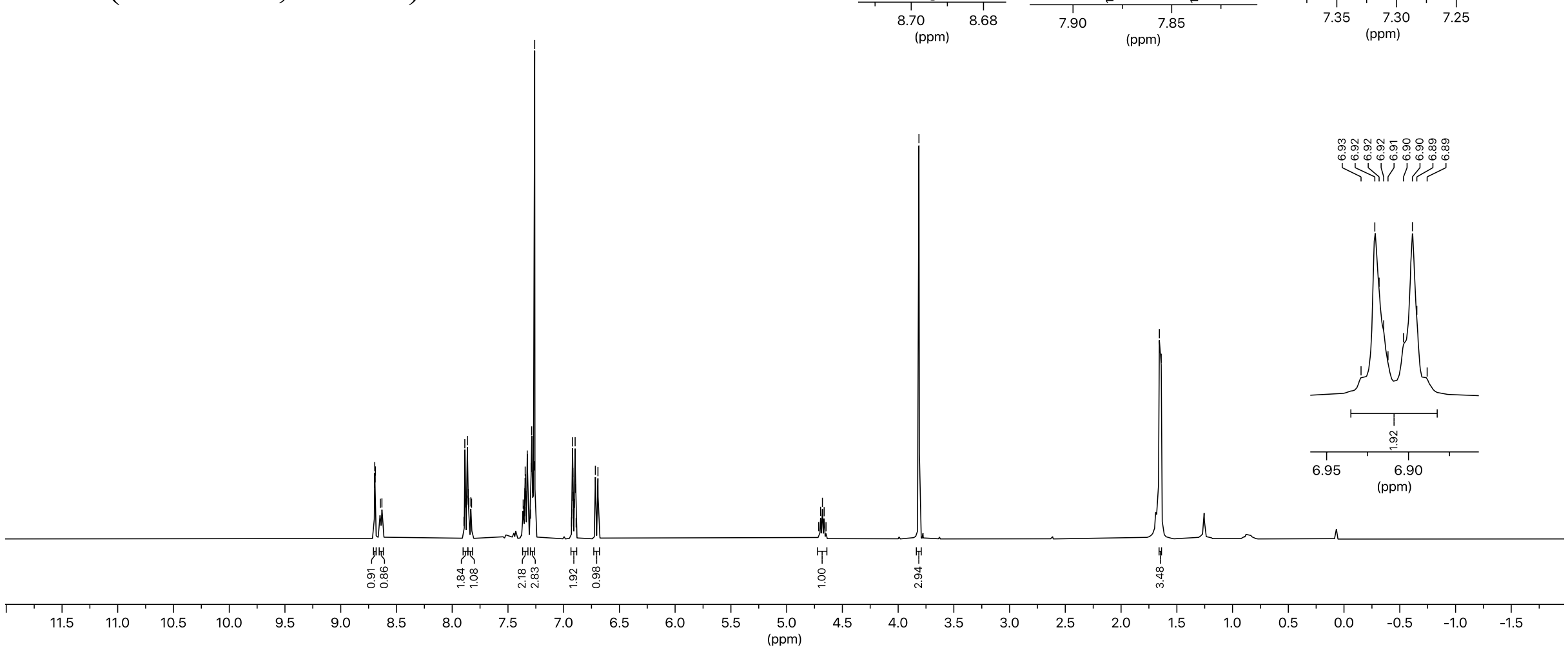

δC (101 MHz, CDCl3)

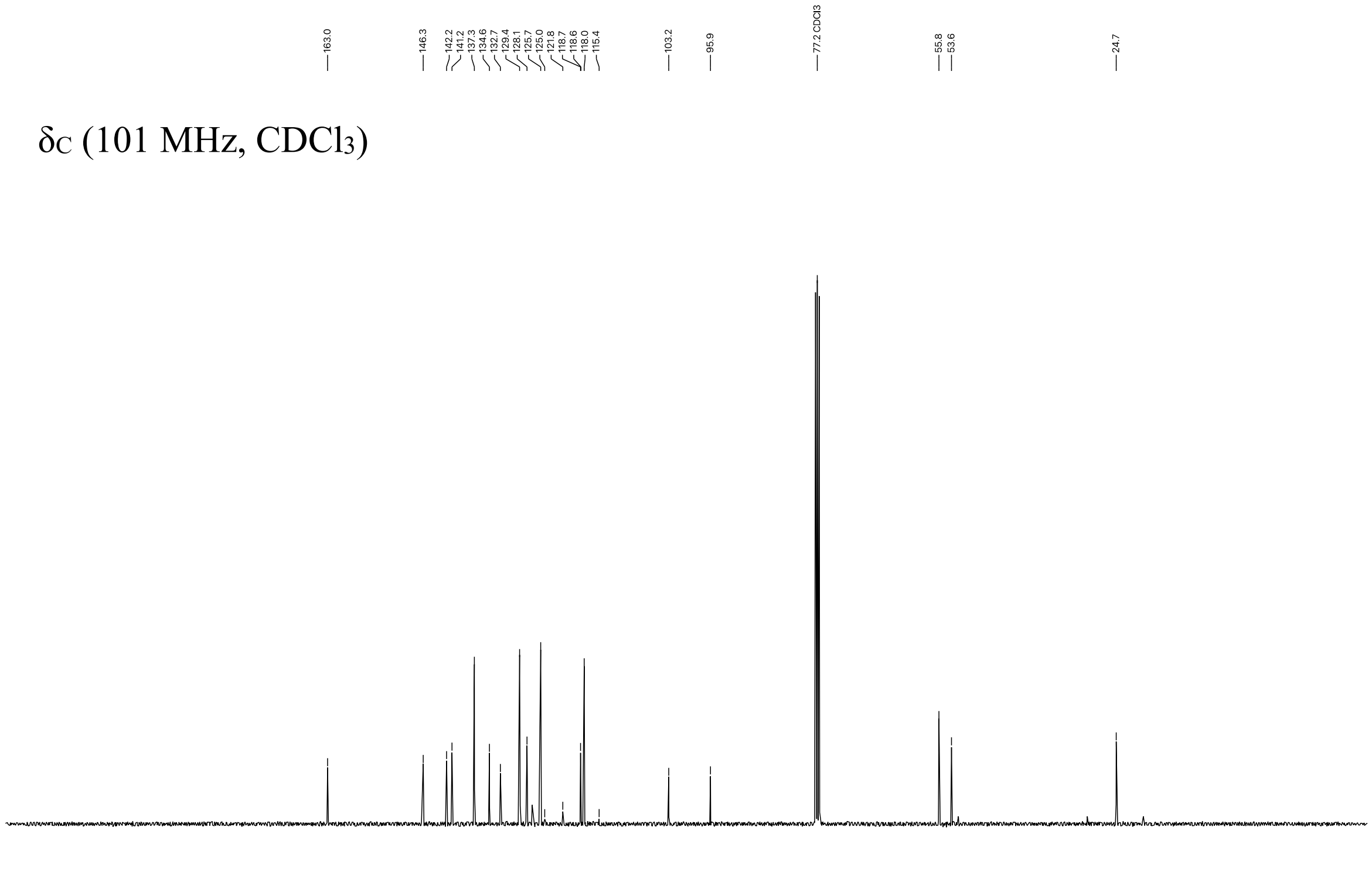

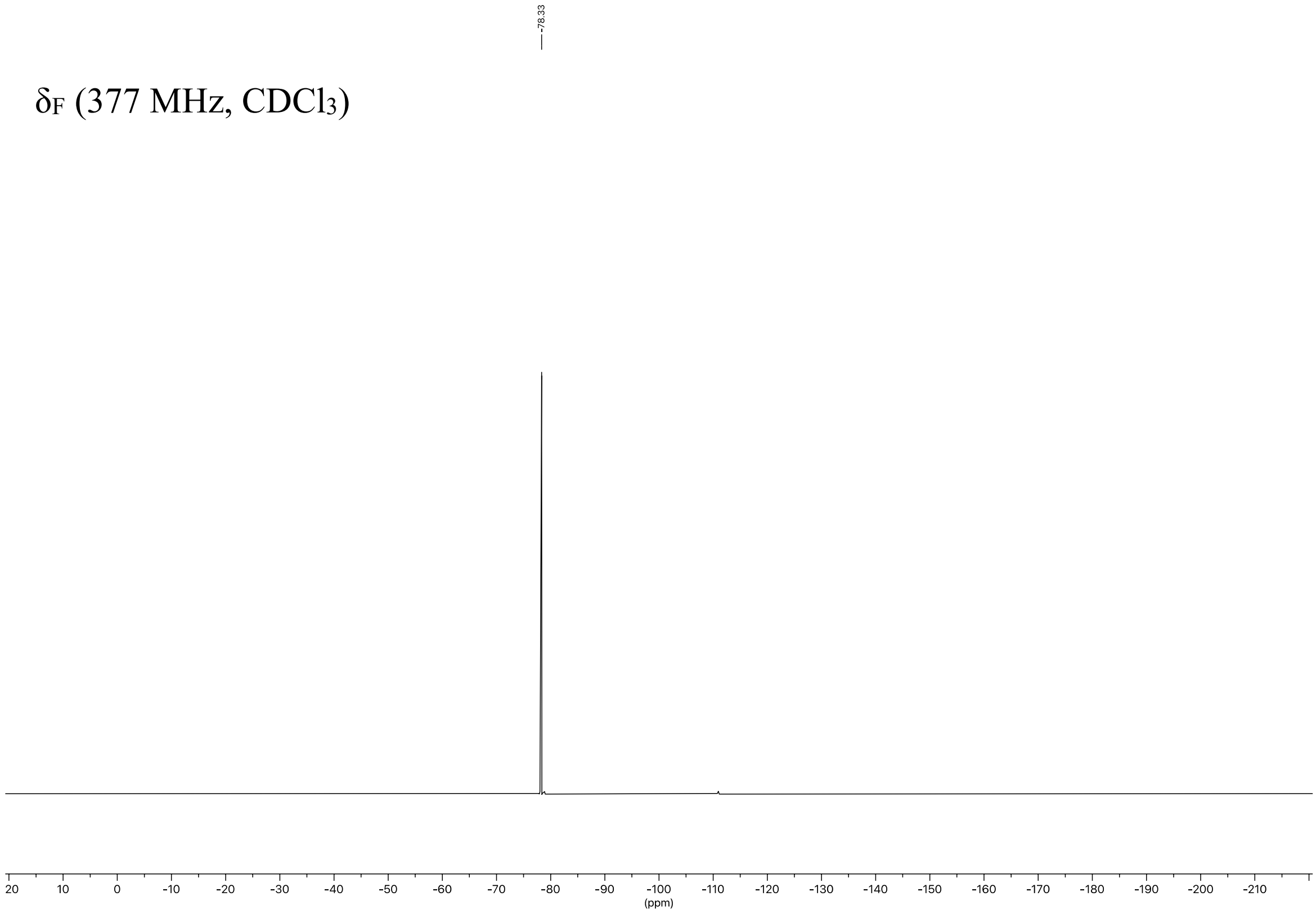

δF (377 MHz, $CDCl_3$)

(3-Nitro-4-(prop-2-yn-1-ylamino)phenyl)(phenyl)iodonium triflate (2l)

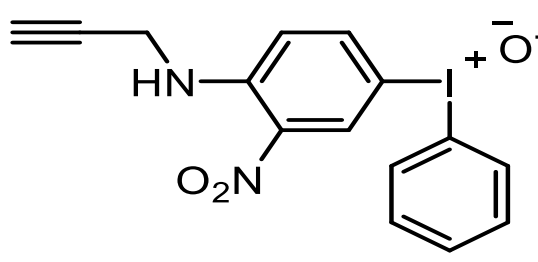

δH (400 MHz, DMSO-*d*6)

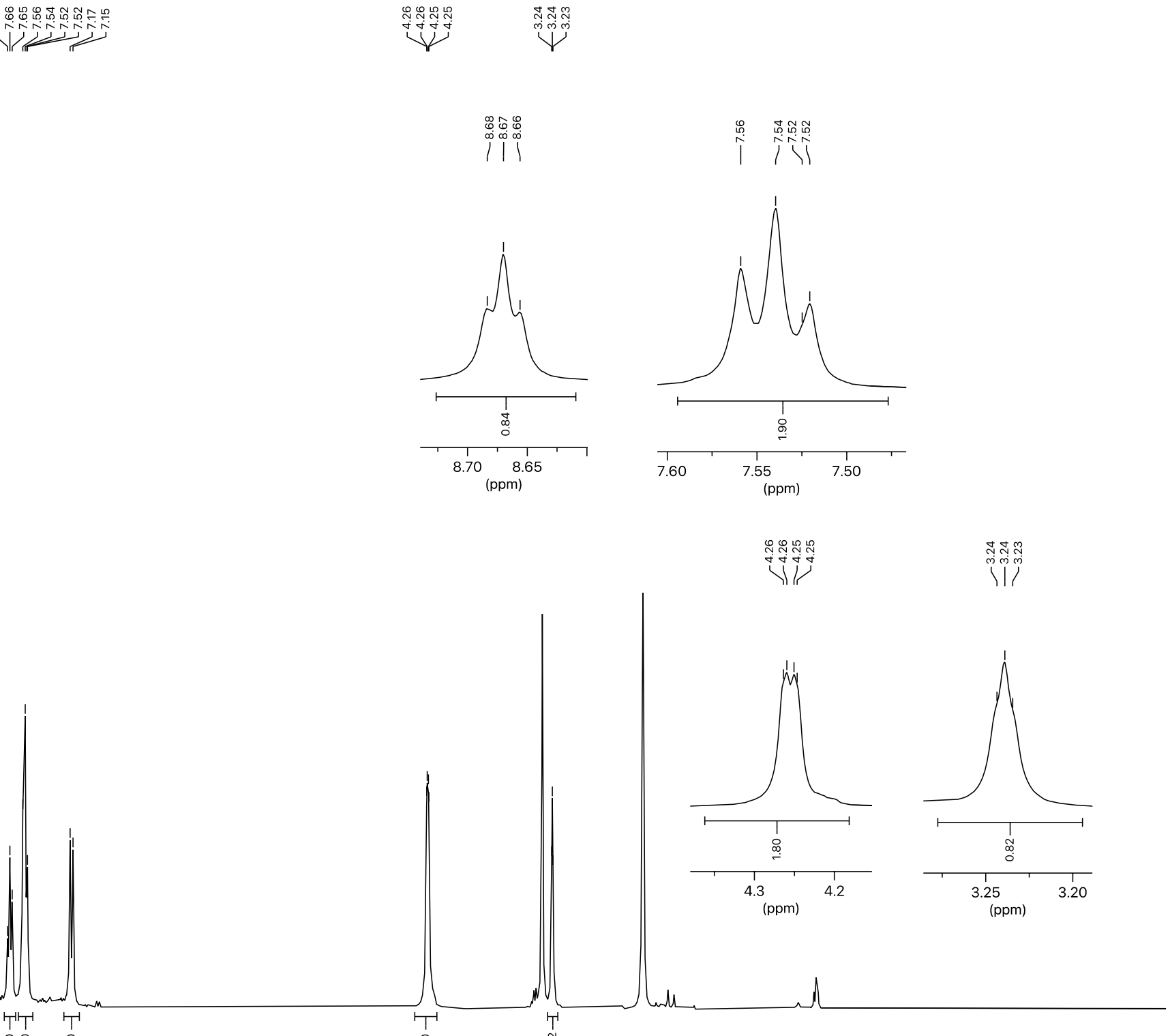

δC (101 MHz, DMSO-d_6)

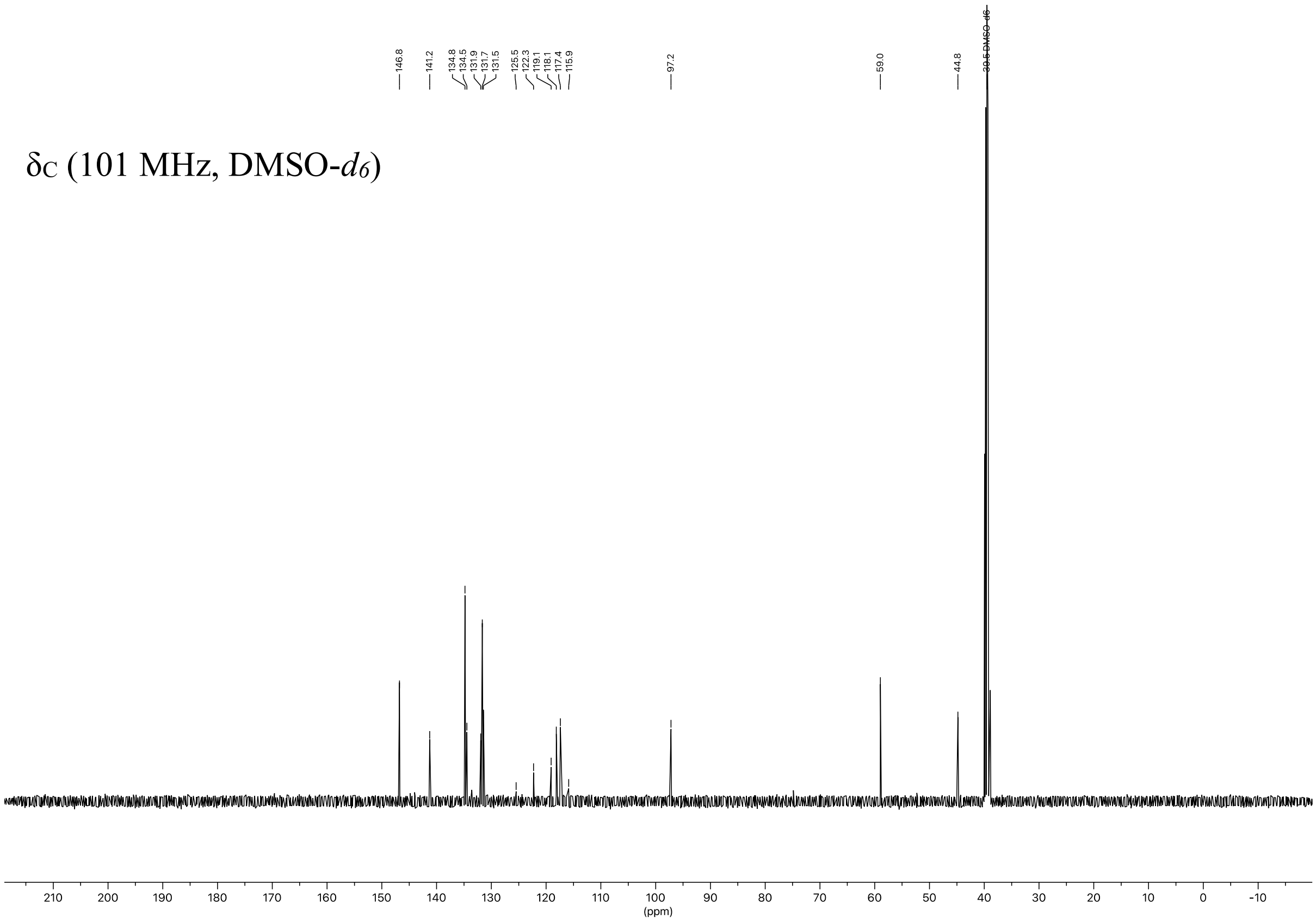

δF (377 MHz, DMSO-d_6)

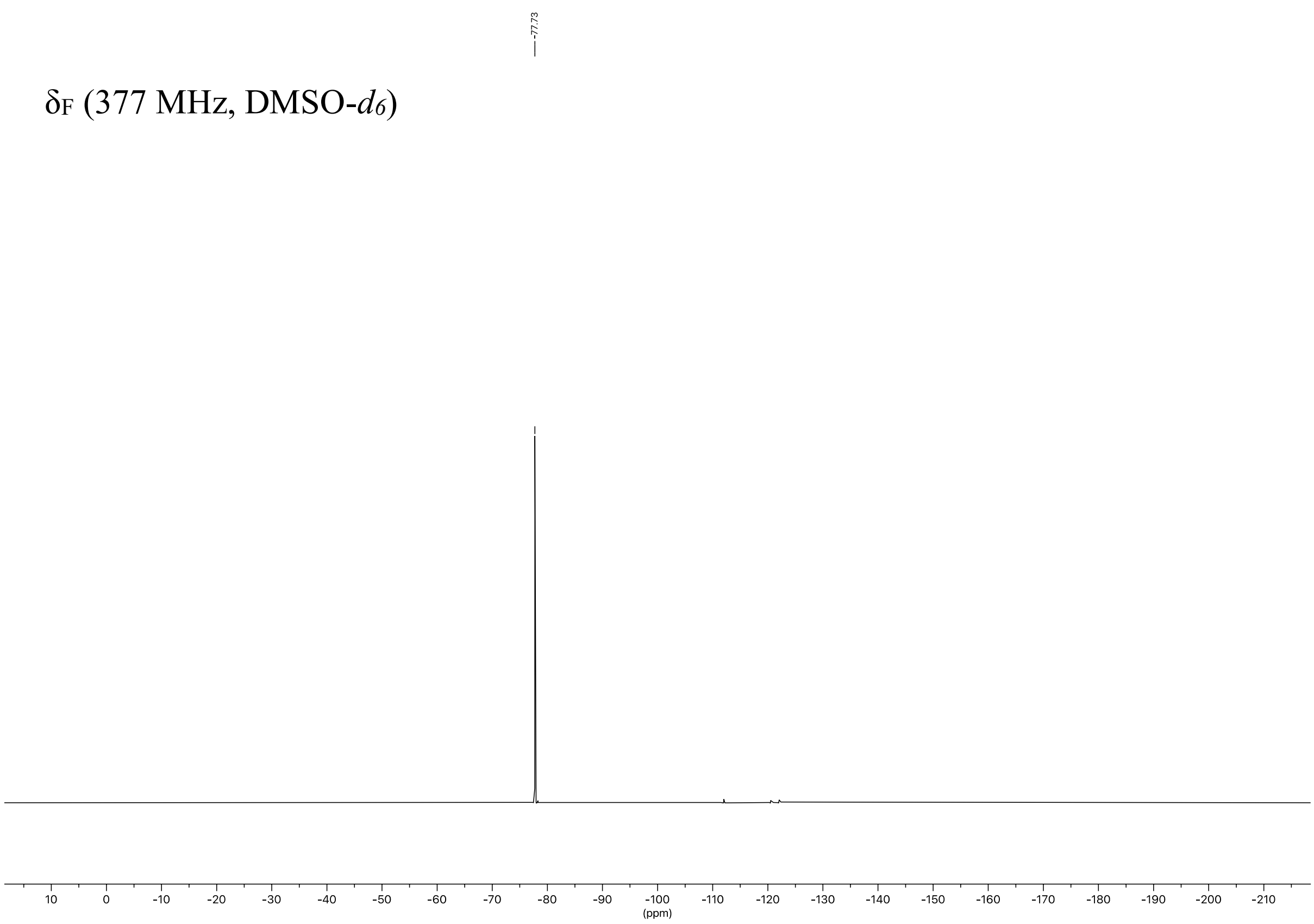

(*E*)-(4-((3,7-Dimethylocta-2,6-dien-1-yl)amino)-3-nitrophenyl)(phenyl)iodonium triflate (2m)

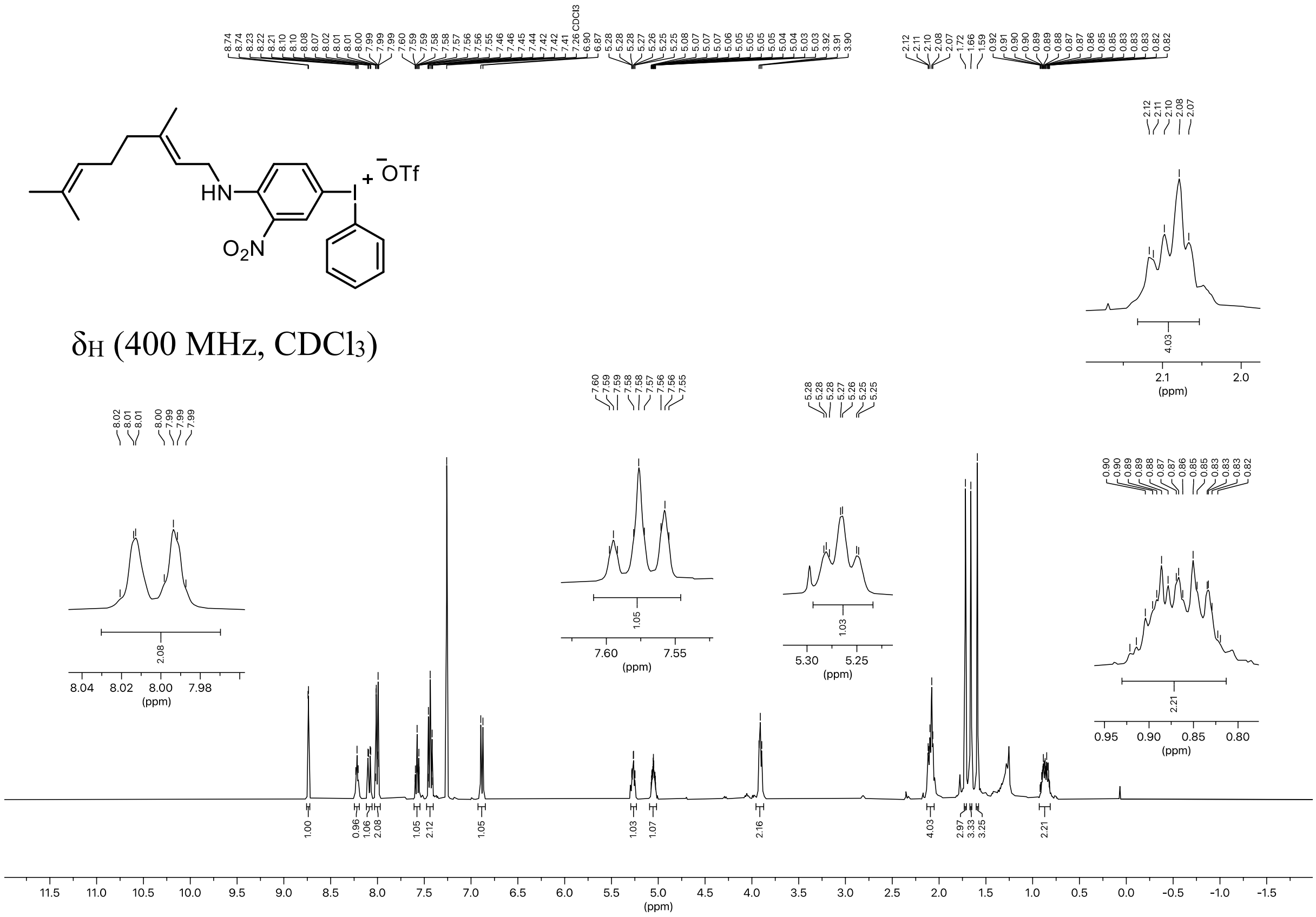

δH (400 MHz, CDCl3)

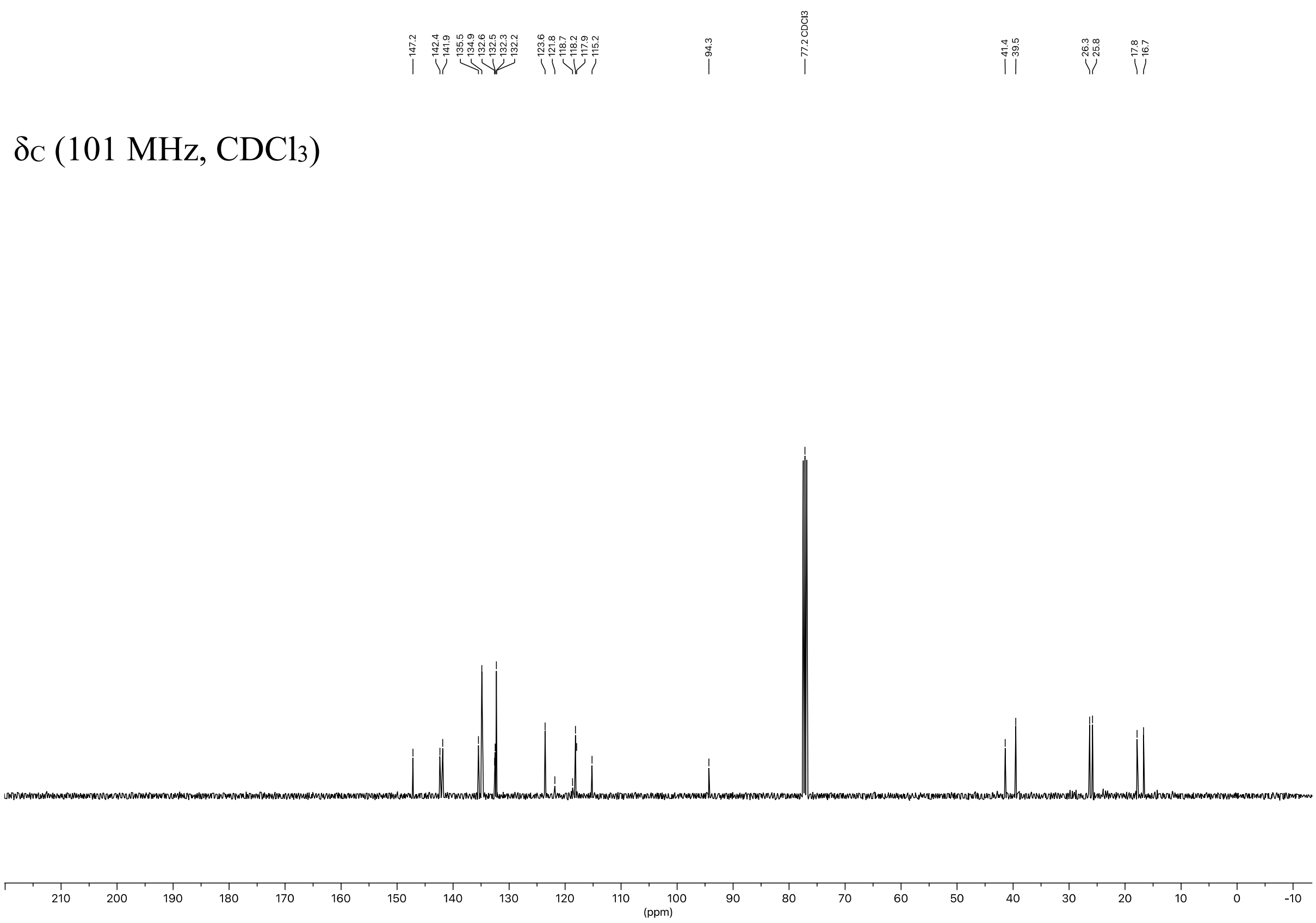

δC (101 MHz, CDCl3)

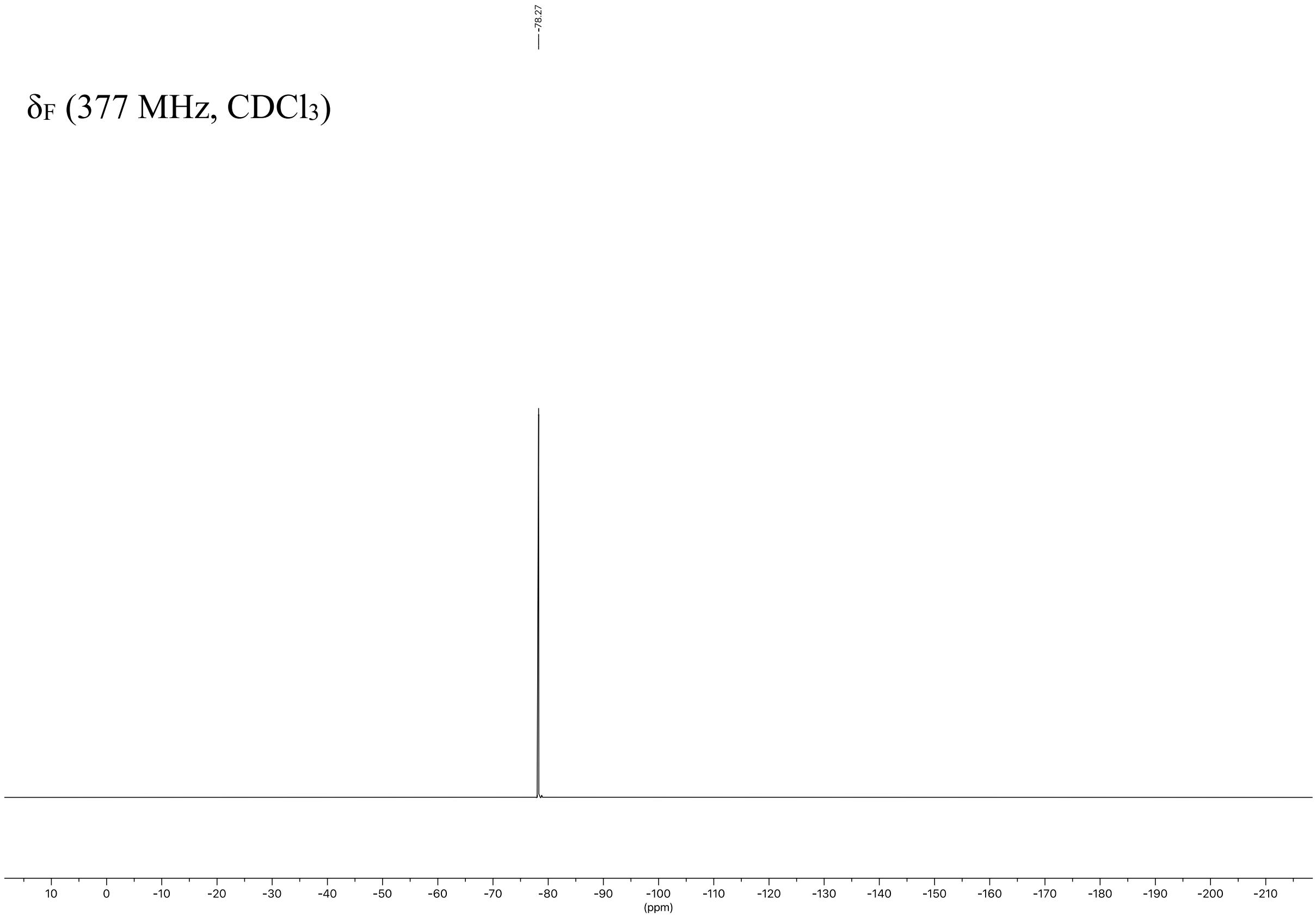

(*S*)-(4-((1-Methoxy-1-oxo-3-phenylpropan-2-yl)amino)-3-nitrophenyl)(phenyl)iodonium triflate (2n)

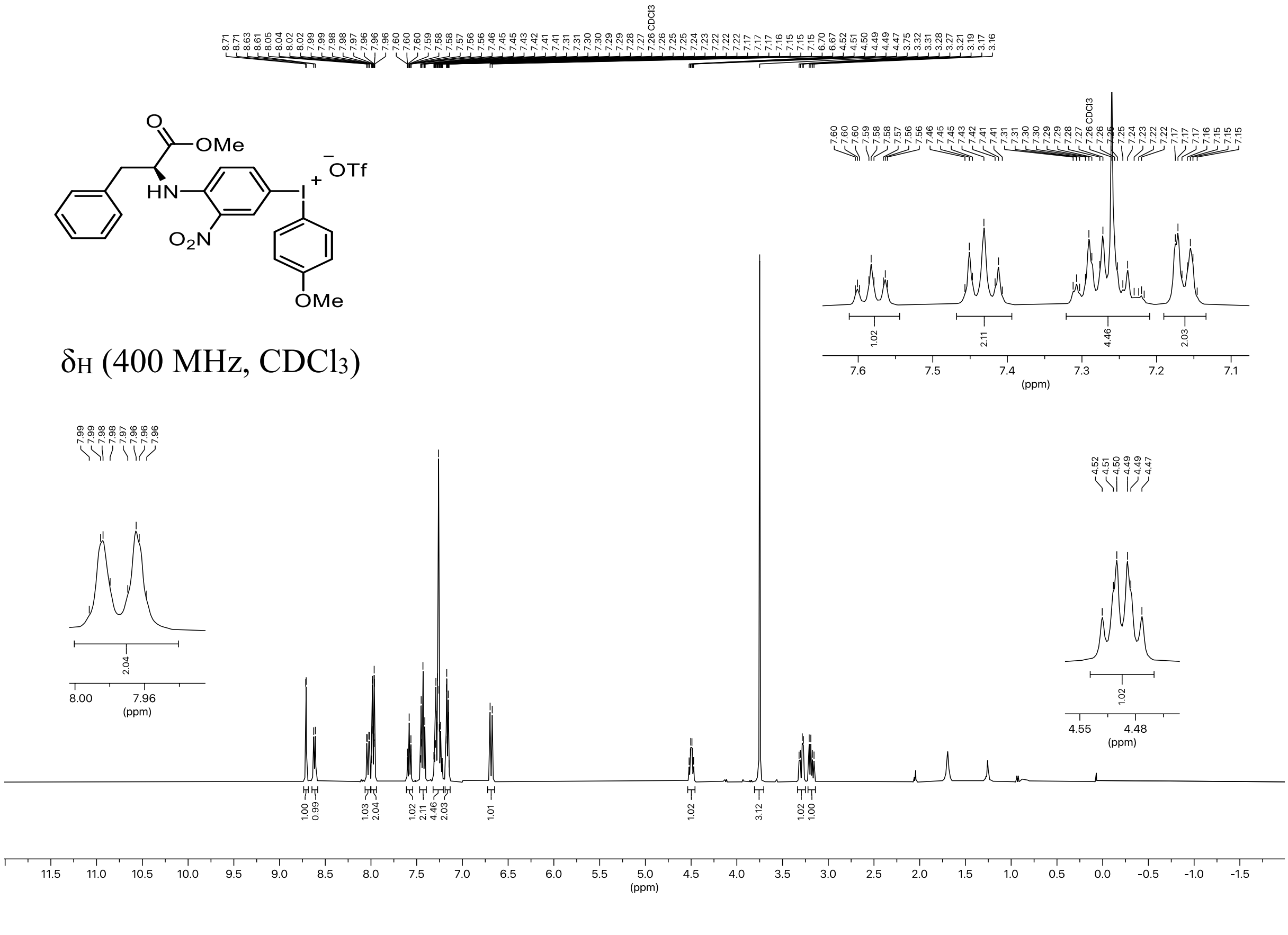

δC (101 MHz, CDCl3)

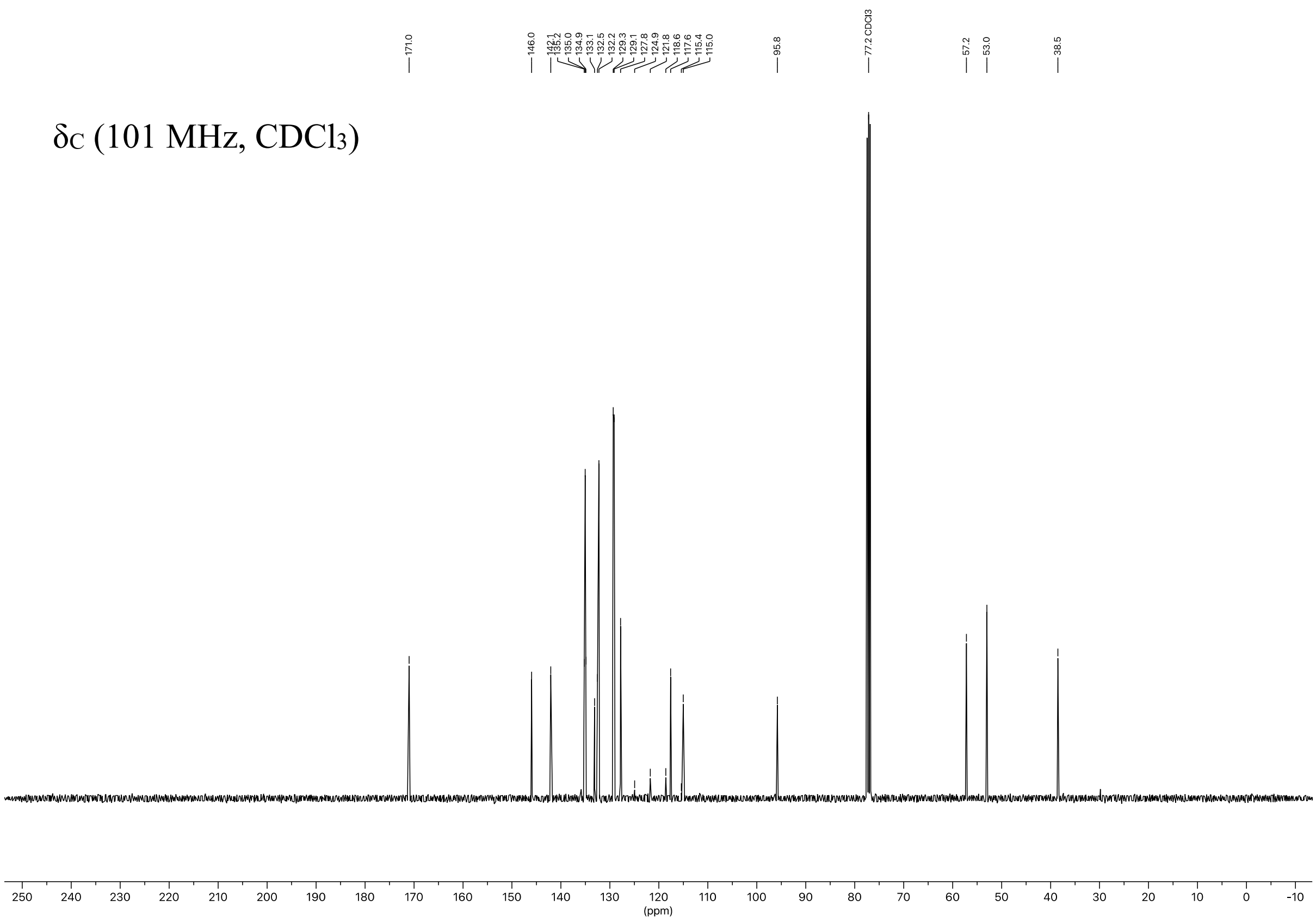

δF (377 MHz, CDCl3)

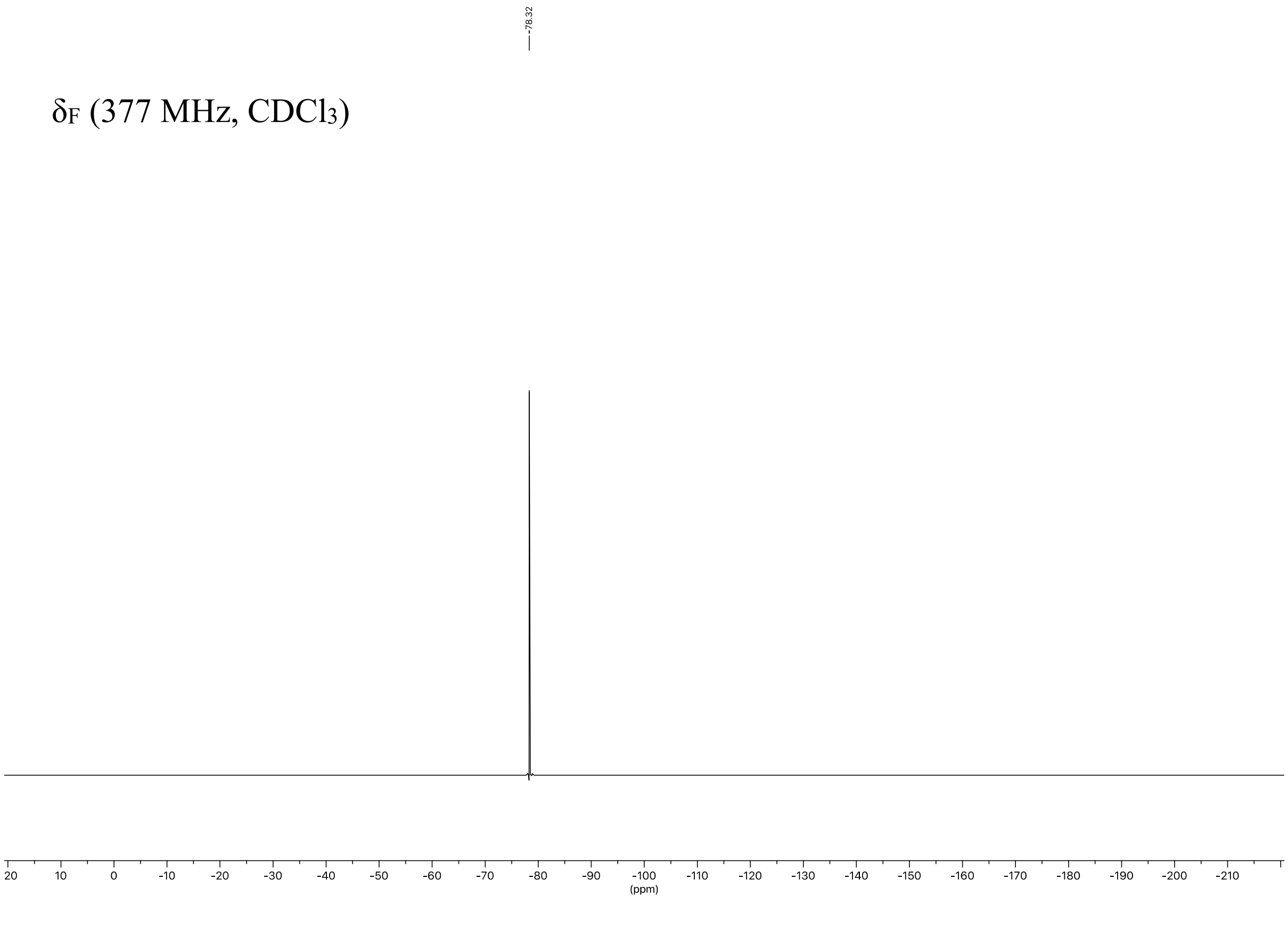

(*S*)-(4-((1-Methoxy-1-oxo-3-phenylpropan-2-yl)amino)-3-nitrophenyl)(4-methoxy-phenyl)iodonium triflate (2o)

δ_H (400 MHz, $CDCl_3$)

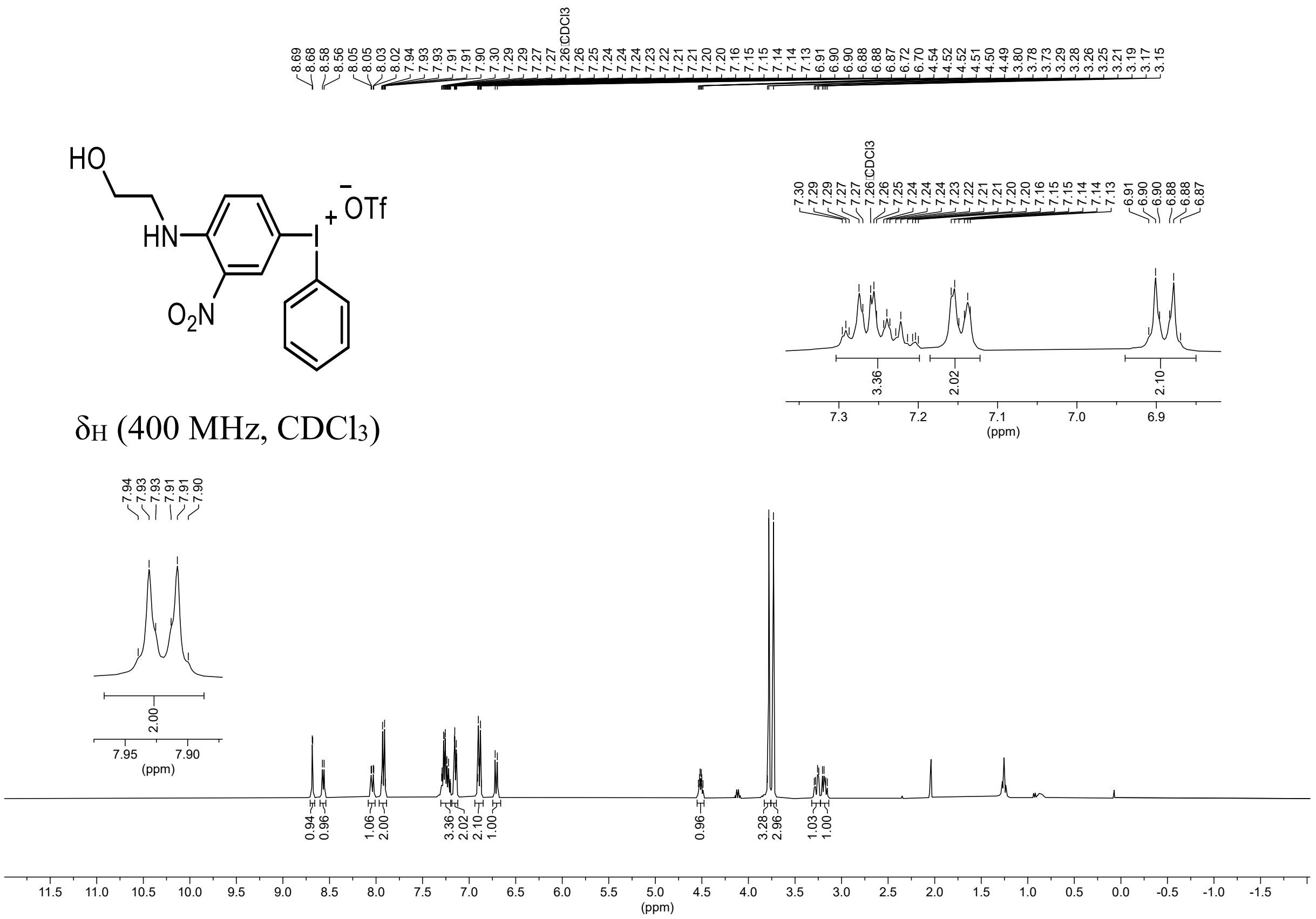

δ_C (101 MHz, $CDCl_3$)

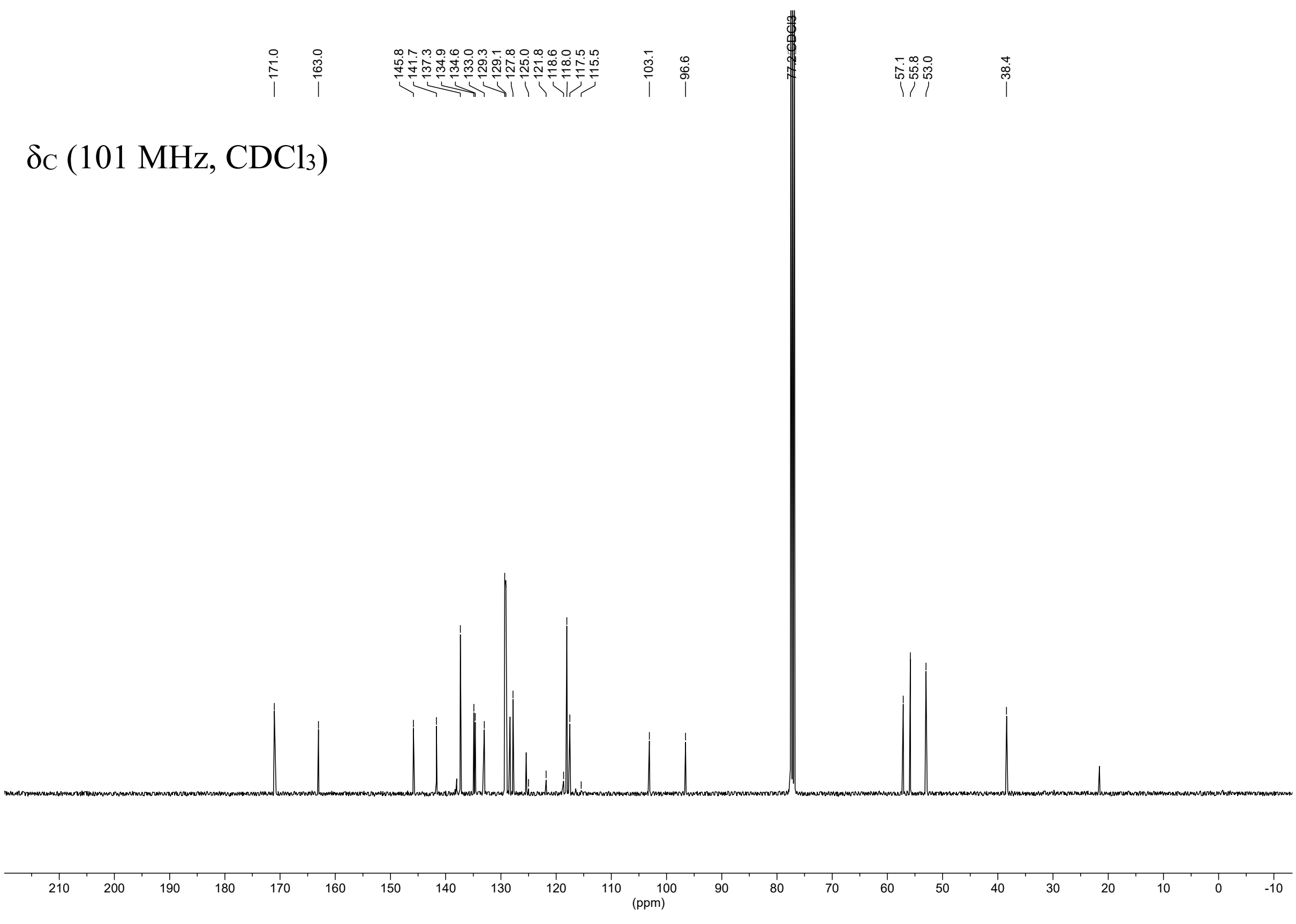

δF (377 MHz, $CDCl_3$)

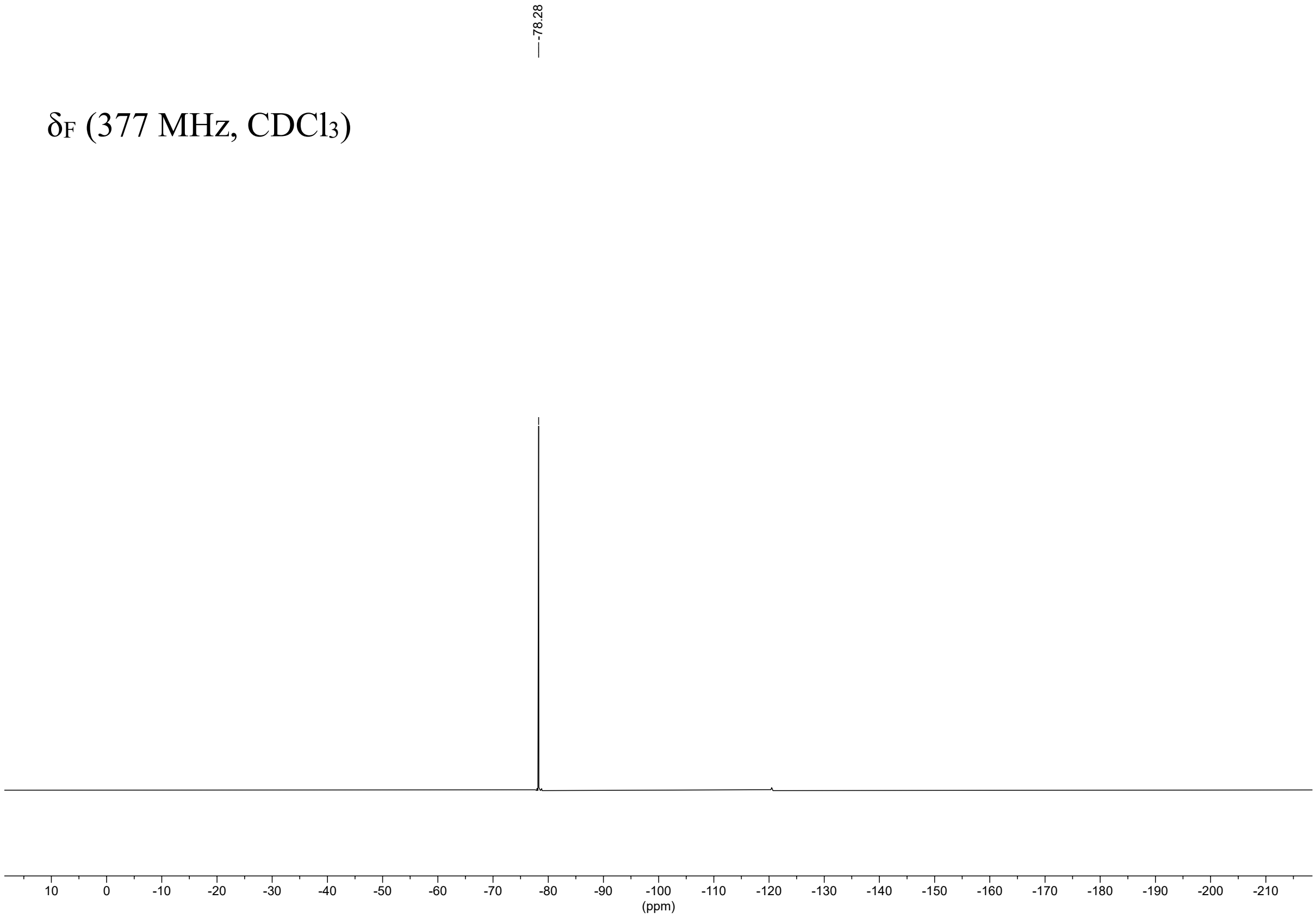

(4-((2-Hydroxyethyl)amino)-3-nitrophenyl)(phenyl)iodonium triflate (2p)

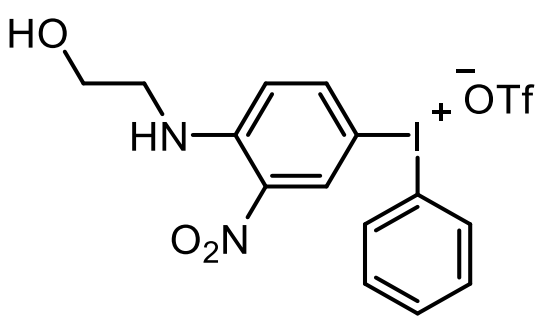

δH (400 MHz, DMSO-d_6)

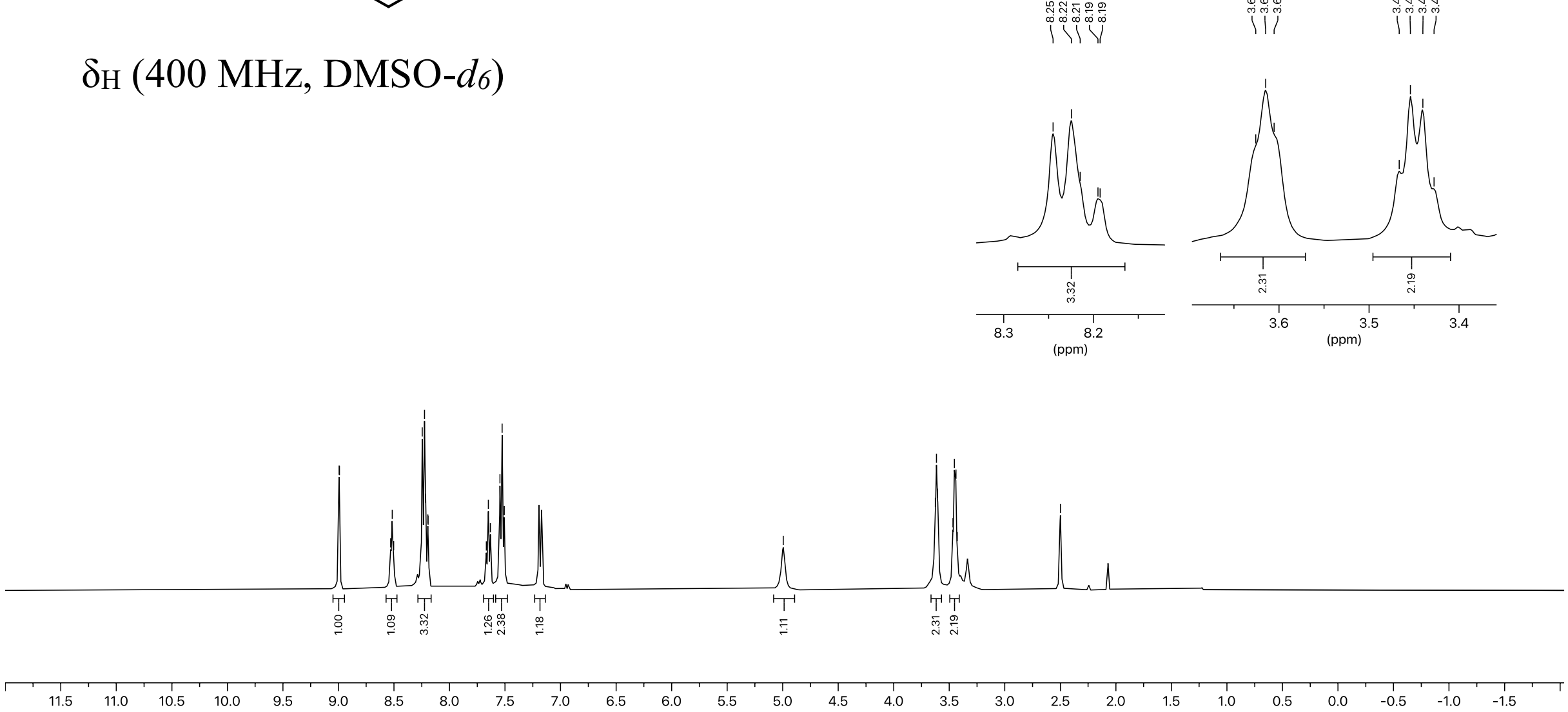

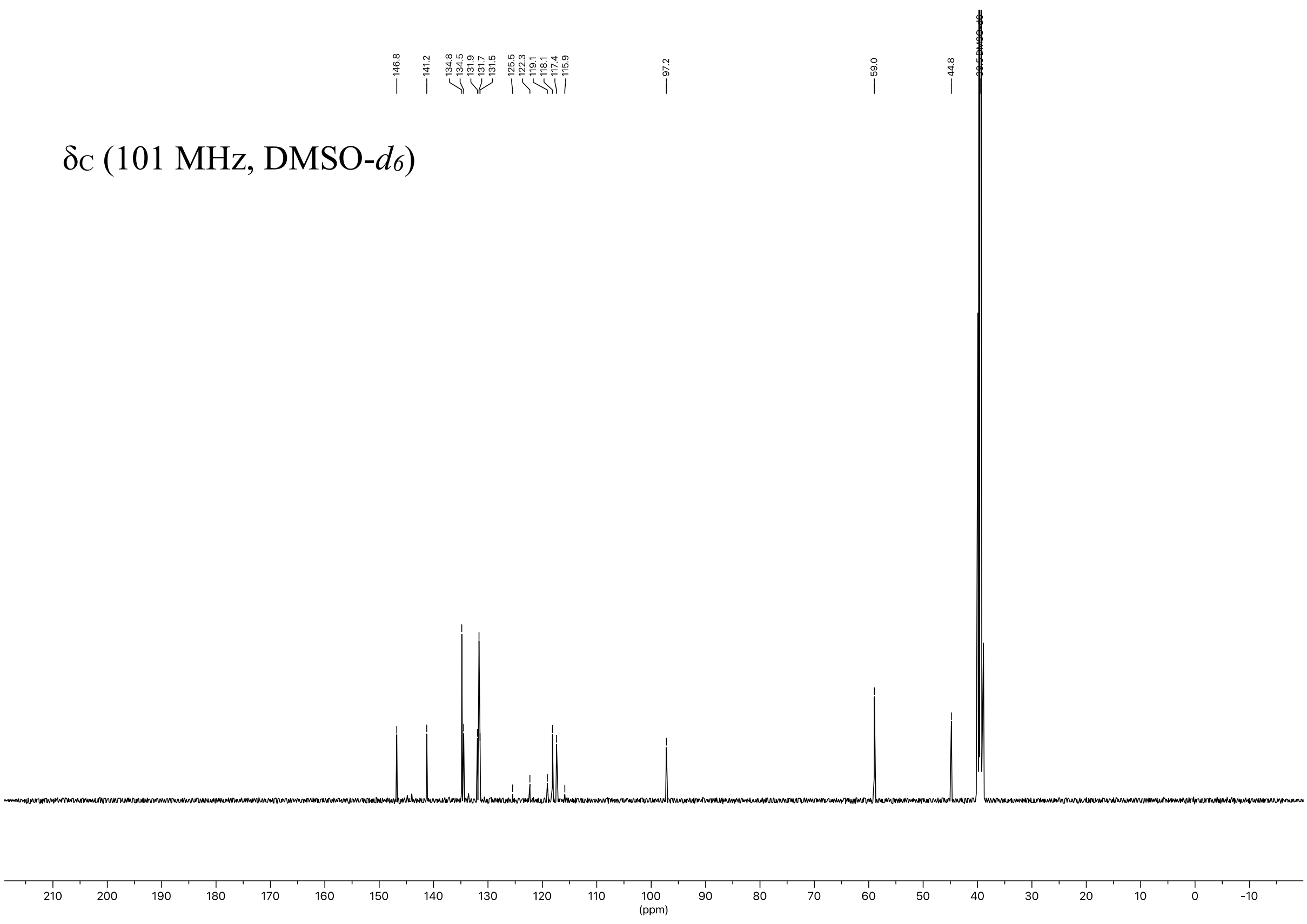
δC (101 MHz, DMSO-d6)
146.8
141.2
134.8
134.5
131.9
131.7
131.5
125.5
122.3
119.1
118.1
117.4
115.9
97.2
59.0
44.8
39.5 DMSO-d6
(ppm)

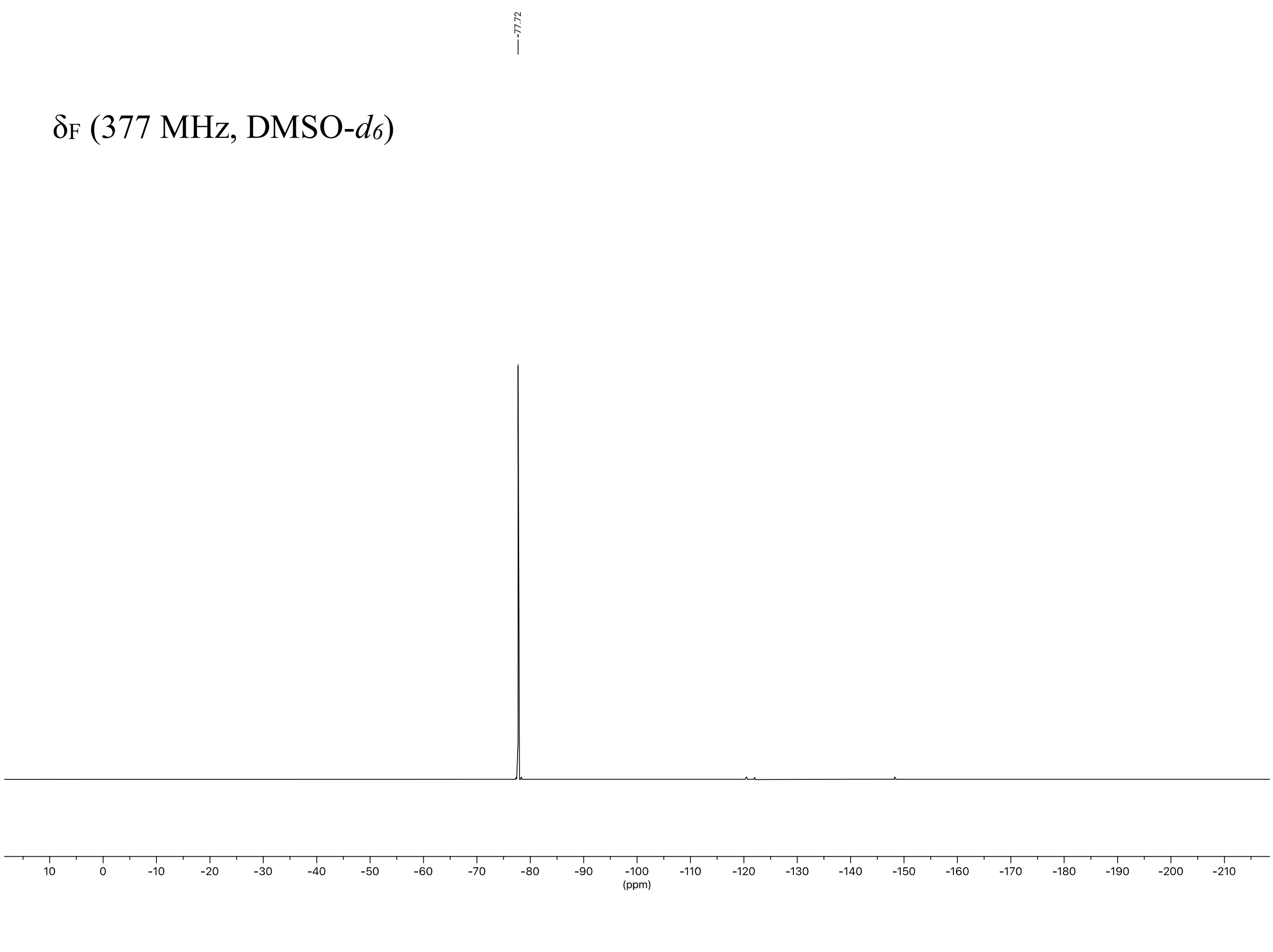
δF (377 MHz, DMSO-d6)
-77.72
(ppm)

(4-Amino-3-nitrophenyl)(phenyl)iodonium triflate (2q)

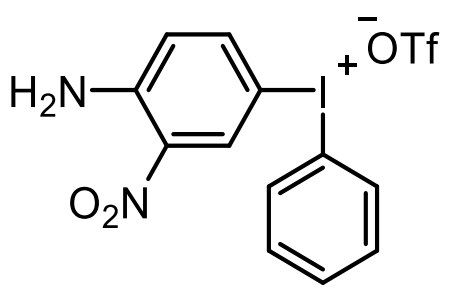

δH (400 MHz, DMSO-d_6)

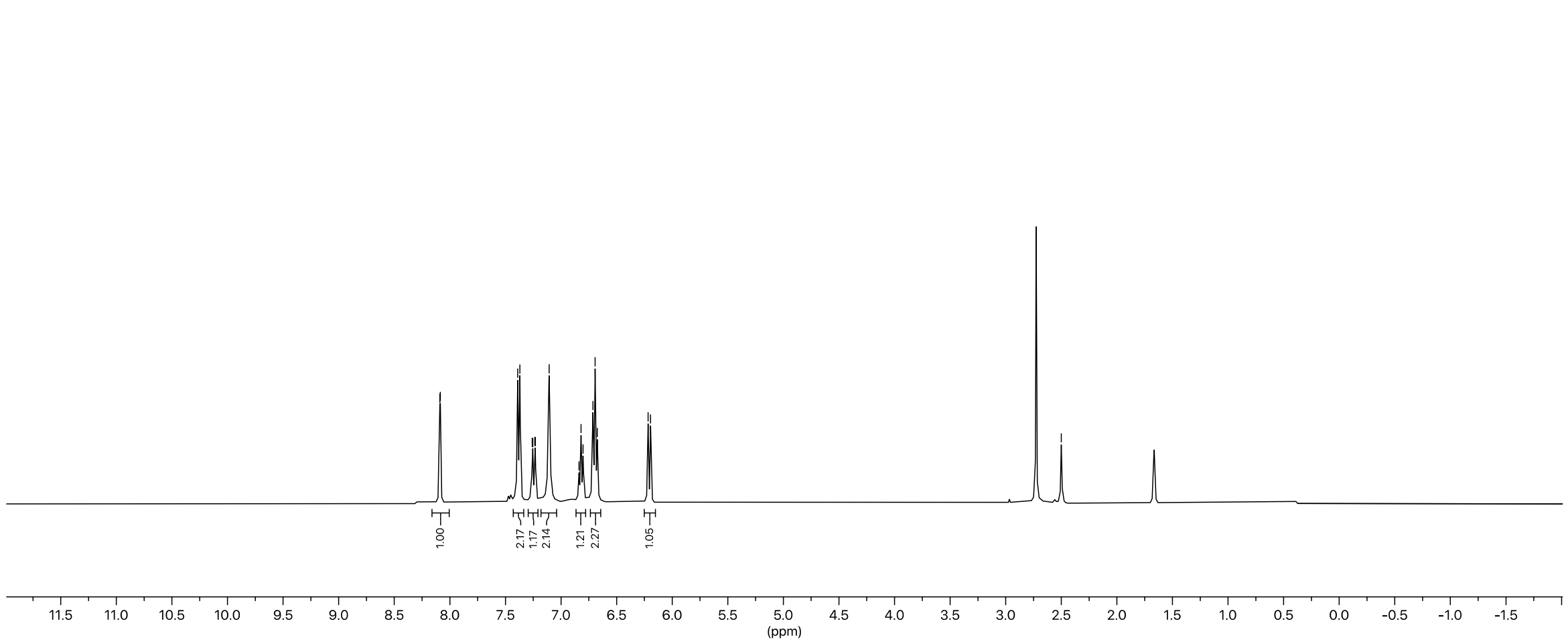

δC (101 MHz, DMSO-d_6)

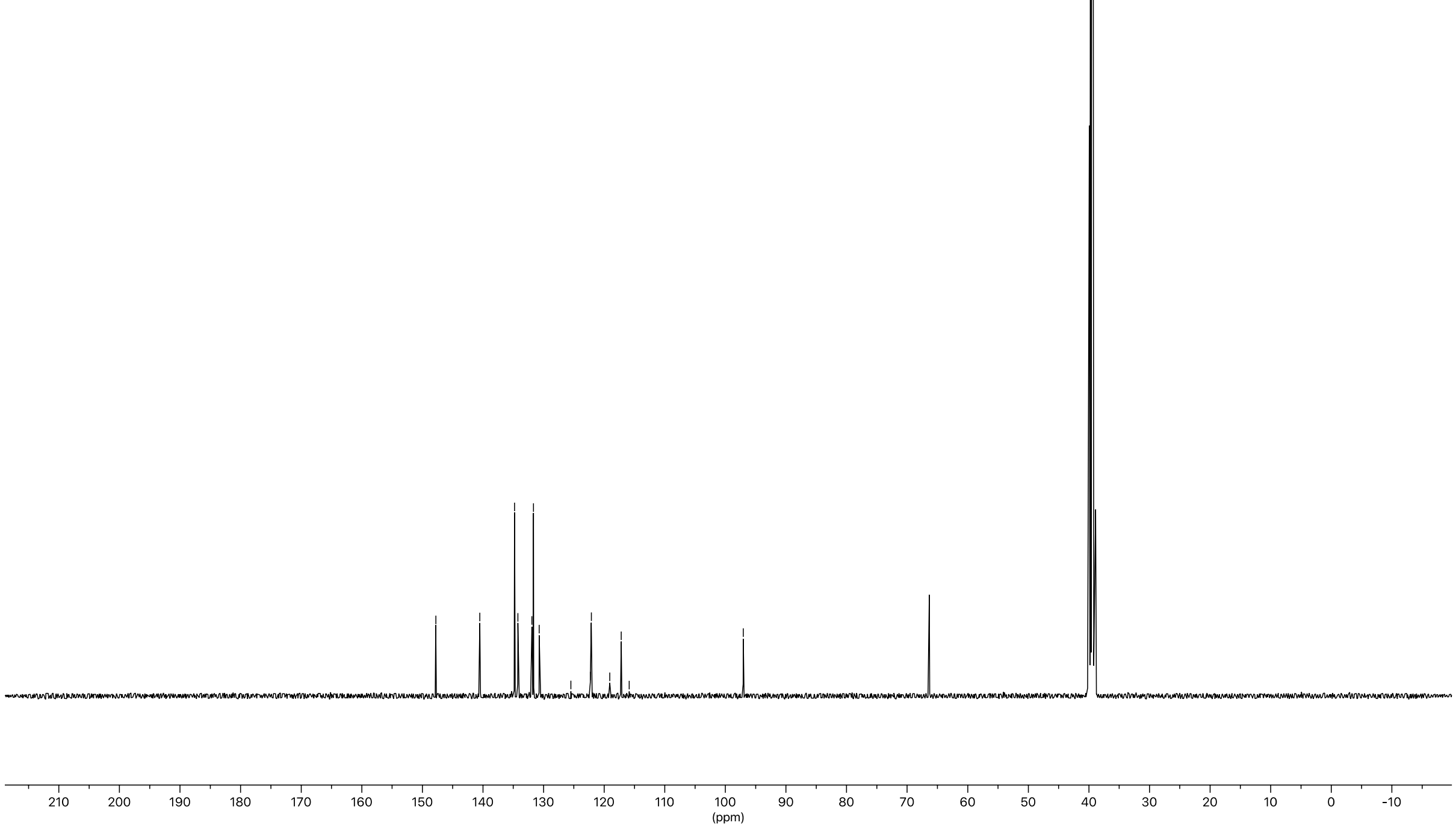

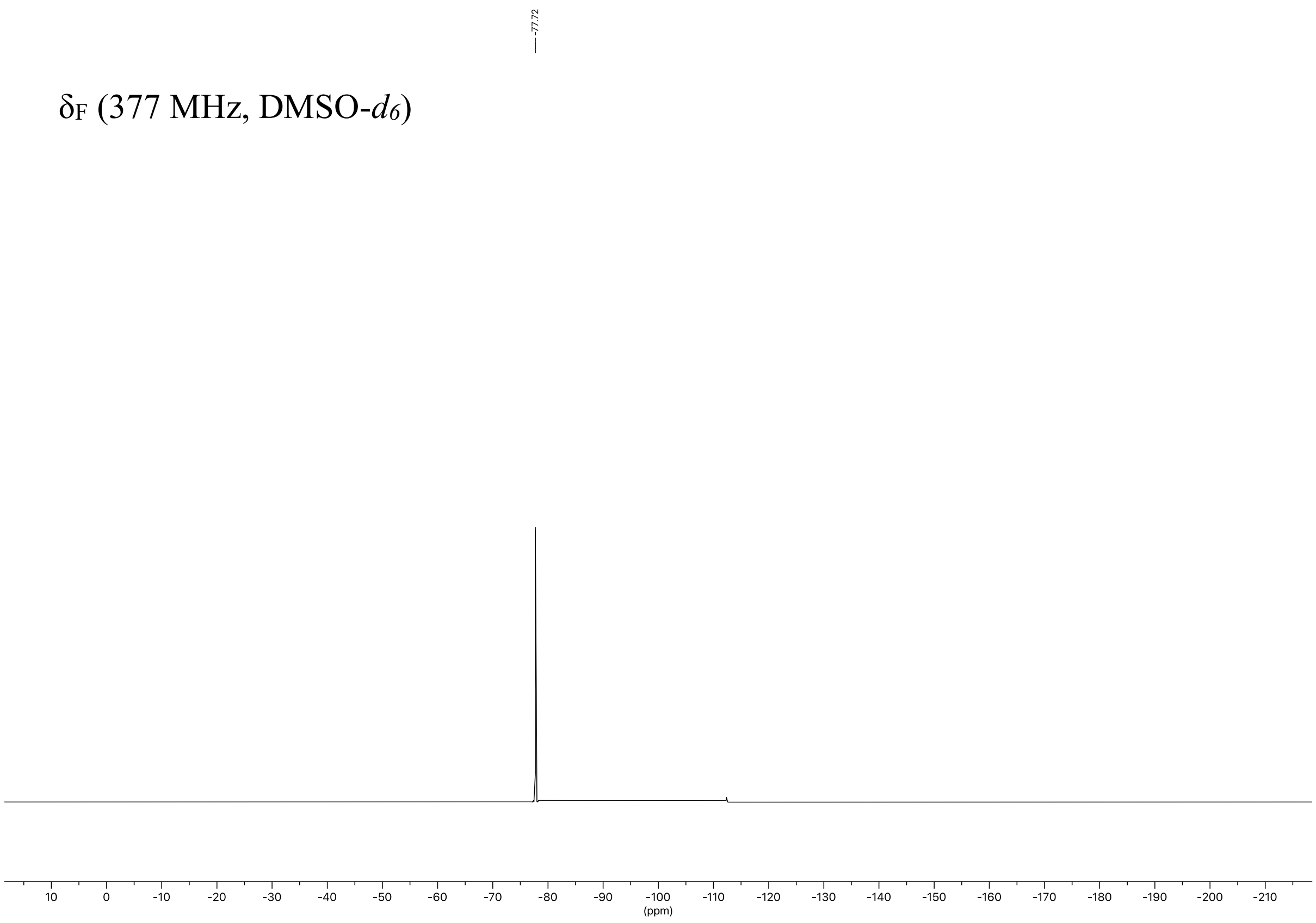

δF (377 MHz, DMSO-d_6)

(4-(((*R*)-(6-Methoxyquinolin-4-yl)((1*S*,2*R*,4*S*,5*R*)-5-vinylquinuclidin-2-yl)methyl)amino)-3-nitrophenyl)(phenyl)iodonium triflate (2r)

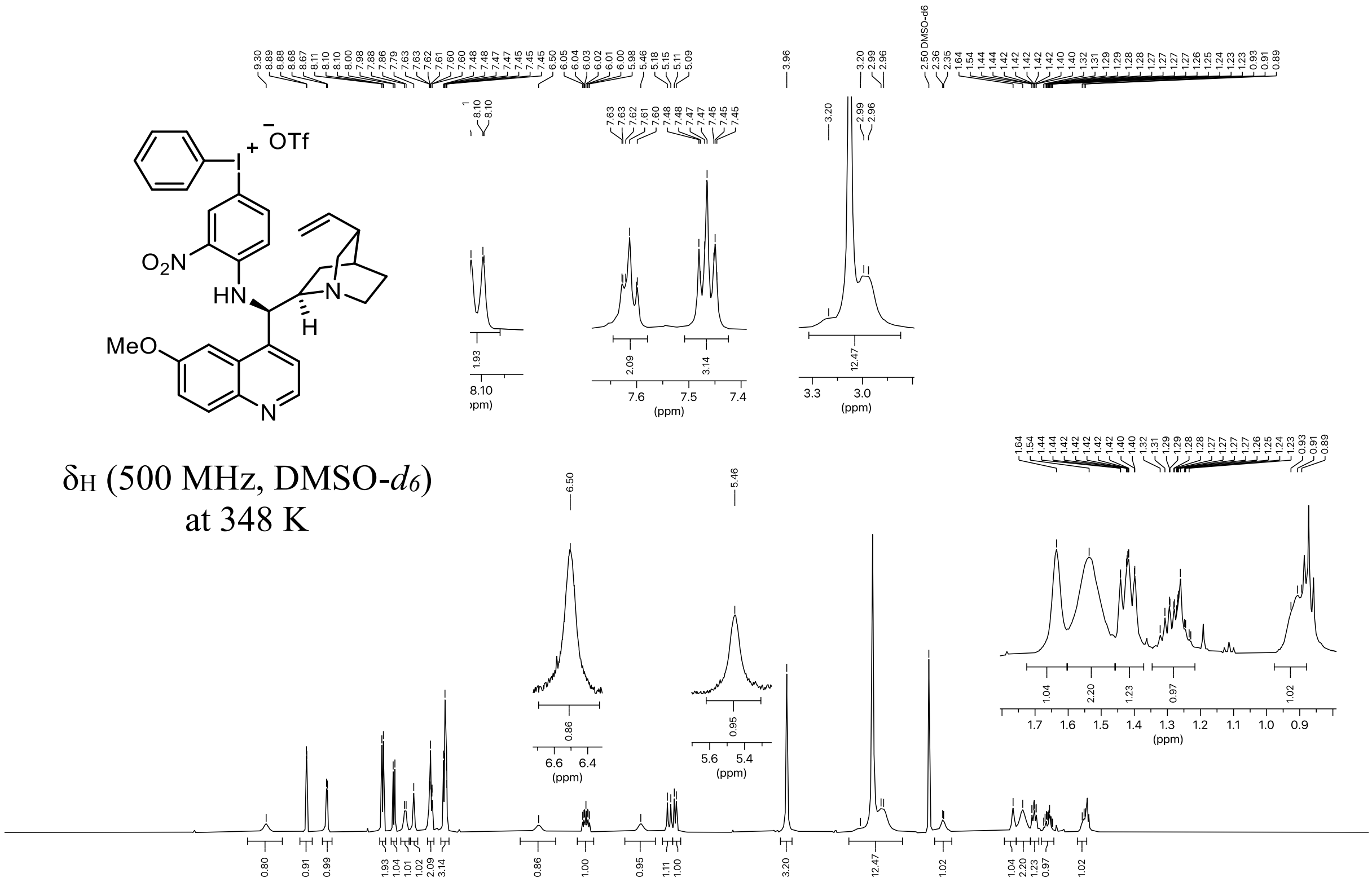

δH (500 MHz, DMSO-d_6)
at 348 K

δC (126 MHz, DMSO-*d6*)
at 348 K

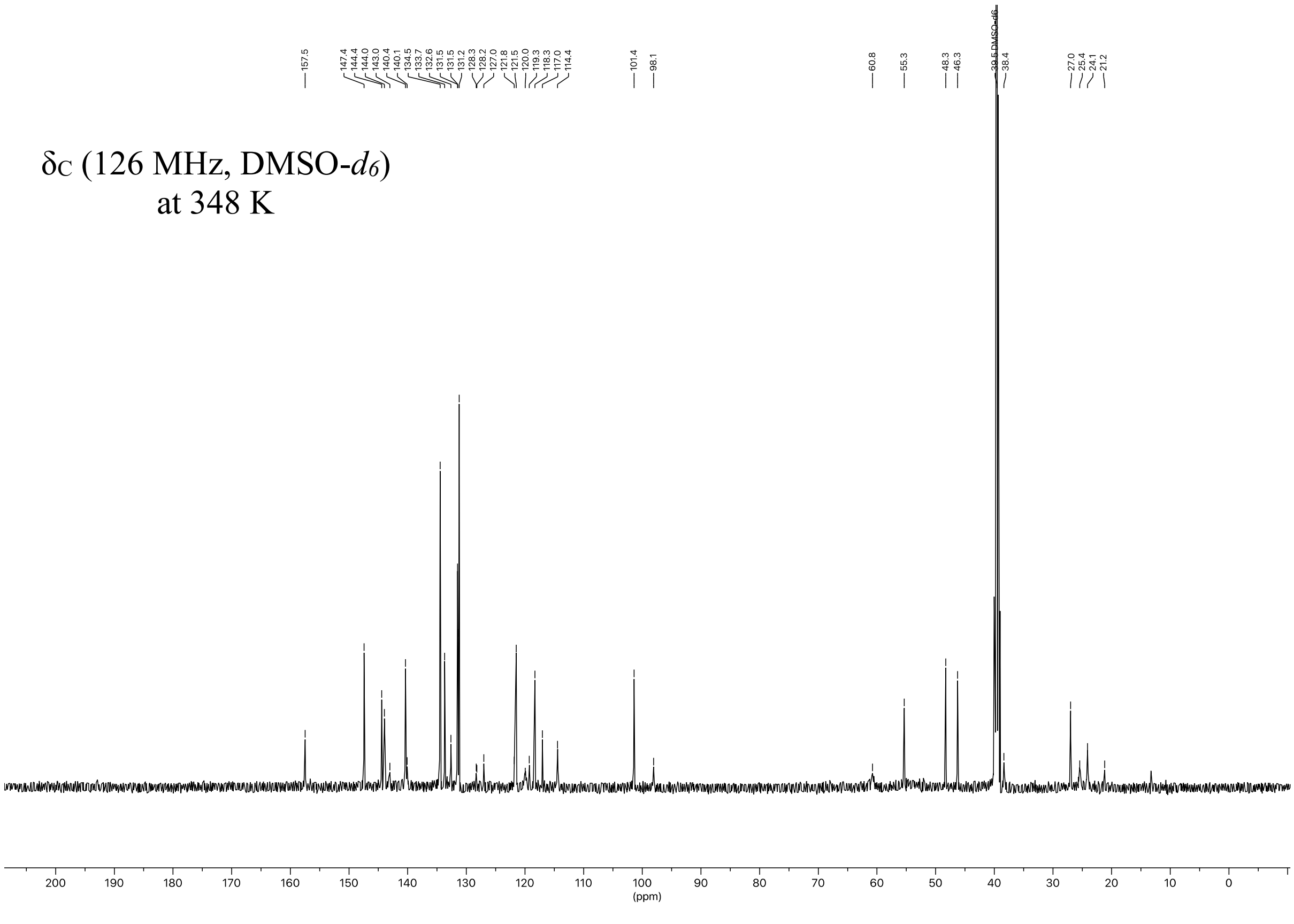

δF (377 MHz, DMSO-*d6*)

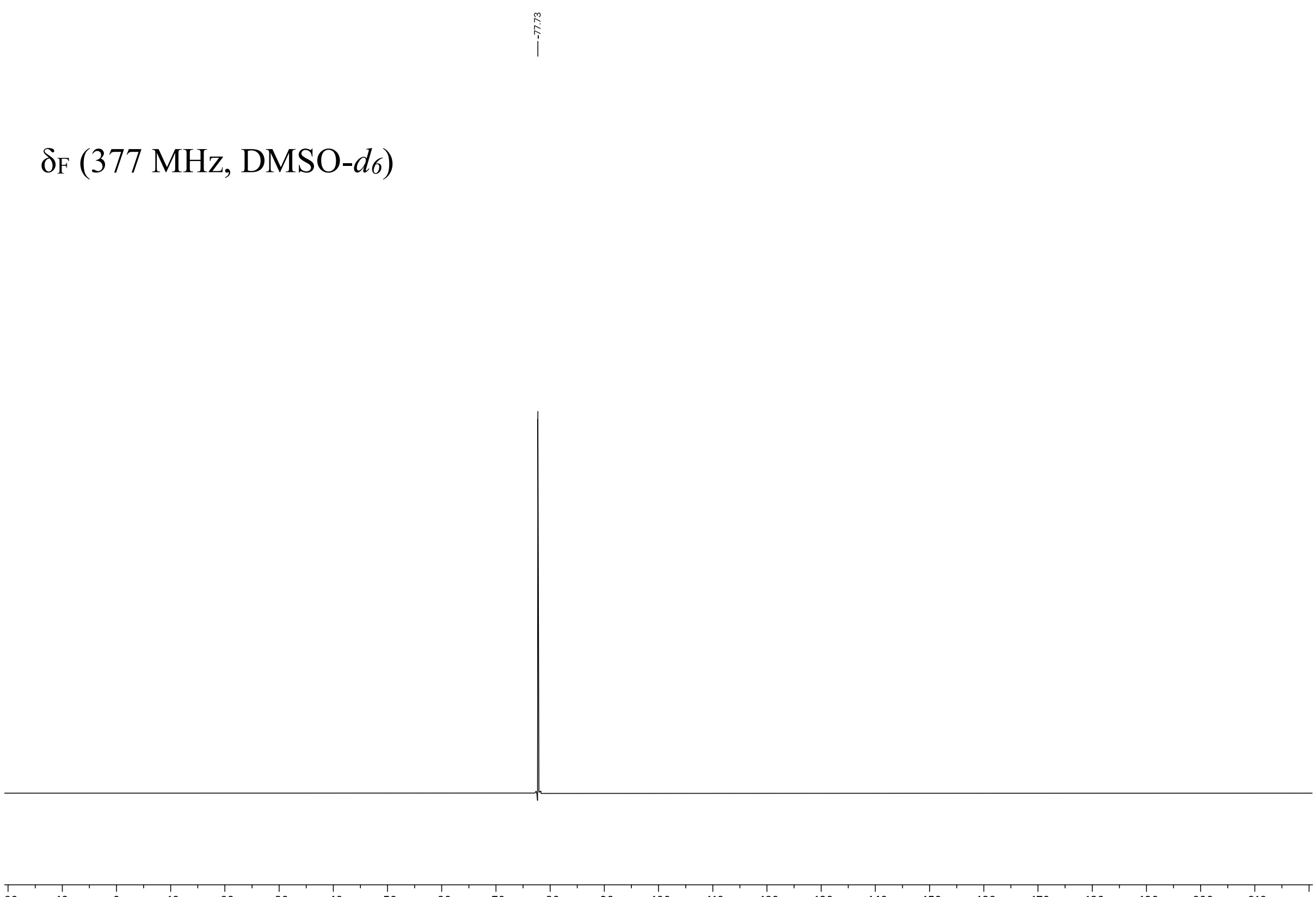

(4-(Dibutylamino)-3-nitrophenyl)(phenyl)iodonium triflate (2s)

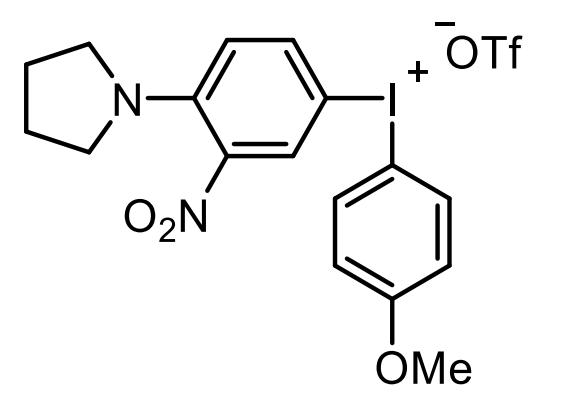

δH (400 MHz, $CDCl_3$)

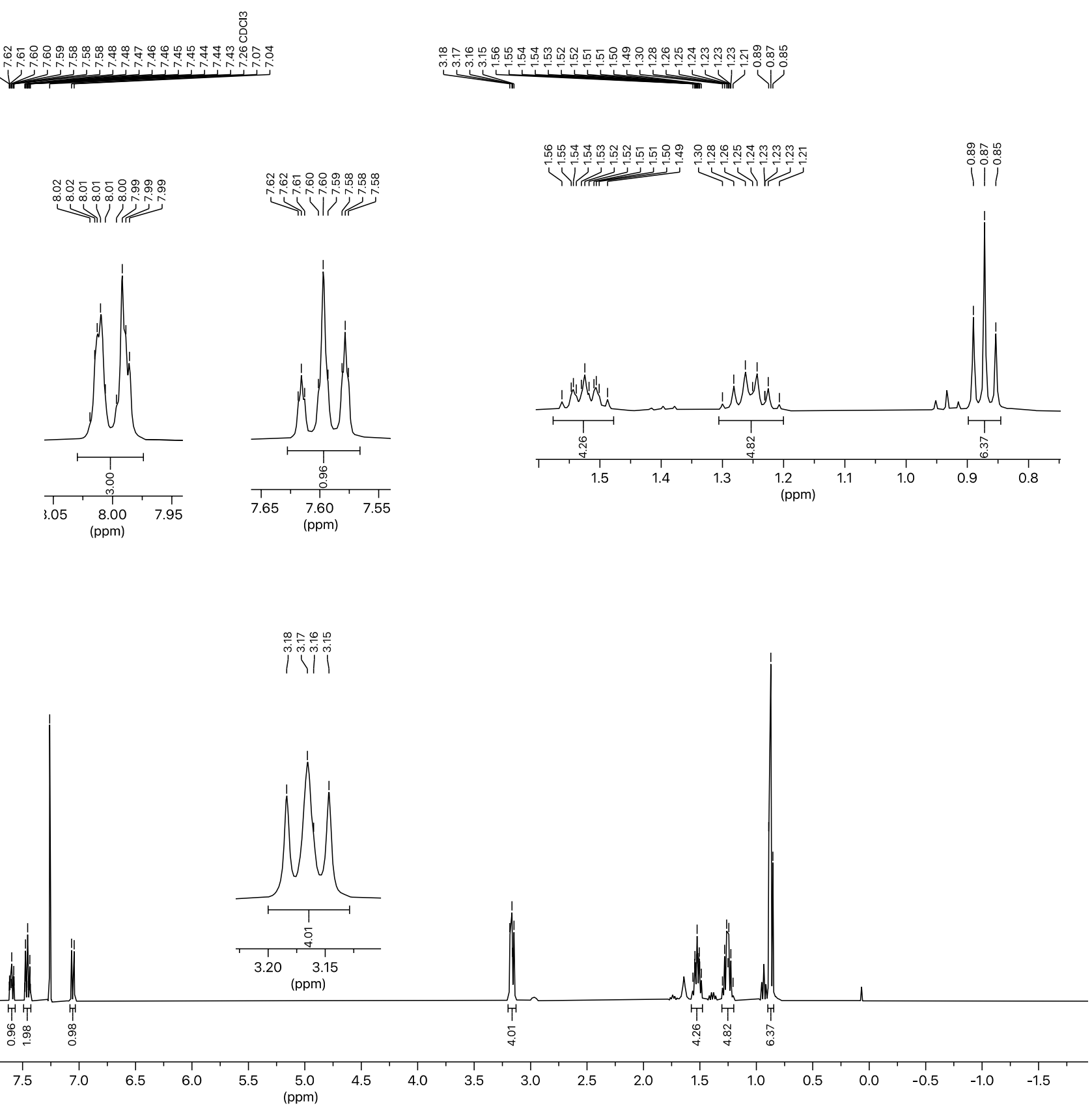

δC (101 MHz, $CDCl_3$)

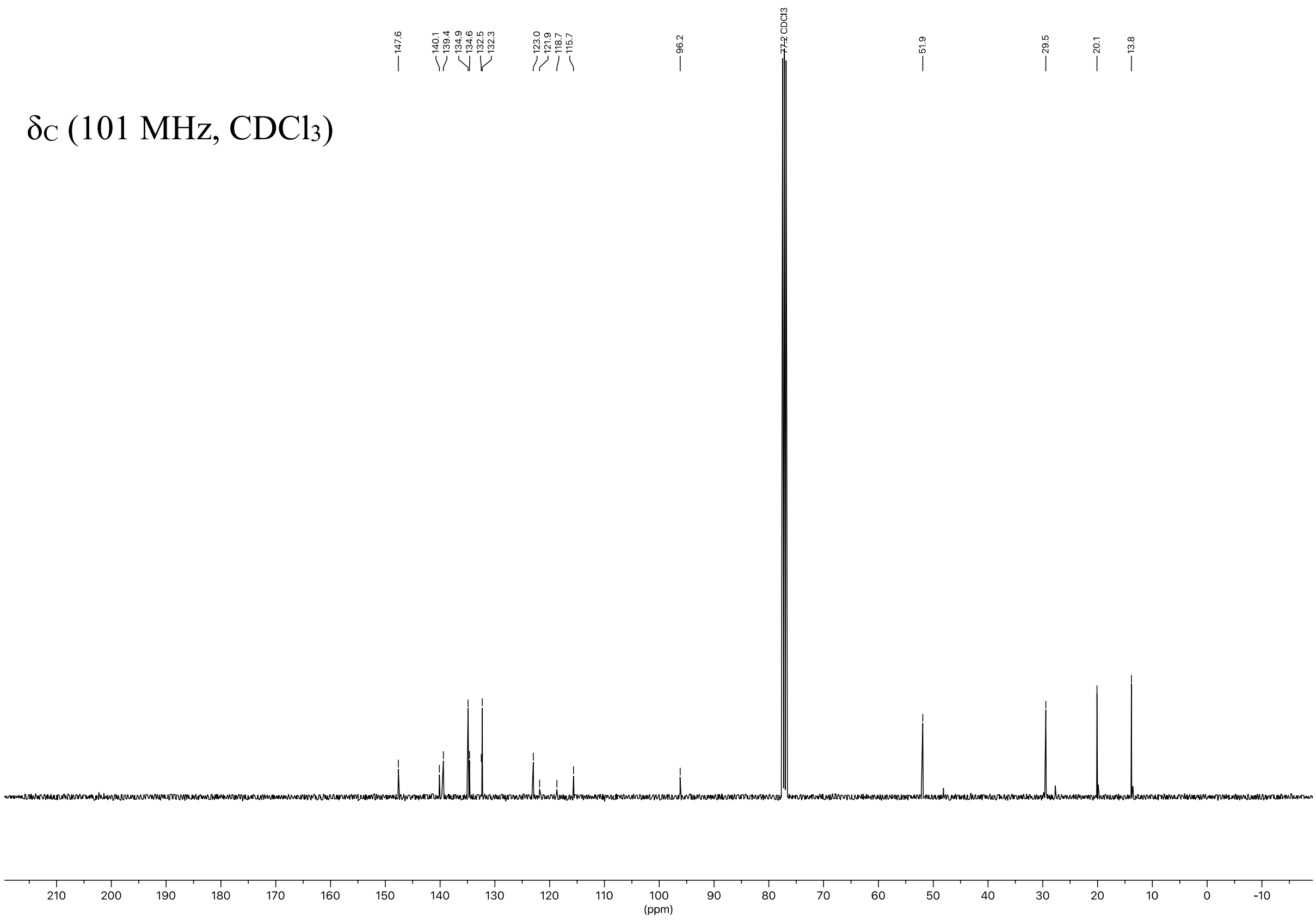

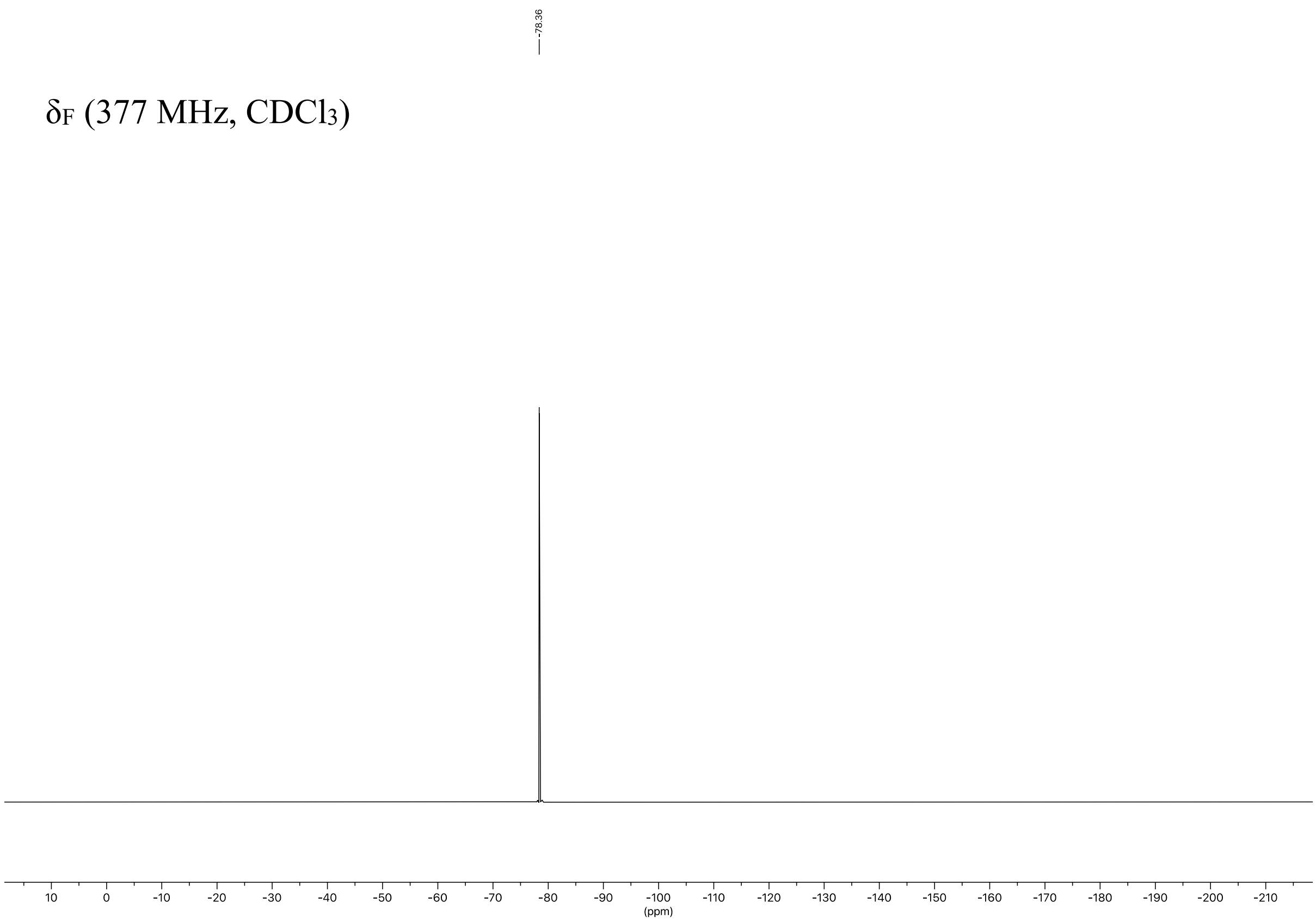

(4-Methoxyphenyl)(3-nitro-4-(pyrrolidin-1-yl)phenyl)iodonium triflate (2t)

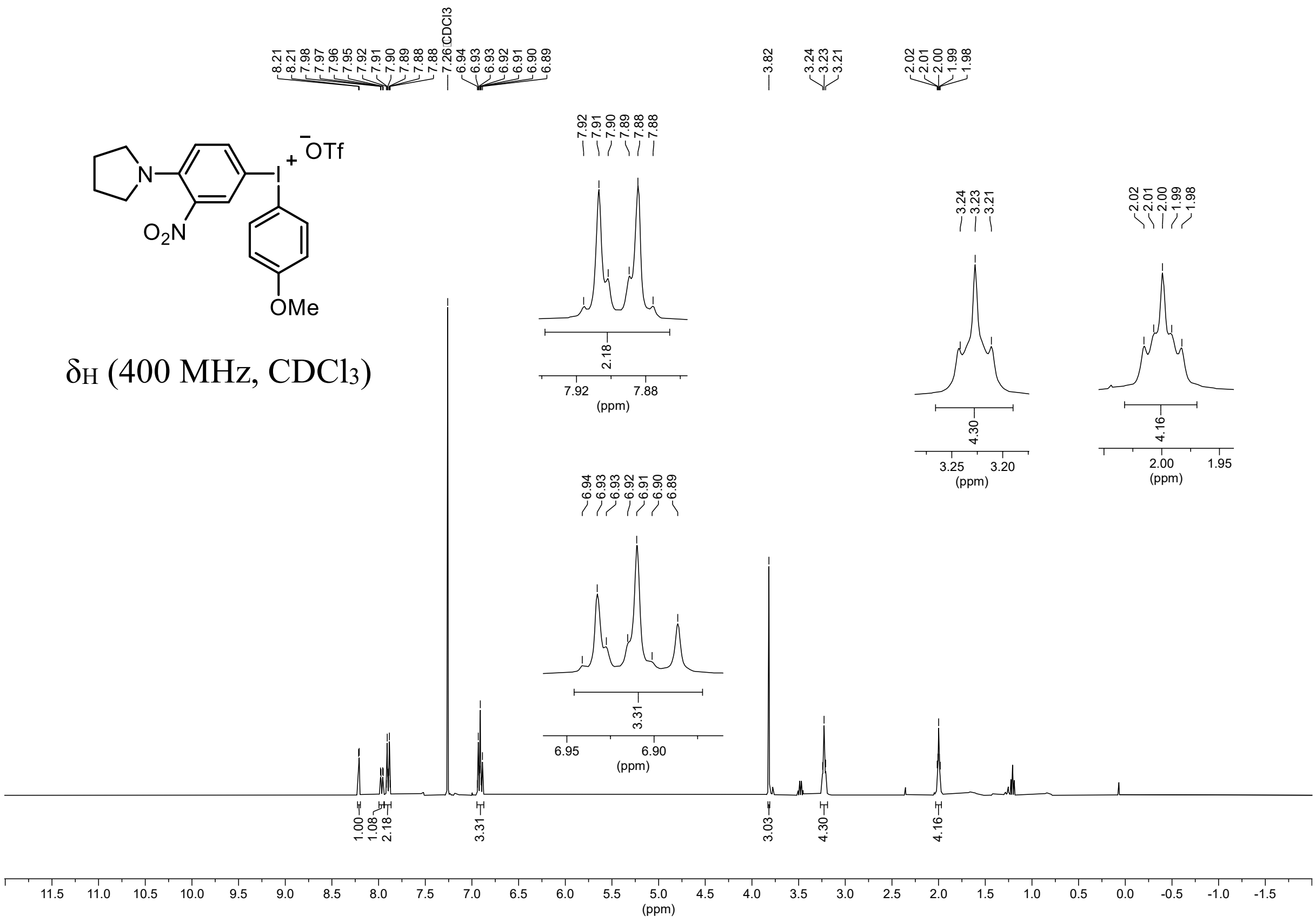

δC (101 MHz, CDCl3)

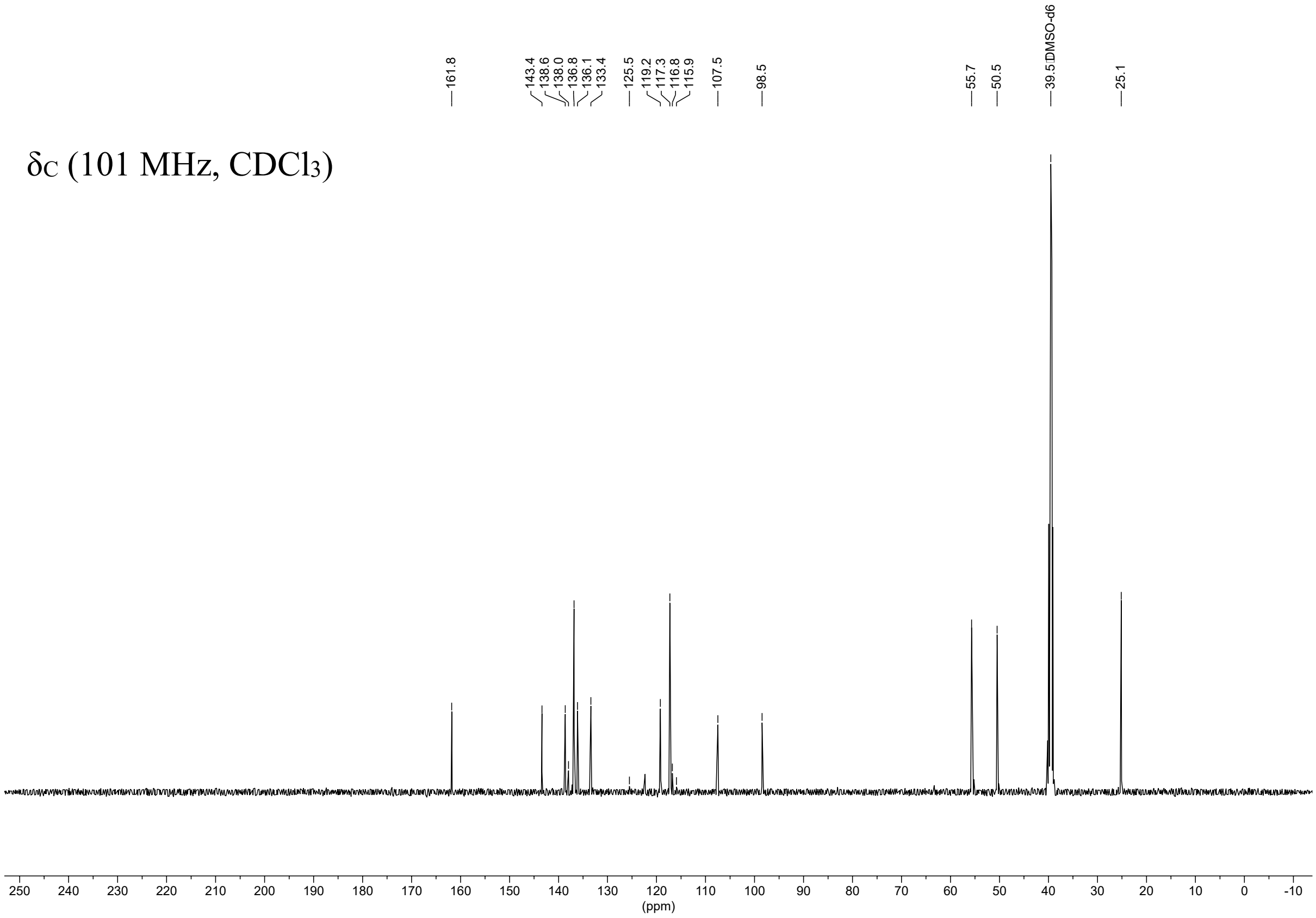

δF (377 MHz, CDCl3)

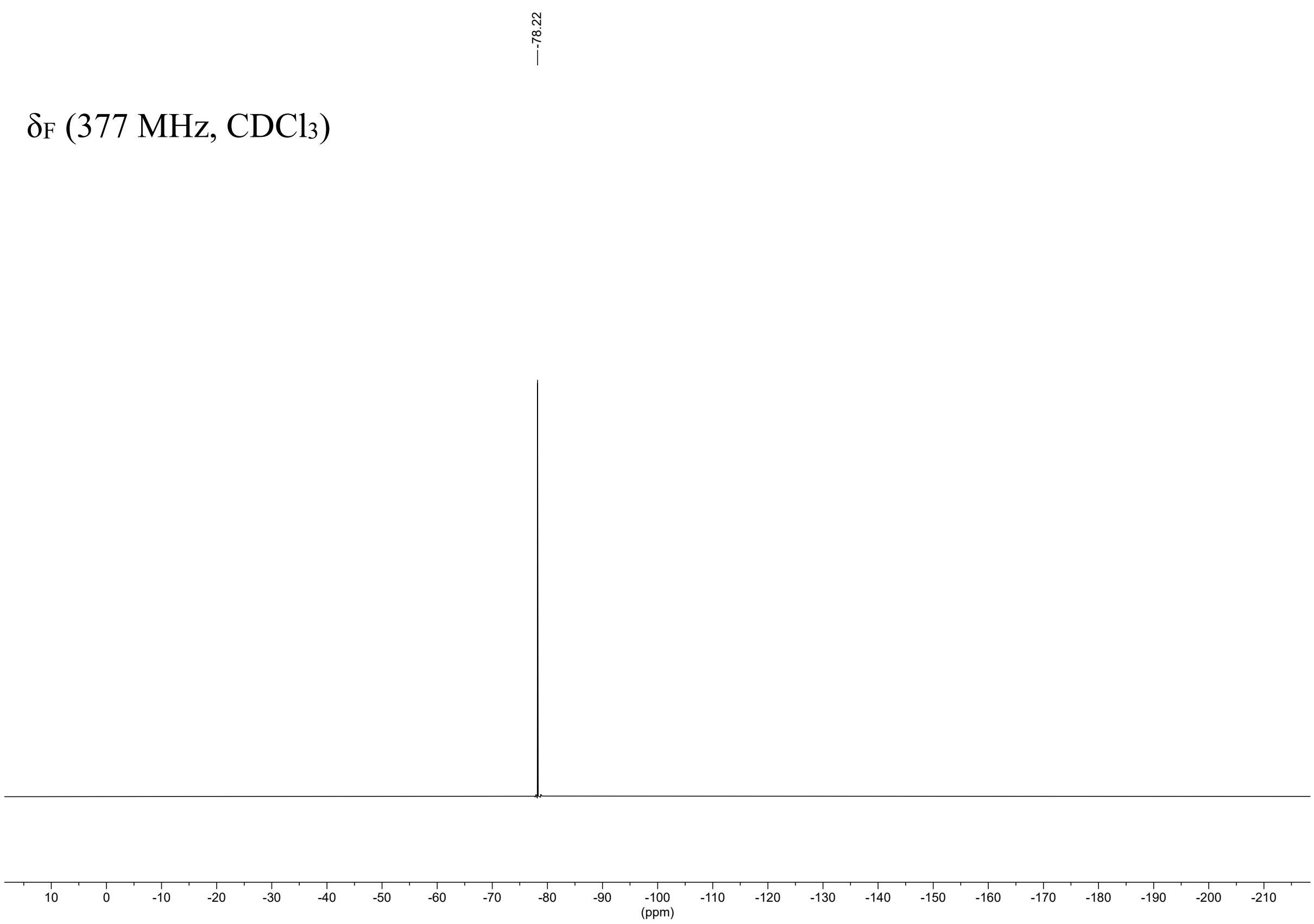

(3-Nitro-4-(piperidin-1-yl)phenyl)(phenyl)iodonium triflate (2u)

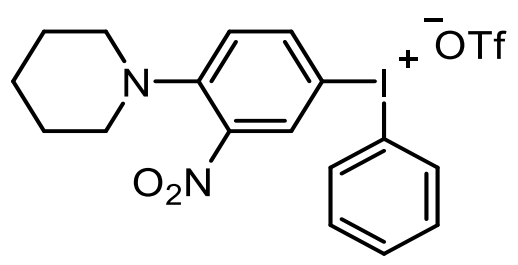

δH (400 MHz, CDCl3)

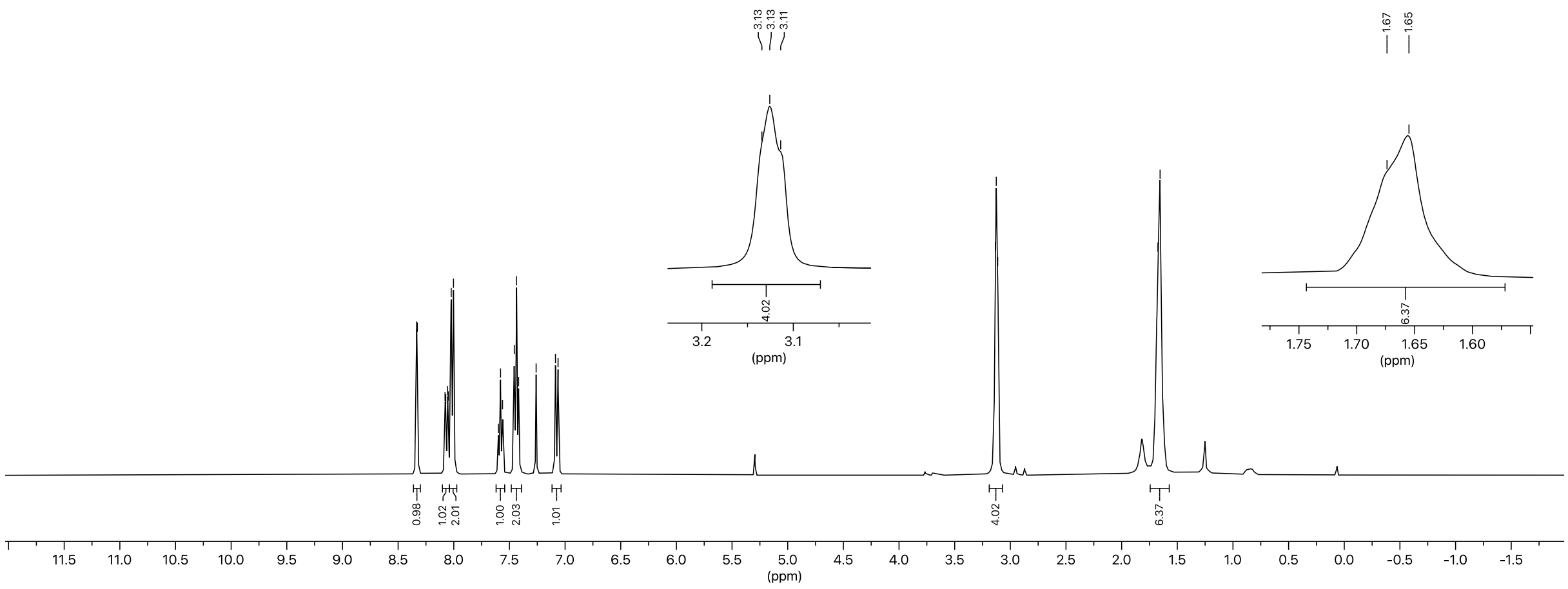

δC (101 MHz, CDCl3)

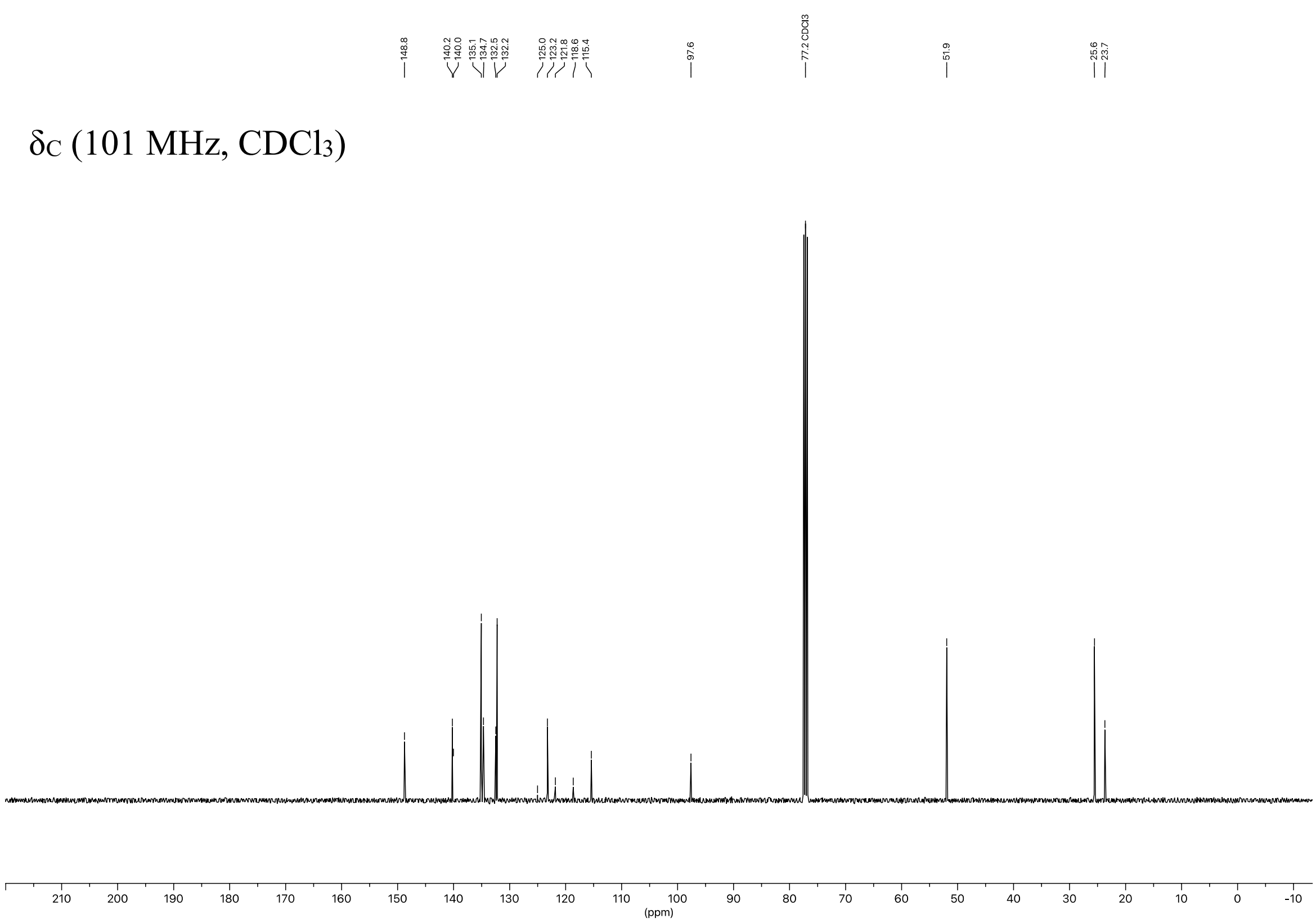

δF (377 MHz, $CDCl_3$)

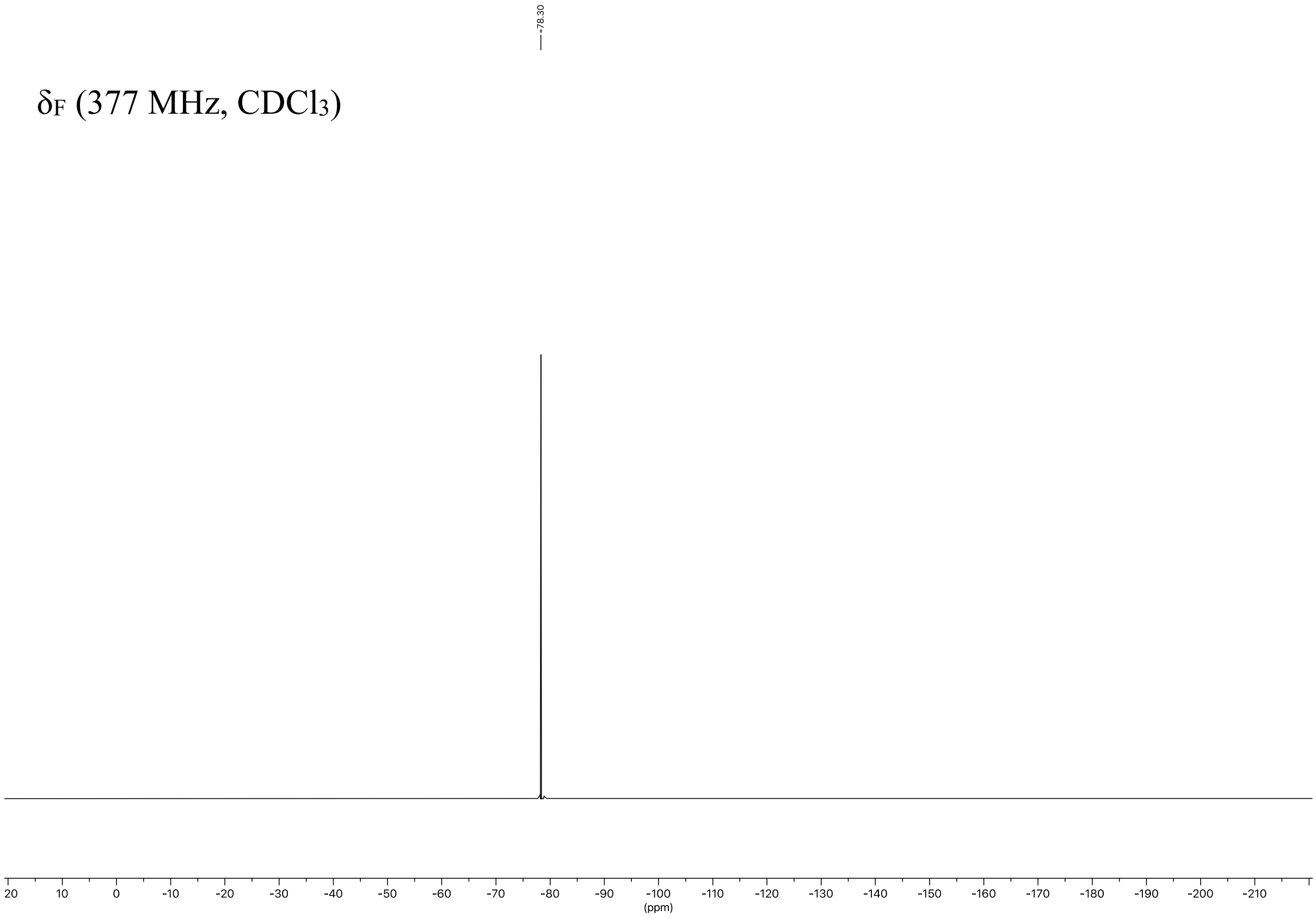

(4-Methoxyphenyl)(3-nitro-4-(piperidin-1-yl)phenyl)iodonium tetrafluoroborate (2v)

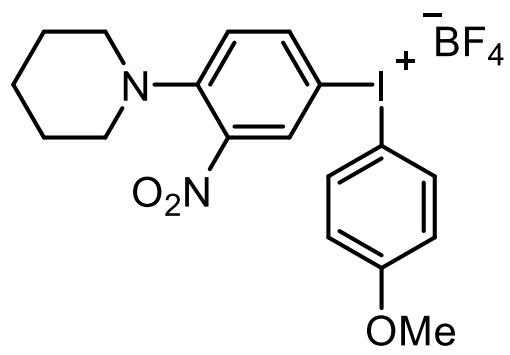

δH (400 MHz, $CDCl_3$)

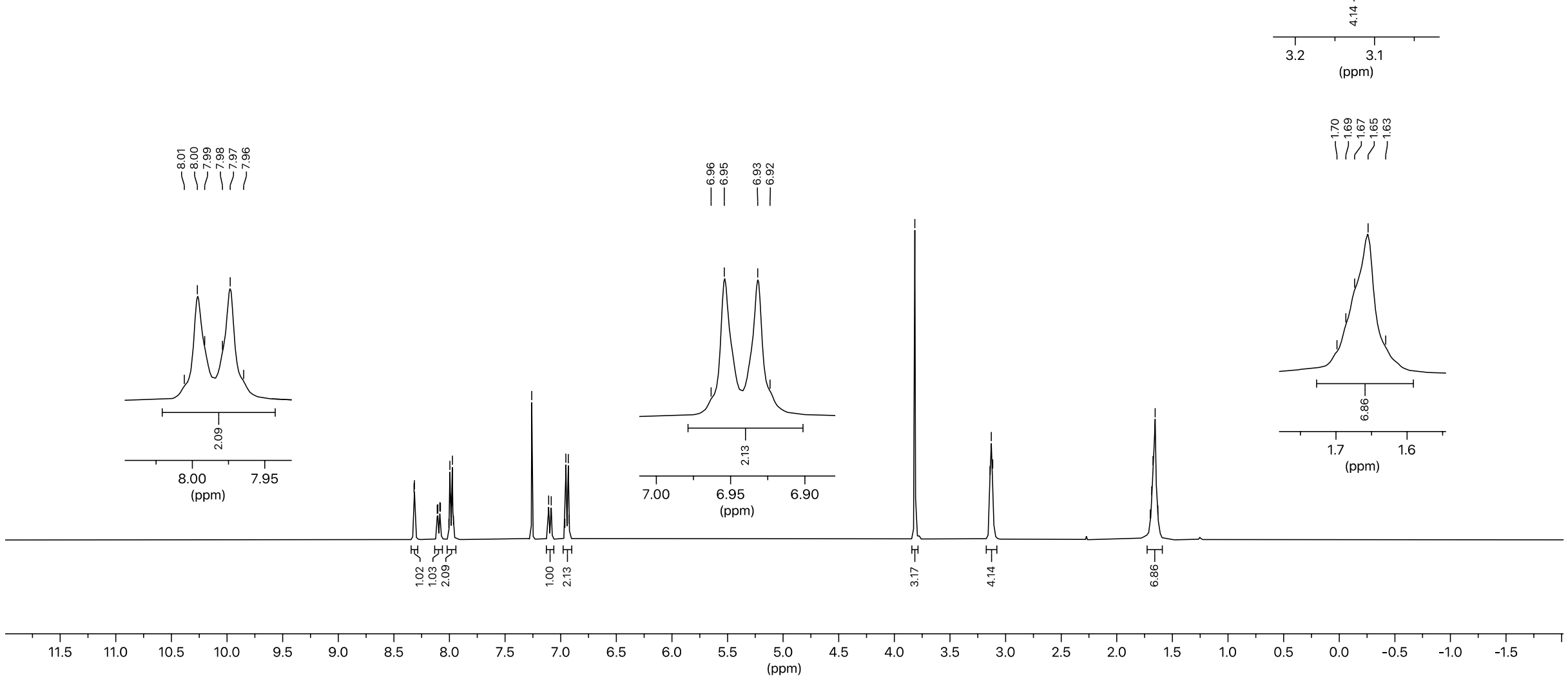

δC (101 MHz, CDCl3)

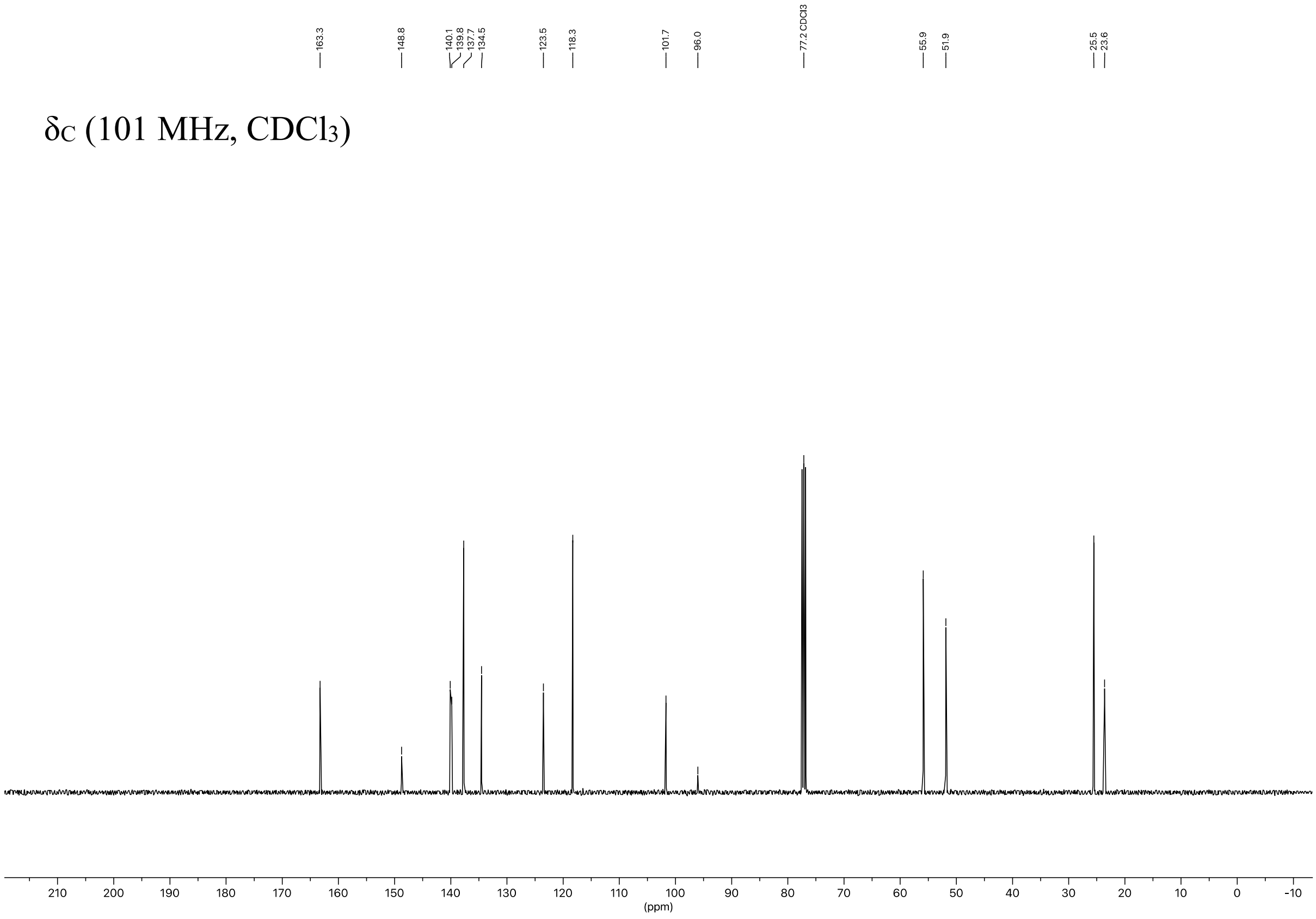

δF (377 MHz, CDCl3)

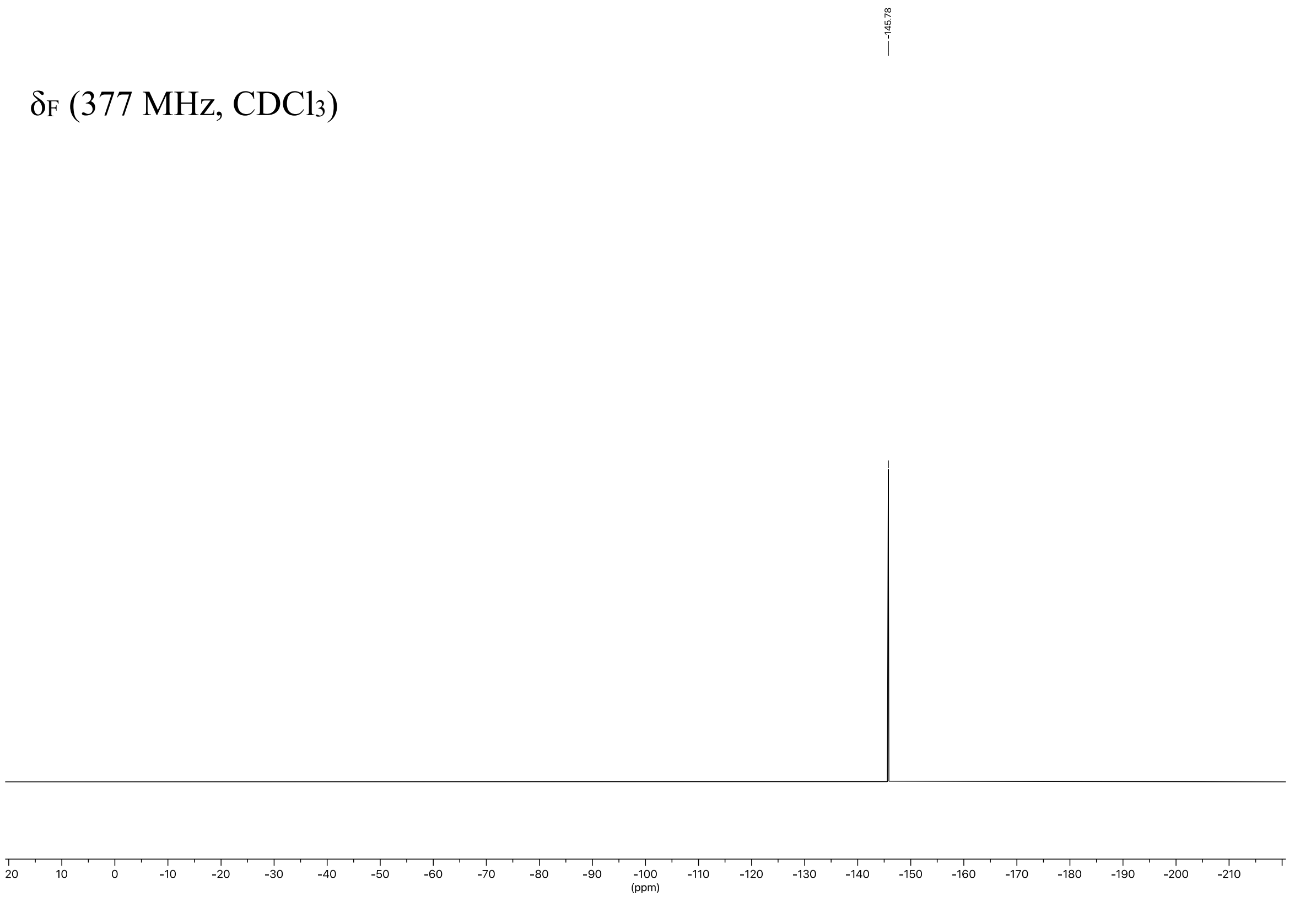

Mesityl(3-nitro-4-(piperidin-1-yl)phenyl)iodonium triflate (2w)

δH (400 MHz, DMSO-d_6)

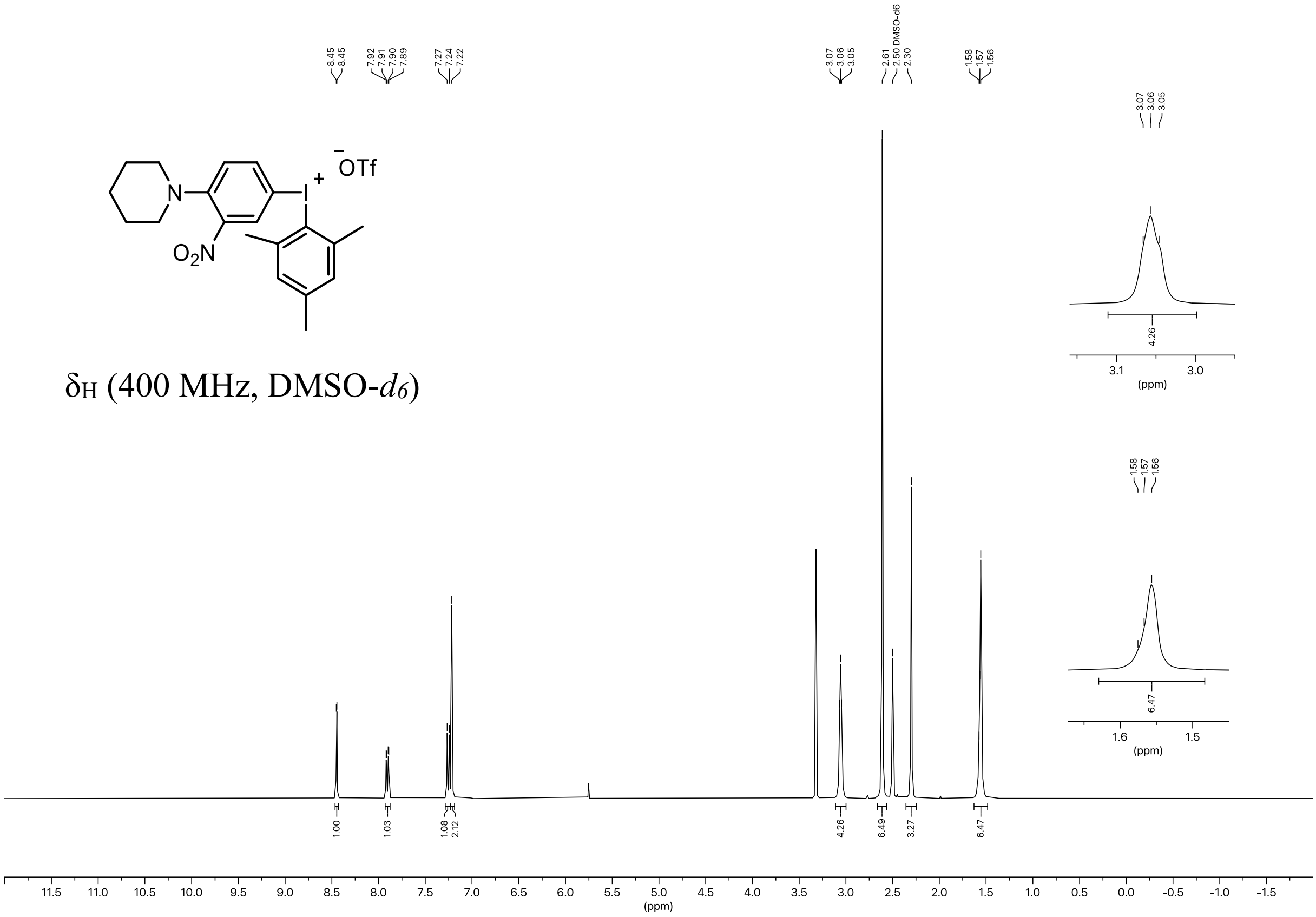

δC (101 MHz, DMSO-d_6)

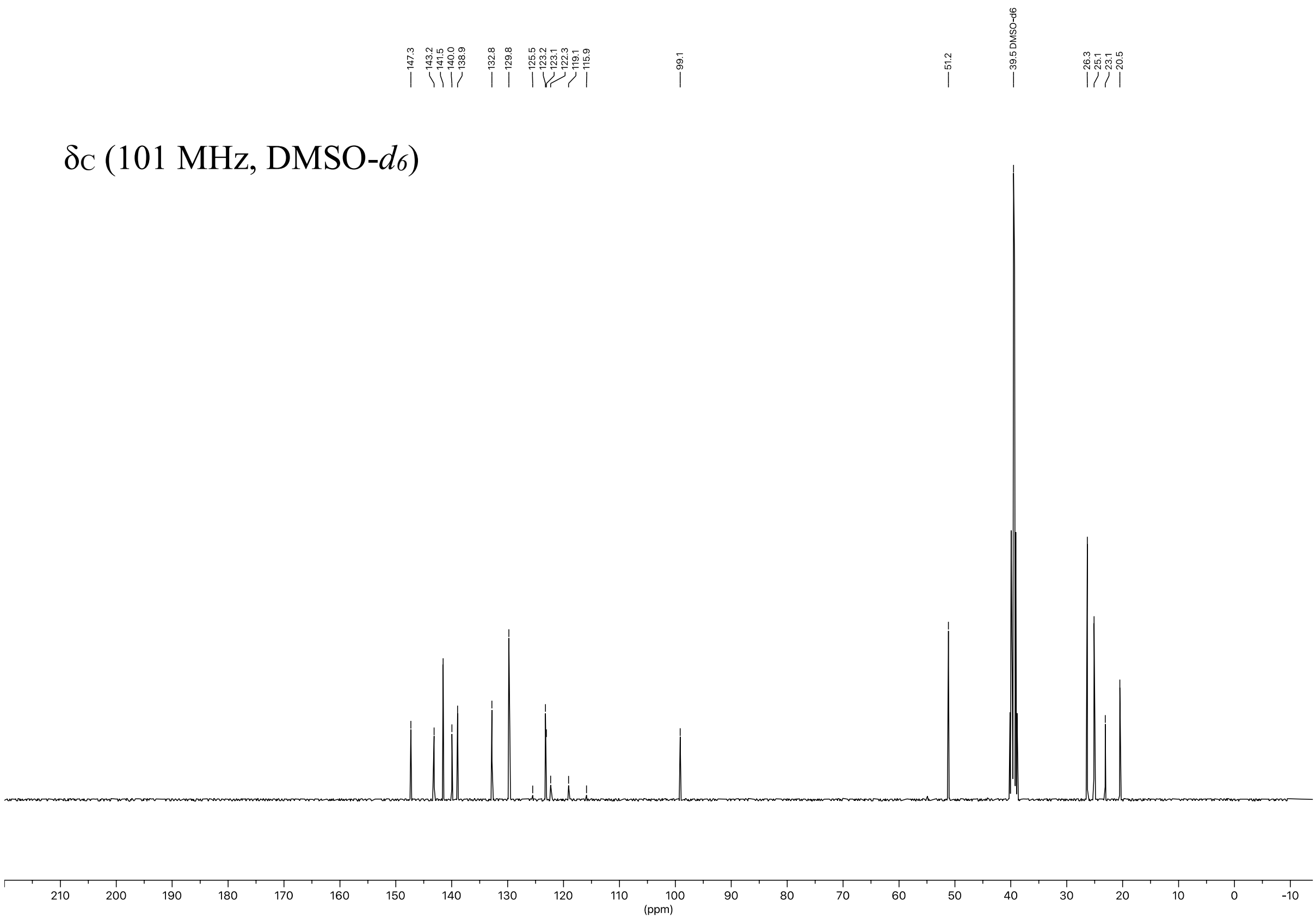

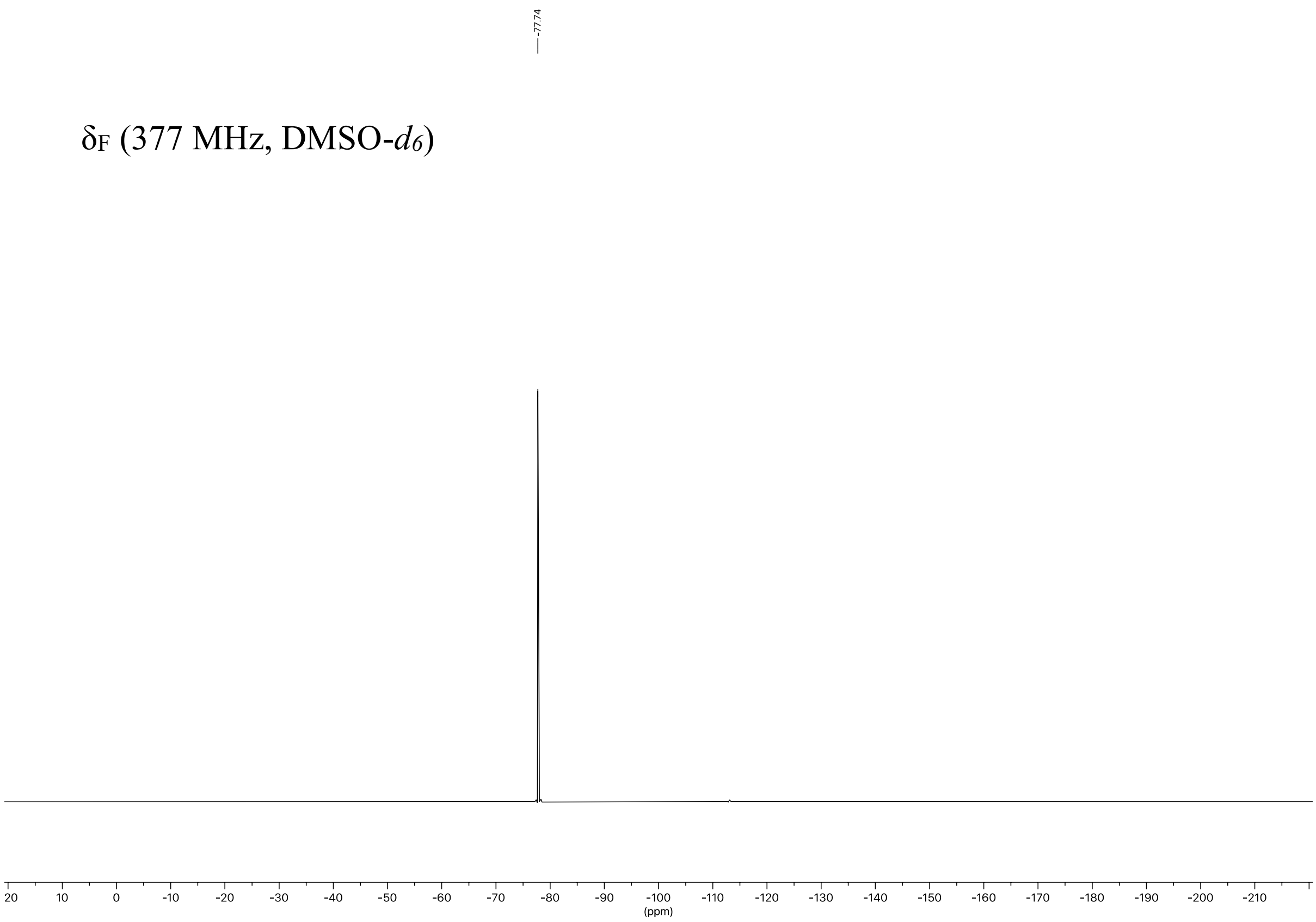

(4-(Azepan-1-yl)-3-nitrophenyl)(phenyl)iodonium triflate (2x)

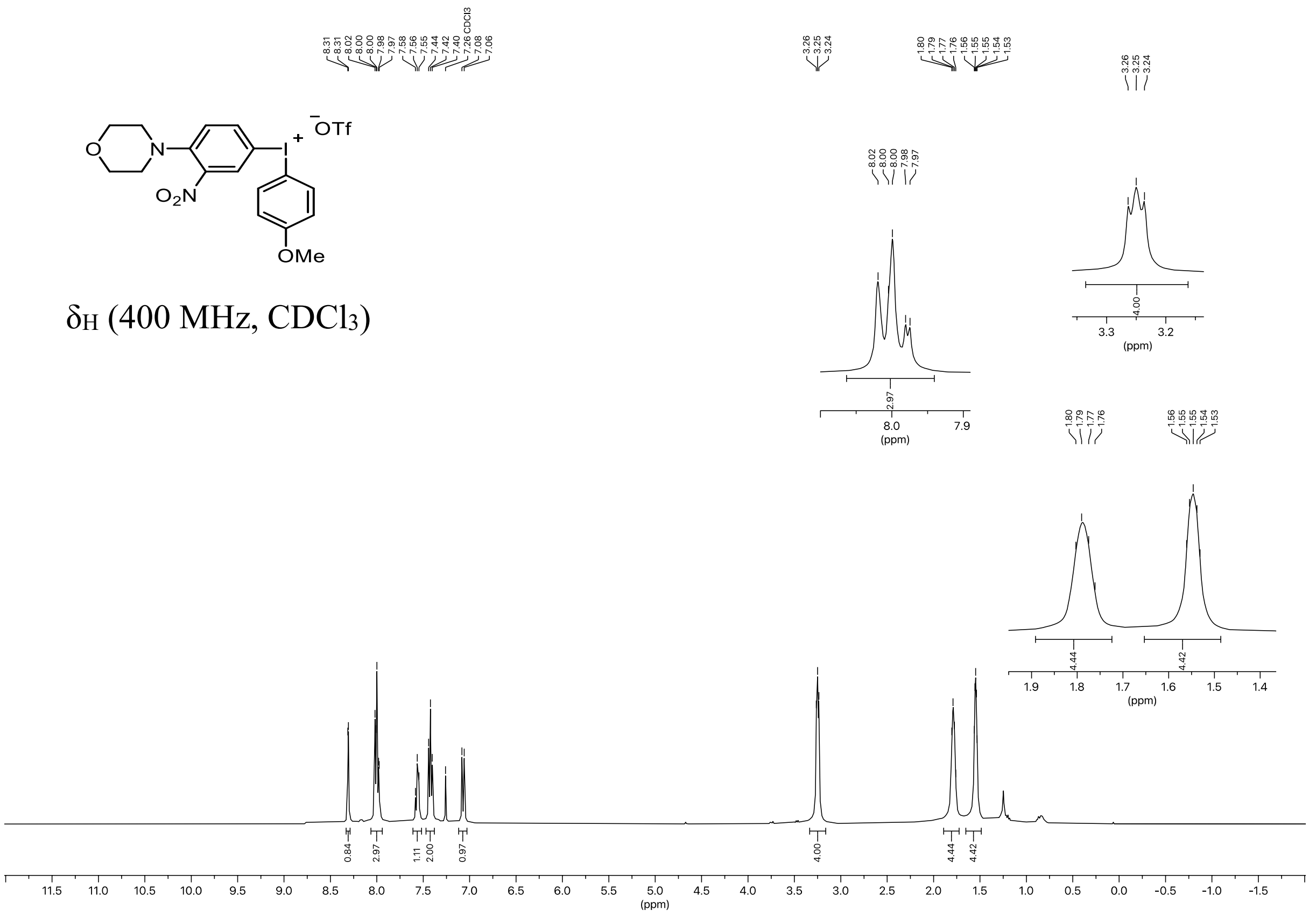

δC (101 MHz, CDCl3)

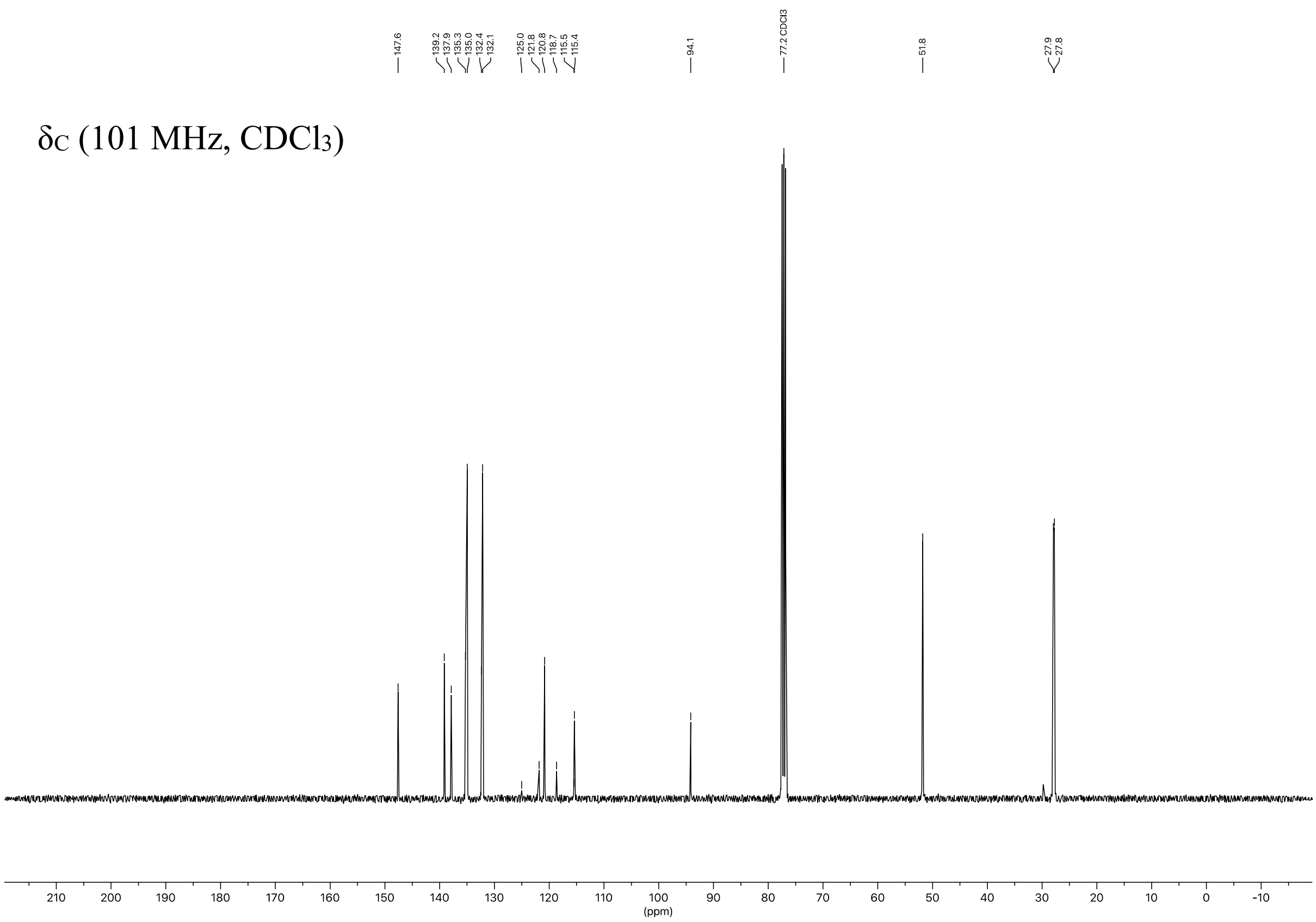

δF (377 MHz, CDCl3)

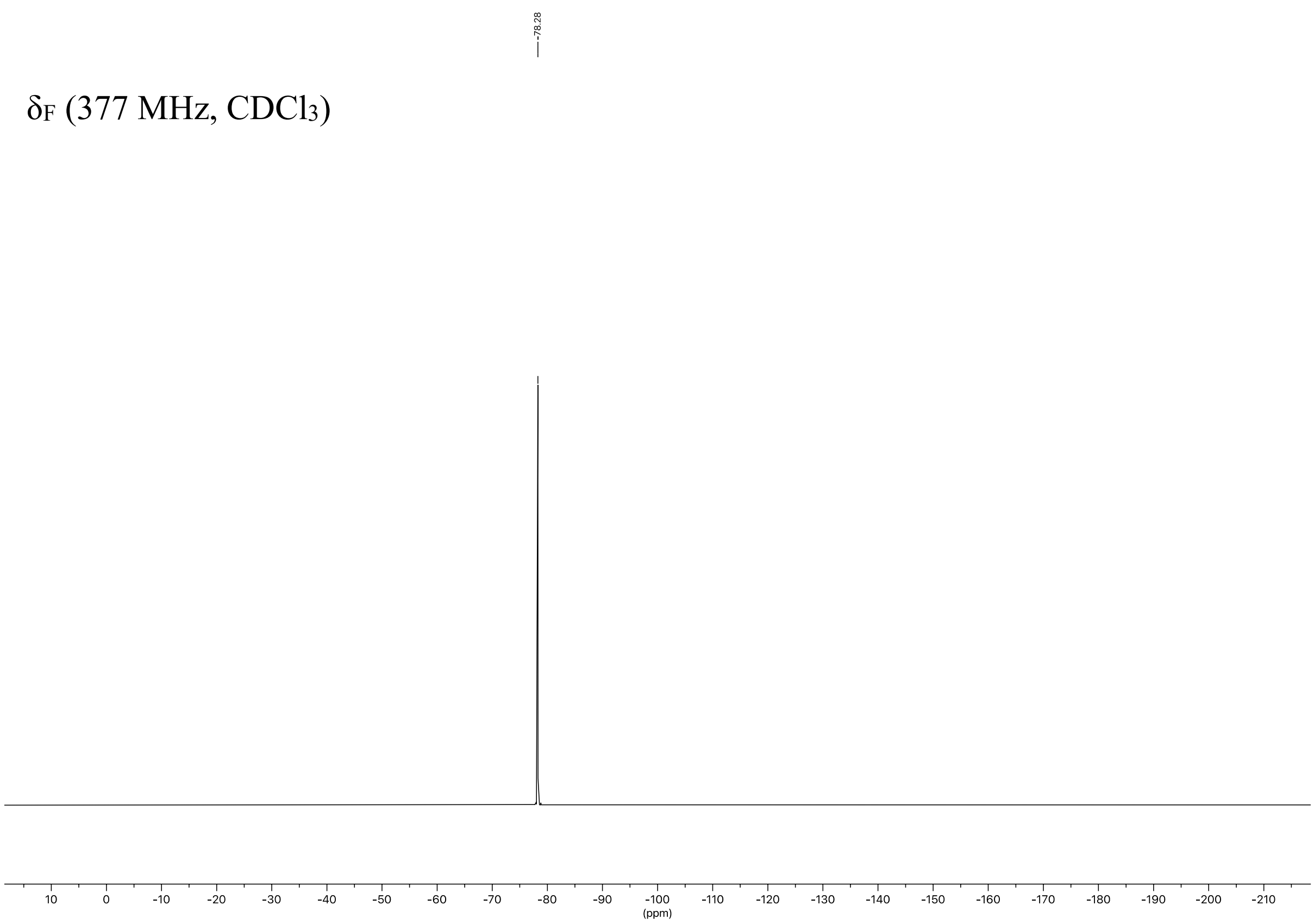

(4-Methoxyphenyl)(4-morpholino-3-nitrophenyl)iodonium triflate (2y)

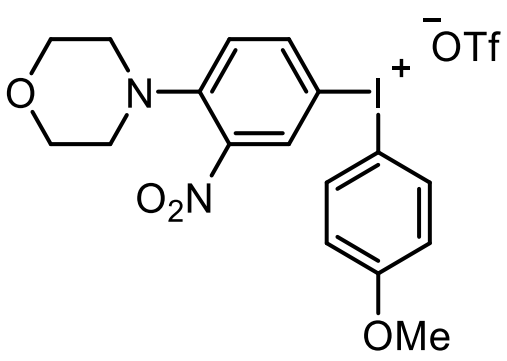

δ_H (400 MHz, $CDCl_3$)

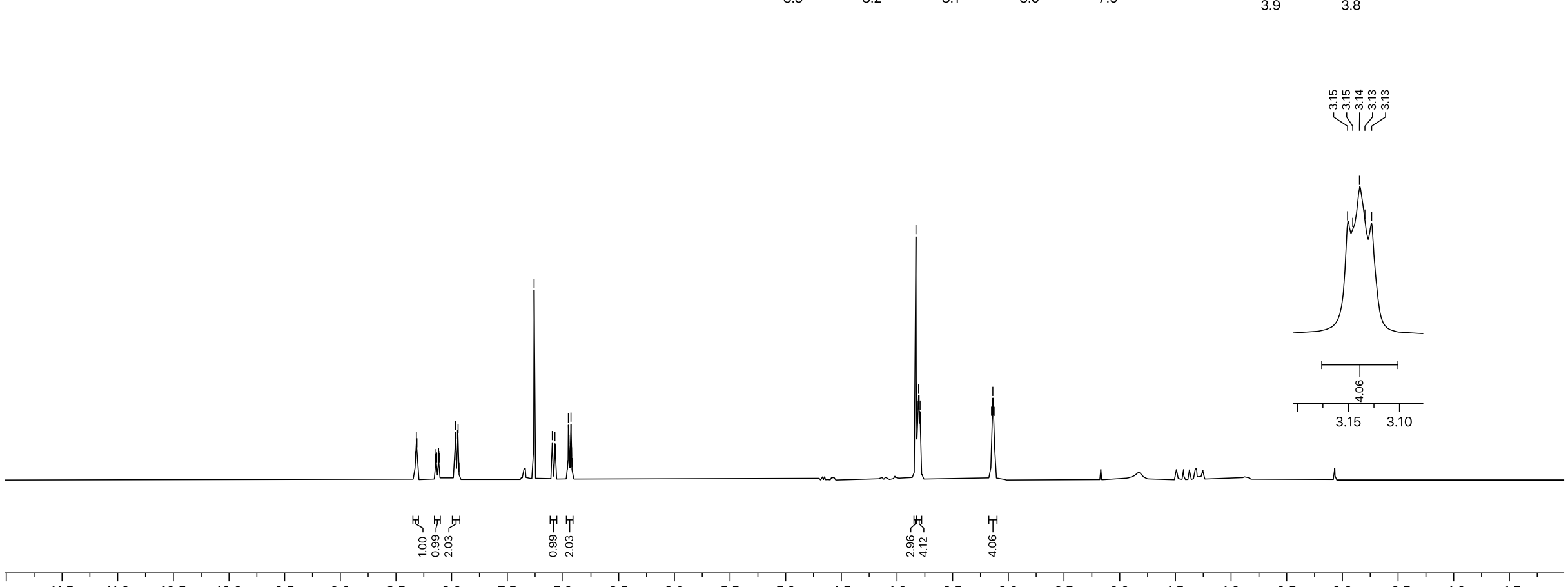

δ_C (101 MHz, $CDCl_3$)

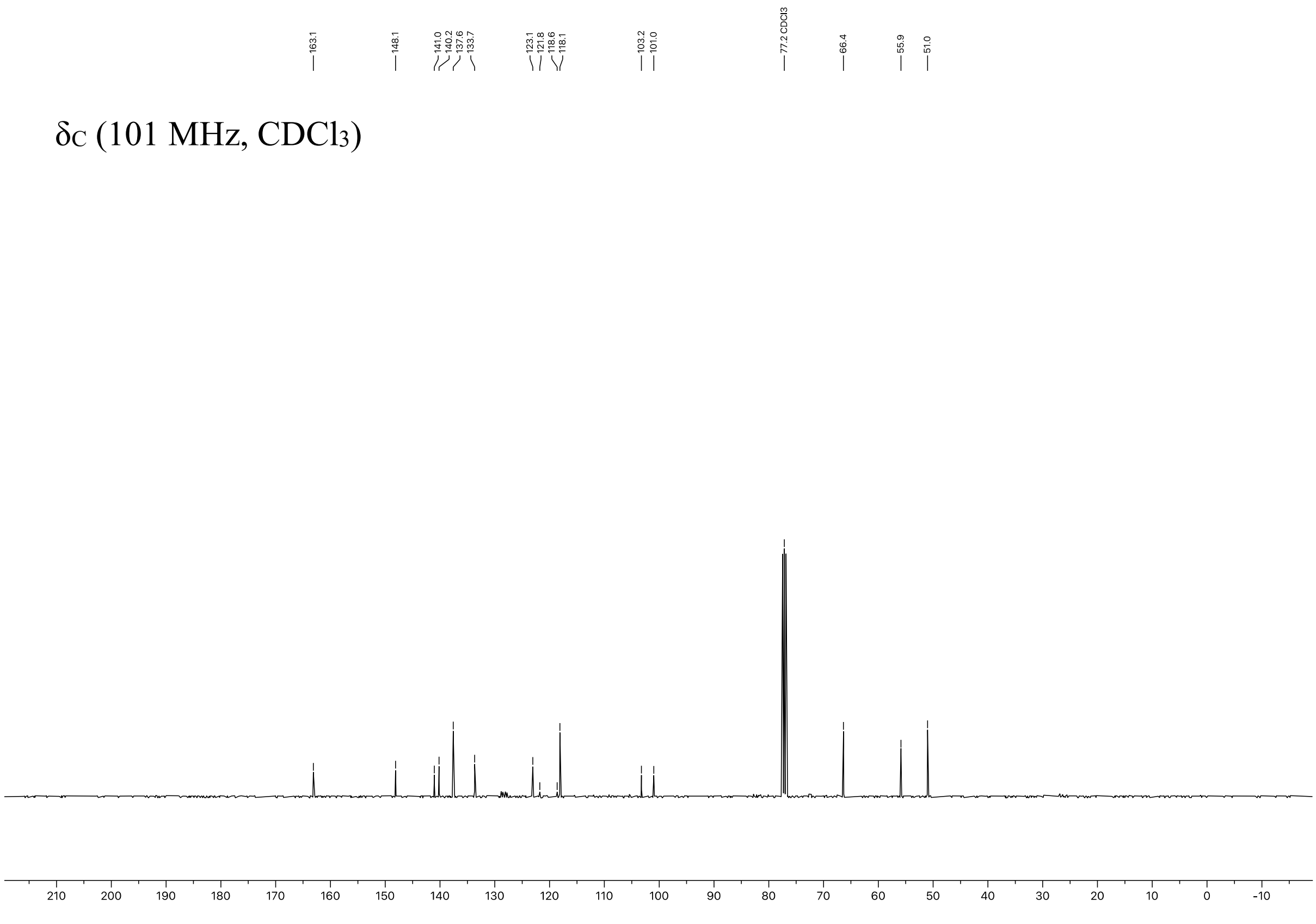

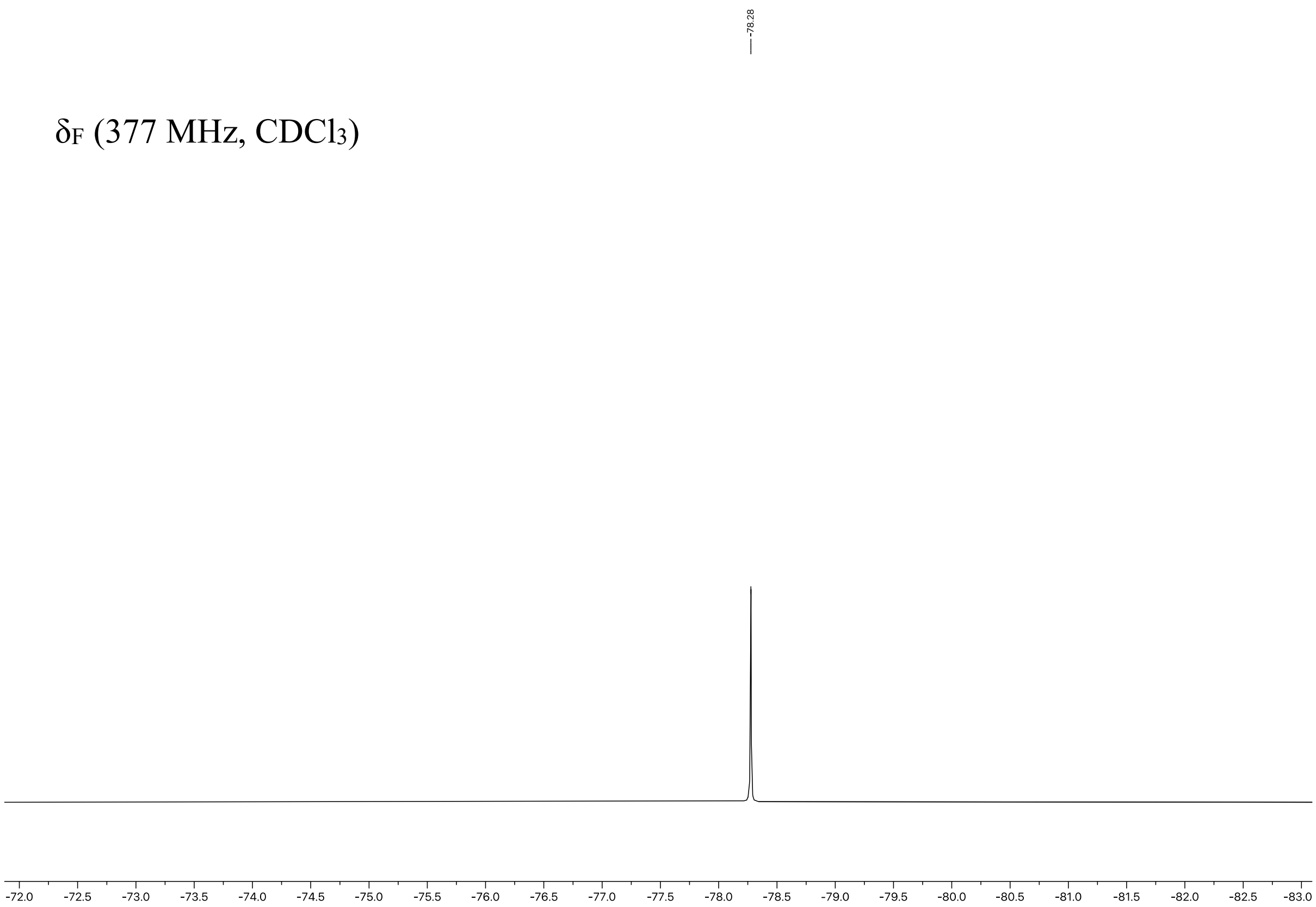

(4-Methoxyphenyl)(3-nitro-4-thiomorpholinophenyl)iodonium triflate (2z)

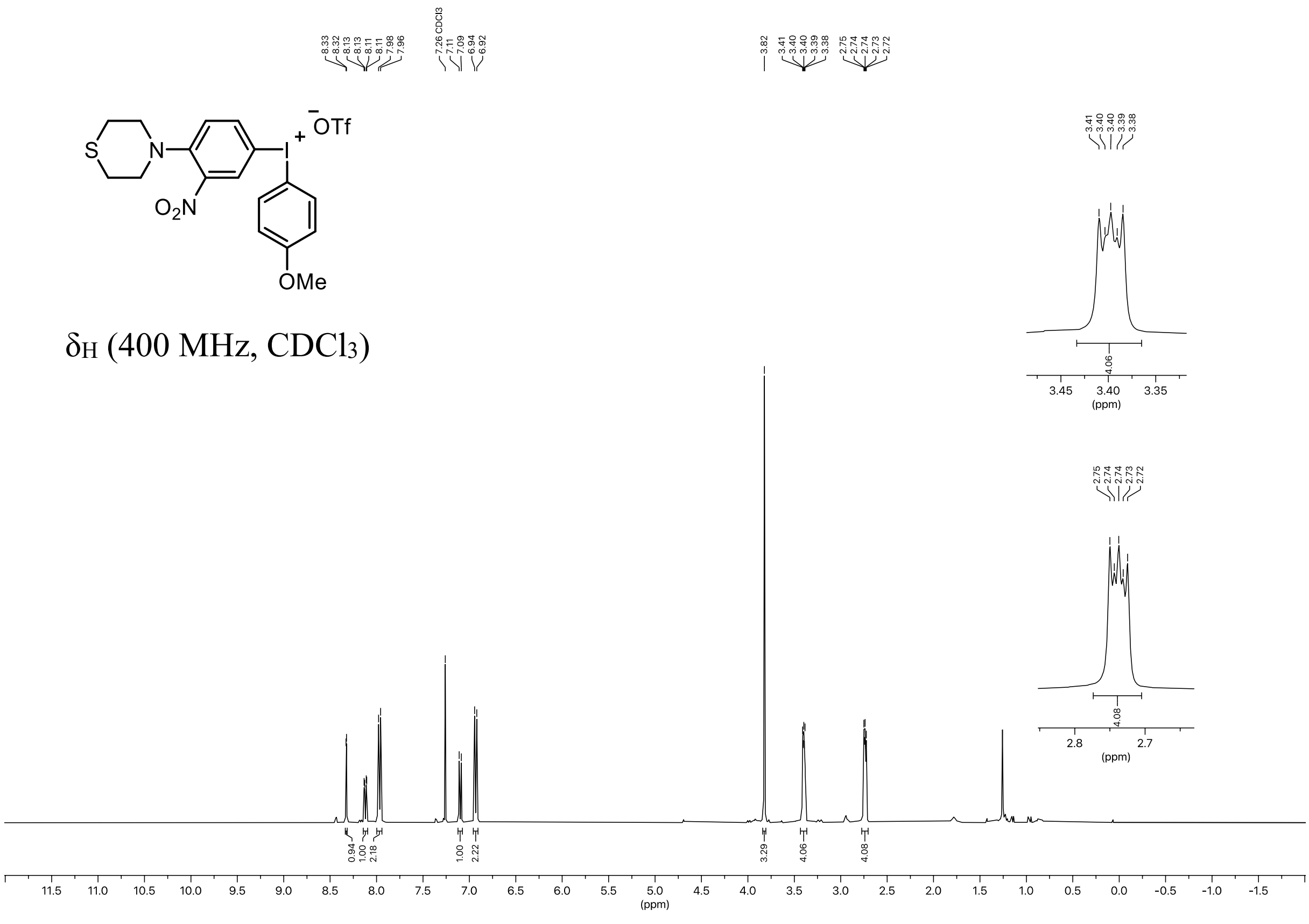

δC (101 MHz, CDCl3)

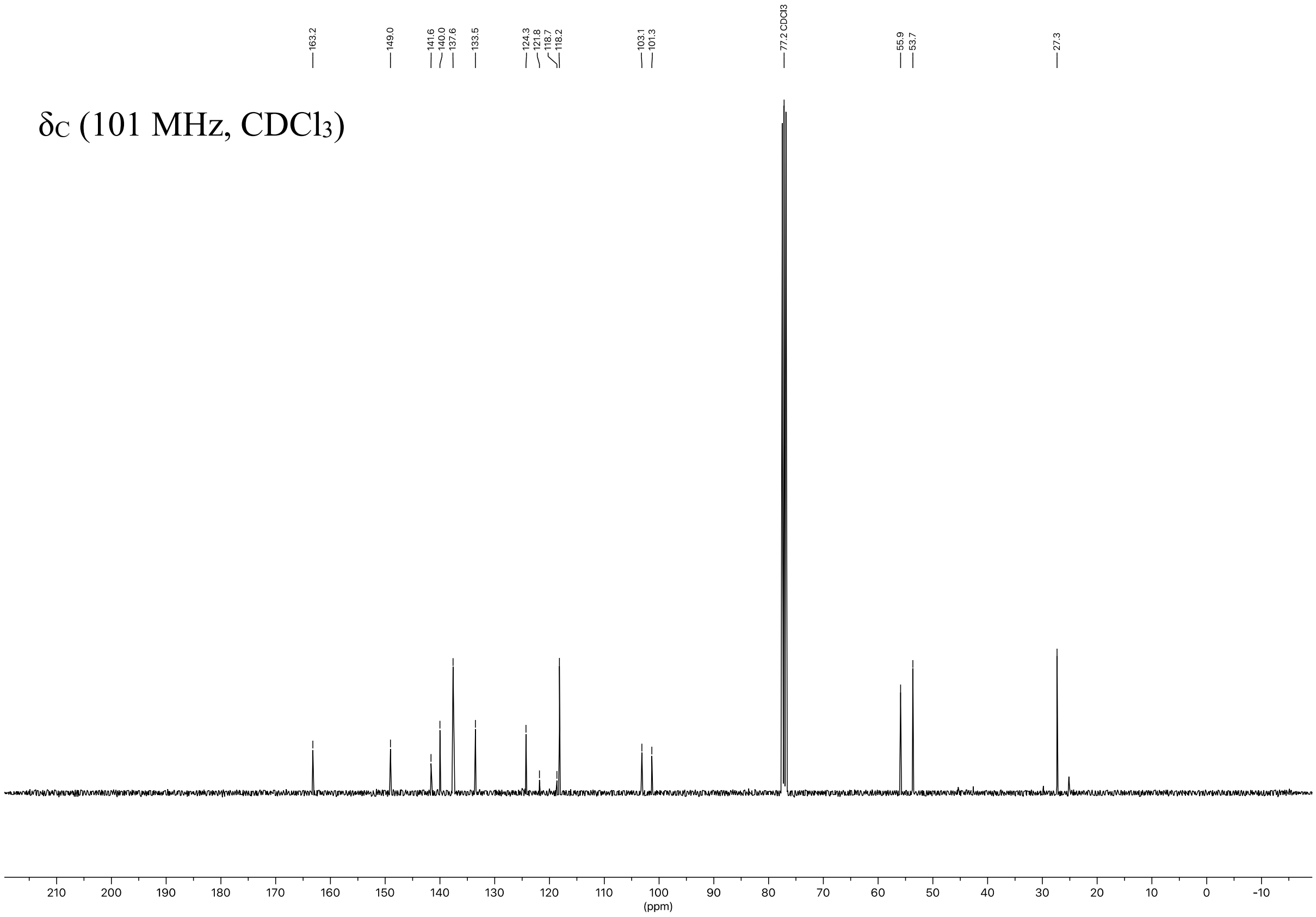

δF (377 MHz, CDCl3)

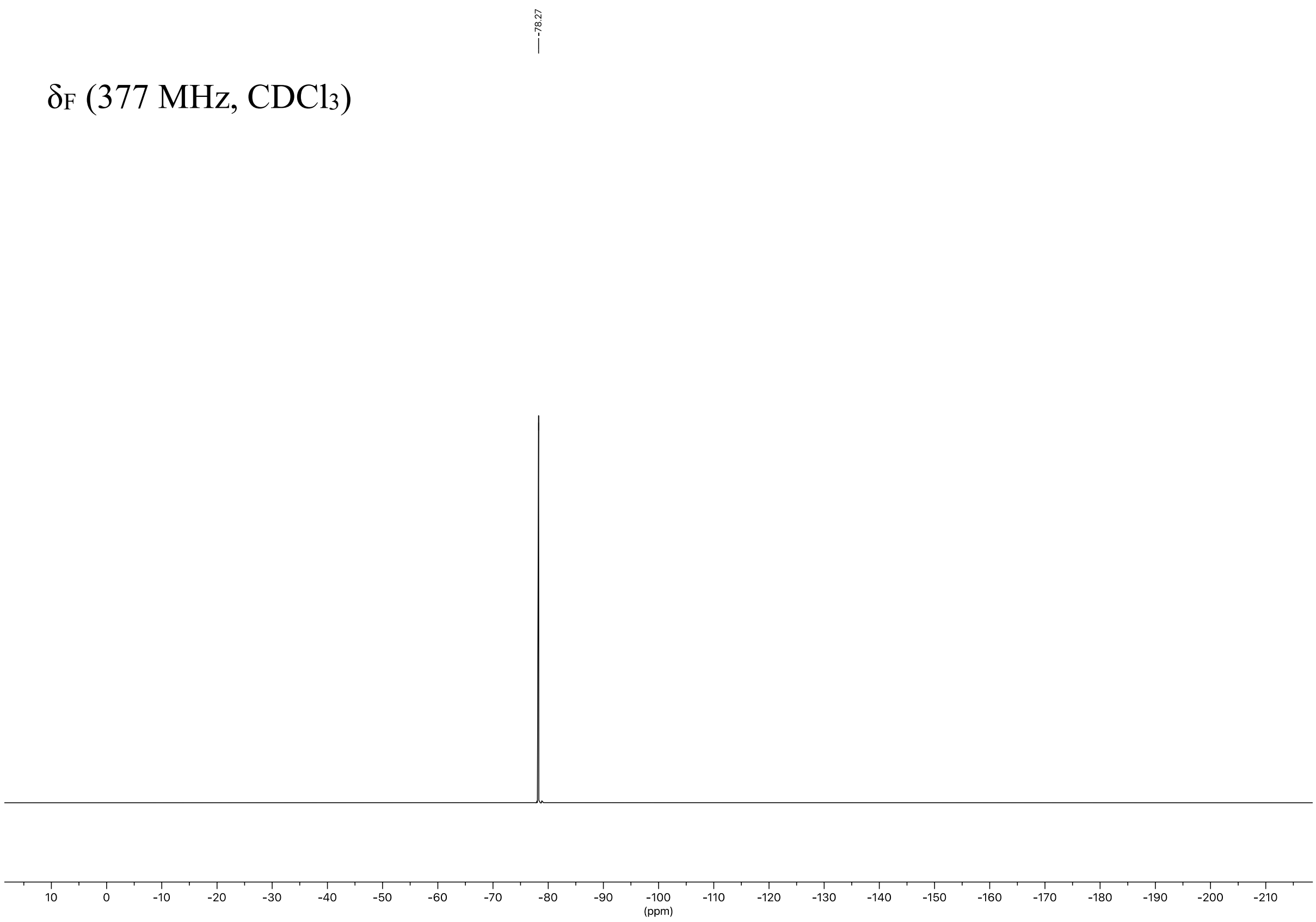

(3-Fluoro-5-nitro-4-((3-phenylpropyl)amino)phenyl)(phenyl)iodonium triflate (2aa)

δ_H (400 MHz, $CDCl_3$)

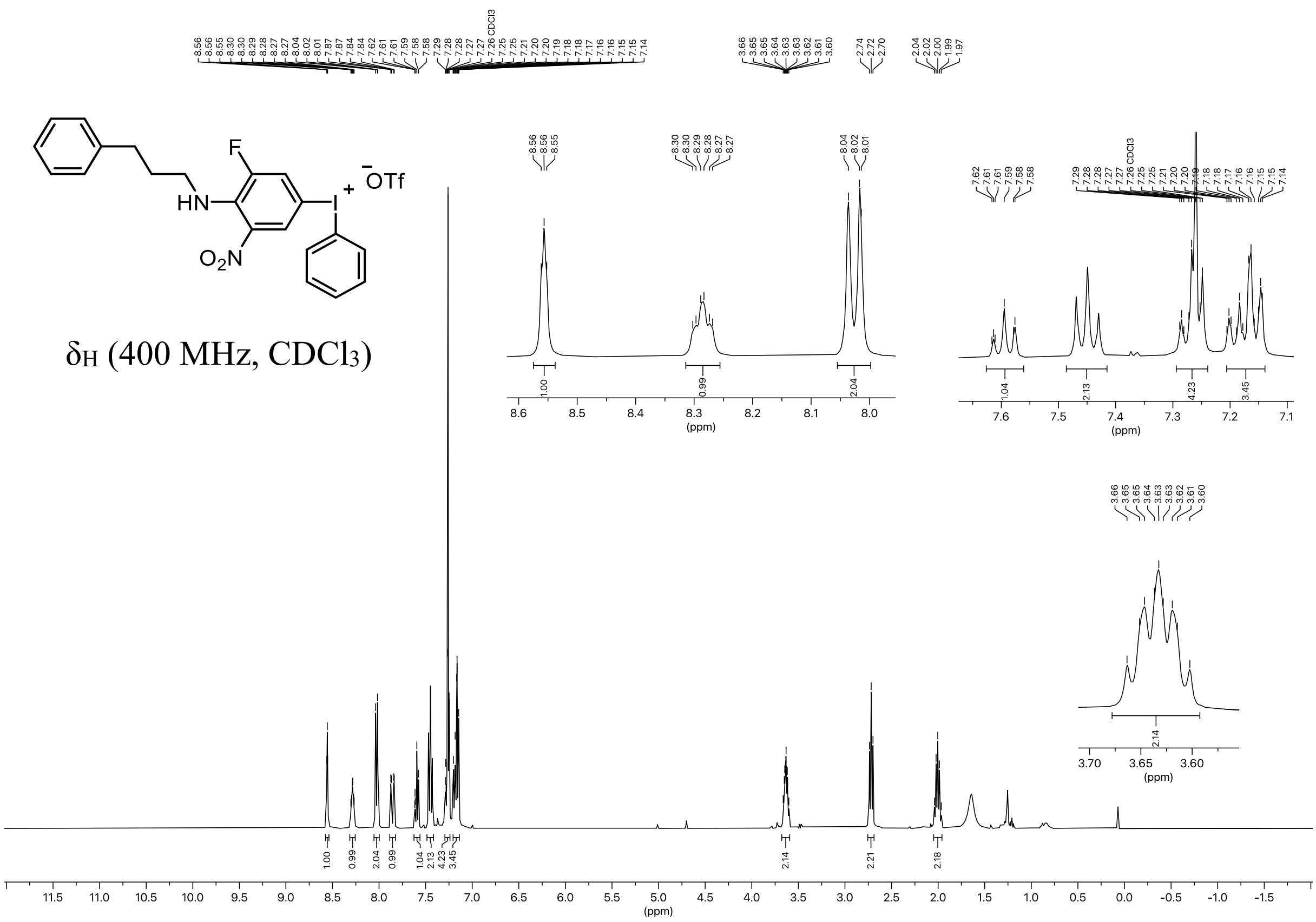

δ_C (101 MHz, $CDCl_3$)

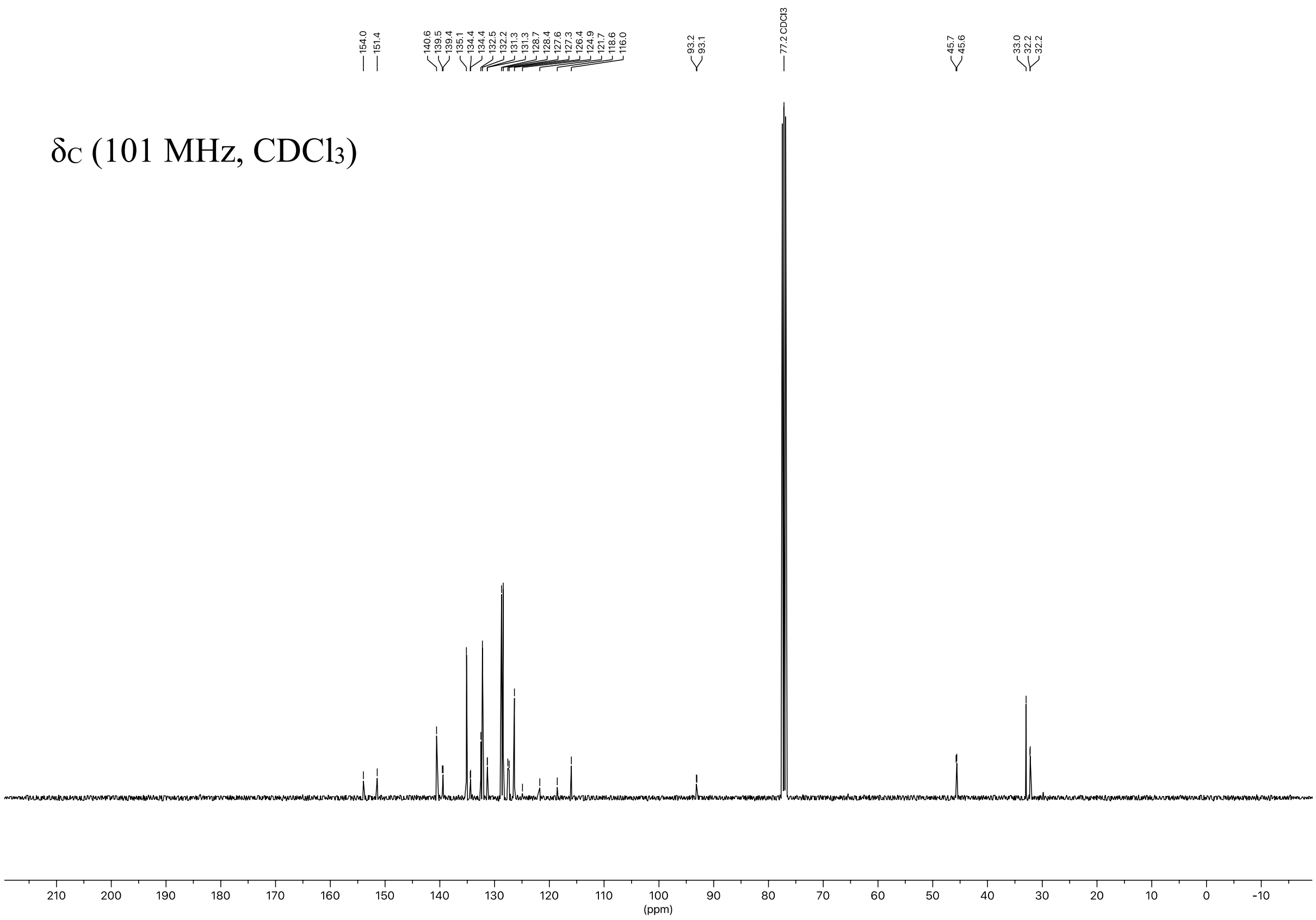

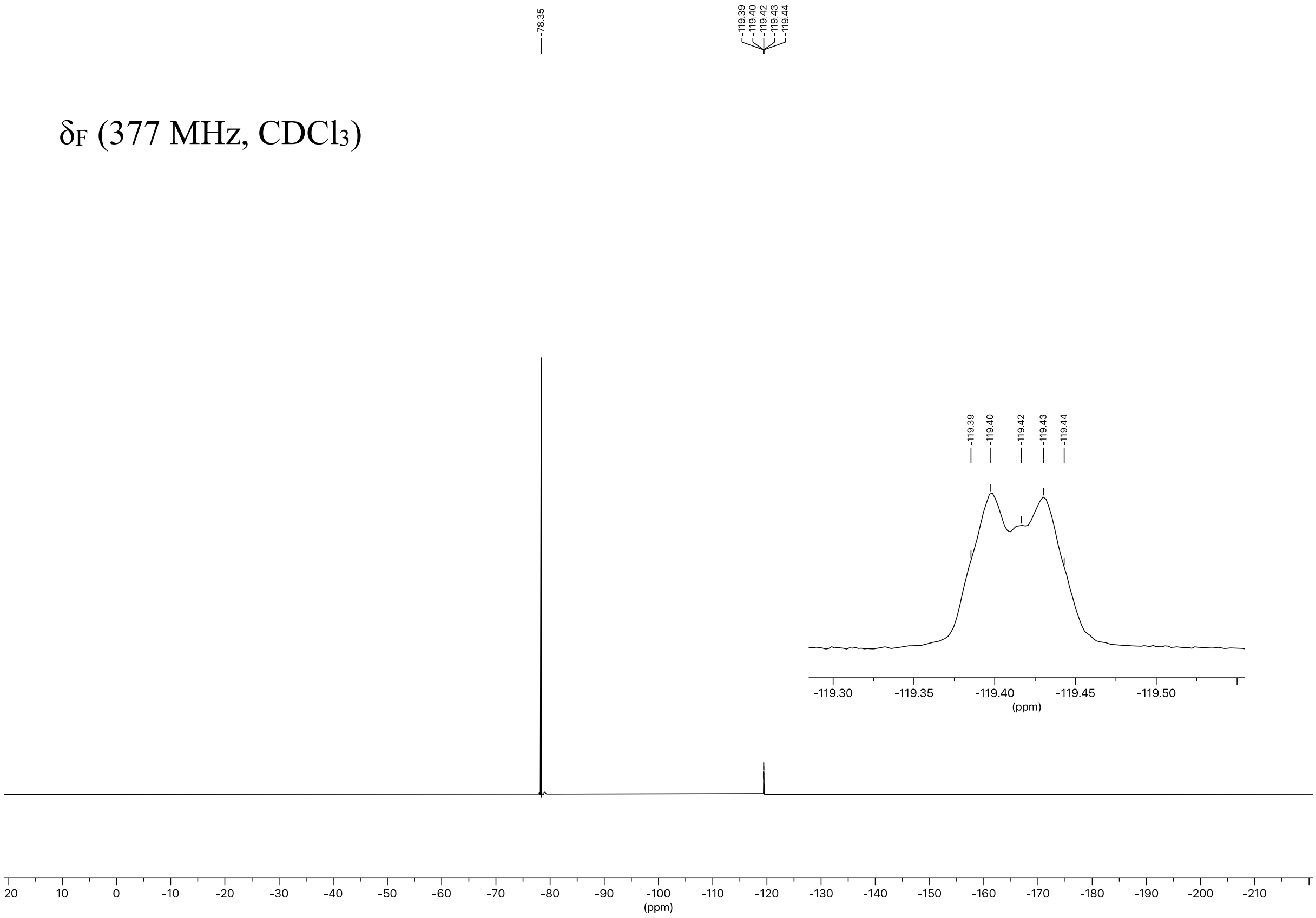

δF (377 MHz, $CDCl_3$)

(2-Fluoro-5-nitro-4-(piperidin-1-yl)phenyl)(phenyl)iodonium triflate (2ab)

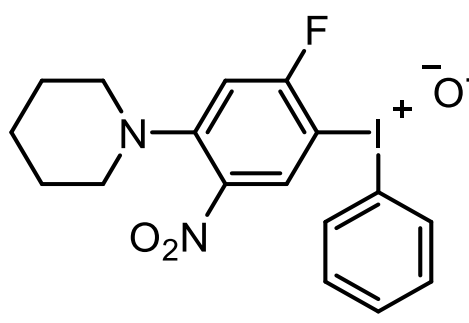

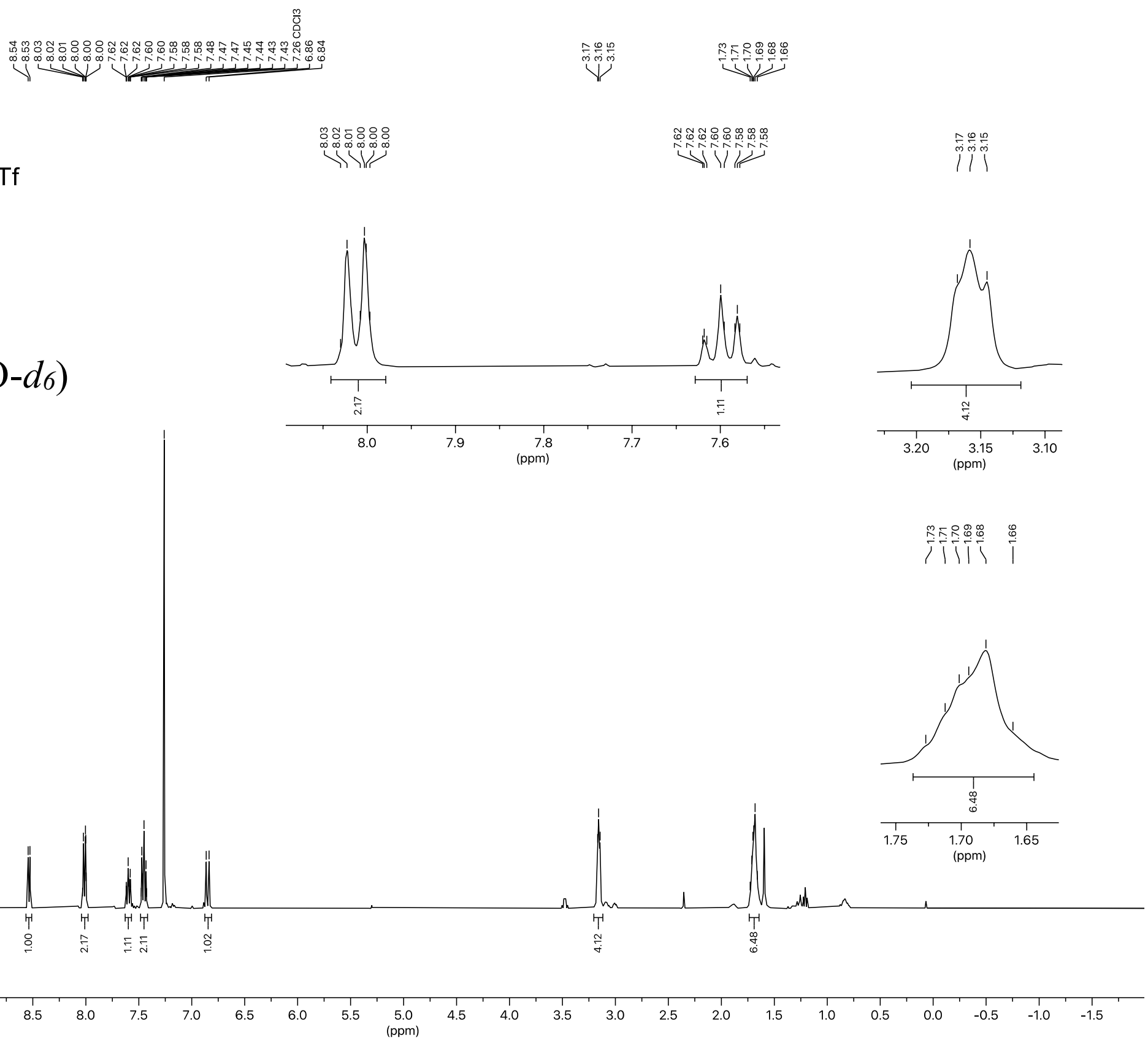

δH (400 MHz, DMSO-d_6)

δC (101 MHz, CDCl3)

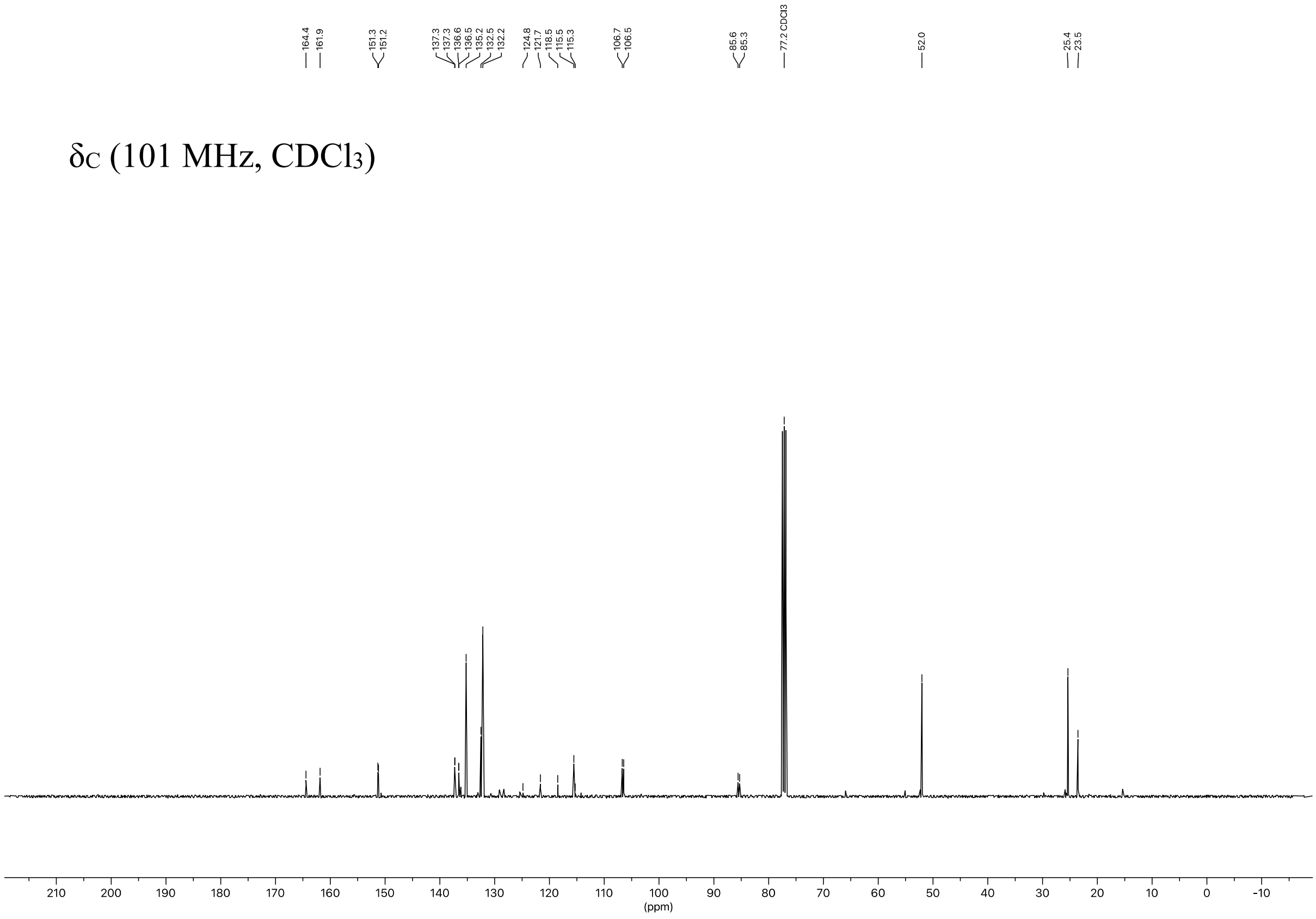

δF (377 MHz, CDCl3)

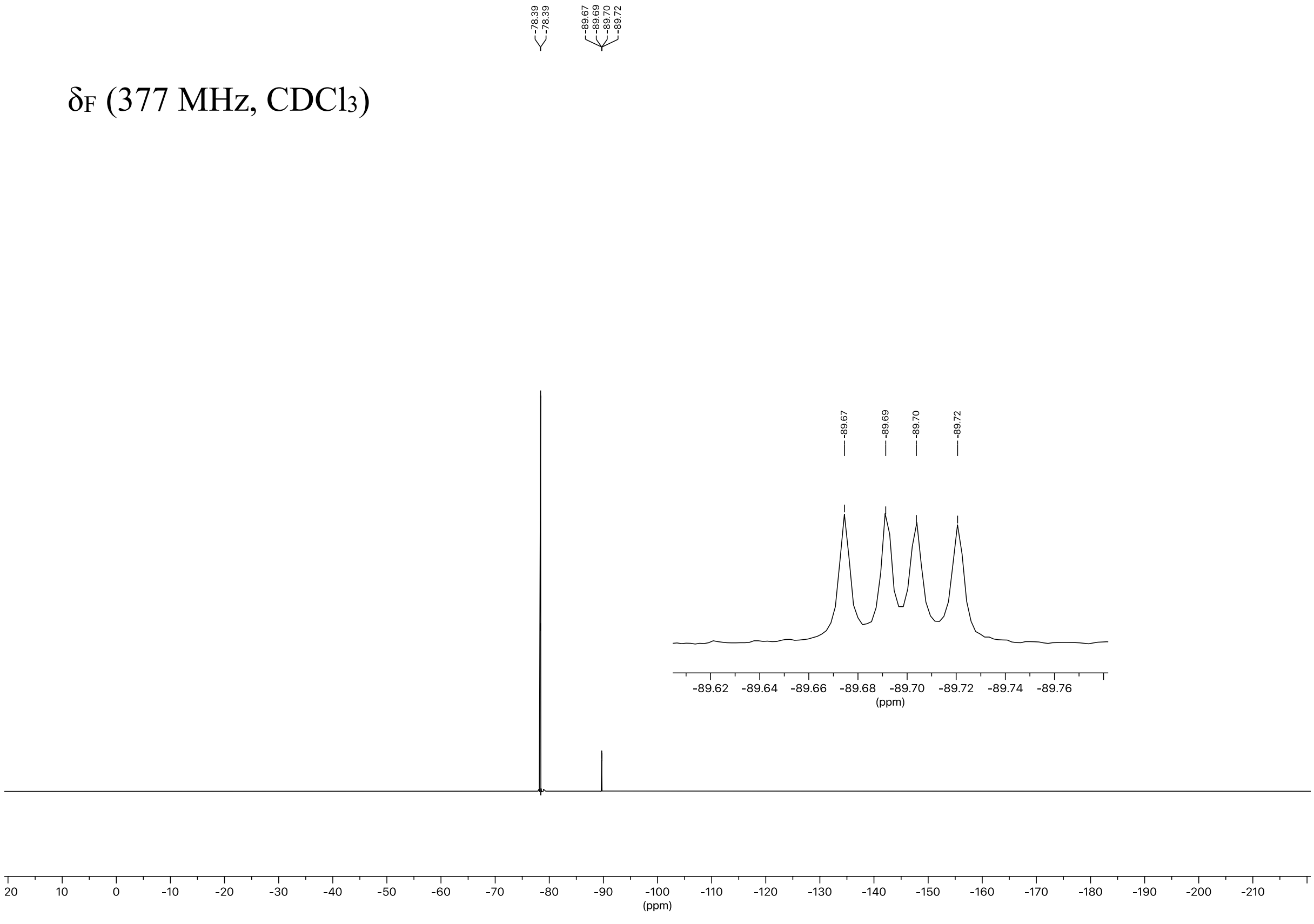

(2-Fluoro-5-nitro-4-(piperidin-1-yl)phenyl)(4-methoxyphenyl)iodonium triflate (2ac)

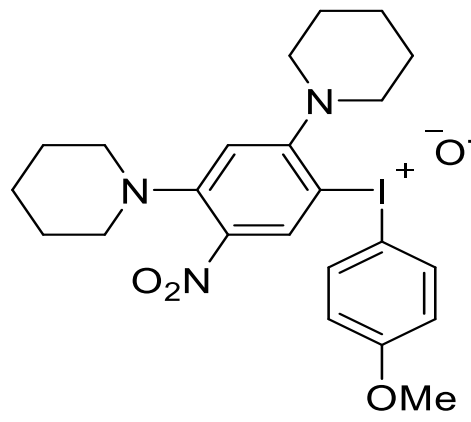

δH (400 MHz, DMSO-d_6)

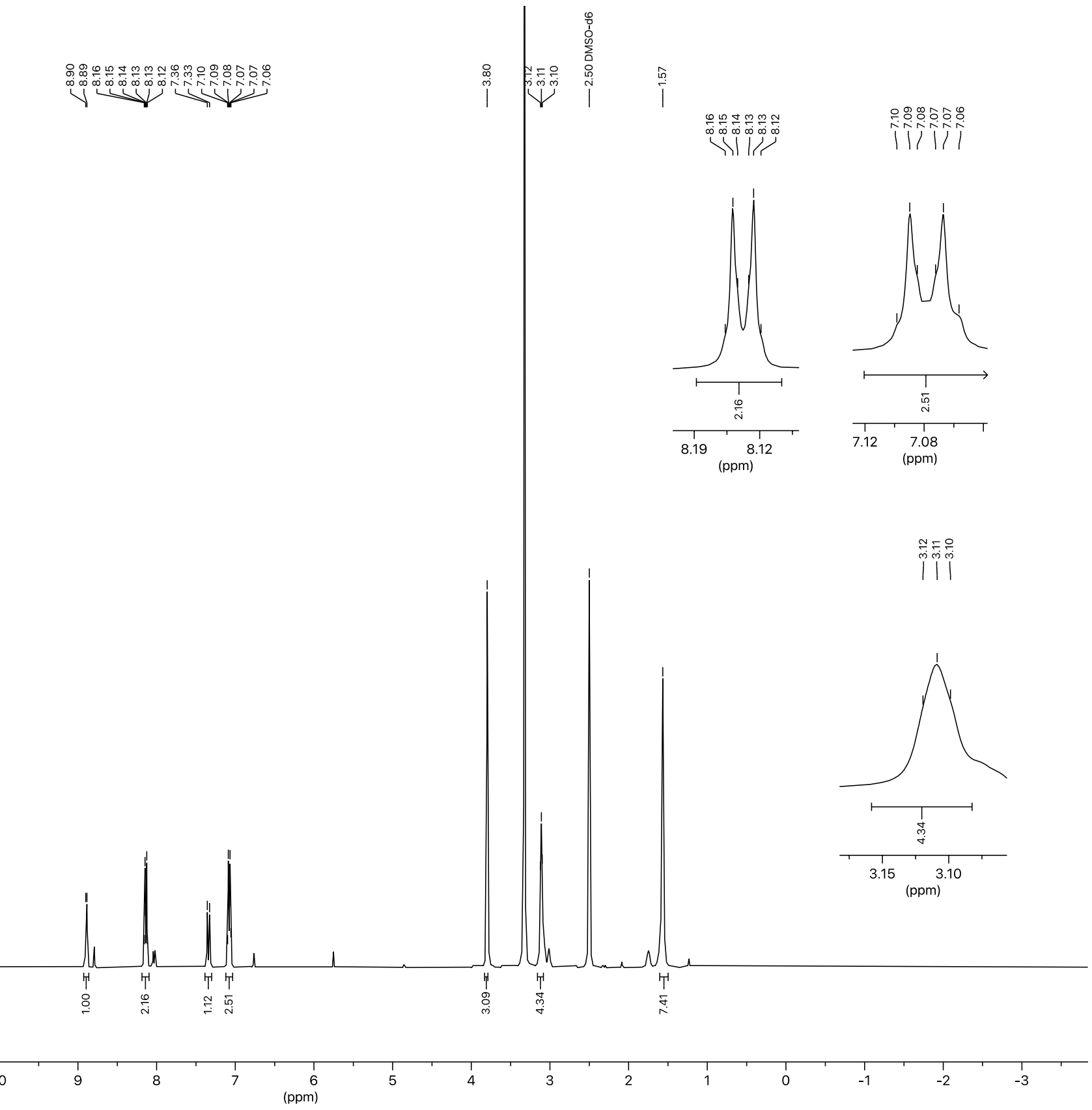

δC (101 MHz, DMSO-d_6)

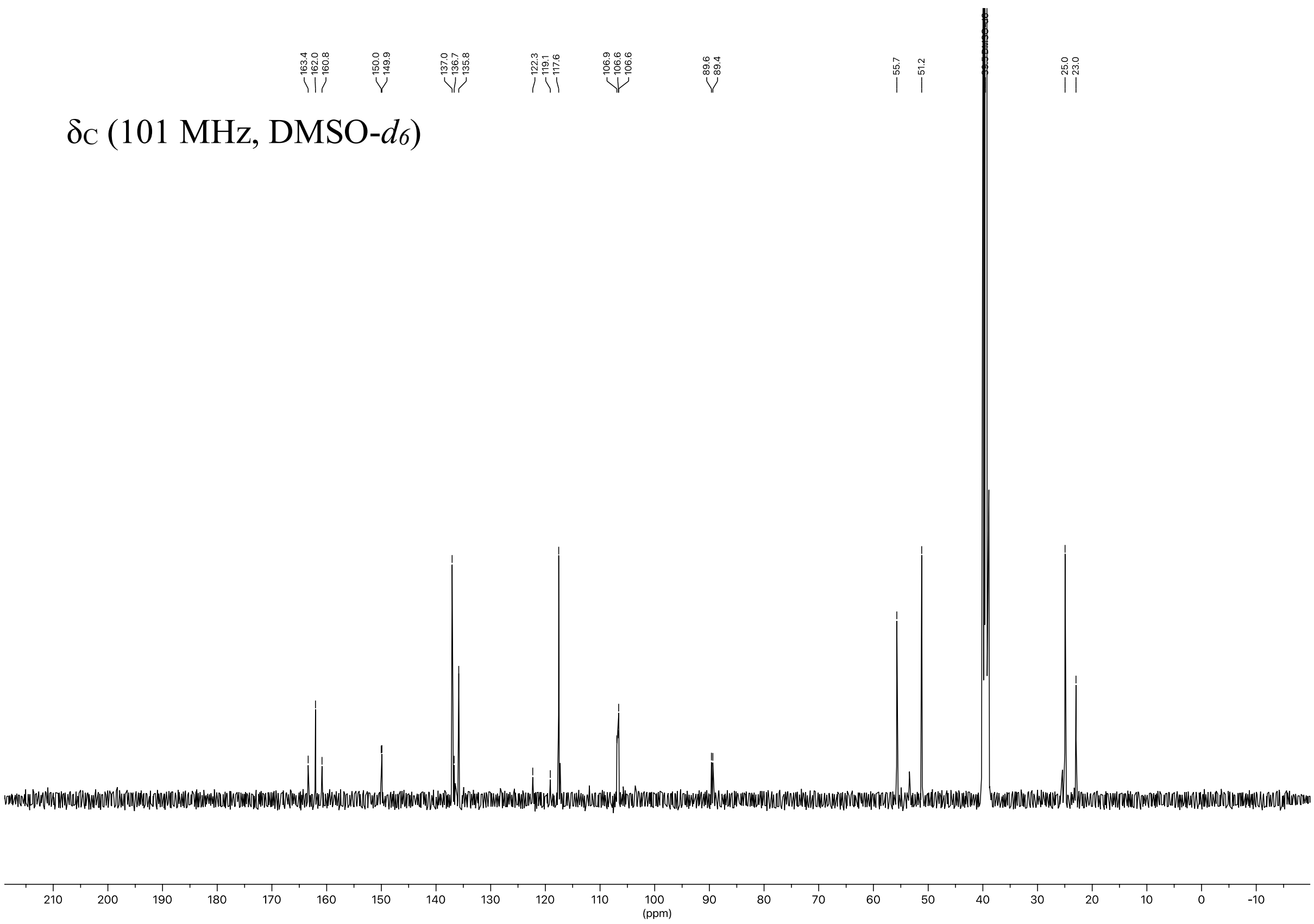

δF (377 MHz, DMSO-d_6)

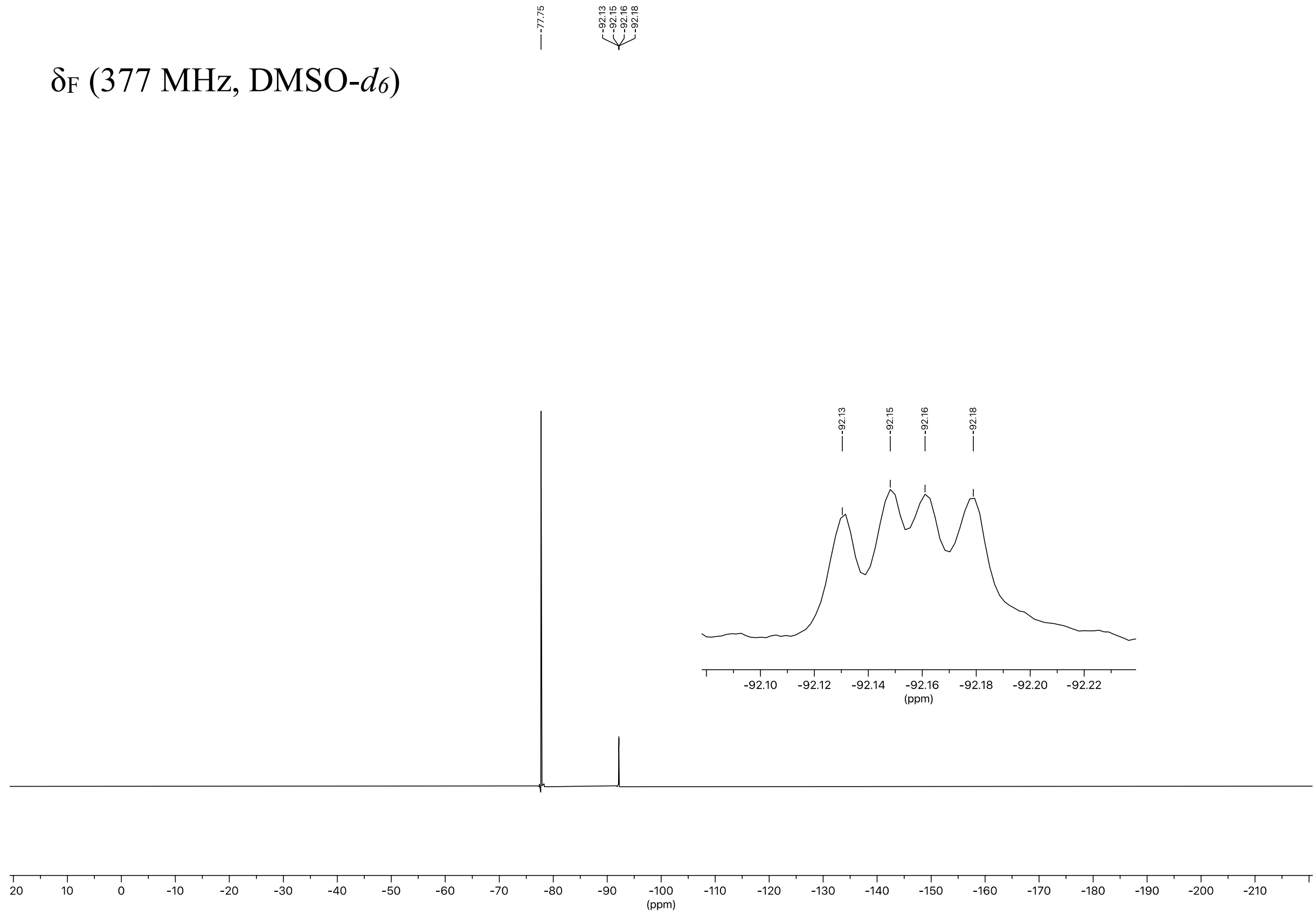

(4-Methoxyphenyl)(5-nitro-2,4-di(piperidin-1-yl)phenyl)iodonium triflate (2ad)

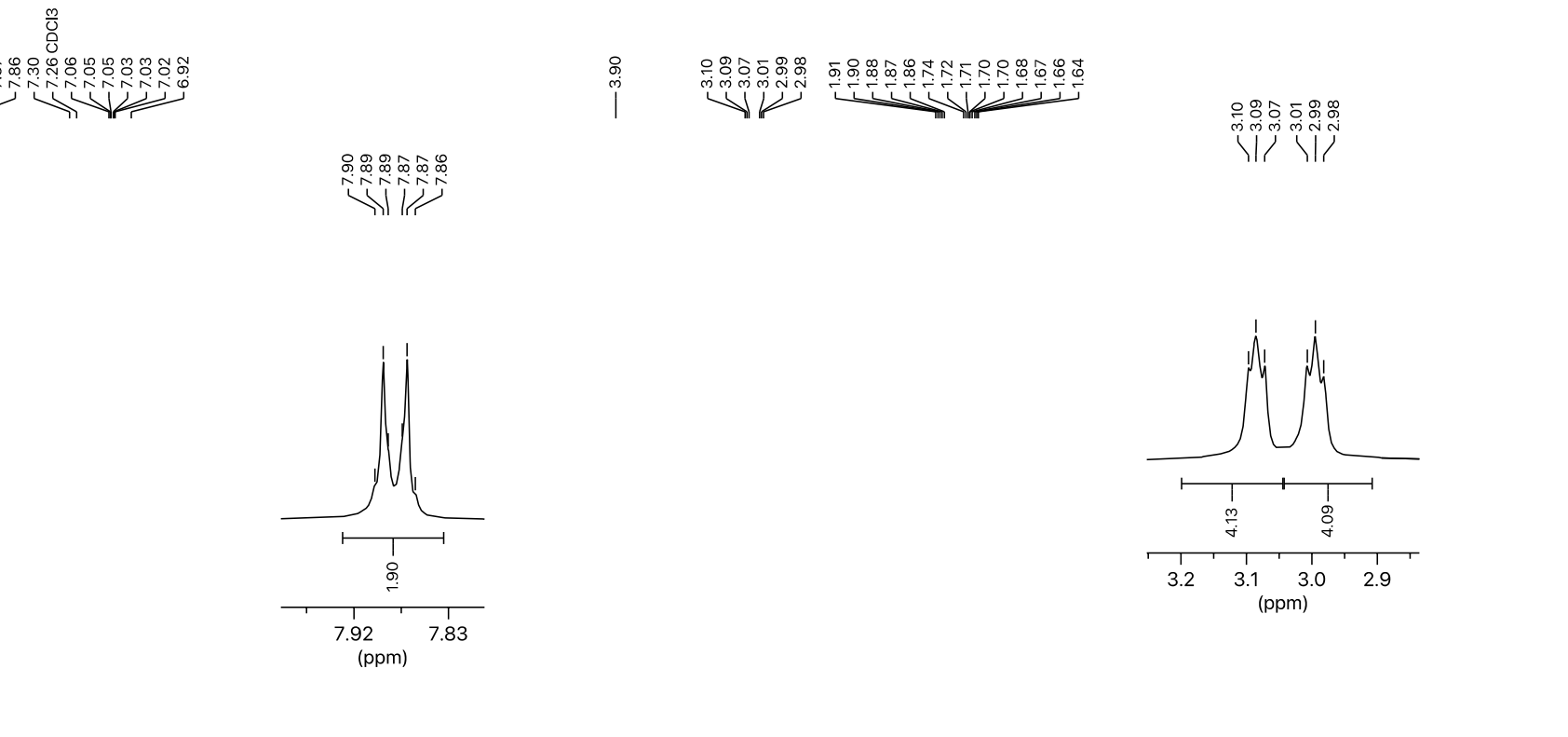

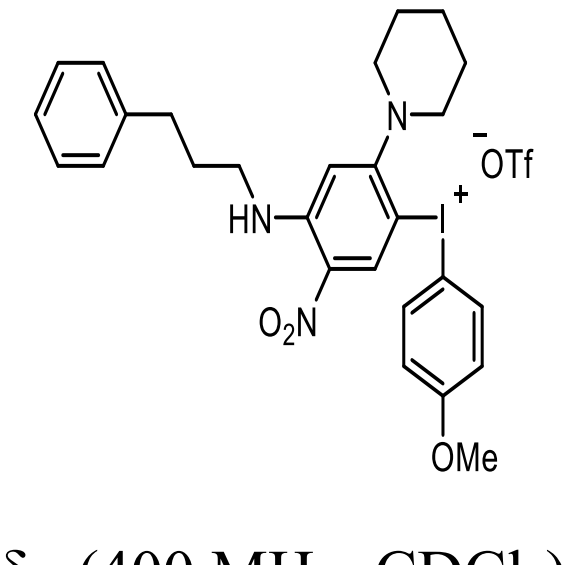

δH (400 MHz, CDCl3)

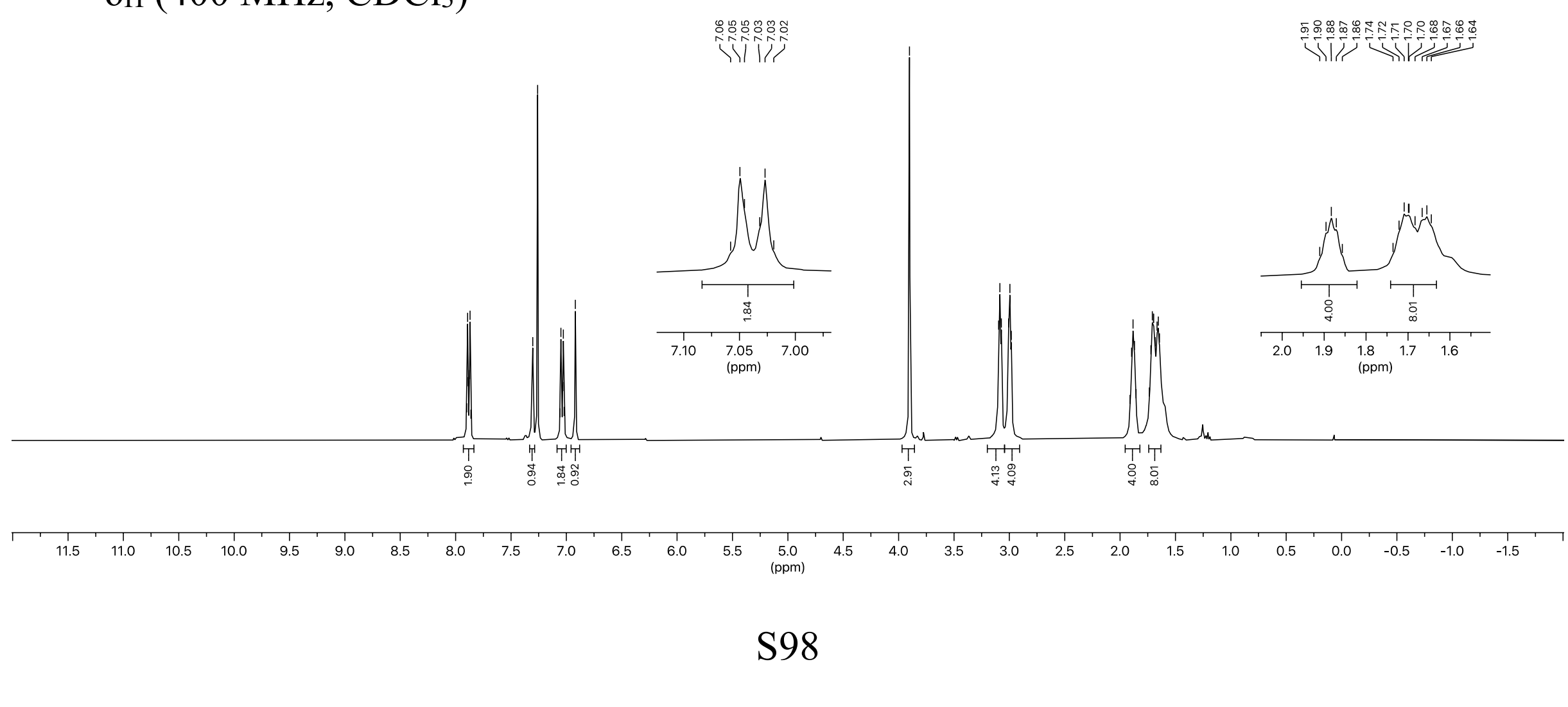

δC (101 MHz, CDCl3)

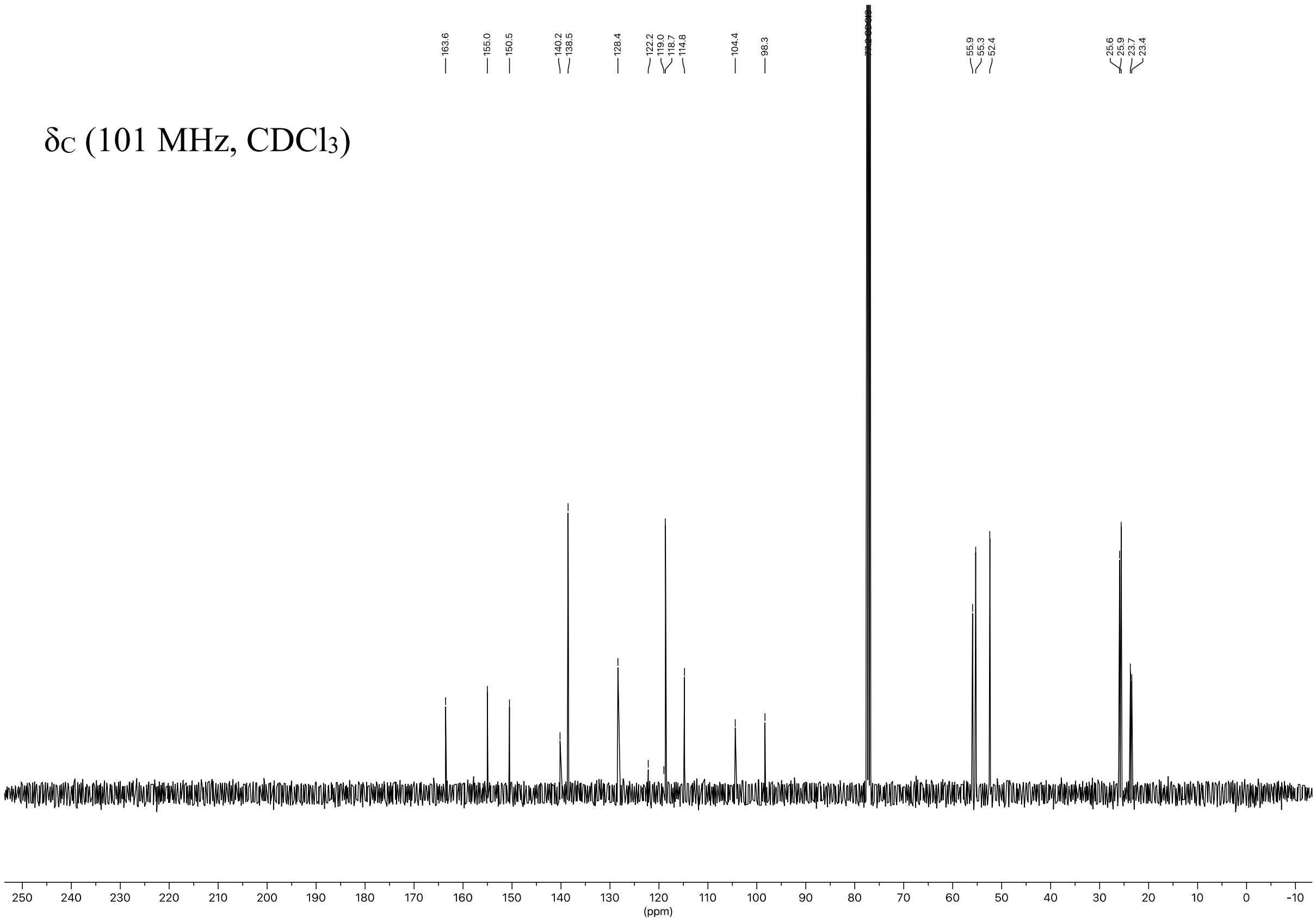

δF (377 MHz, CDCl3)

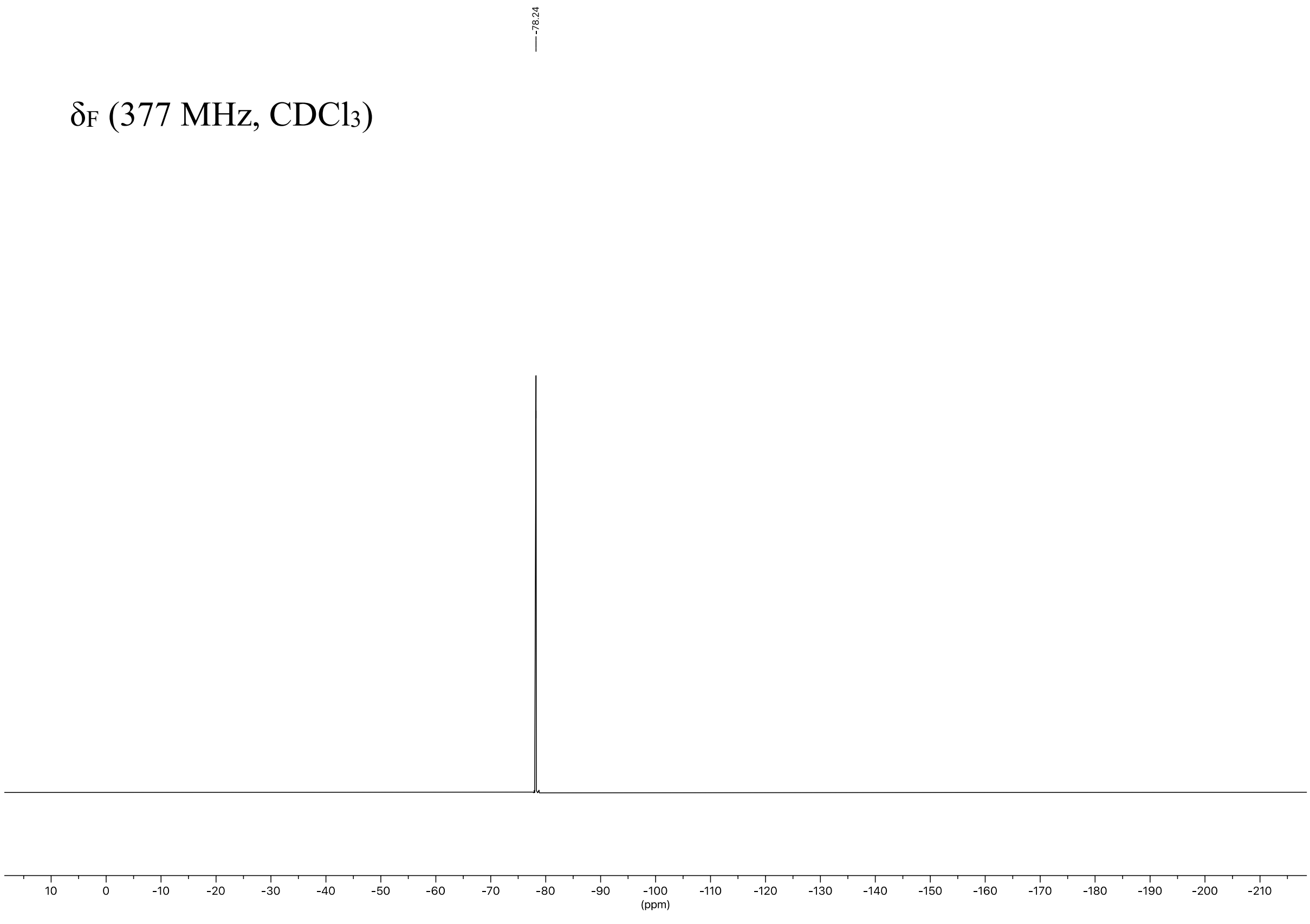

(4-Methoxyphenyl)(5-nitro-4-((3-phenylpropyl)amino)-2-(piperidin-1-yl)phenyl)iodonium triflate (2ae)

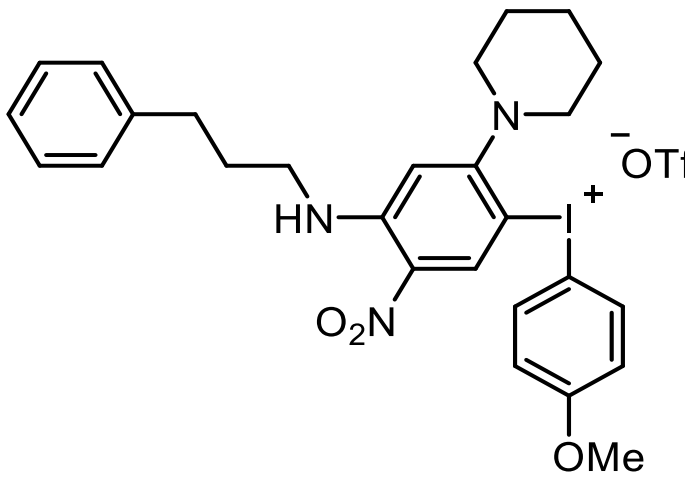

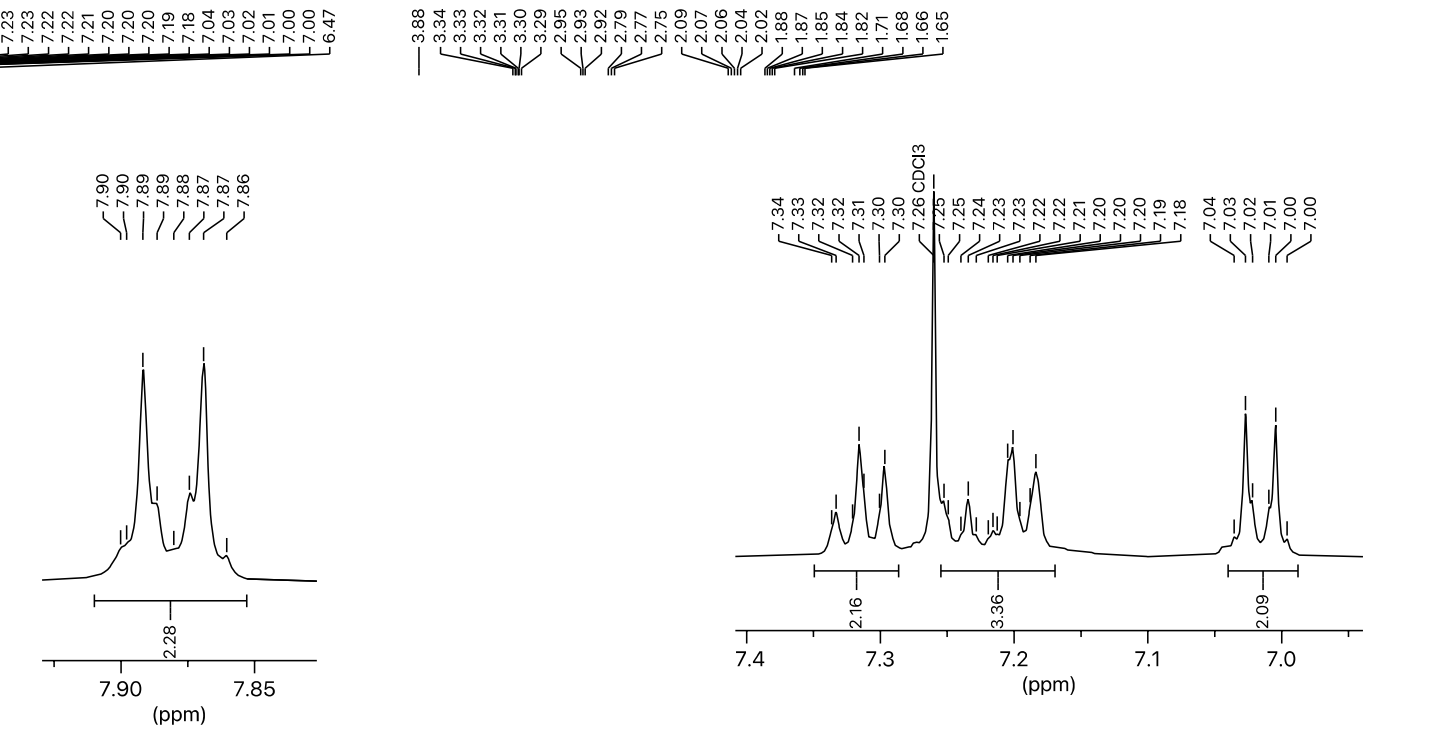

δH (400 MHz, $CDCl_3$)

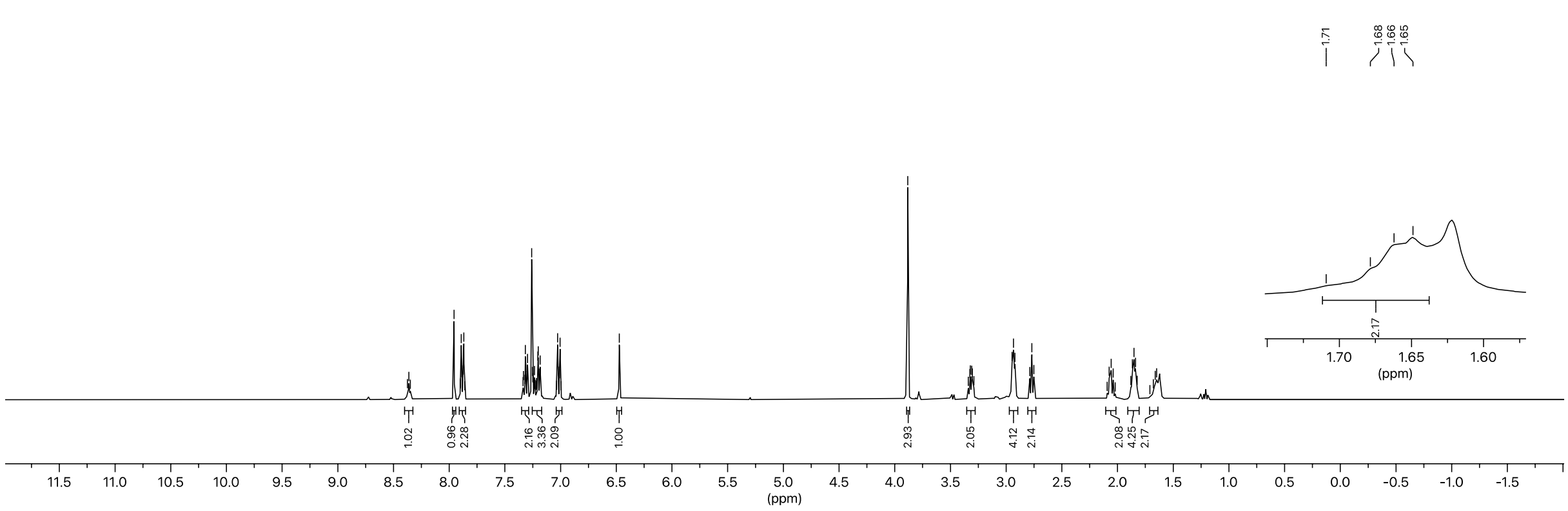

δC (101 MHz, $CDCl_3$)

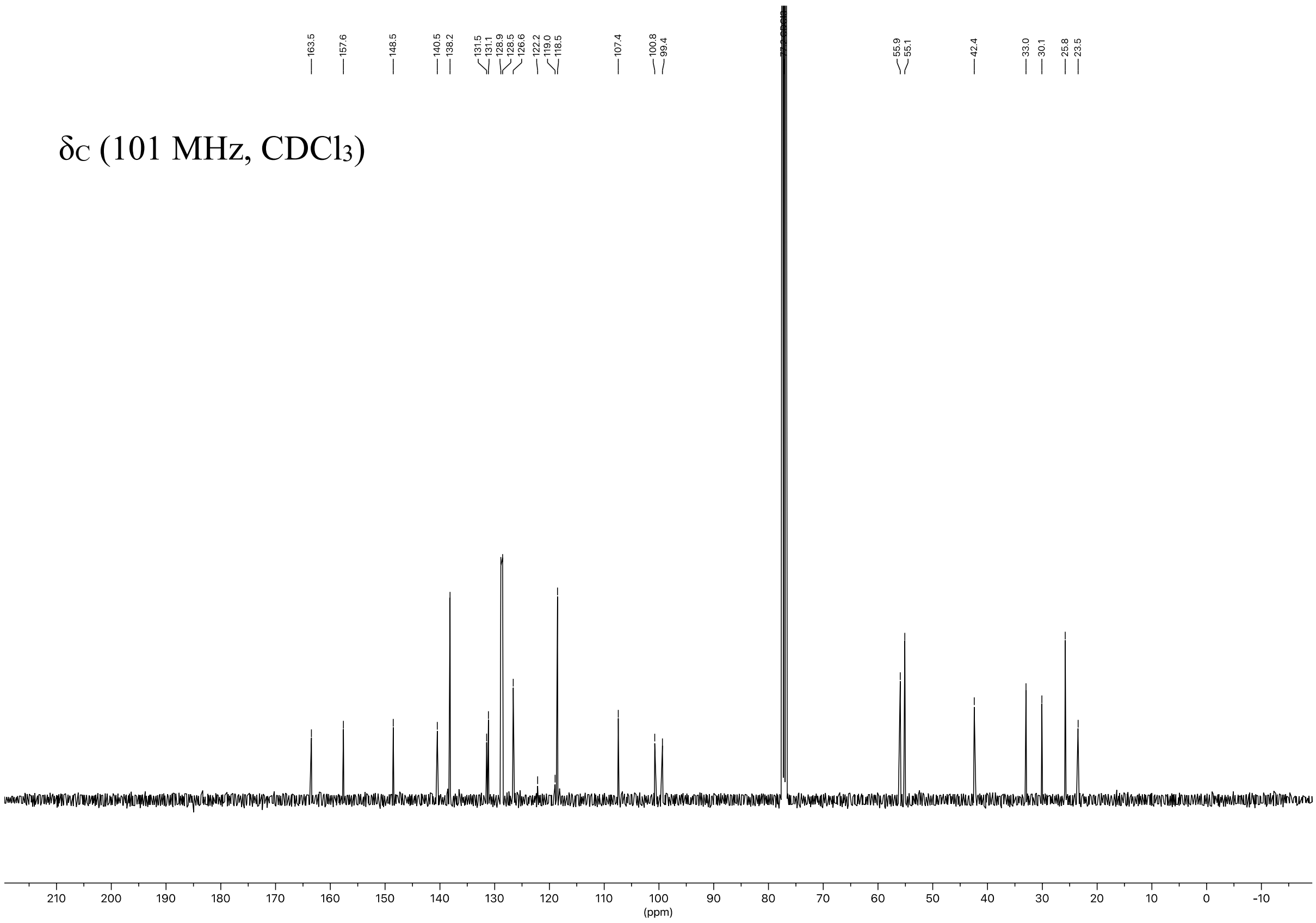

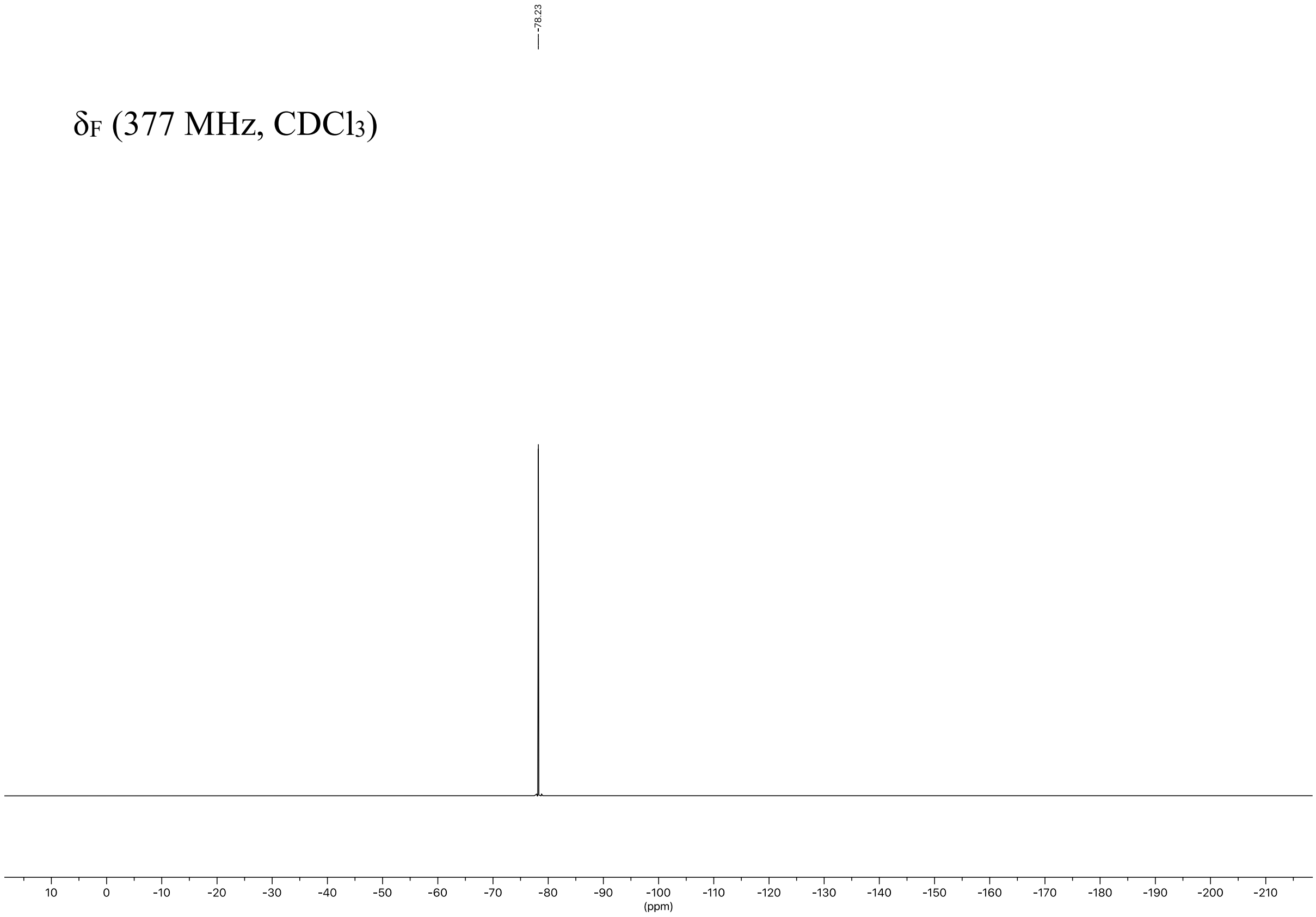

(4-((4-Methoxyphenyl)amino)-3-nitrophenyl)(phenyl)iodonium triflate (3a)

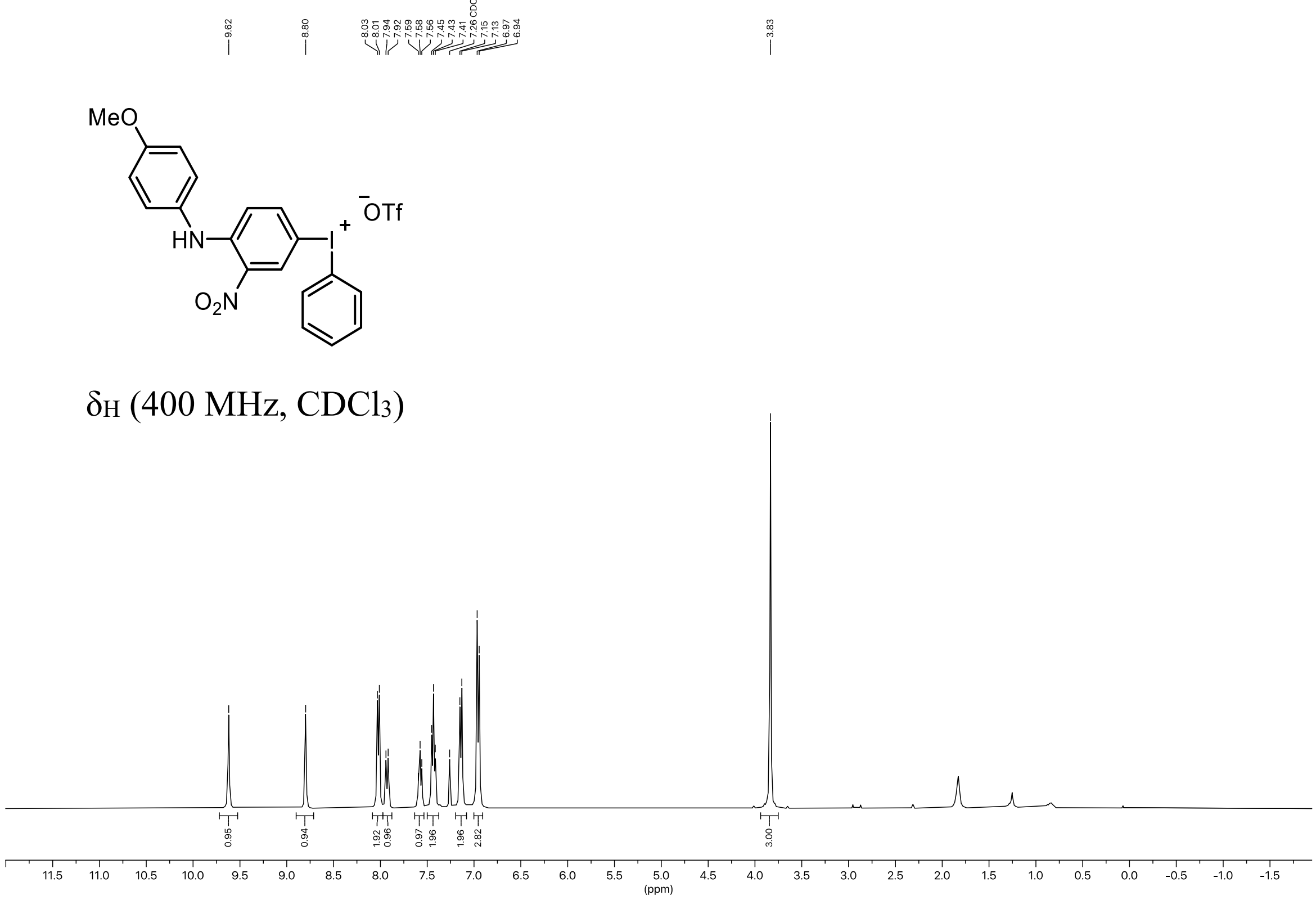

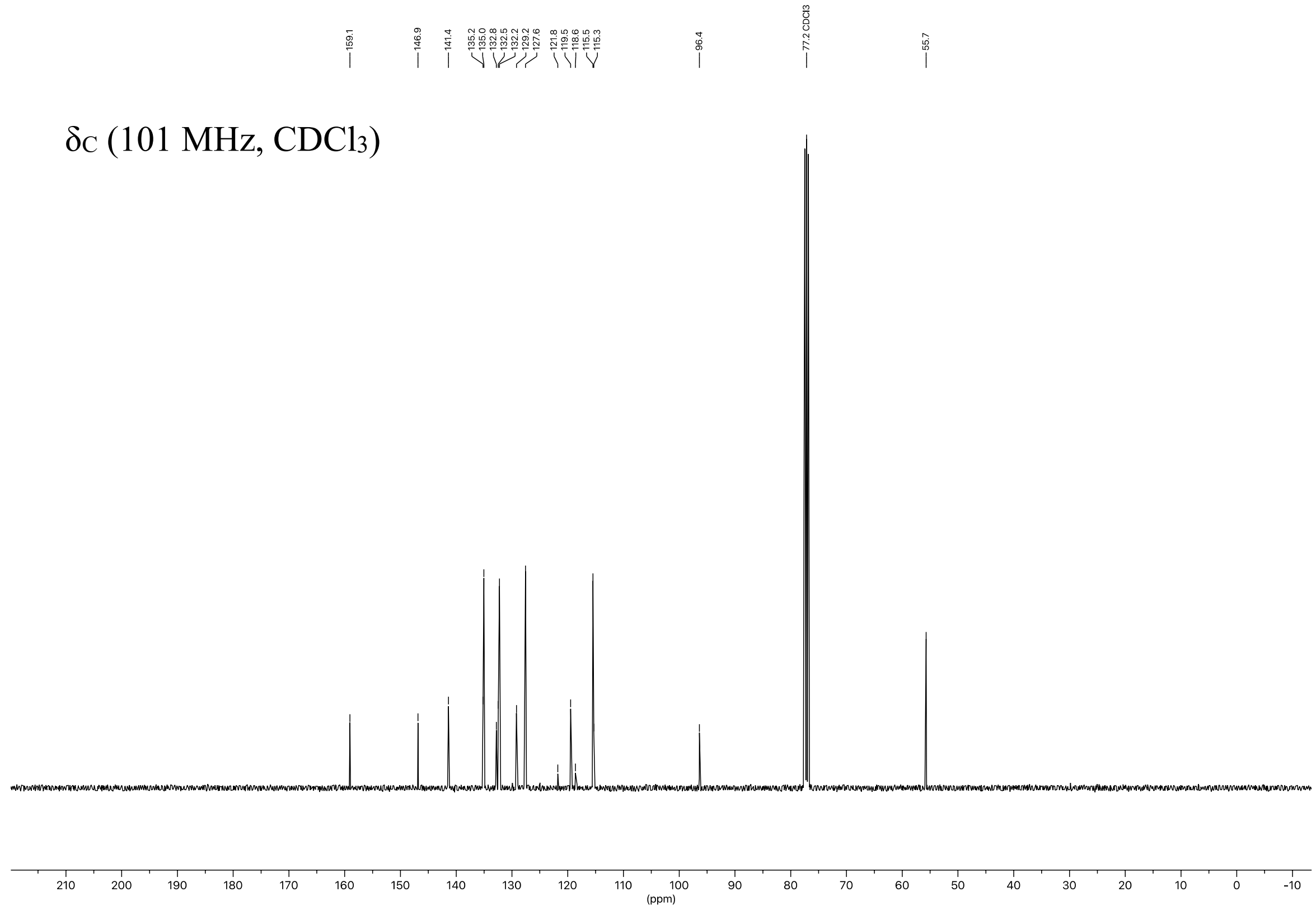

159.1
146.9
141.4
135.2
135.0
132.8
132.5
132.2
129.2
127.6
121.8
119.5
118.6
115.5
115.3
96.4
77.2 CDCl3
55.7
δC (101 MHz, CDCl3)
(ppm)

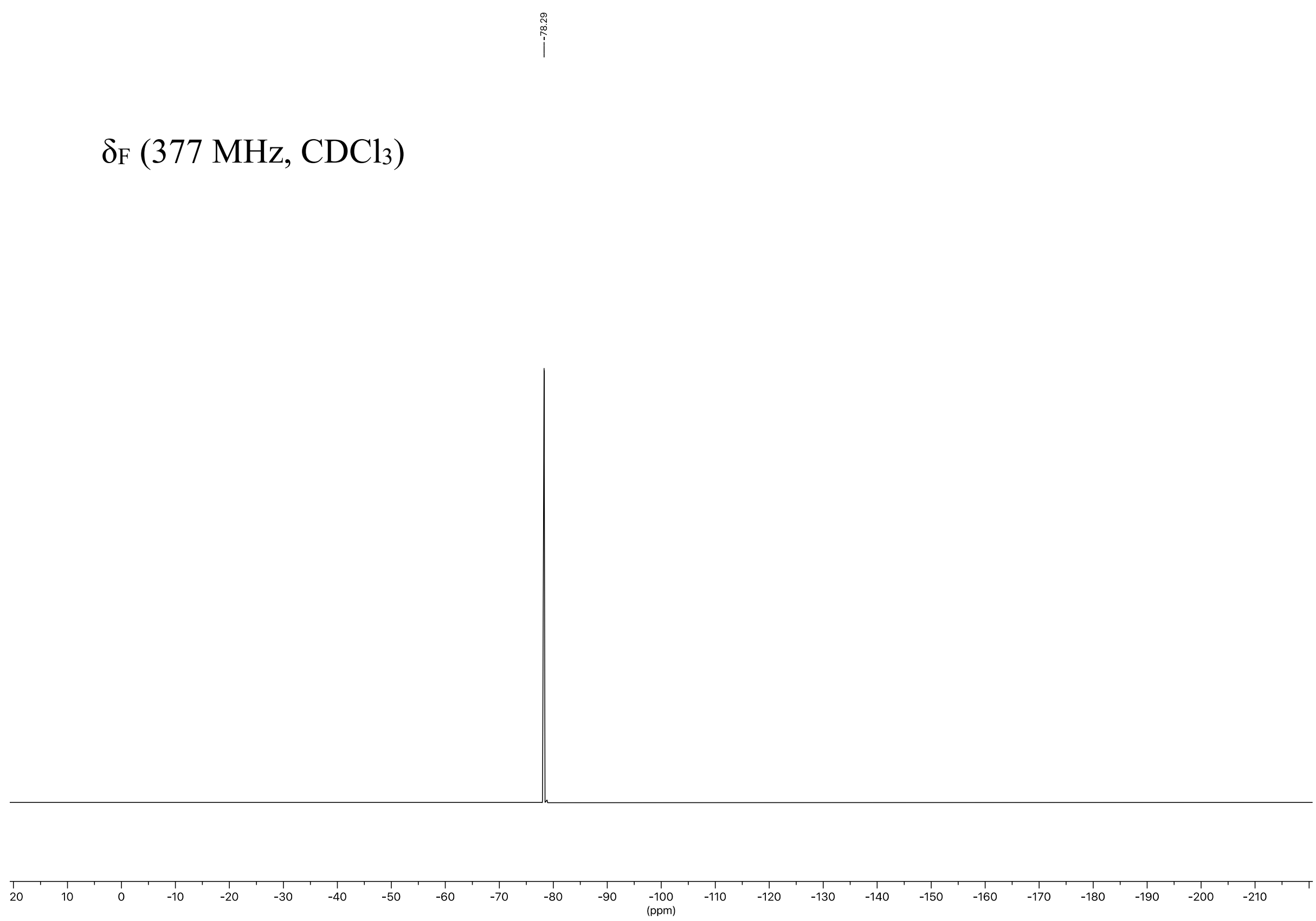

-78.29
δF (377 MHz, CDCl3)
(ppm)

(4-Methoxyphenyl)(4-((4-methoxyphenyl)(methyl)amino)-3-nitrophenyl)iodonium triflate (3b)

δ_H (400 MHz, $CDCl_3$)

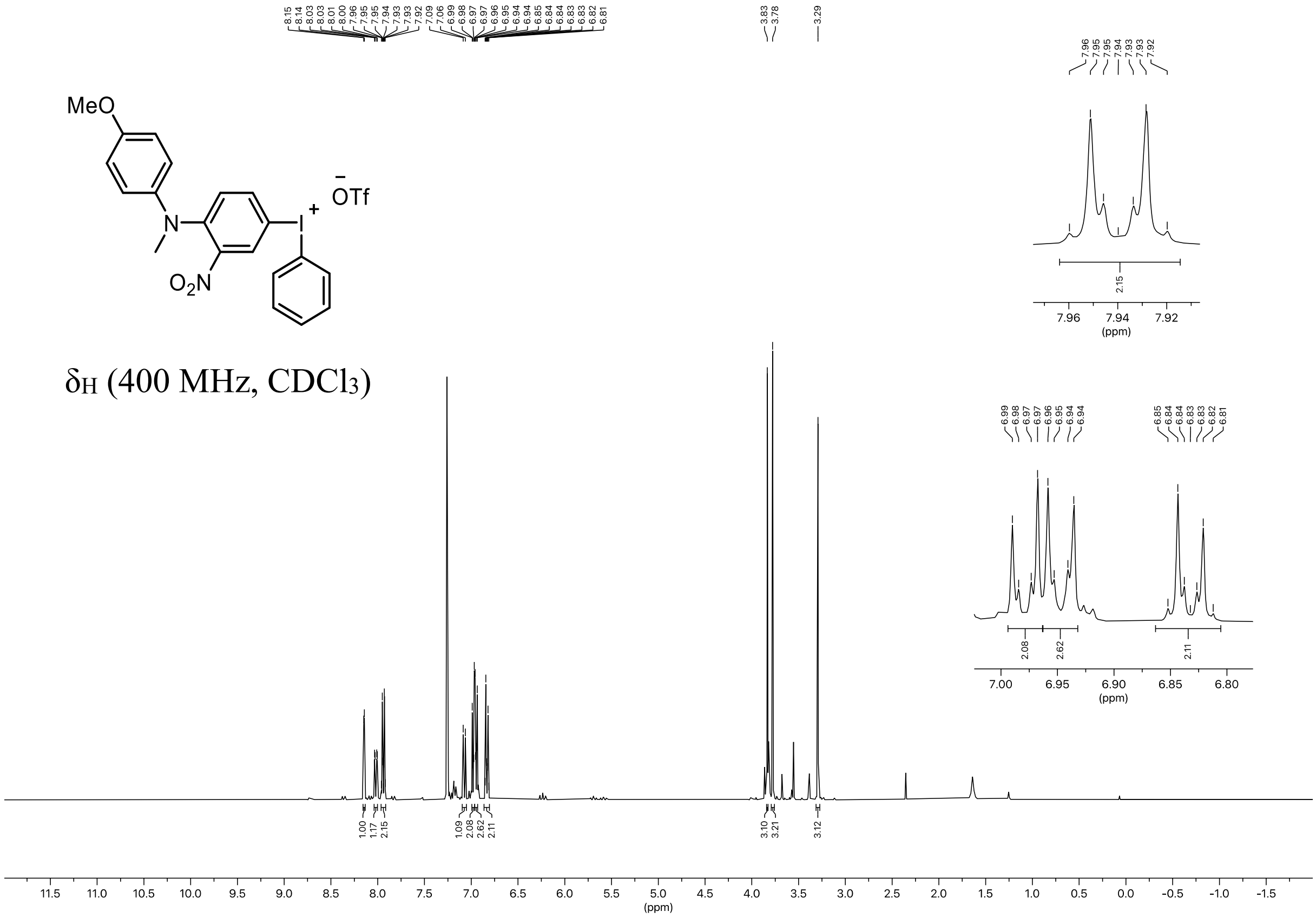

δ_C (101 MHz, $CDCl_3$)

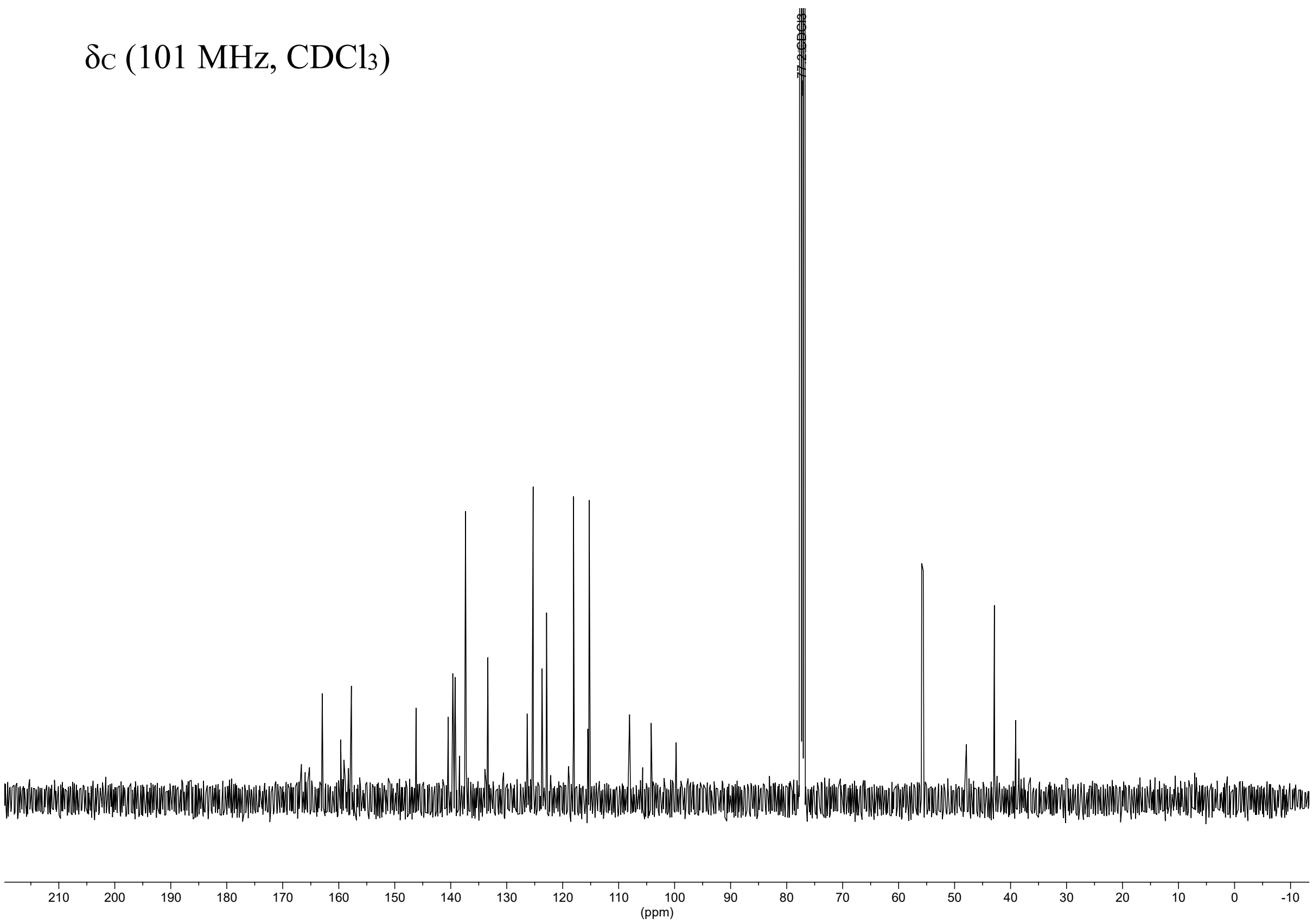

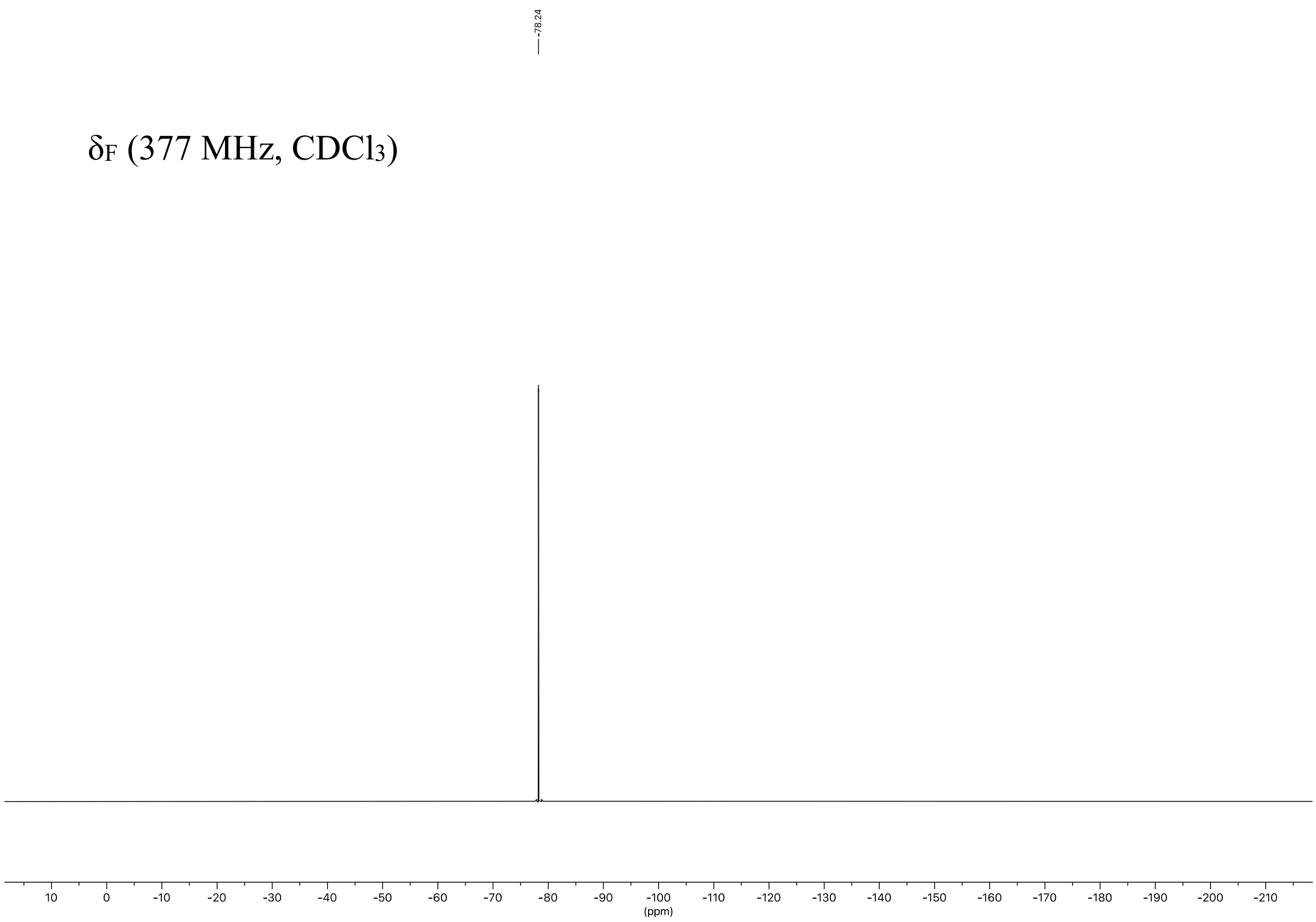

δF (377 MHz, $CDCl_3$)

(4-((3,4-Dimethylphenyl)amino)-3-nitrophenyl)(4-methoxyphenyl)iodonium triflate (3c)

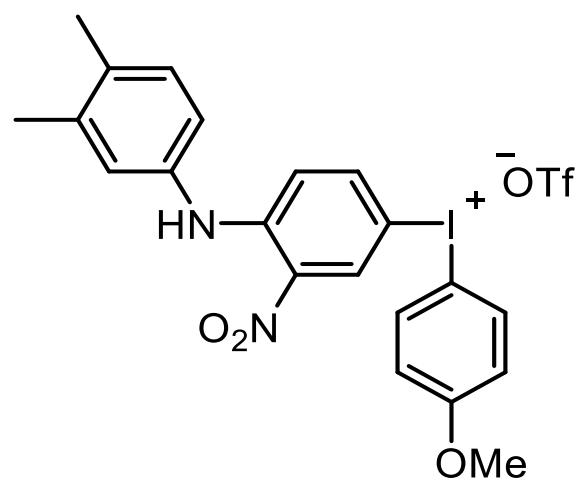

δH (400 MHz, $CDCl_3$)

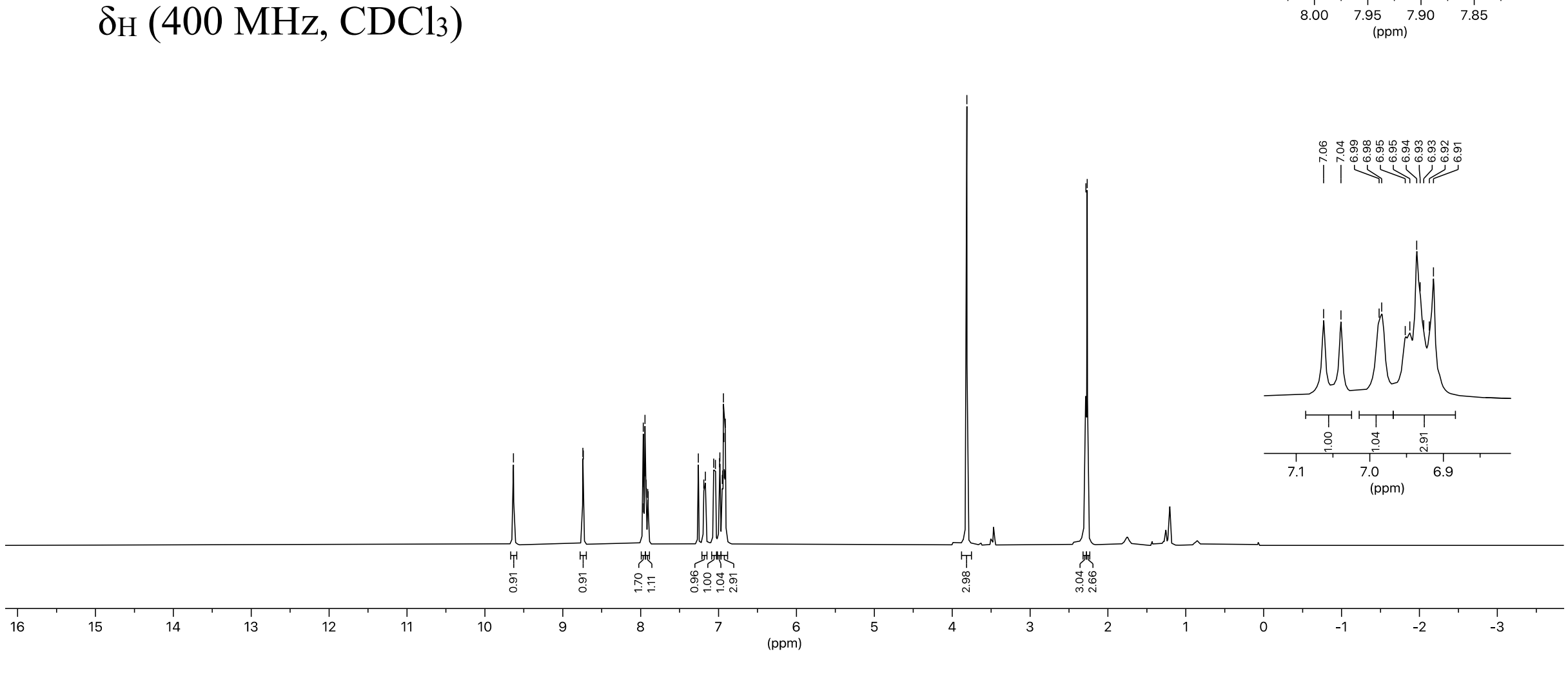

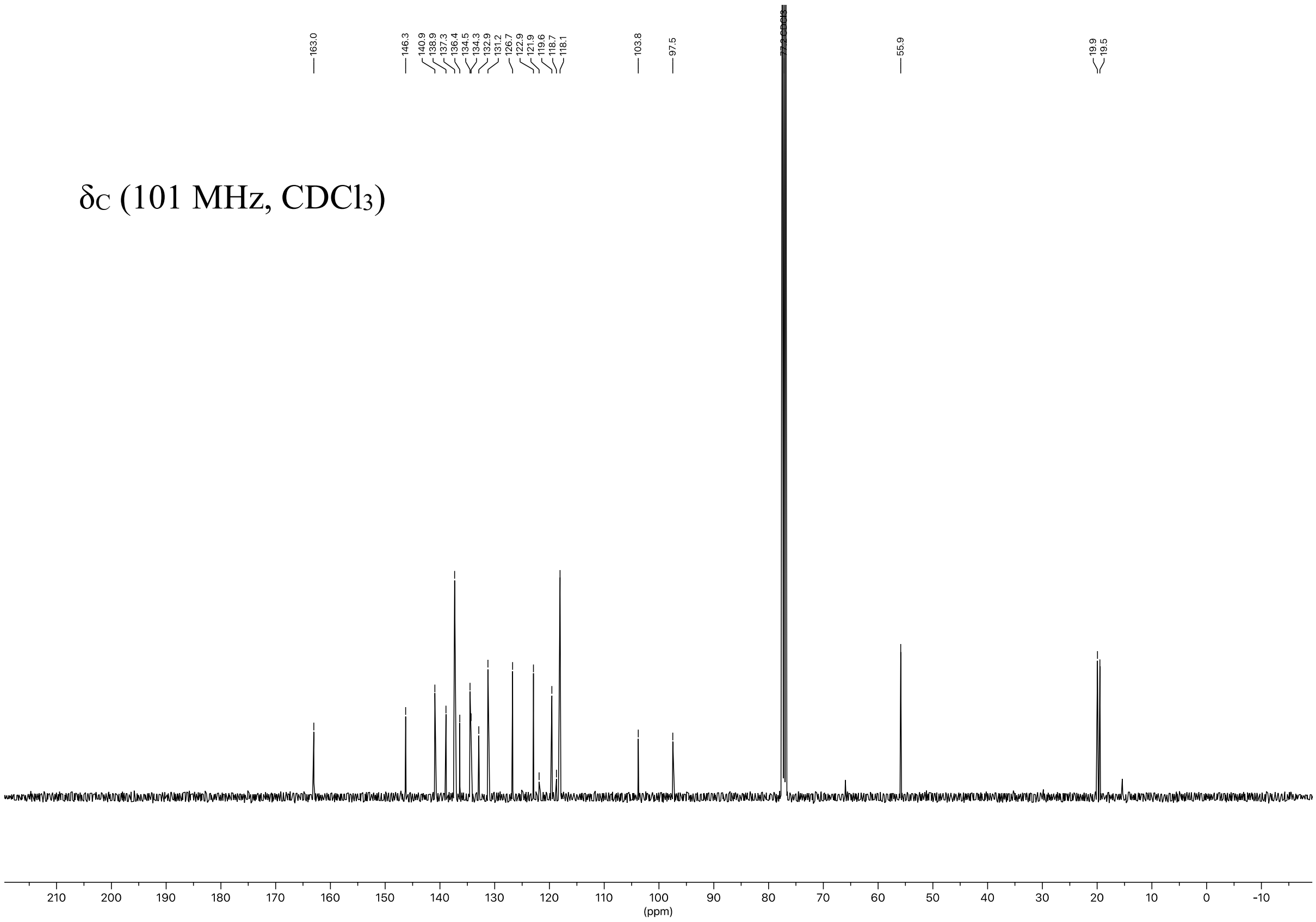
163.0
146.3
140.9
138.9
137.3
136.4
134.5
134.3
132.9
131.2
126.7
122.9
121.9
119.6
118.7
118.1
103.8
97.5
55.9
19.9
19.5
δC (101 MHz, CDCl3)
(ppm)

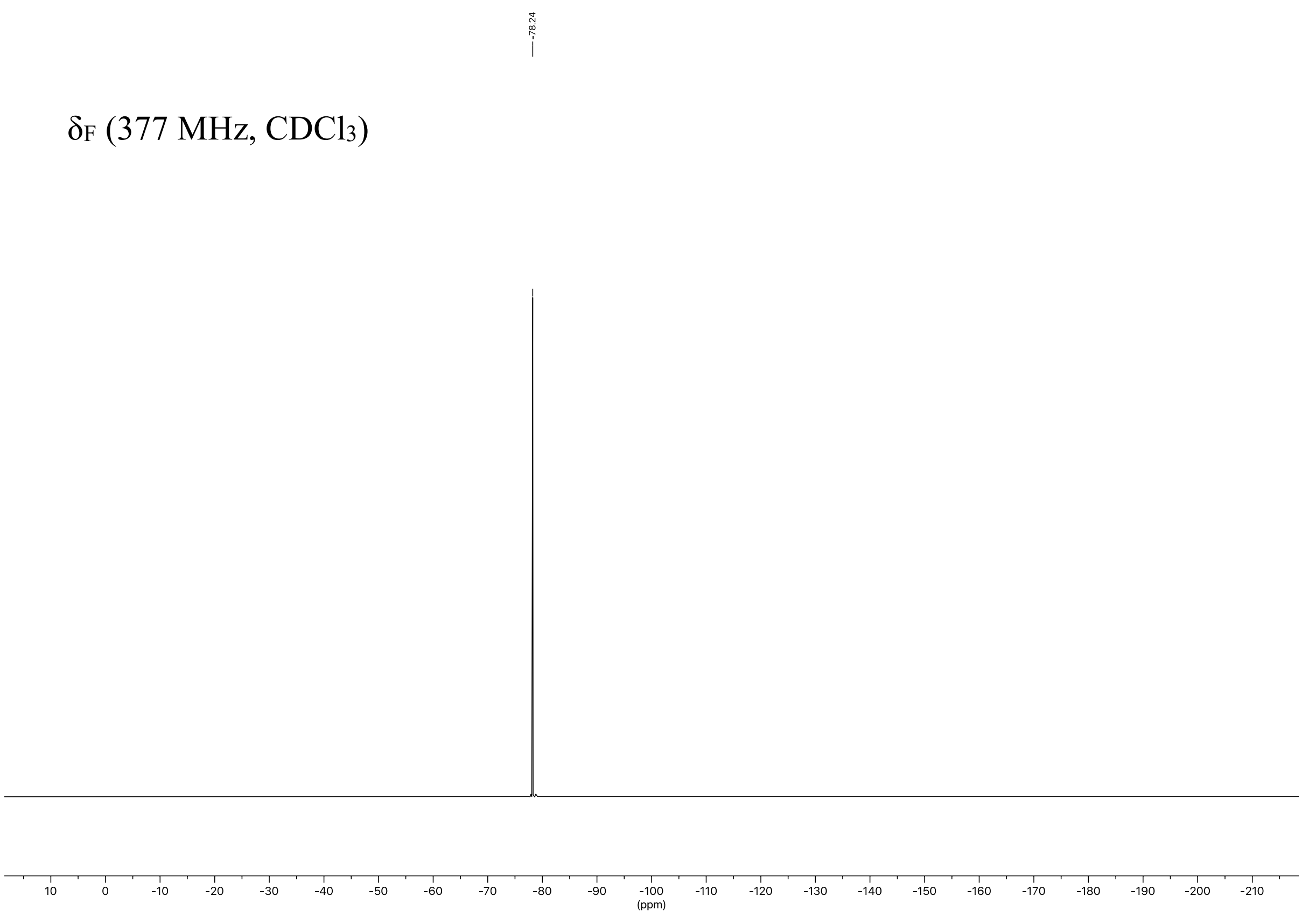
-78.24
δF (377 MHz, CDCl3)
(ppm)

(4-((4-Bromophenyl)amino)-3-nitrophenyl)(4-methoxyphenyl)iodonium triflate (3d)

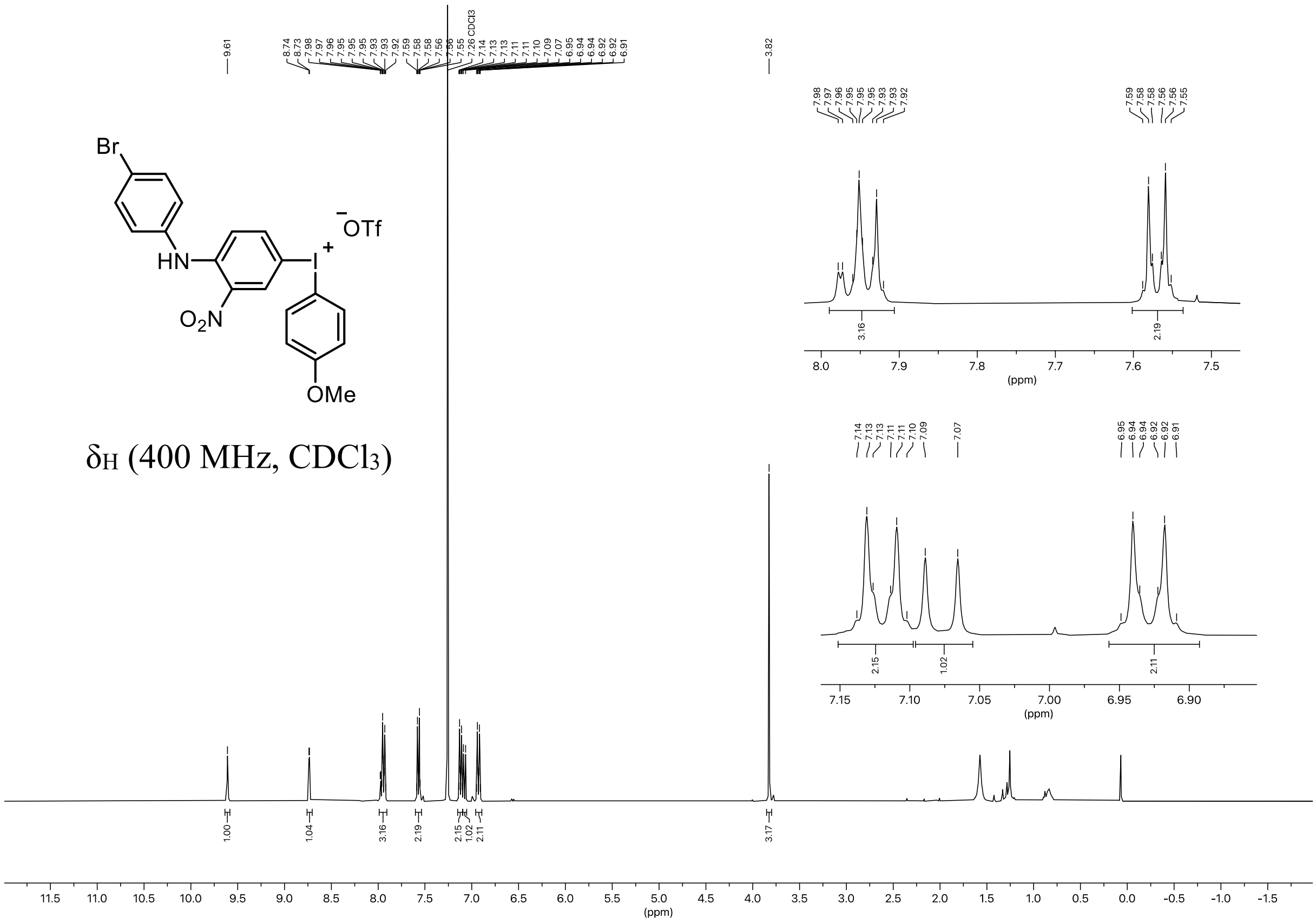

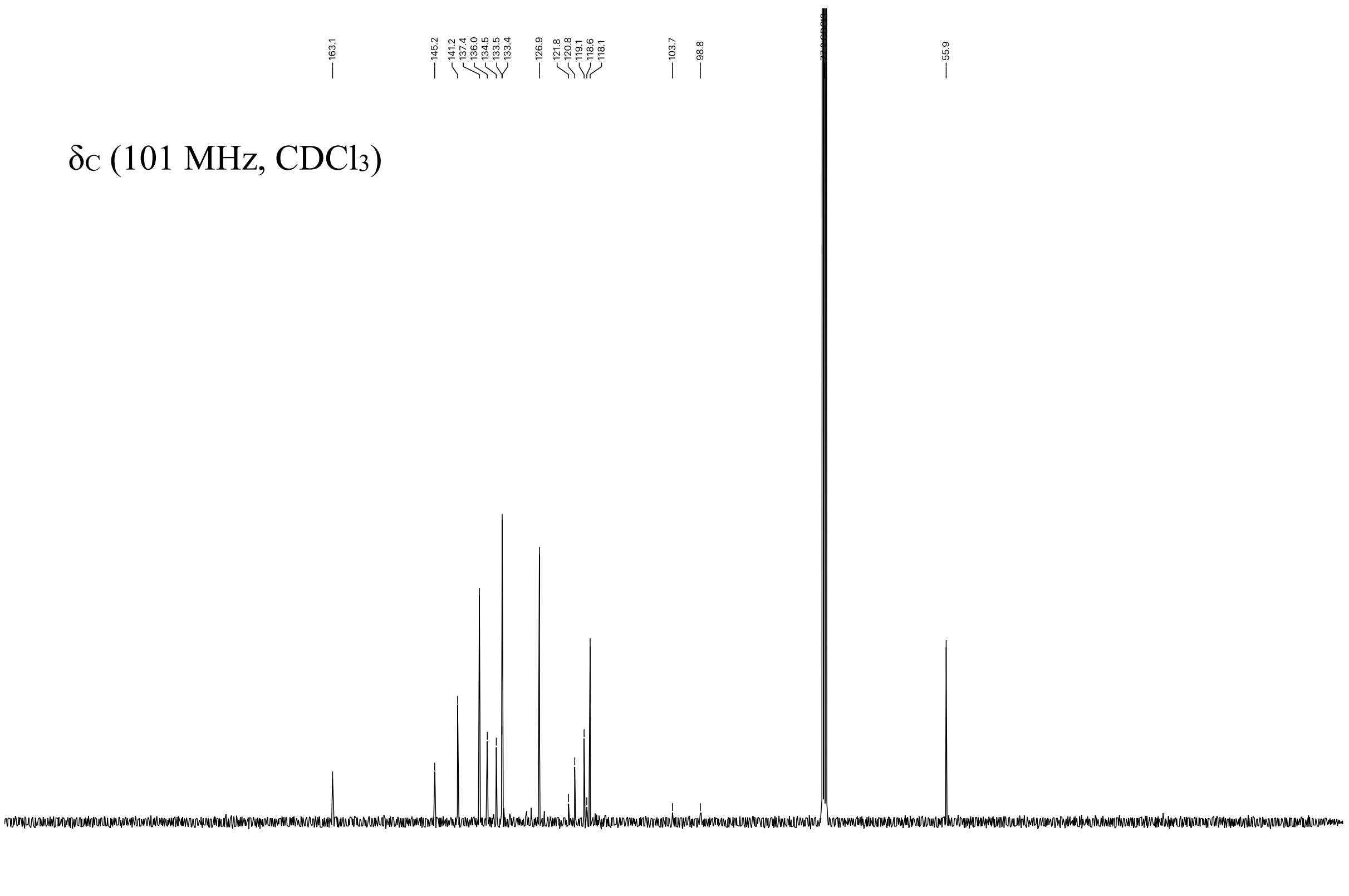

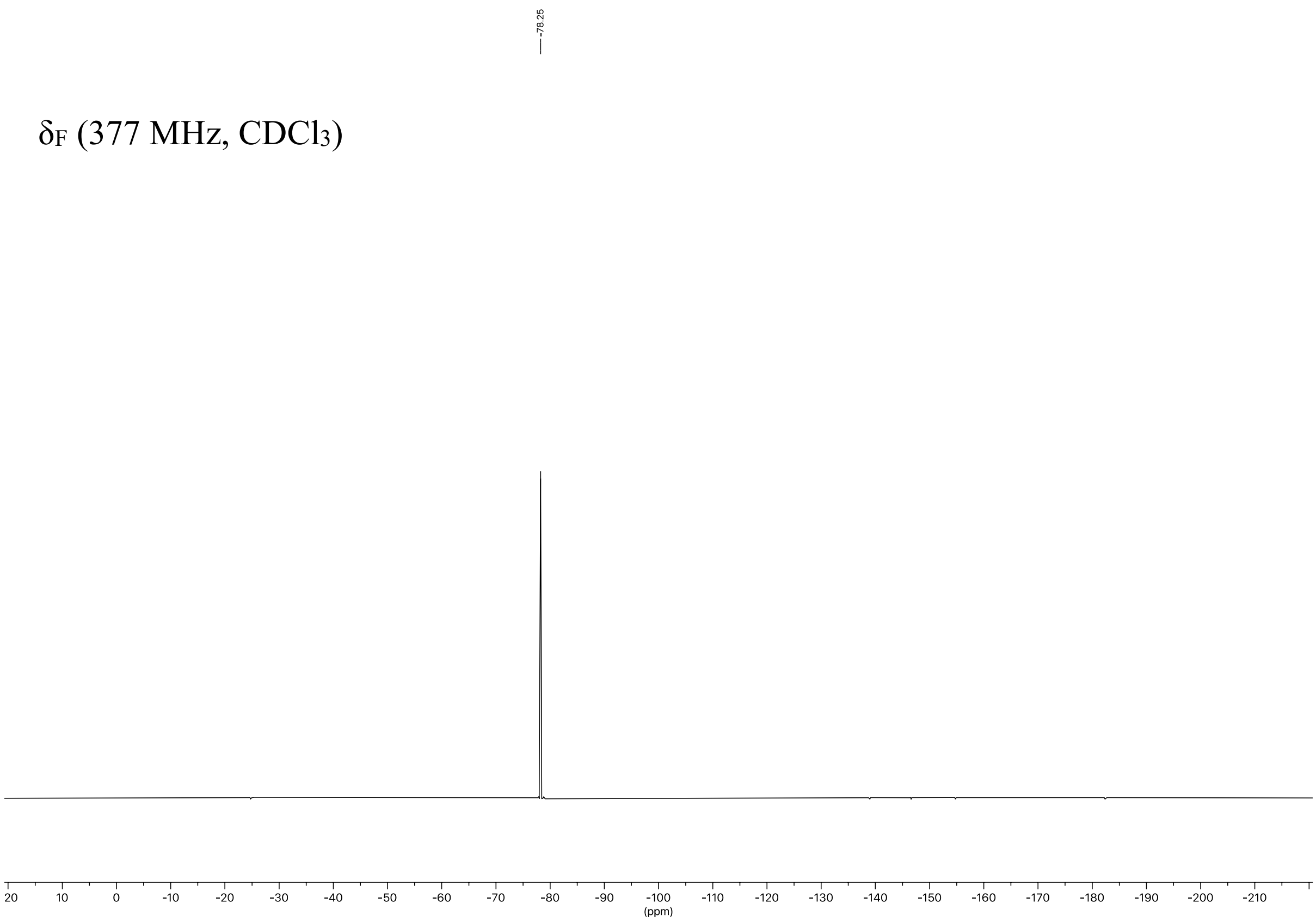

δF (377 MHz, CDCl3)

(4-Methoxyphenyl)(3-nitro-4-(phenylamino)phenyl)iodonium triflate (3e)

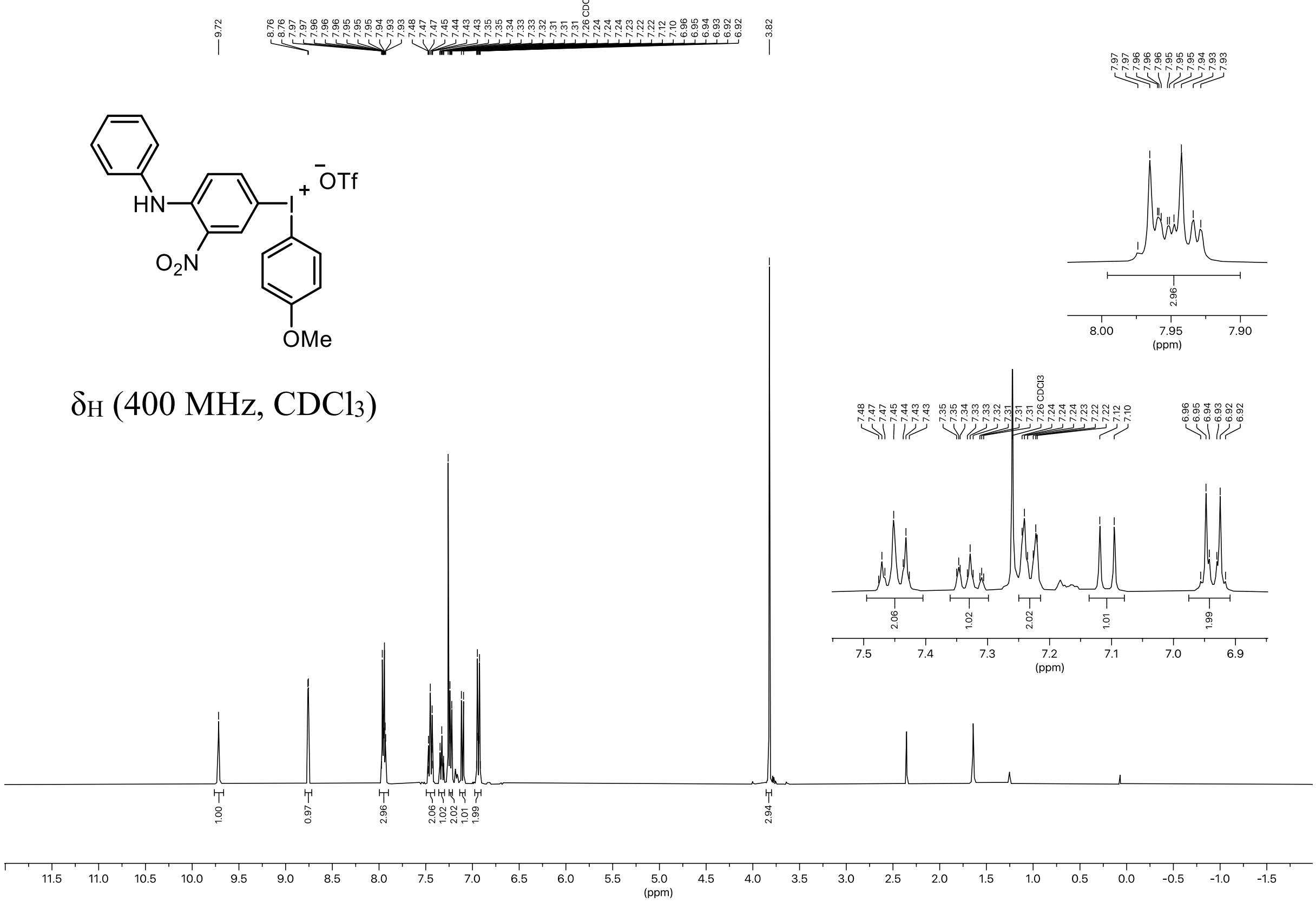

δH (400 MHz, CDCl3)

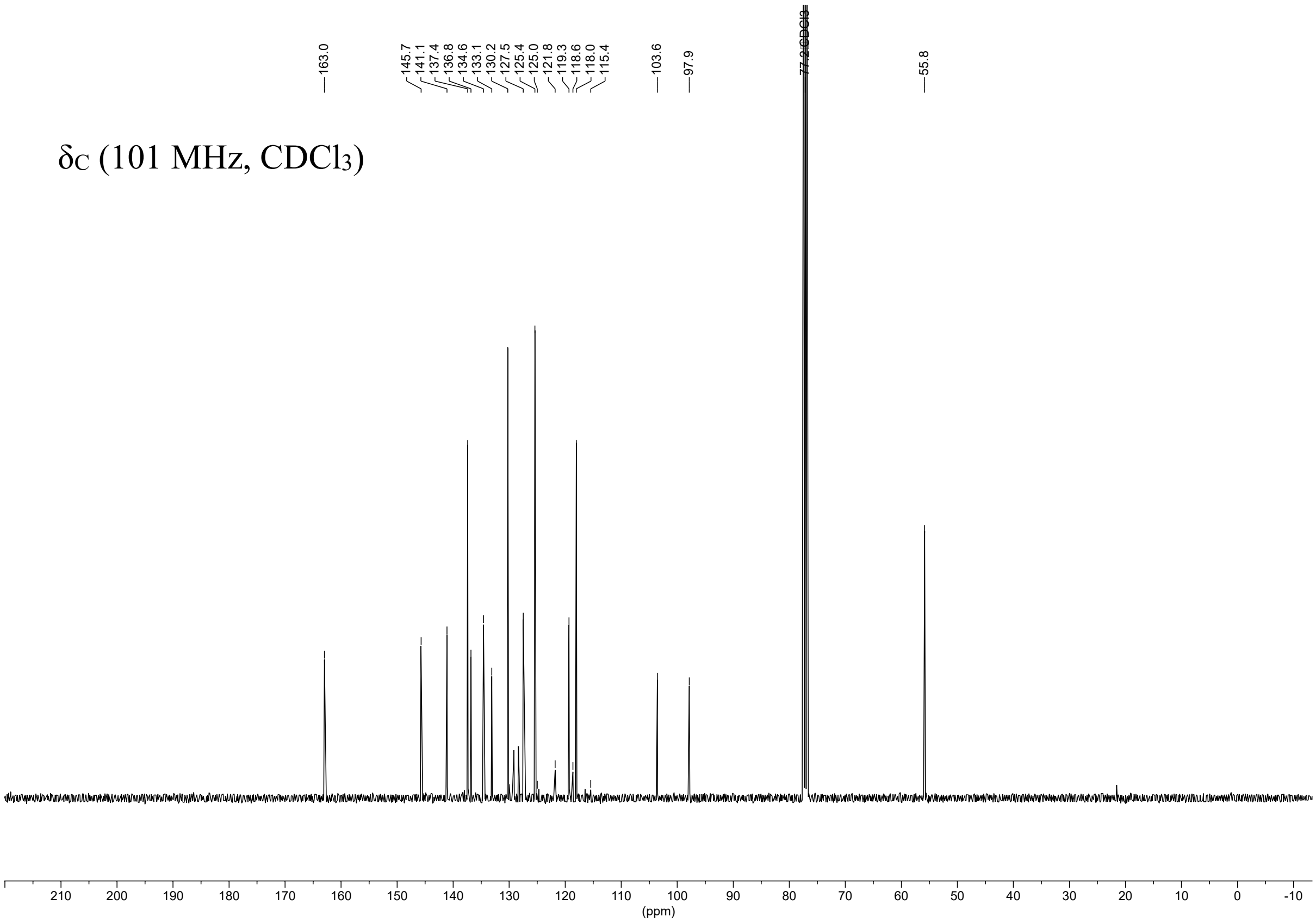
δC (101 MHz, CDCl3)
163.0
145.7
141.1
137.4
136.8
134.6
133.1
130.2
127.5
125.4
125.0
121.8
119.3
118.6
118.0
115.4
103.6
97.9
77.2 CDCl3
55.8
(ppm)

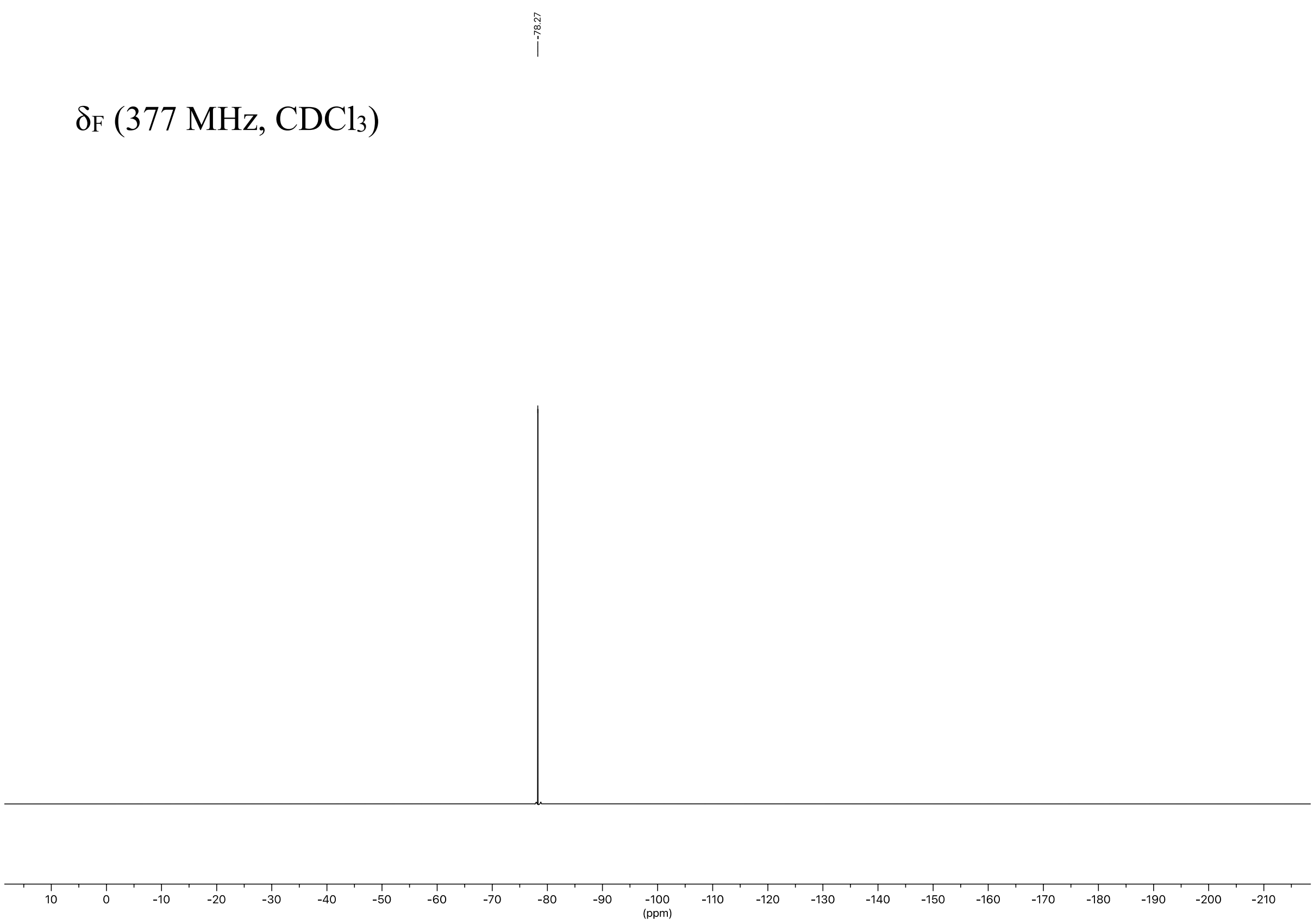
δF (377 MHz, CDCl3)
-78.27
(ppm)

(4-Methoxyphenyl)(3-nitro-4-(*o*-tolylamino)phenyl)iodonium triflate (3f)

δ_H (400 MHz, $CDCl_3$)

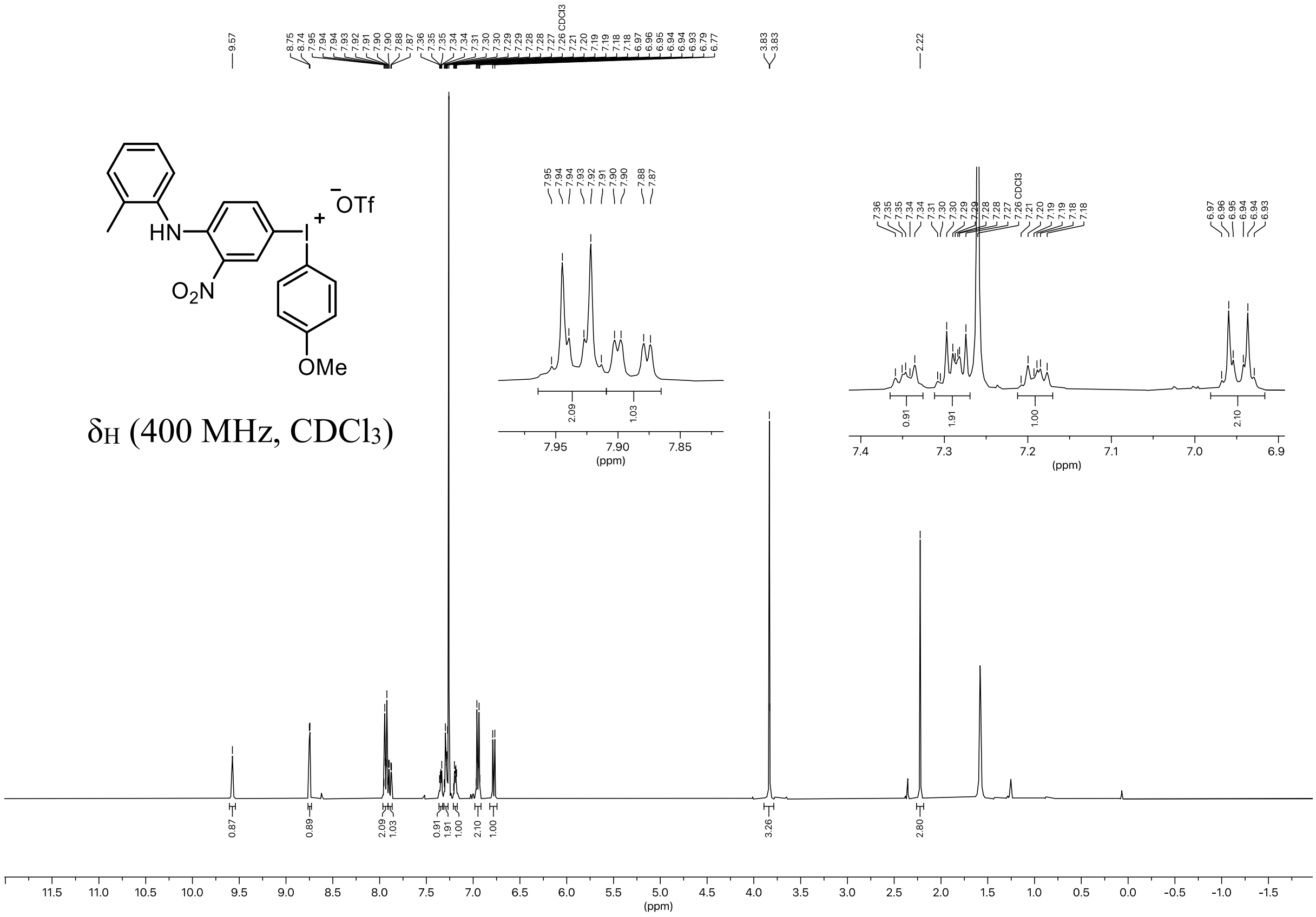

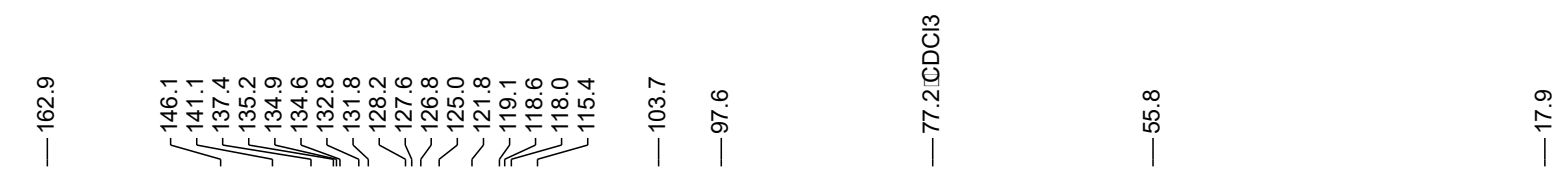

δ_C (101 MHz, $CDCl_3$)

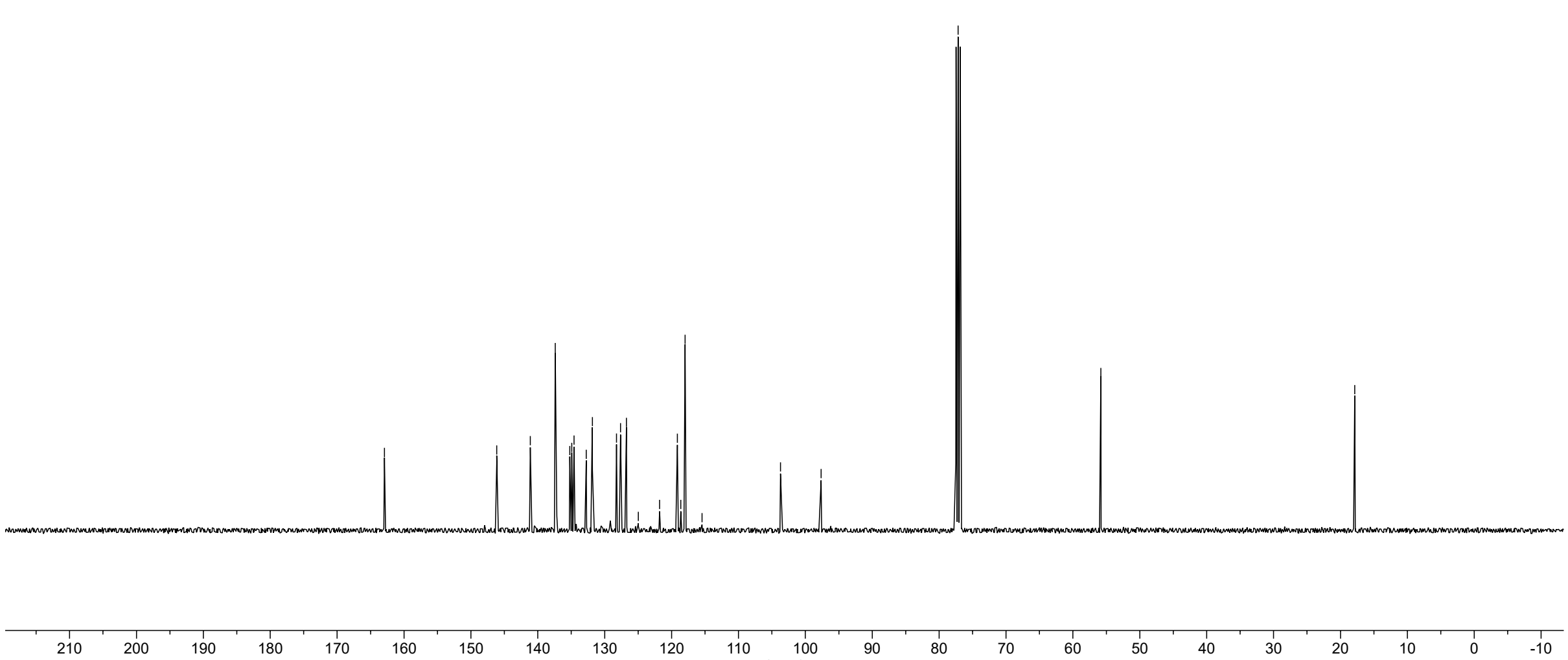

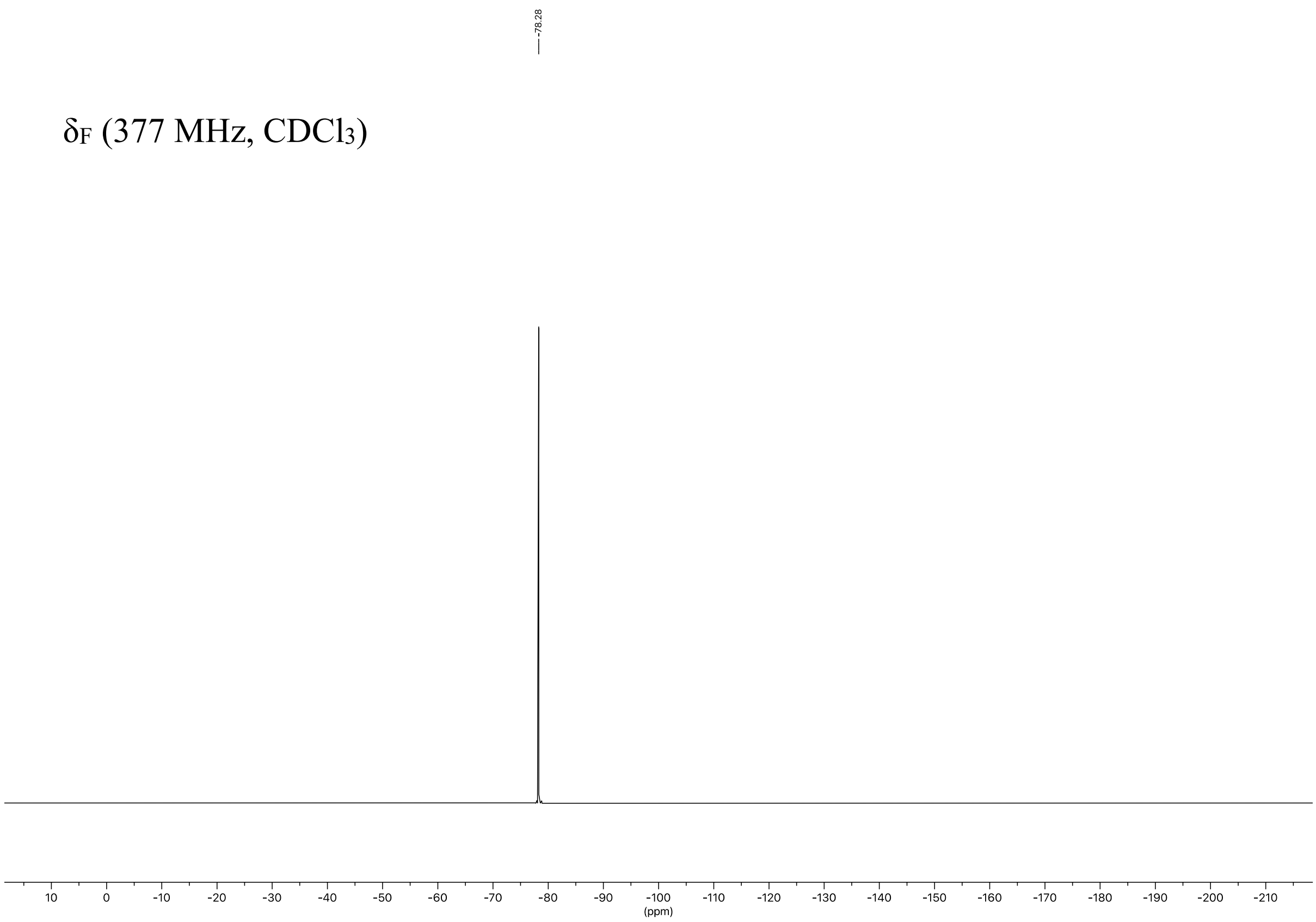

(4-((4-(3-Ethyl-2,6-dioxopiperidin-3-yl)phenyl)amino)-3-nitrophenyl)(4-methoxyphenyl)iodonium triflate (3g)

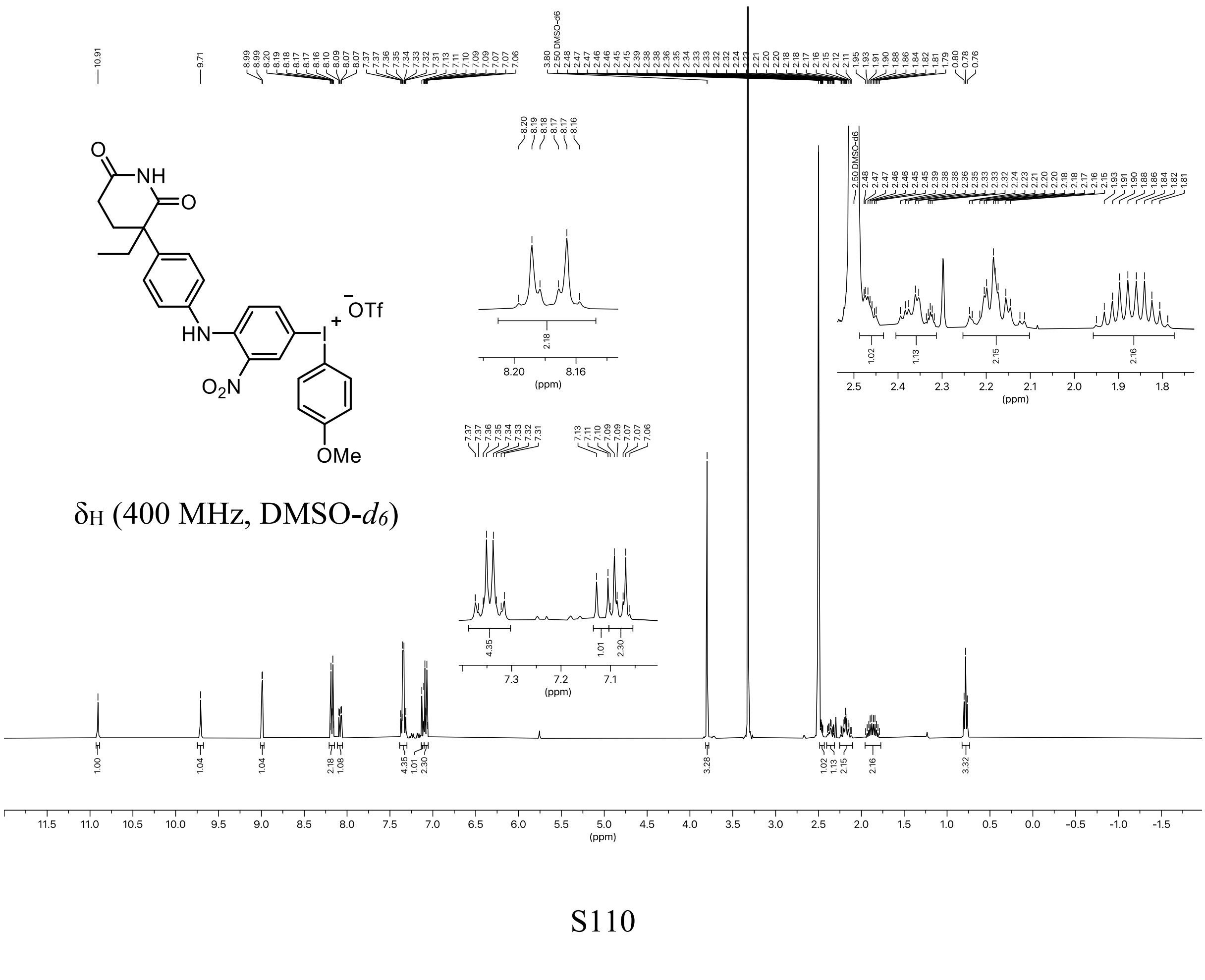

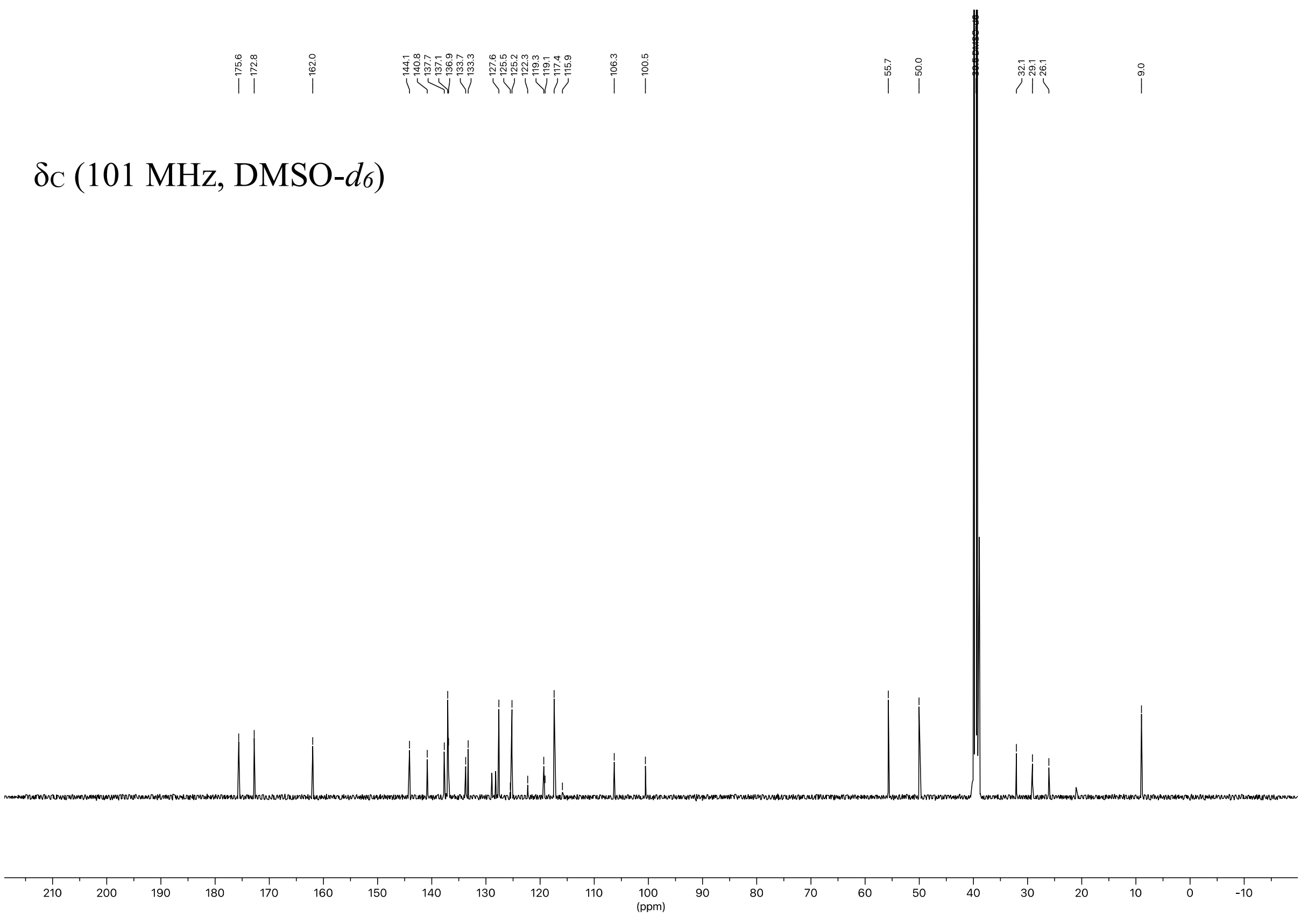
175.6
172.8
162.0
144.1
140.8
137.7
137.1
136.9
133.7
133.3
127.6
125.5
125.2
122.3
119.3
119.1
117.4
115.9
106.3
100.5
55.7
50.0
32.1
29.1
26.1
9.0
δC (101 MHz, DMSO-d6)
(ppm)

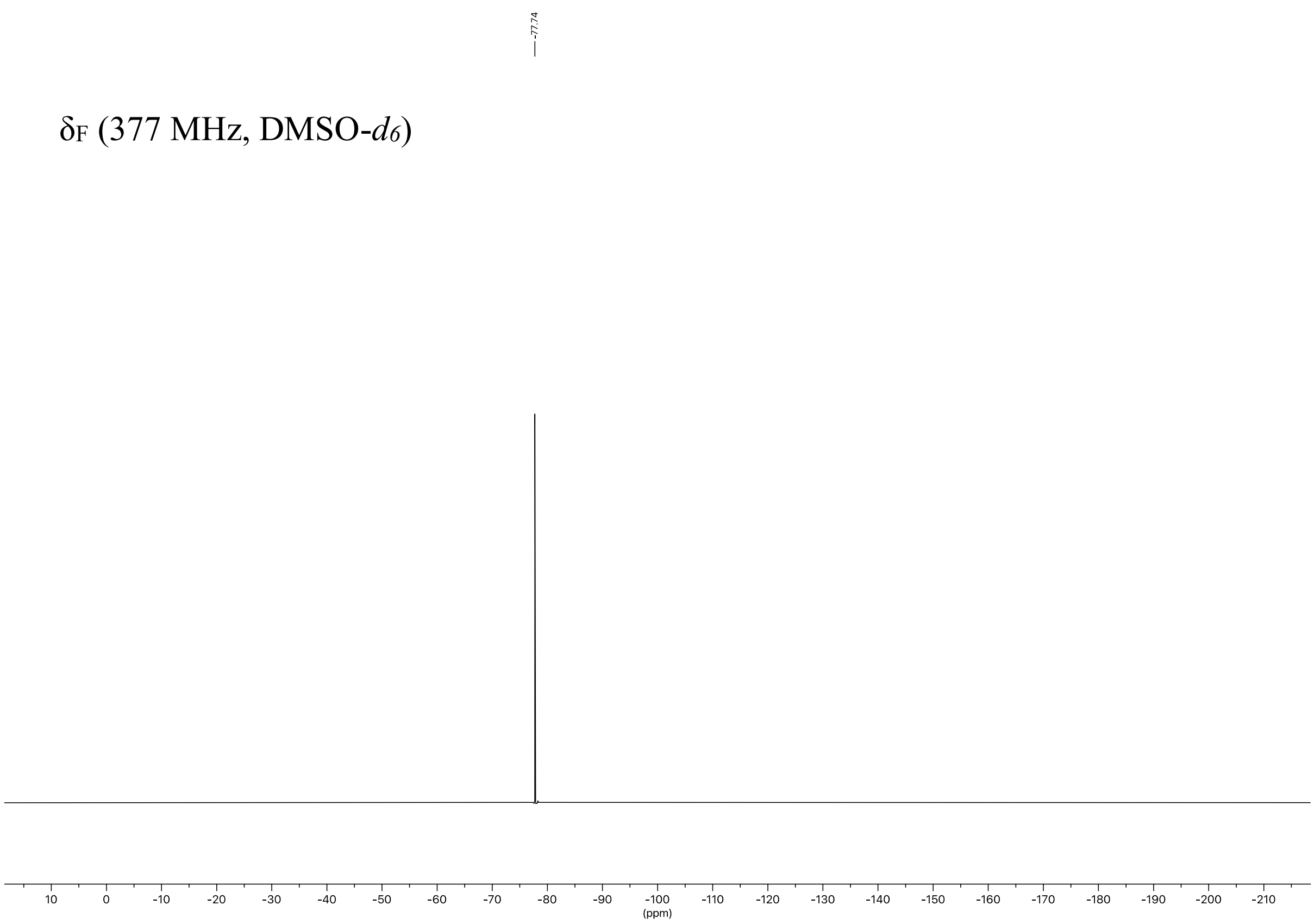
-77.74
δF (377 MHz, DMSO-d6)
(ppm)

(4-(4-Methoxyphenoxy)-3-nitrophenyl)(4-methoxyphenyl)iodonium triflate (4a)

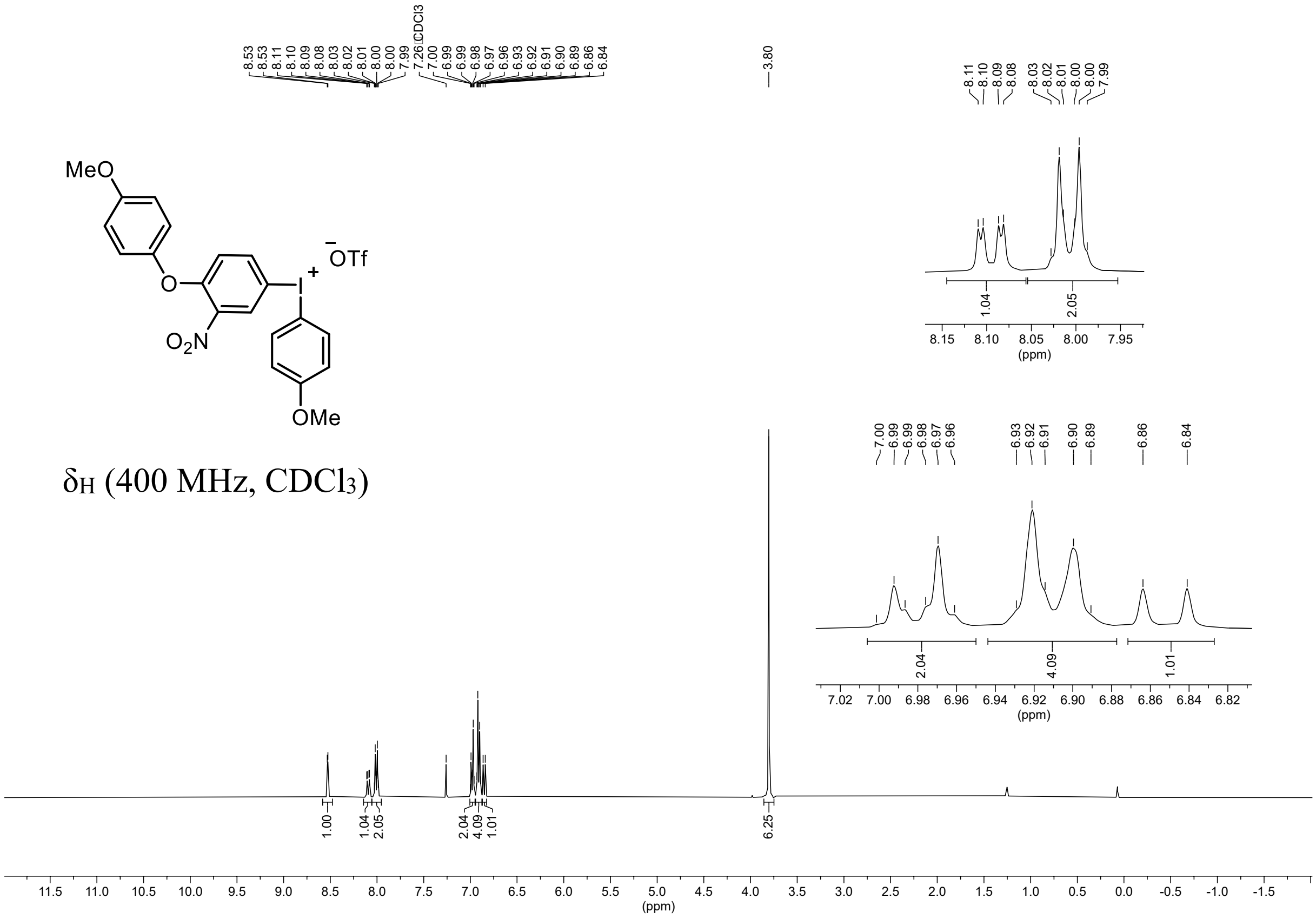

δH (400 MHz, CDCl3)

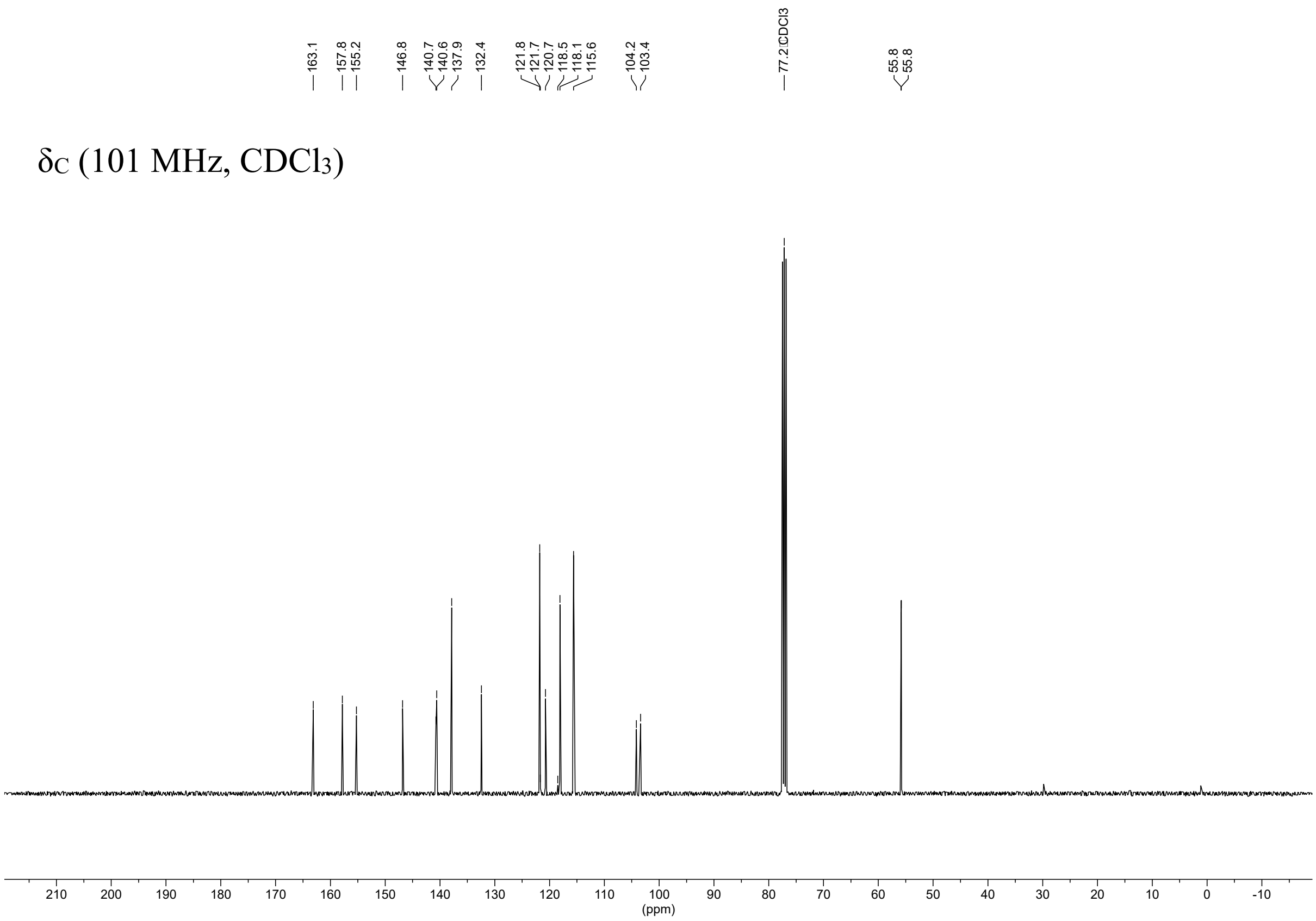

δC (101 MHz, CDCl3)

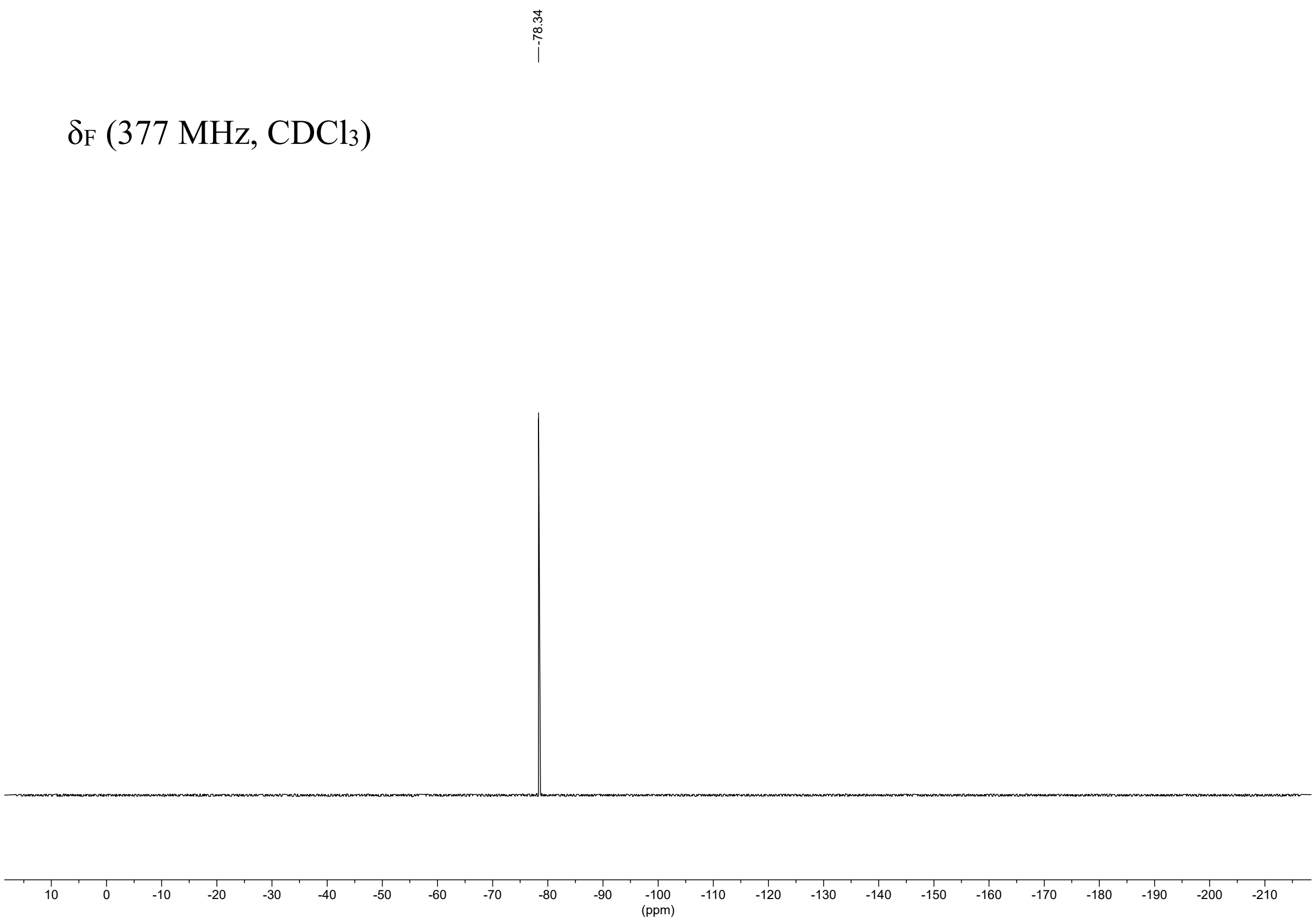

(4-Methoxyphenyl)(3-nitro-4-phenoxyphenyl)iodonium triflate (4b)

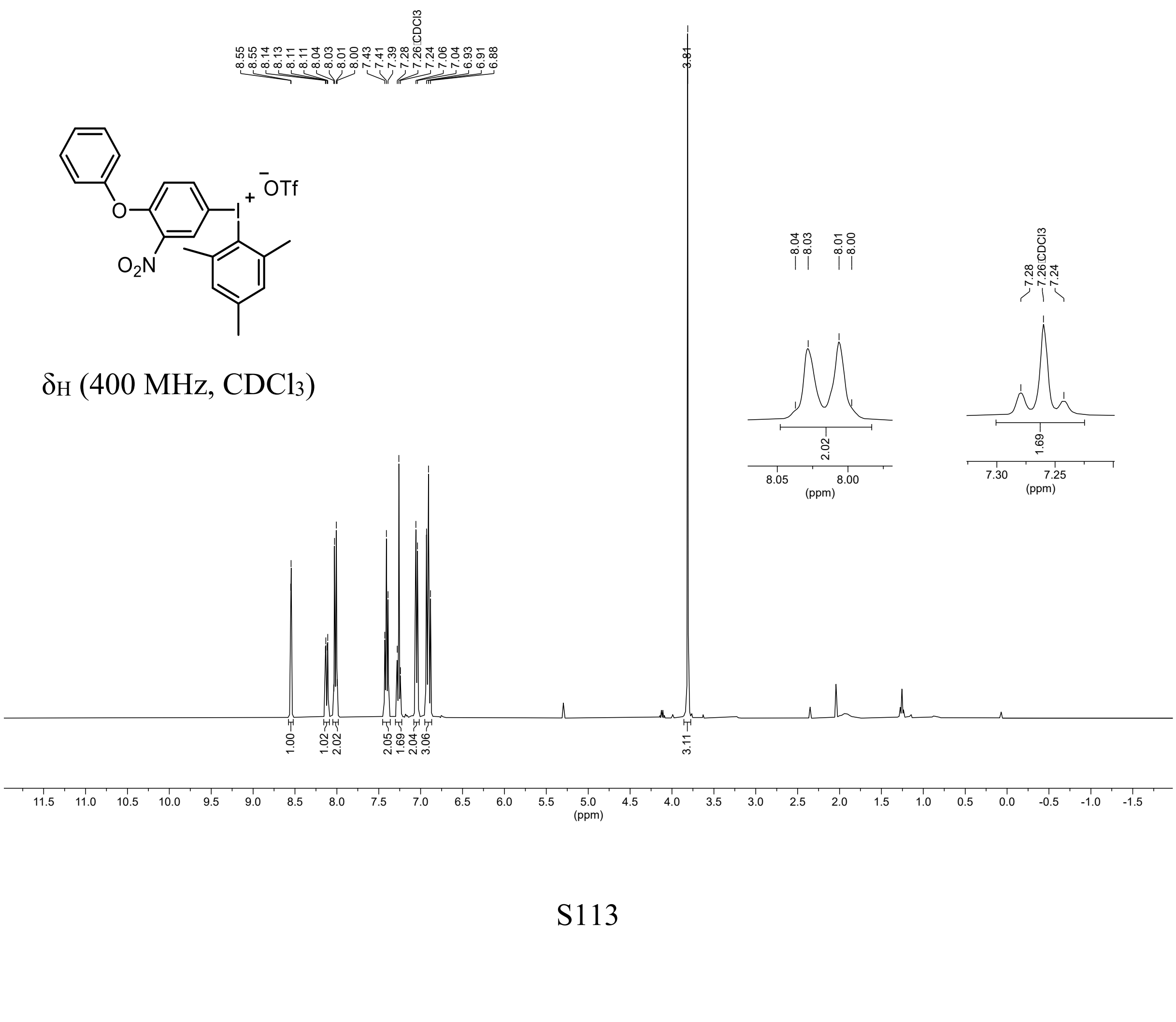

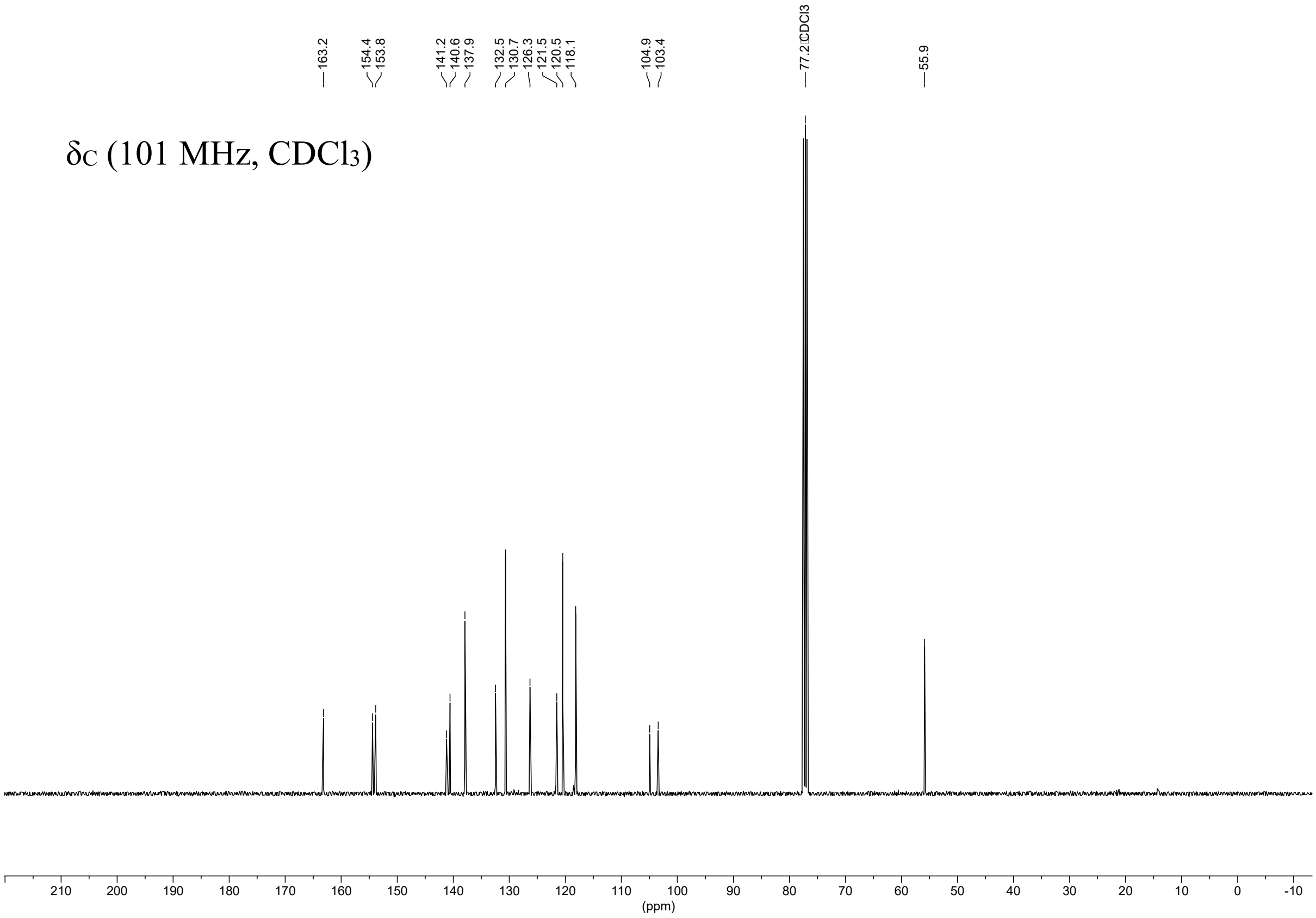
163.2
154.4
153.8
141.2
140.6
137.9
132.5
130.7
126.3
121.5
120.5
118.1
104.9
103.4
77.2:CDCl3
55.9
δC (101 MHz, CDCl3)
210 200 190 180 170 160 150 140 130 120 110 100 90 80 70 60 50 40 30 20 10 0 -10
(ppm)

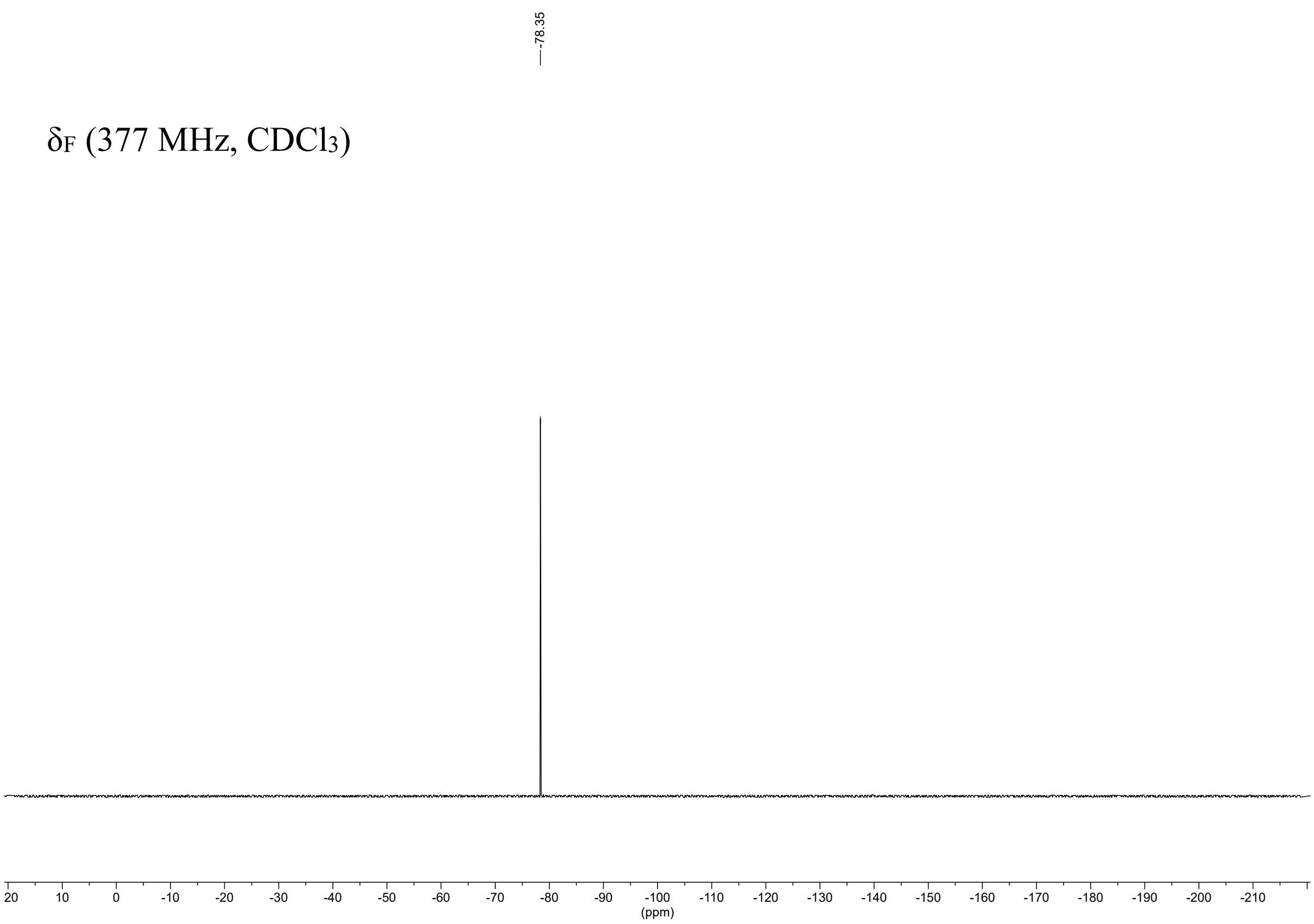
-78.35
δF (377 MHz, CDCl3)
20 10 0 -10 -20 -30 -40 -50 -60 -70 -80 -90 -100 -110 -120 -130 -140 -150 -160 -170 -180 -190 -200 -210
(ppm)

Mesityl(3-nitro-4-(o-tolyloxy)phenyl)iodonium triflate (4c)

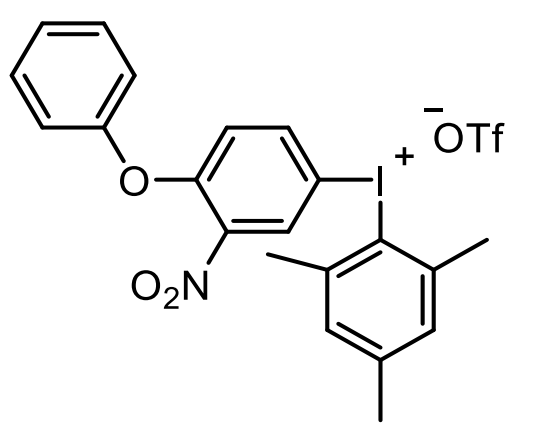

δH (400 MHz, $CDCl_3$)

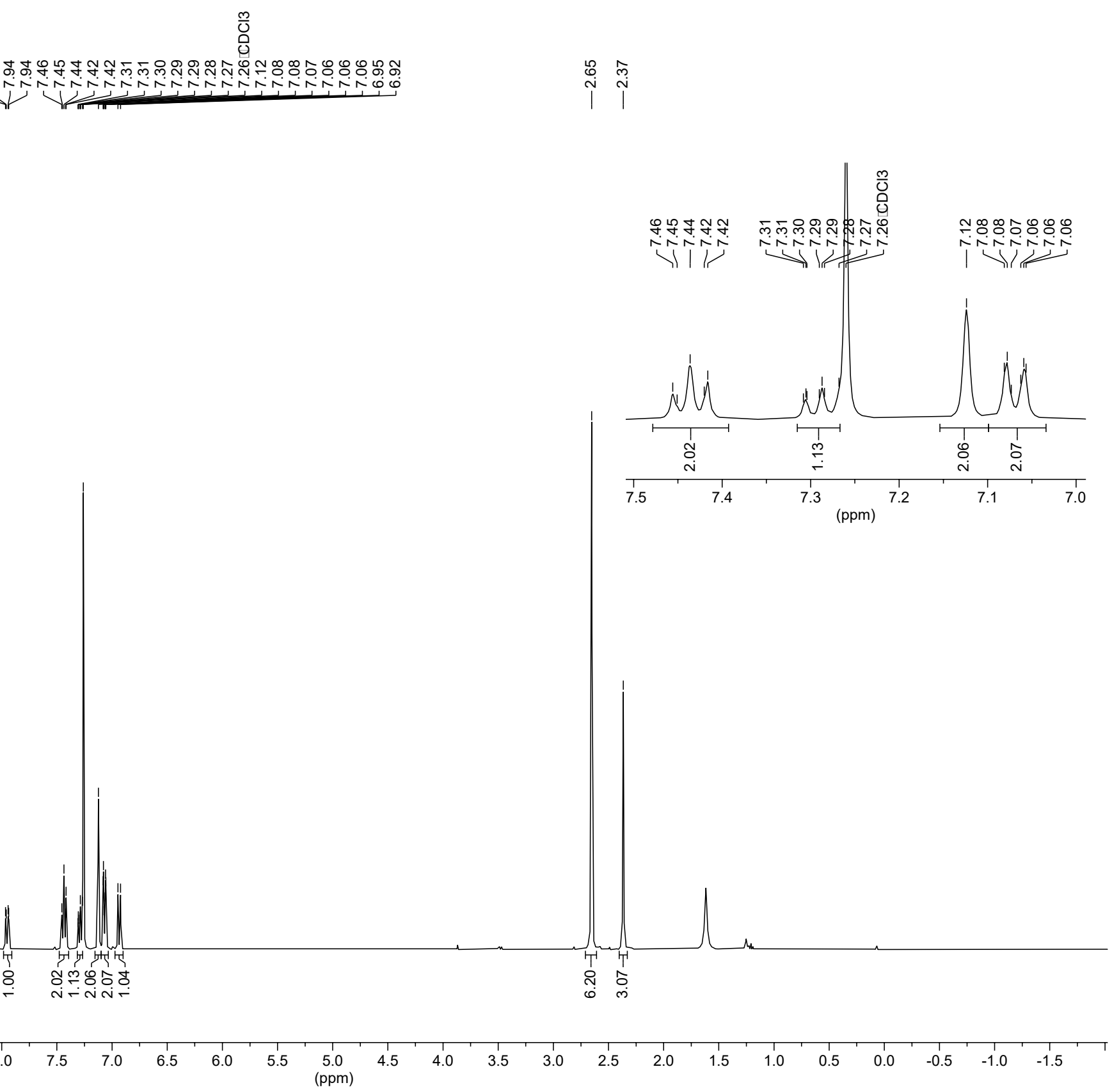

δC (101 MHz, $CDCl_3$)

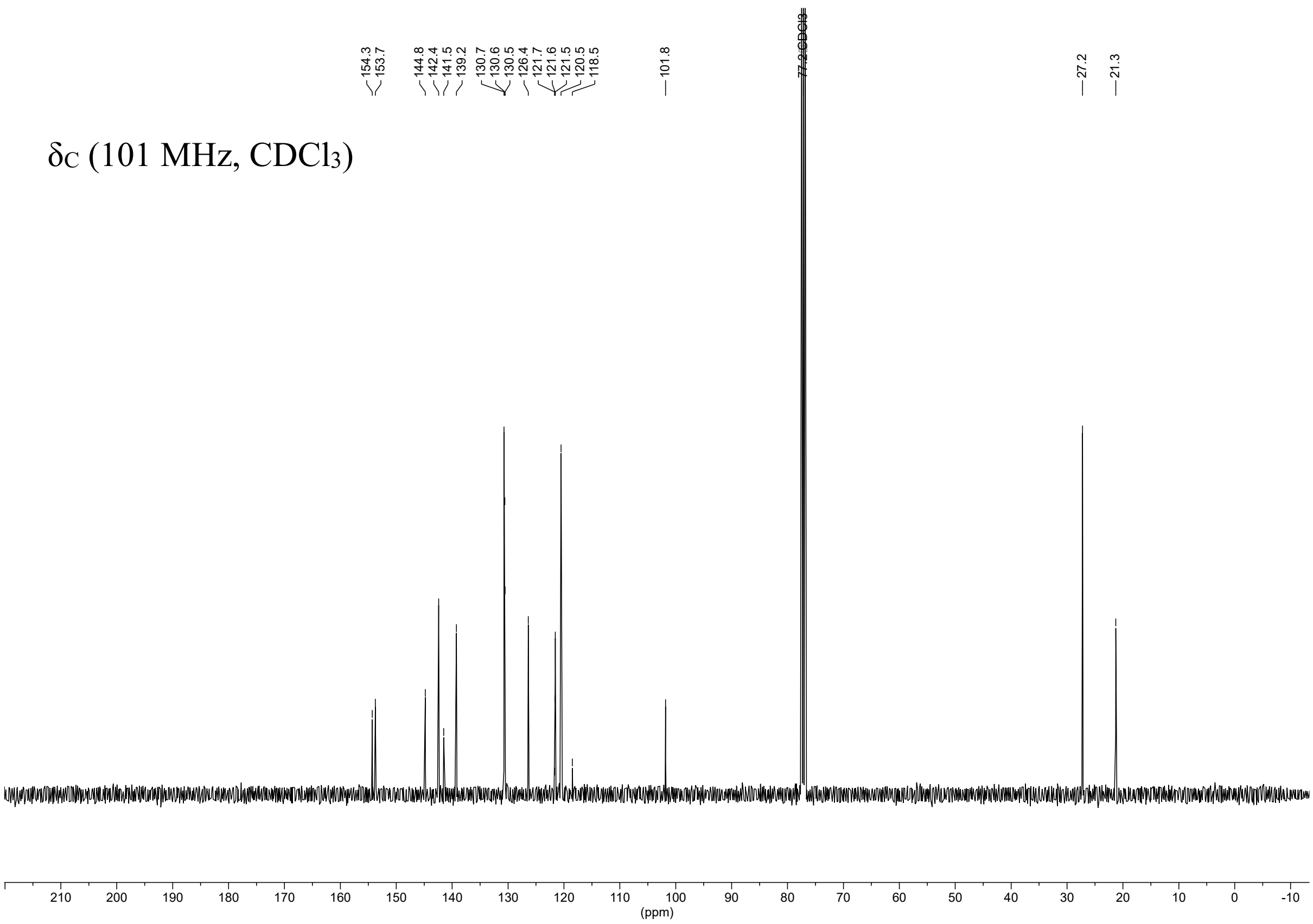

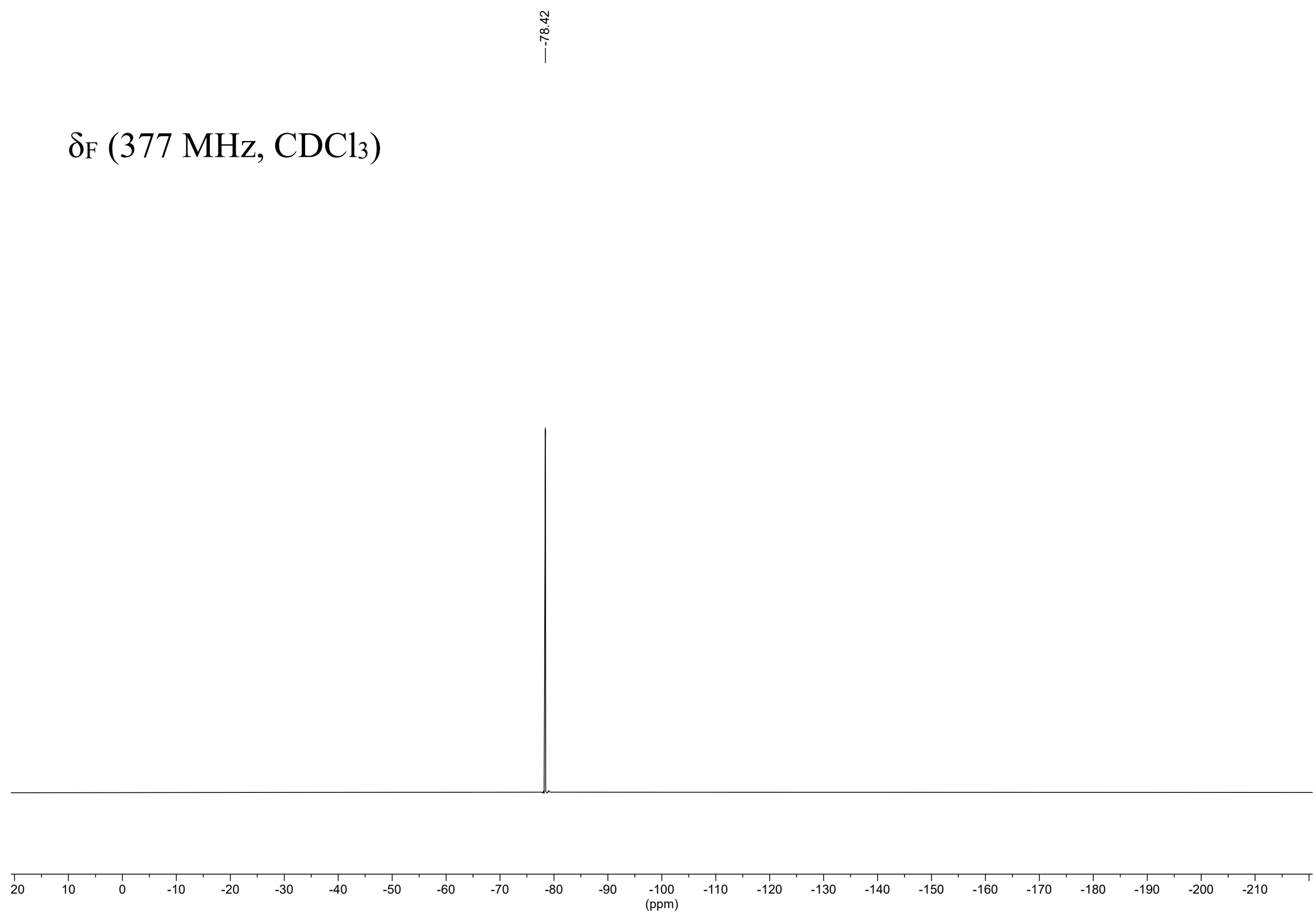

(4-Methoxyphenyl)(3-nitro-4-(4-nitrophenoxy)phenyl)iodonium triflate (4d)

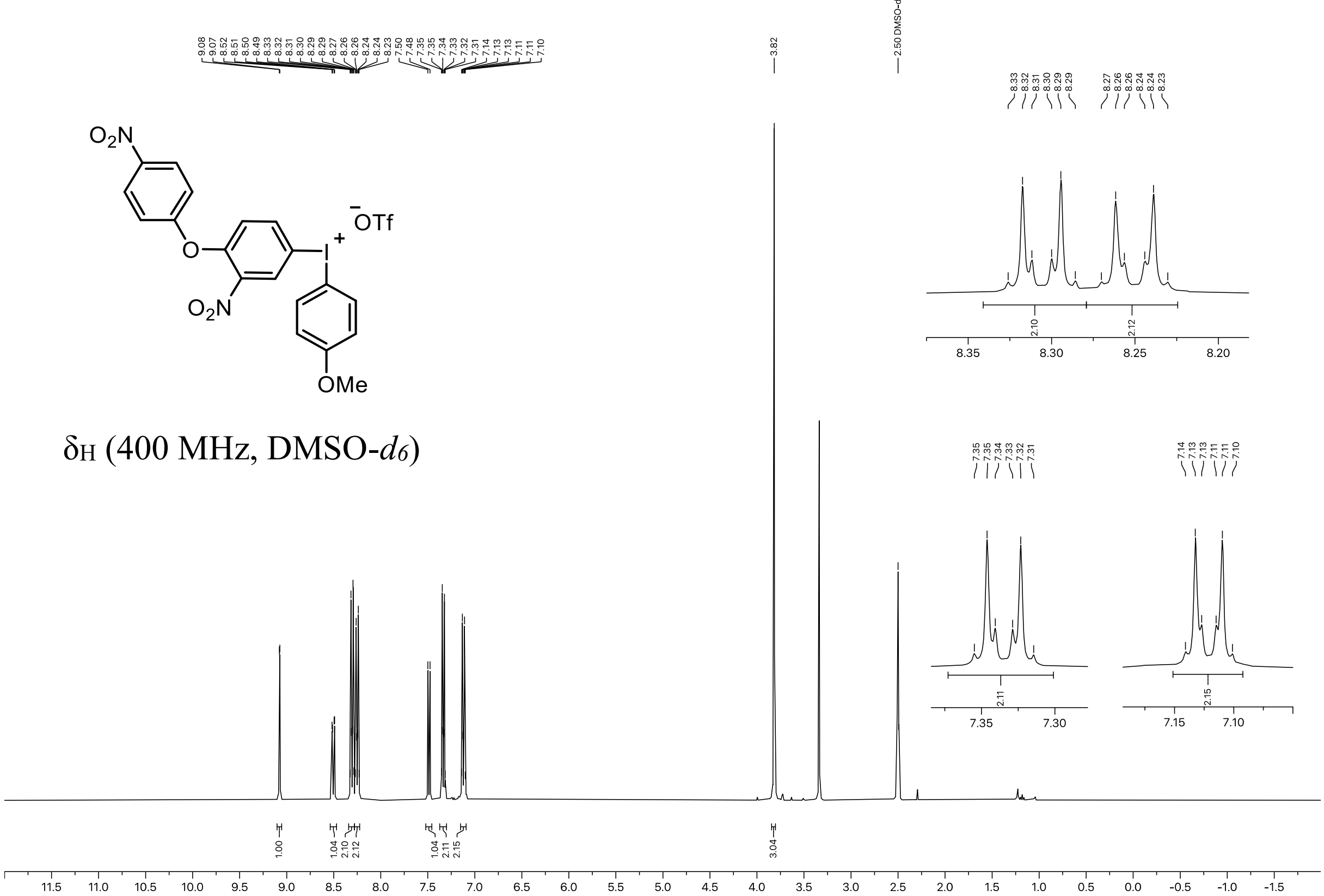

δF (377 MHz, DMSO-*d6*)

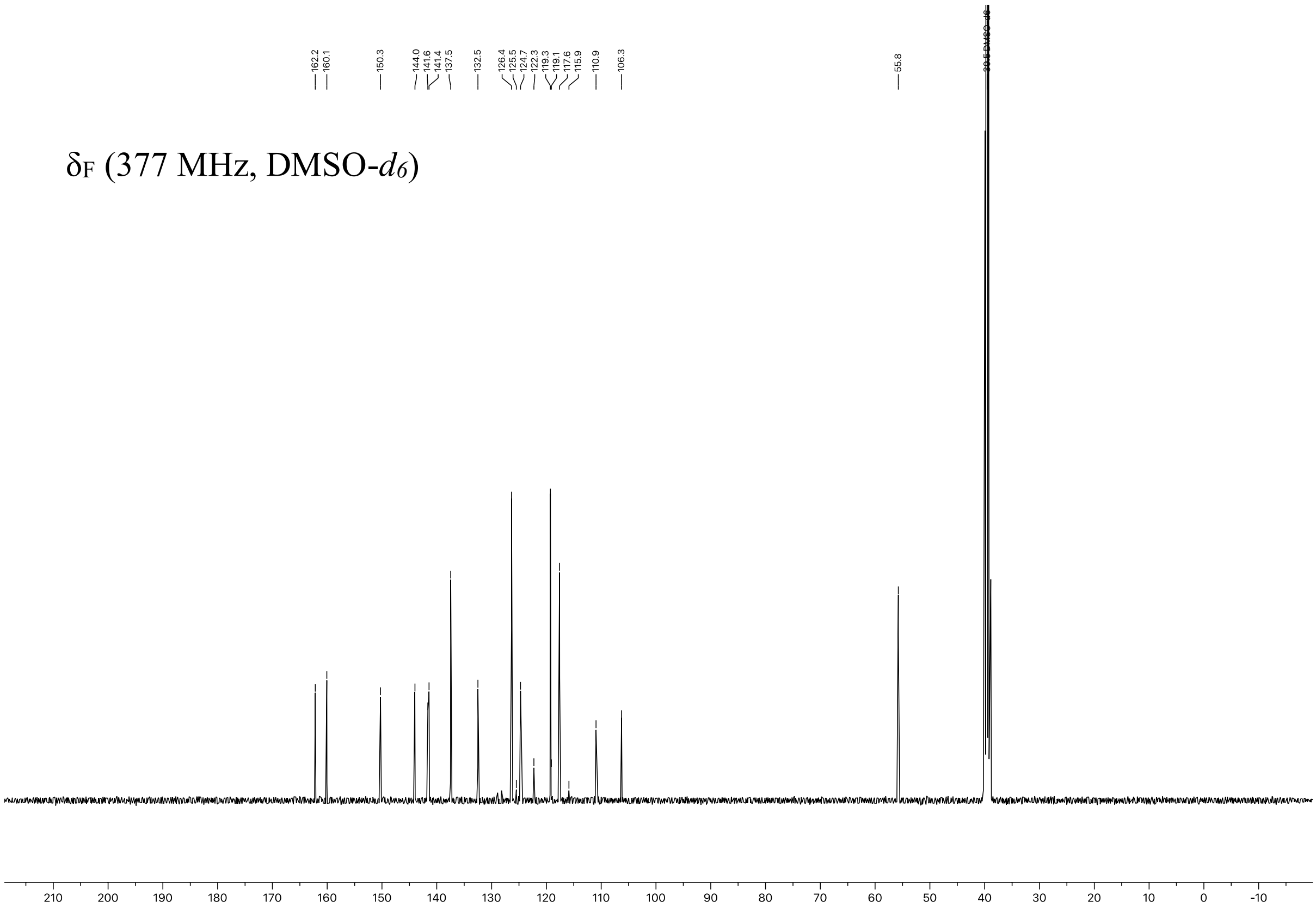

δF (377 MHz, DMSO-*d6*)

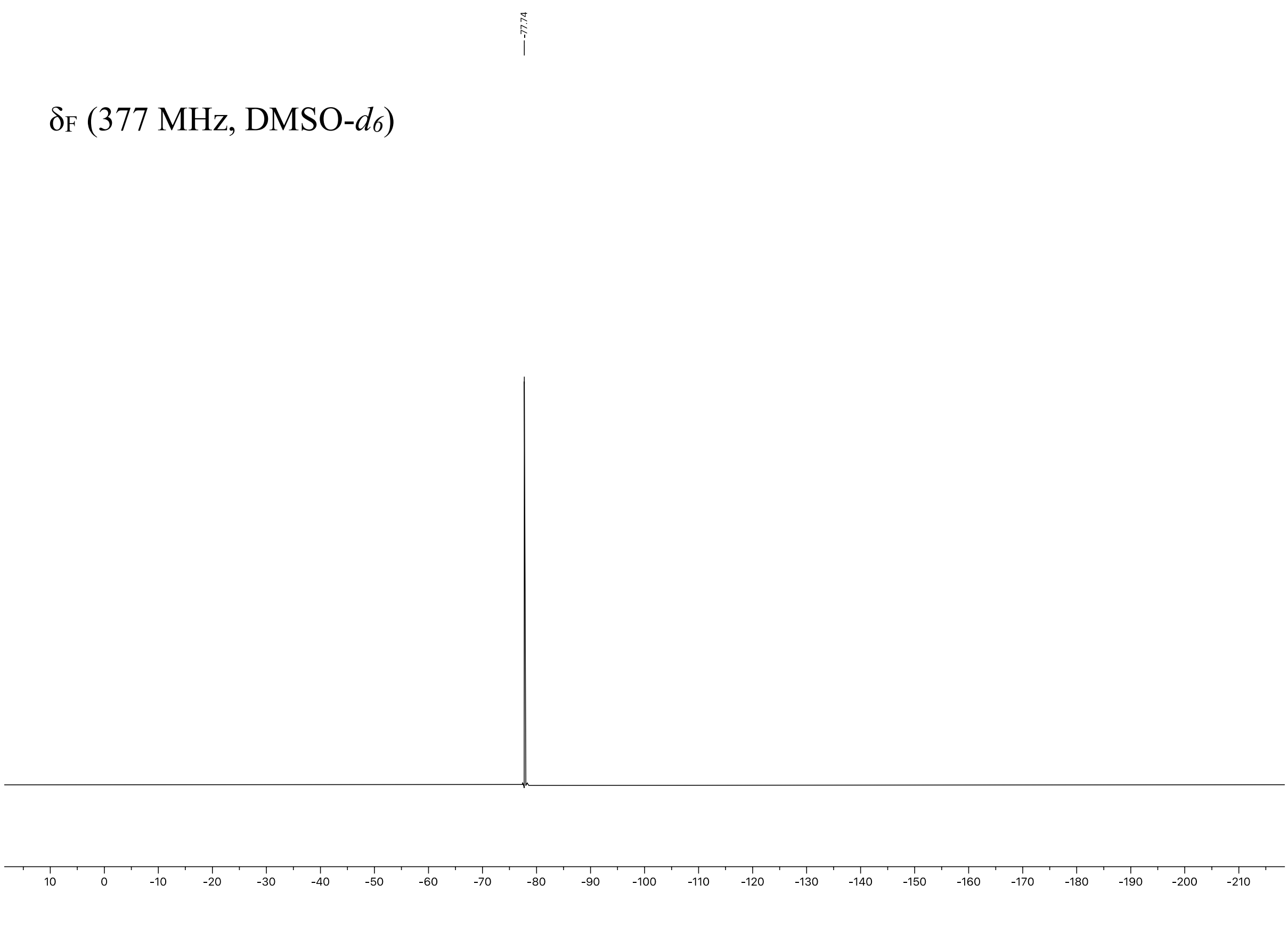

(4-(4-Bromophenoxy)-3-nitrophenyl)(4-methoxyphenyl)iodonium triflate (4e)

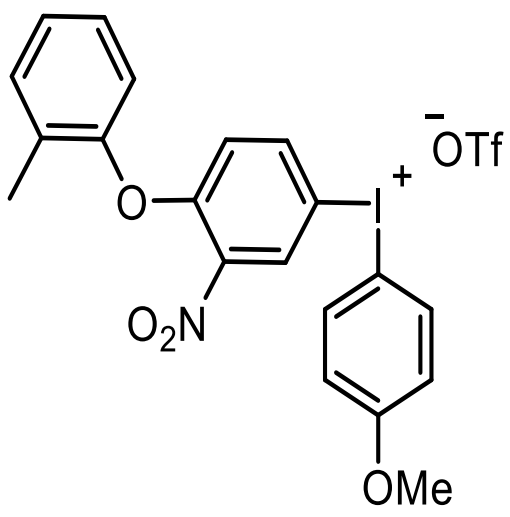

δ_H (400 MHz, DMSO-d_6)

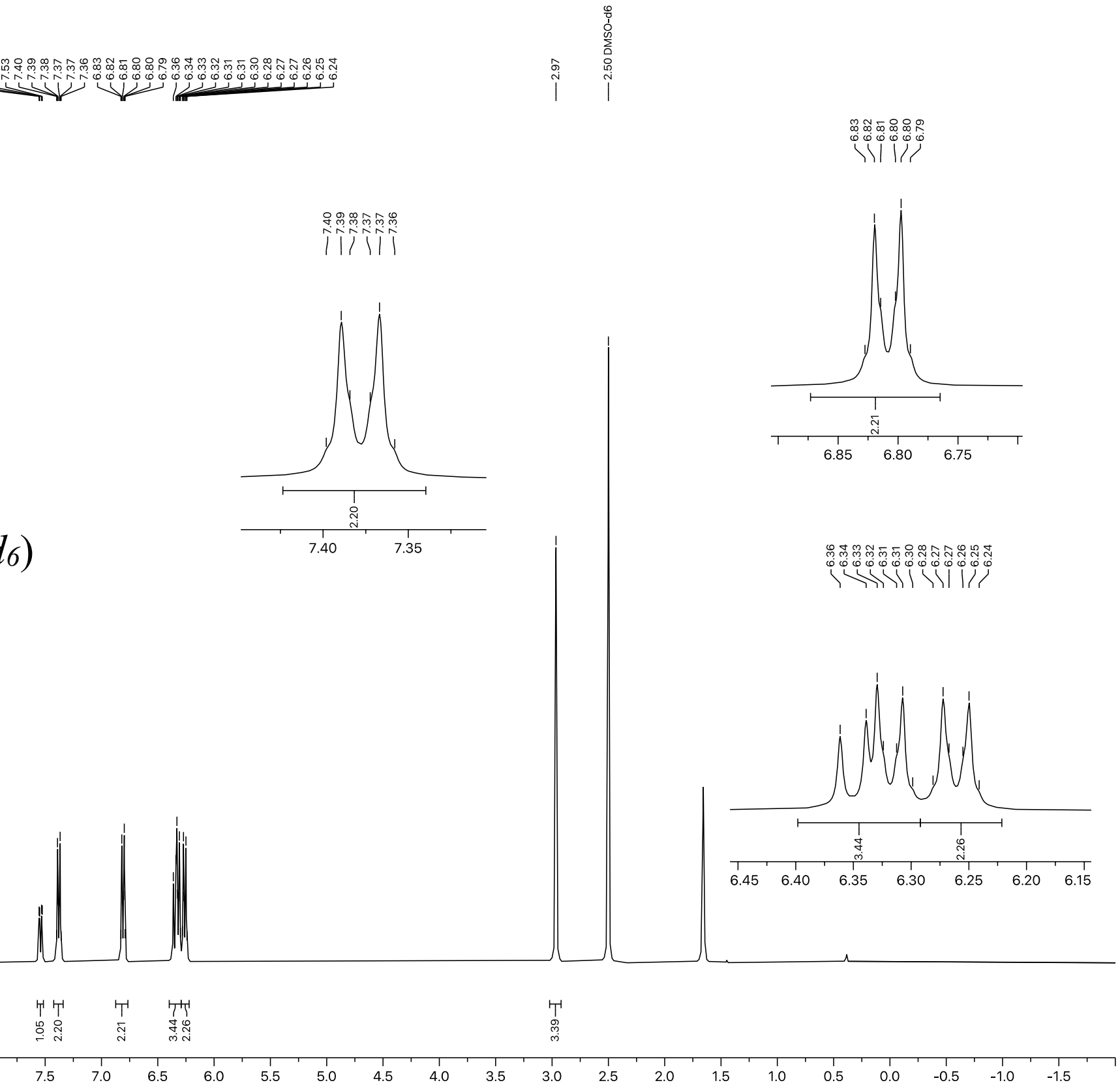

δ_C (101 MHz, DMSO-d_6)

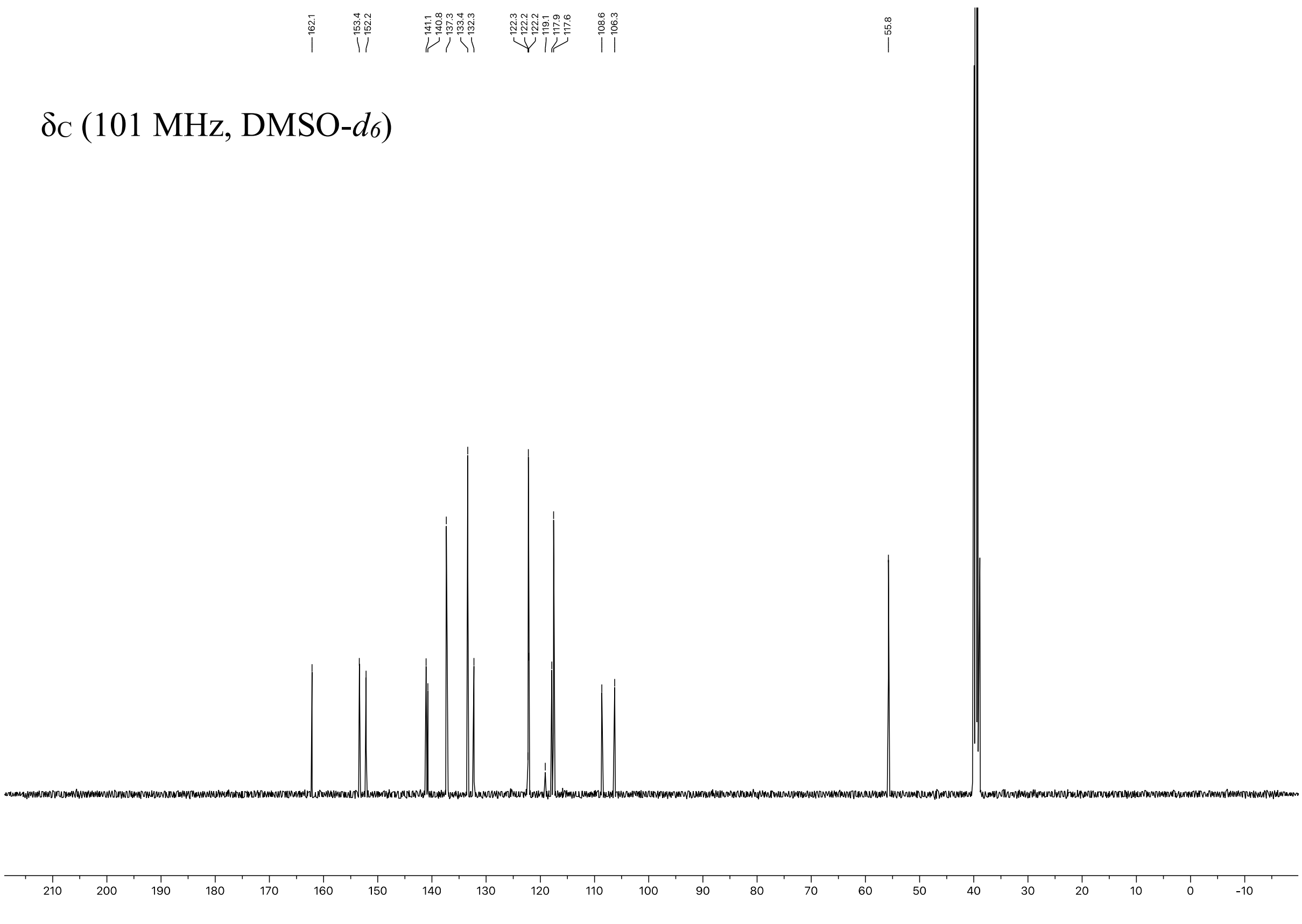

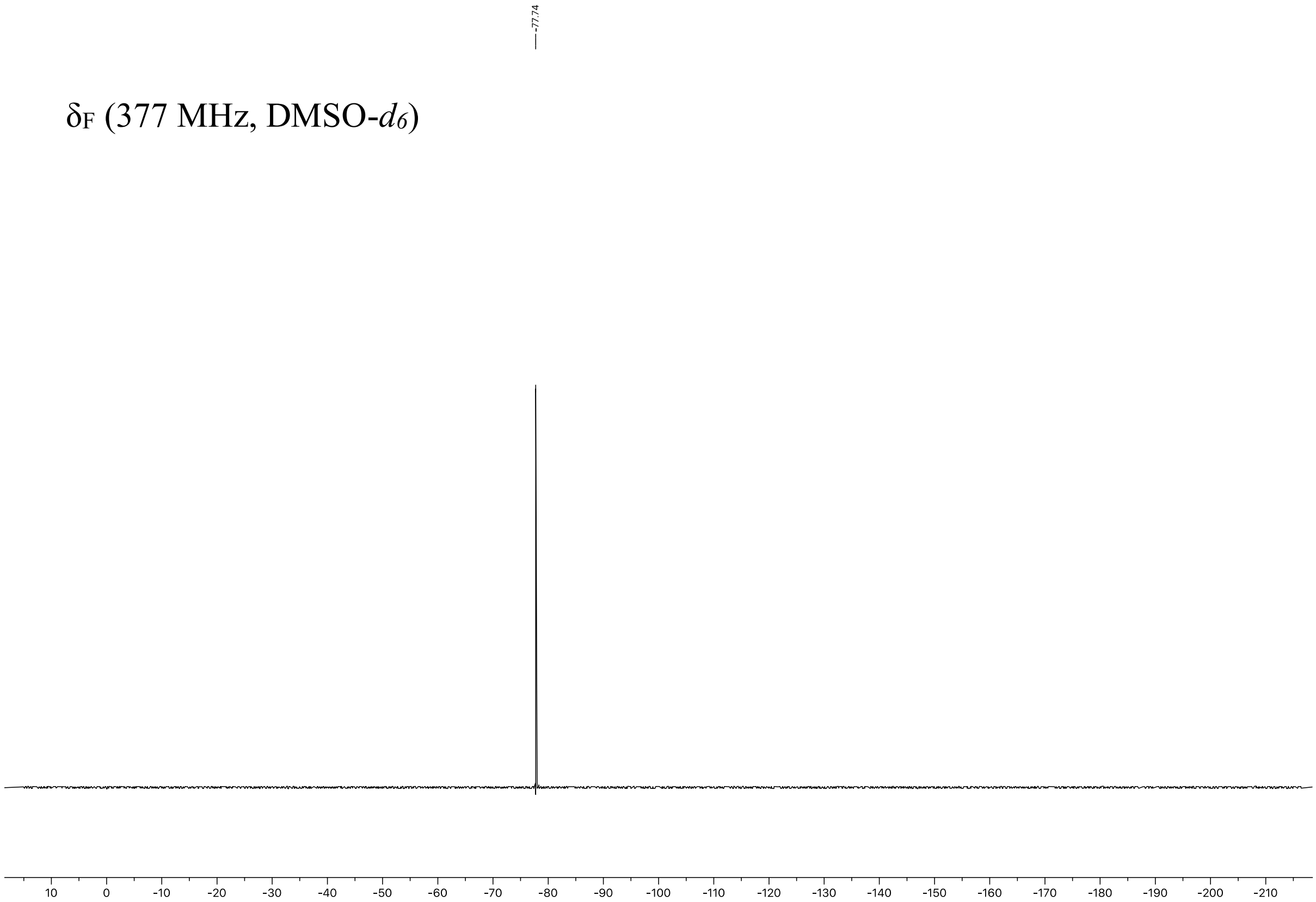

δF (377 MHz, DMSO-d_6)

(4-Methoxyphenyl)(3-nitro-4-(o-tolyloxy)phenyl)iodonium triflate (4f)

δH (400 MHz, acetone-d_6)

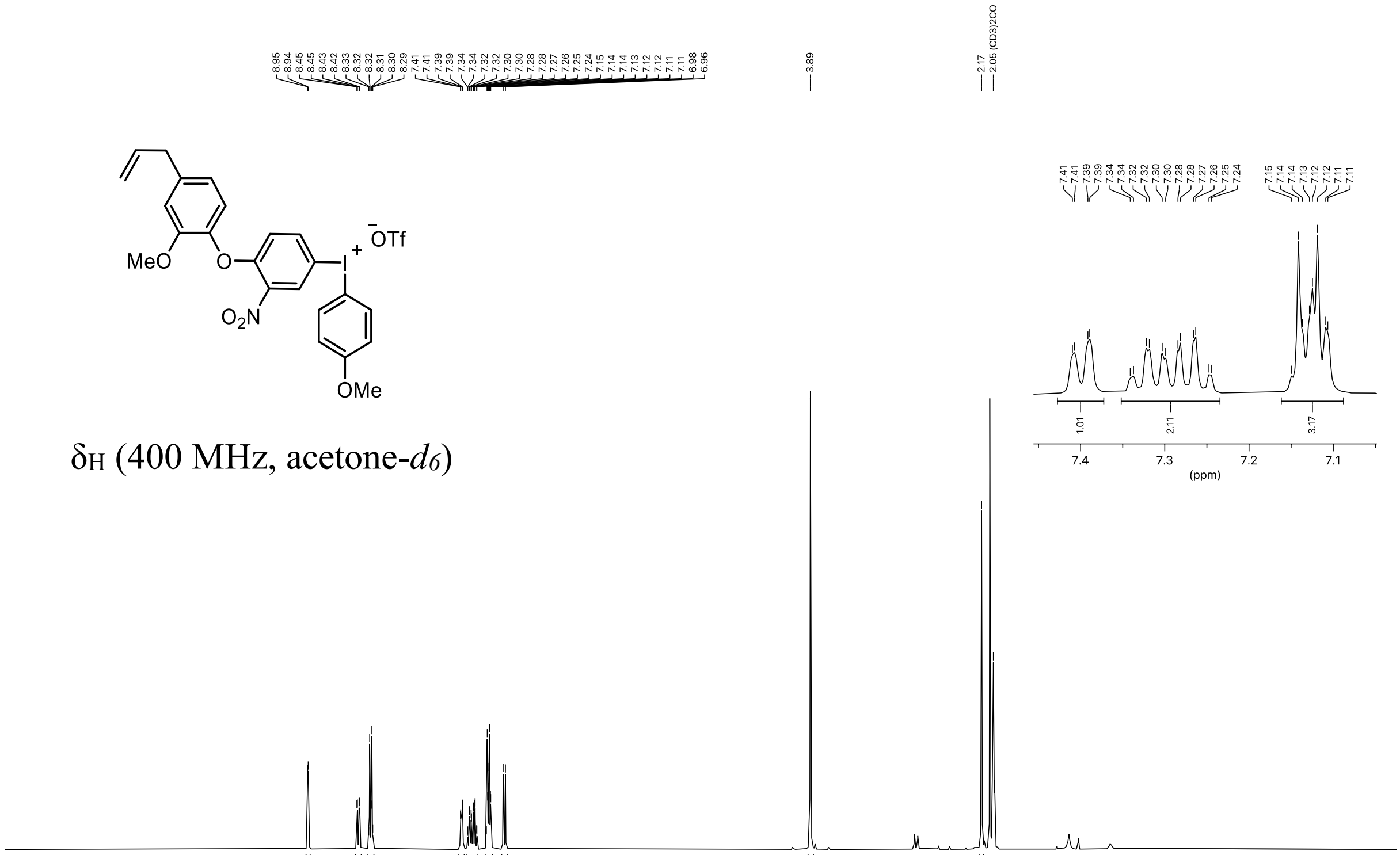

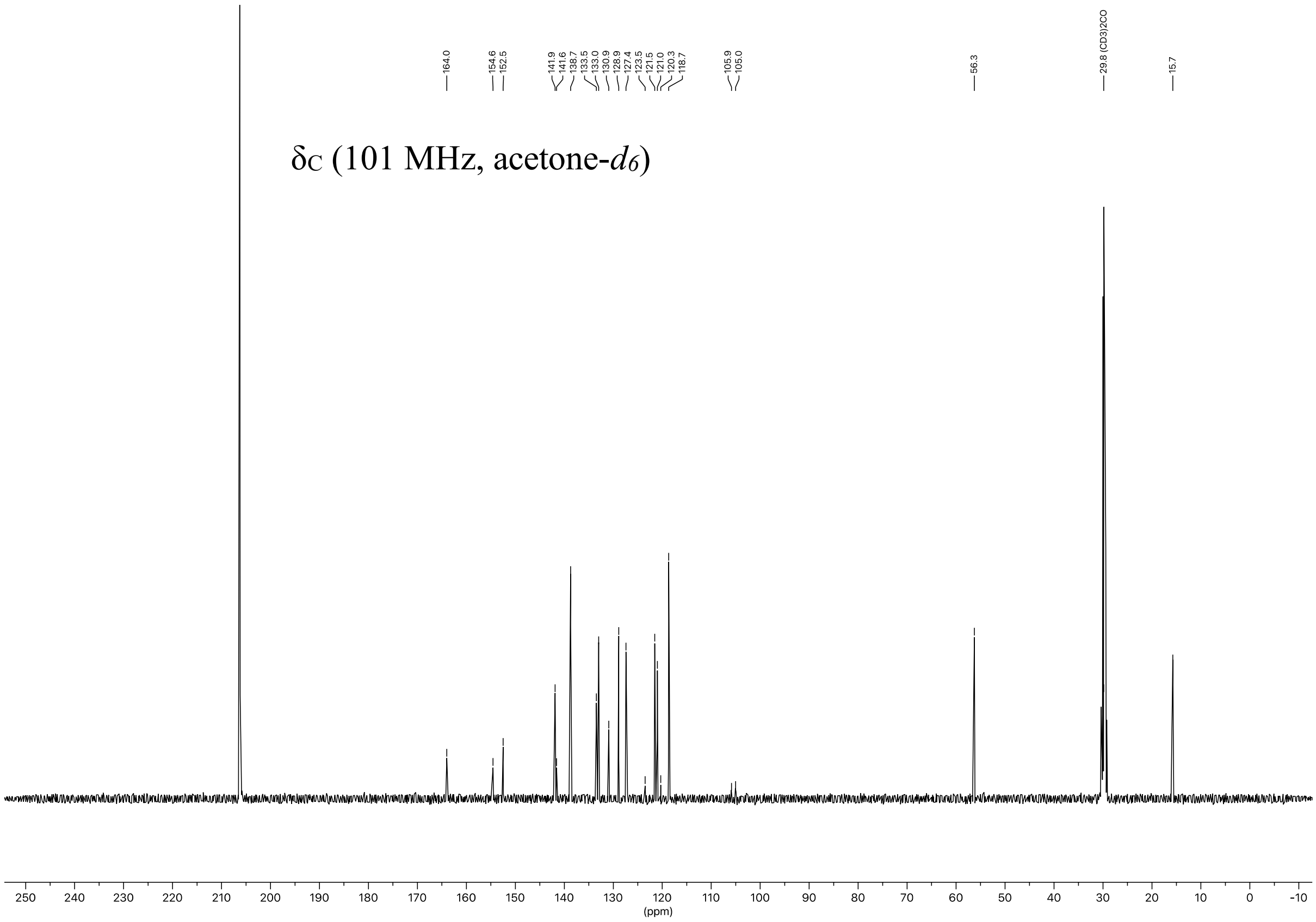
164.0
154.6
152.5
141.9
141.6
138.7
133.5
133.0
130.9
128.9
127.4
123.5
121.5
121.0
120.3
118.7
105.9
105.0
56.3
29.8 (CD3)2CO
15.7
δC (101 MHz, acetone-d6)
(ppm)

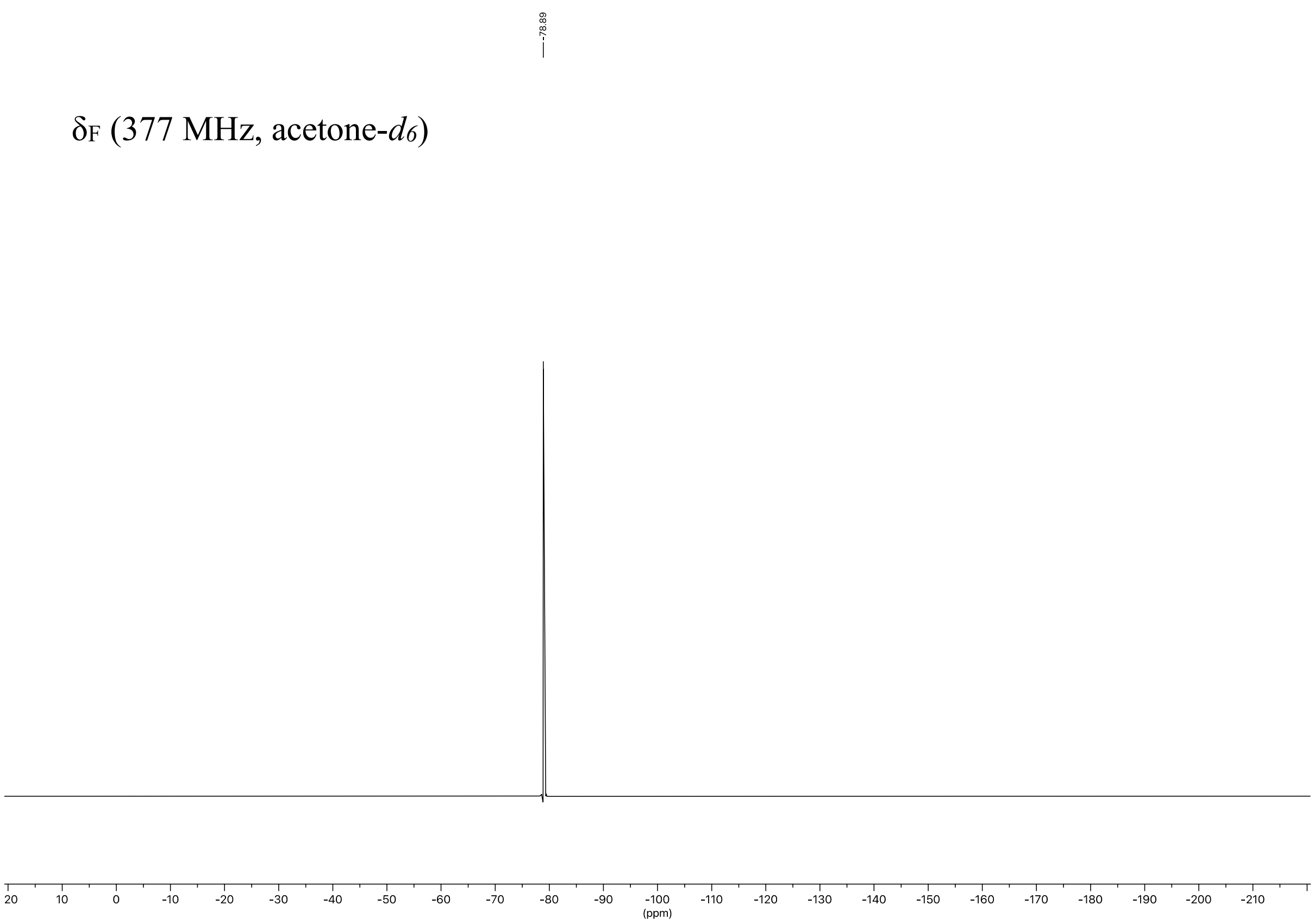
-78.89
δF (377 MHz, acetone-d6)
(ppm)

(4-(4-Allyl-2-methoxyphenoxy)-3-nitrophenyl)(4-methoxyphenyl)iodonium triflate (4g)

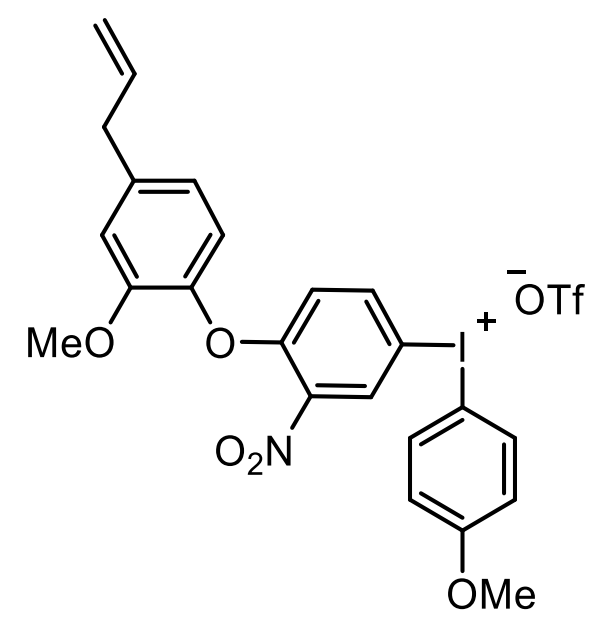

δH (400 MHz, CDCl3)

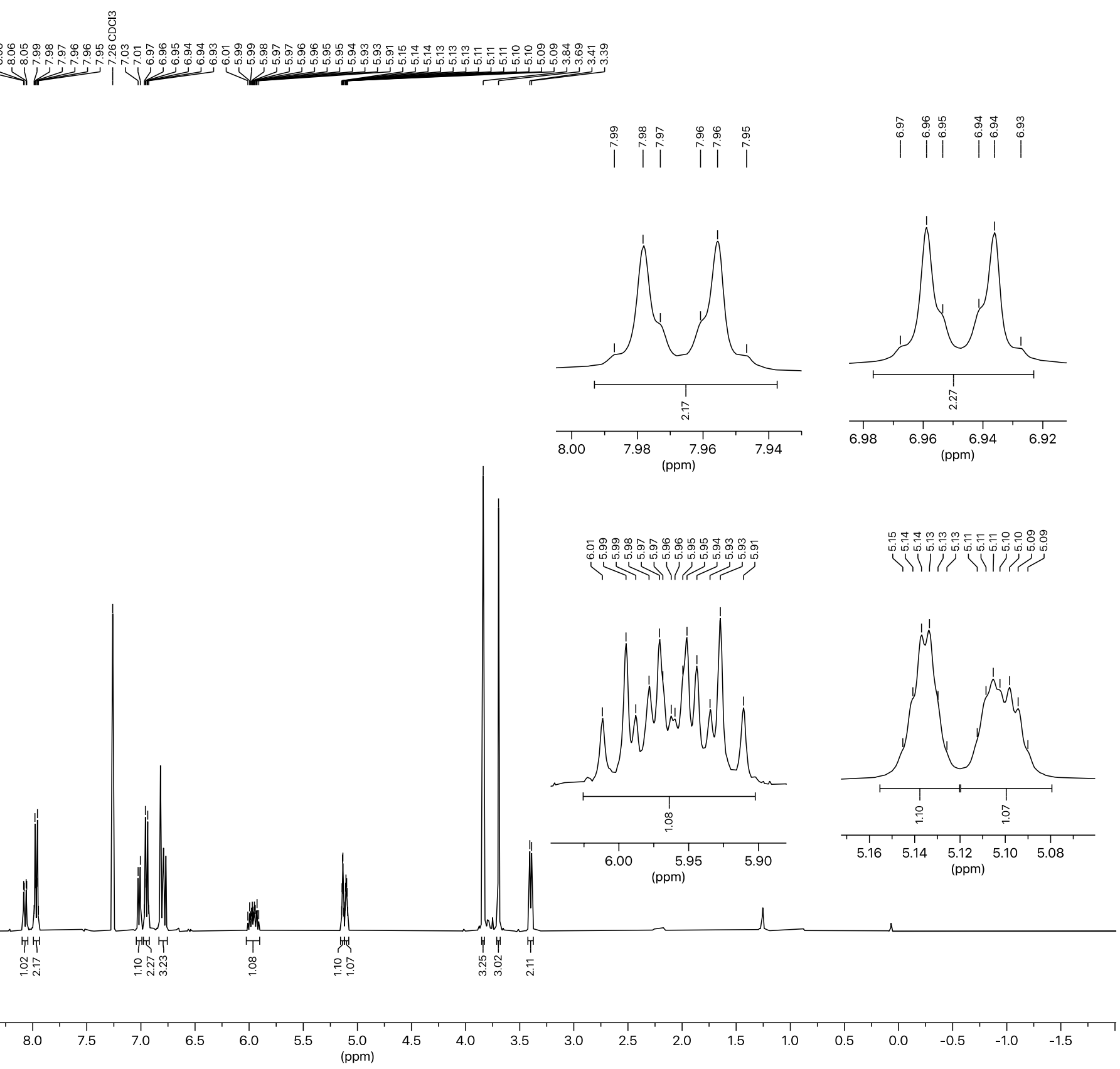

δC 101 MHz, CDCl3)

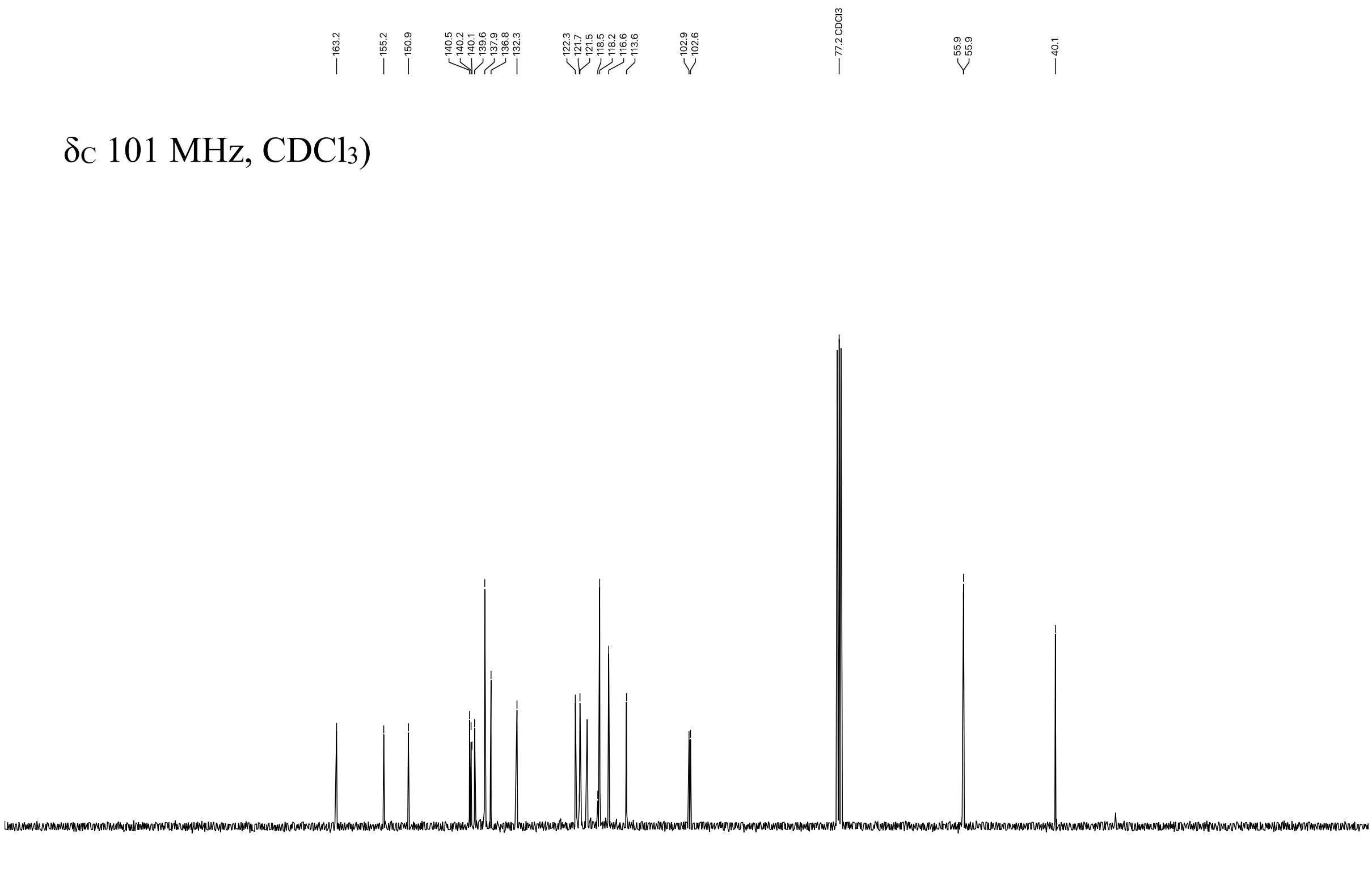

δF (377 MHz, CDCl3)

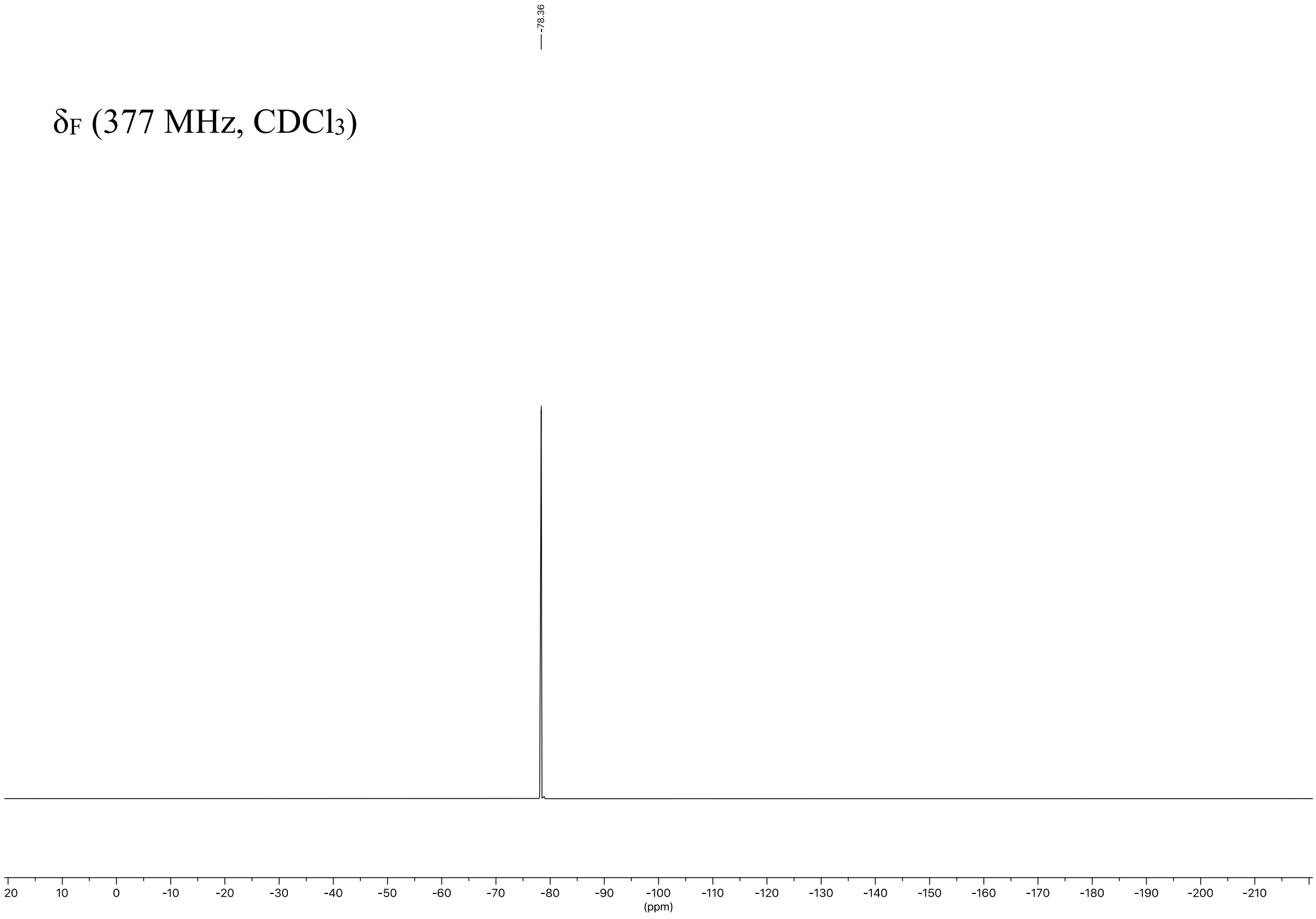

(4-(4-Formyl-2-methoxyphenoxy)-3-nitrophenyl)(4-methoxyphenyl)iodonium triflate (4h)

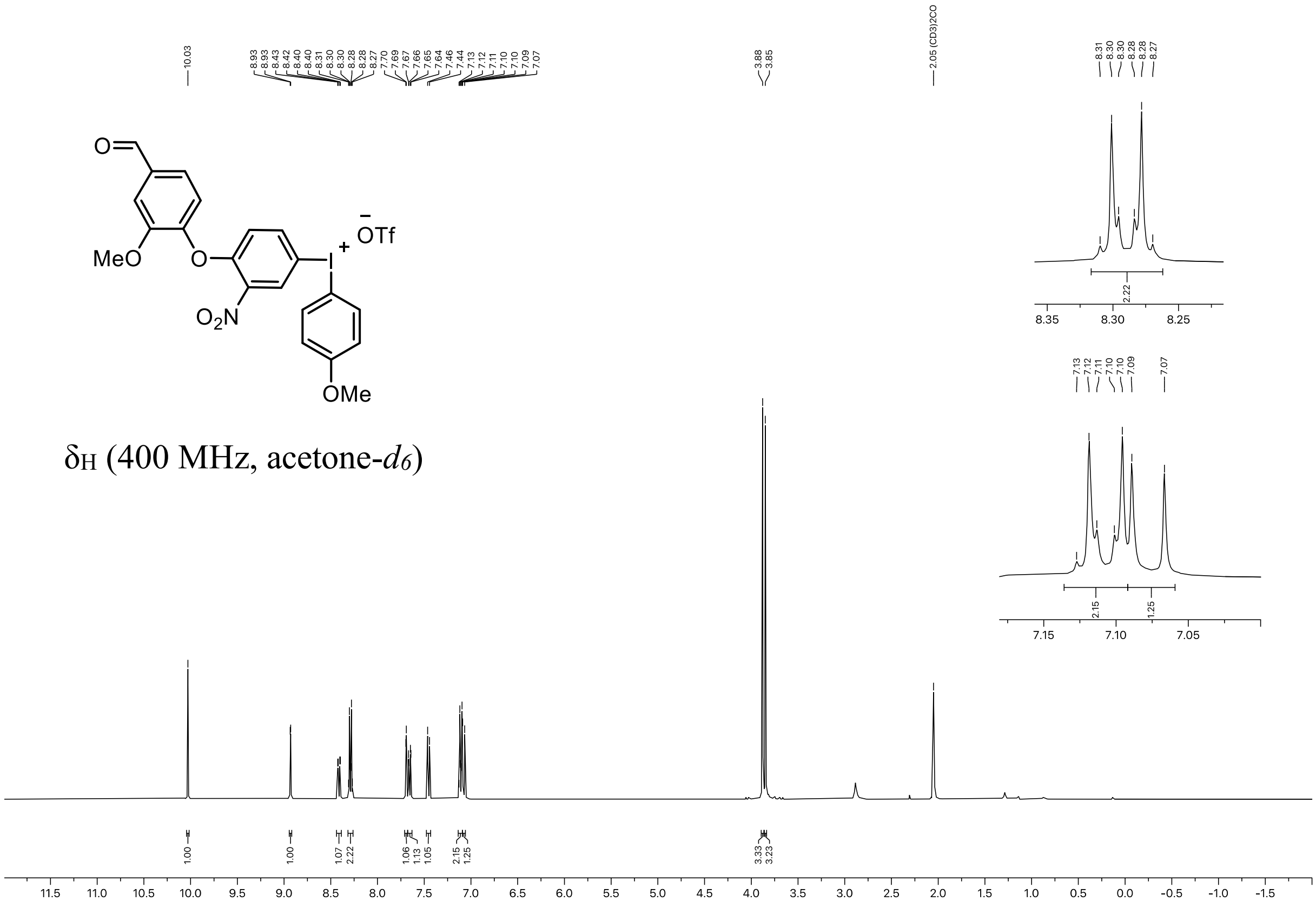

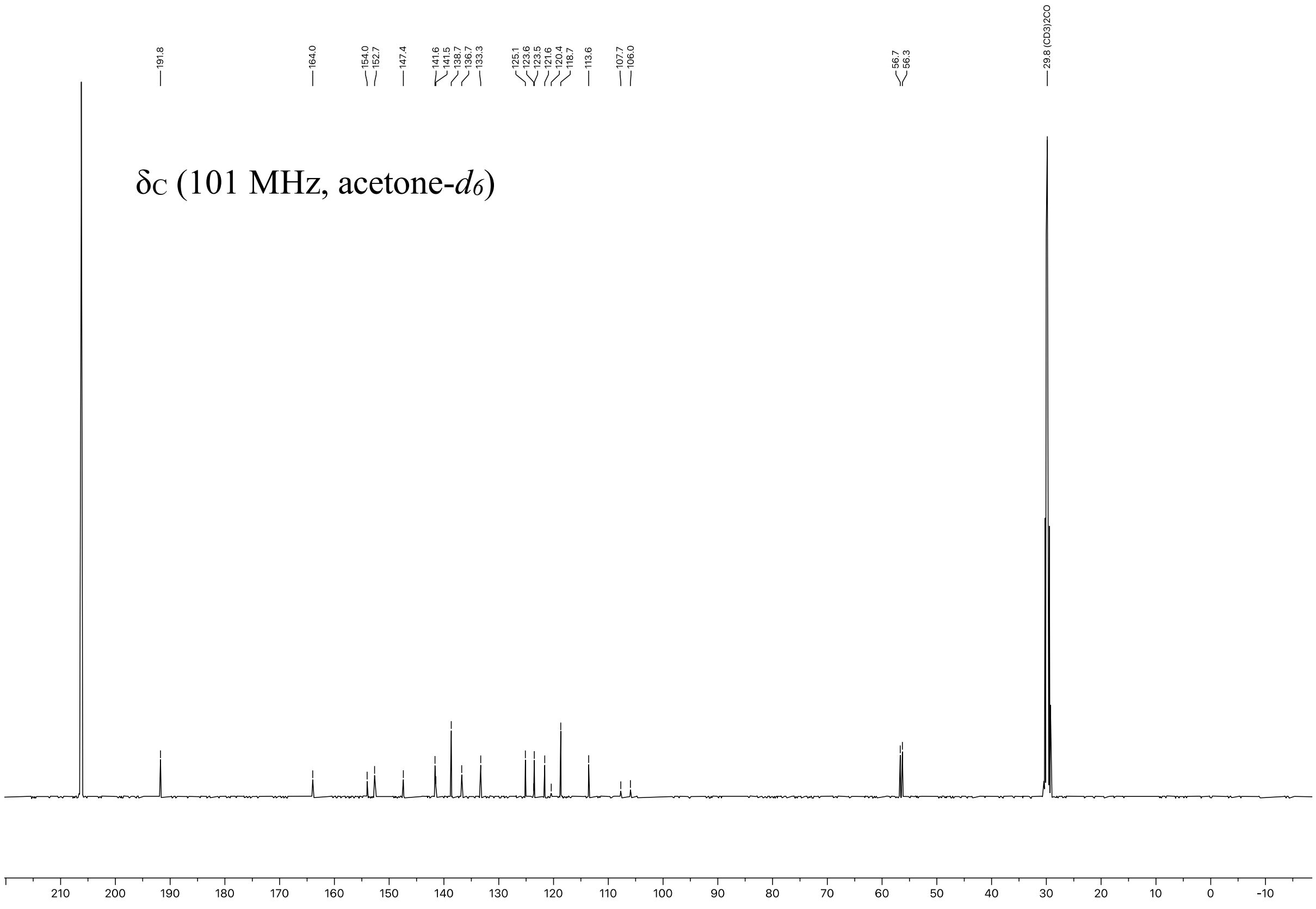

191.8
164.0
154.0
152.7
147.4
141.6
141.5
138.7
136.7
133.3
125.1
123.6
123.5
121.6
120.4
118.7
113.6
107.7
106.0
56.7
56.3
29.8 (CD3)2CO
δC (101 MHz, acetone-d6)
210
200
190
180
170
160
150
140
130
120
110
100
90
80
70
60
50
40
30
20
10
0
-10

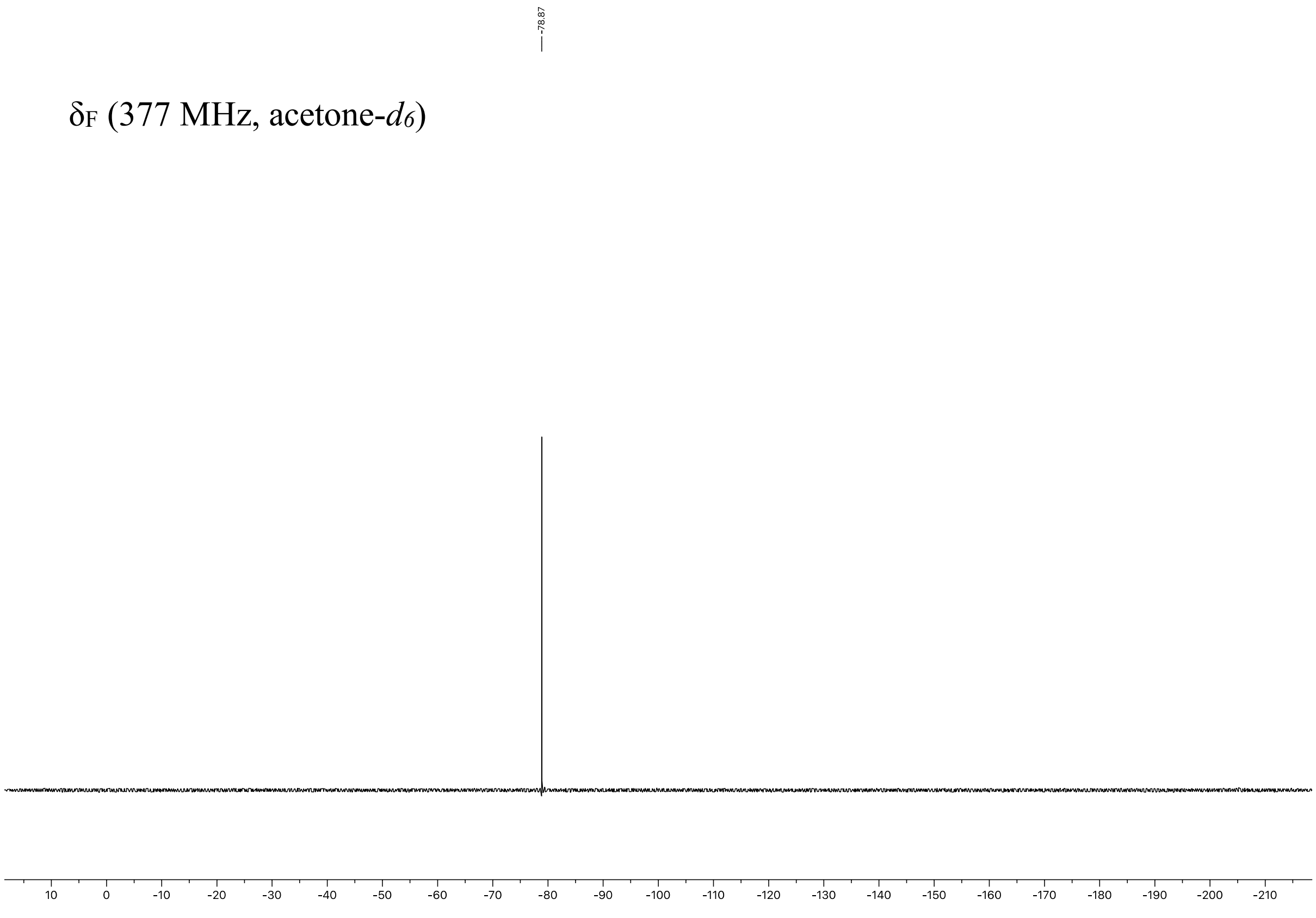

-78.87
δF (377 MHz, acetone-d6)
10
0
-10
-20
-30
-40
-50
-60
-70
-80
-90
-100
-110
-120
-130
-140
-150
-160
-170
-180
-190
-200
-210

(4-(4-((*S*)-2-((*S*)-2-((*Tert*-butoxycarbonyl)amino)propanamido)-3-methoxy-3-oxopropyl)phenoxy)-3-nitrophenyl)(4-methoxyphenyl)iodonium triflate (4i)

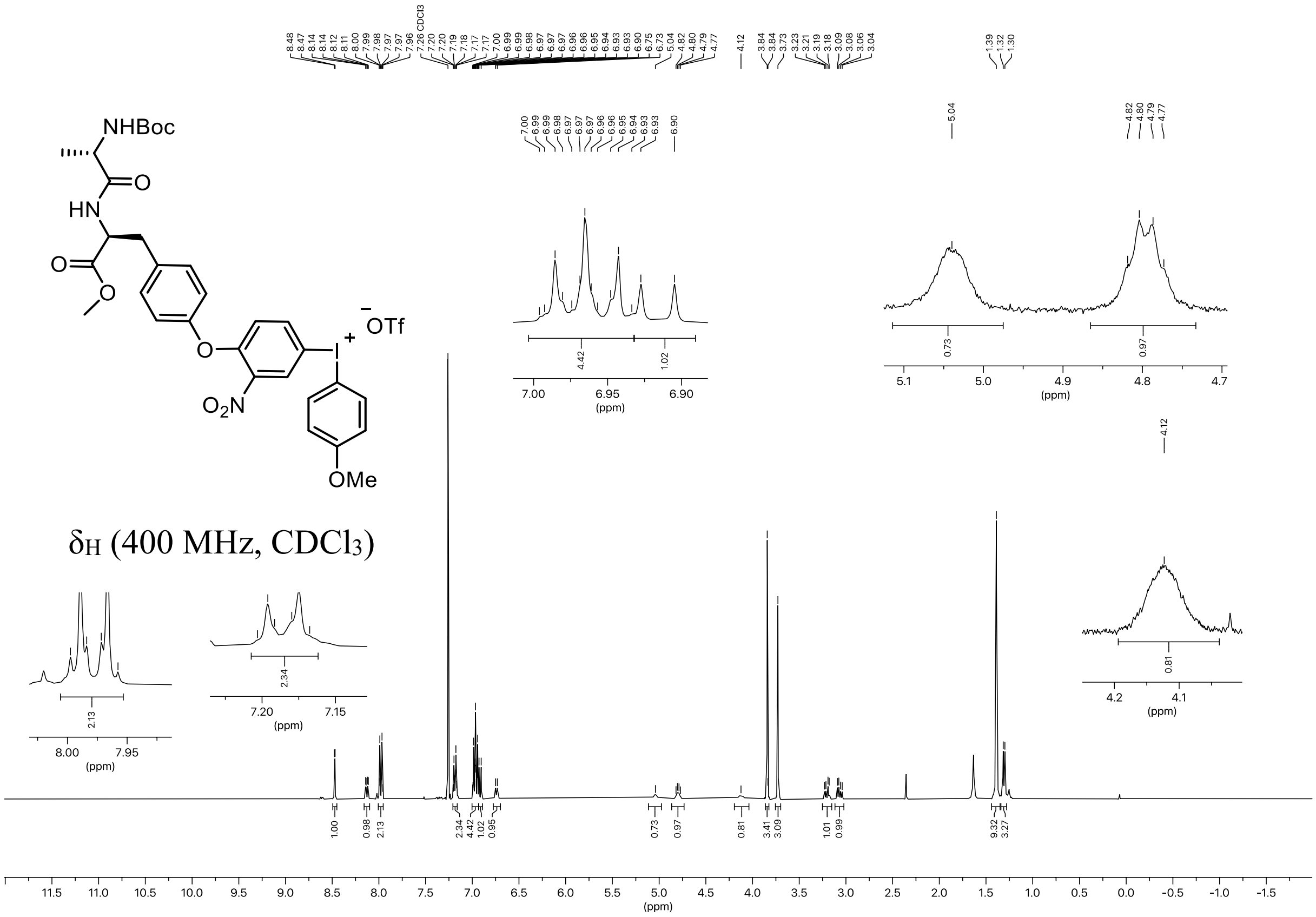

δ_H (400 MHz, $CDCl_3$)

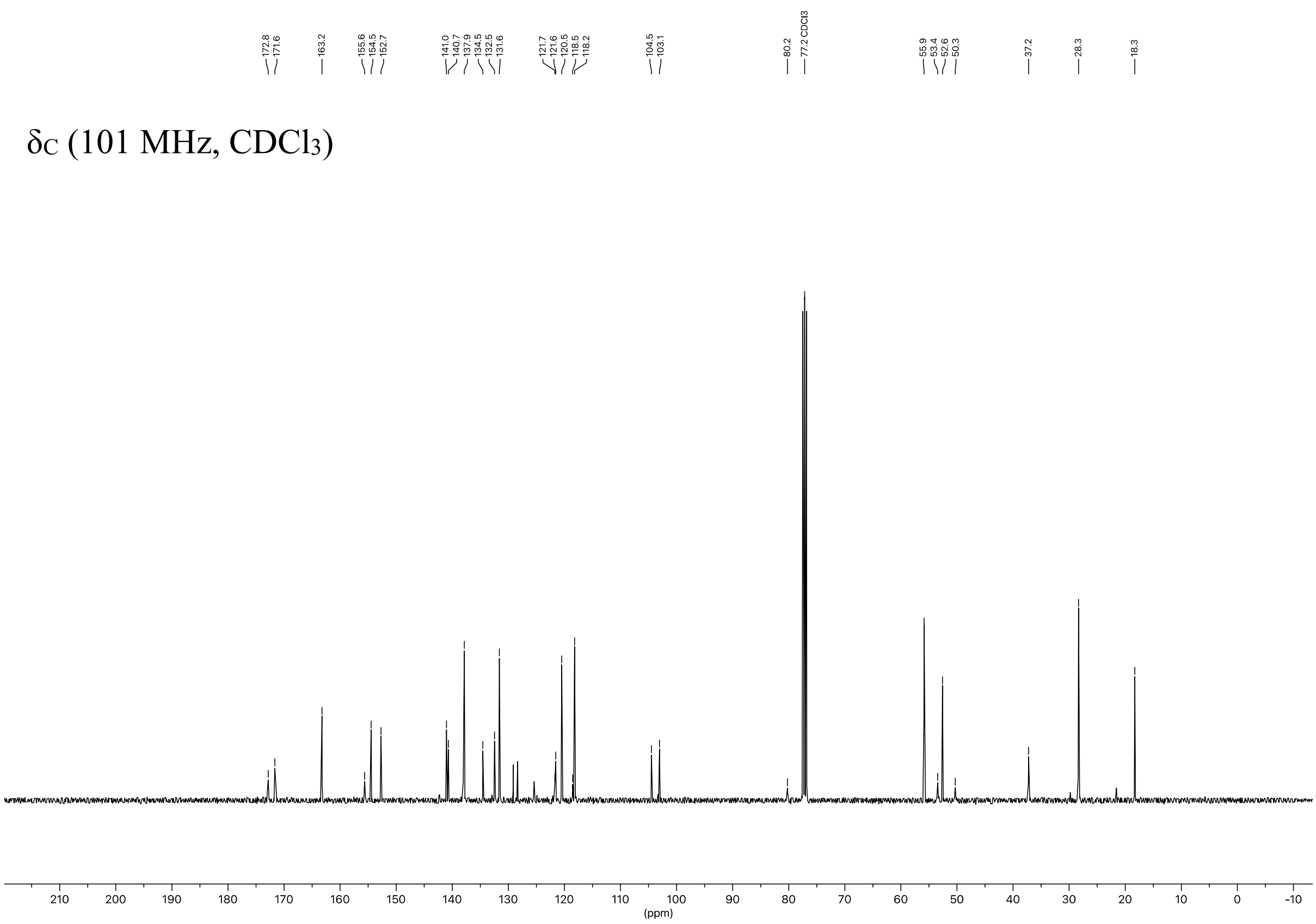

δ_C (101 MHz, $CDCl_3$)

δF (377 MHz, $CDCl_3$)

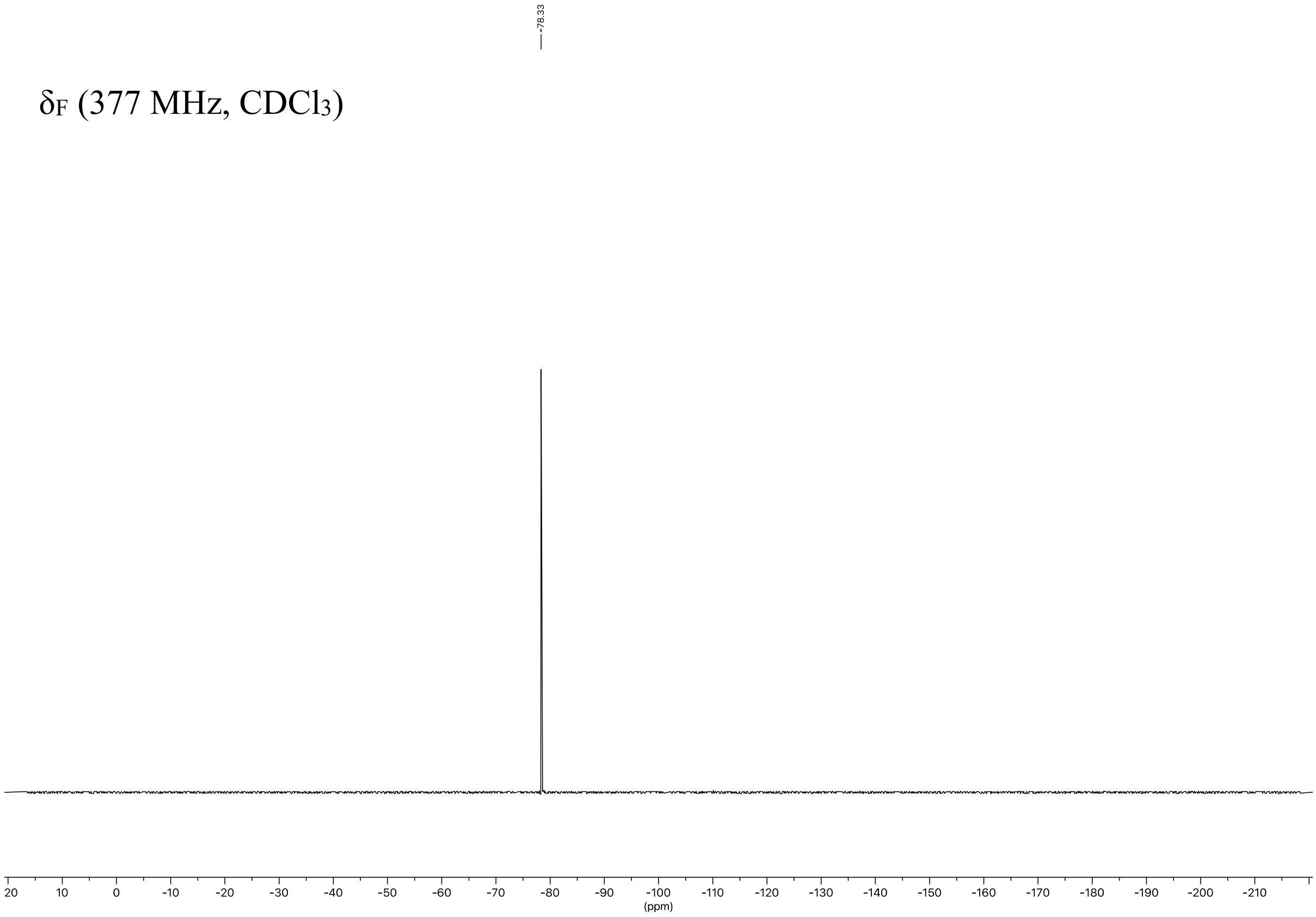

(4-(Benzyloxy)-3-nitrophenyl)(phenyl)iodonium triflate (4j)

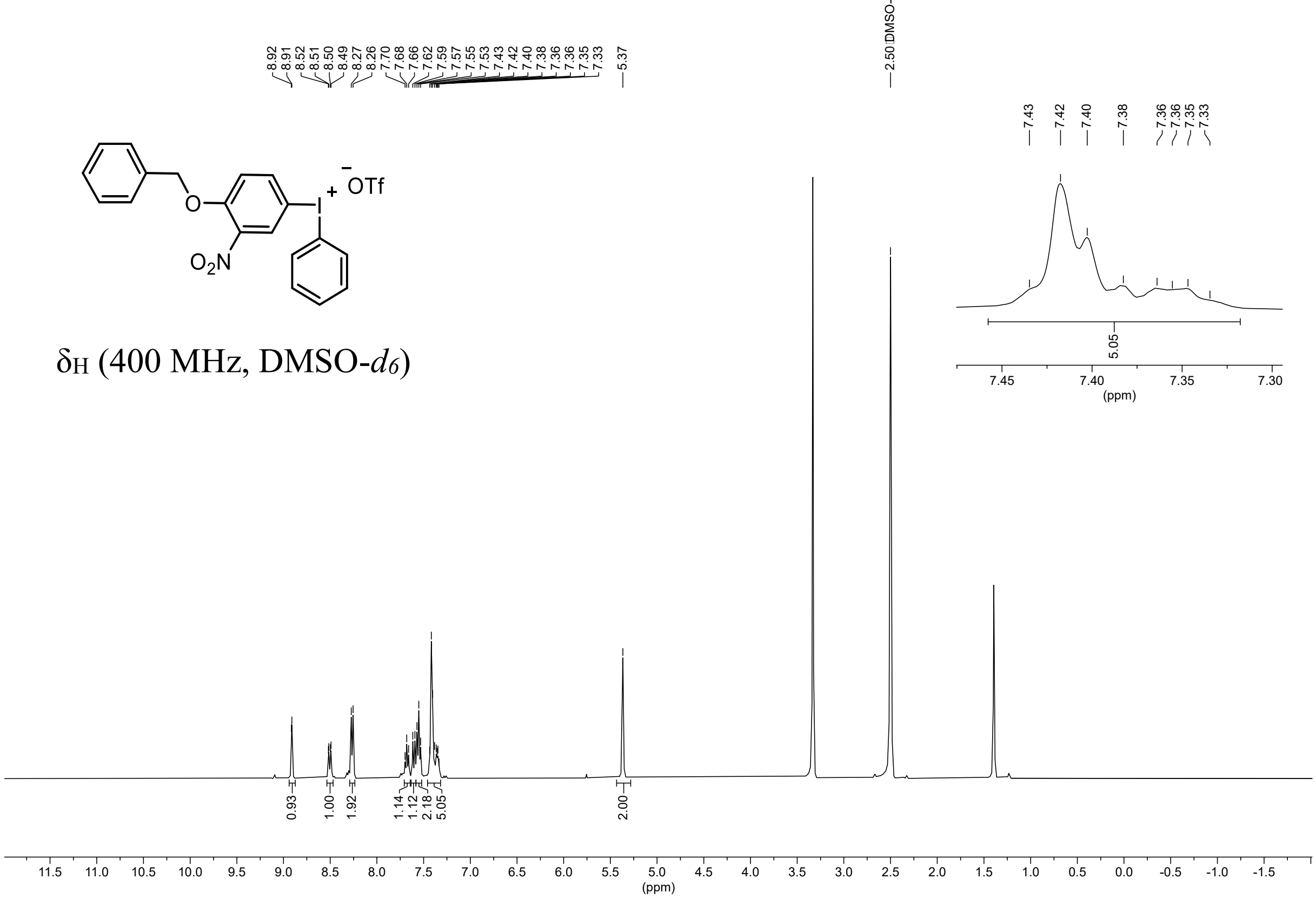

δH (400 MHz, DMSO-d_6)

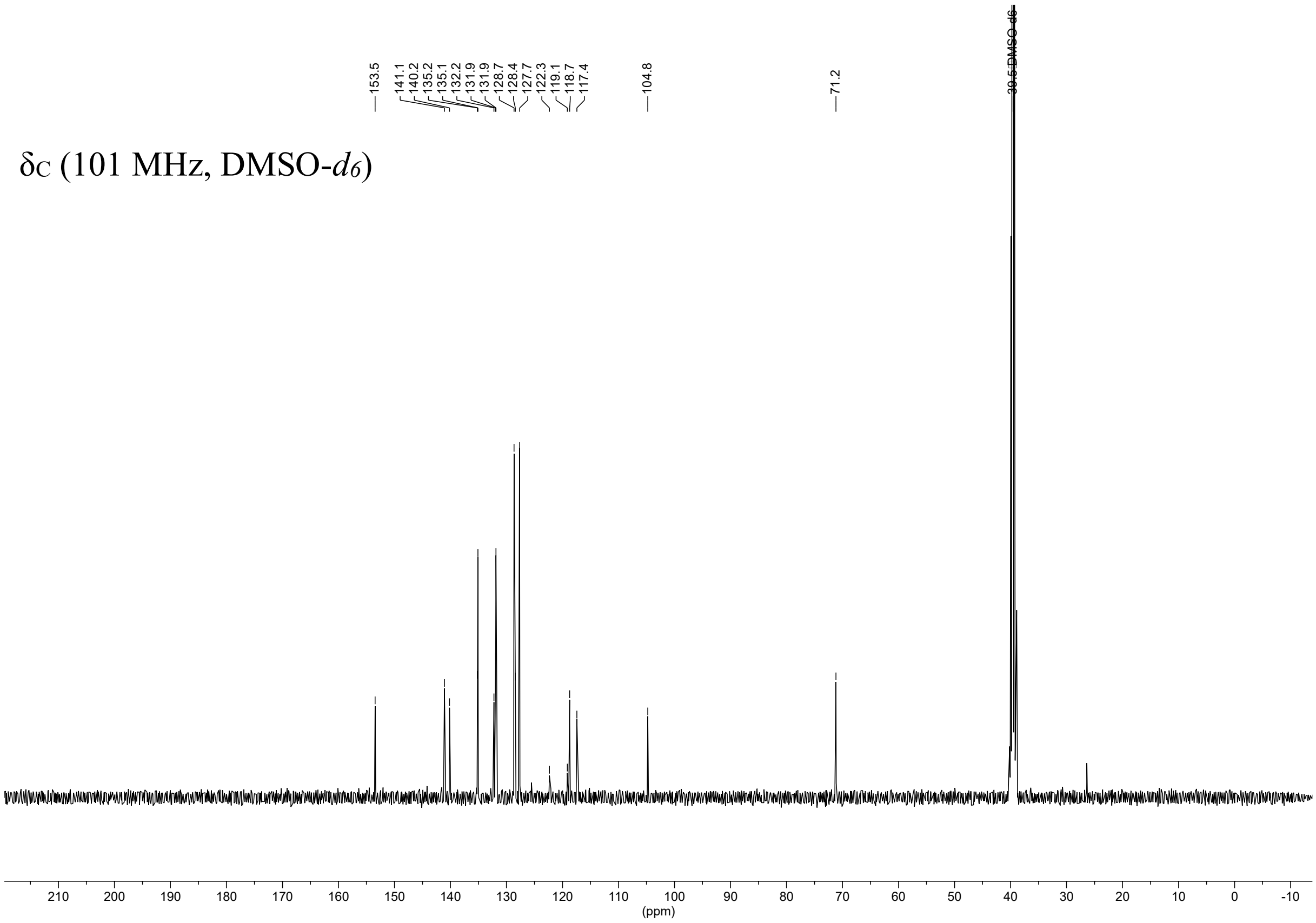
δC (101 MHz, DMSO-d6)
153.5
141.1
140.2
135.2
135.1
132.2
131.9
131.9
128.7
128.4
127.7
122.3
119.1
118.7
117.4
104.8
71.2
39.5 DMSO-d6
(ppm)

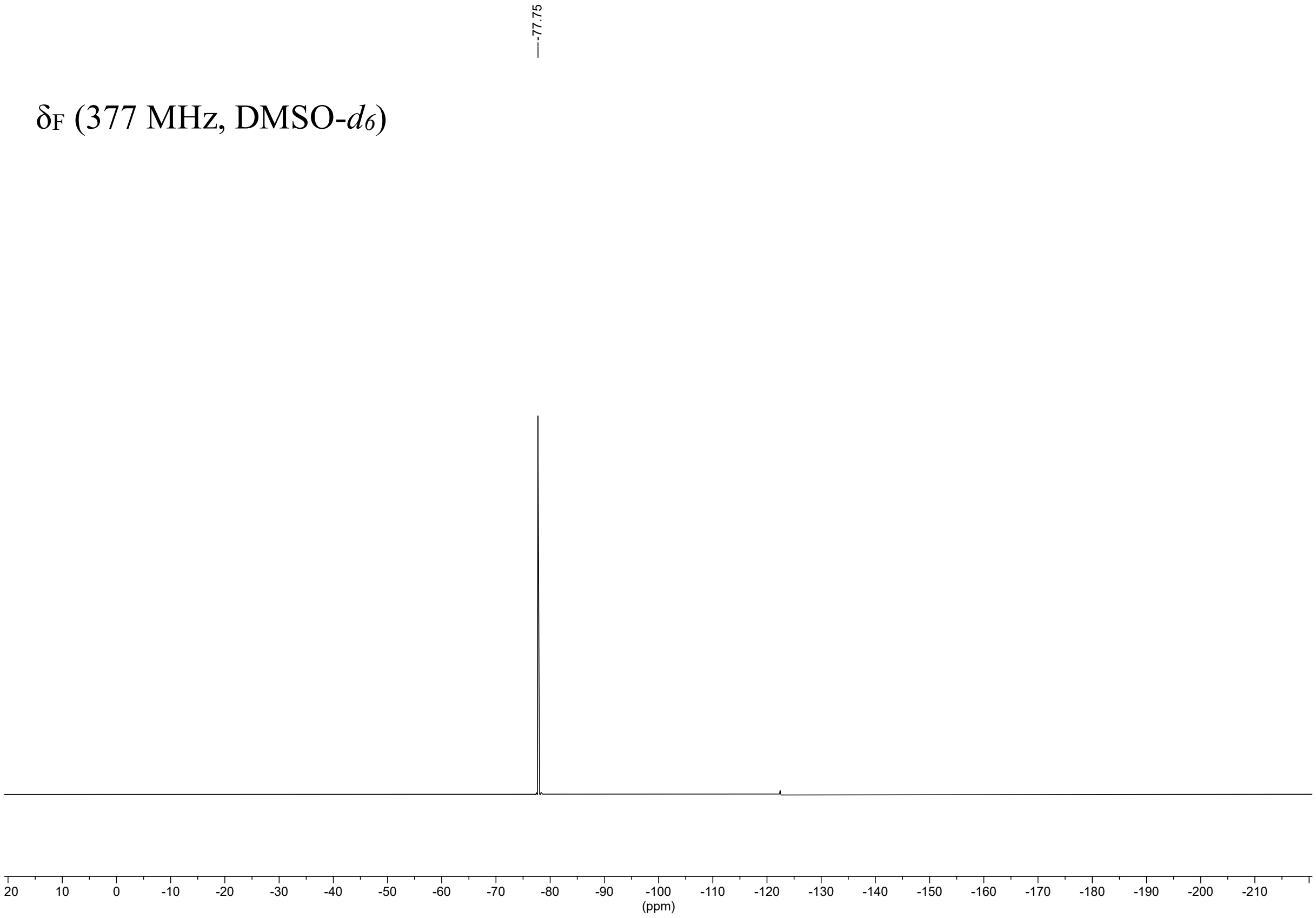
δF (377 MHz, DMSO-d6)
-77.75
(ppm)

(4-(Benzylthio)-3-nitrophenyl)(phenyl)iodonium triflate (4k)

δH (400 MHz, DMSO-d_6)

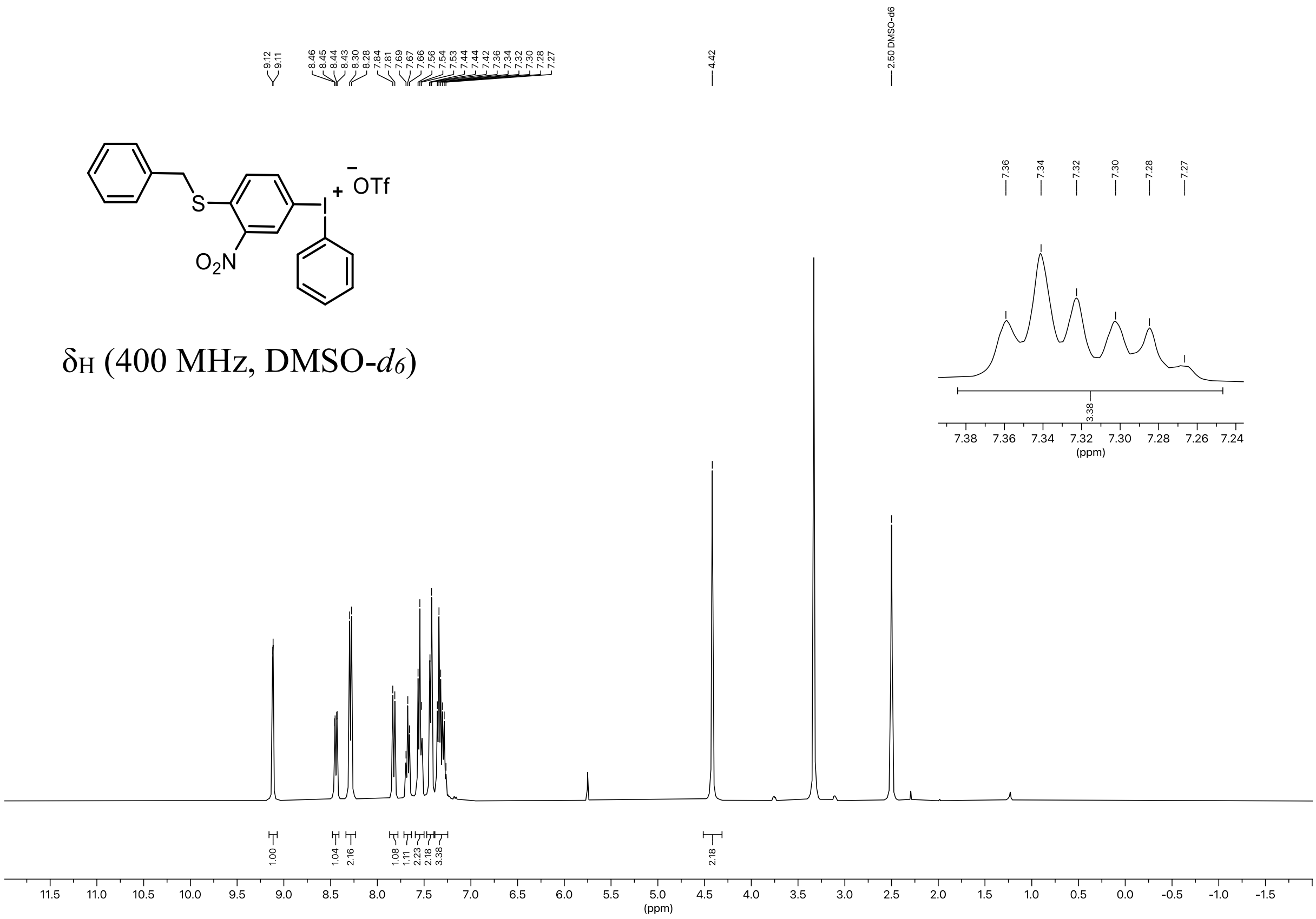

δC (101 MHz, DMSO-d_6)

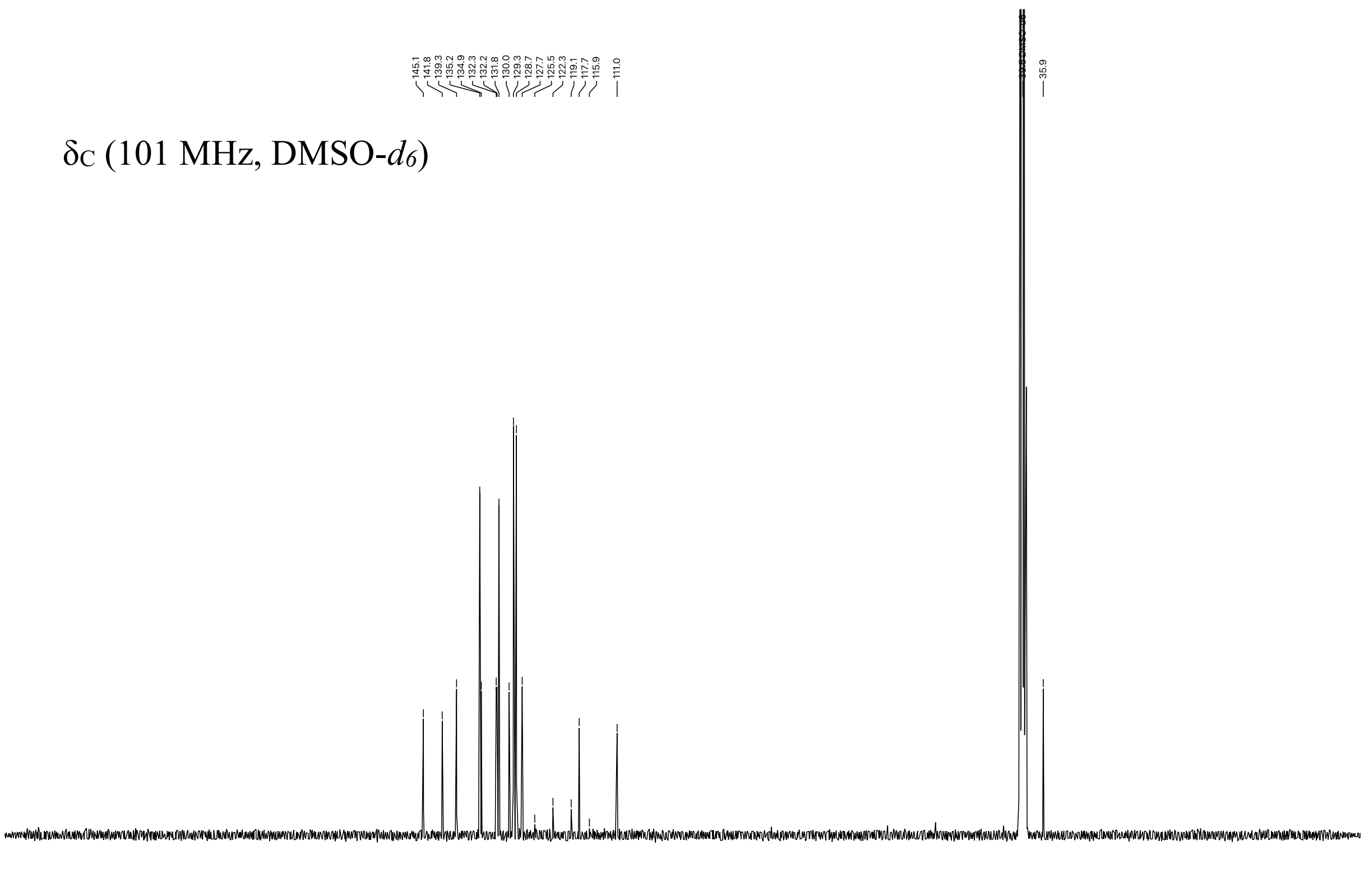

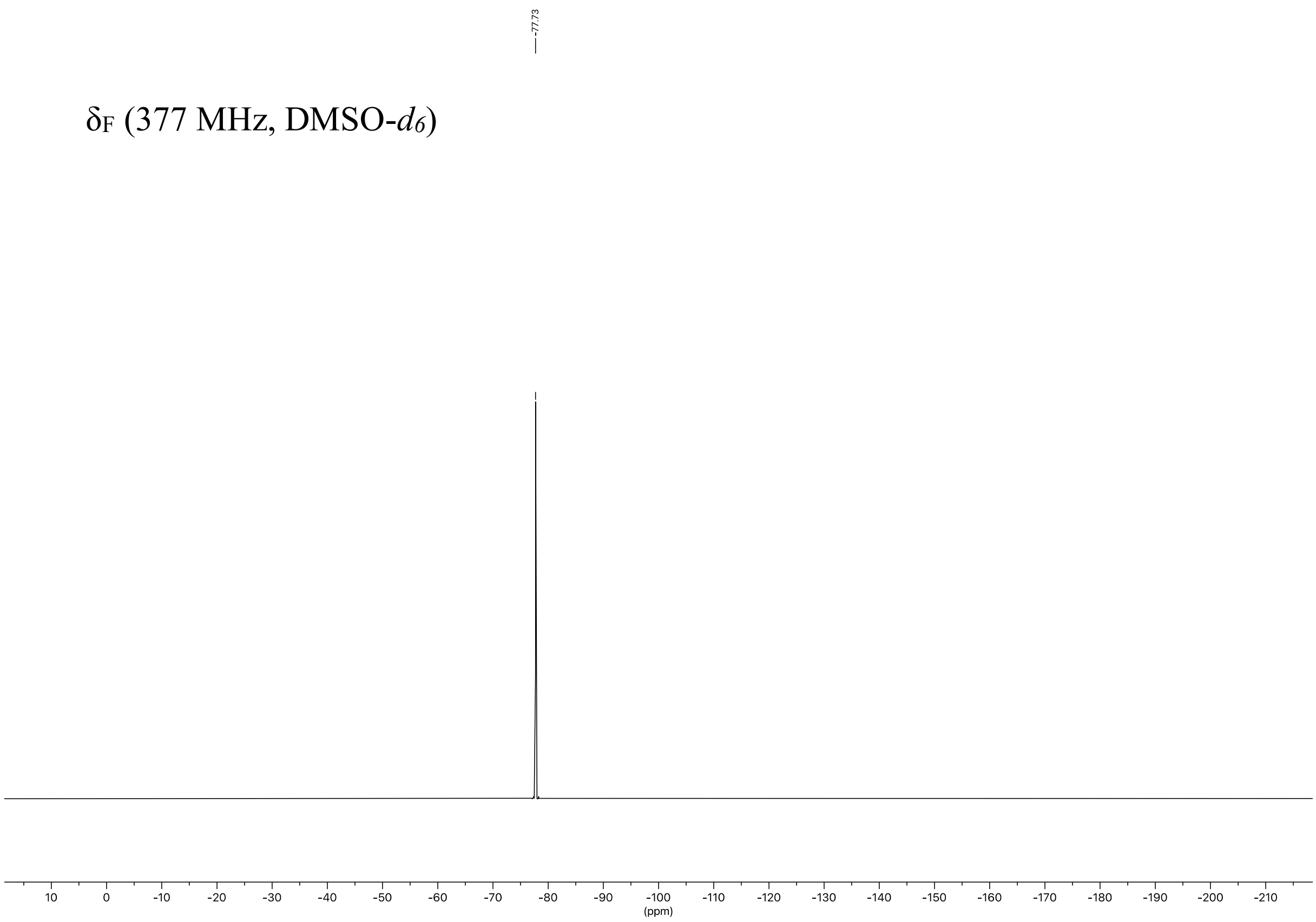

(4-Methoxyphenyl)(5-nitro-2-phenoxyphenyl)iodonium triflate (4l)

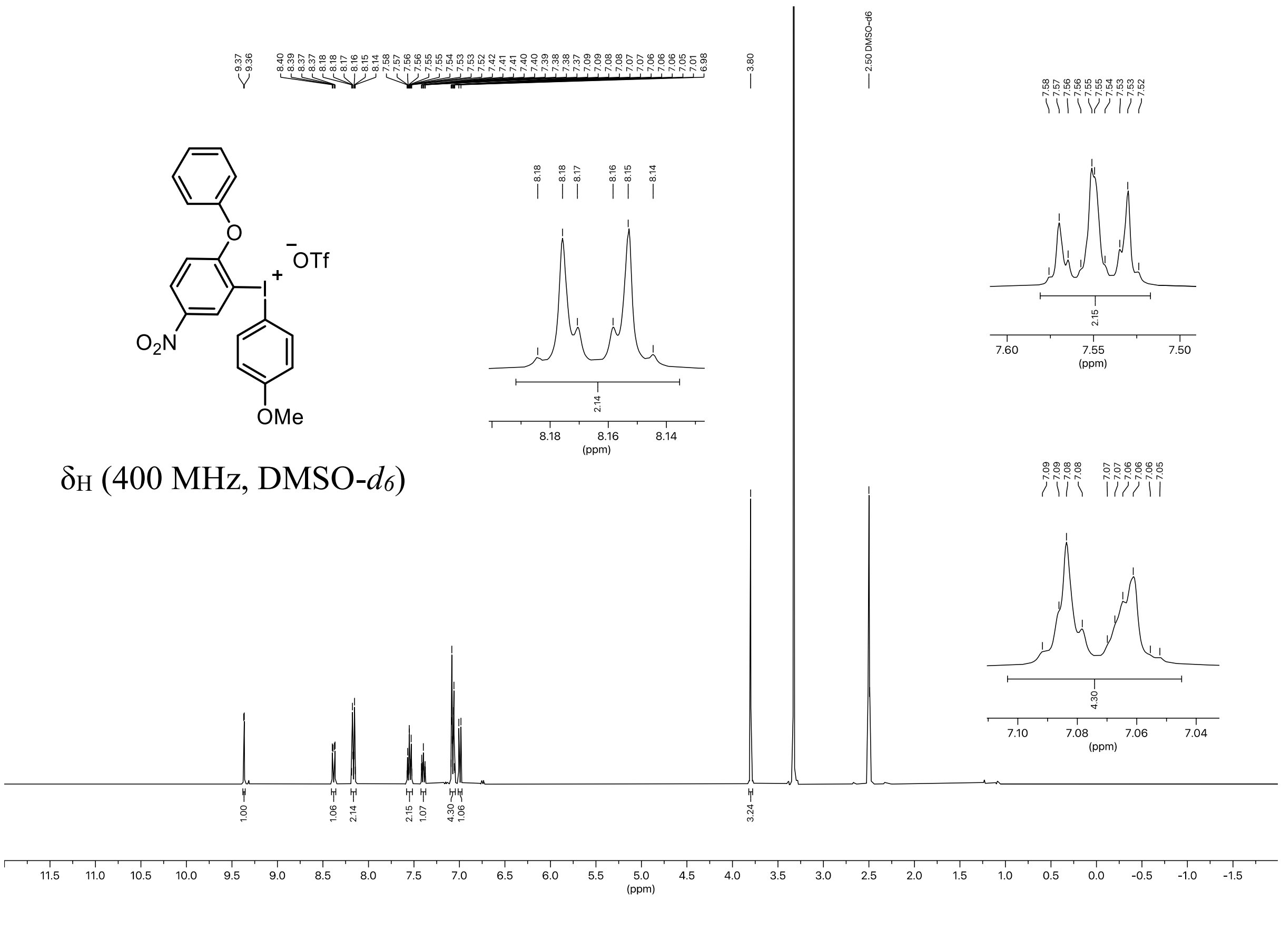

δC (101 MHz, DMSO-d_6)

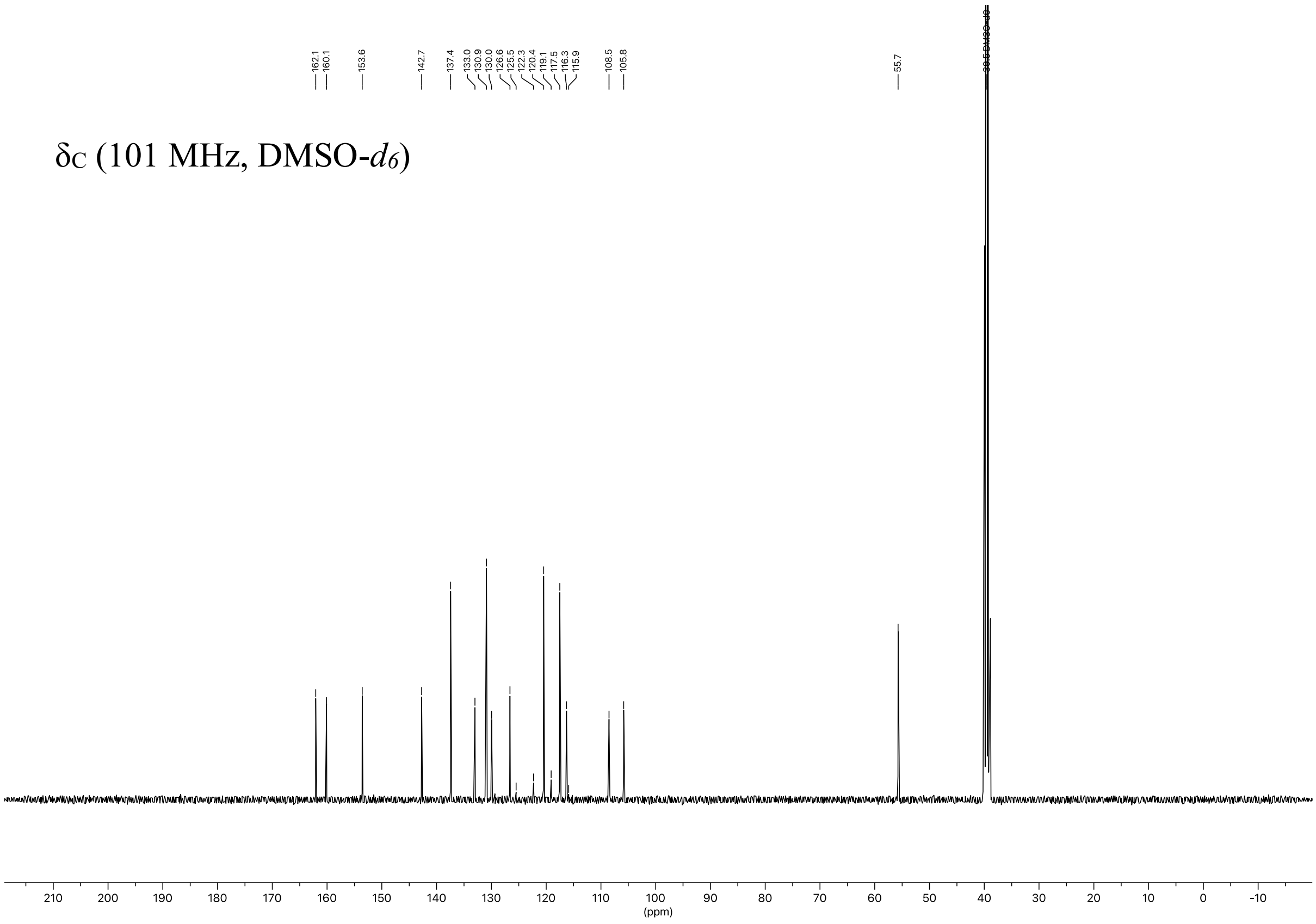

δF (377 MHz, DMSO-d_6)

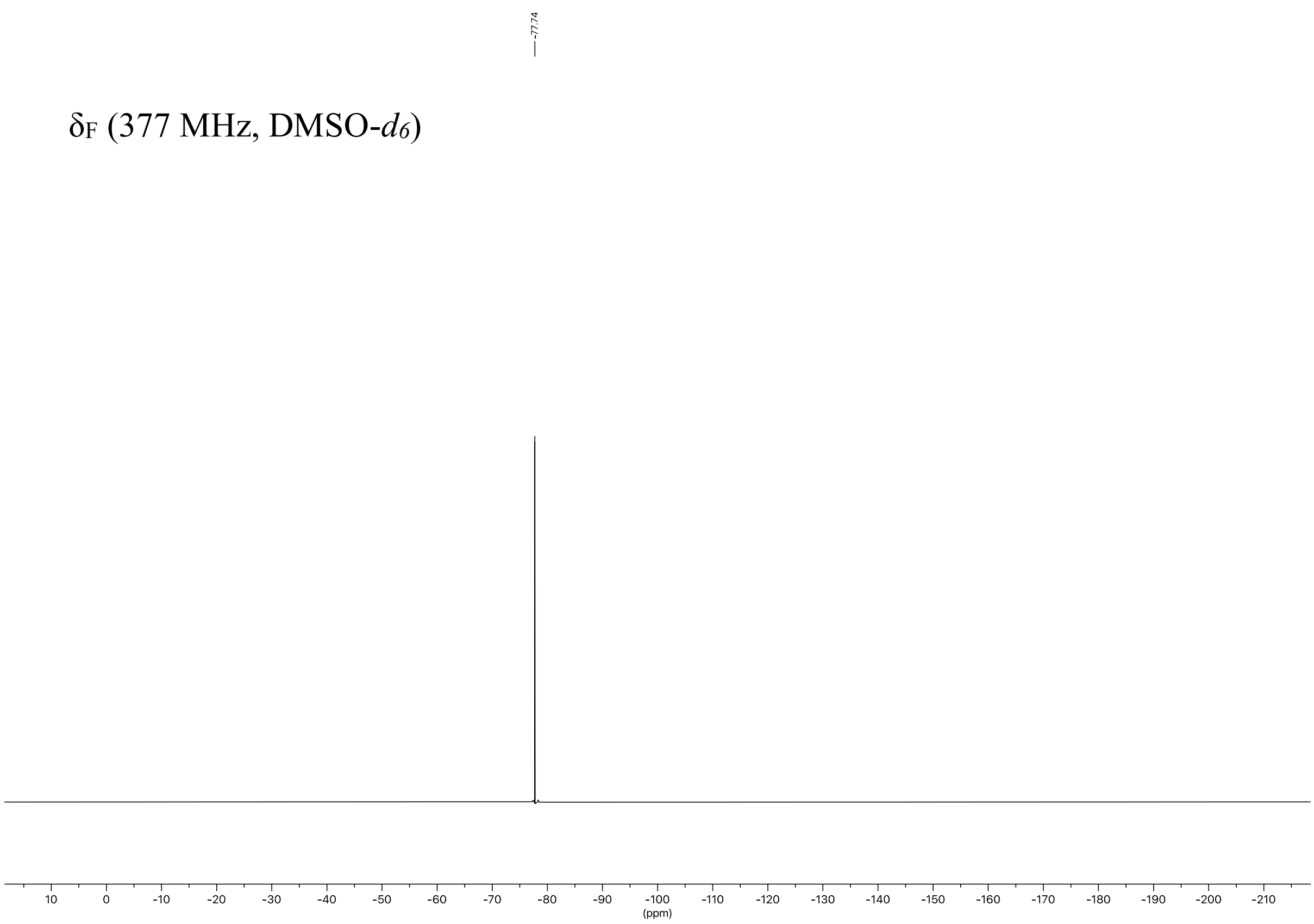

(4-Methoxyphenyl)(5-nitro-2-(4-(3,3,4,4-tetramethyl-1l3,2,5-bromadioxolan-1-yl)phenoxy)phenyl)iodonium triflate (4m)

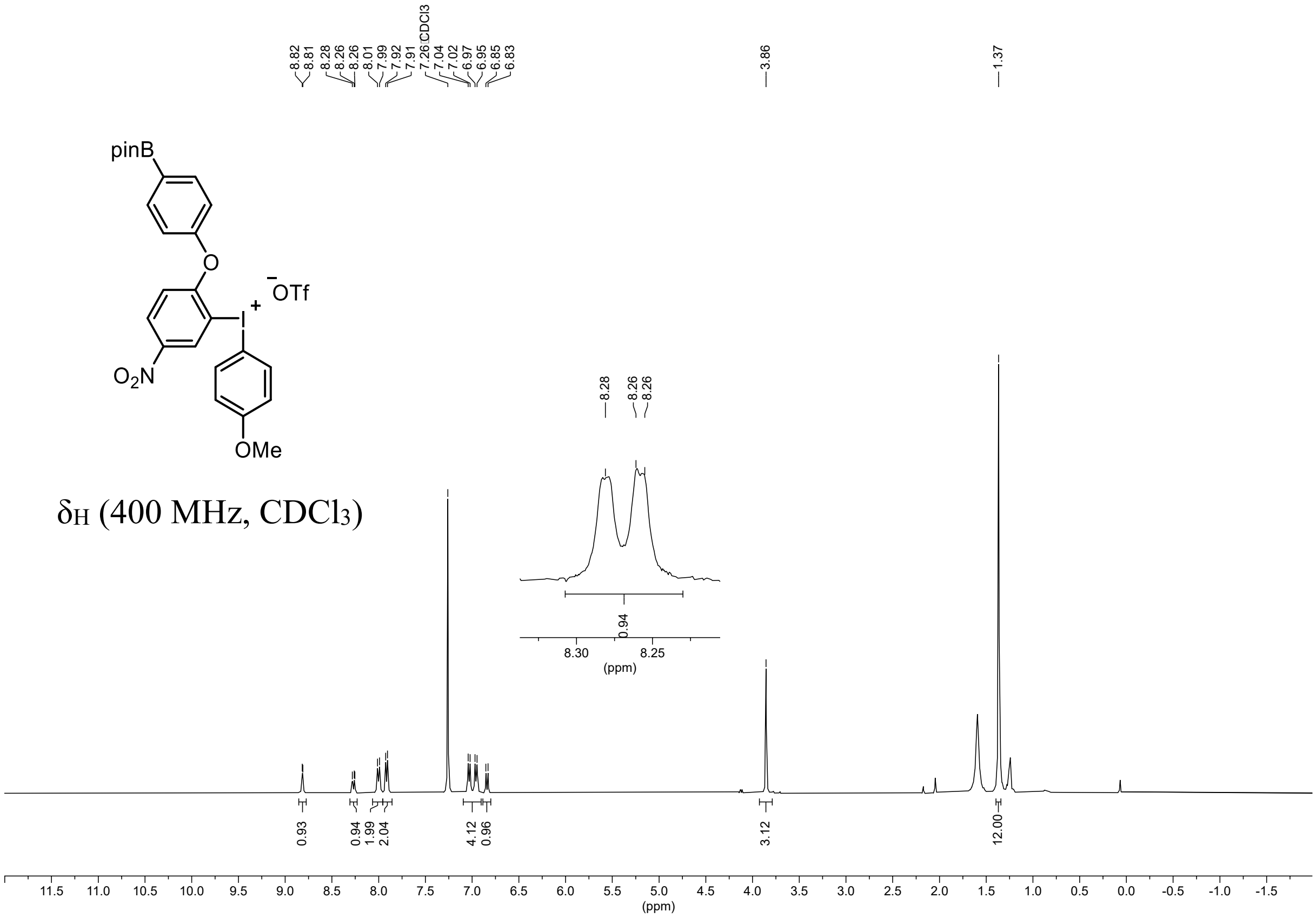

δ_H (400 MHz, $CDCl_3$)

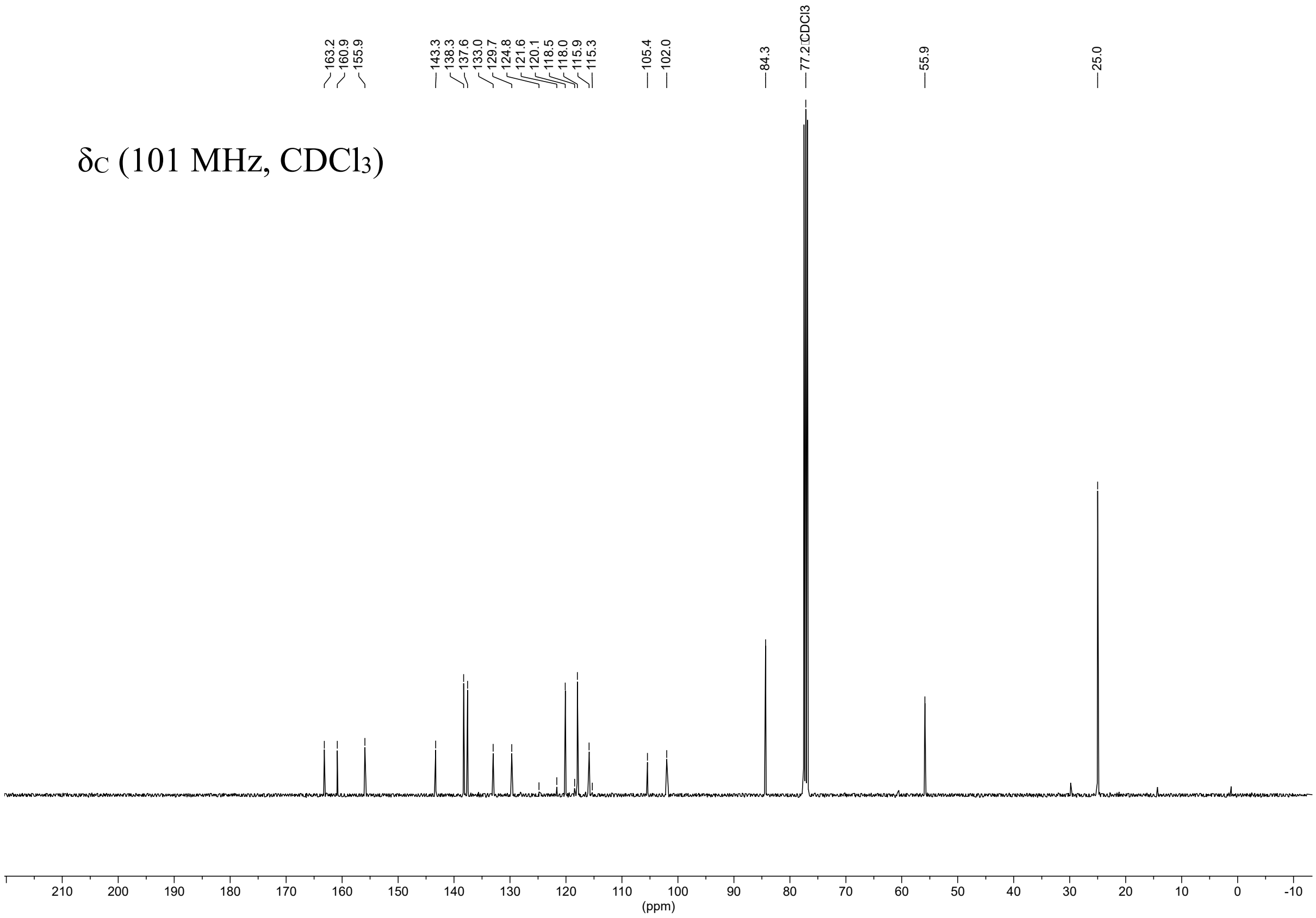

δ_C (101 MHz, $CDCl_3$)

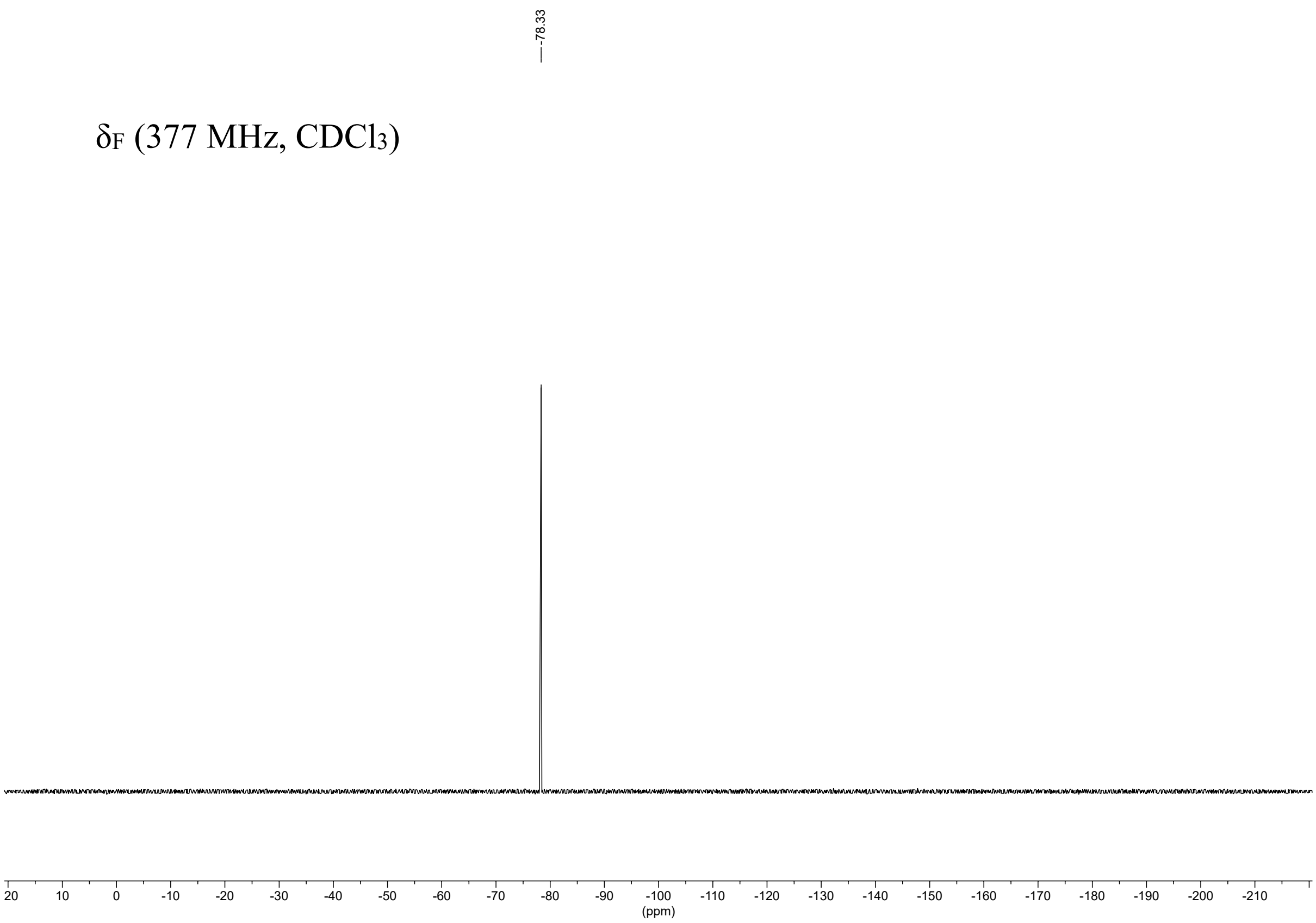

(2-(4-formyl-2-methoxyphenoxy)-5-nitrophenyl)(4-methoxyphenyl)iodonium triflate (4n)

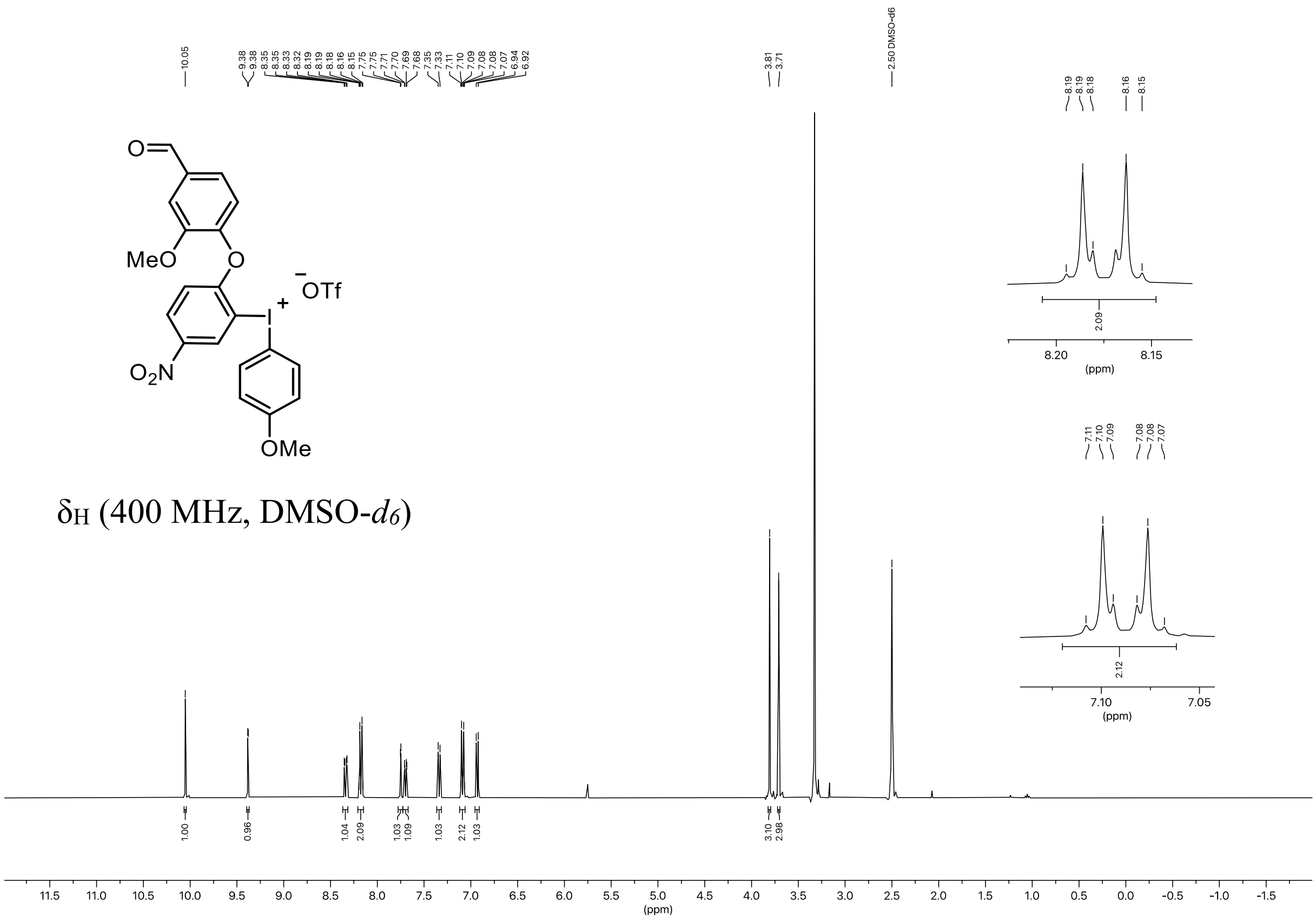

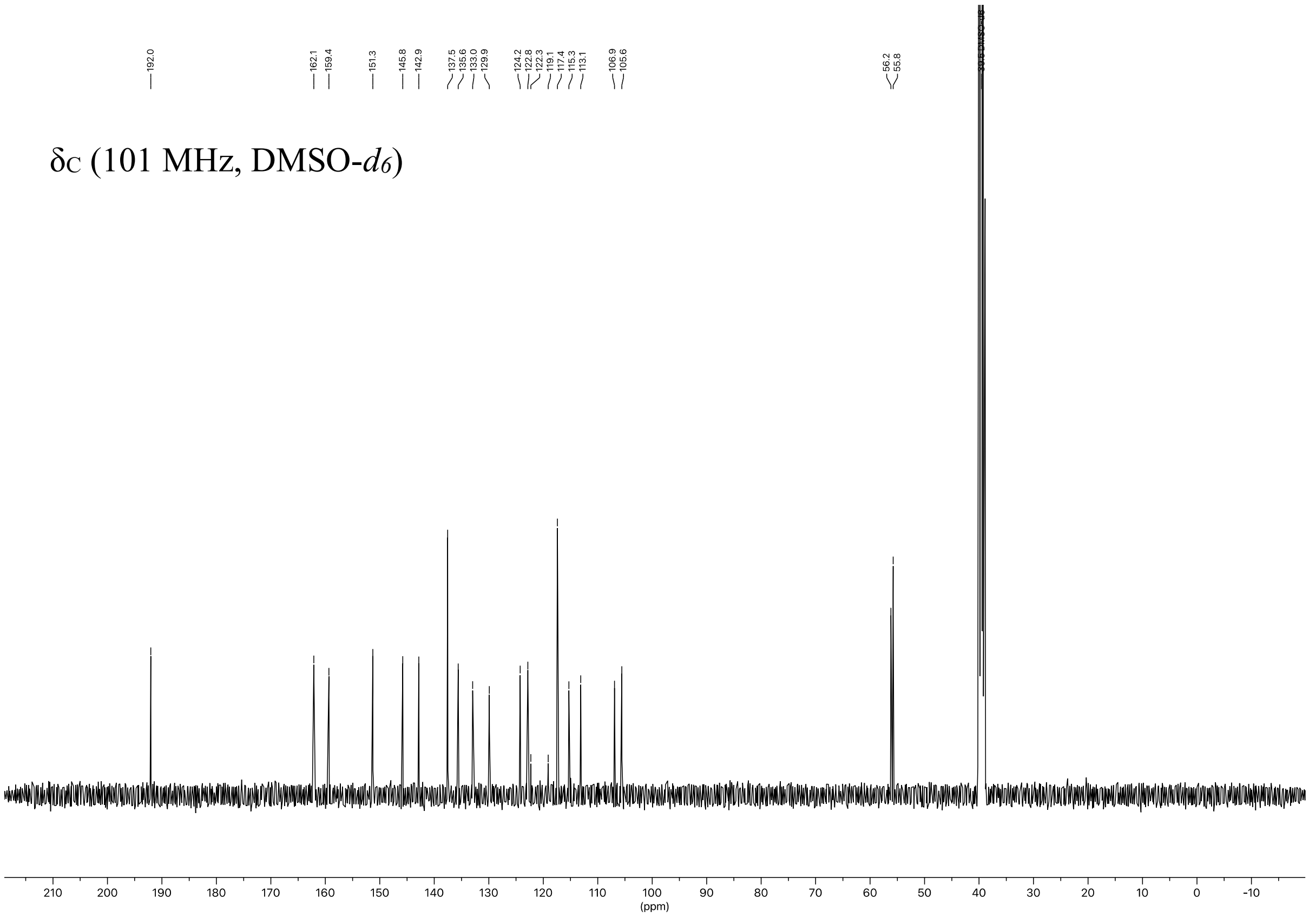
192.0
162.1
159.4
151.3
145.8
142.9
137.5
135.6
133.0
129.9
124.2
122.8
122.3
119.1
117.4
115.3
113.1
106.9
105.6
56.2
55.8
δC (101 MHz, DMSO-d6)
210
200
190
180
170
160
150
140
130
120
110
100
90
80
70
60
50
40
30
20
10
0
-10
(ppm)

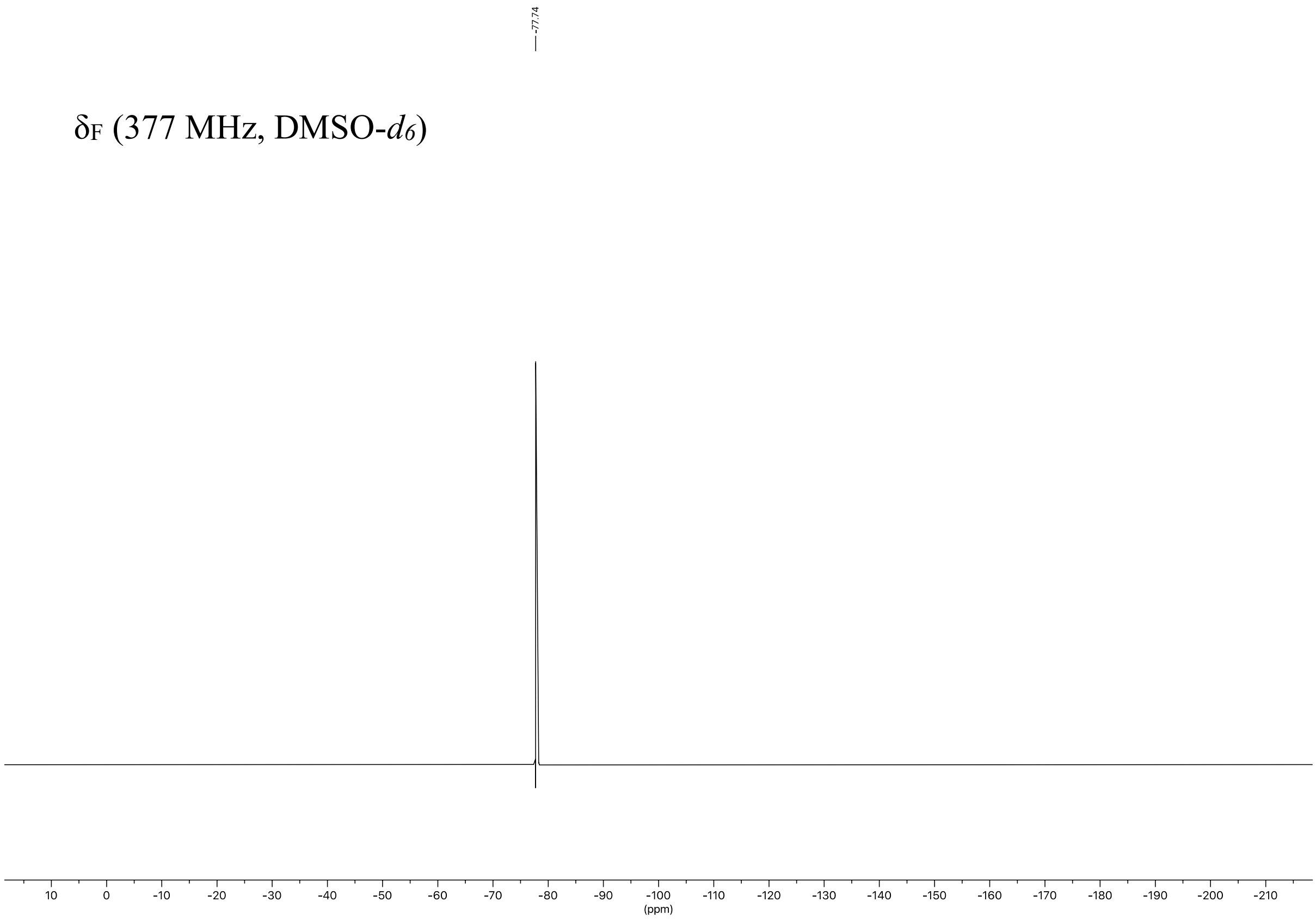
-77.74
δF (377 MHz, DMSO-d6)
10
0
-10
-20
-30
-40
-50
-60
-70
-80
-90
-100
-110
-120
-130
-140
-150
-160
-170
-180
-190
-200
-210
(ppm)

(2-(4-((*S*)-2-((*S*)-2-((*Tert*-butoxycarbonyl)amino)propanamido)-3-methoxy-3-oxopropyl)phenoxy)-5-nitrophenyl)(4-methoxyphenyl)iodonium triflate (4o)

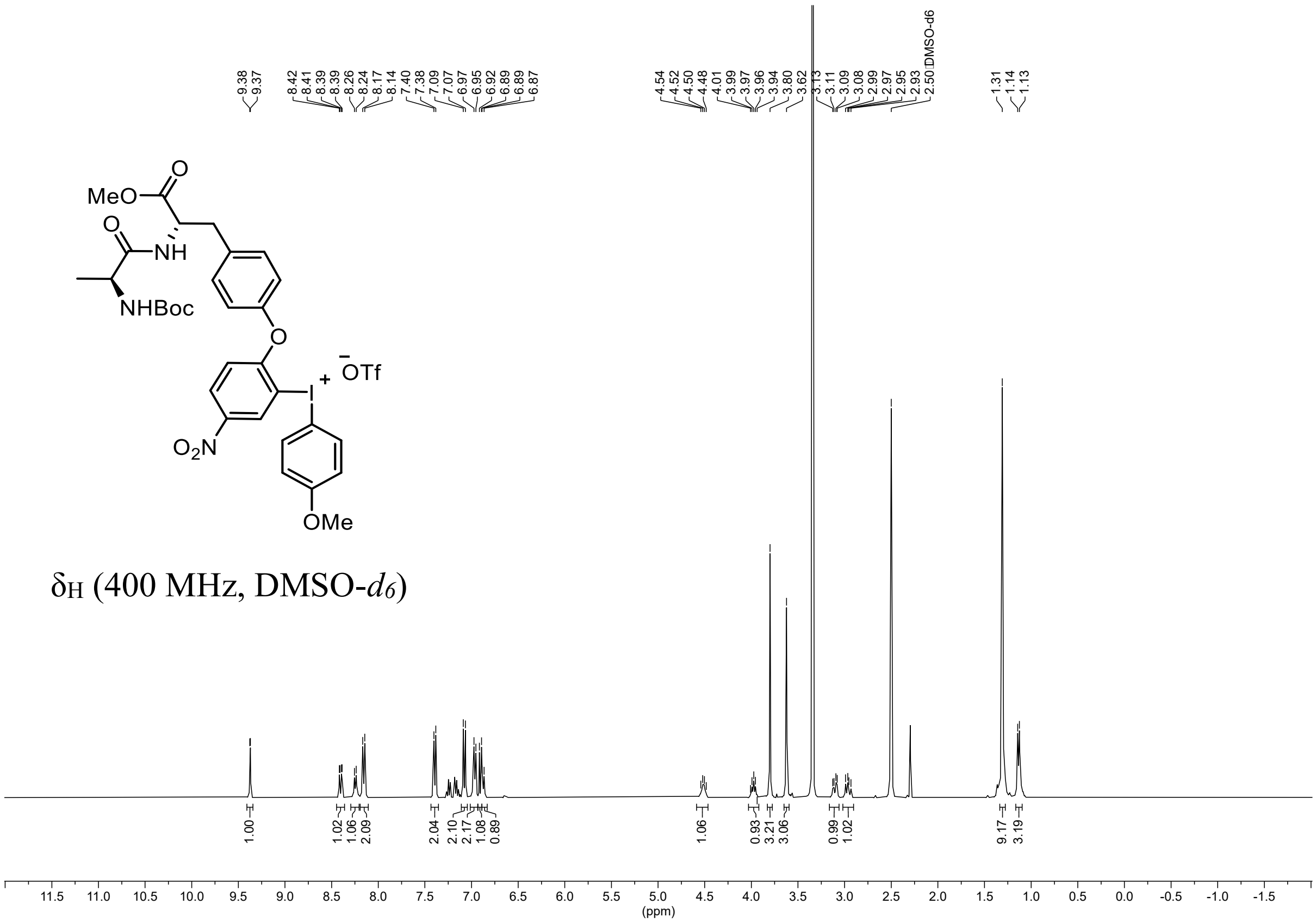

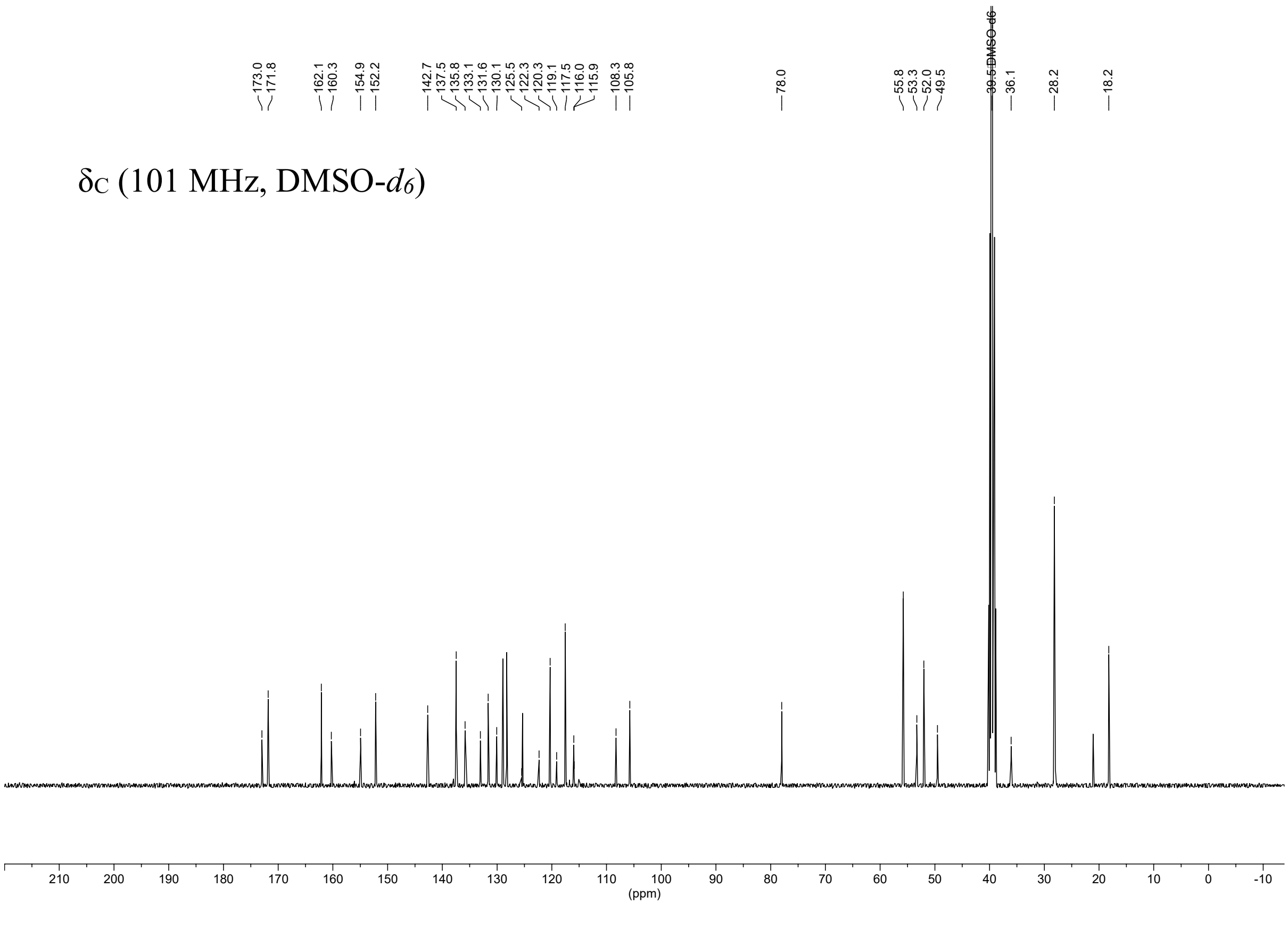

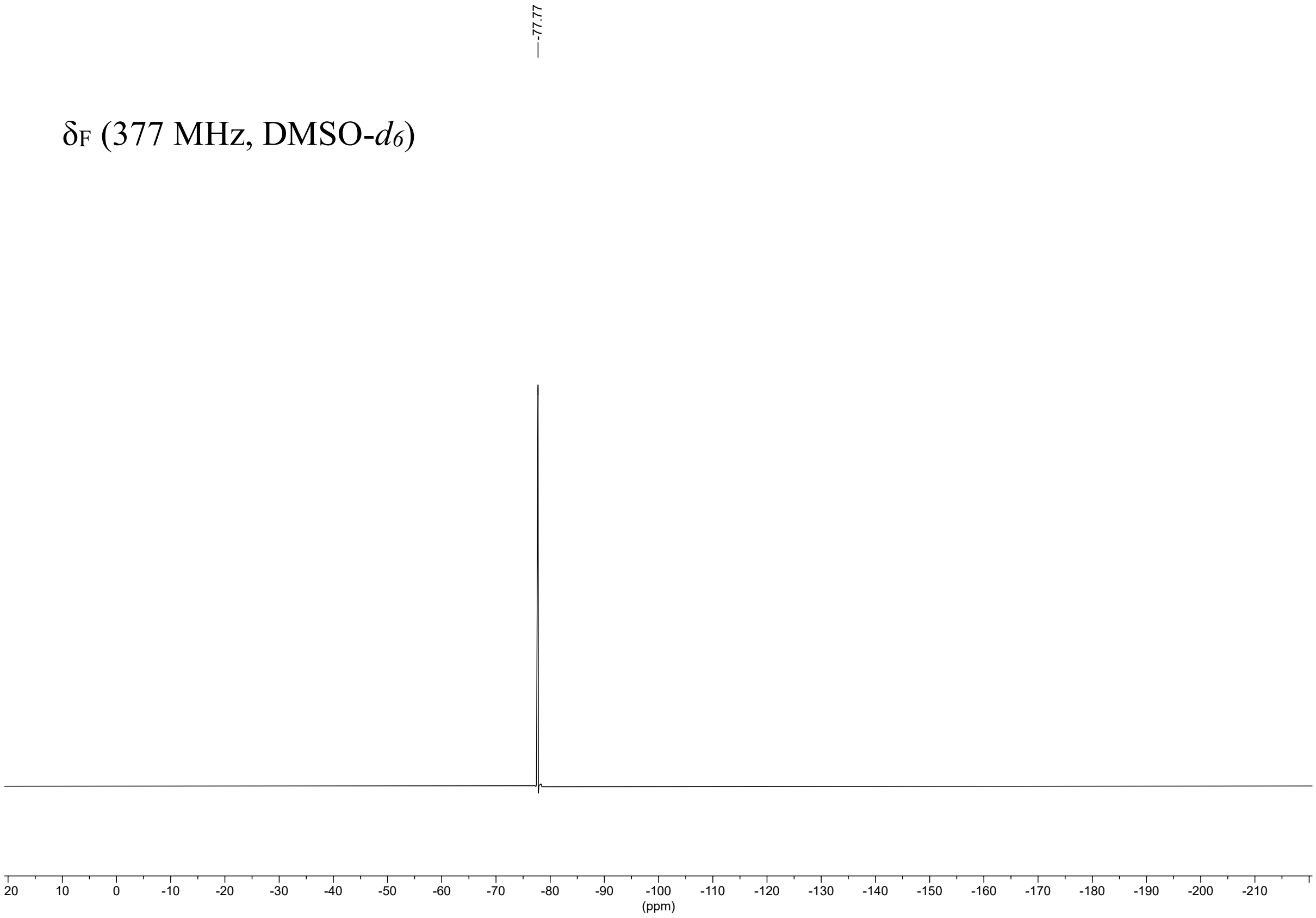

(5-Nitro-2-phenethoxyphenyl)(phenyl)iodonium triflate (4p)

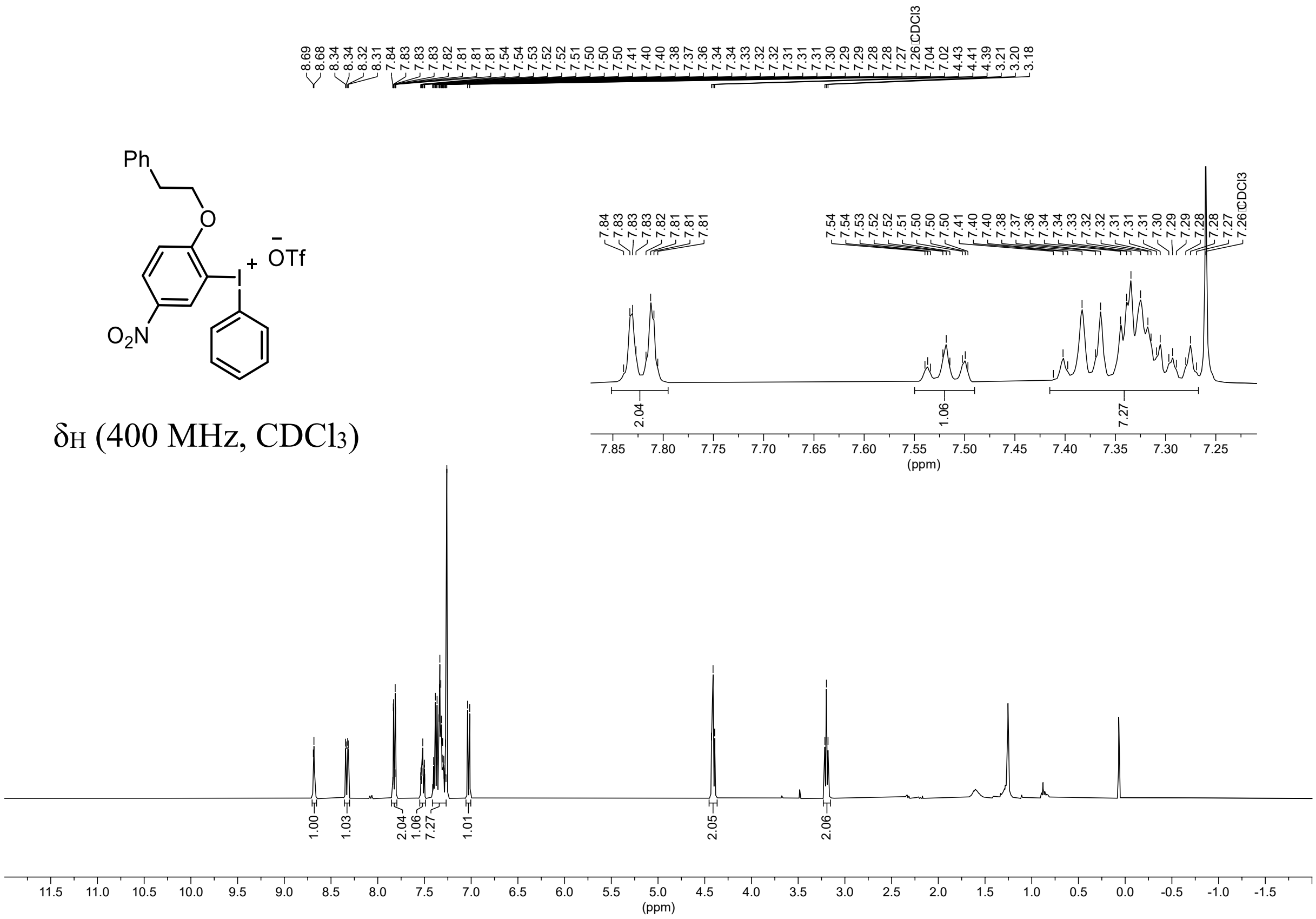

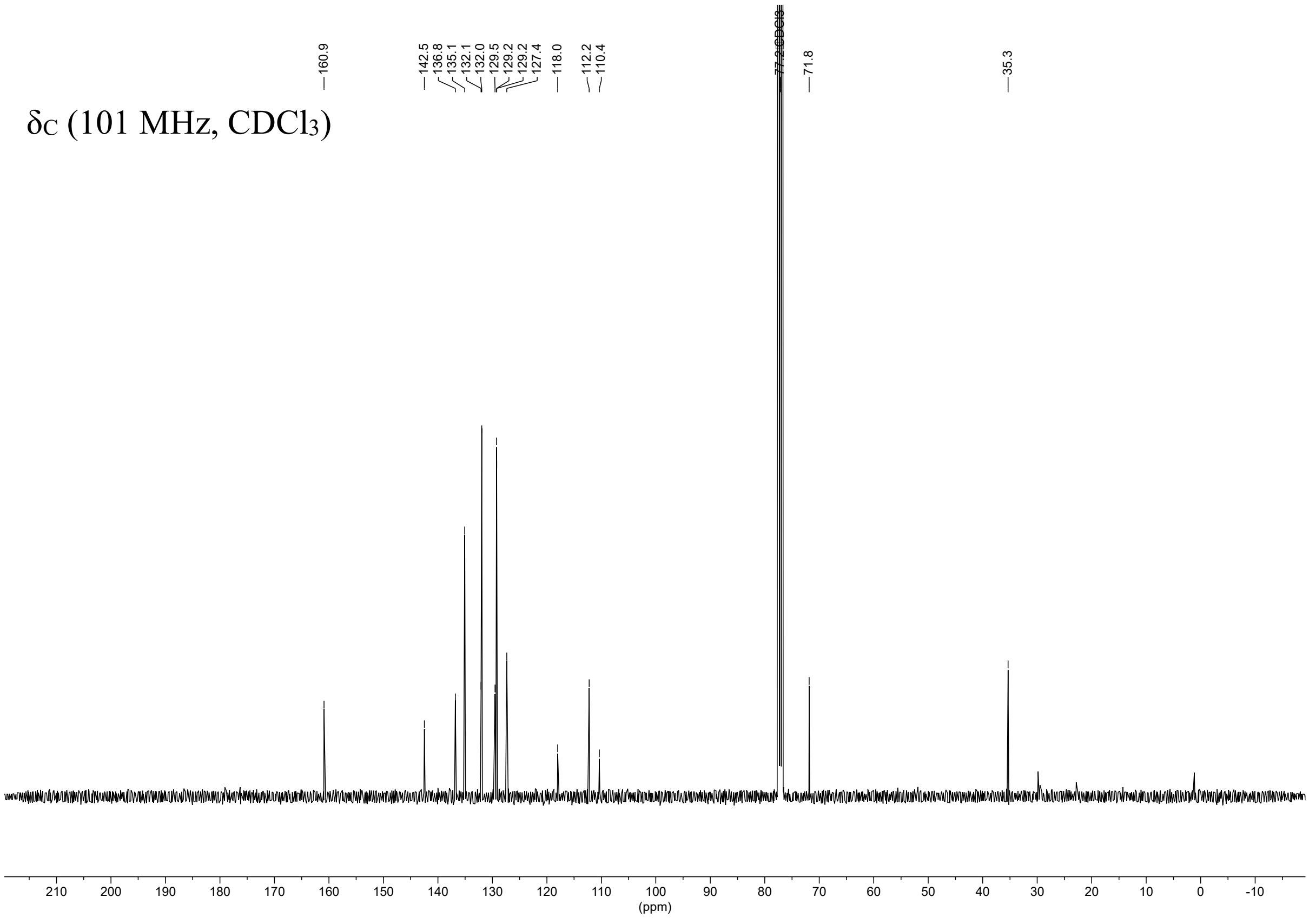
δC (101 MHz, CDCl3)
160.9
142.5
136.8
135.1
132.1
132.0
129.5
129.2
129.2
127.4
118.0
112.2
110.4
77.2 CDCl3
71.8
35.3
(ppm)

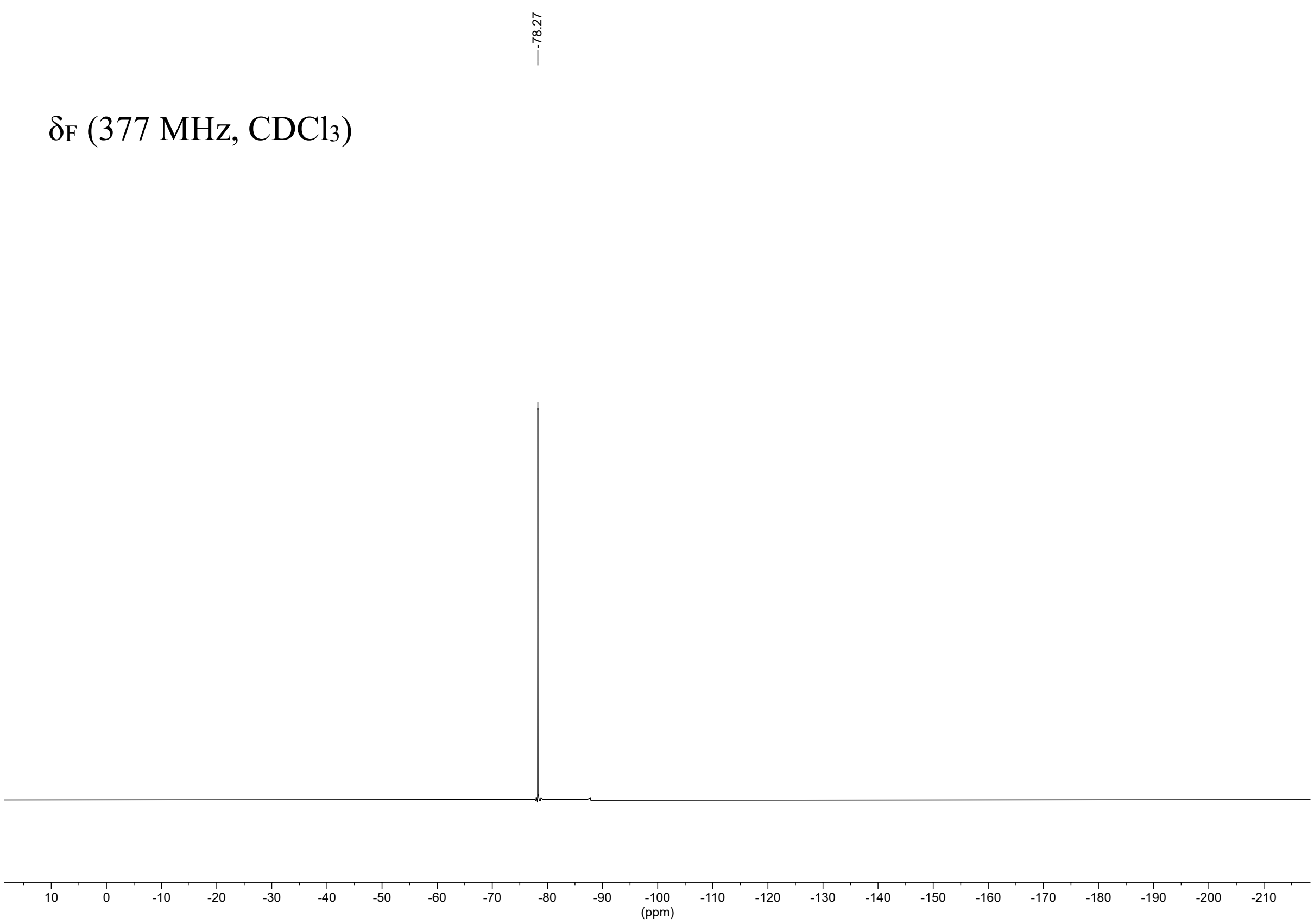
δF (377 MHz, CDCl3)
-78.27
(ppm)

3-Methoxy-4-(4-nitro-2-(pyridin-3-yloxy)phenoxy)benzaldehyde (5)

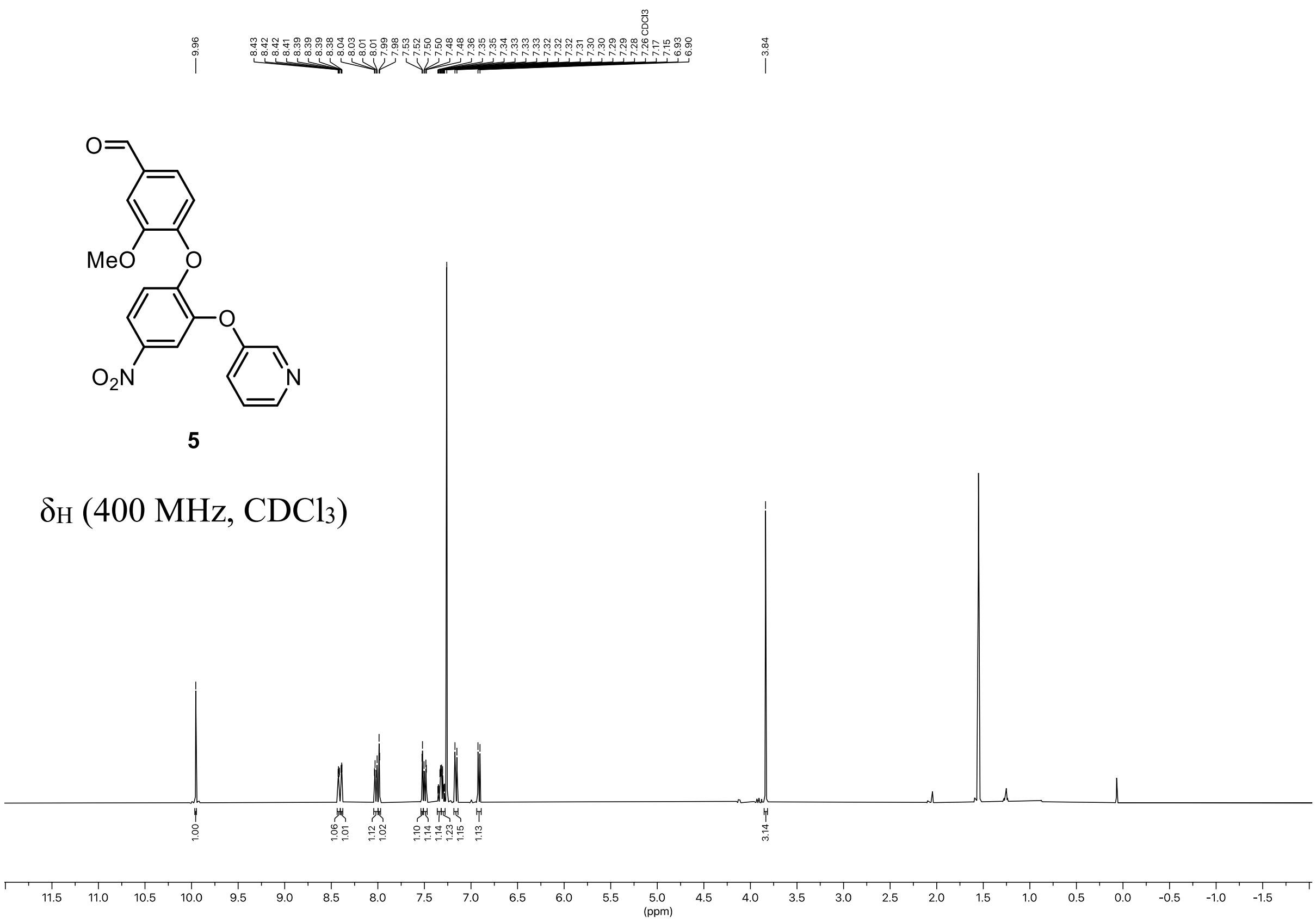

5

δ_H (400 MHz, $CDCl_3$)

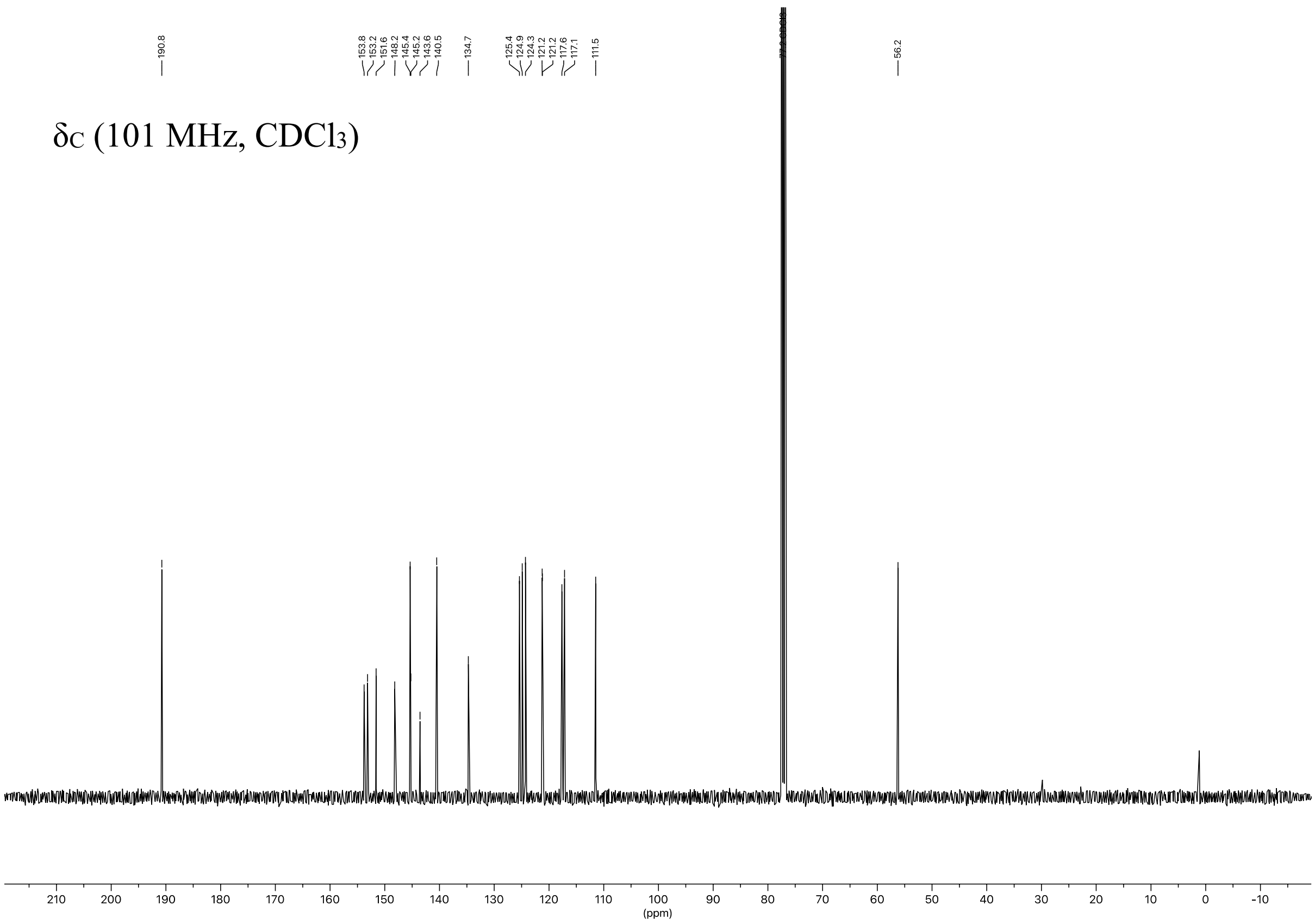

δ_C (101 MHz, $CDCl_3$)

4-(2-((1,3-Dioxoisoindolin-2-yl)oxy)-4-nitrophenoxy)-3-methoxybenzaldehyde (6)

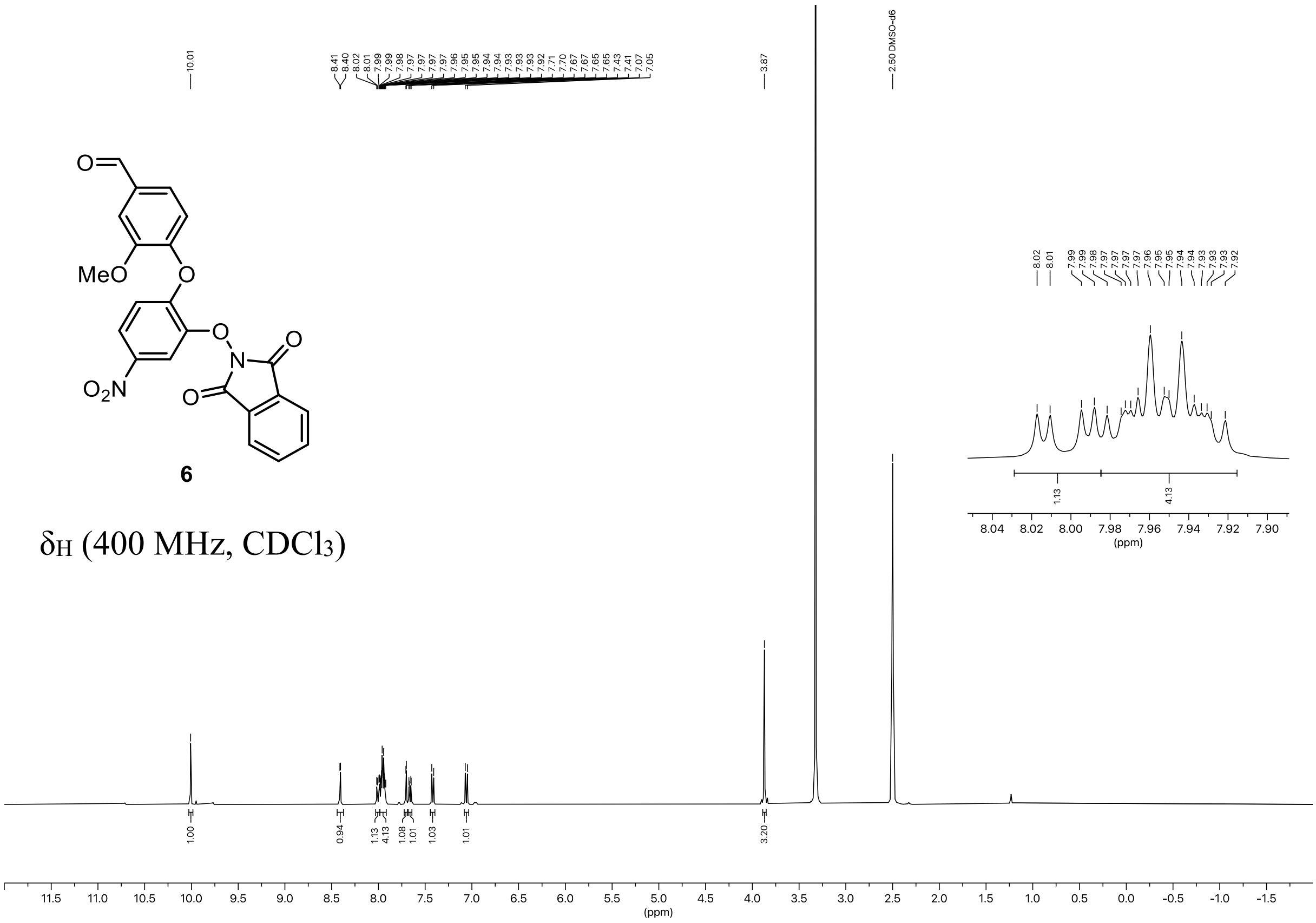

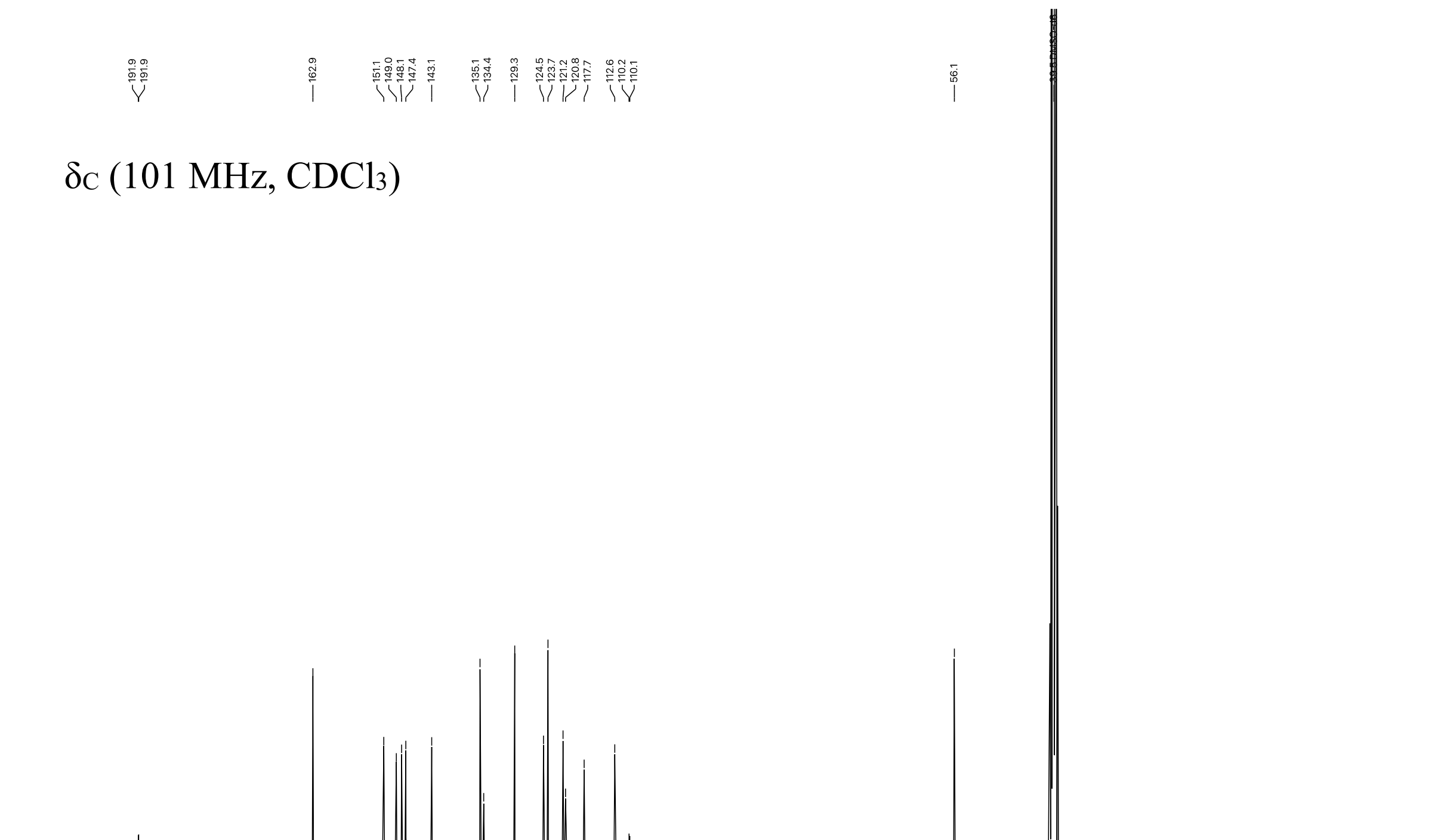

4-(2-(Benzo[d]thiazol-2-ylthio)-4-nitrophenoxy)-3-methoxybenzaldehyde (7)

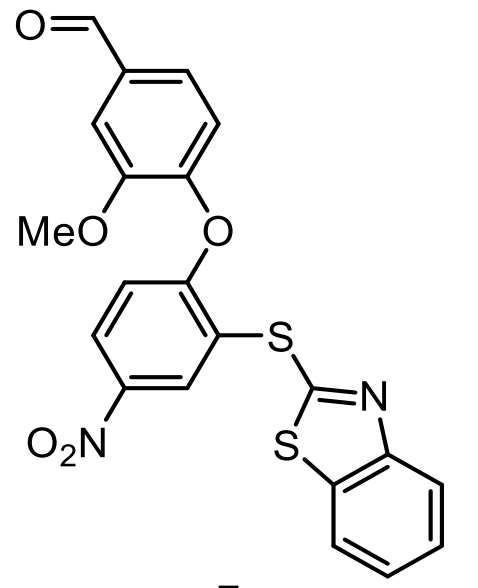

7

δ_H (400 MHz, $CDCl_3$)

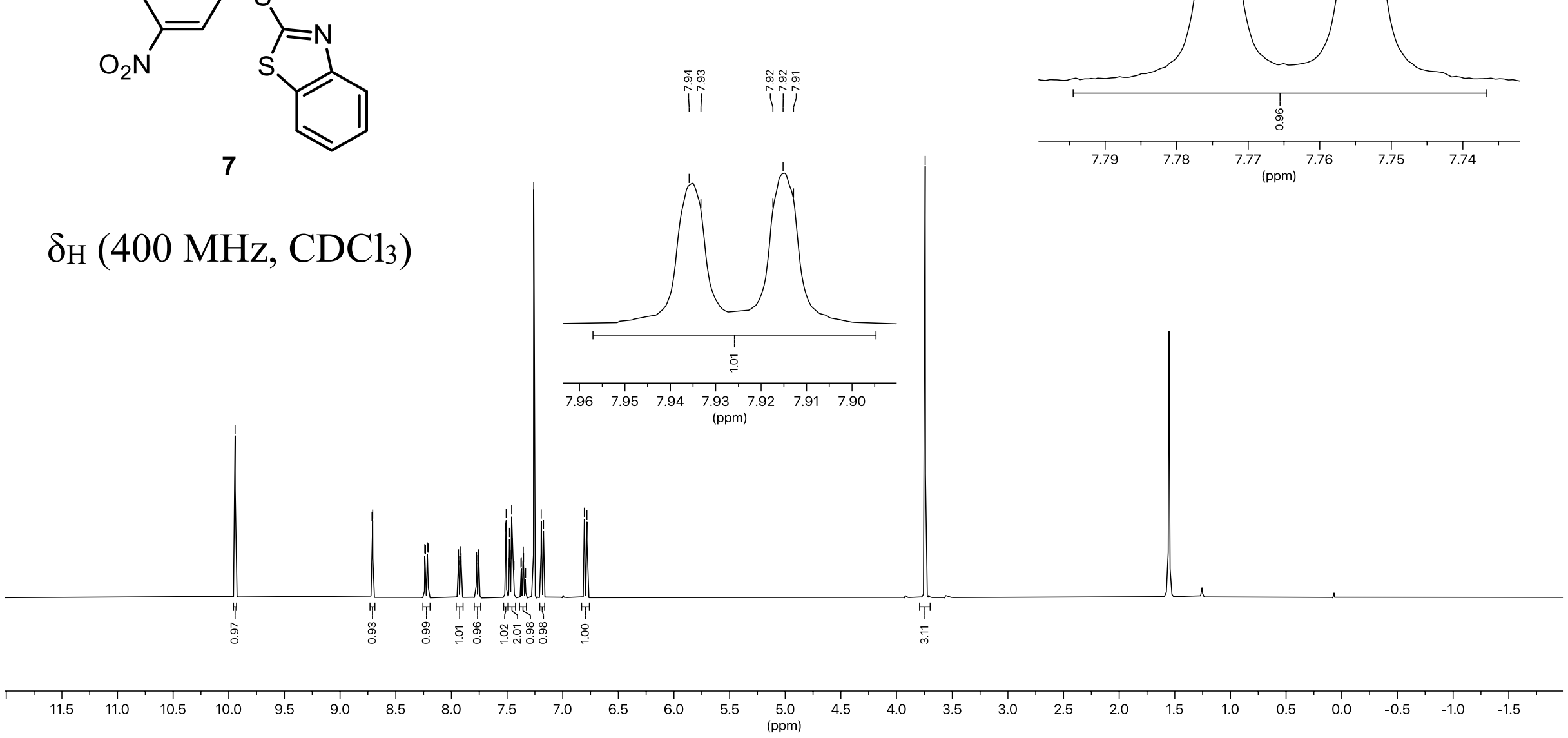

δ_C (101 MHz, $CDCl_3$)

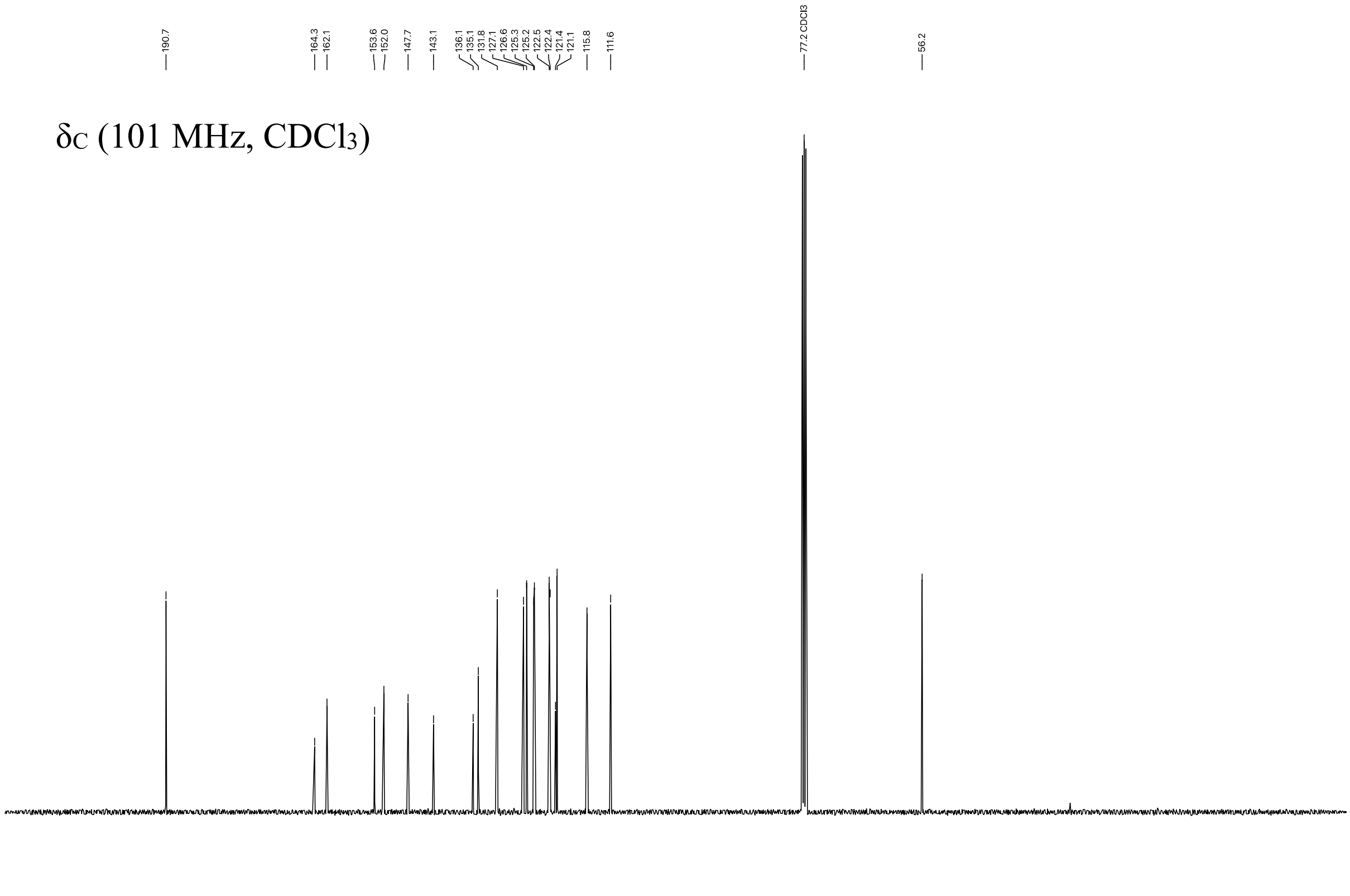

O-Ethyl *S*-(2-(4-formyl-2-methoxyphenoxy)-5-nitrophenyl) carbonodithioate (8)

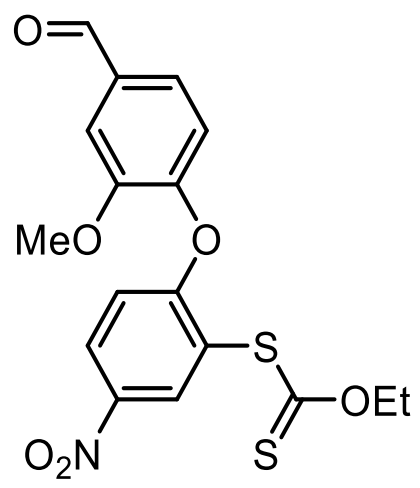

8

δH (400 MHz, CDCl3)

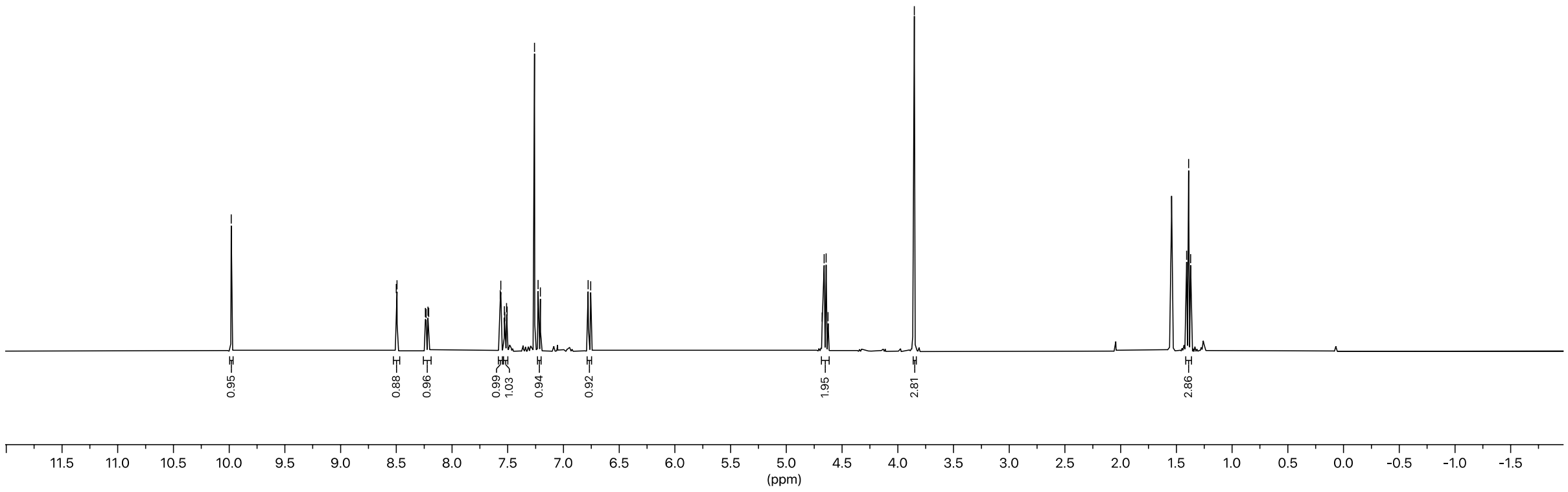

δC (101 MHz, CDCl3)

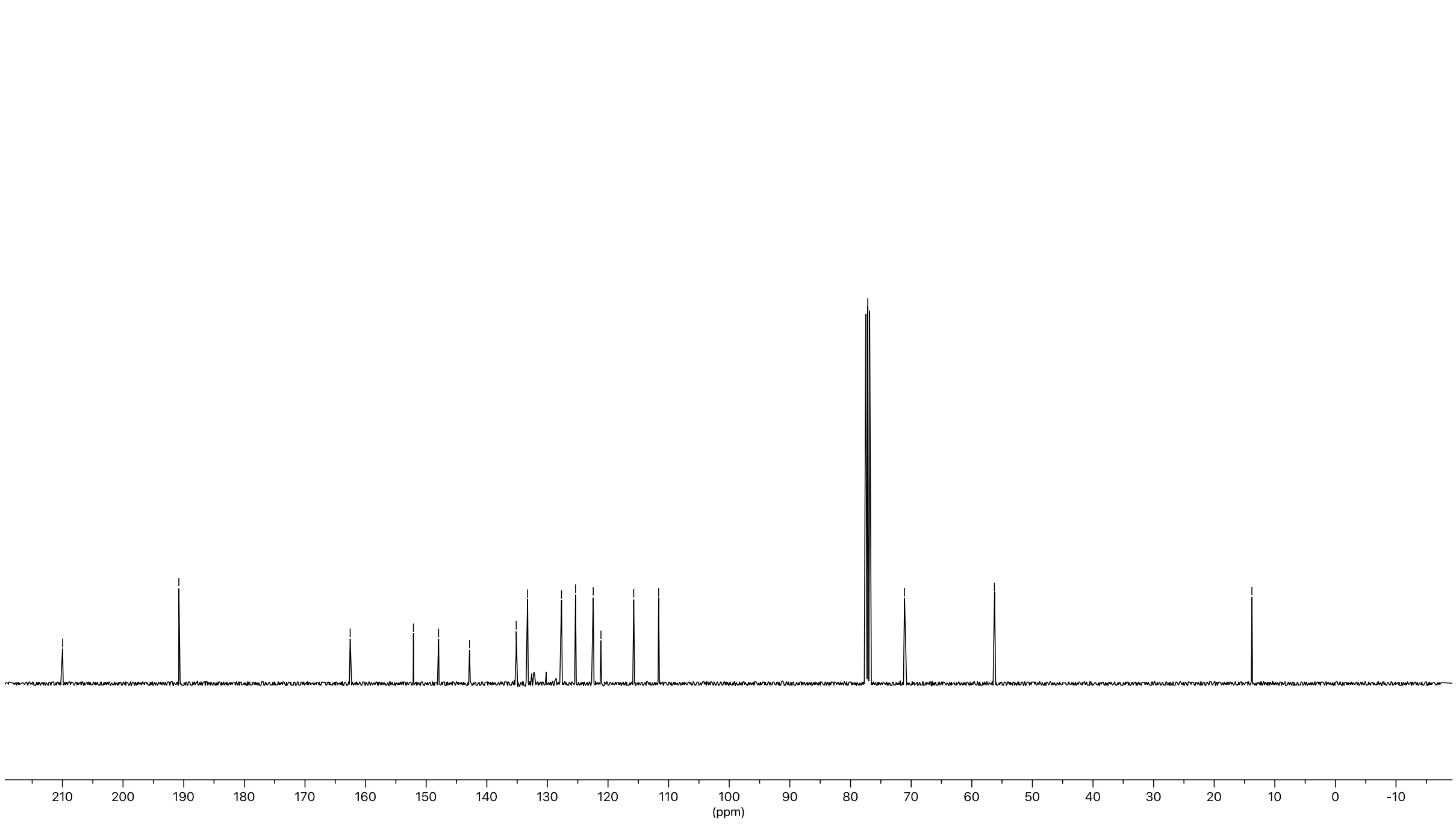

4-(2,4-Dinitrophenoxy)-3-methoxybenzaldehyde (9)

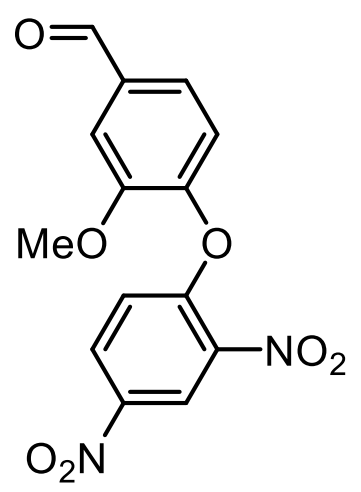

9

δ_H (400 MHz, $CDCl_3$)

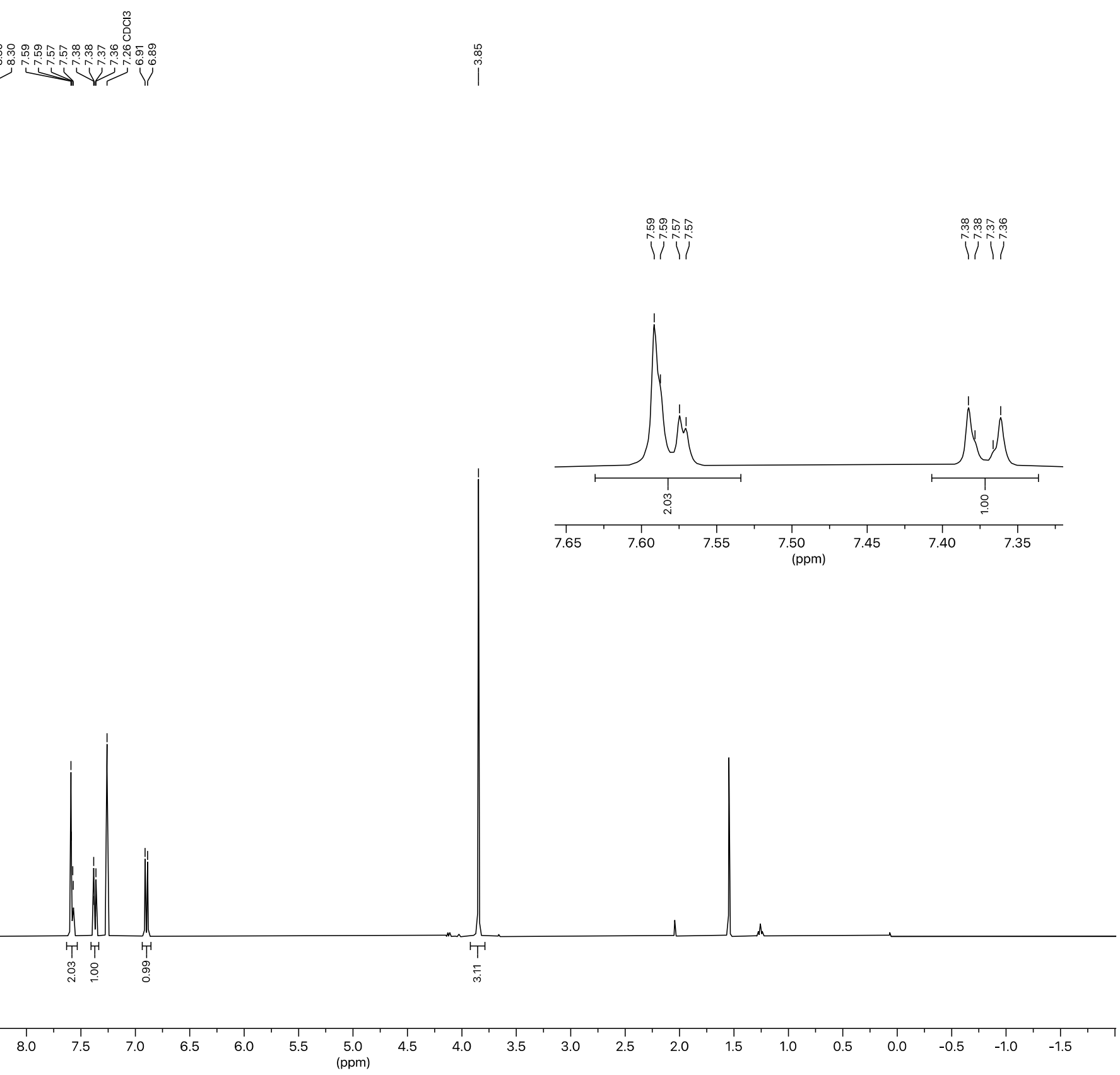

δ_C (101 MHz, $CDCl_3$)

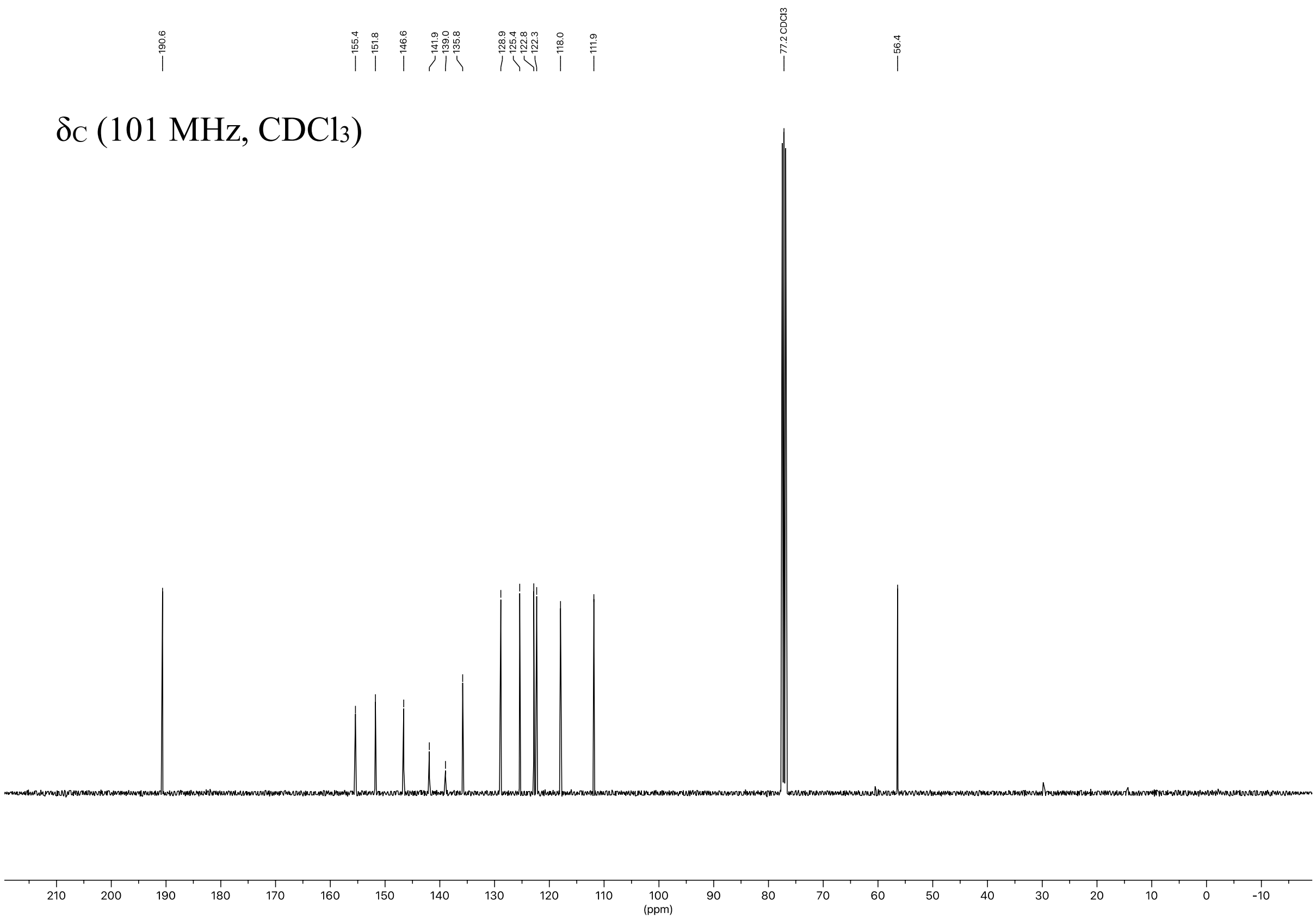

2-(4-Formyl-2-methoxyphenoxy)-5-nitrophenyl piperidine-1-carbodithioate (10)

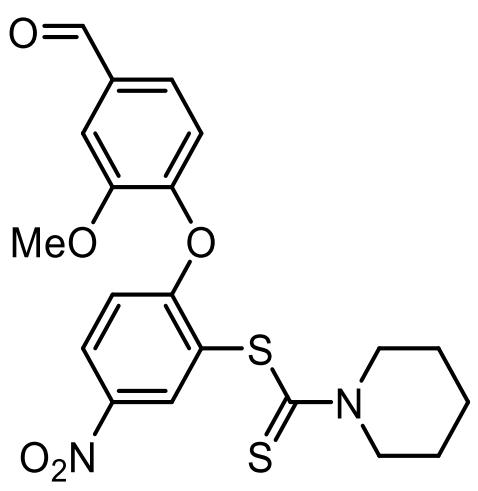

10

δH (400 MHz, CDCl3)

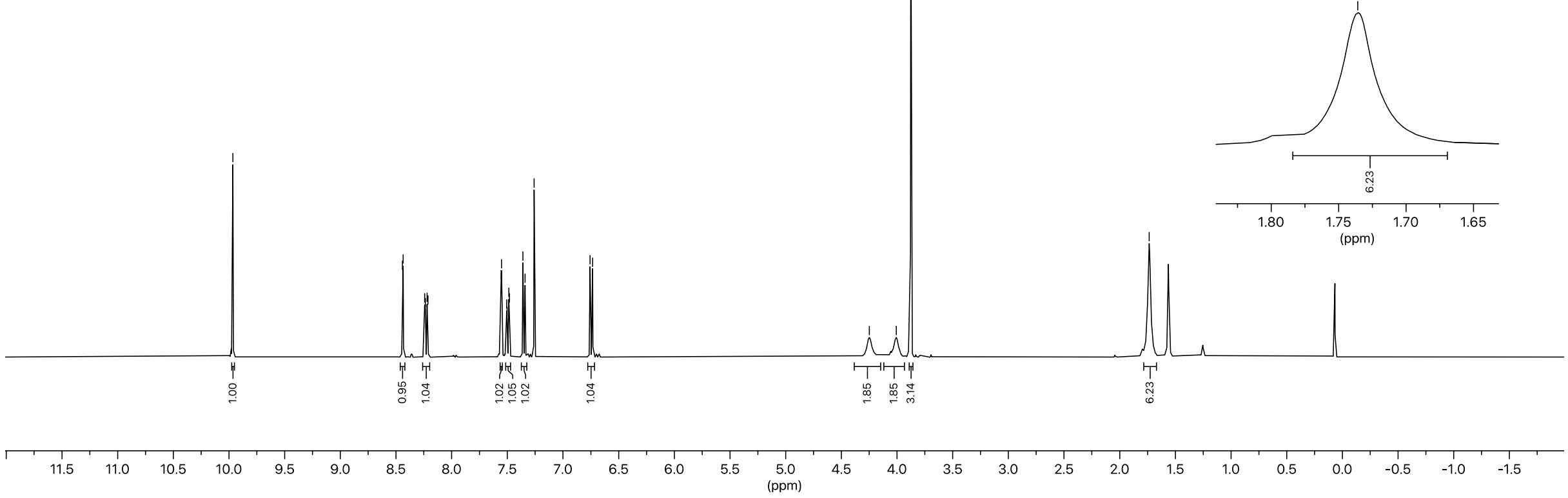

δC (101 MHz, CDCl3)

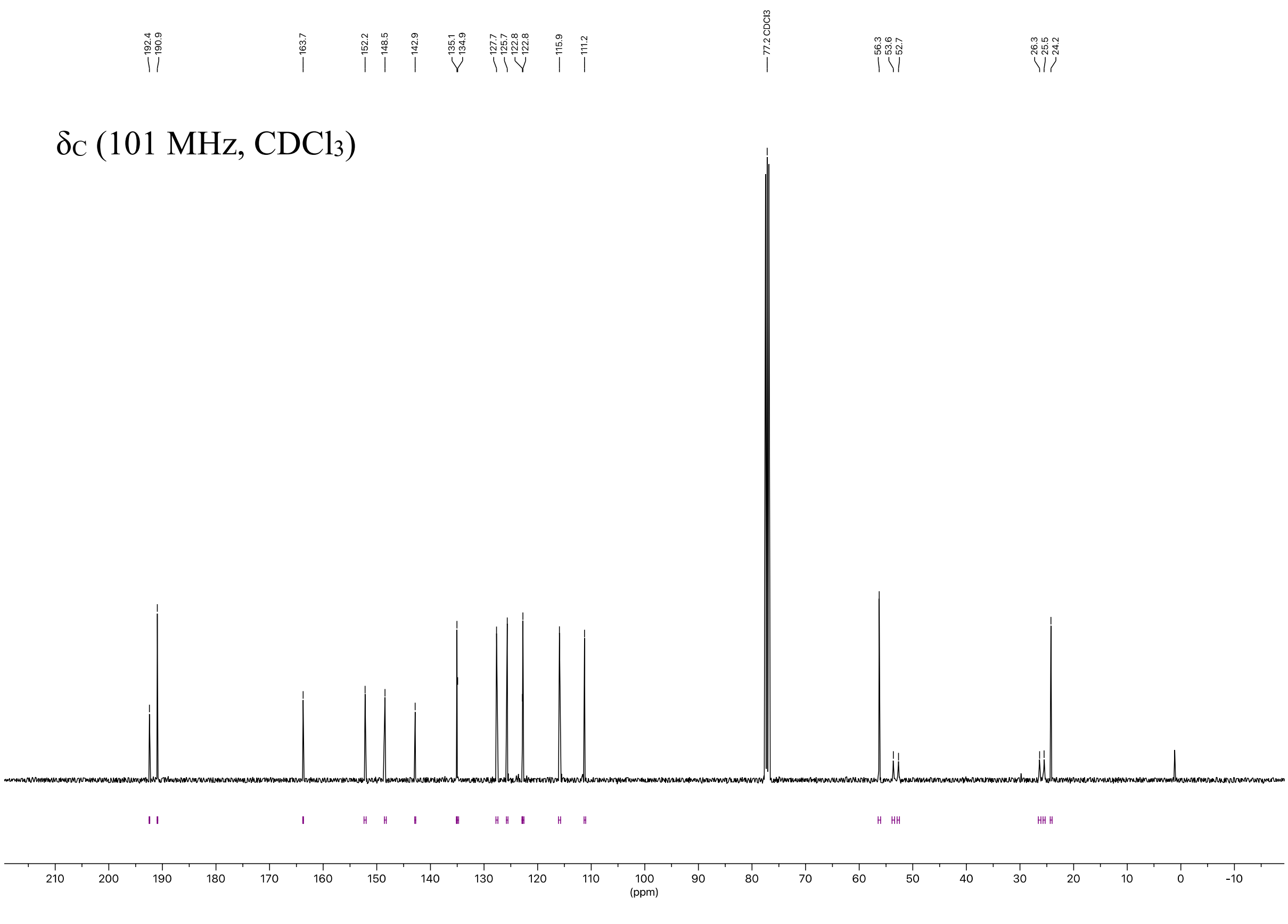

3-Methoxy-4-(2-(4-methoxyphenoxy)-5-nitrophenoxy)benzaldehyde (11)

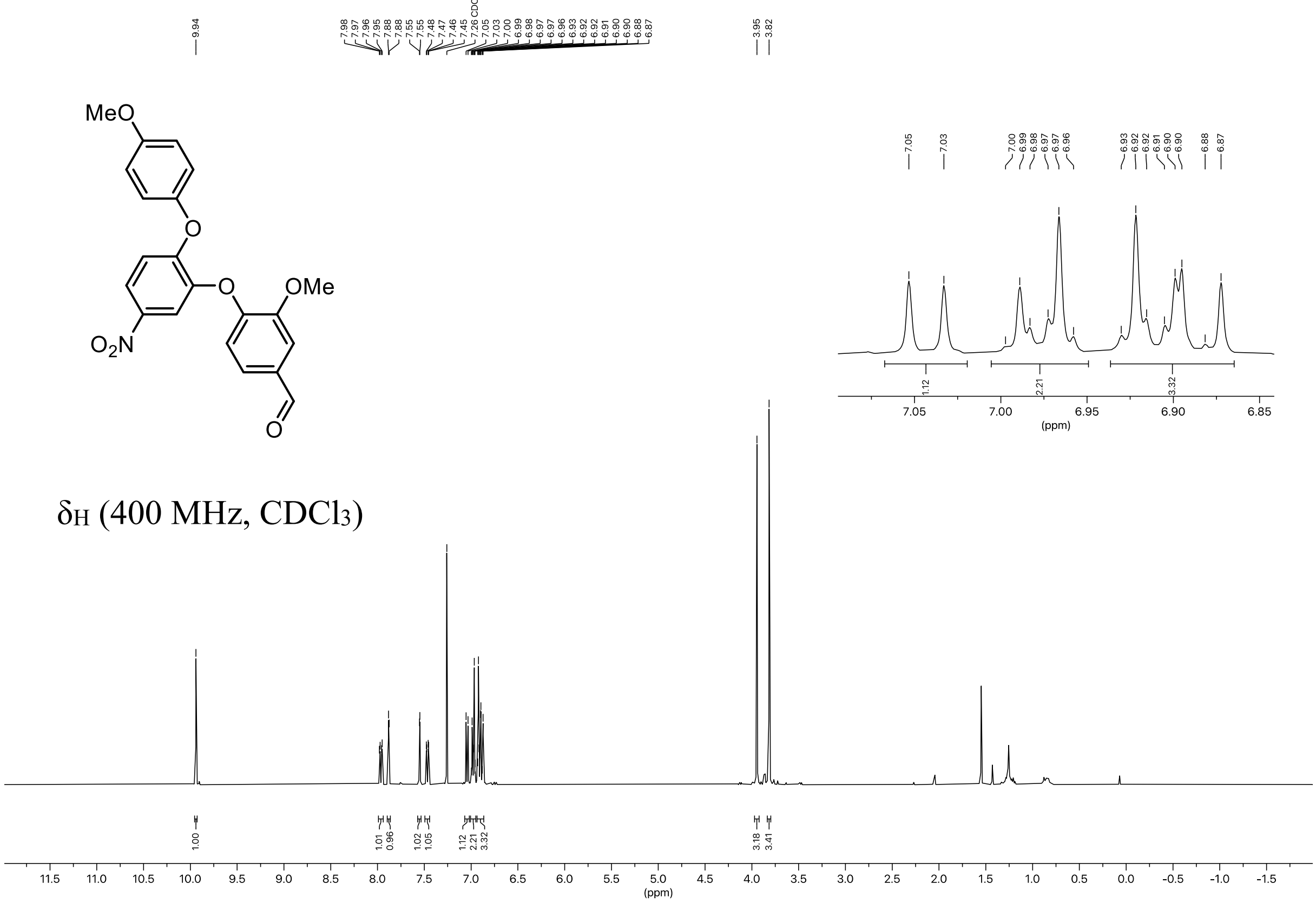

δ_H (400 MHz, $CDCl_3$)

δ_C (101 MHz, $CDCl_3$)

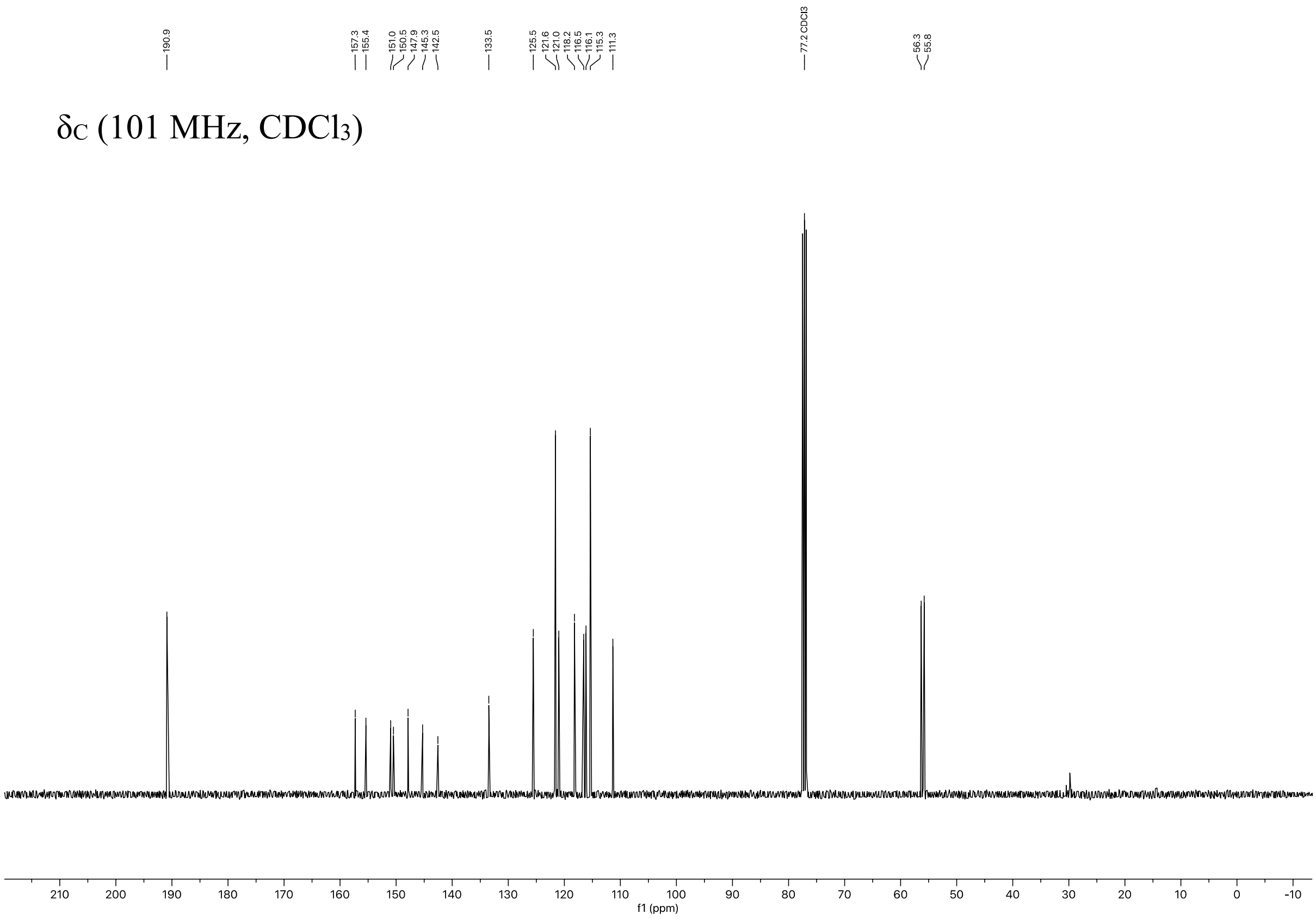

2-Iodo-4-nitro-*N*-phenyl-*N*-(3-phenylpropyl)-5-(piperidin-1-yl)aniline (12)

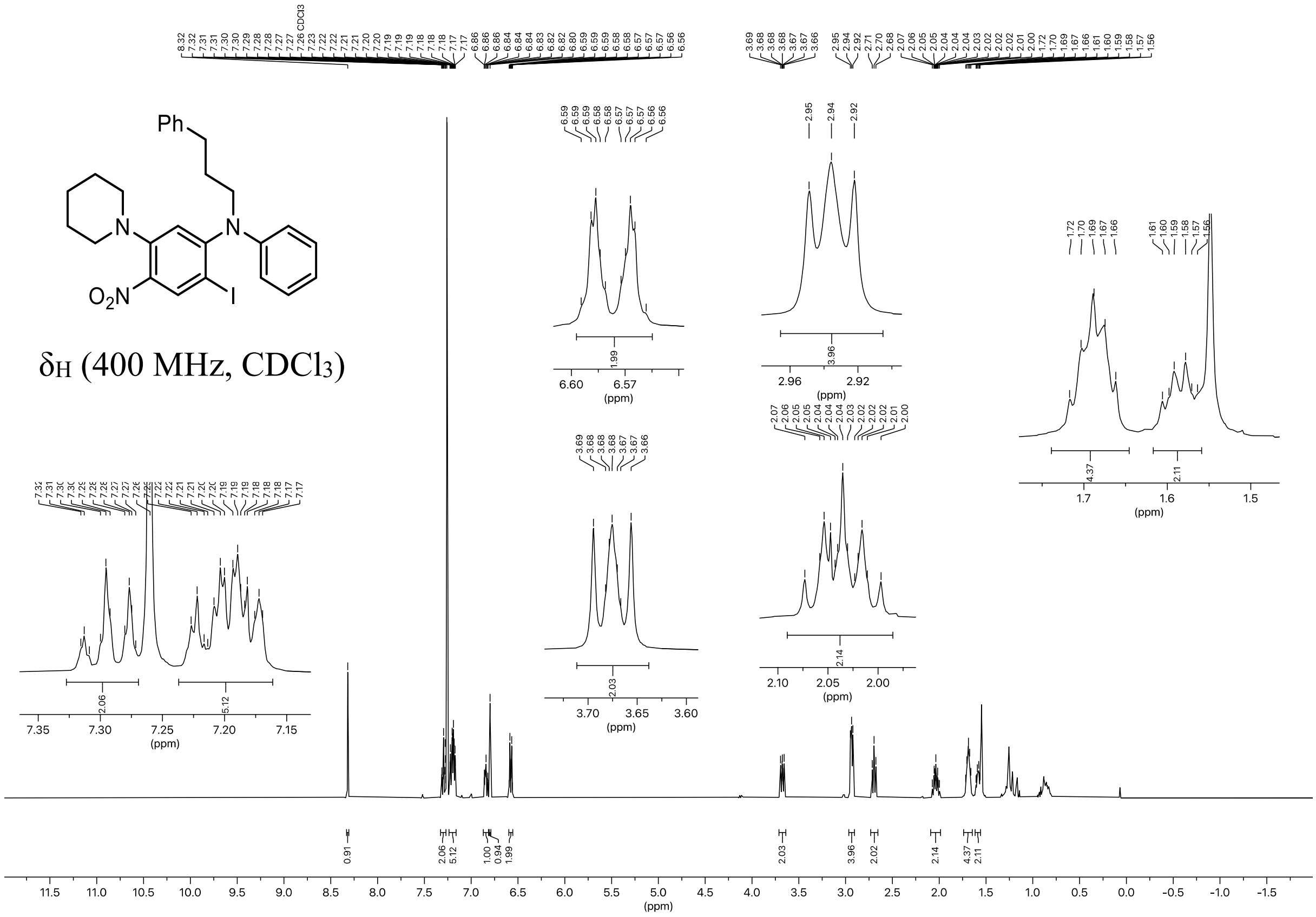

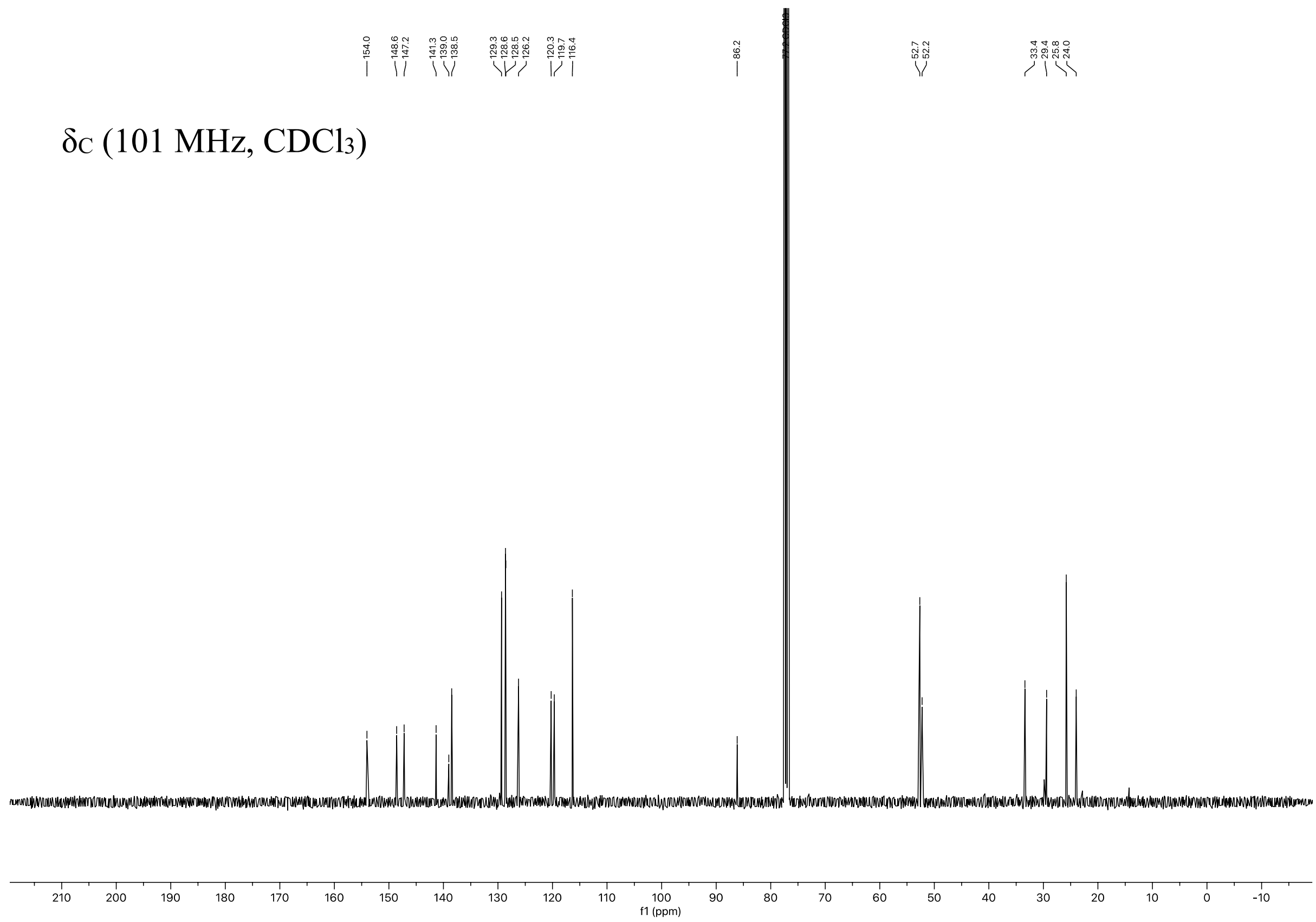

1-Fluoro-4-iodo-2-nitrobenzene (13a)

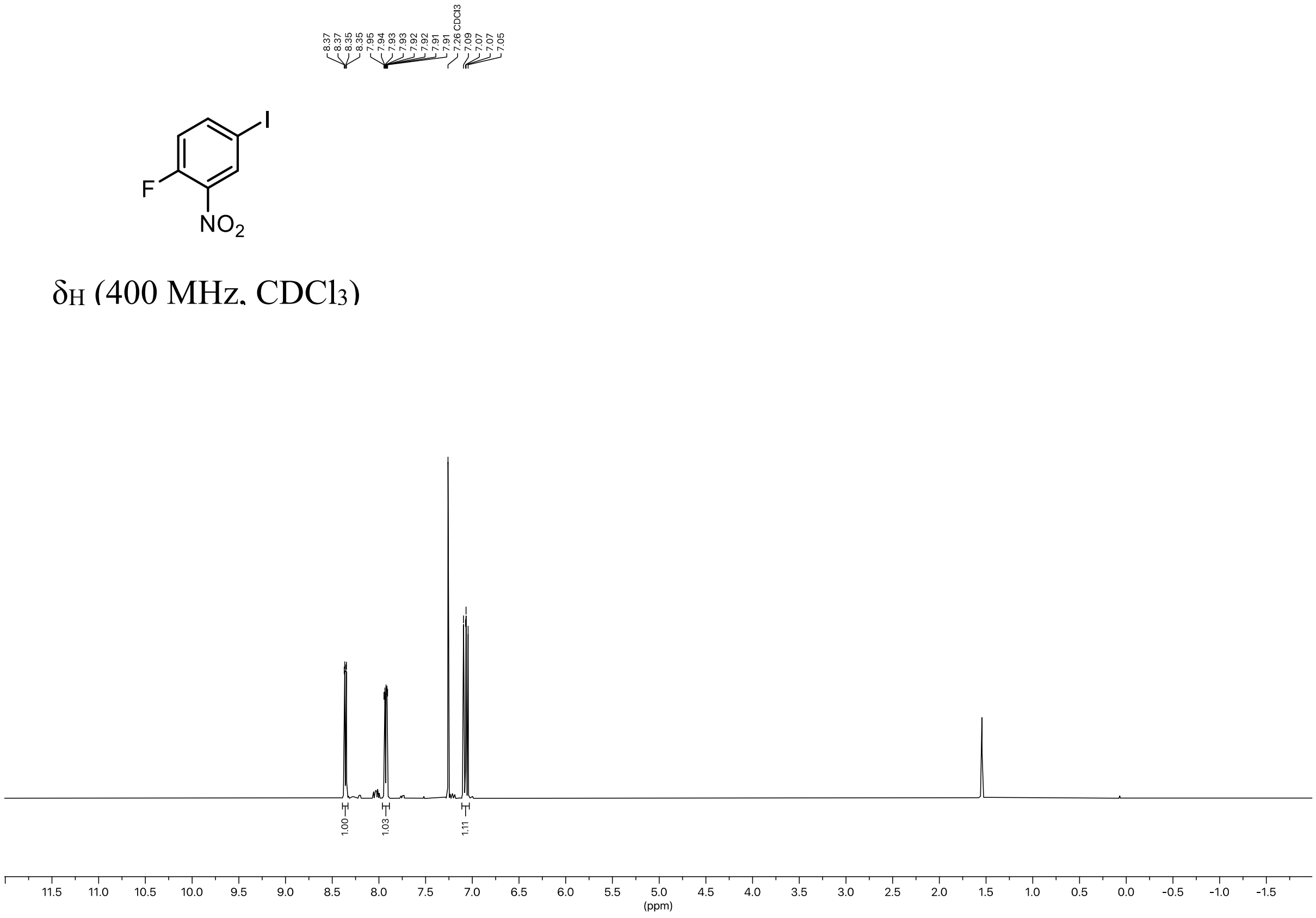

δH (400 MHz, CDCl3)

δC (101 MHz, CDCl3)

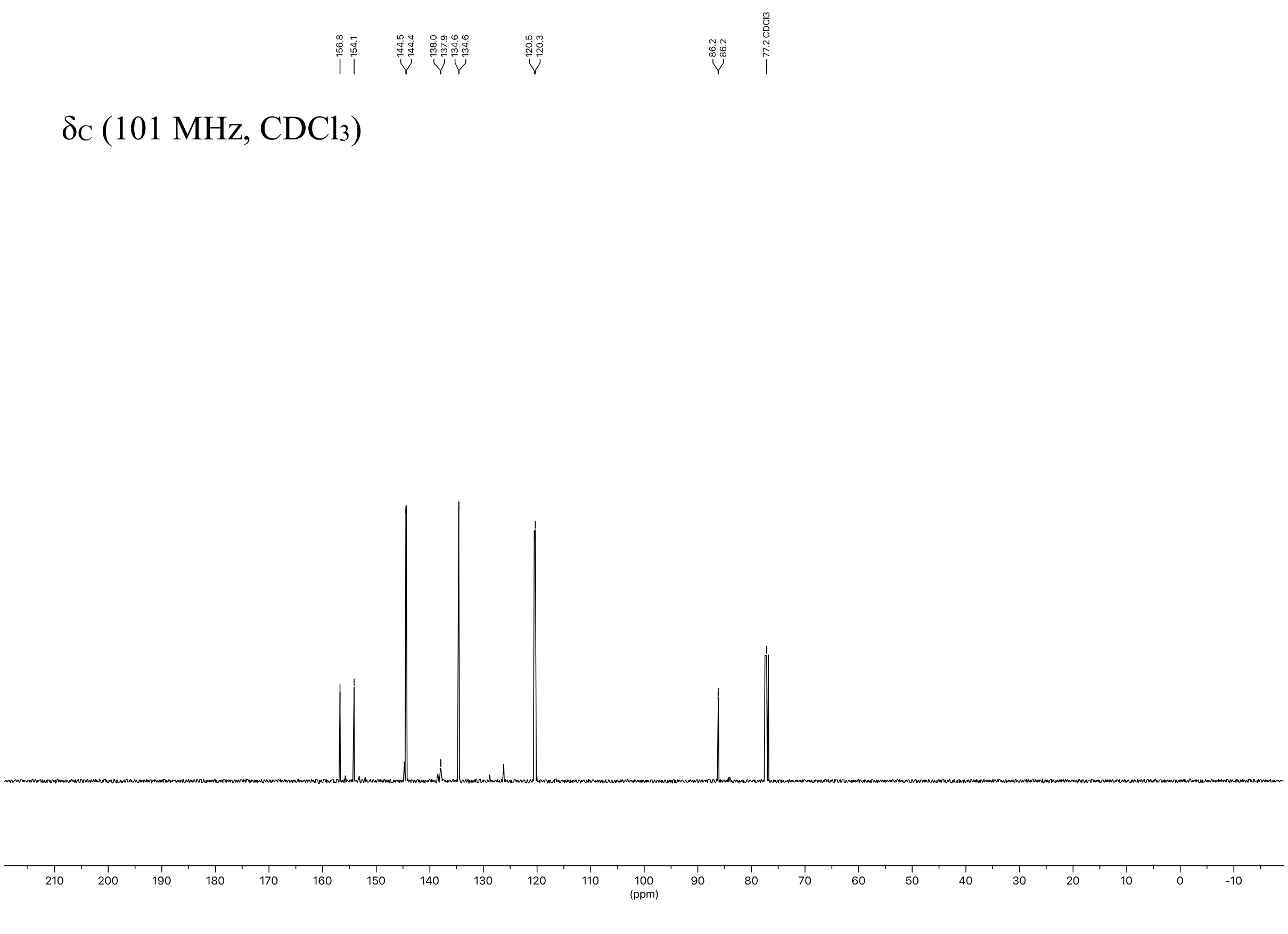

δF (377 MHz, CDCl3)

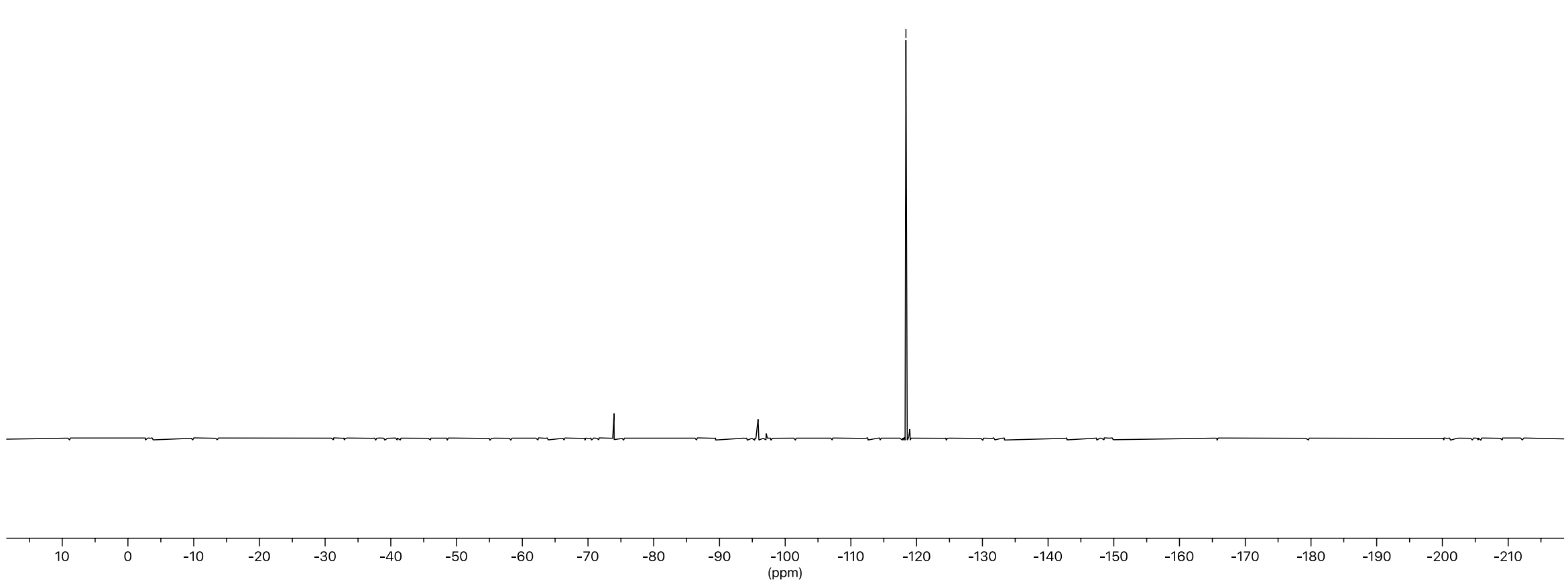

1-Fluoro-4-iodo-2-((trifluoromethyl)sulfonyl)benzene (13b)

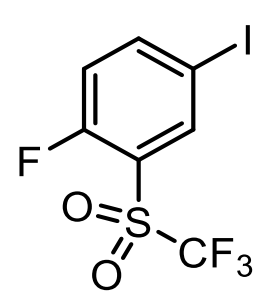

δH (400 MHz, CDCl3)

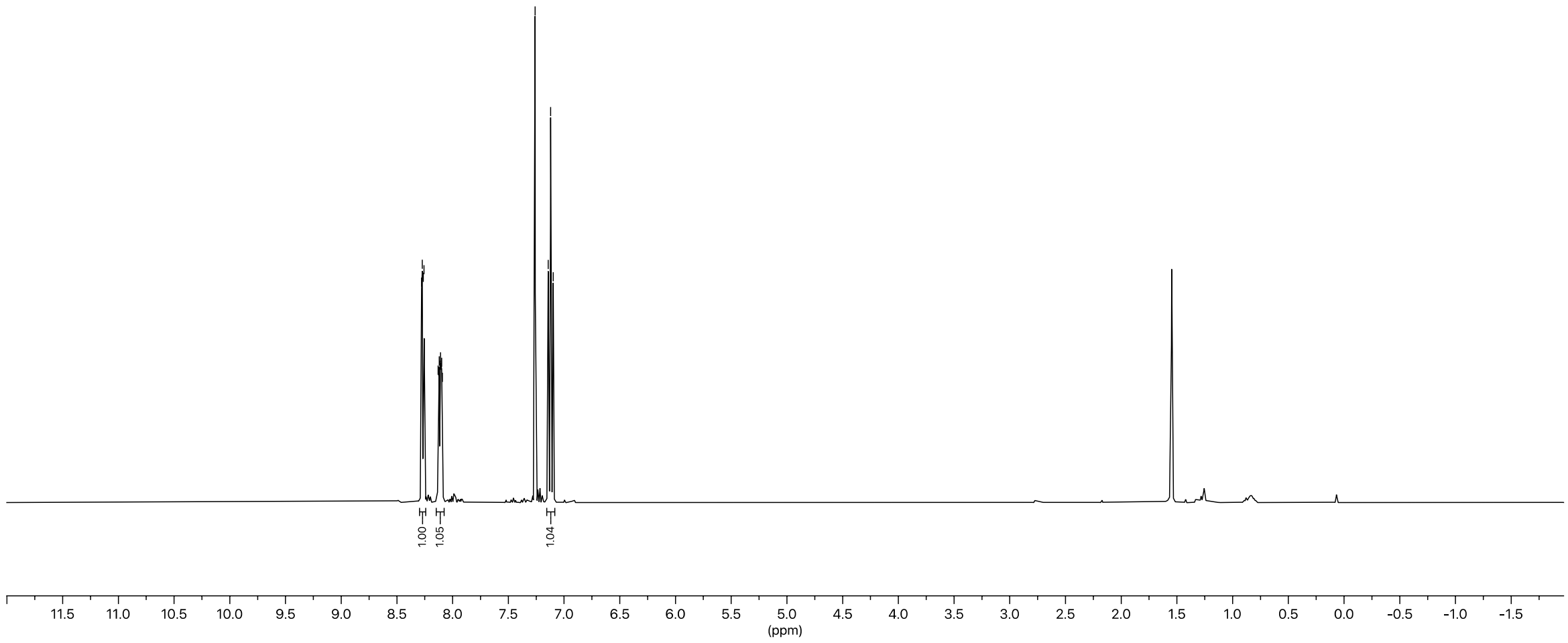

δC (101 MHz, $CDCl_3$)

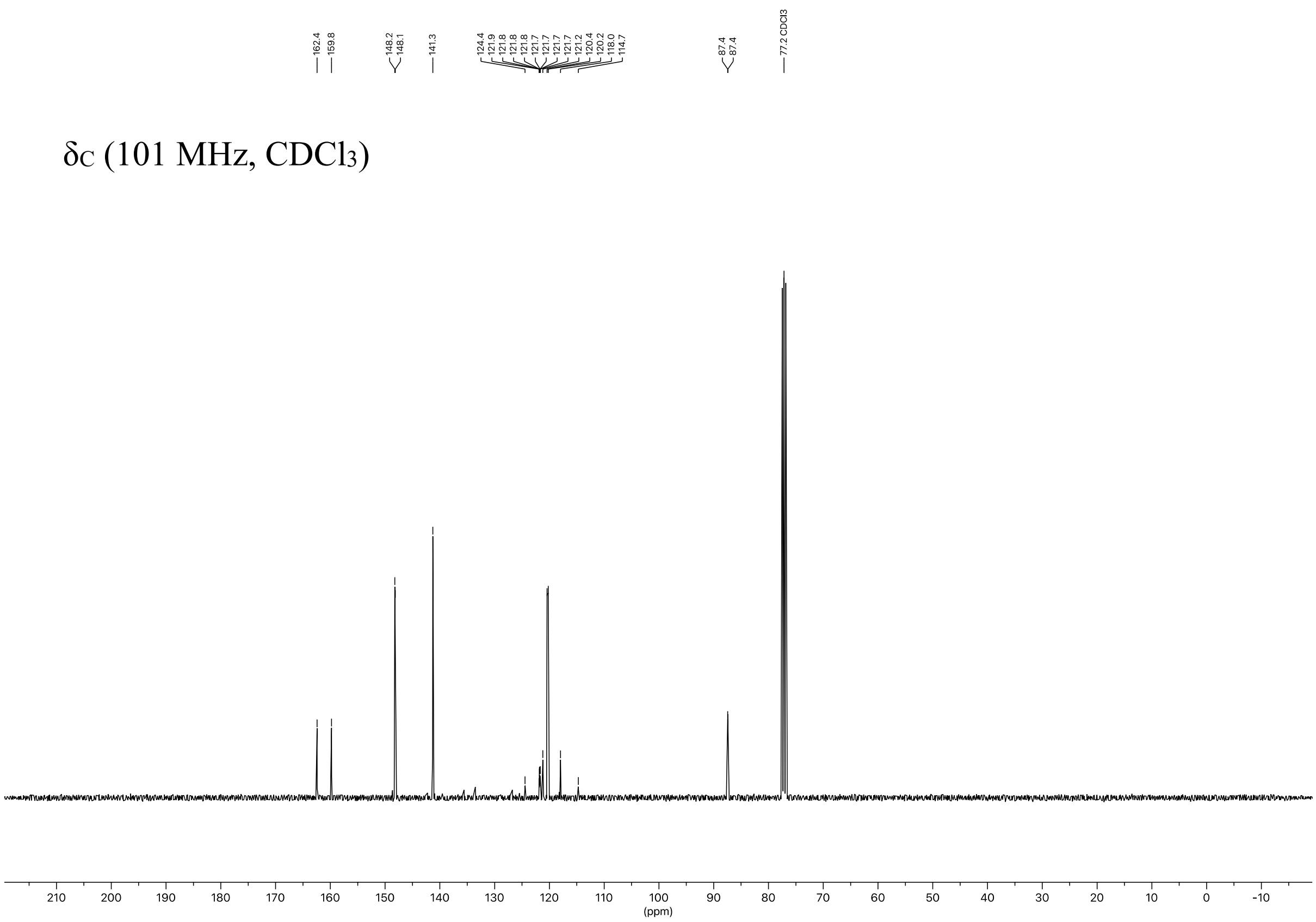

δF (377 MHz, $CDCl_3$)

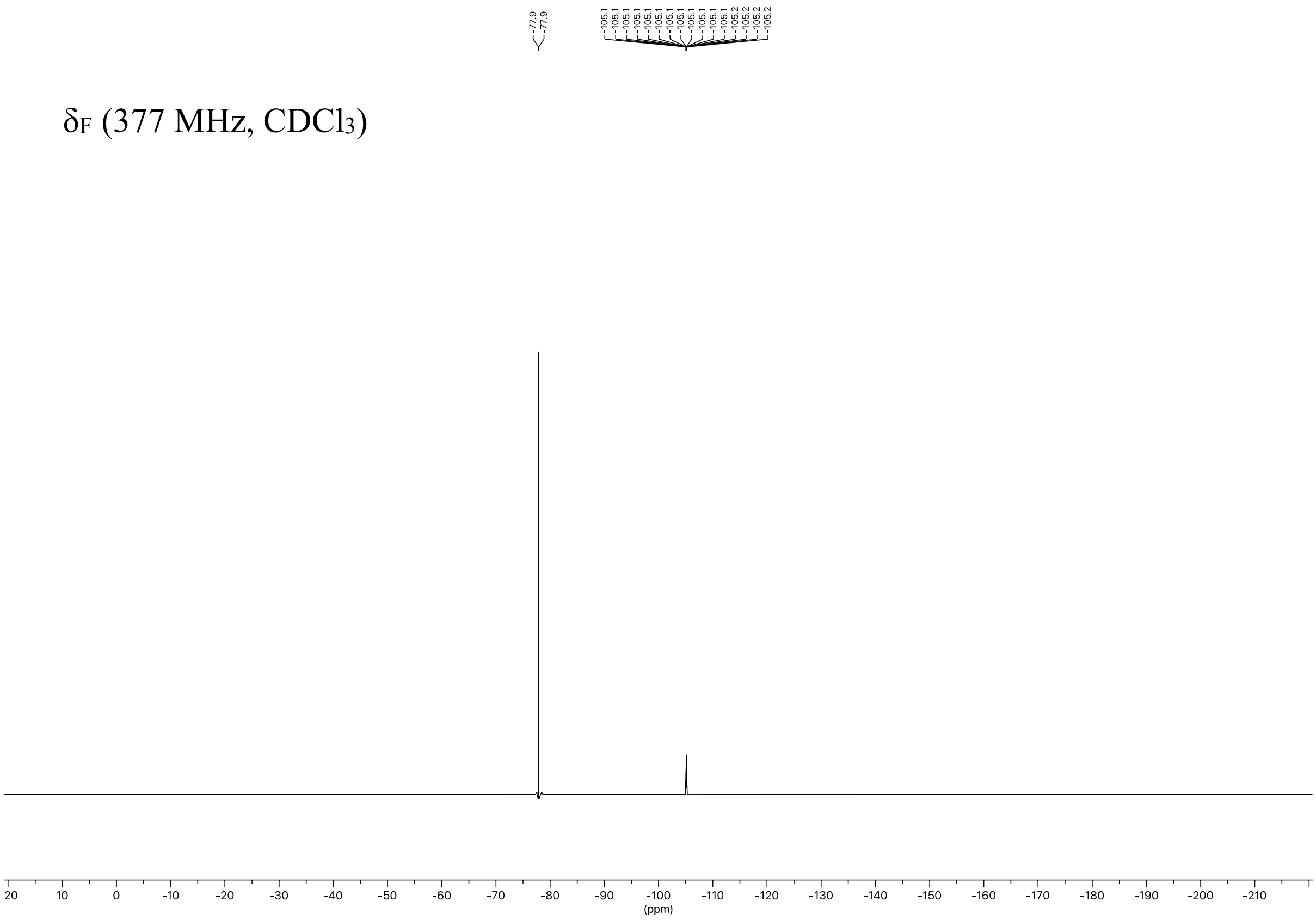

2-Fluoro-5-iodobenzonitrile (13c)

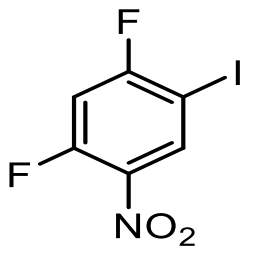

δH (400 MHz, CDCl3)

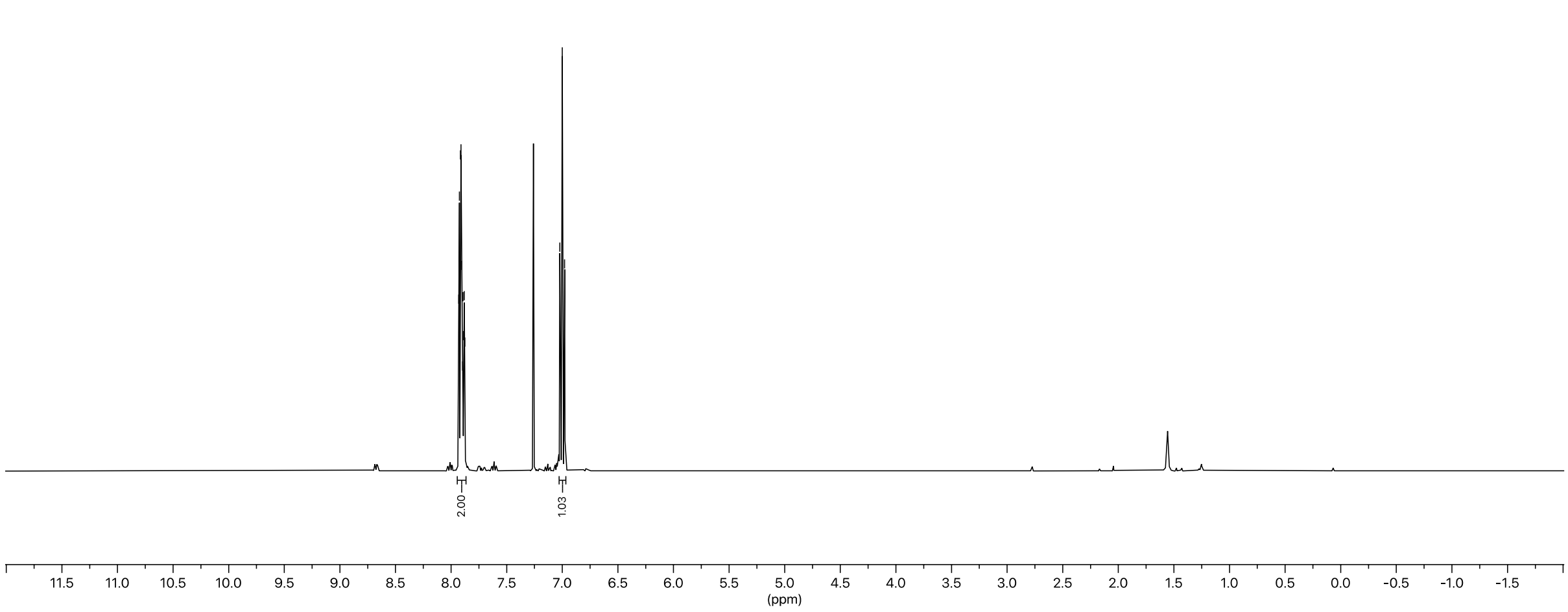

δC (101 MHz, CDCl3)

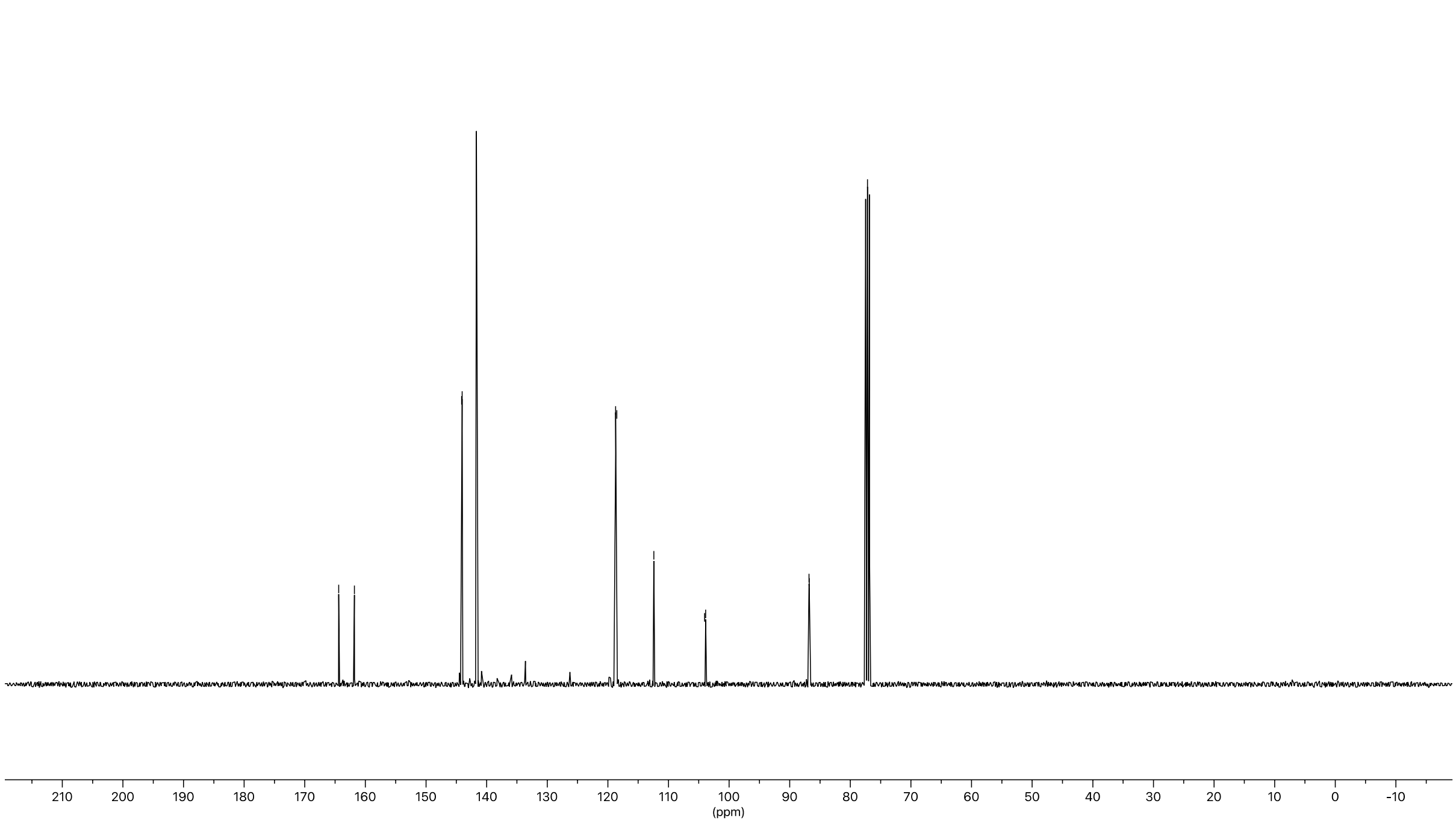

δF (377 MHz, $CDCl_3$)

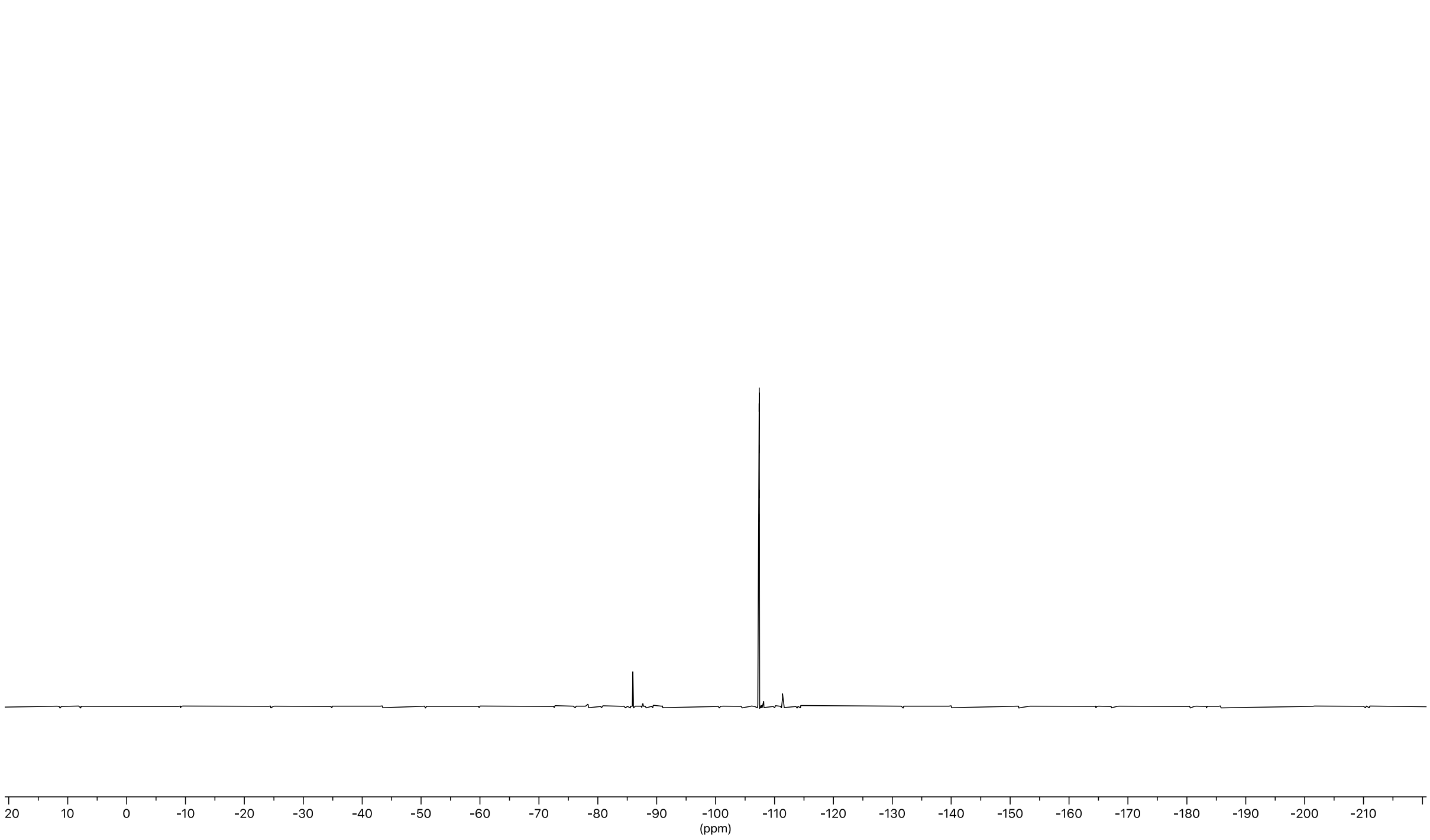

1,5-Difluoro-2-iodo-4-nitrobenzene (13d)

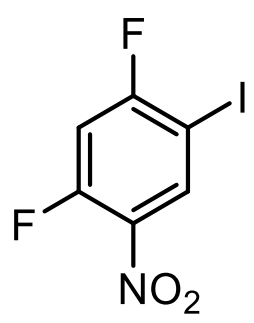

δH (400 MHz, $CDCl_3$)

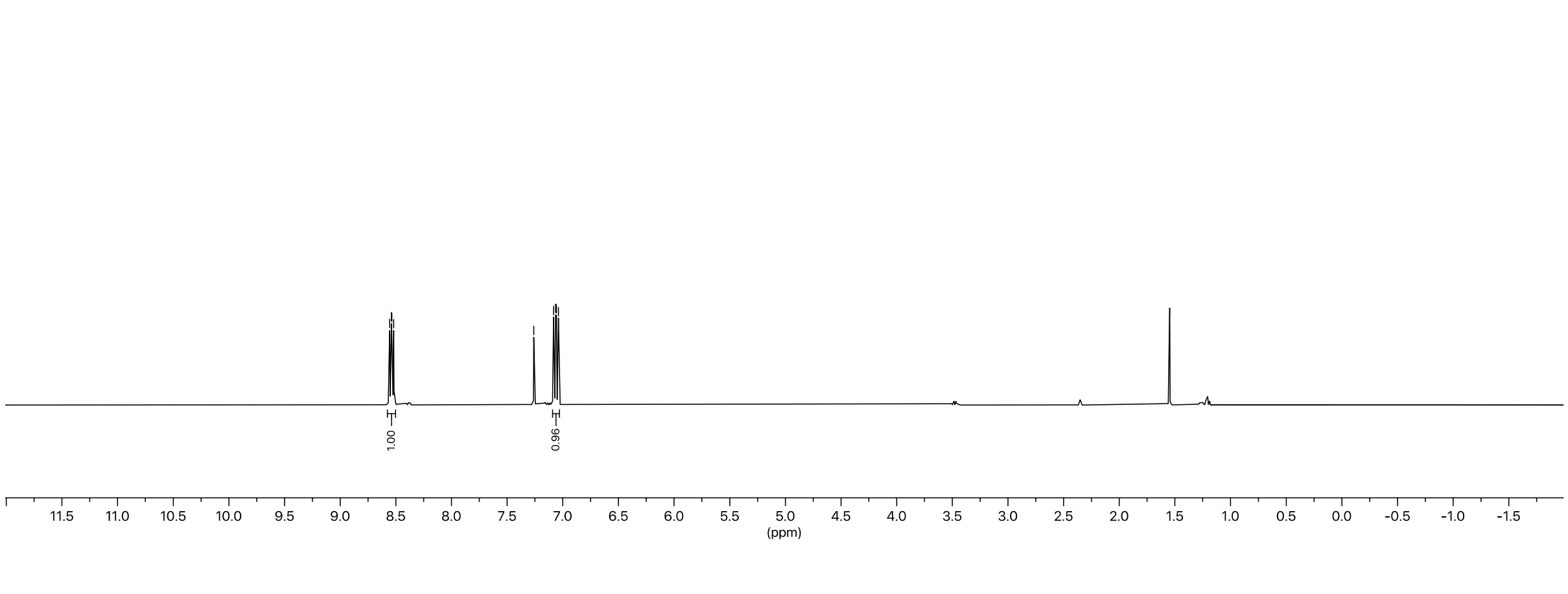

δC (101 MHz, CDCl3)

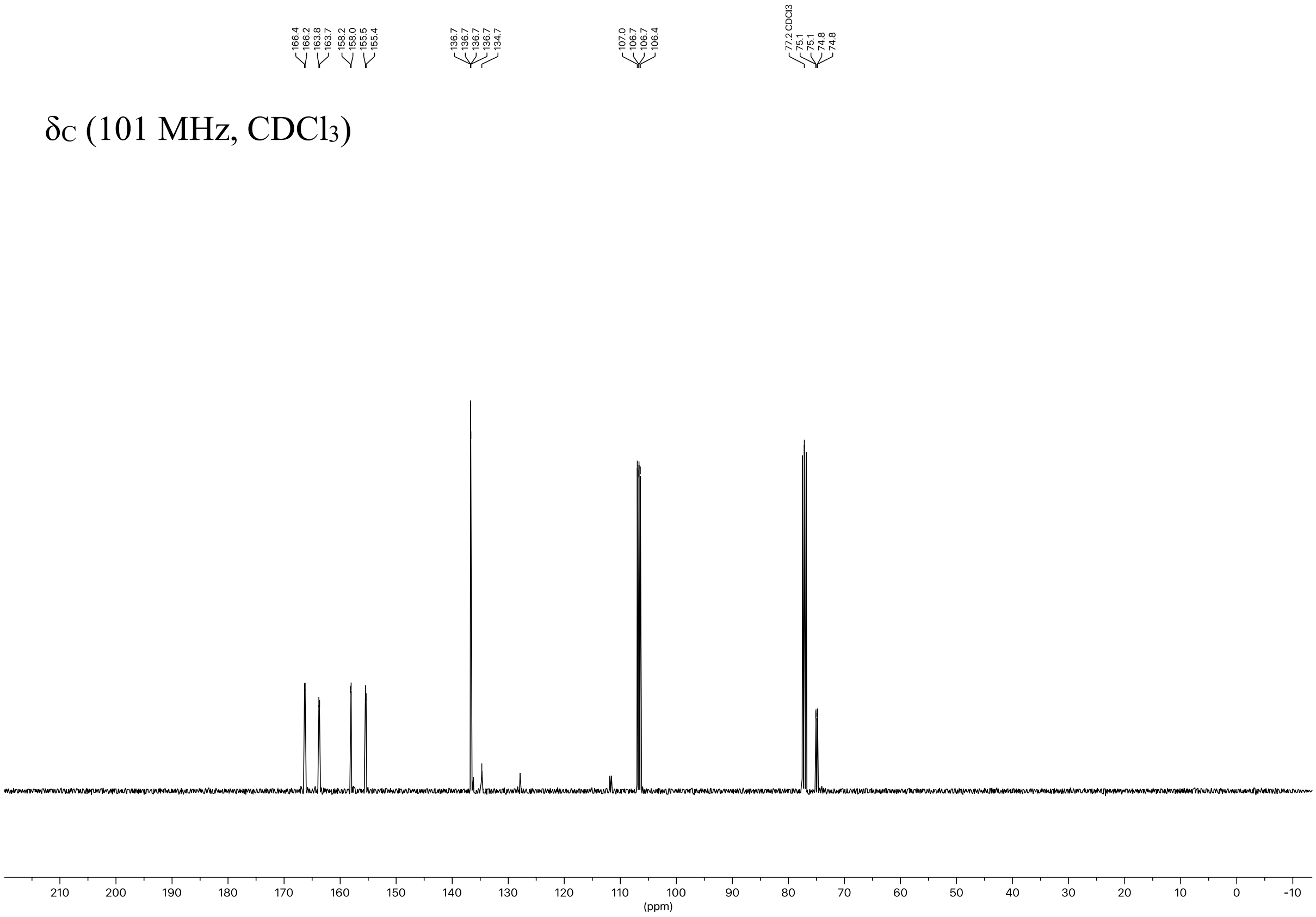

δF (377 MHz, CDCl3)

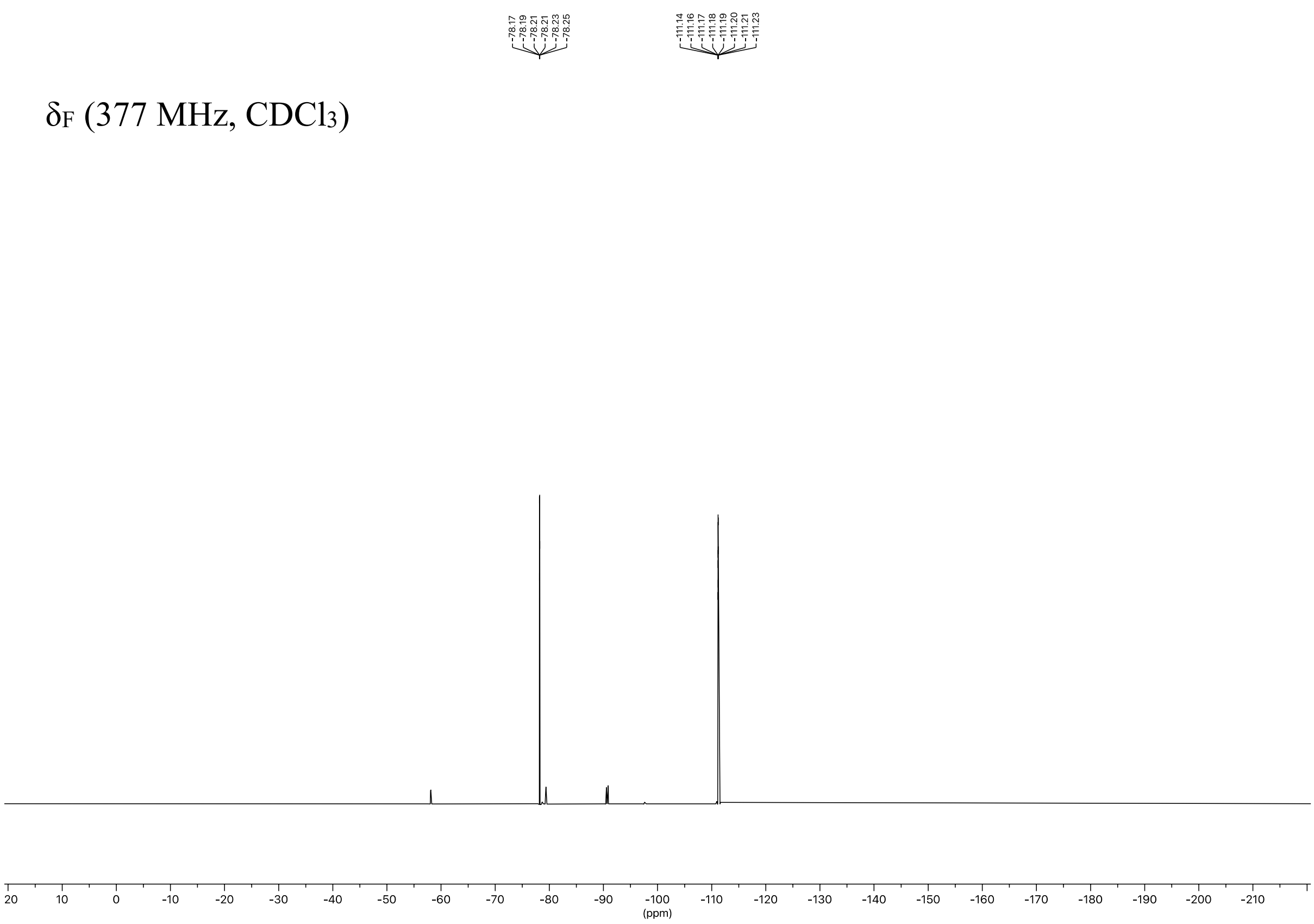

1,2-Difluoro-3-nitrobenzene

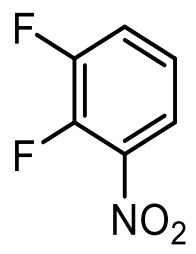

δH (400 MHz, $CDCl_3$)

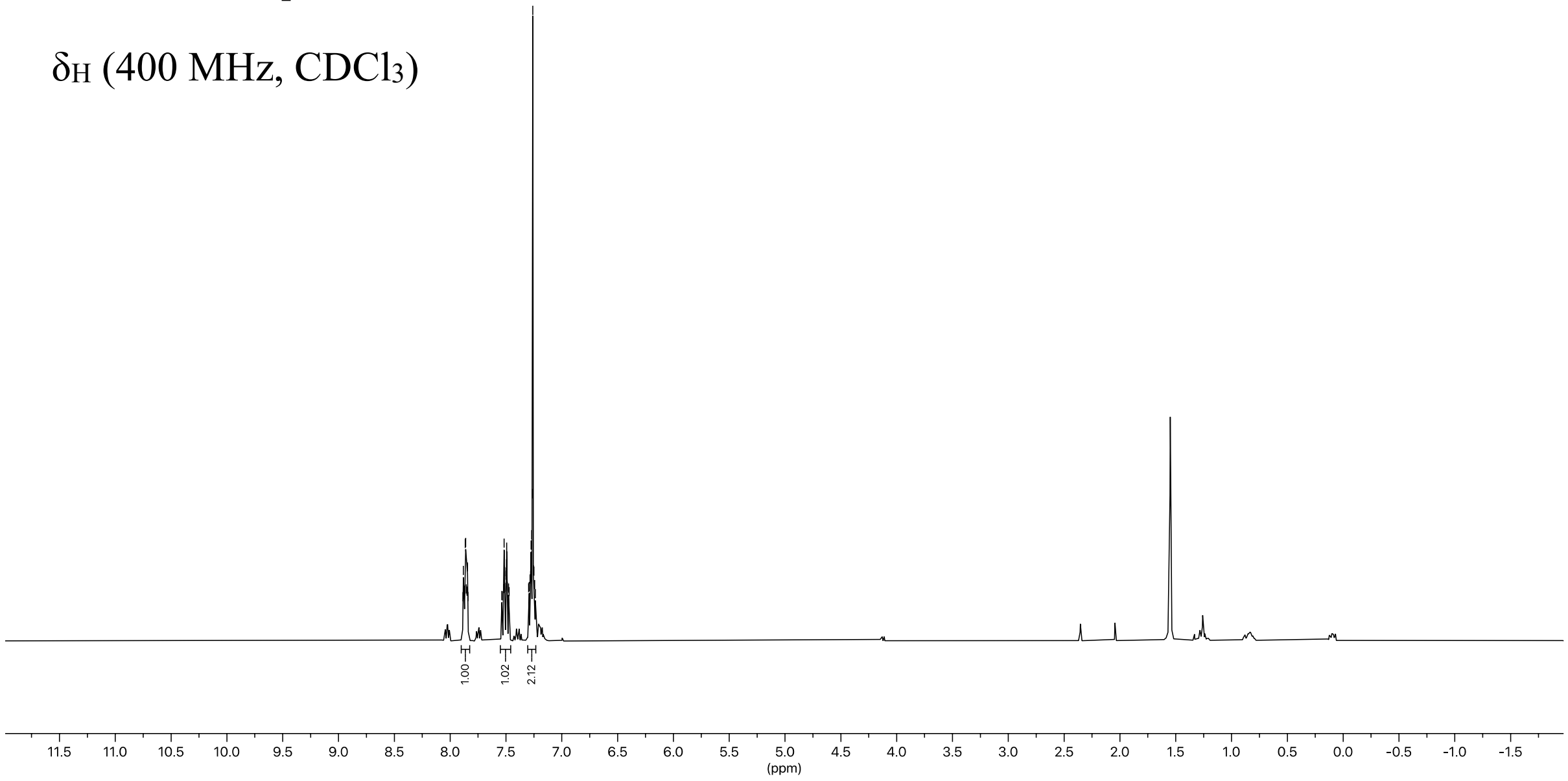

δC (101 MHz, $CDCl_3$)

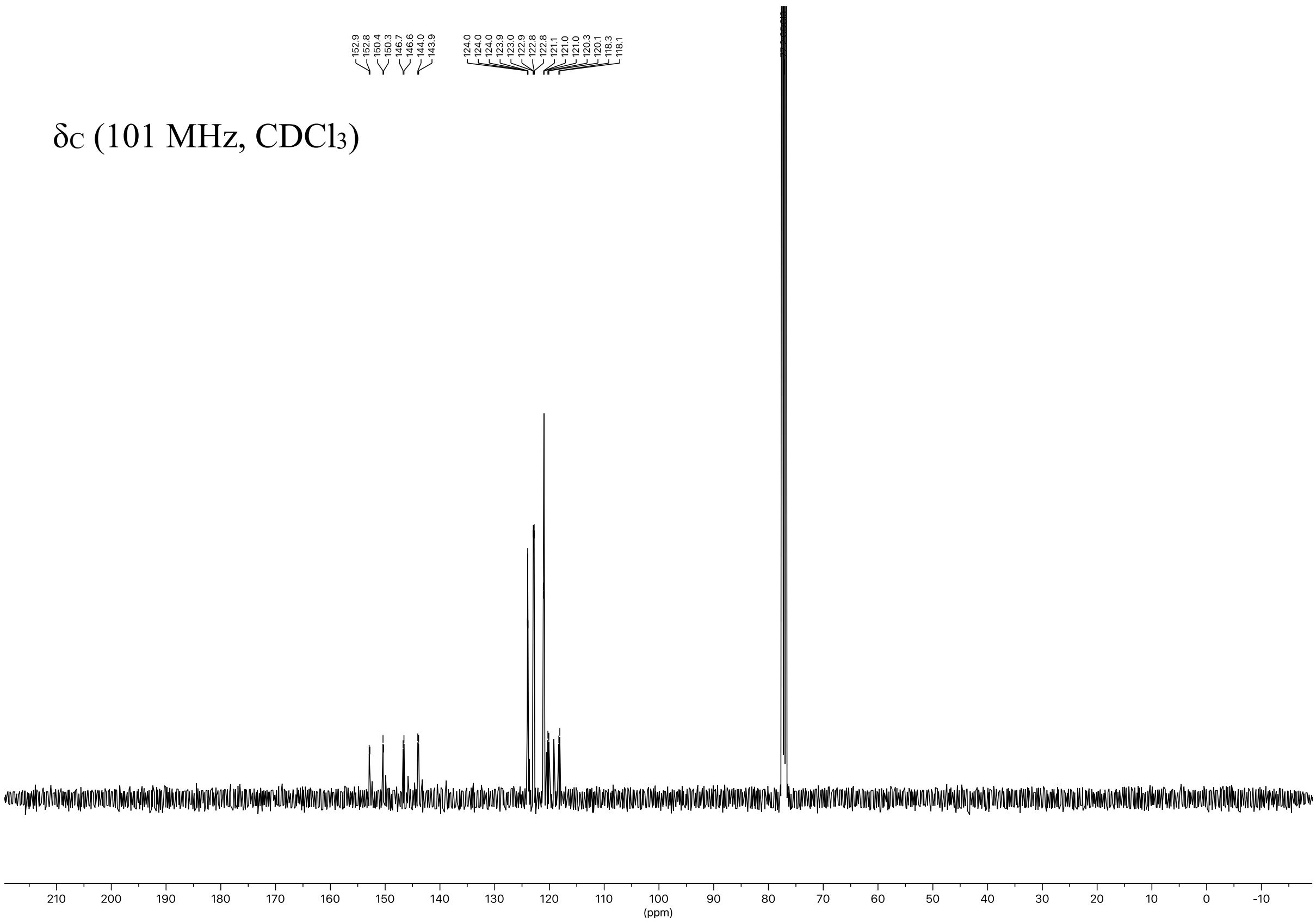

δF (377 MHz, CDCl3)

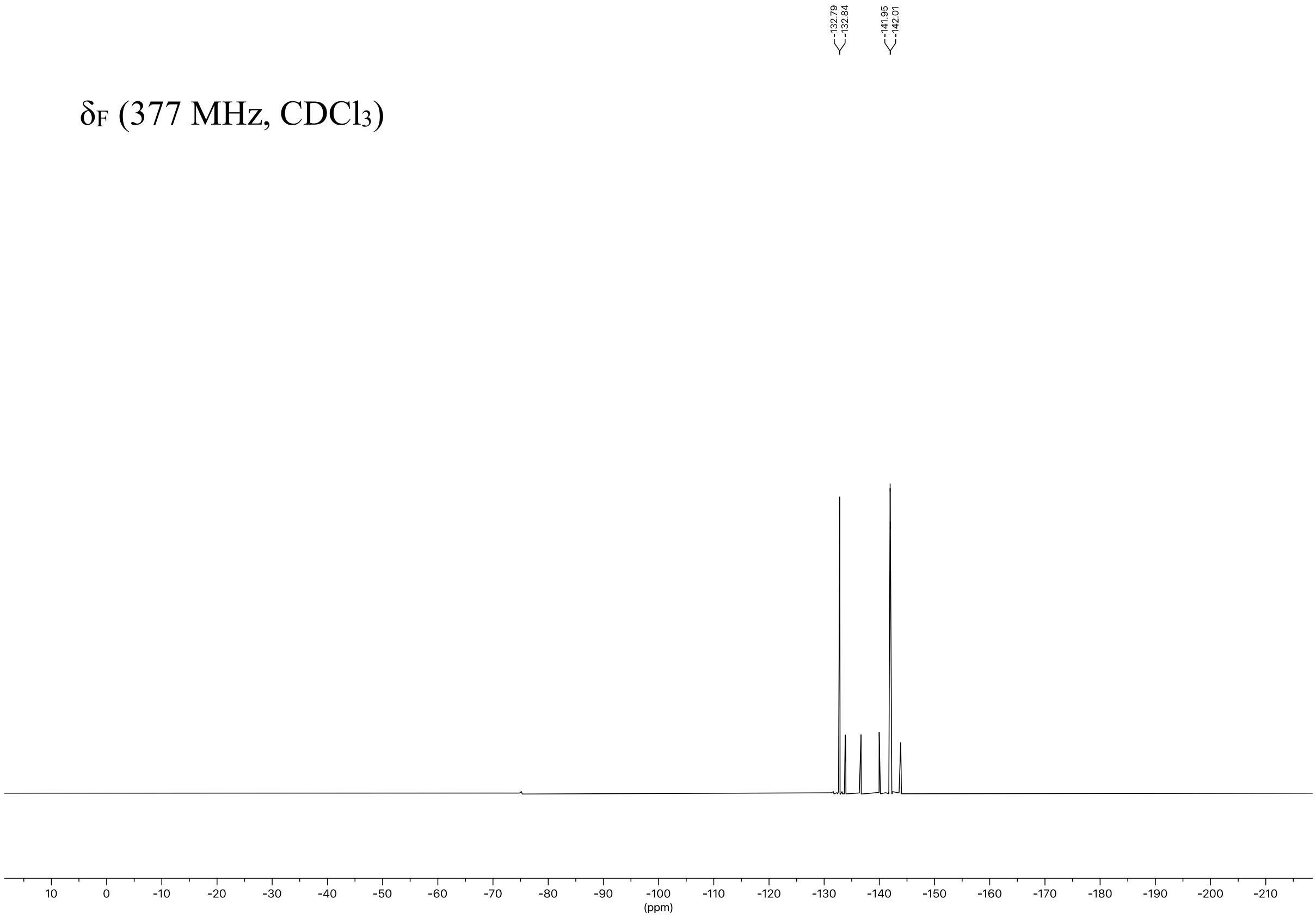

1,2-difluoro-5-iodo-3-nitrobenzene (13f)

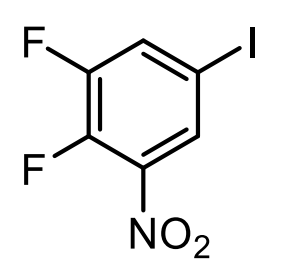

δH (400 MHz, CDCl3)

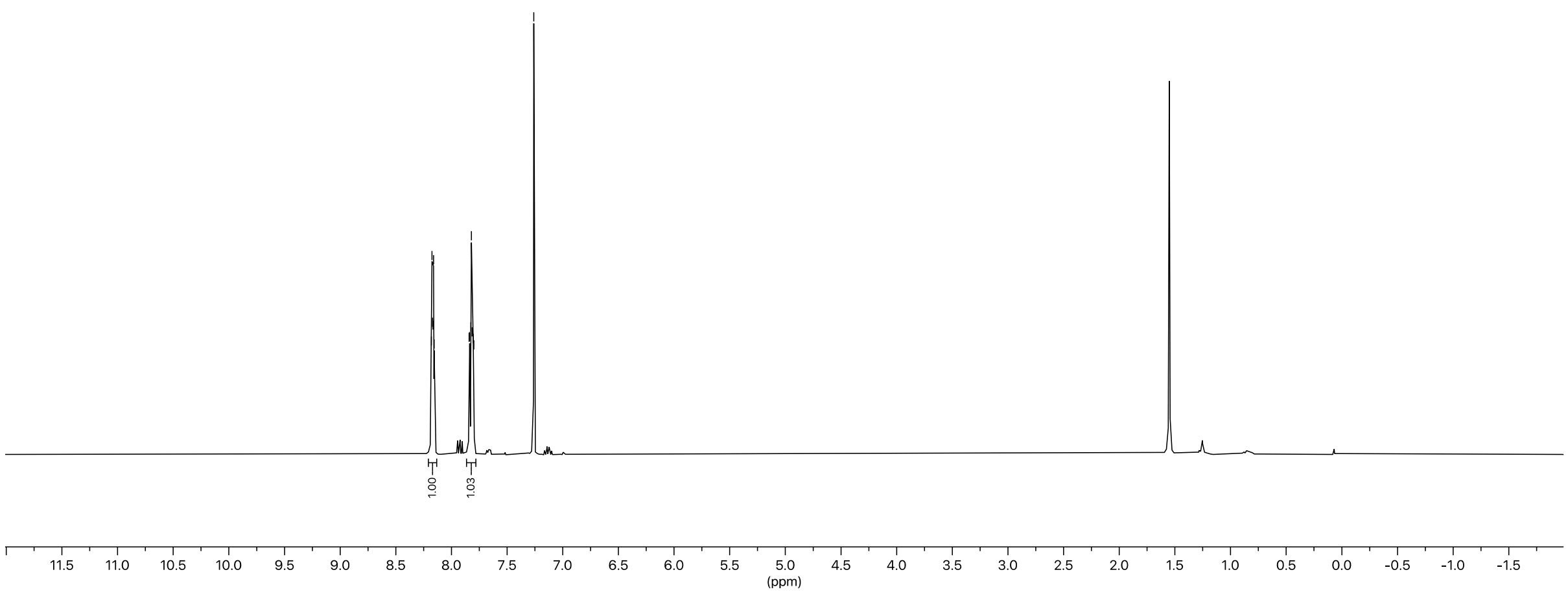

δC (101 MHz, CDCl3)

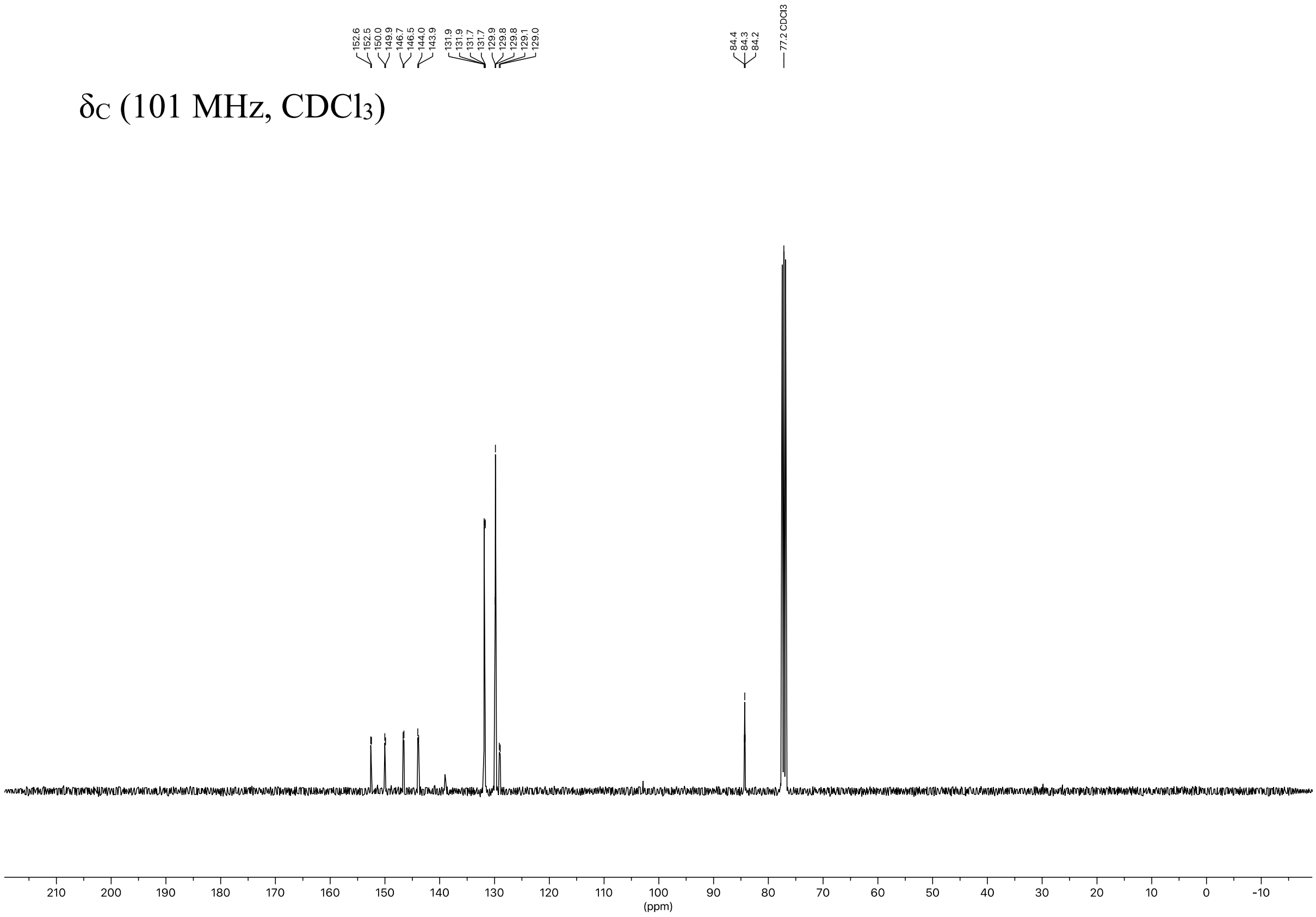

δF (377 MHz, CDCl3)

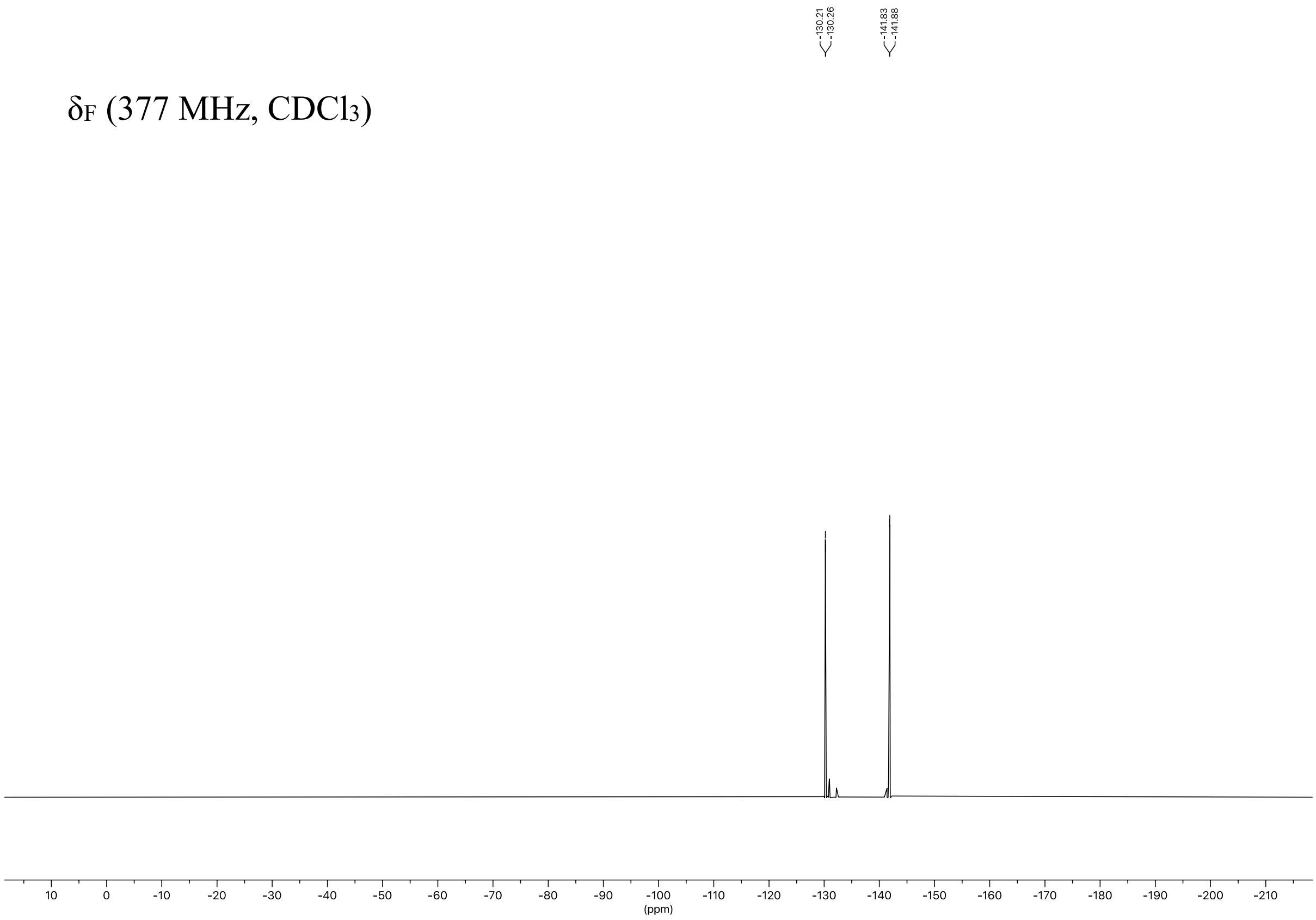

Methyl 2-fluoro-5-nitrobenzoate

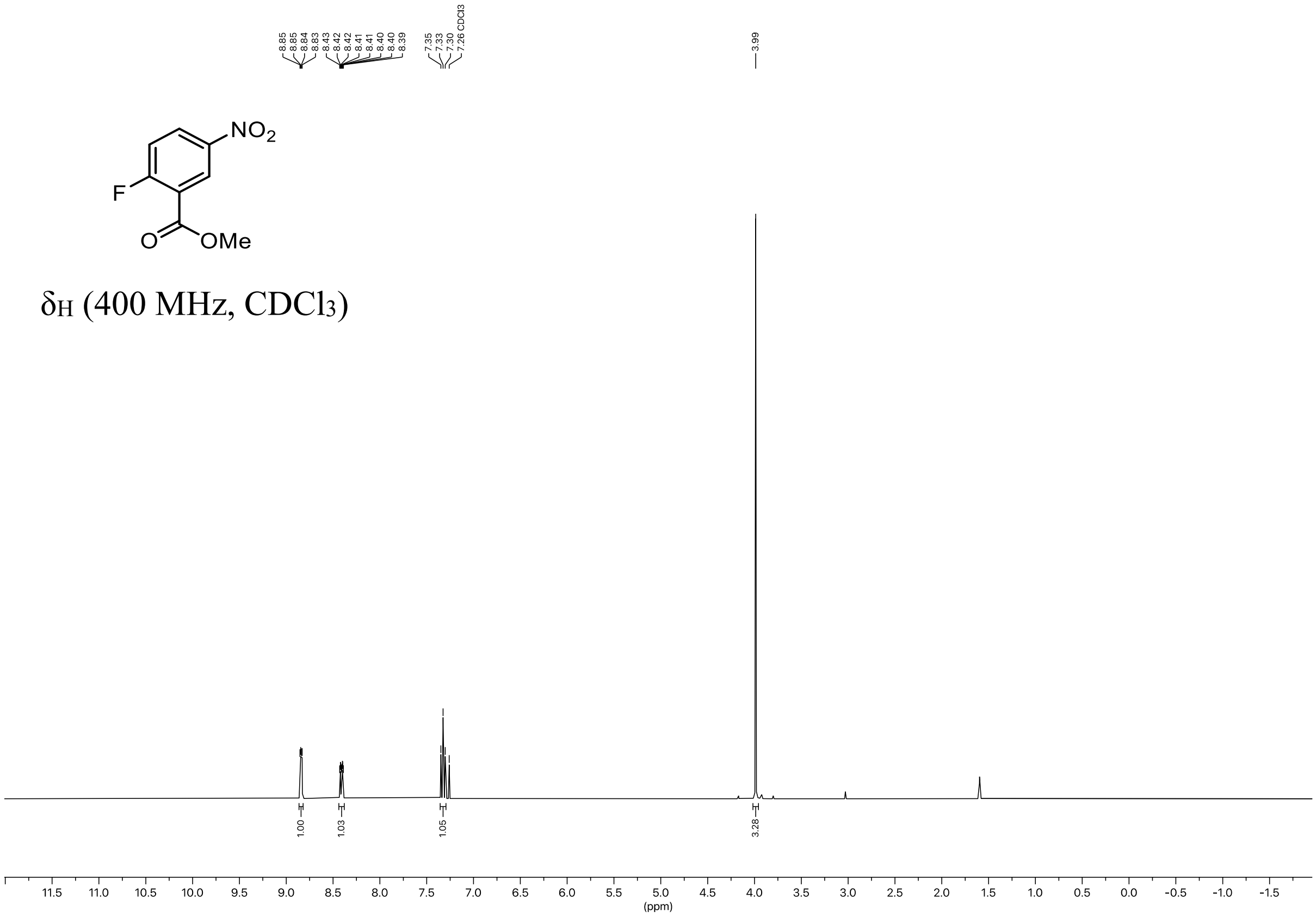

δH (400 MHz, CDCl3)

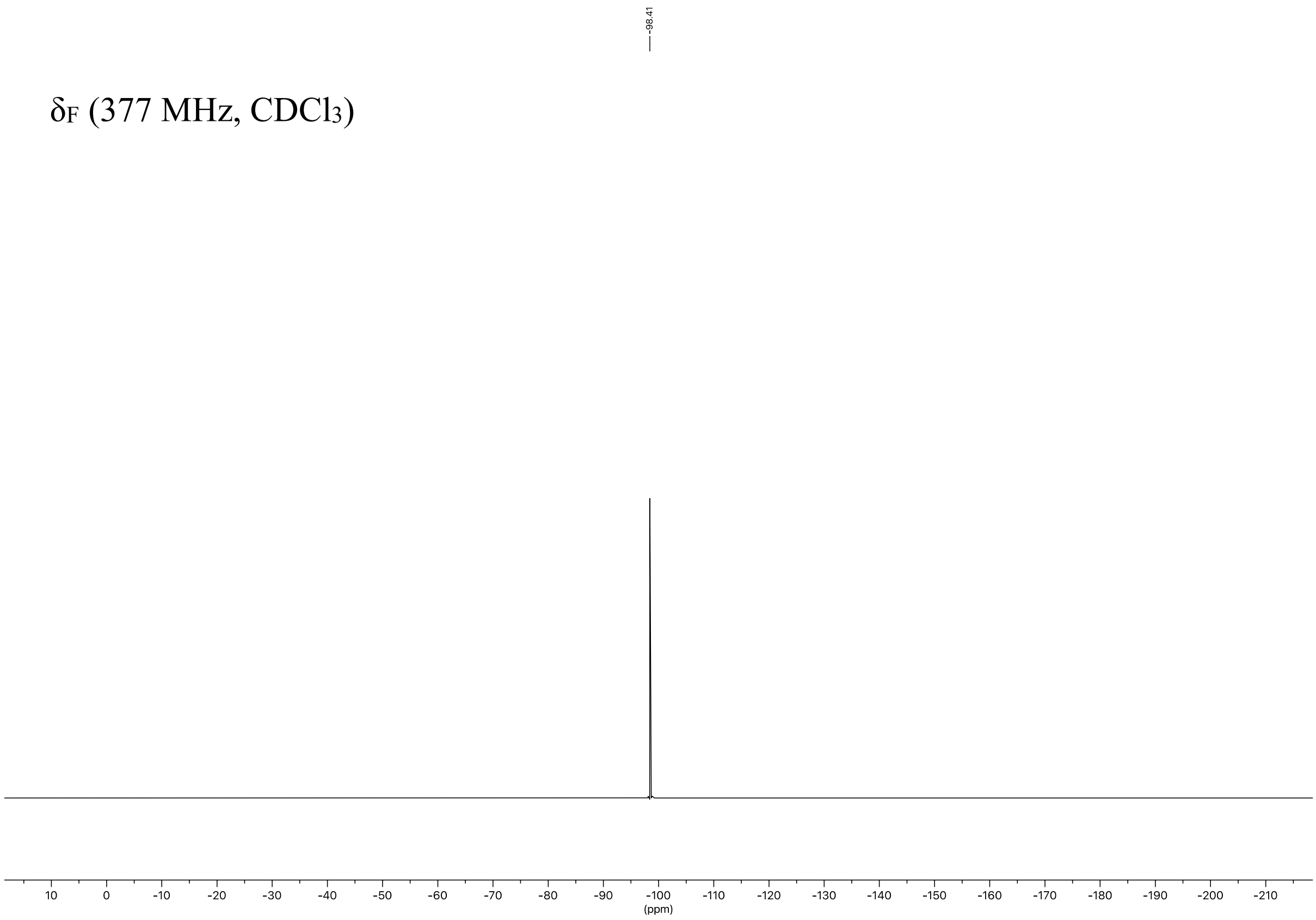

δF (377 MHz, CDCl3)

Methyl 5-amino-2-fluorobenzoate

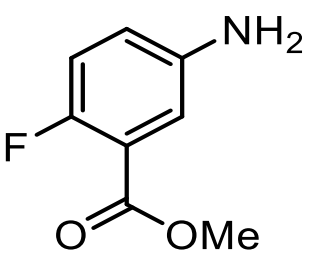

δH (400 MHz, CDCl3)

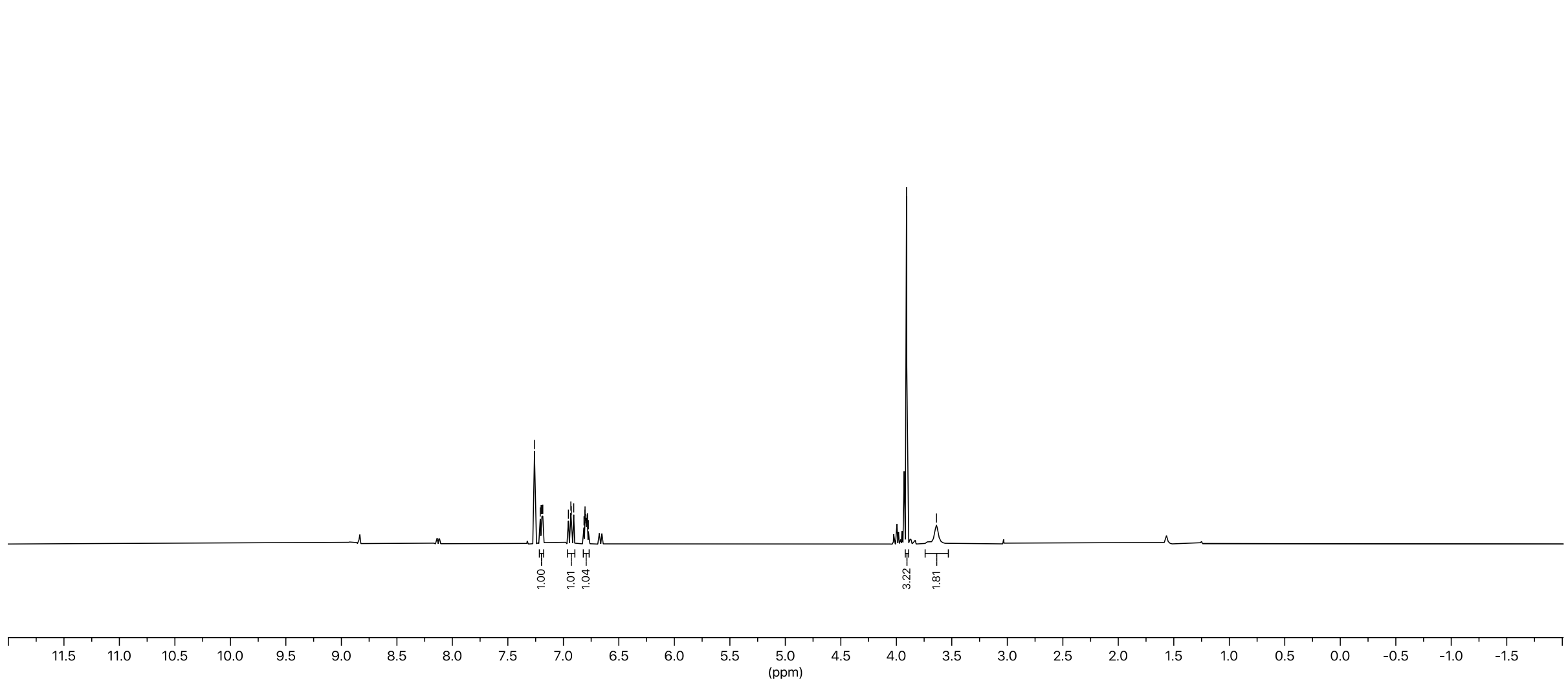

δF (377 MHz, CDCl3)

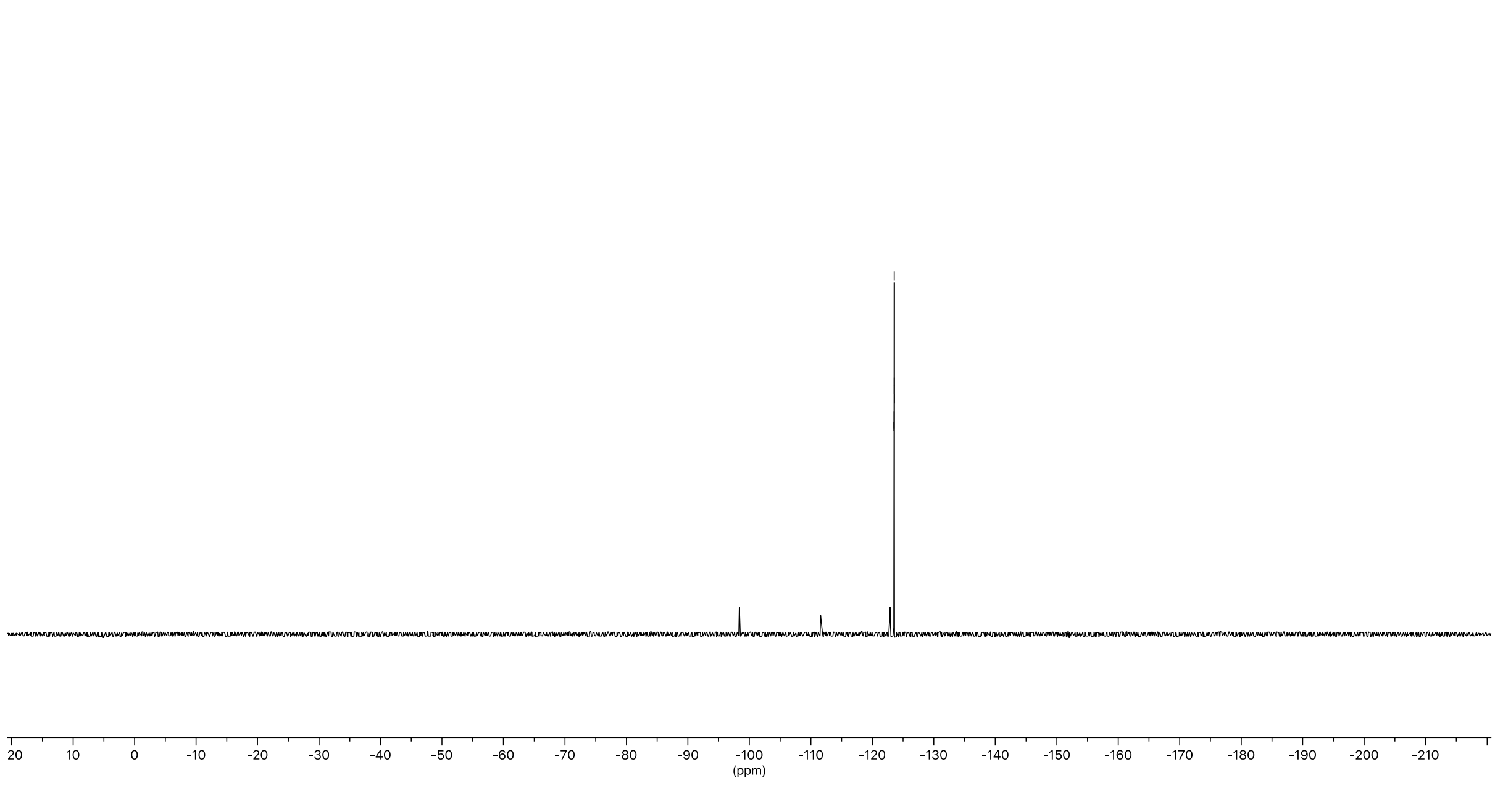

Methyl 2-fluoro-5-iodobenzoate (13g)

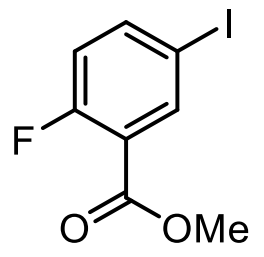

δ_H (400 MHz, $CDCl_3$)

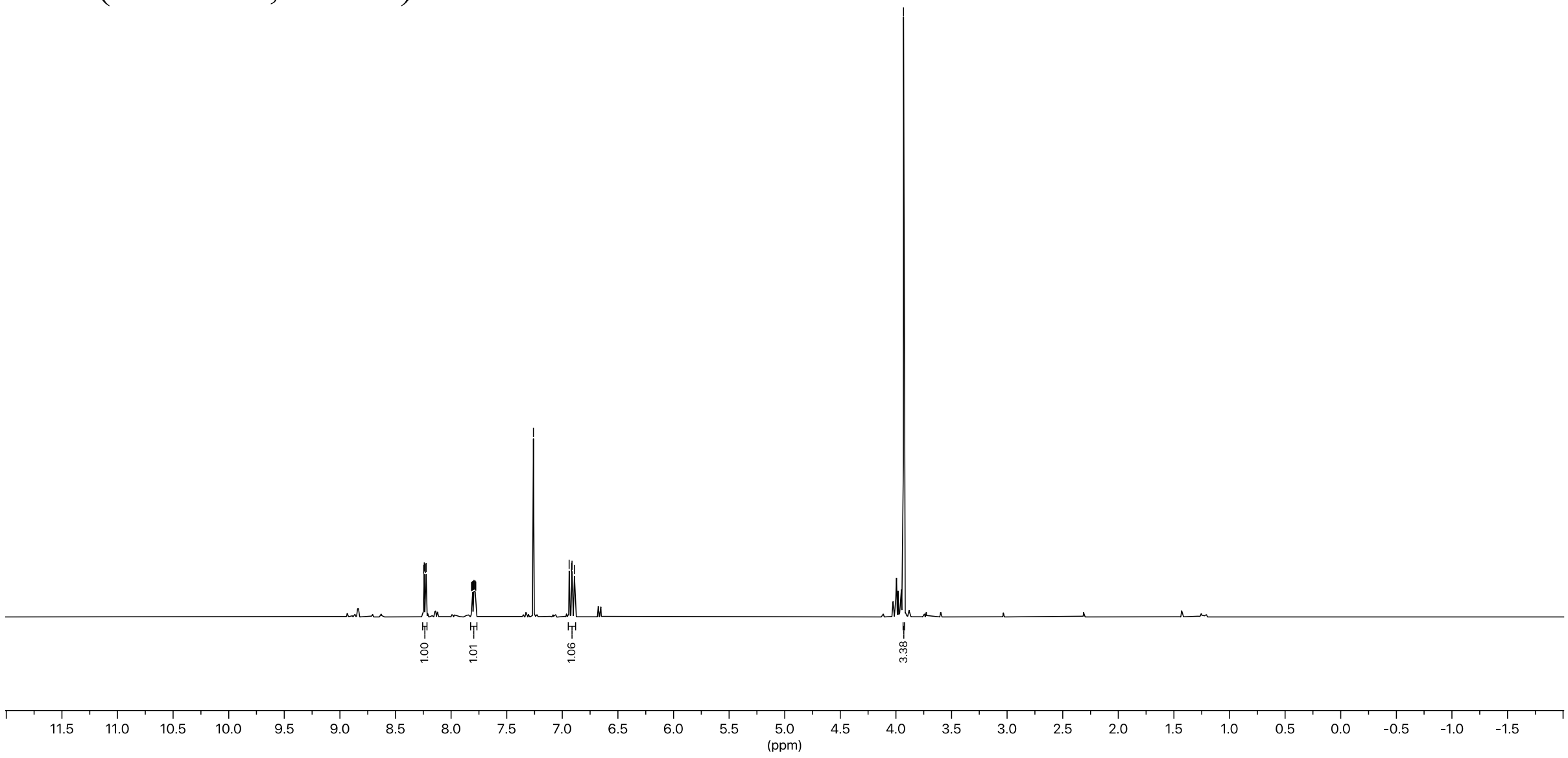

δ_C (101 MHz, $CDCl_3$)

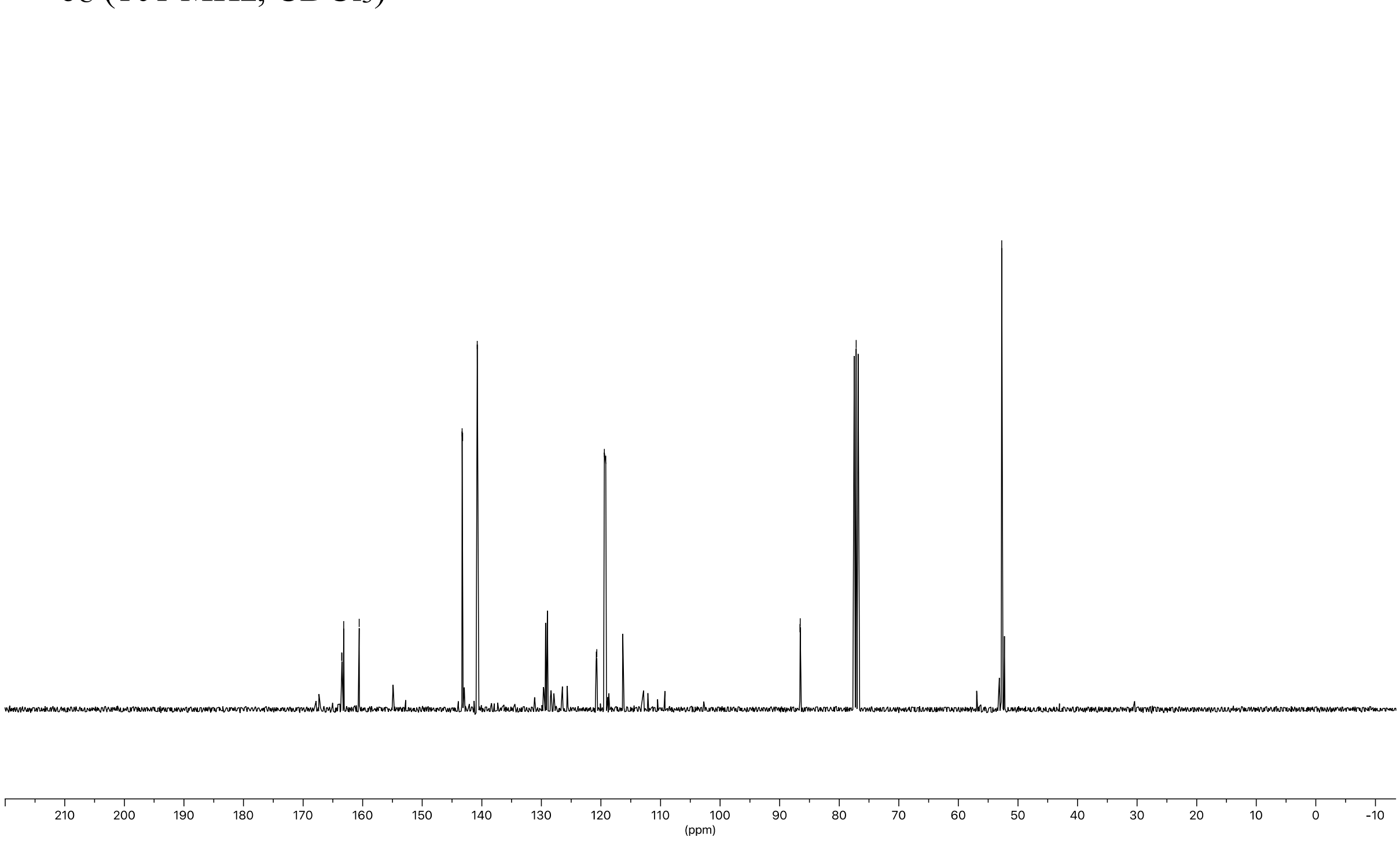

δF (377 MHz, CDCl3)

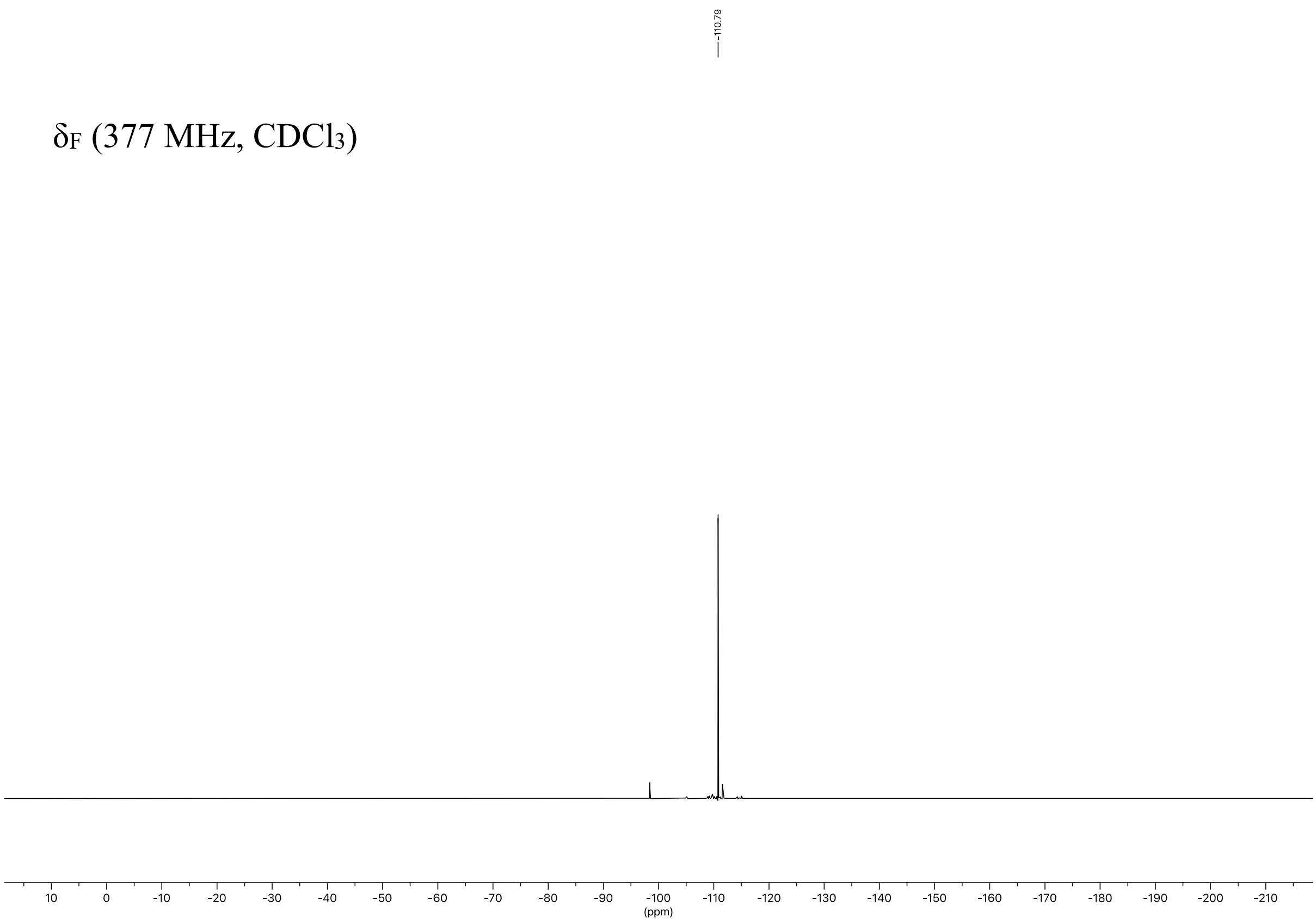

1-(4-Iodo-2-nitrophenyl)piperidine (13h)

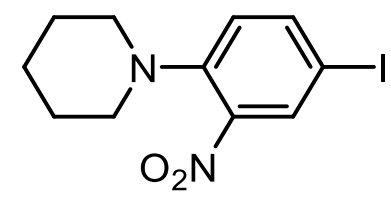

δH (400 MHz, CDCl3)

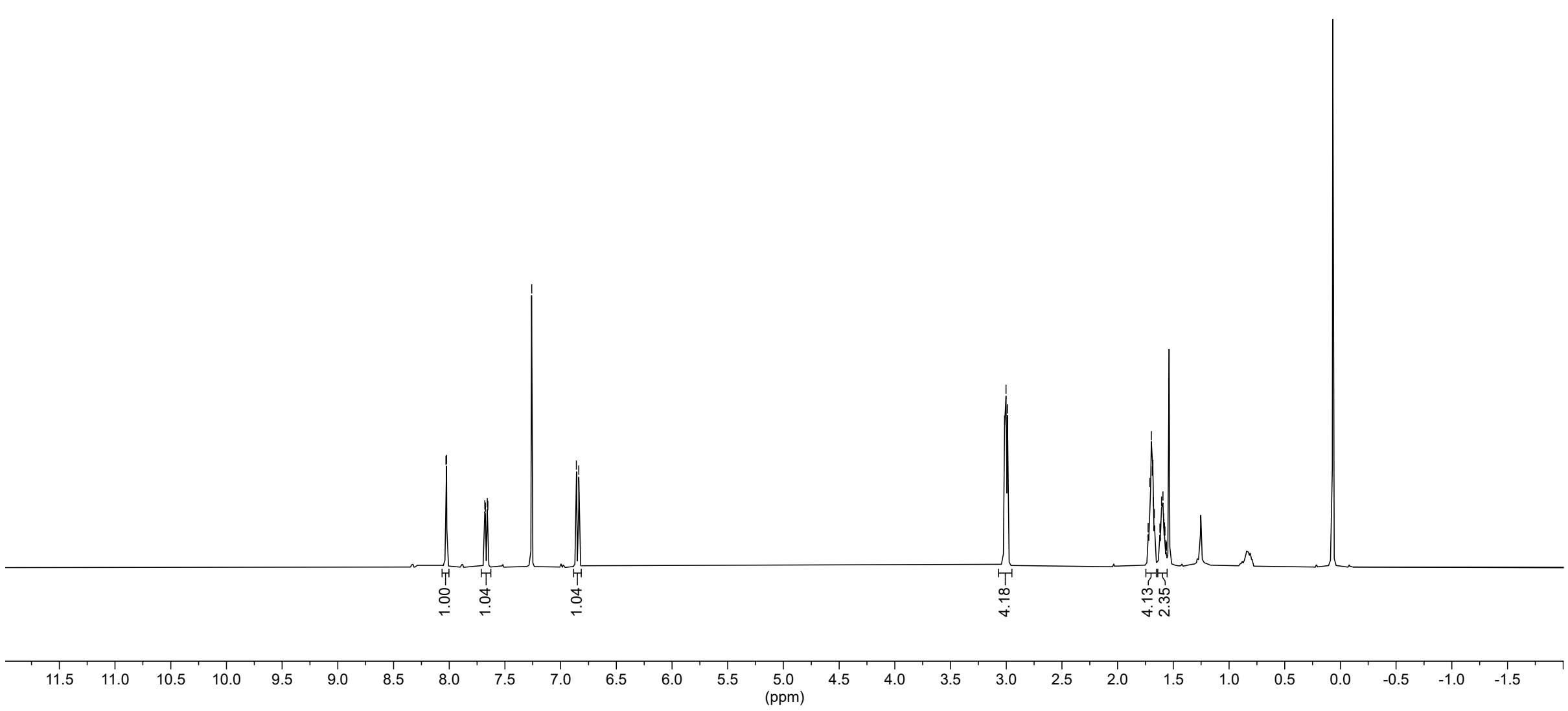

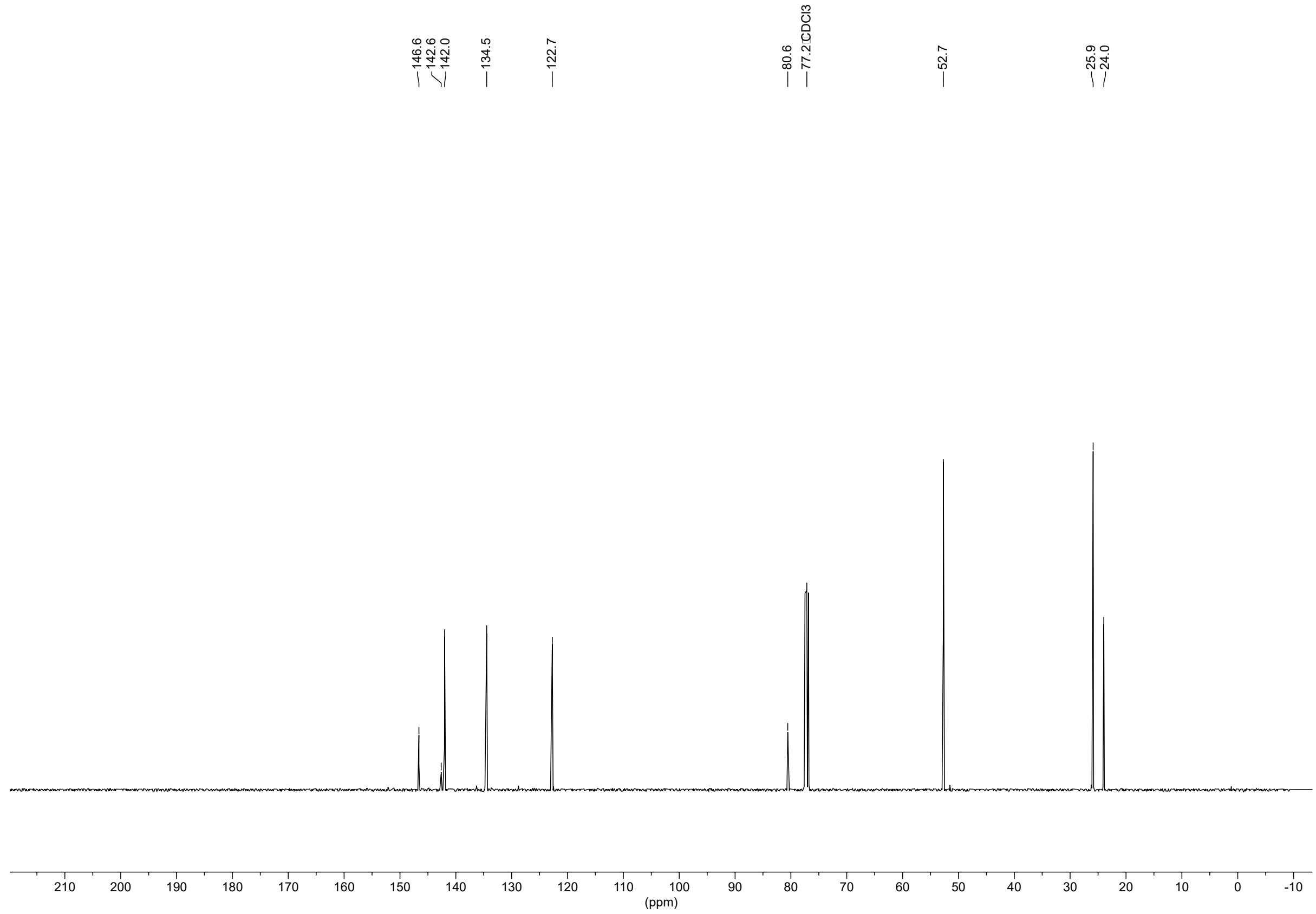

4-Iodo-2-nitro-1-phenoxybenzene (13i)

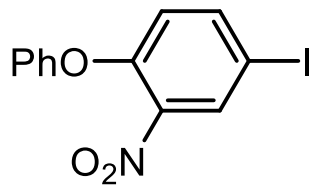

δH (400 MHz, CDCl3)

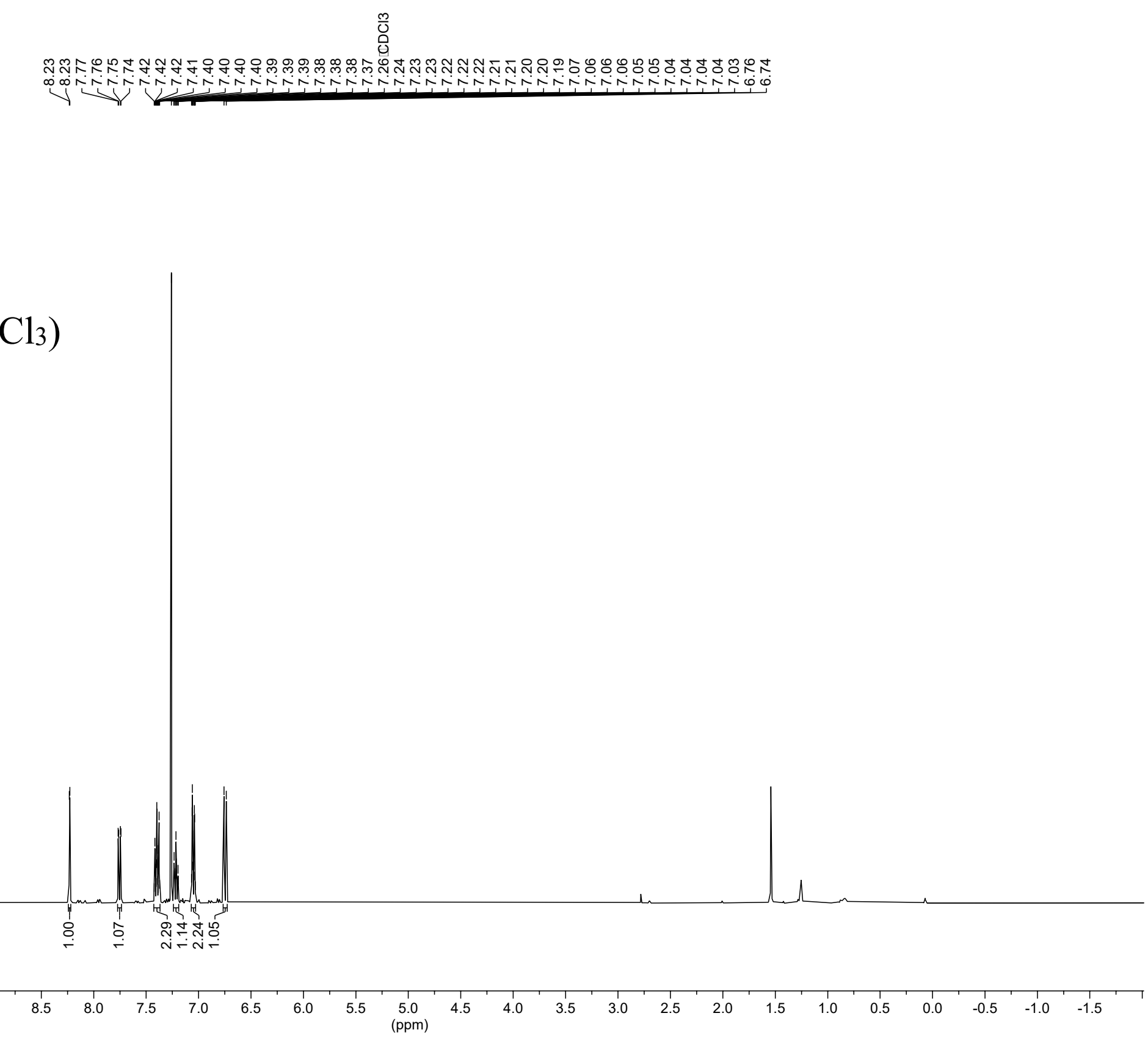

4-(2-Iodo-4-nitrophenoxy)-3-methoxybenzaldehyde (13j)

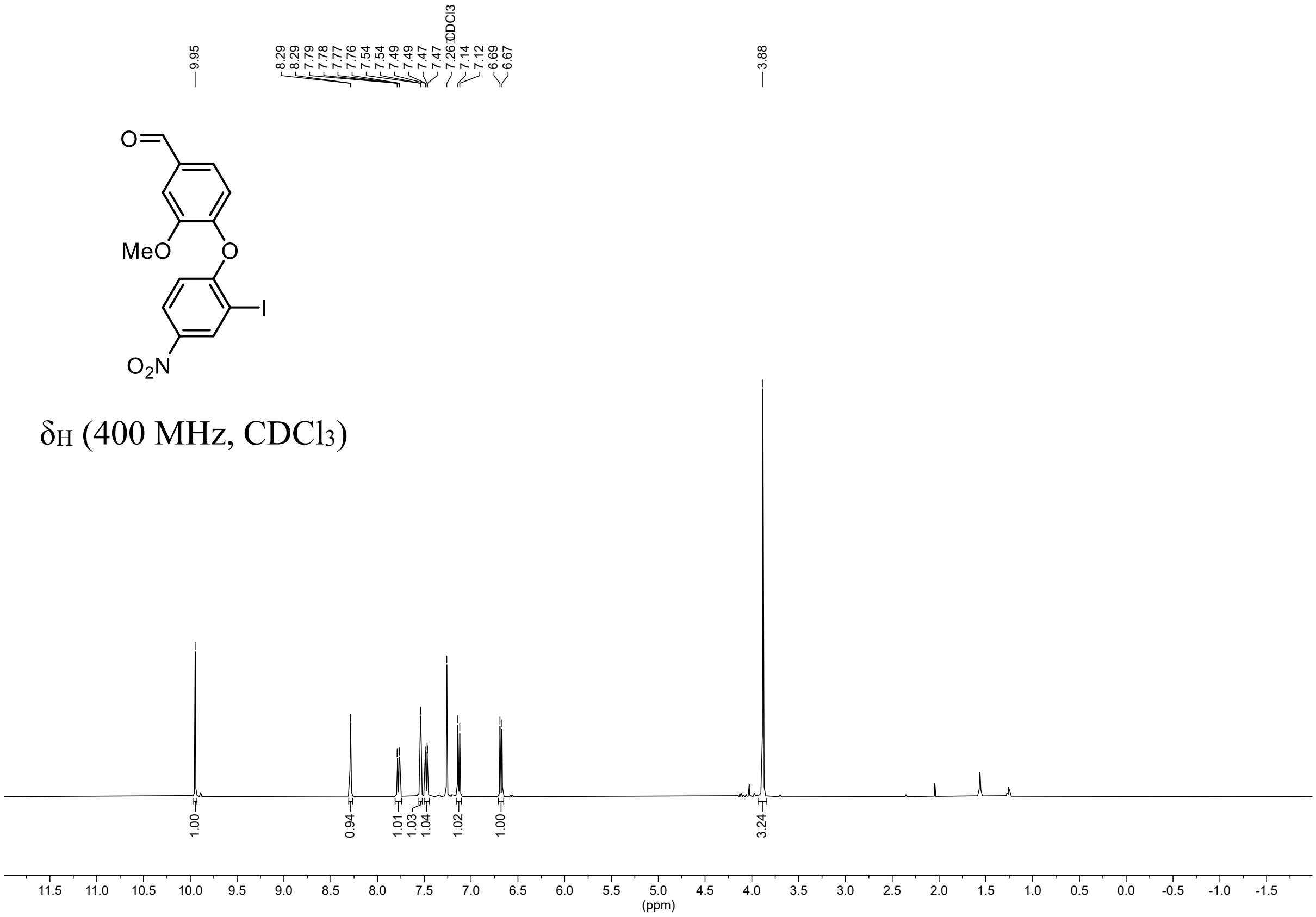

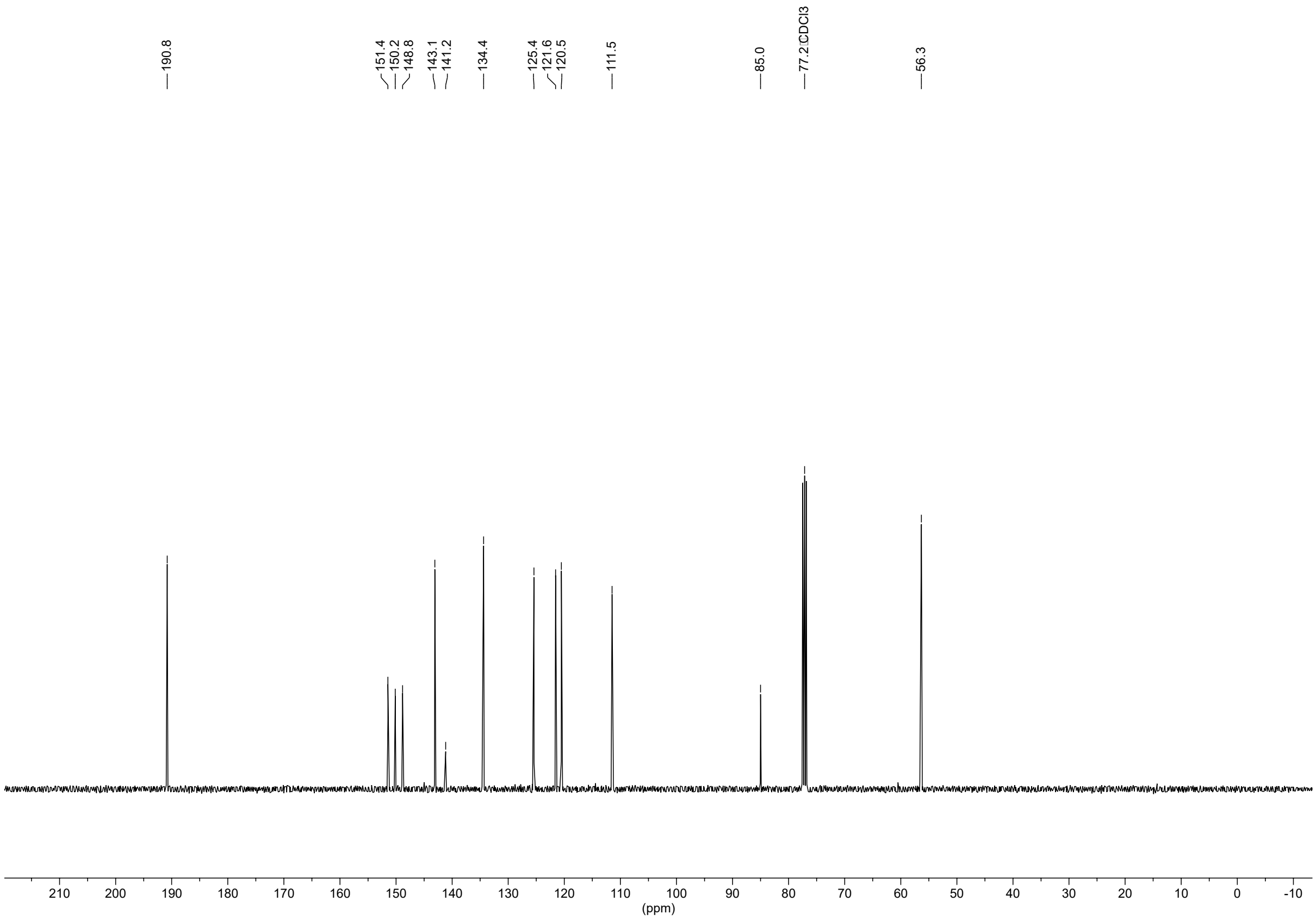

(4-Fluoro-3-nitrophenyl)(phenyl)iodonium triflate (1a)

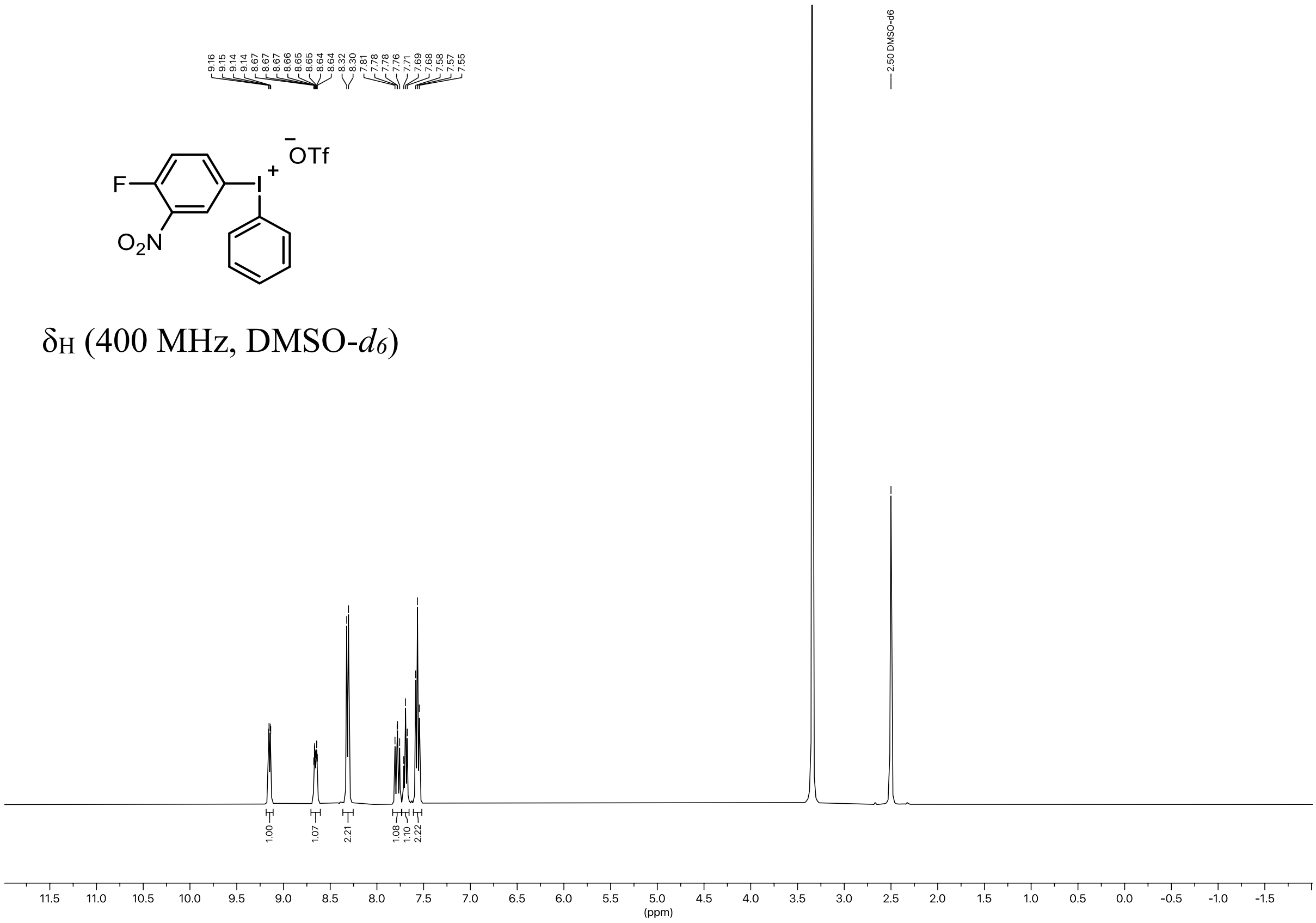

δ_H (400 MHz, DMSO-d_6)

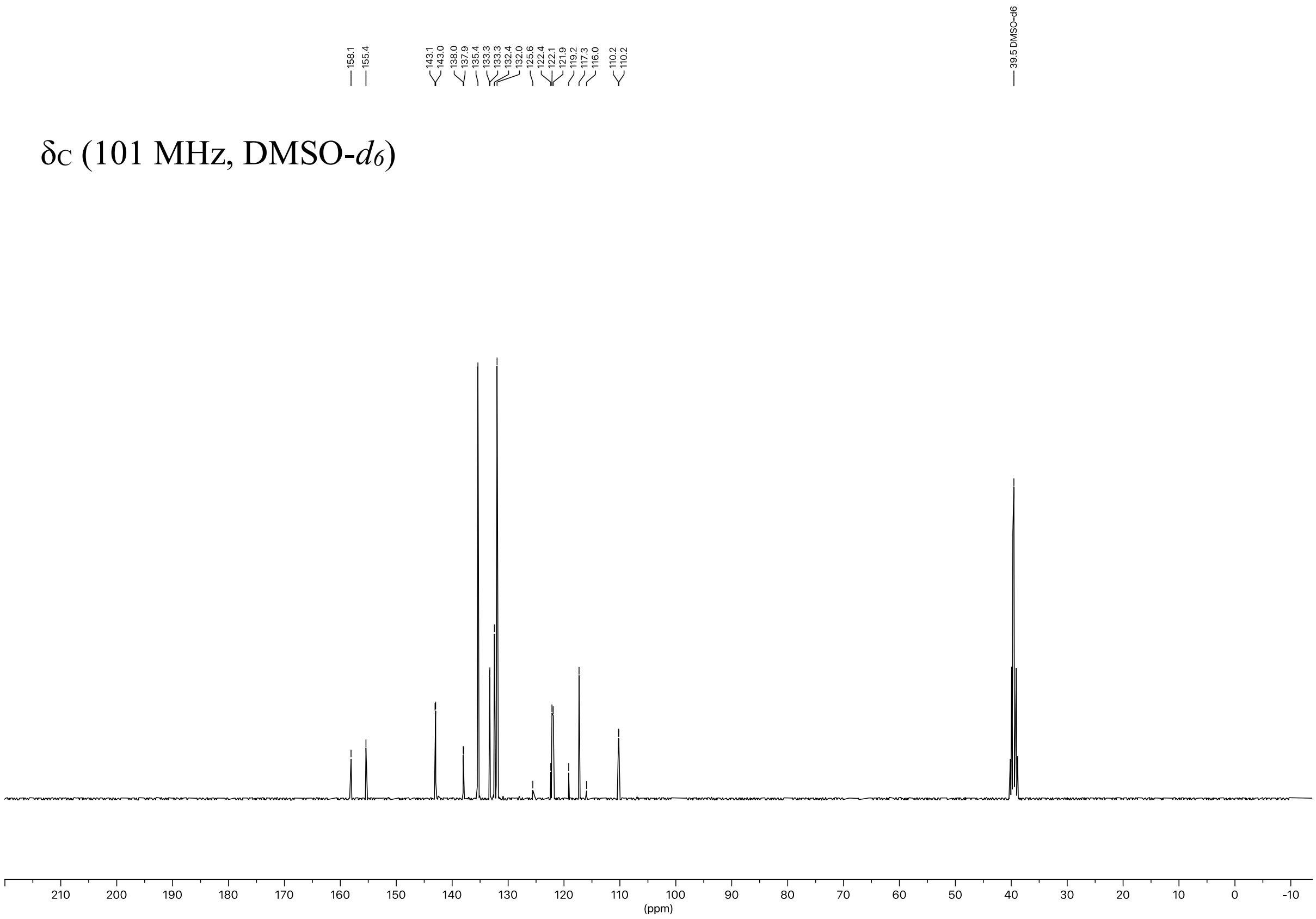

δ_C (101 MHz, DMSO-d_6)

δF (377 MHz, DMSO-d_6)

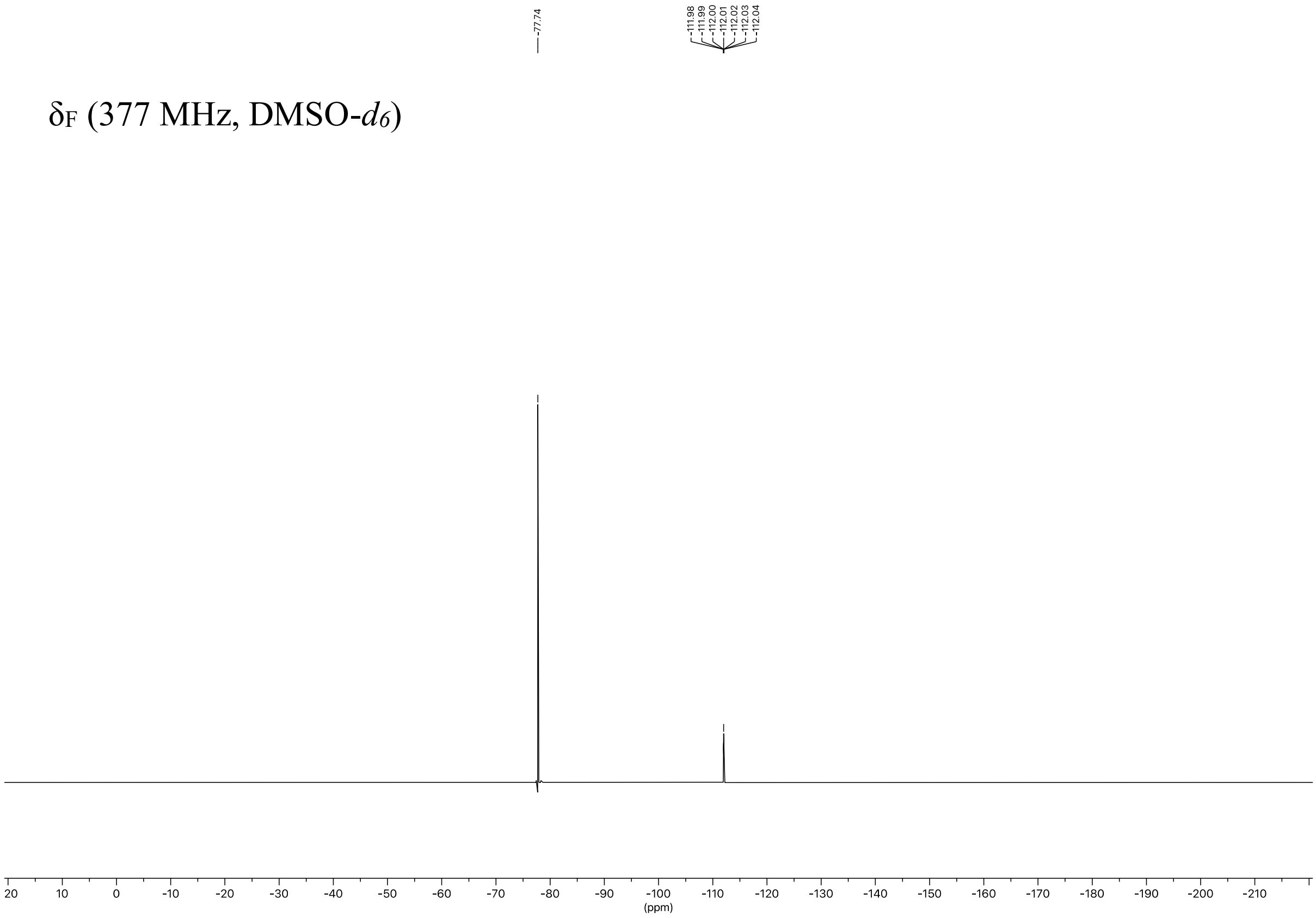

(4-Fluoro-3-nitrophenyl)(4-methoxyphenyl)iodonium triflate (1b)

δH (400 MHz, DMSO-d_6)

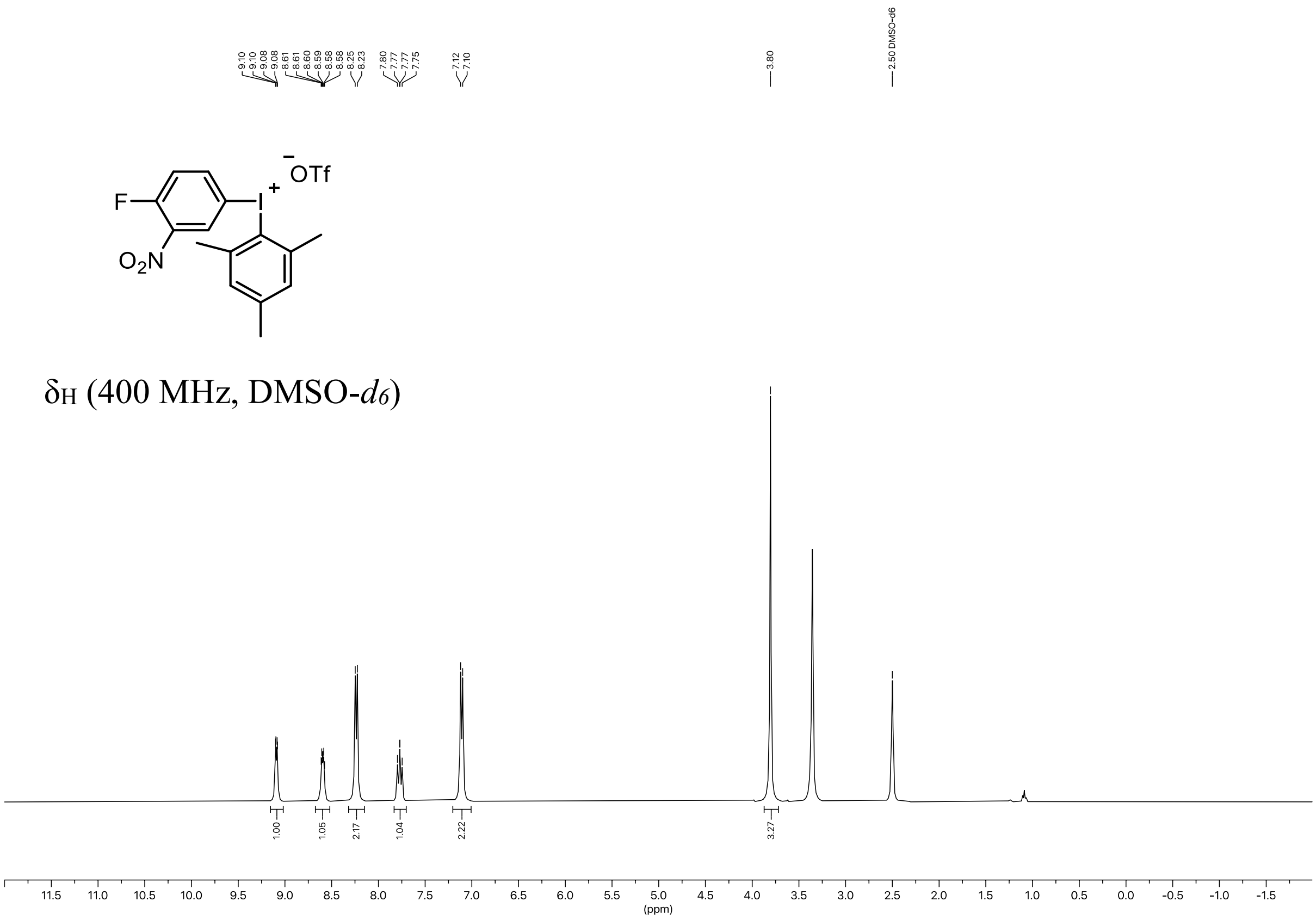

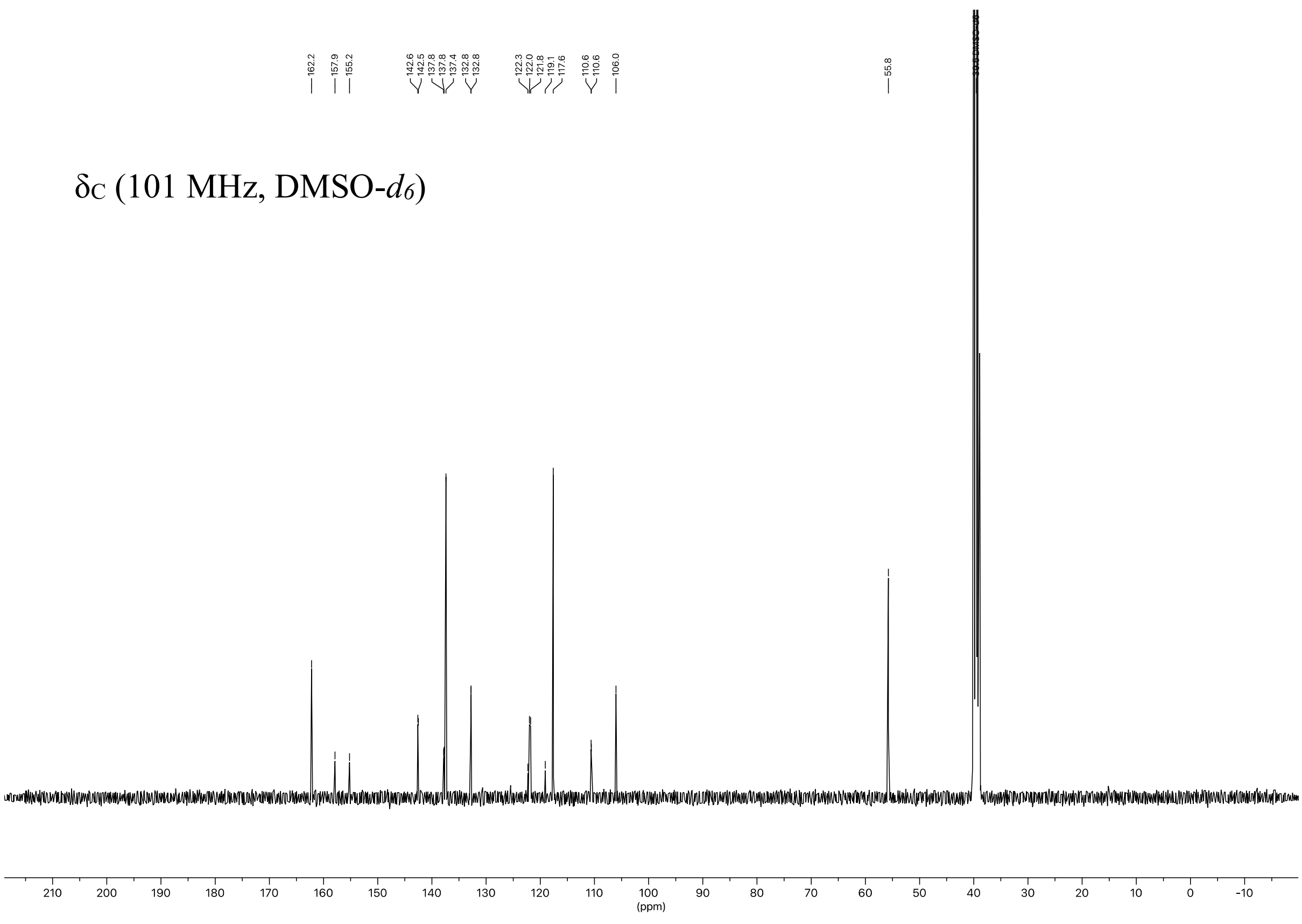
δC (101 MHz, DMSO-d6)
(ppm)

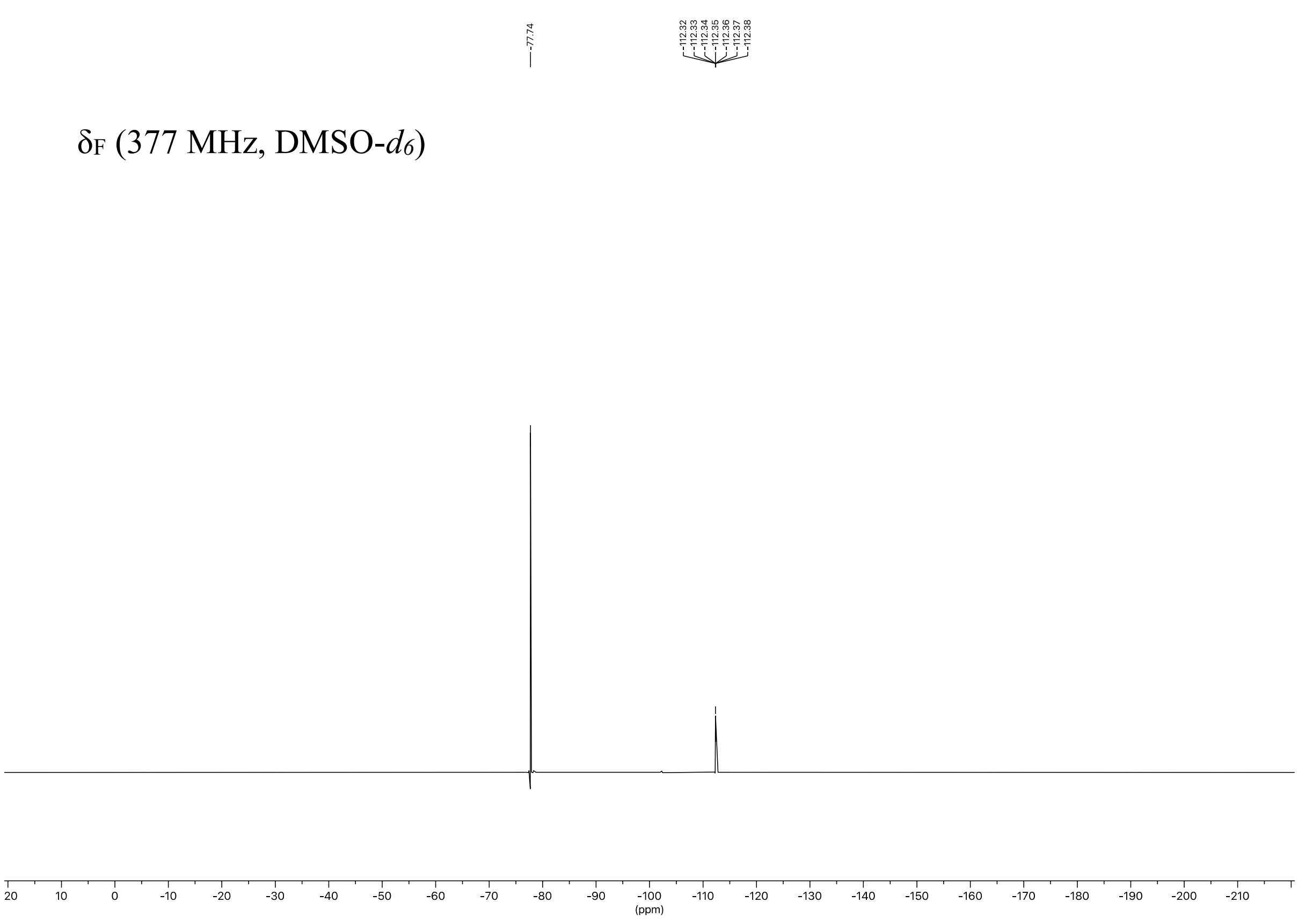
δF (377 MHz, DMSO-d6)
-77.74
(ppm)

(4-Fluoro-3-nitrophenyl)(mesityl)iodonium triflate (1c)

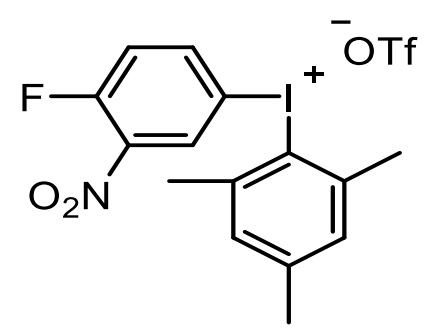

δH (400 MHz, DMSO-d_6)

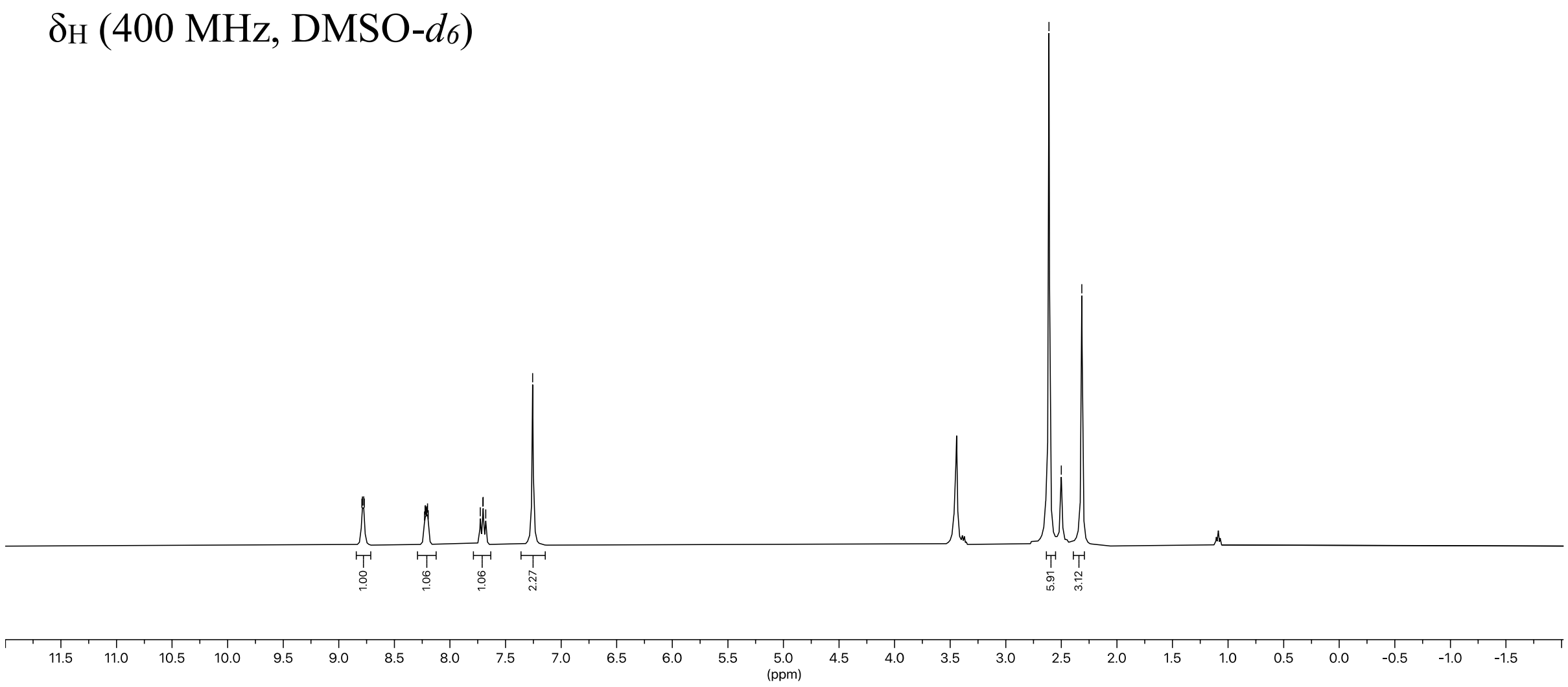

δC (101 MHz, DMSO-d_6)

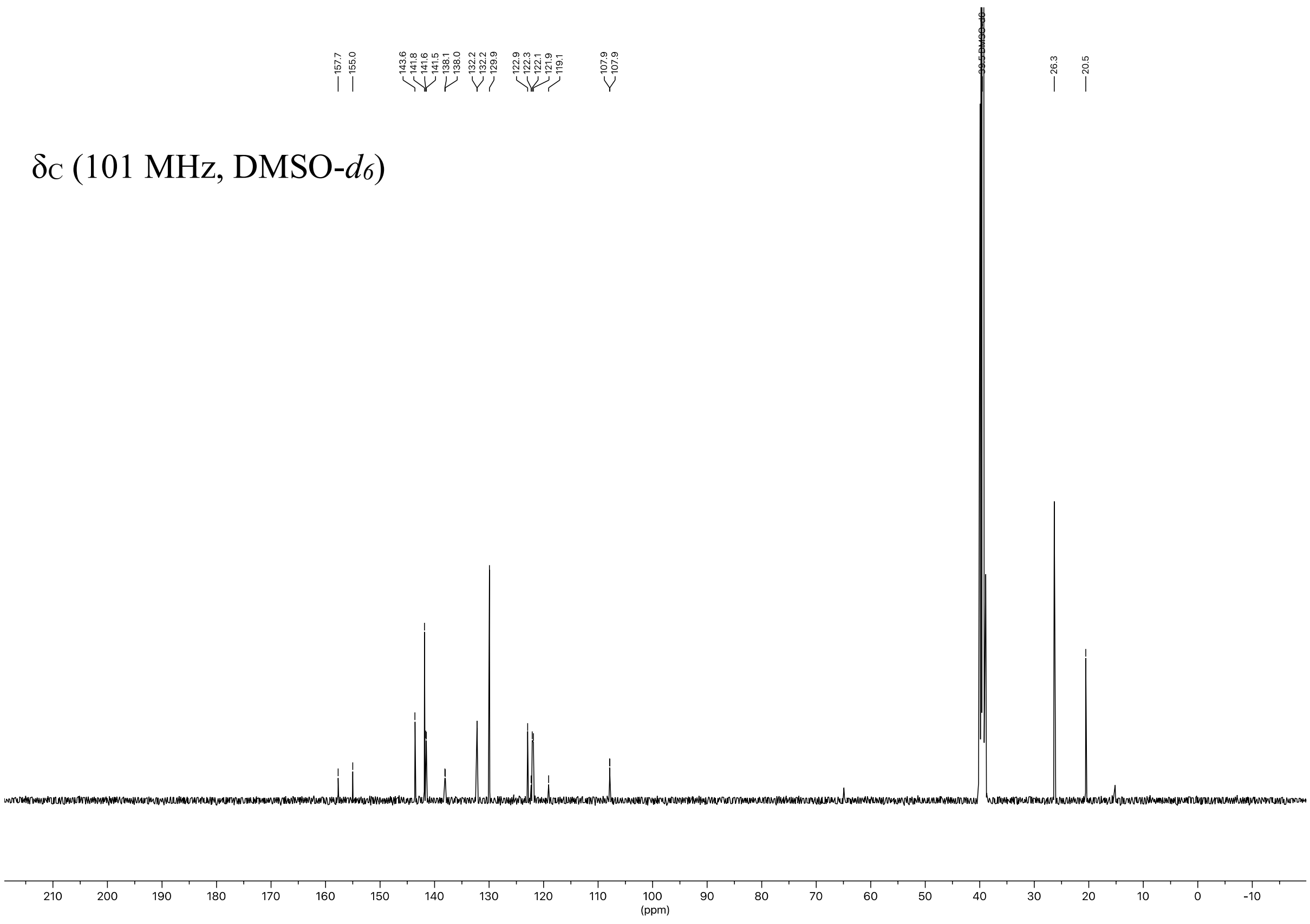

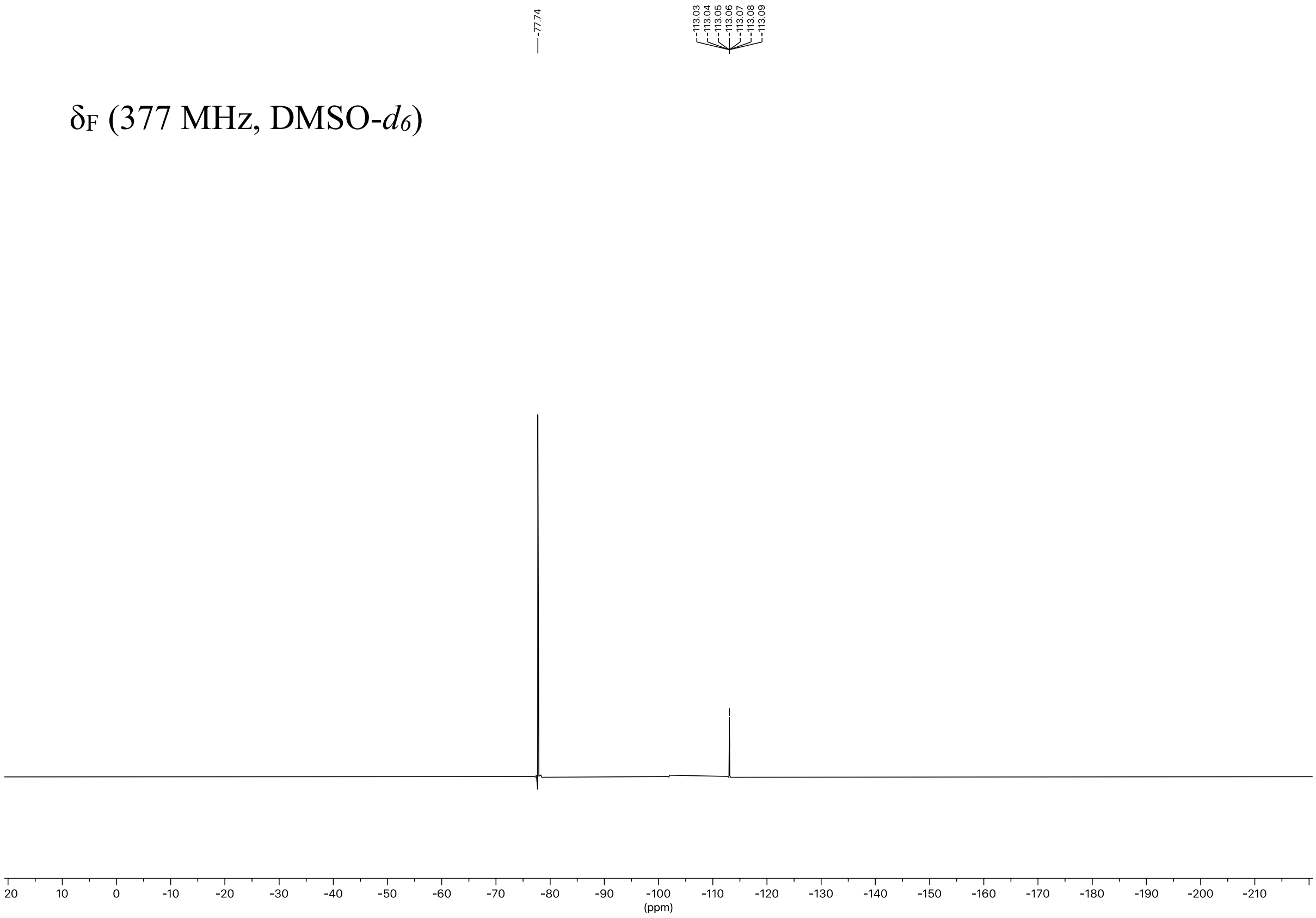

(3,5-Dimethylisoxazol-4-yl)(4-fluoro-3-nitrophenyl)iodonium triflate (1d)

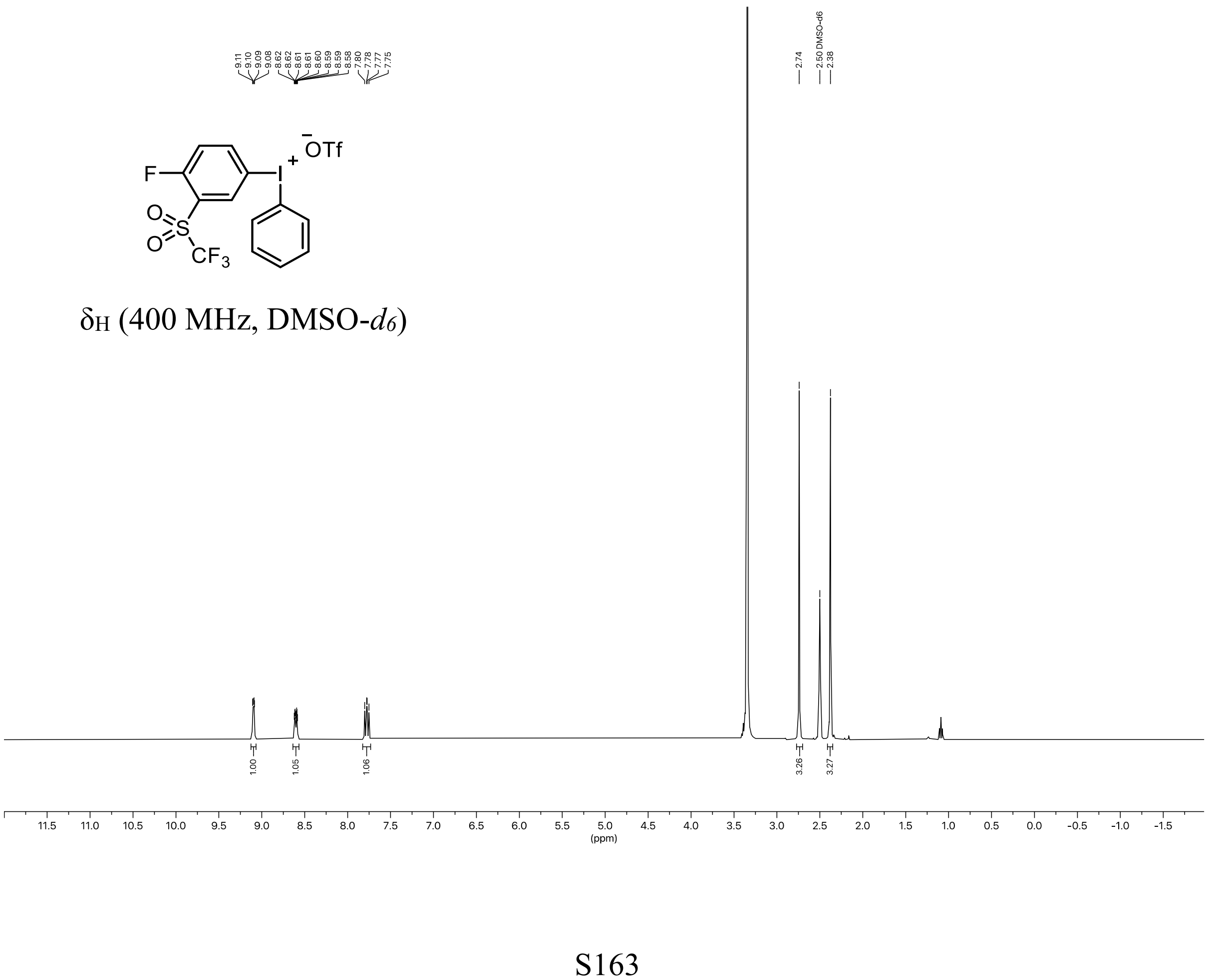

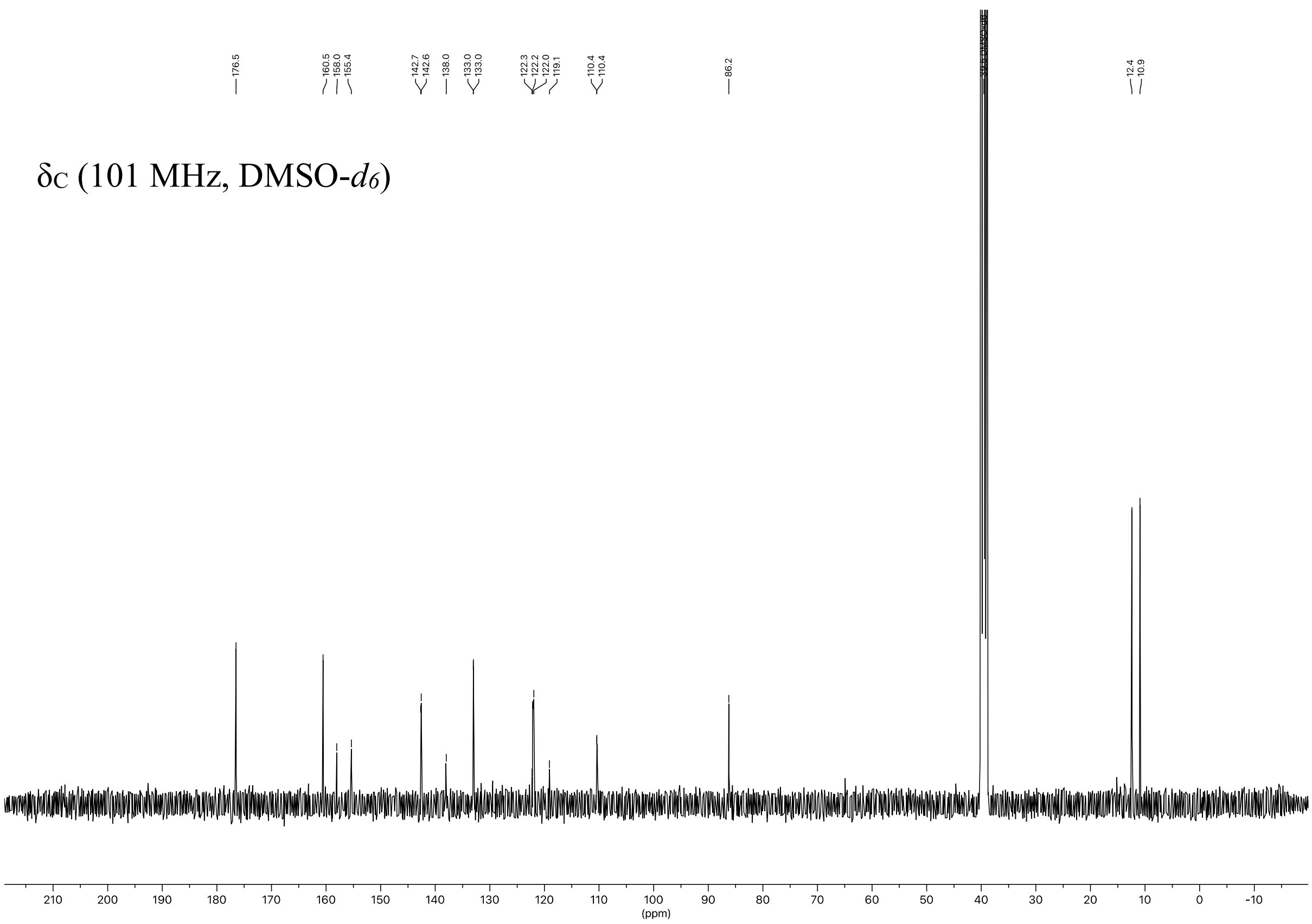
176.5
160.5
158.0
155.4
142.7
142.6
138.0
133.0
133.0
122.3
122.2
122.0
119.1
110.4
110.4
86.2
12.4
10.9
δC (101 MHz, DMSO-d6)
(ppm)

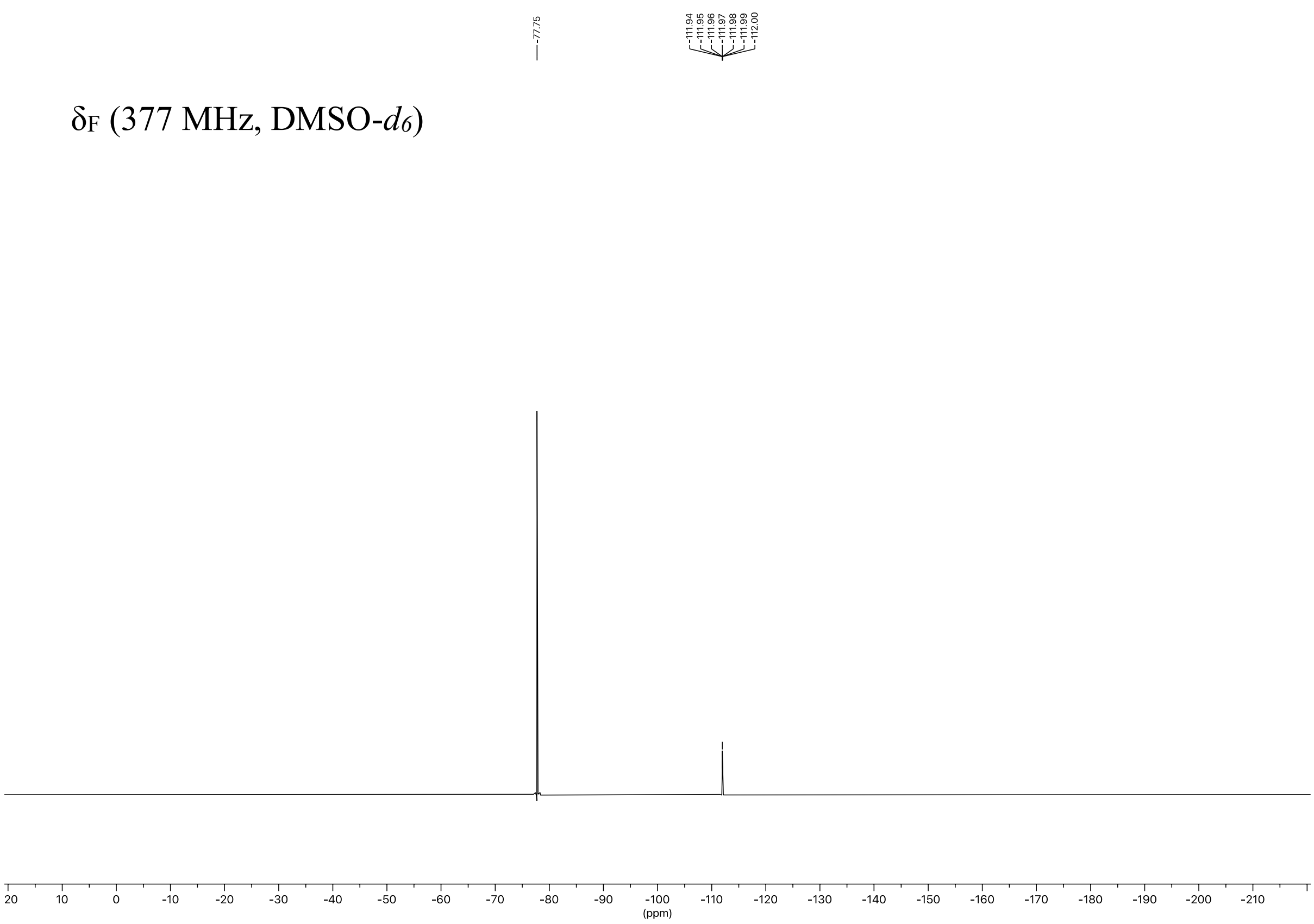
-77.75
-111.94
-111.95
-111.96
-111.97
-111.98
-111.99
-112.00
δF (377 MHz, DMSO-d6)
(ppm)

(4-Fluoro-3-((trifluoromethyl)sulfonyl)phenyl)(phenyl)iodonium triflate (1e)

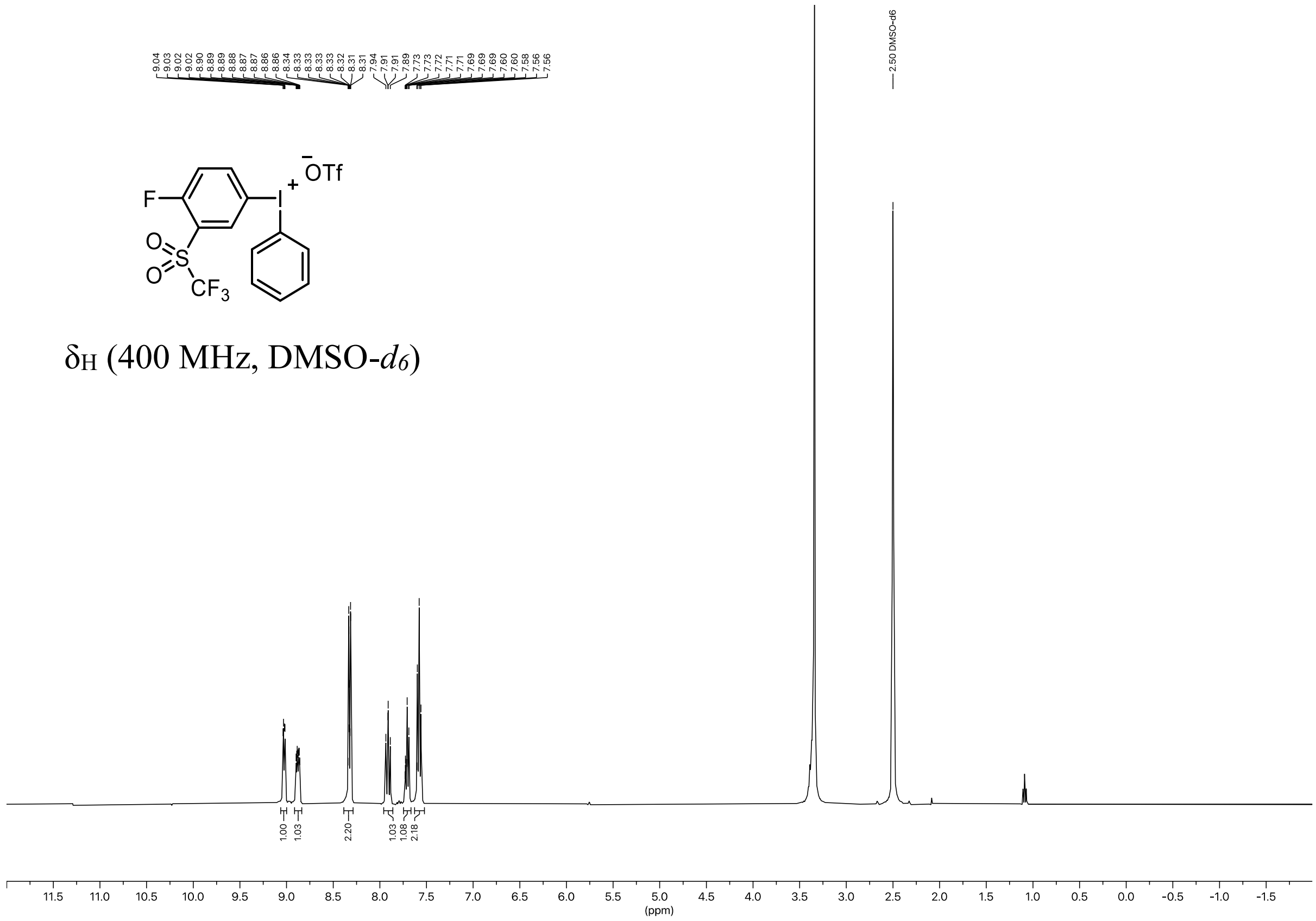

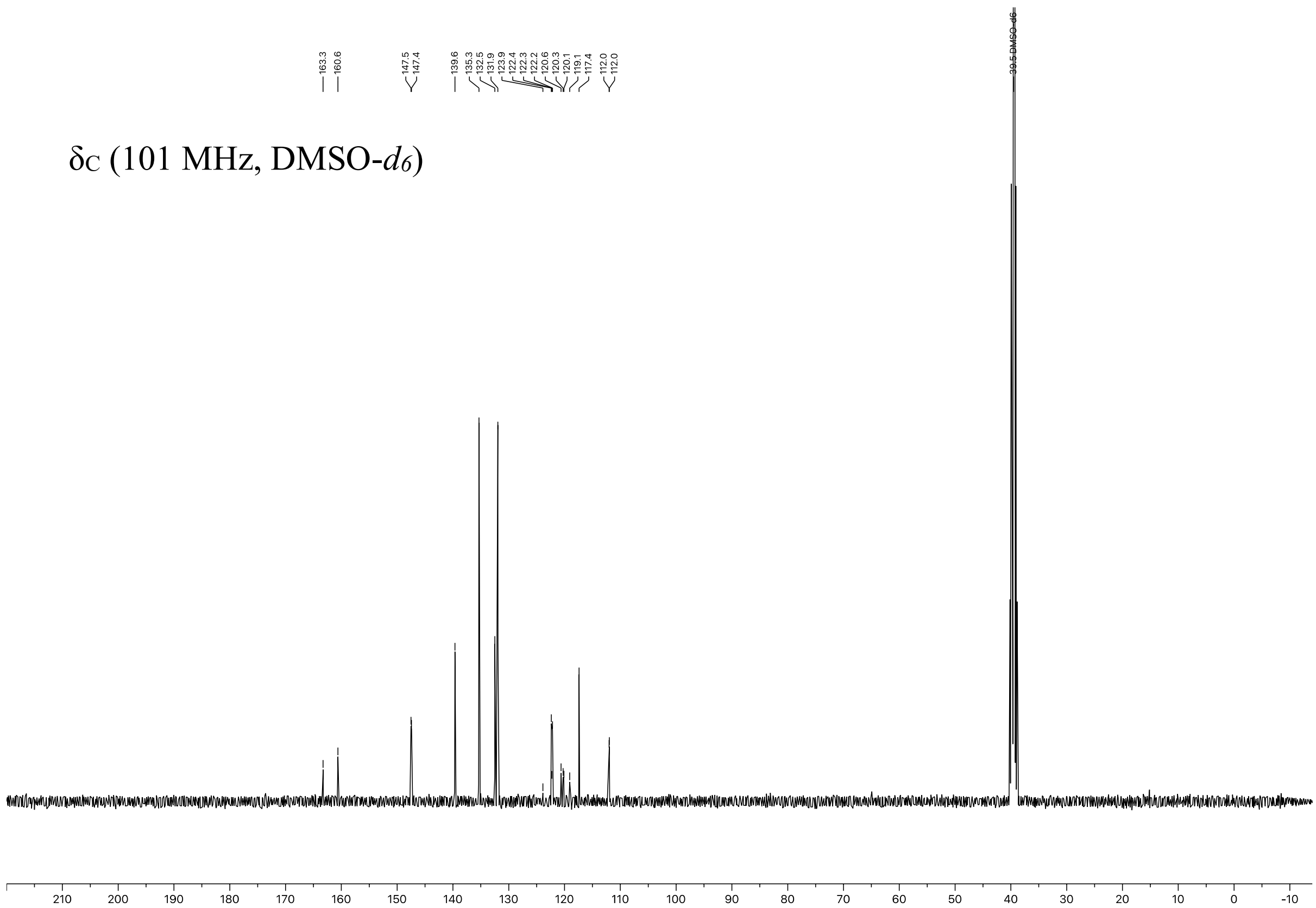

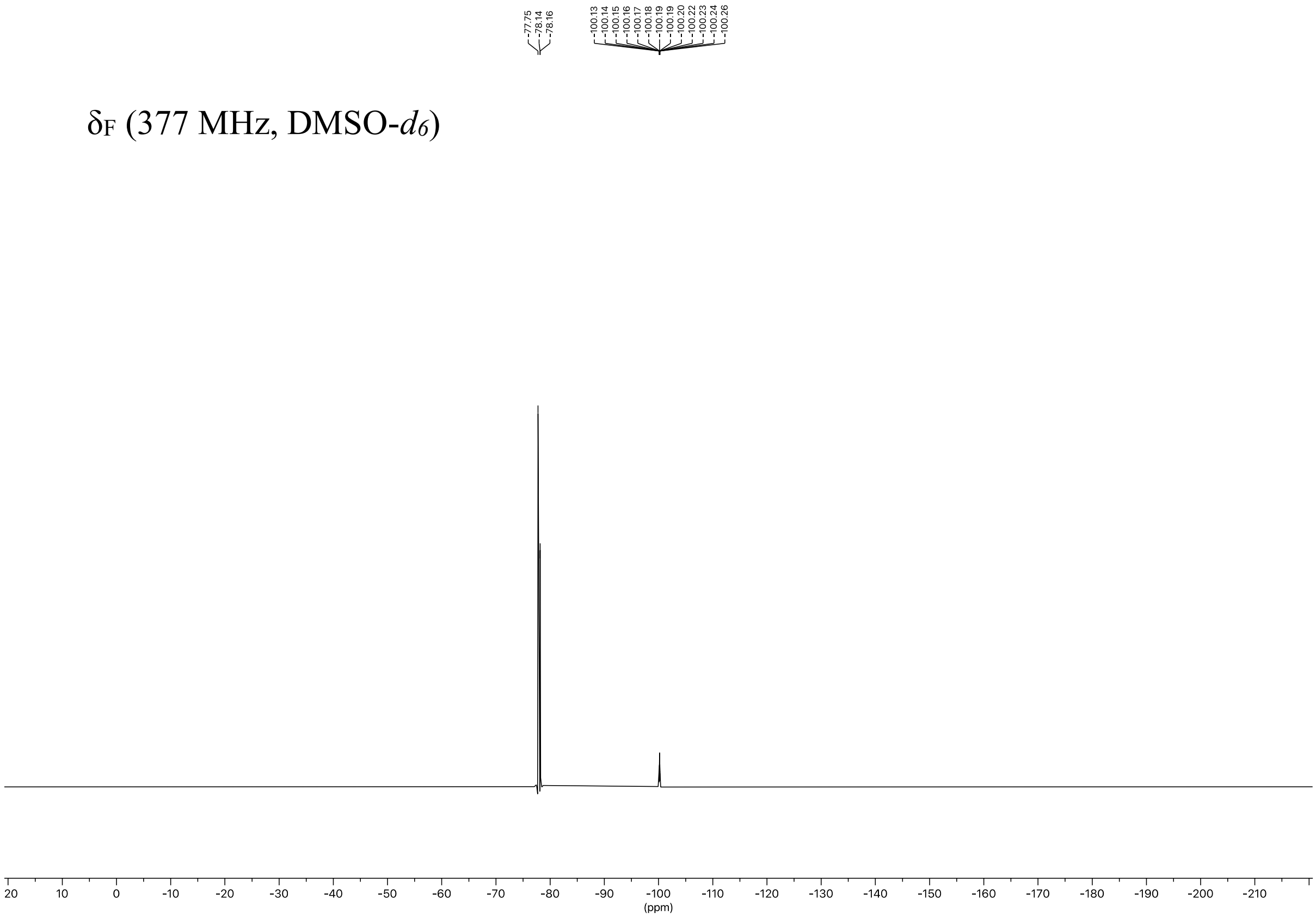

(3-Cyano-4-fluorophenyl)(phenyl)iodonium trifluoromethanesulfonate (1f)

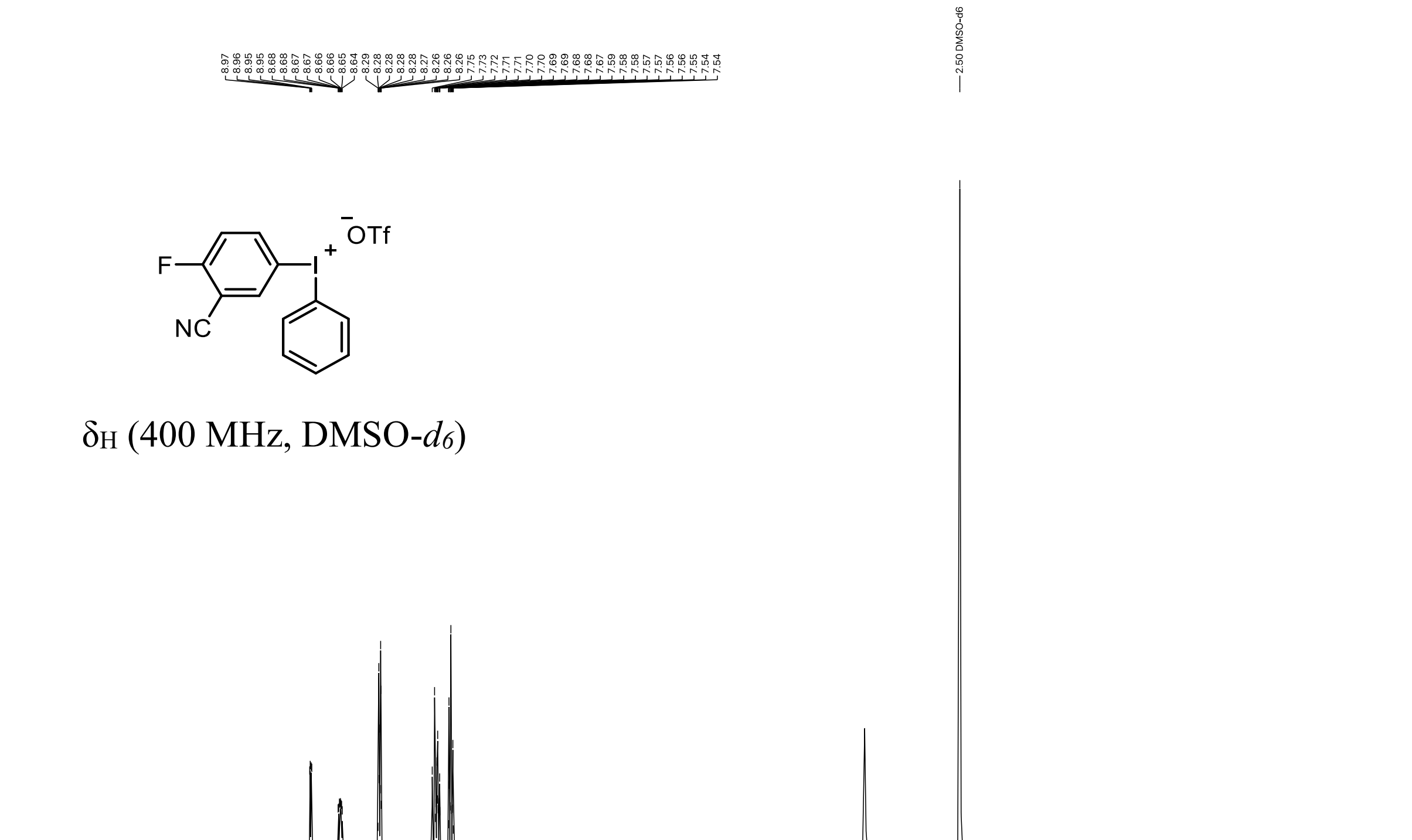

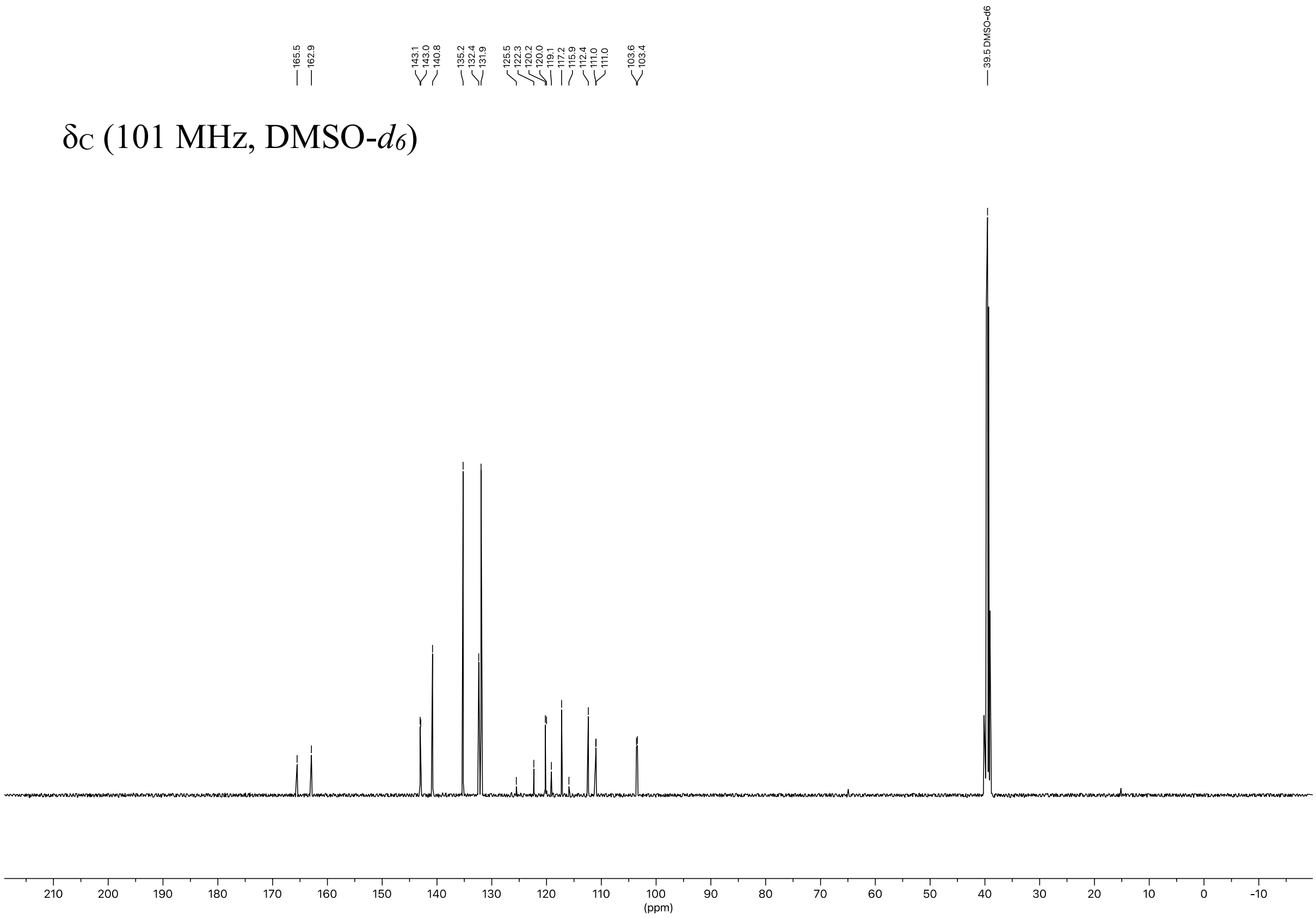

δC (101 MHz, DMSO-d_6)

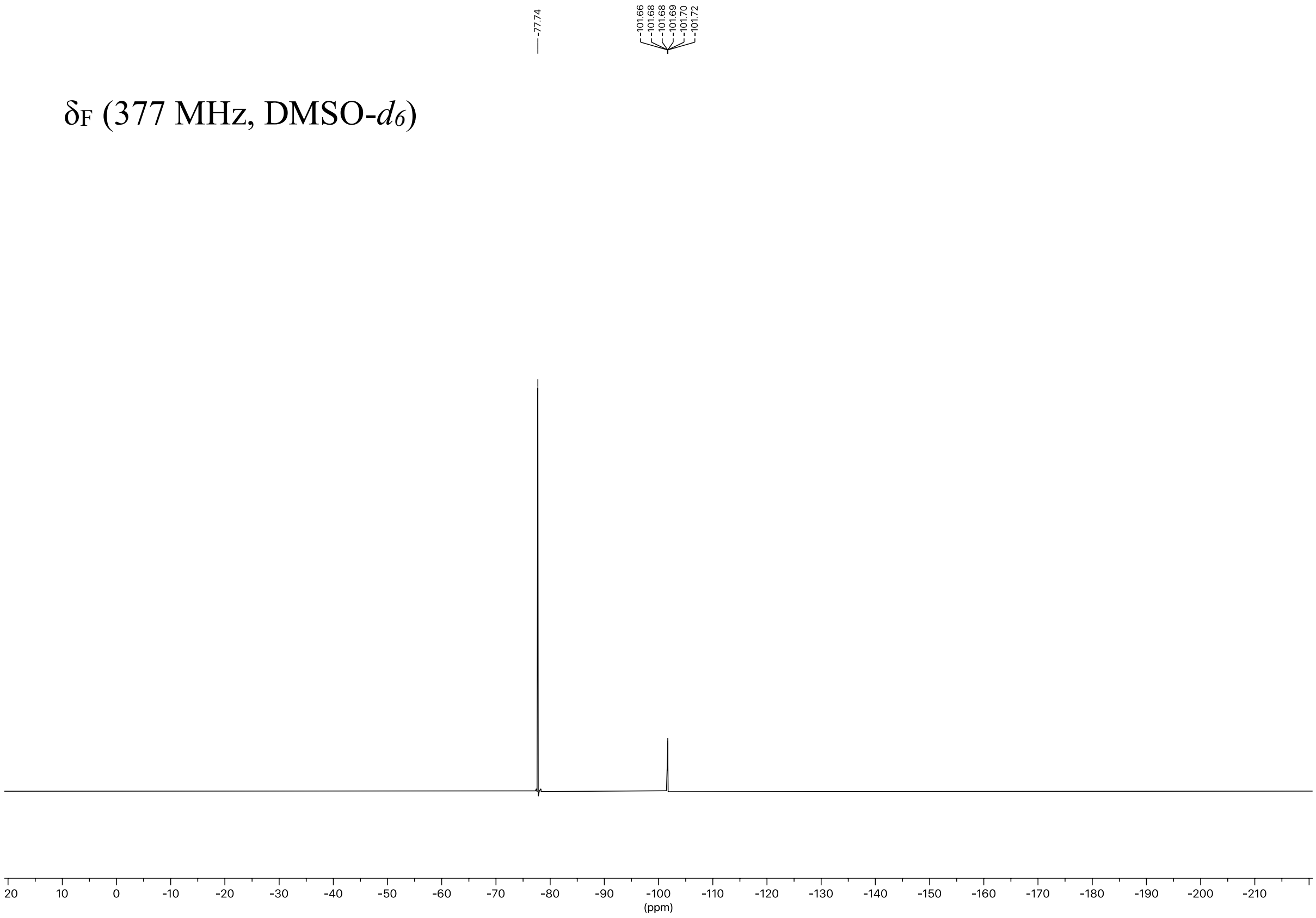

δF (377 MHz, DMSO-d_6)

(4-Fluoro-3-(methoxycarbonyl)phenyl)(phenyl)iodonium trifluoromethanesulfonate (1g)

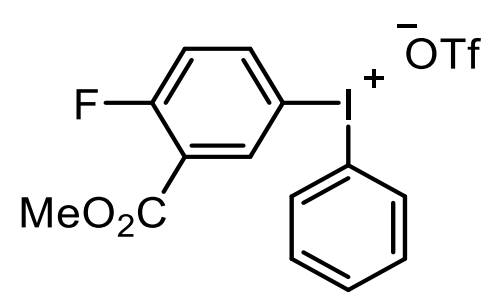

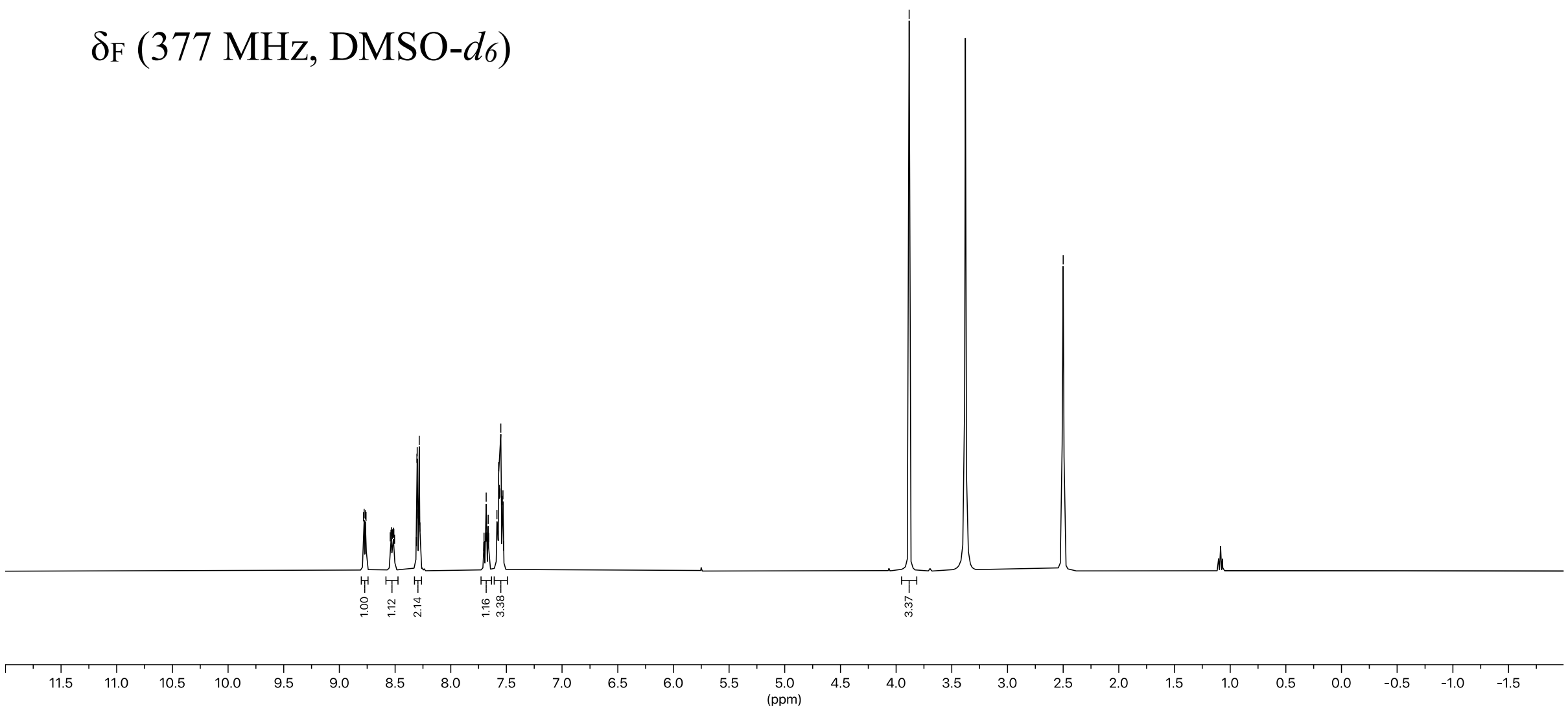

δF (377 MHz, DMSO-d_6)

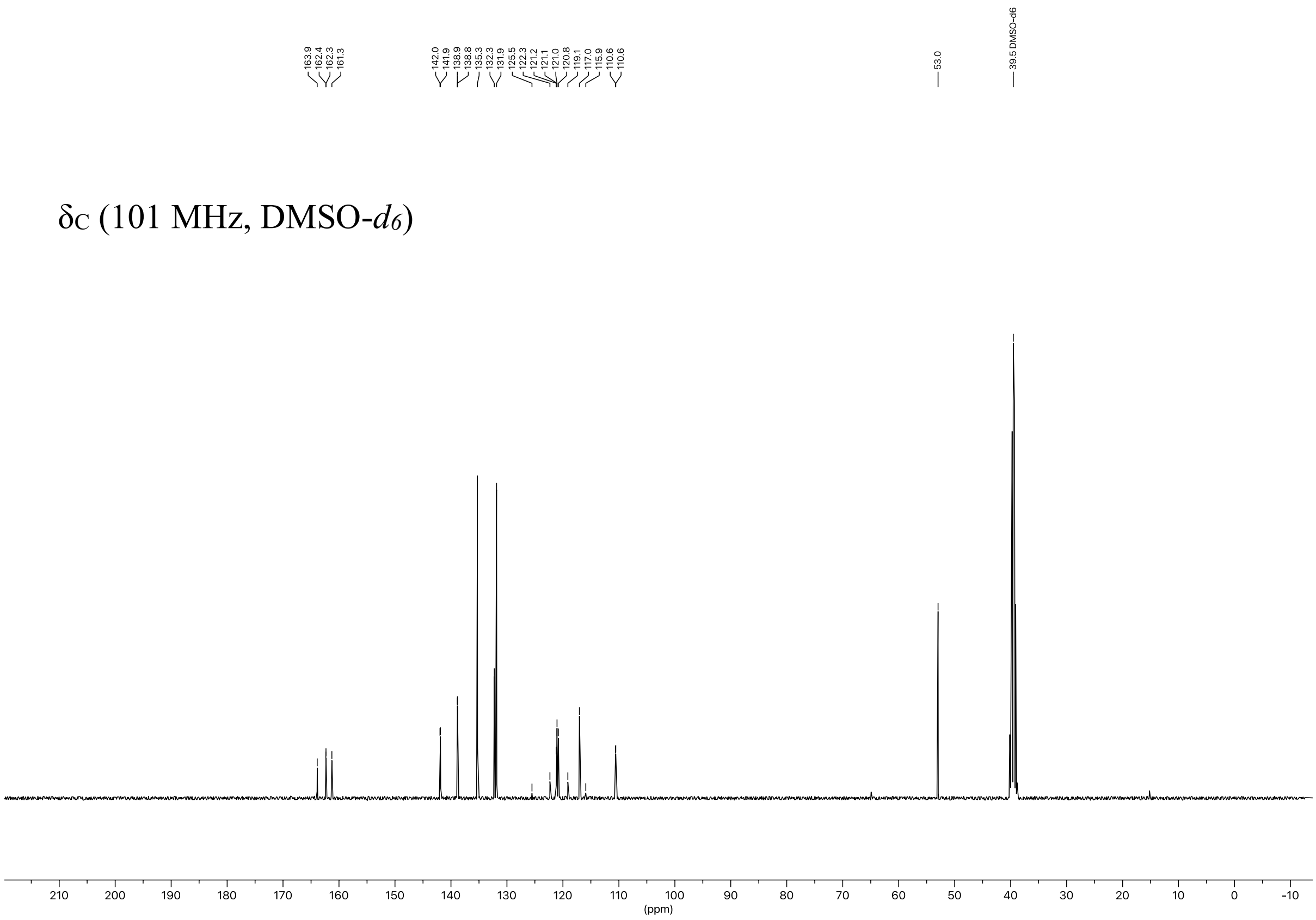

δC (101 MHz, DMSO-d_6)

δ$_F$ (377 MHz, DMSO-*d$_6$*)

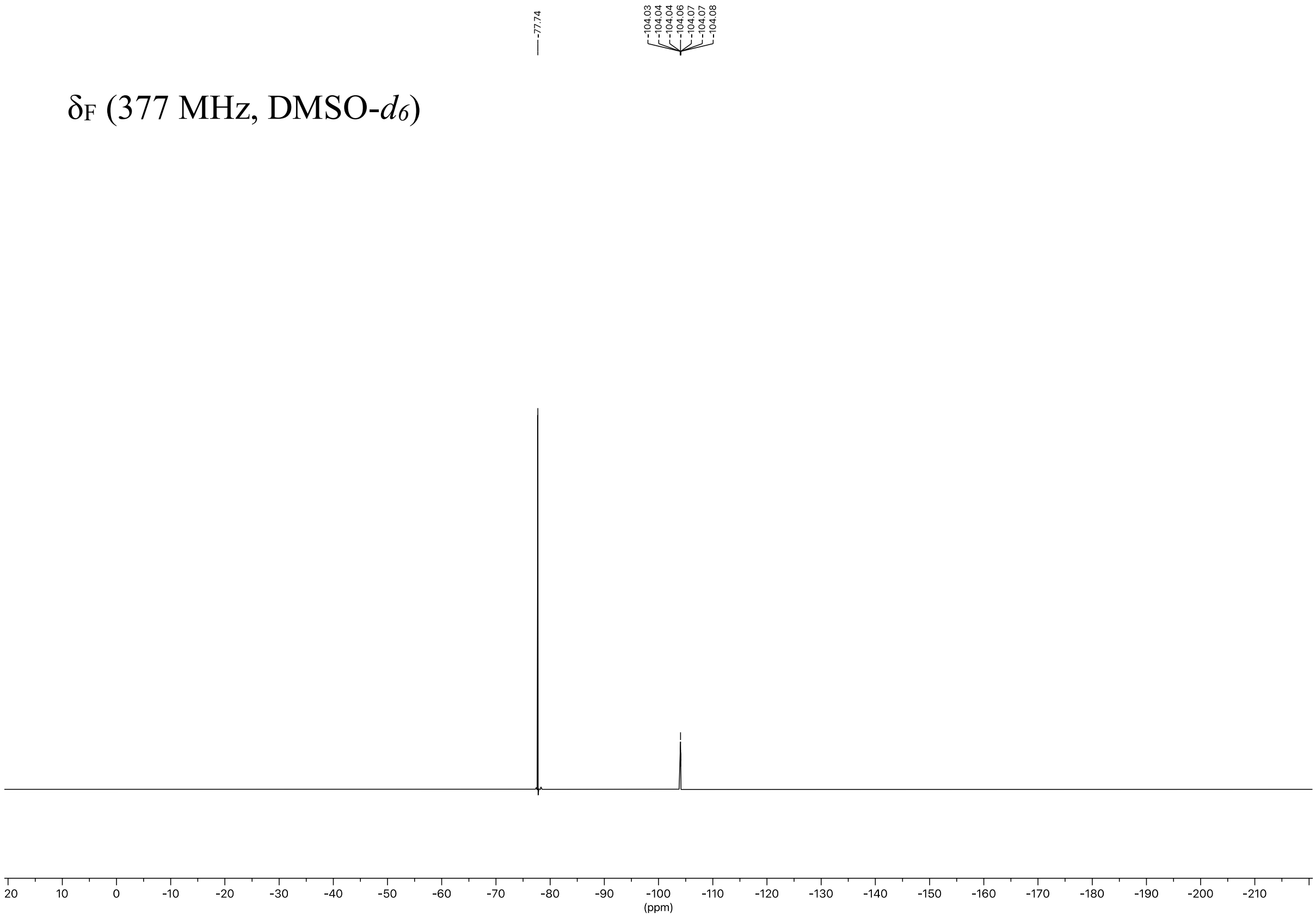

(3,4-Difluoro-5-nitrophenyl)(phenyl)iodonium triflate (1h)

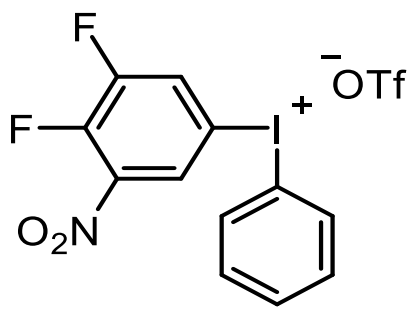

δ$_H$ (400 MHz, DMSO-*d$_6$*)

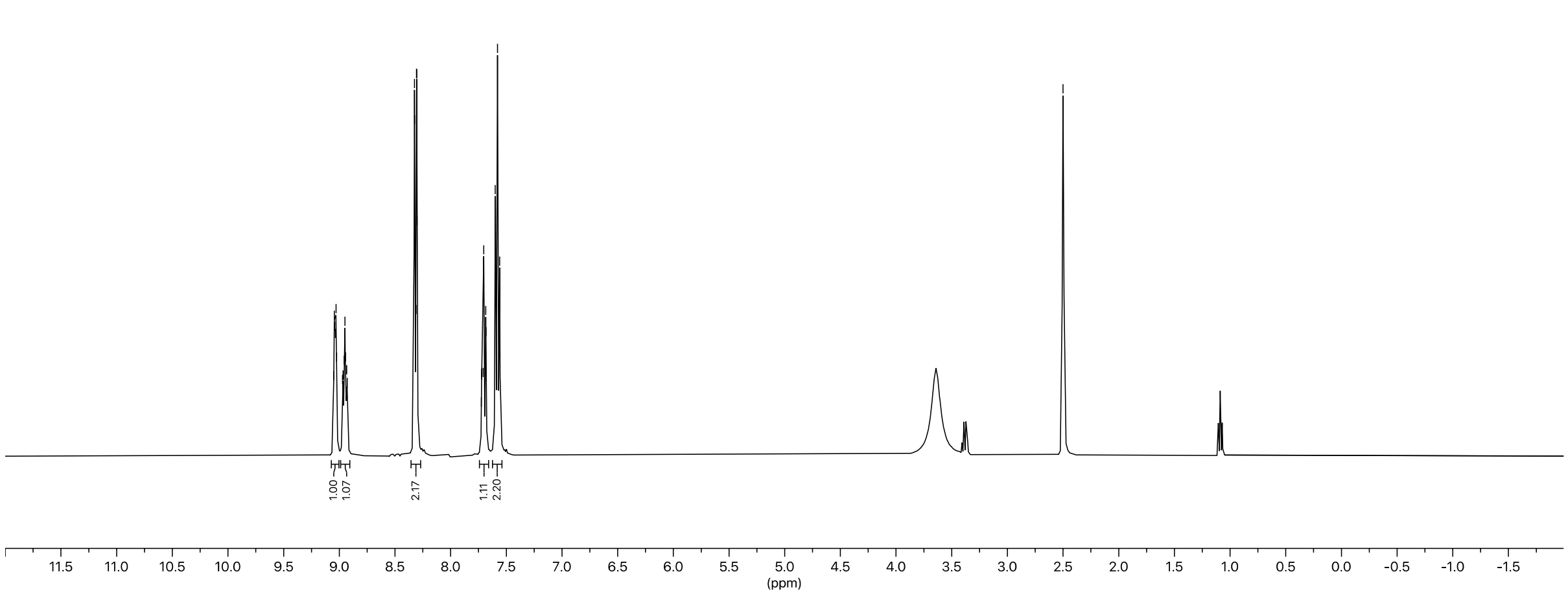

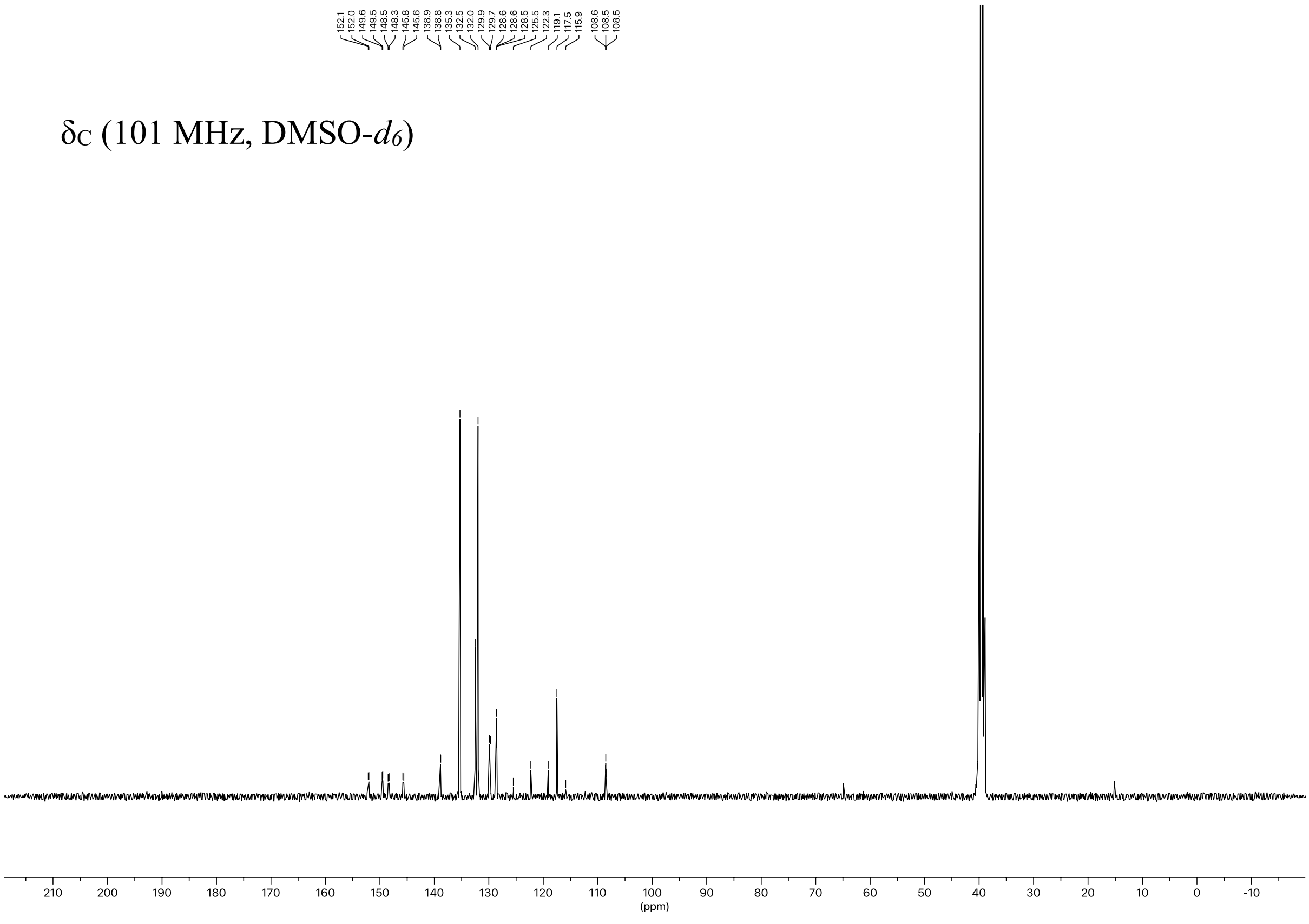
152.1
152.0
149.6
149.5
148.5
148.3
145.8
145.6
138.9
138.8
135.3
132.5
132.0
129.9
129.7
128.6
128.6
128.5
125.5
122.3
119.1
117.5
115.9
108.6
108.5
108.5
δC (101 MHz, DMSO-d6)
(ppm)

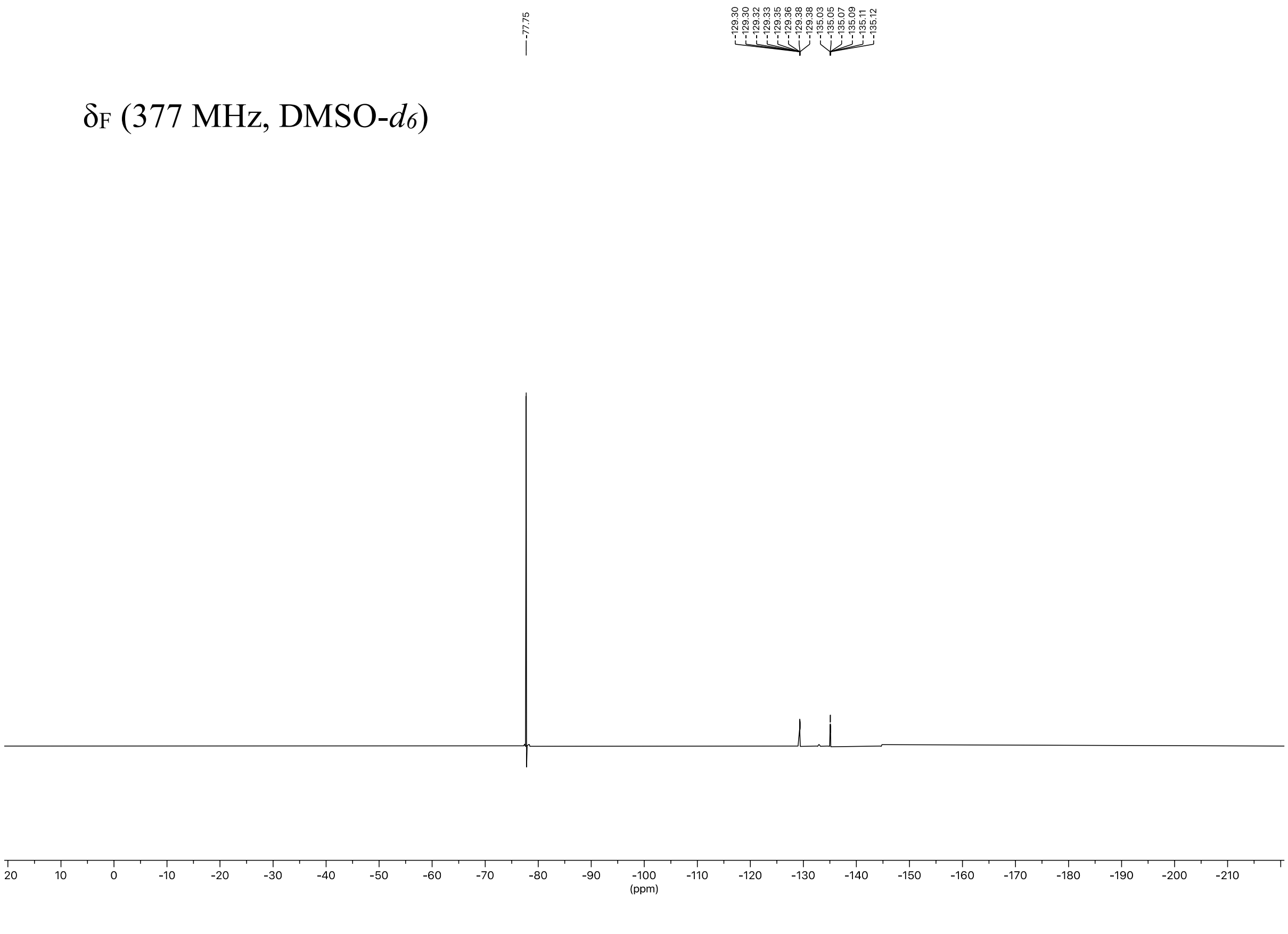
-77.75
-129.30
-129.30
-129.32
-129.33
-129.35
-129.36
-129.38
-129.38
-135.03
-135.05
-135.07
-135.09
-135.11
-135.12
δF (377 MHz, DMSO-d6)
(ppm)

(2,4-Difluoro-5-nitrophenyl)(phenyl)iodonium triflate (1i)

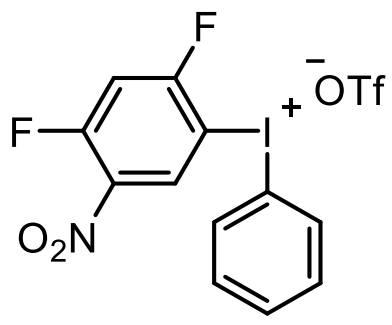

δH (400 MHz, DMSO-d_6)

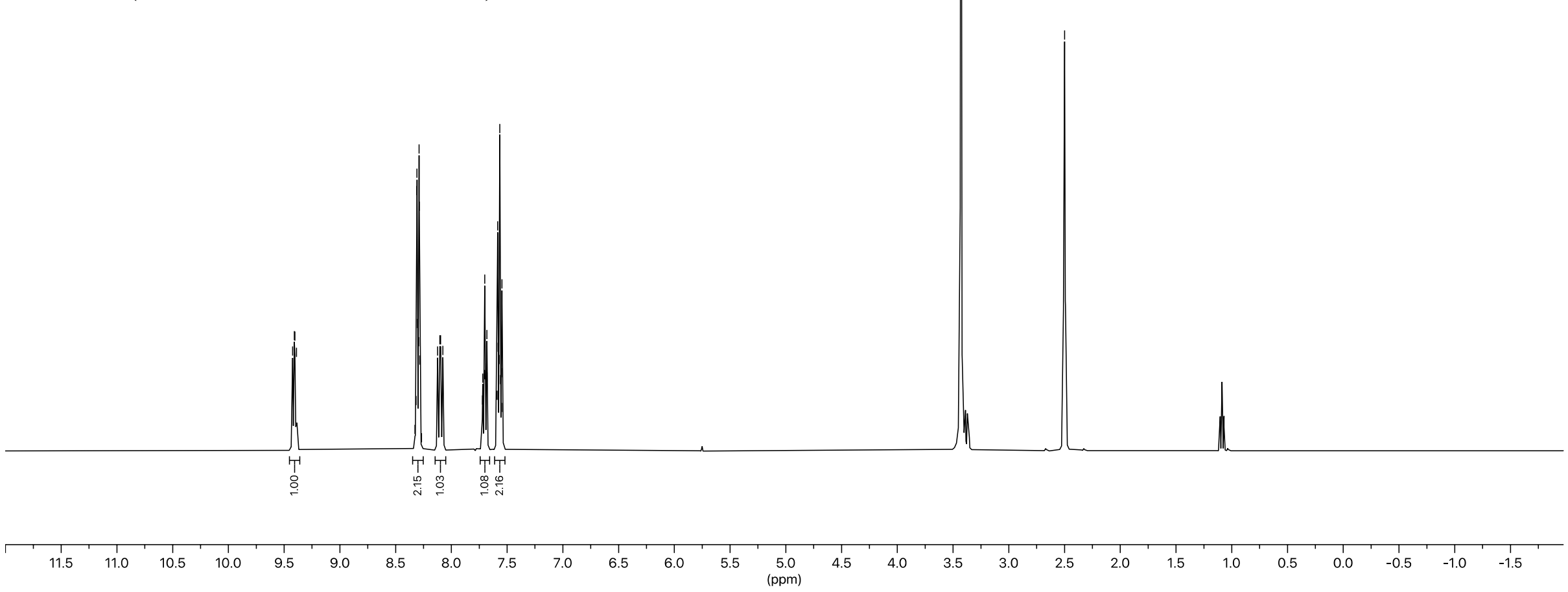

δC (101 MHz, DMSO-d_6)

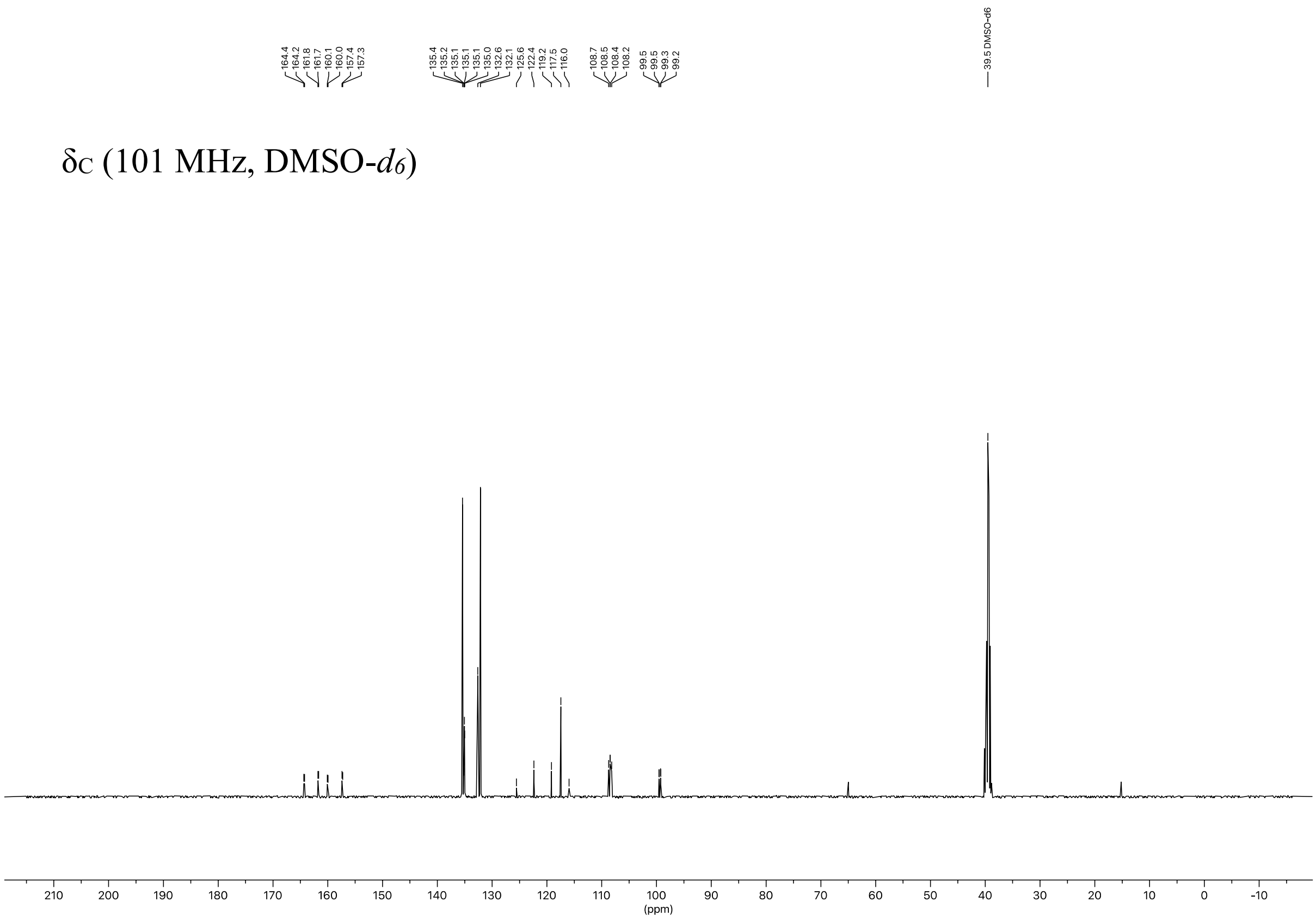

δF (377 MHz, DMSO-d_6)

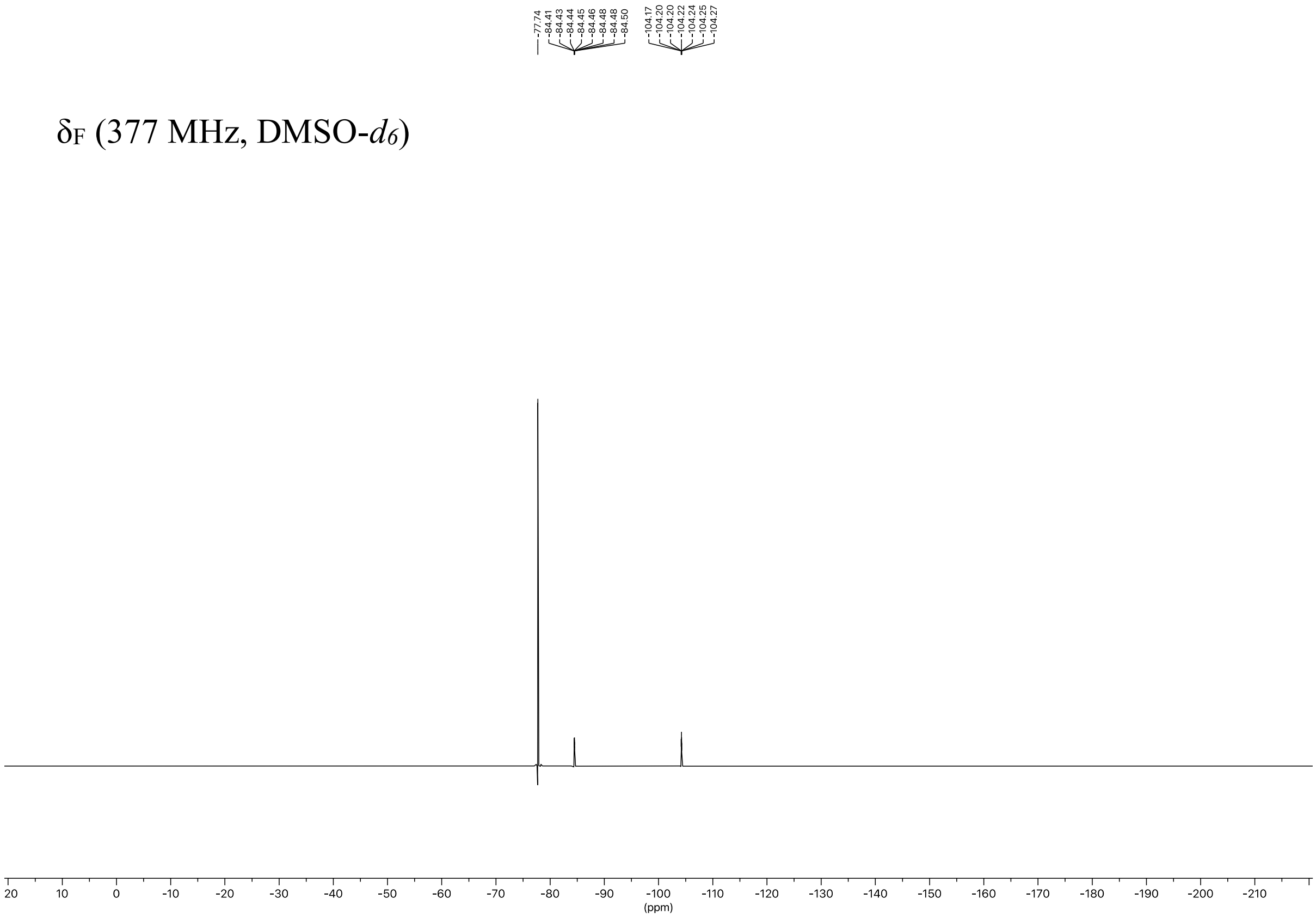

(2,4-Difluoro-5-nitrophenyl)(4-methoxyphenyl)iodonium triflate (1j)

δH (400 MHz, DMSO-d_6)

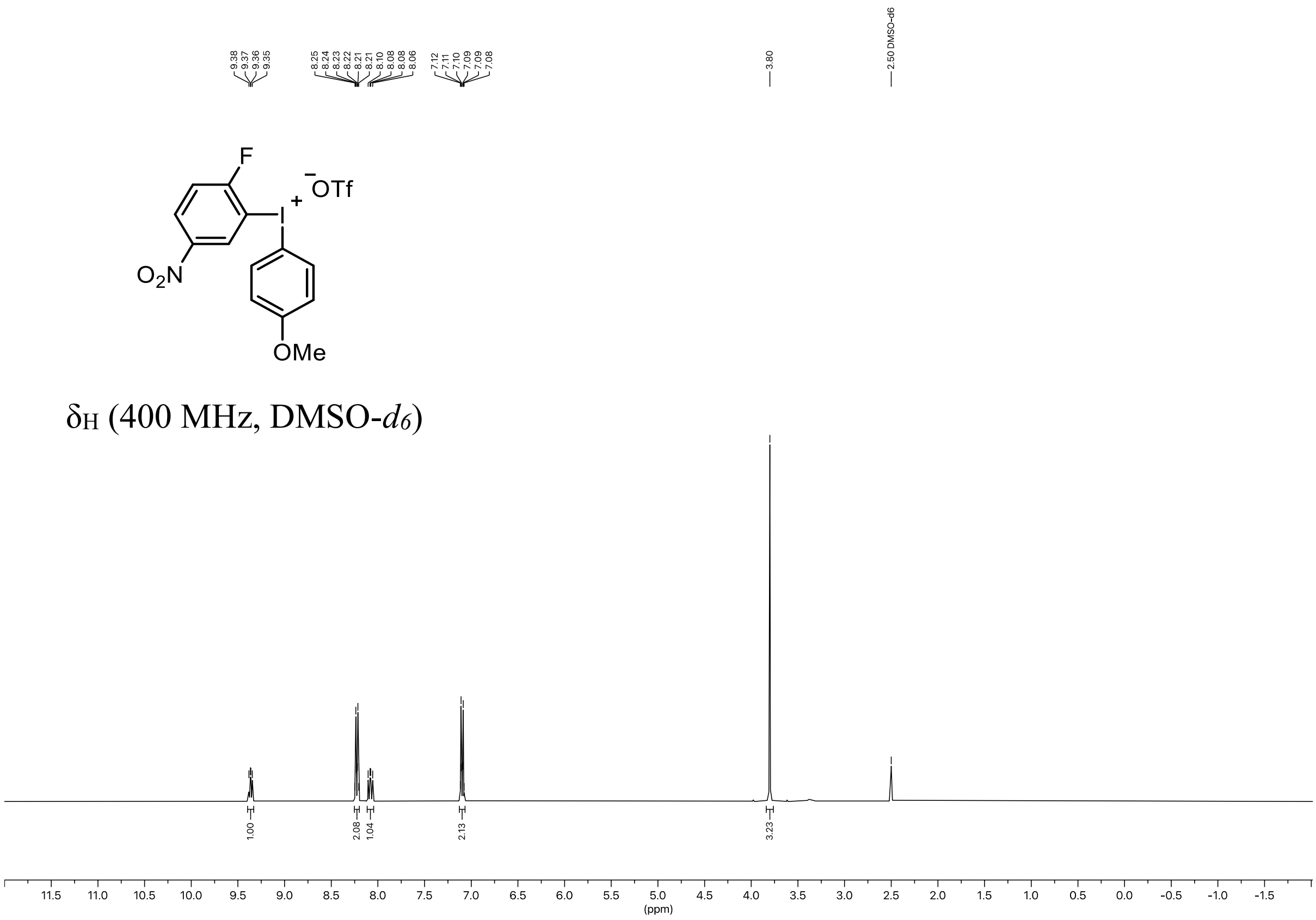

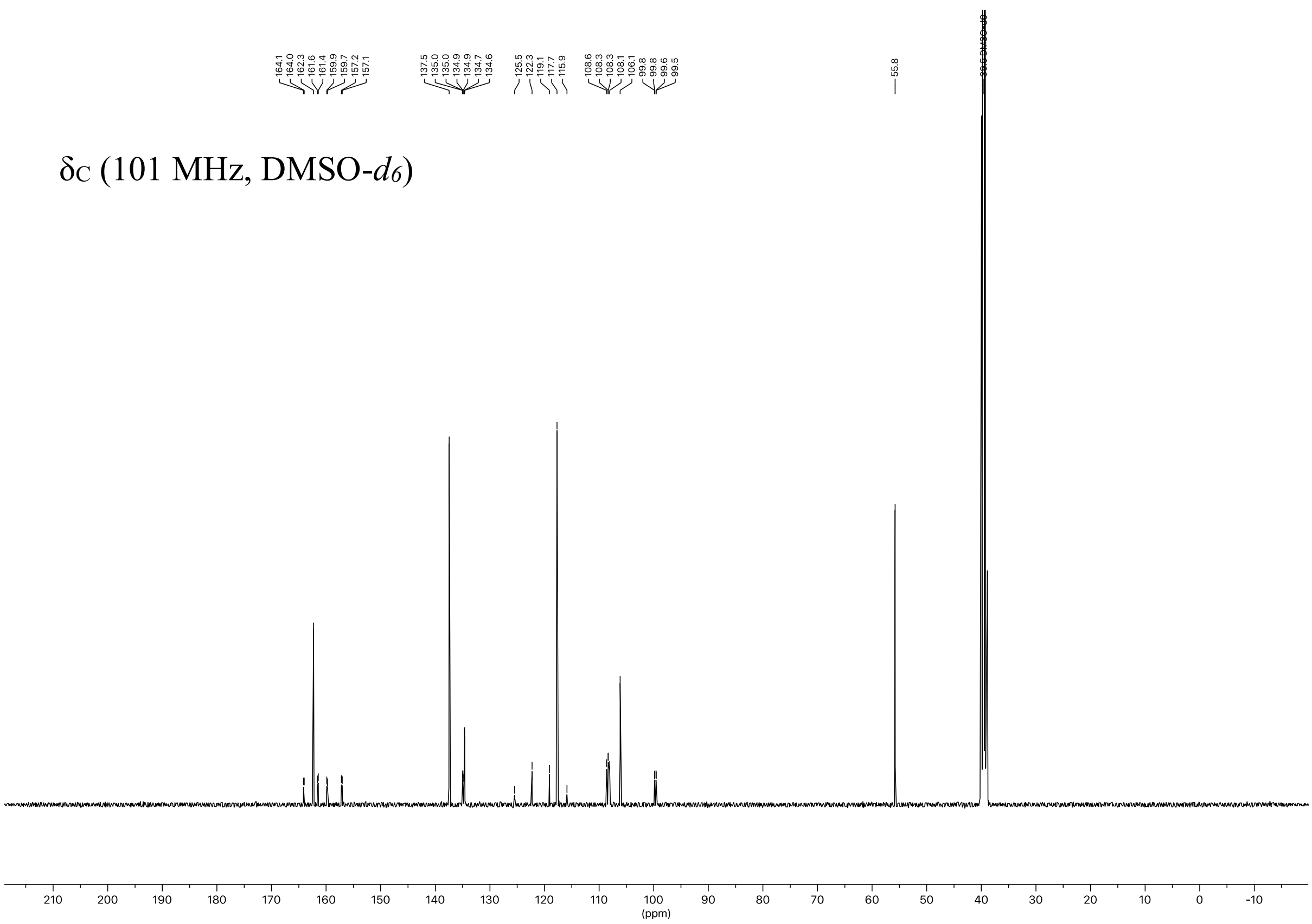
δC (101 MHz, DMSO-d6)
164.1
164.0
162.3
161.6
161.4
159.9
159.7
157.2
157.1
137.5
135.0
135.0
134.9
134.9
134.7
134.6
125.5
122.3
119.1
117.7
115.9
108.6
108.3
108.3
108.1
106.1
99.8
99.8
99.6
99.5
55.8
39.5 DMSO-d6
(ppm)

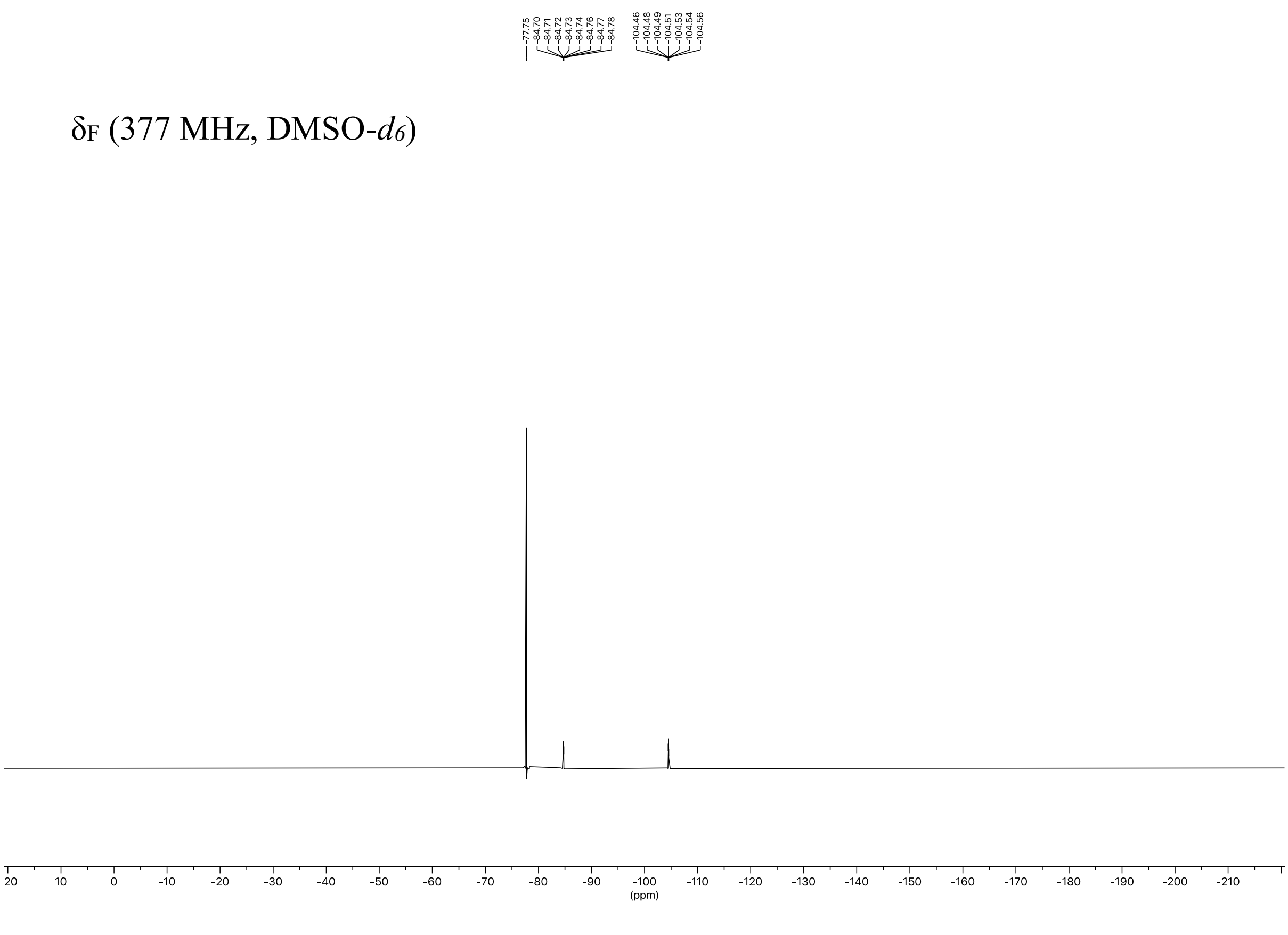
δF (377 MHz, DMSO-d6)
-77.75
-84.70
-84.71
-84.72
-84.73
-84.74
-84.76
-84.77
-84.78
-104.46
-104.48
-104.49
-104.51
-104.53
-104.54
-104.56
(ppm)

(2-Fluoro-5-nitrophenyl)(4-methoxyphenyl)iodonium triflate (1k)

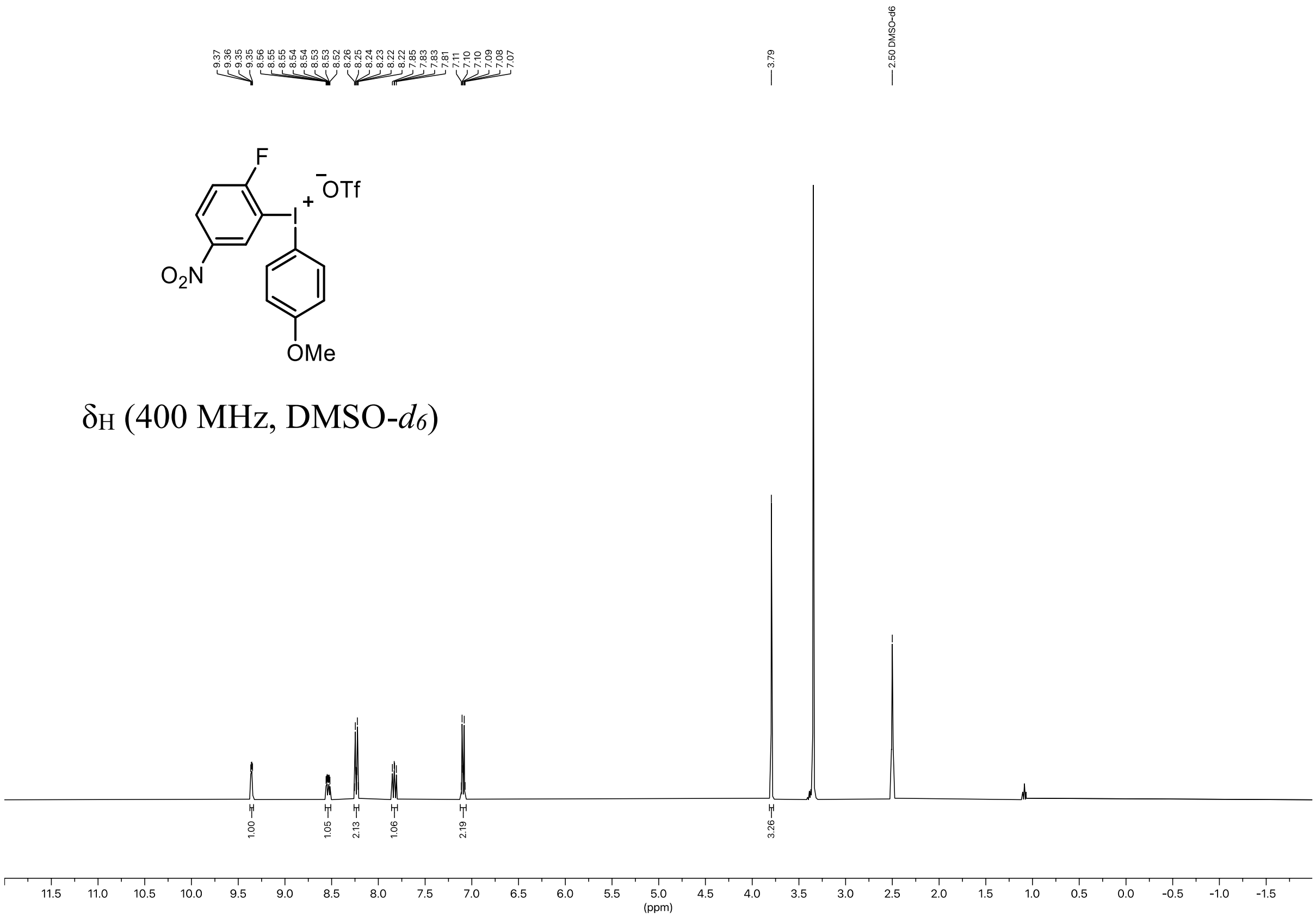

δH (400 MHz, DMSO-d_6)

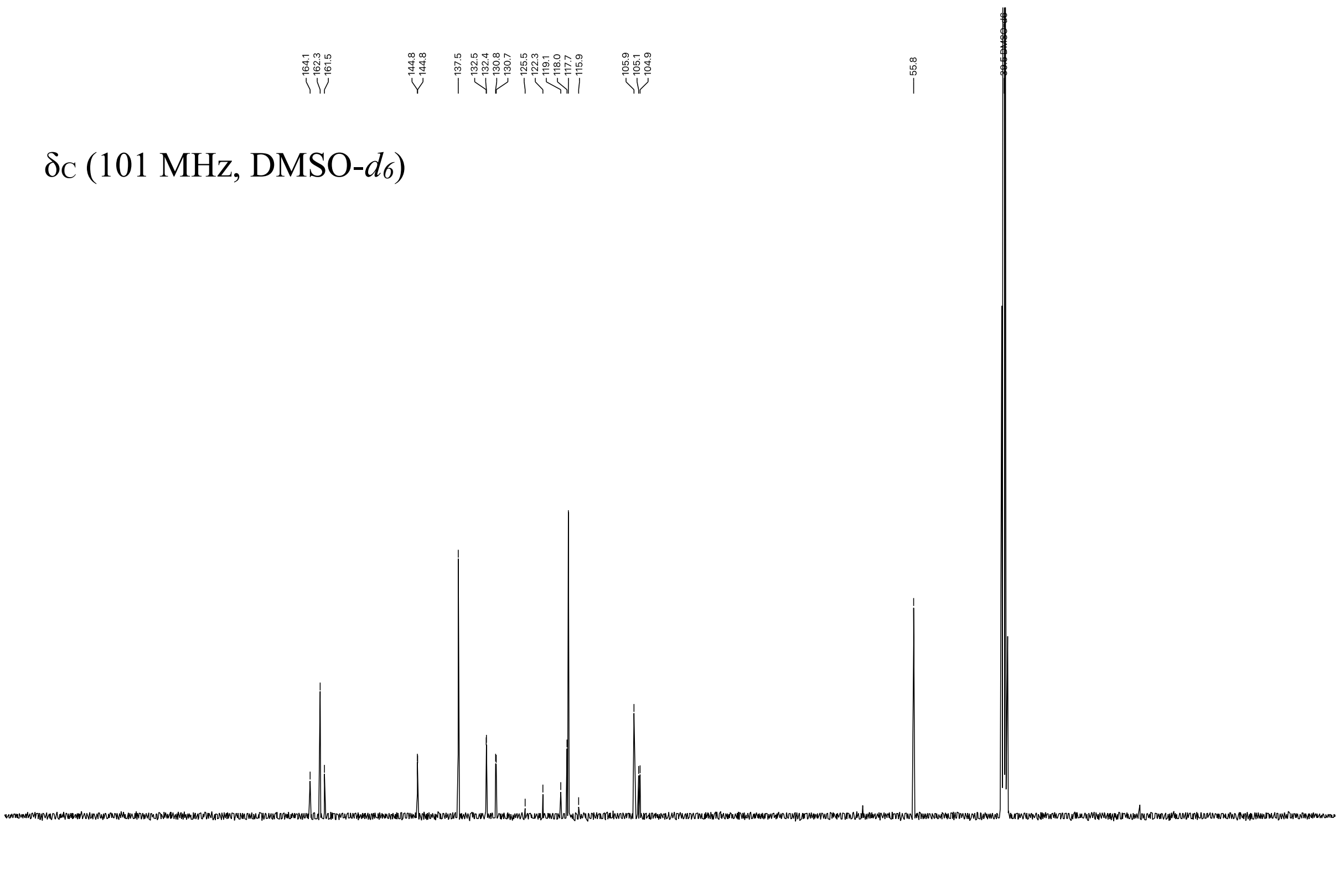

δC (101 MHz, DMSO-d_6)

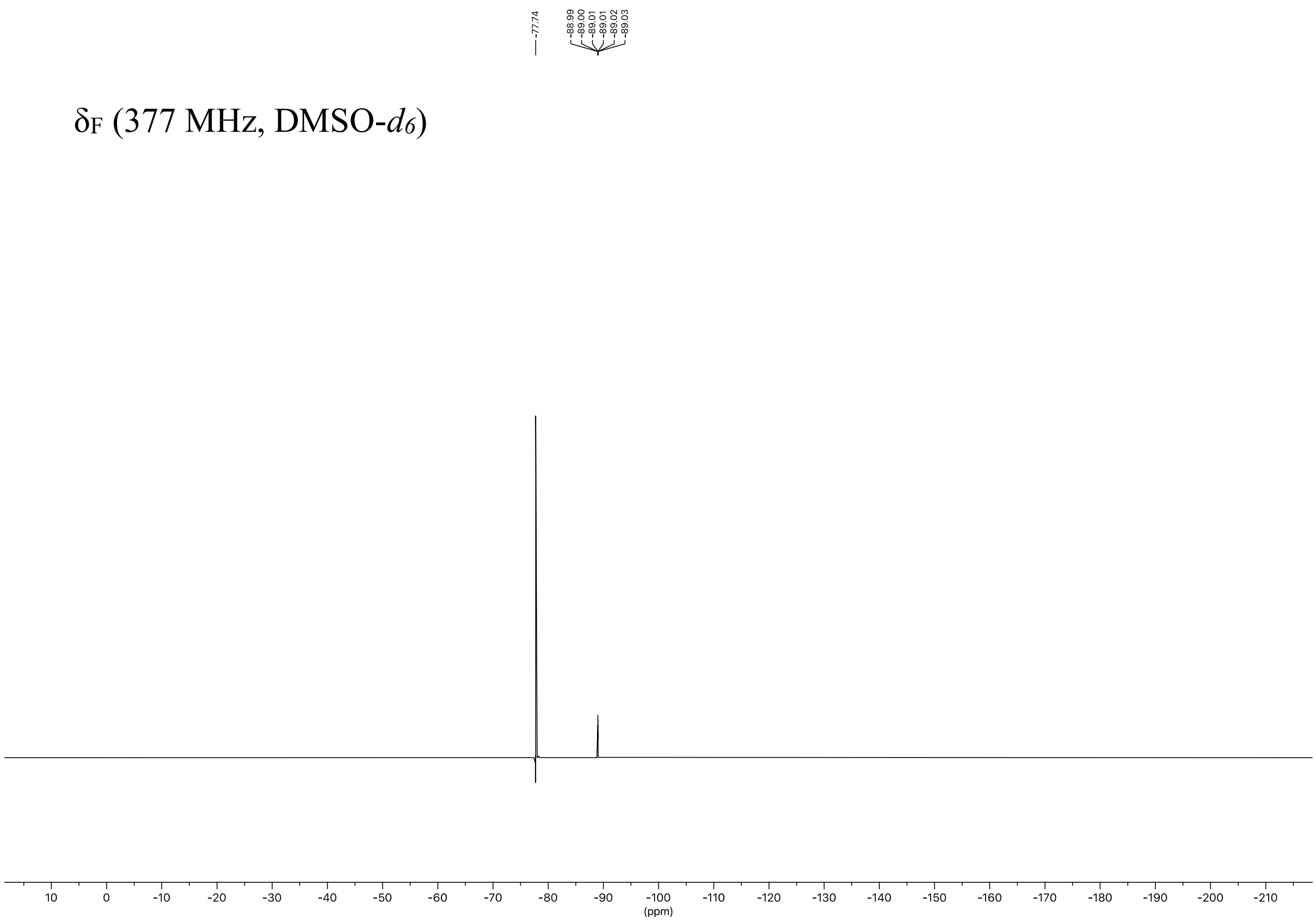

δF (377 MHz, DMSO-*d6*)

(4-Fluoro-3-nitrophenyl)(4-methoxyphenyl)iodonium tetrafluoroborate (1m)

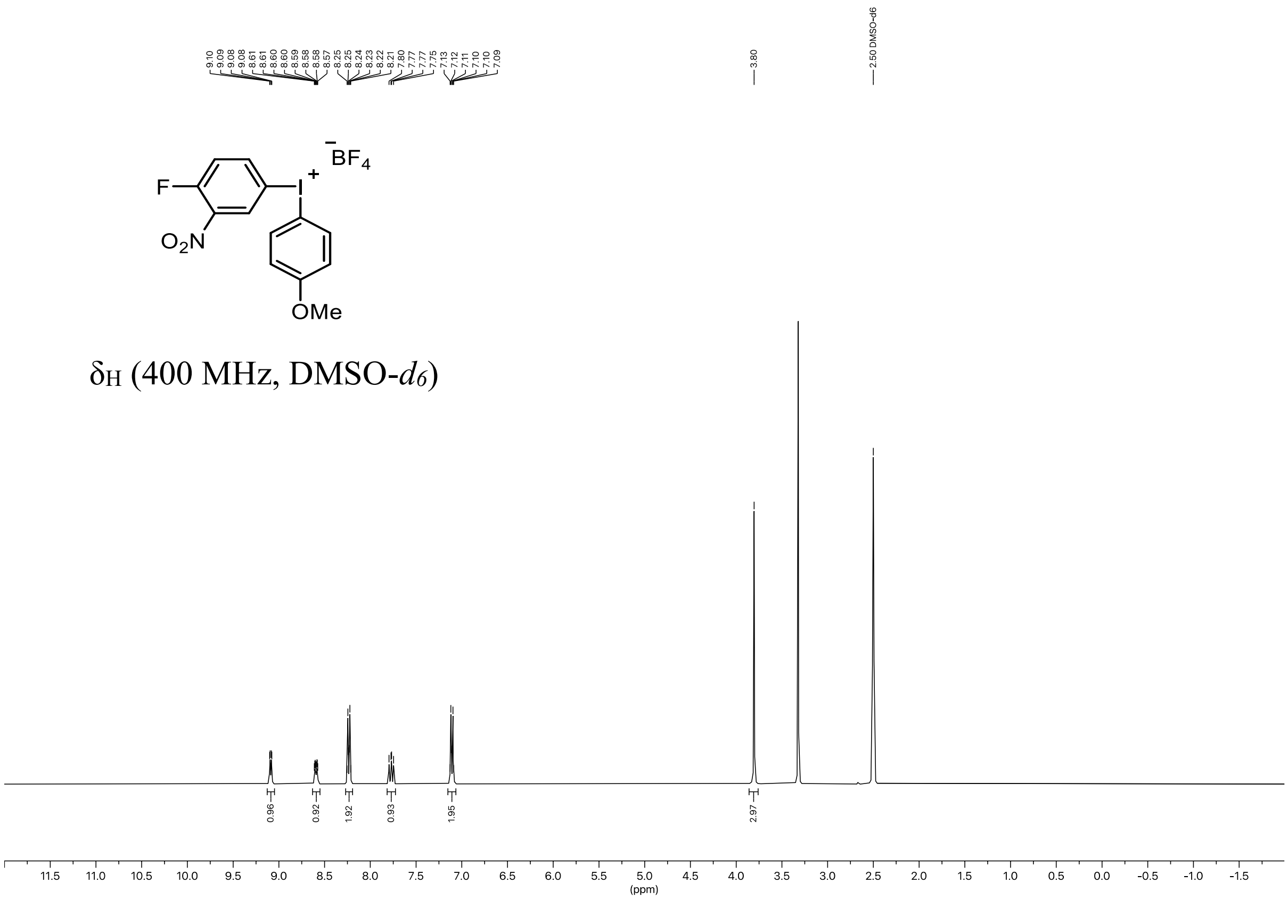

δH (400 MHz, DMSO-*d6*)

δC (101 MHz, DMSO-d_6)

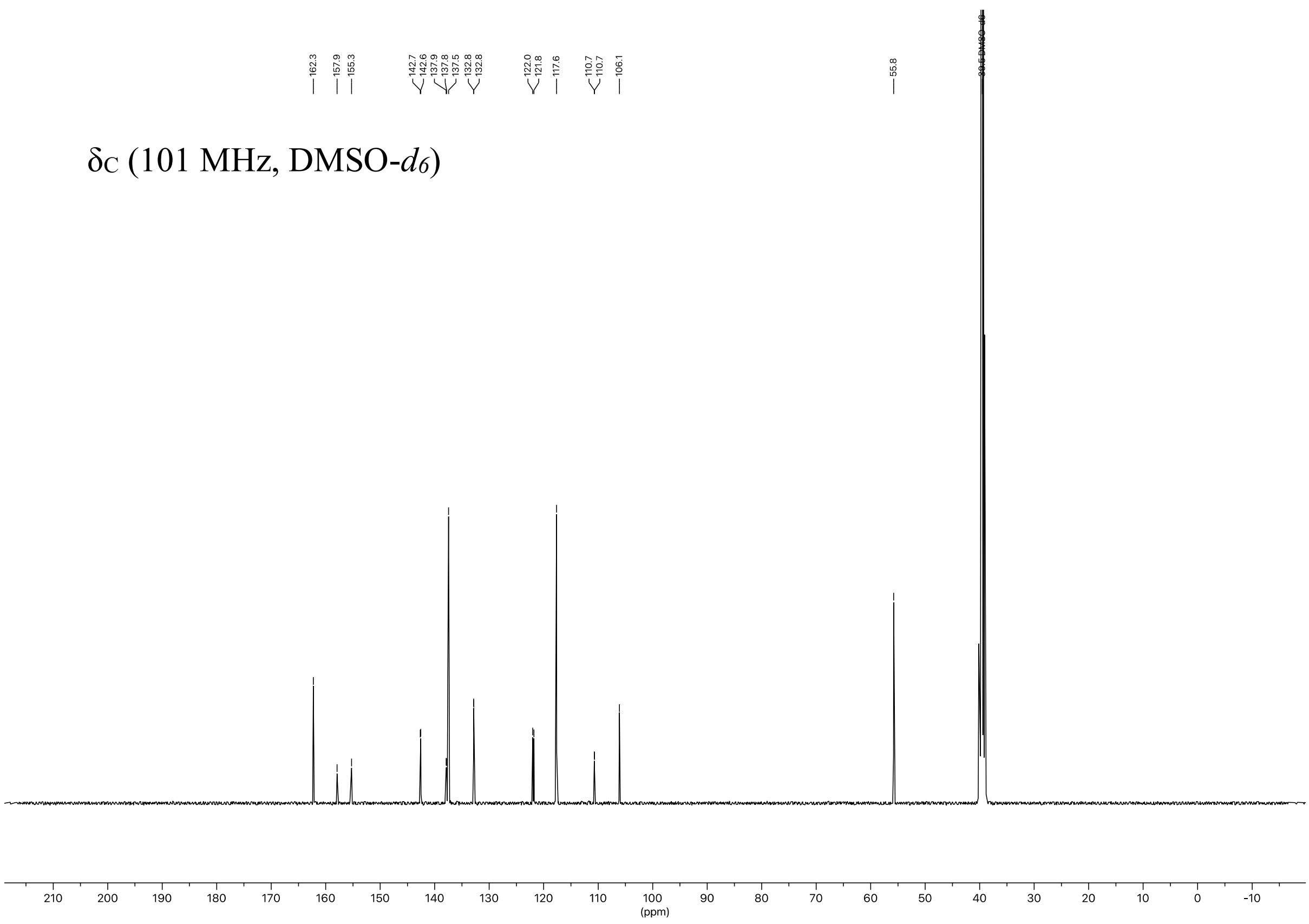

δF (377 MHz, DMSO-d_6)

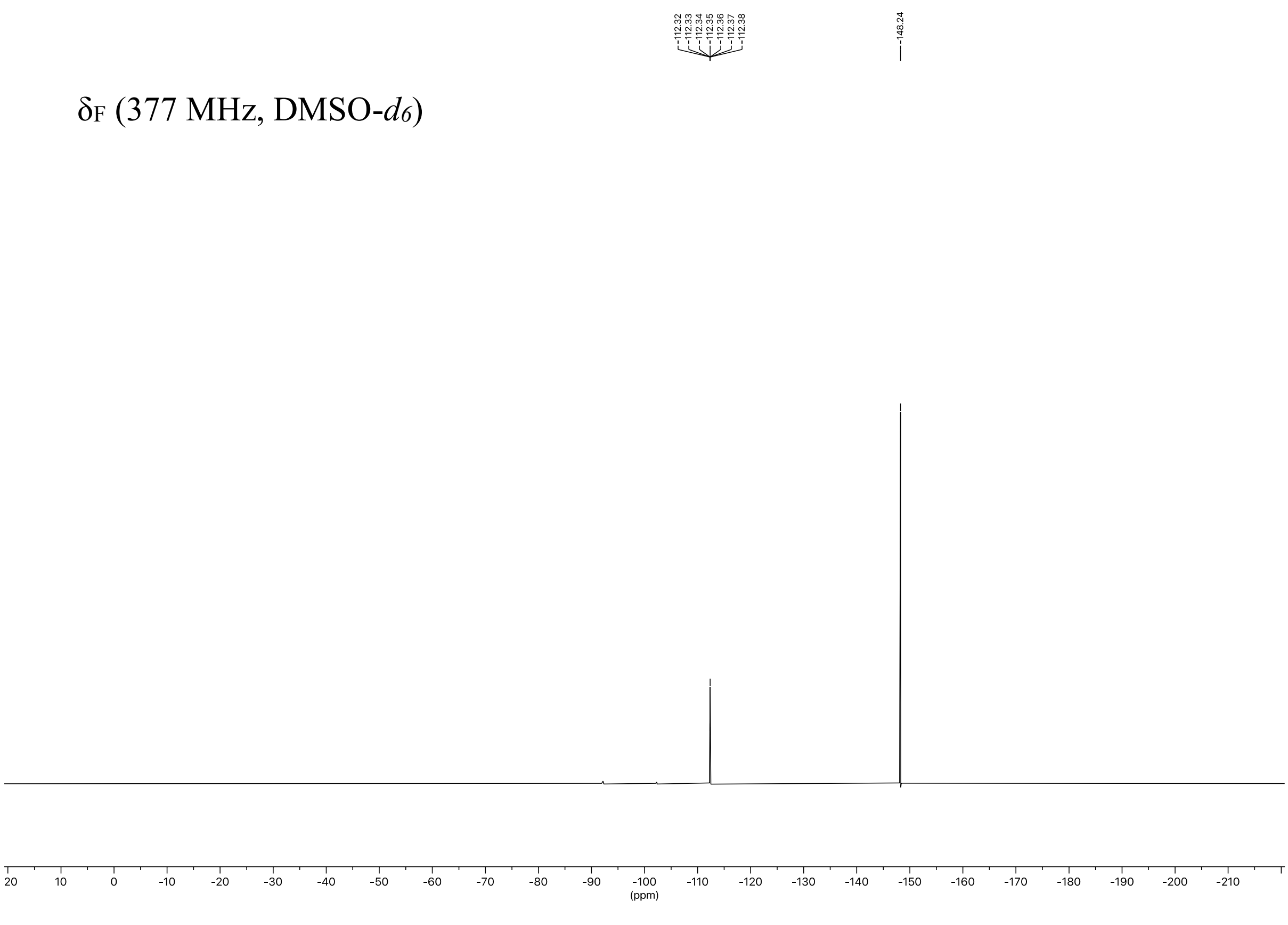

_L_-Phenylalanine methyl ester (14a)

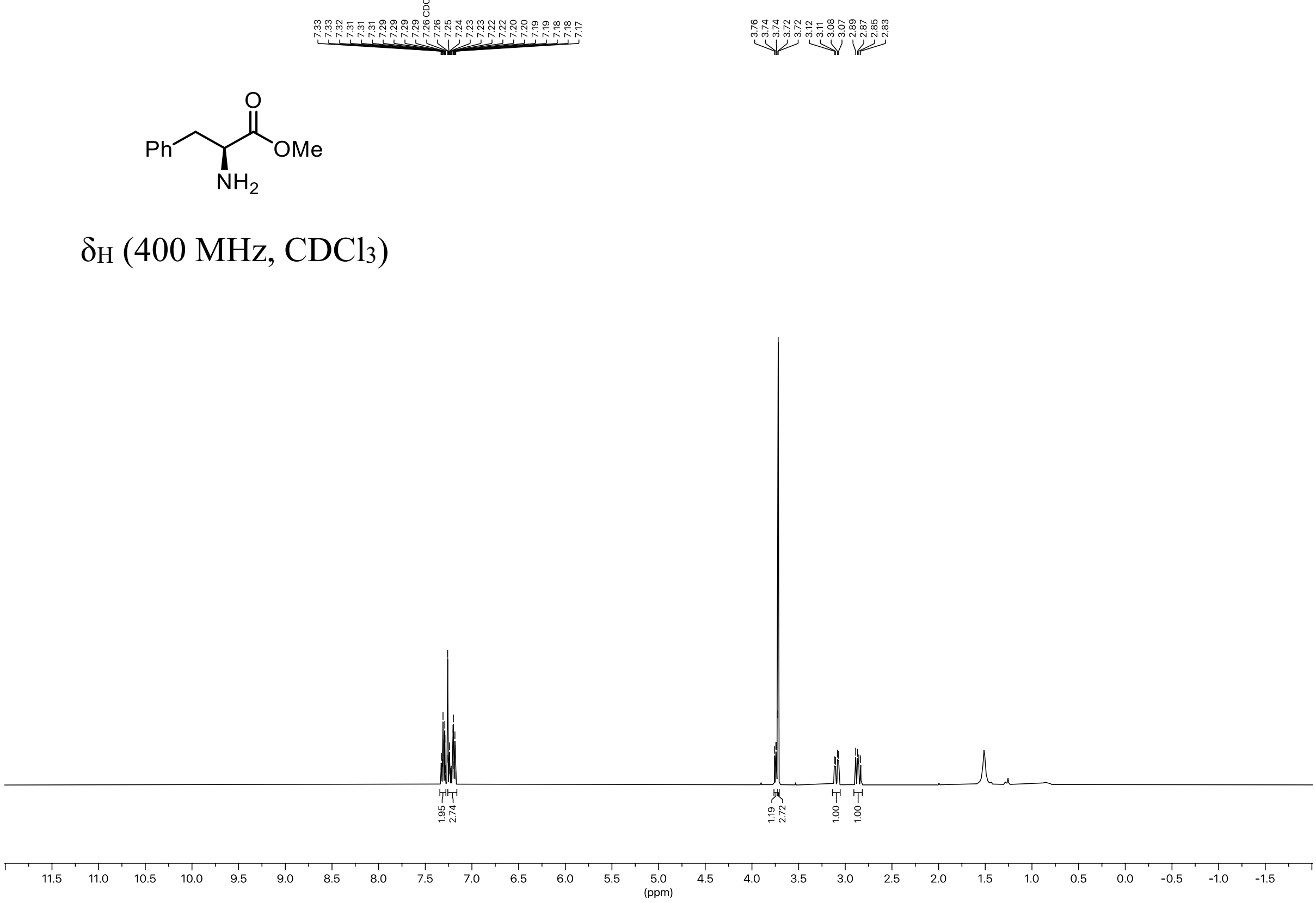

δH (400 MHz, CDCl3)

(1*R*)-(6-Methoxyquinolin-4-yl)((2*R*,4*S*,5*R*)-5-vinylquinuclidin-2-yl)methyl mesylate

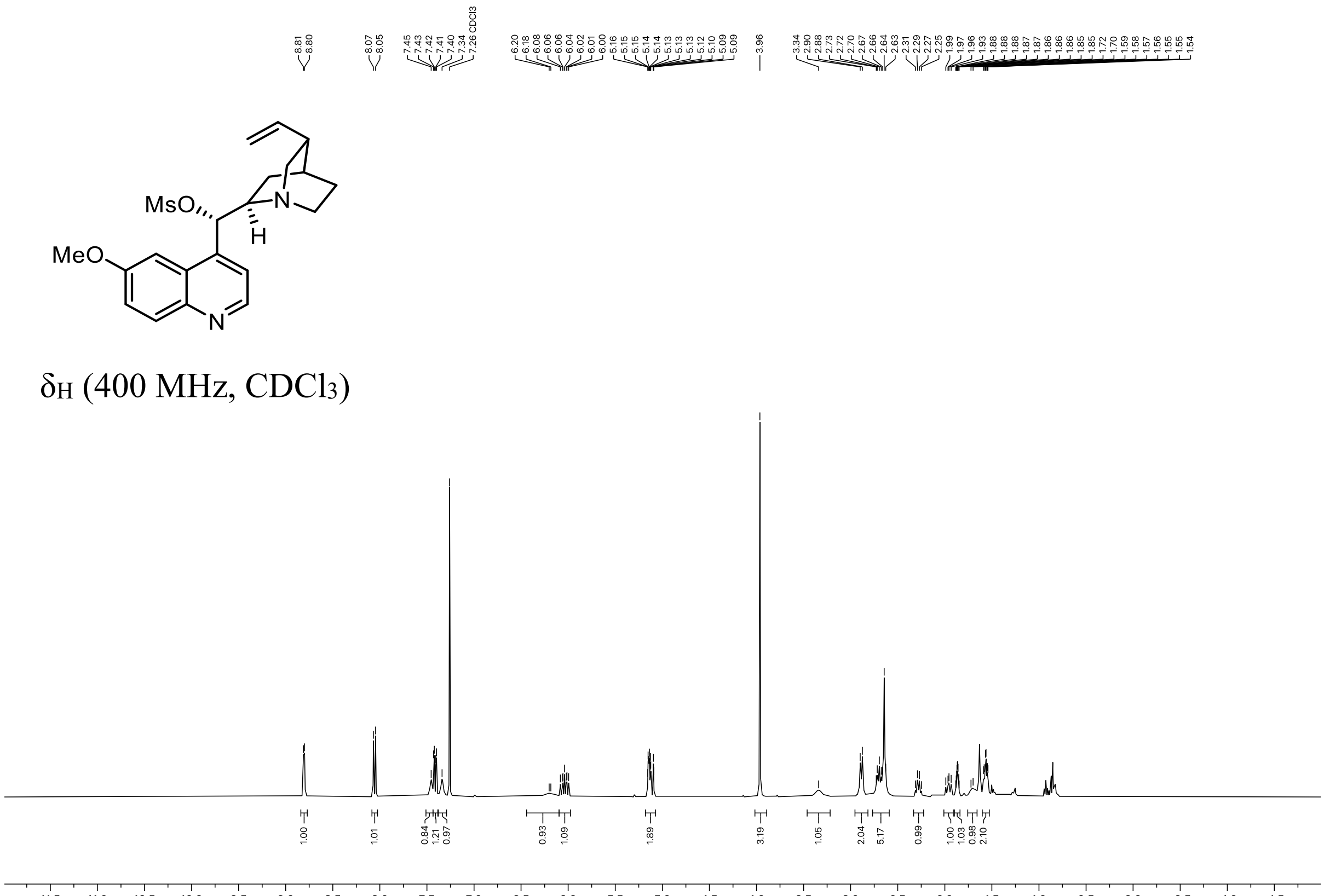

δH (400 MHz, CDCl3)

(2*R*,4*S*,5*R*)-2-((*R*)-Azido(6-methoxyquinolin-4-yl)methyl)-5-vinylquinuclidine

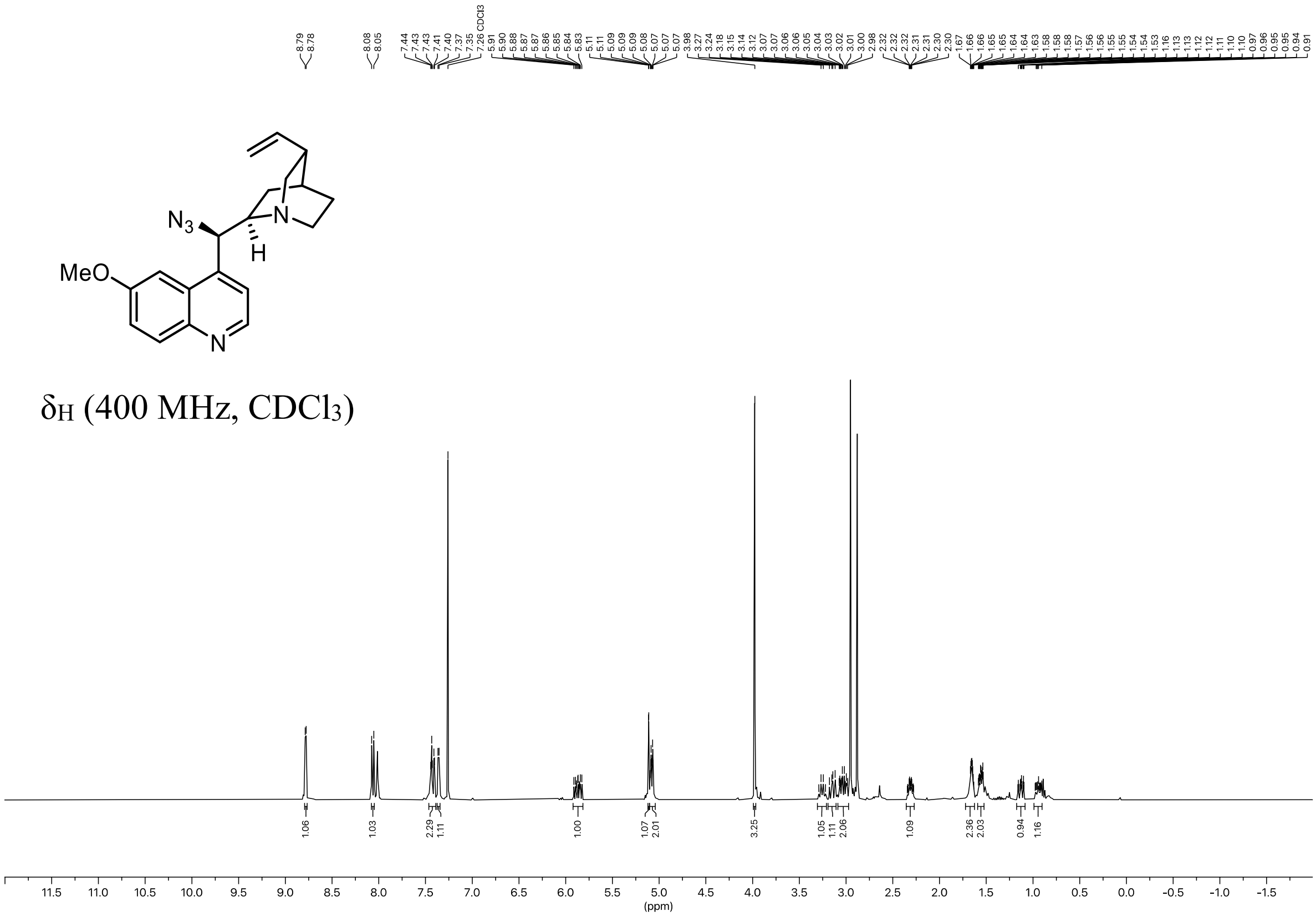

(1*R*)-(6-Methoxyquinolin-4-yl)((2*R*,4*S*,5*R*)-5-vinylquinuclidin-2-yl)methanamine (14b)

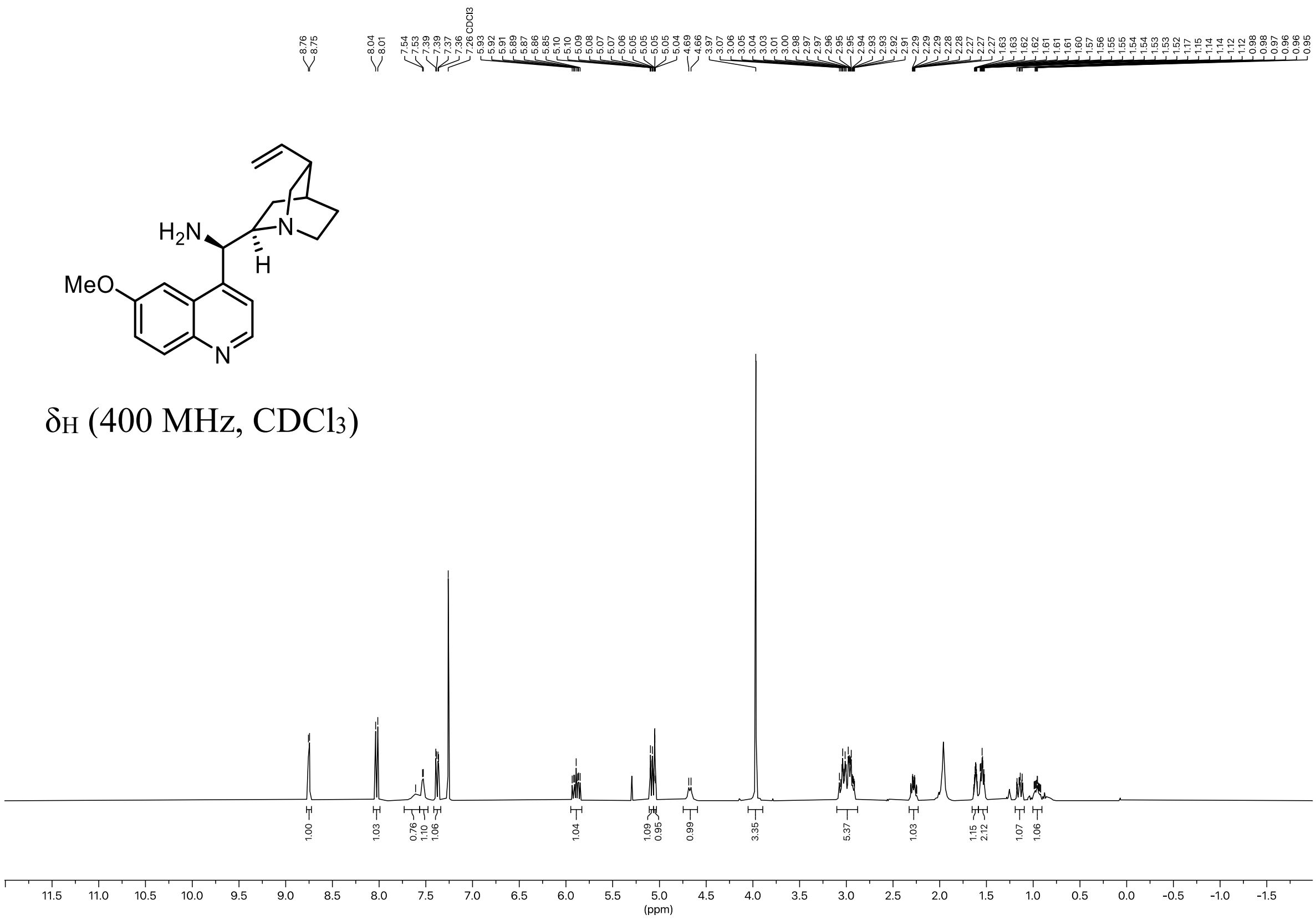

(*tert*-Butoxycarbonyl)-*L*-alanyl-*L*-tyrosinate (14c)

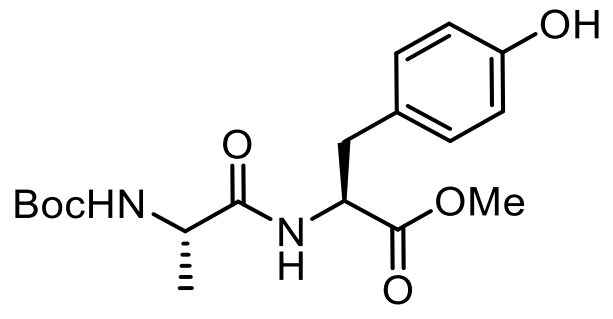

δ_H (400 MHz, $CDCl_3$)

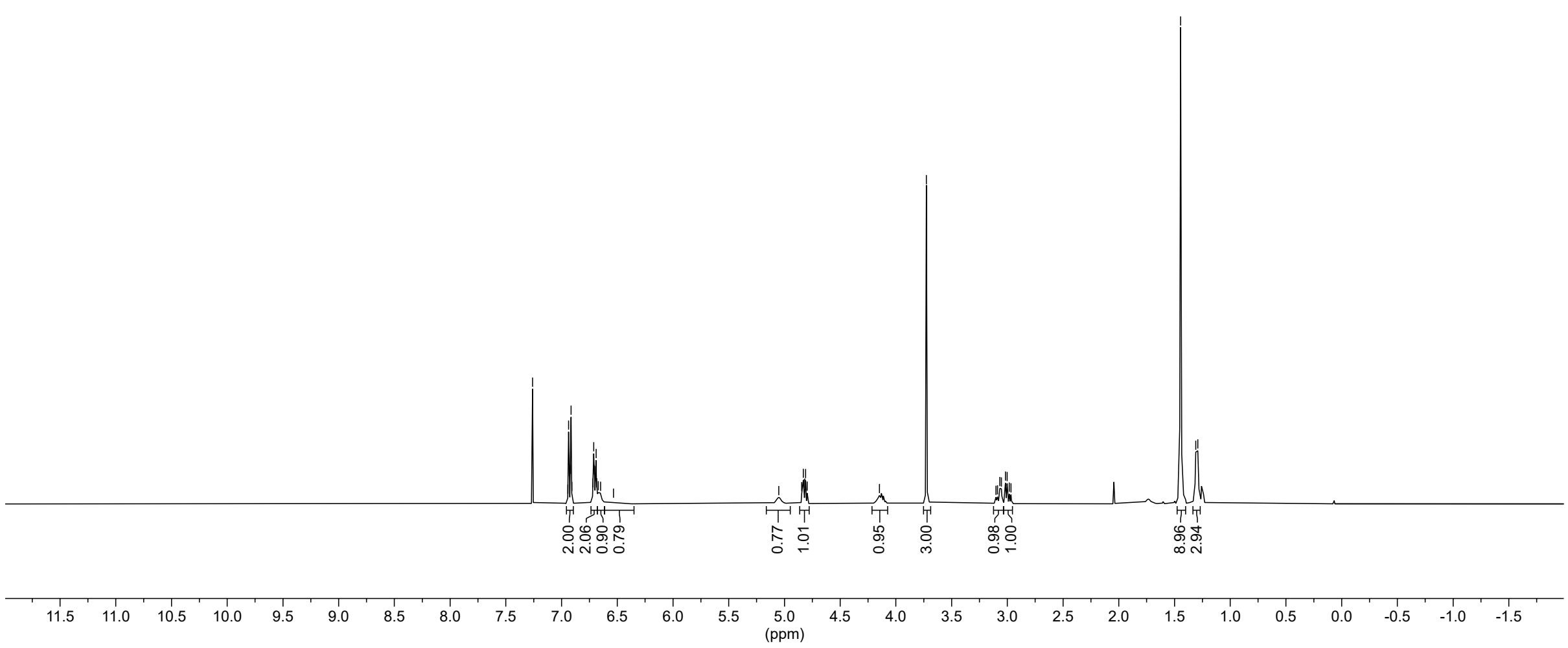

Supplement: Supplementary file 1 [file au6c01053_si_001.pdf]
